# Catalytic N-radical cascade reaction of hydrazones by oxidative deprotonation electron transfer and TEMPO mediation

**DOI:** 10.1038/ncomms11188

**Published:** 2016-04-06

**Authors:** Xiao-Qiang Hu, Xiaotian Qi, Jia-Rong Chen, Quan-Qing Zhao, Qiang Wei, Yu Lan, Wen-Jing Xiao

**Affiliations:** 1 CCNU-uOttawa Joint Research Centre, Key Laboratory of Pesticide and Chemical Biology, Ministry of Education, College of Chemistry, Central China Normal University, 152 Luoyu Road, Wuhan 430079, China; 2 School of Chemistry and Chemical Engineering, Chongqing University, Chongqing 400030, China

## Abstract

Compared with the popularity of various C-centred radicals, the N-centred radicals remain largely unexplored in catalytic radical cascade reactions because of a lack of convenient methods for their generation. Known methods for their generation typically require the use of N-functionalized precursors or various toxic, potentially explosive or unstable radical initiators. Recently, visible-light photocatalysis has emerged as an attractive tool for the catalytic formation of N-centred radicals, but the pre-incorporation of a photolabile groups at the nitrogen atom largely limited the reaction scope. Here, we present a visible-light photocatalytic oxidative deprotonation electron transfer/2,2,6,6-tetramethylpiperidine-1-oxyl (TEMPO)-mediation strategy for catalytic N-radical cascade reaction of unsaturated hydrazones. This mild protocol provides a broadly applicable synthesis of 1,6-dihydropyradazines with complete regioselectivity and good yields. The 1,6-dihydropyradazines can be easily transformed into diazinium salts that showed promising *in vitro* antifungal activities against fungal pathogens. DFT calculations are conducted to explain the mechanism.

## Abstract (web summary)

The use of N-centred radicals is limited by the difficulties in their preparation. Here, the authors report a photoredox method for the formation of N-centred radicals on hydrazones and their use for the synthesis of heterocyclic compounds through a radical cascade reaction.

## Graphical abstract

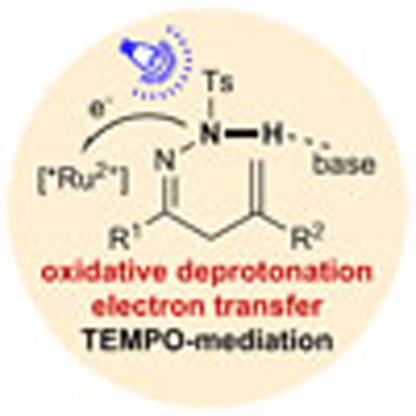

Synthetic chemists continuously strive for fast, selective and high yielding reactions under mild conditions. Radical reactions, especially the radical cascades, provide a potential access to such ideal transformations and have attracted considerable attention of synthetic community because of their typically mild conditions, short reaction times and high efficiency^1^,^2^. Although various carbon radicals have been widely used in catalytic radical-based cascade reactions^3^,^4^,^5^, however, the chemistry of N-centred radicals in this regard remains largely unexplored because of a lack of convenient and general methods for their generation^6^,^7^. Known methods for their generation typically require the use of N-functionalized precursors or various toxic, potentially explosive or unstable radical initiators. Pioneered by Nicolaou's discovery of the *o*-iodoxybenzoic acid-mediated conversion of *N*-aryl amides and carbamates into the corresponding nitrogen radicals^8^, the groups of Chiba^9^ and Lei^10^, respectively, developed two efficient methods for the generation of 1,3-diazaallyl and amidyl radicals by Cu- and Ni-catalyzed oxidative cleavage of N–H bonds of amidines and *N*-alkoxyamides using O_2_ and di-tertiary butyl peroxide as terminal oxidants at high temperatures. Recently, Li^11^ and Chemler^12^ also independently reported the Cu- and Ag-catalyzed oxidative formation of amidyl radicals in the presence of stoichiometric MnO_2_ and Selectfluor reagents as oxidants. Despite these impressive advancements, the search for new efficient protocols for direct catalytic conversion of the N–H bonds into the corresponding N-centred radicals under mild conditions has become an increasingly significant, yet challenging priority in the development of new N-radical cascade reactions.

In recent years, the visible-light photocatalysis has been established as a powerful technique that facilitates selectively activating organic molecules and chemical bonds to indentify new chemical reactions under mild conditions^13^,^14^,^15^,^16^. As the notable early studies by MacMillan^17^ and Sanford^18^ on neutral N-centred radical-mediated photocatalytic C–H amination of aldehydes and (hetero)arenes, several promising visible-light photocatalytic protocols have been developed by other groups for generating N-centred radicals and C–N bond formation (Fig. 1a)^19^,^20^,^21^,^22^,^23^. Despite their advantages, these methods require the introduction of a photolabile substituent at the nitrogen atom as a handle for photo-activation. The use of the visible-light photocatalysis in initiating strong N–H bond activation and application in neutral N-centred radical-mediated catalytic cascade reactions have been, until recently, very limited. The Knowles' group recently reported an elegant combination of iridium photocatalyst and phosphate base for a direct homolytic cleavage of strong N–H bonds of *N*-arylamides to access amidyl radicals by a concerted proton-coupled electron transfer, which allowed an efficient radical cascade reaction towards N-heterocycle synthesis^24^. Exploring new reactivity of N-containing compounds in the field of visible-light photocatalysis is an integral part of our recent ongoing research endeavours^25^,^26^,^27^,^28^. For example, our group has recently developed a direct catalytic conversion of the N–H bonds of β,γ-unsaturated hydrazones into N-centred hydrazonyl radicals by visible-light-induced photoredox catalysis, which enables an efficient and mild approach to intramolecular alkene hydroamination and oxyamination for synthesis of 4,5-dihydropyrazole derivatives^28^. In this reaction, a highly regioselective 5-exo radical cyclization of an N-centred radical was observed. It should be noted that the groups of Han^29^,^30^ and Chiba^31^ have also independently reported stoichiometric amounts of tetramethylpiperidine-1-oxyl (TEMPO)-mediated intramolecular cyclization of hydrazonyl radicals for pyrazoline synthesis. Inspired by these studies, we considered exploration of the reactivity of hydrazones in catalytic N-radical cascade reactions to assemble biologically and synthetically important dihydropyradazine scaffolds^32^, inaccessible using other thermal methods^29^,^30^,^31^,^33^ or our own previous protocols.

To this end, herein, we report an oxidative deprotonation electron transfer (ODET)/TEMPO-mediation strategy for direct N–H bond activation and catalytic N-radical cascade reactions of unsaturated hydrazones (Fig. 1b). This mild protocol represents the first, to our knowledge, broadly applicable synthesis of 1,6-dihydropyradazines with good regioselectivity and yield, achieved by merge of visible-light photocatalysis and TEMPO mediation.

## Results

### Reaction design

**Figure 1:**
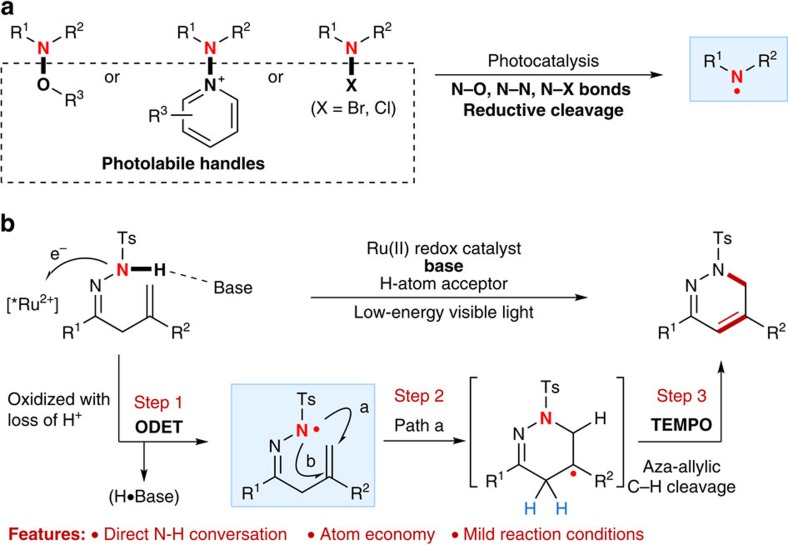
Reaction design. (**a**) Visible-light-induced photocatalytic generation of N-centred radicals from N-functionalized precursors. (**b**) Our blueprint for catalytic N-radical cascade reaction of hydrazones: merge of oxidative deprotonation electron transfer (ODET) activation of N–H bond with TEMPO mediation.

To realize the target catalytic N-radical cascade reaction of unsaturated hydrazones as shown in Fig. 1b, several major challenges would probably be encountered, such as the controlled formal homolysis of the recalcitrant N–H bond for the formation of the neutral N-centred hydrazonyl radical, regioselectivity of the N-radical cyclization step (for example, 6-*endo* and 5-*exo*, path a versus path b)^34^,^35^ and selective homolytic activation of aza-allylic C–H bond in C-centred radical intermediate. Notably, it has been recently documented by MacMillan^36^,^37^, Knowles^24^,^38^,^39^ and our group^28^,^40^ that the addition of a suitable Brønsted acid, Lewis acid or base could facilitate some otherwise inaccessible photocatalytic event by weakening chemical bonds of reactants and co-catalysts or modulating their redox potential. It has also been demonstrated by López and Gómez that complete 6-*endo*-selectivity over 5-*exo* ring closure in radical cyclization of C-centred radicals can be controlled by the radical property, substitution pattern at C-5 or ring strain of substrate^34^,^35^. Quite recently, the MacMillan group also first integrated elementary hydrogen atom transfer (HAT) process into H-bond catalysis, and achieved a highly selective photoredox α-alkylation/lactonization cascade of alcohols^41^. Based on these inspiring studies, we hypothesized that the aforementioned regioselective N-radical cascade reaction could possibly be achieved by merging visible-light photoredox with TEMPO-mediated HAT process, wherein the N–H bond might be directly converted into the corresponding N-centred hydrazonyl radical through an ODET and the aza-allylic C–H bond can probably be homolytically cleavaged by a suitable H-atom acceptor such as TEMPO^42^.

**Figure 2:**
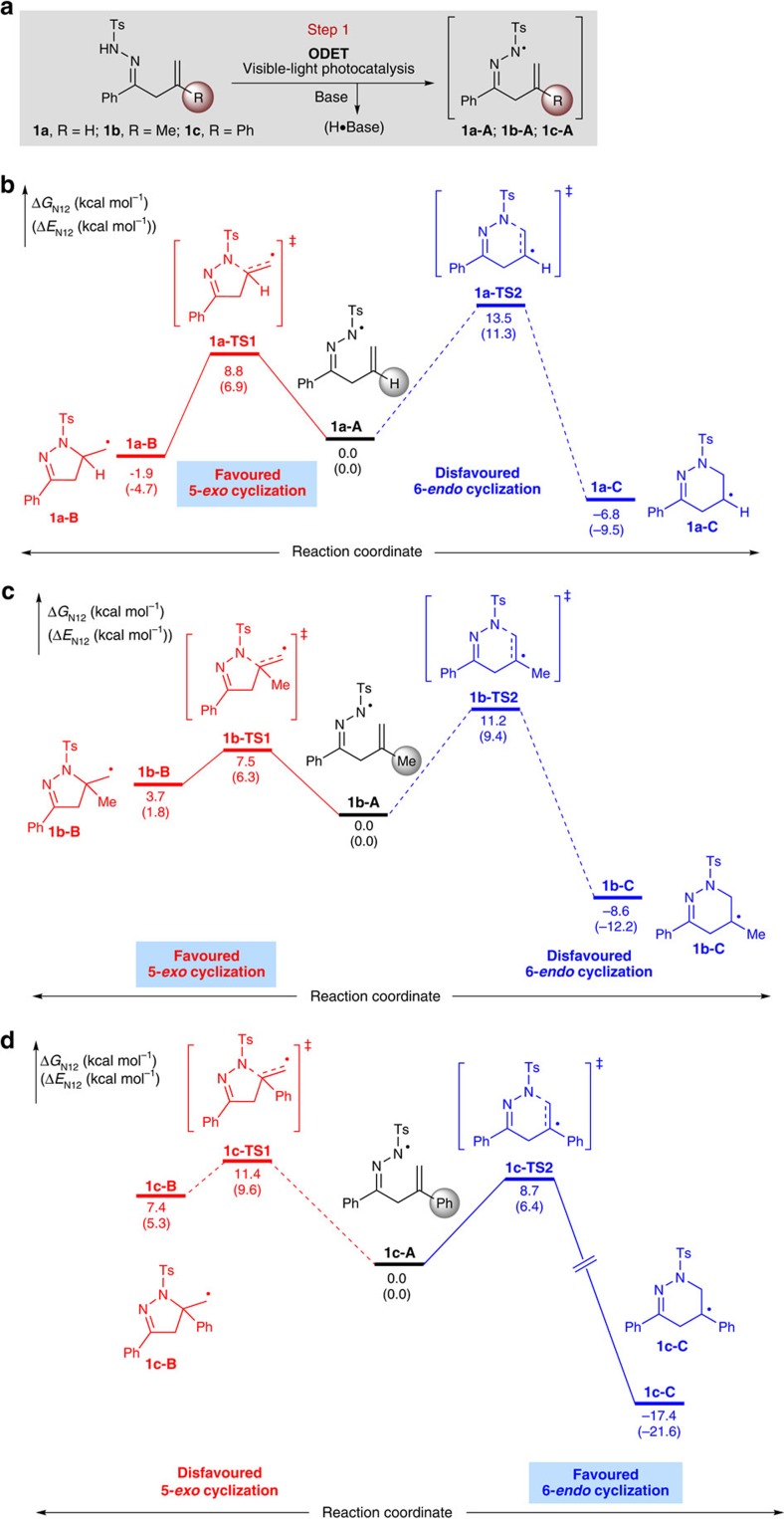
Reaction development. (**a**) Generation of N-radicals by visible-light photocatalysis. (**b**) Free energy profiles for 5-*exo* and 6-*endo* radical cyclizations of **1a-A**. (**c**) Free energy profiles for 5-*exo* and 6-*endo* radical cyclizations of **1b-A**. (**d**) Free energy profiles for 5-*exo* and 6-*endo* radical cyclizations of **1c-A**.

To test the feasibility of this strategy, we initially conducted density functional theory (DFT) calculations on the cyclization step of N-centred radical intermediates **1a-A**, **1b-A** and **1c-A** with sterically and electronically diverse substituents at the 2-position of the alkene (Fig. 2a; see Supplementary Notes 1–3 for details). The energies given in this work are N-12//6-311+G(d, p)//B3 LYP/6-31G(d) calculated Gibbs free energies in chloroform. See the Supplementary Information for more computational details.)^43^. As expected, both 5-*exo* and 6-*endo* N-radical-mediated radical cyclizations are possible pathways. For example, the study showed that the 5-*exo*-trig radical cyclization of **1a-A** with an activation free energy of only 8.8 kcal mol^−1^ via **1a-TS1** is much more favoured than its 6-*endo*-trig variant (activation free energy of 13.5 kcal mol^−1^; Fig. 1b). It was also found that **1b-A** would undergo 5*-exo* cyclization through **1b-TS1** more feasibly than its 6-*endo* cyclization via **1b-TS2**, as shown by their activation free energy (Fig. 1c, 7.5 versus 11.2 kcal mol^−1^). Interestingly, the 6-*endo* cyclization of **1c-A** with a phenyl group at the 2-position of the alkene moiety proved to be easier to accomplish through **1c-TS2**, with a relatively low activation free energy of 8.7 kcal mol^−1^, to give the C-centred radical intermediate **1c-C** (Fig. 1d). Encouraged by these computational results, we proceeded to perform experimental studies with these substrates to explore the feasibility of the desired 6-*endo* radical cyclization.

**Figure 3:**
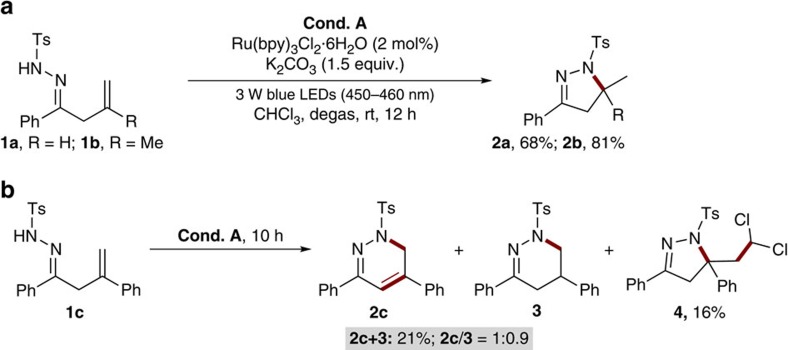
Initial results. (**a**) Reaction of substrate **1a** and **1b** under condition **A**. (**b**) Reaction of substrate **1c** under condition **A**. Unless otherwise noted, condition **A**: reaction were run with **1** (0.2 mmol), Ru(bpy)_3_Cl_2_·6H_2_O (2mol%), K_2_CO_3_ (0.3 mmol), 3 W blue light-emitting diodes (450–460 nm) irradiation and CHCl_3_ (4.0 mL) at rt for 10–12 h.

Under our previously developed visible-light photocatalytic conditions for hydroamination of β,γ-unsaturated hydrazones^28^, substrates **1a** and **1b** indeed underwent 5-*exo* radical cyclization reactions smoothly to give the corresponding products **2a** and **2b** in 68% and 81% yields, respectively (Fig. 3a). These results also provided a solid support for the above computational investigations into these substrates. Interestingly, the reaction of **1c** resulted in the formation of a complex mixture with a complete conversion (Fig. 3b). Careful analysis of the reaction mixture revealed that an inseparable mixture of products **2c** and **3** can be obtained in 21% yield with a ratio of 1:0.9. Meanwhile, product **4** was also isolated in 16% yield, which might be formed through another radical cascade reaction between **1c** and the reaction solvent CHCl_3_ via radical intermediate **1c-B**. The structures of **2a-2c**, **3** and **4** were fully characterized by their ^1^H and ^13^C NMR spectra and mass data, and compound **4** was further characterized by single-crystal X-ray analysis (see Supplementary Fig. 79 for details). Note that the biologically significant 1,6-dihydropyridazines of type **2c** cannot be easily prepared using traditional methods^33^. These observations suggested that further optimization of reaction parameters might result in the exclusive formation of the desired 1,6-dihydropyridazines.

### Optimization of reaction conditions

Encouraged by these initial results, we continued to optimize the reaction conditions with **1c** as a model substrate to further improve the selectivity and yield (Table 1). Inspired by the recently demonstrated wide applicability of nitroxides in organic synthesis and their unique properties^44^,^45^, we initially focused on nitroxides as potential additives. Surprisingly, it was found that the addition of TEMPO (1.0 equiv.) did not quench the reaction; instead, it resulted in a clean reaction and gave the desired 1,6-dihydropyridazine **2c** in 89% yield (entry 1). Based on our blueprint of the reaction, we postulated that TEMPO might serve as a H-atom acceptor to abstract aza-allylic H-atom by an HAT process^41^. Then, we simply screened inorganic bases such as NaOH, Na_2_CO_3_ and Na_2_HPO_4_, and established that the base also played an important role in the reaction, with K_2_CO_3_ identified as the best choice (entries 2–4). With K_2_CO_3_ as the base, we also briefly examined several other common solvents and CHCl_3_ proved to be the best reaction media with tetrahydrofuran, MeOH, dimethylformamide and CH_3_CN giving relatively low yields (entries 5–8). Then, we evaluated the effect of photocatalysts on the reaction under otherwise identical conditions. Interestingly, the use of Ir(ppy)_2_(dtbbpy)PF_6_ as a photocatalyst provided comparable results, whereas organic photocatalyst Eosin Y was ineffective for the reaction (entries 9–10). It has been well documented that TEMPO can serve not only as a radical scavenger but also as an oxidant in transition-metal catalysis^44^,^45^. Thus, we continued to test several other oxidants, such as K_2_S_2_O_8_ and 2,3-dichloro-5,6-dicyano-1,4-benzoquinone (see Supplementary Table 1 for details). However, all the reactions with these oxidants resulted in a complex mixture without formation of any desired product, suggesting that TEMPO might act as a radical trap to abstract the α-hydrogen atom from intermediate **1c-C** to facilitate the target N-radical cascade reaction pathway (entries 11–12). In the control experiments with CHCl_3_ or CH_3_CN as the solvent, only very little or no desired products were detected in the absence of photocatalyst, base, TEMPO or light; large amounts of starting materials remained intact, highlighting the critical role of all the parameters in the reaction (entries 13–16; see Supplementary Table 2 for details).

### Substrate scope

**Figure 4:**
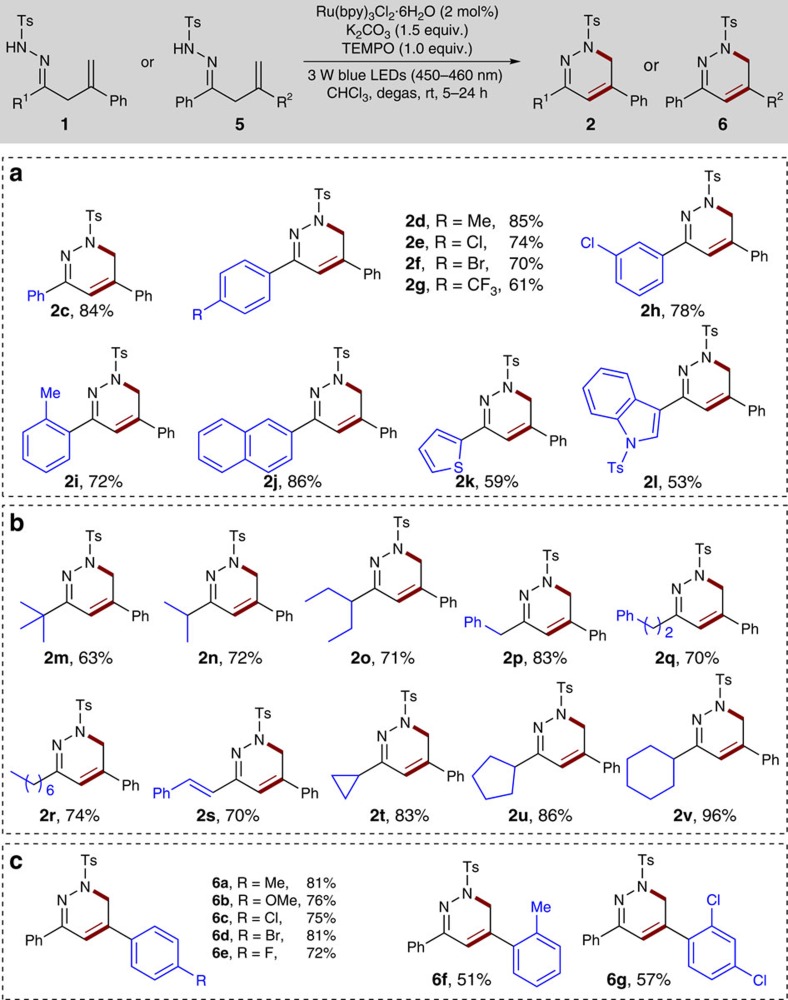
Reaction scope of unsaturated hydrazones. (**a**) Investigation of the effects of arene substitution of hydrazones. (**b**) Substrate scope of aliphatic unsaturated hydrazones. (**c**) Substrate scope of alkene moieties. Unless otherwise noted, reactions were run with **1** or **5** (0.3mmol), Ru(bpy)_3_Cl_2_·6H_2_O (0.006mmol, 2.0mol%), K_2_CO_3_ (0.45mmol), TEMPO (0.3mmol) and CHCl_3_ (6.0ml) at rt for 5–24 h under irradiation with 3 W blue light-emitting diodes (450–460 nm).

Under the optimized conditions, we then evaluated the substrate scope of this transformation with a variety of β,γ-unsaturated hydrazones (Fig. 4). First, we examined the effects of arene substitution using a wide range of β,γ-unsaturated hydrazones **1c-1i**. It was found that the reaction with various β,γ-unsaturated hydrazones bearing electron-neutral, electron-poor (for example, Cl, Br, CF_3_) or electron-rich (for example, Me) substituents at the 2-, 3- or 4-position of the aromatic ring proceeded well to deliver the corresponding products **2c**-**2i** with yields ranging from 61 to 85%. Notably, those aryl bromides are amenable to further synthetic elaborations through transition-metal-catalyzed C–C coupling reactions. Product **2f** was also characterized by single-crystal X-ray analysis (see Supplementary Fig. 79 for details). Moreover, 2-naphthyl substituted hydrazone **1j** reacted well to afford product **2j** in 86% yield. Considering the known medicinal chemistry, it is noteworthy that various heterocycles can be incorporated into the hydrazone substrates with no apparent deleterious effect on the reaction efficiency. For example, 2-thiophenyl and 3-indolyl substituted hydrazones were tolerated well to give the desired products **2k** and **2l** in 59% and 53% yields, respectively. More importantly, the substrate scope of the current protocol can be successfully extended to aliphatic β,γ-unsaturated hydrazones. Thus, the reaction with a series of linear and branched aliphatic β,γ-unsaturated hydrazones **1m-1r** can undergo the radical cascade reaction smoothly under standard conditions, although with prolonged reaction times, to afford the products **2m**-**2r** in 63–83% yield. The β,γ-unsaturated hydrazone **1s** bearing a styryl group also appeared to be viable for the reaction, producing a 70% yield of **2s**. Remarkably, cyclic substituents, such as cyclopropyl, cyclopentyl and cyclohexyl groups, could also be easily incorporated into the 1,6-dihydropyridazine scaffold with high yields (**2t**-**2v**, 83–96%).

Encouraged by these results, we proceeded to examine the scope of alkene moieties by incorporation of various substituents into the phenyl ring. As highlighted in Fig. 4c, the substitution patterns and electronic properties of the aromatic ring showed no apparent effect on the reaction efficiency either. For example, all the electron-releasing (for example, 4-Me, 2-Me and 4-MeO) and electron-withdrawing (for example, 4-F, 4-Cl, 4-Br, 2,4-2Cl) groups were well tolerated under the standard conditions, furnishing the expected products **6a-6g** in 51–81% yield. Interestingly, during our subsequent biological studies with 1,6-dihydropyridazines **2-** and **6**-derived diazinium salts, it was found that such aromatic substituents at the 2-position of the alkene are critical to their antifungal *in vitro* activities. It should be noted that we did not detect any 5-*exo* cyclization products in all cases^29^,^30^.

### Mechanistic investigations

**Figure 5:**
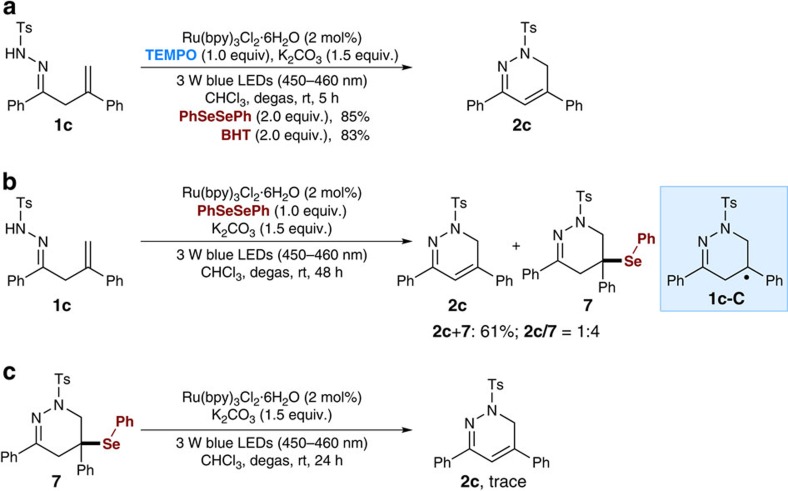
Mechanistic investigations. (**a**) Trapping the C-centred intermediate by addition of PhSeSePh or BHT under the standard conditions. (**b**) Trapping the C-centred intermediate by addition of PhSeSePh under the standard conditions in the absence of TEMPO. (**c**) Control experiment with selenide-adduct **7** under the standard conditions.

To gain additional insights into the reaction mechanism, several control experiments were conducted with model substrate **1c** (Fig. 5; see Supplementary Discussion for details). To further confirm the formation of C-centred intermediate of type **1c**-**C** during the reaction, common radical trapping agents (PhSeSePh or 2,6-di-tert-butyl-4-methylphenol, BHT) were added to the reaction system to capture the radical intermediate (Fig. 5a). However, no trapping products were observed; instead, only the 1,6-dihydropyridazine **2c** was produced and isolated in 85% and 83% yields, respectively. In contrast, without addition of TEMPO, the reaction with PhSeSePh as a radical trapping agent furnished a mixture of desired **2c** and selenide-adduct **7** (61% yield, 1:4 ratio; see Supplementary Fig. 78 for details), and compound **7** should be formed from radical intermediate **1c**-**C** and PhSeSePh (Fig. 5b). Then, we obtained the pure selenide-adduct **7** by semi-preparative high-performance liquid chromatography purification and re-subjected it to the standard reaction conditions without TEMPO (Fig. 5c). However, we did not detect any desired product **2c** even after 24 h and compound **7** remained intact, suggesting that selenide-adduct **7** should not be the possible intermediate for the formation of 1,6-dihydropyridazine **2c**.

**Figure 6:**
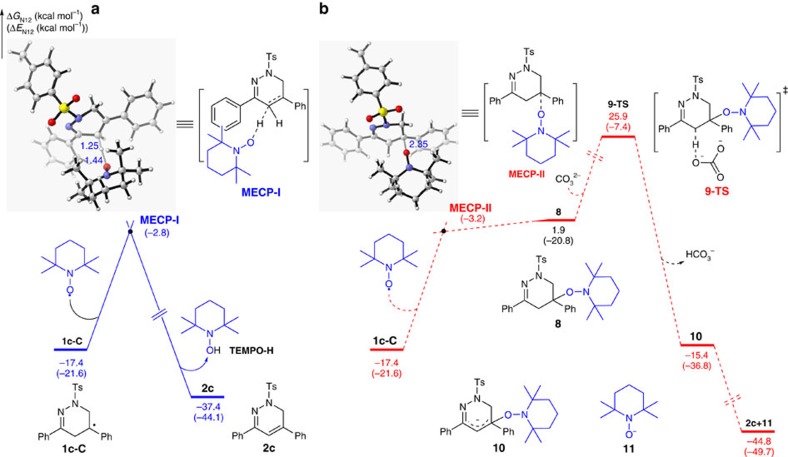
Calculation studies. (**a**) Free energy profile for the transformation of C-centred radical **1c-C** into product **2c** through a TEMPO-mediated HAT process. (**b**) Free energy profile for the transformation of C-centred radical **1c-C** into product **2c** through carbon radical trapping/elimination process.

To further determine the role of TEMPO, we also calculated the free energy of the subsequent transformation of C-centred radical intermediate **1c**-**C** into the final product **2c** via the minimum energy crossing point (MECP; Fig. 6)^43^. As shown in Fig. 6a, the computational results showed that the TEMPO might facilitate the conversion of the intermediate **1c**-**C** into the final product **2c** through a TEMPO-mediated HAT process, because the calculated energy barrier (Δ*E*) for the aza-allylic hydrogen atom abstraction via **MECP-I** is only 18.8 kcal mol^−1^. Moreover, the generation of product **2c** is exergonic by 20.0 kcal mol^−1^ compared with the intermediate **1c**-**C**. Recently, a similar trapping of carbon radical and elimination of TEMPO-H process in the presence of base has been identified by Chiba's group as the possible pathway in TEMPO-mediated C–H bond oxygenation of oximes and hydrazones^46^. Inspired by this work, another possible pathway involving carbon radical trapping/elimination sequence of **1c**-**C** in the presence of base was also considered in calculation. As shown in Fig. 6b, the combination of radical **1c**-**C** with TEMPO occurs through **MECP-II**, and the energy barrier (Δ*E*) of which is 18.4 kcal mol^−1^. Although this energy barrier is close to that of **MECP-I** formation (Fig. 6a), the formation of TEMPO-adduct **8** is endergonic by 19.3 kcal mol^−1^ compared with **1c**-**C**. Moreover, the activation free energy of subsequent deprotonation, which occurs via transition state **9-TS**, reaches as high as 43.3 kcal mol^−1^. According to these results, the sequential combination of carbon radical **1c**-**C** with TEMPO and elimination process appears to be thermodynamically unfavourable. Moreover, we also intended to isolate the possible intermediate **8** upon ∼50% conversion of model substrate **1c**. Unfortunately, all the attempts met failure, although a trace amount of intermediate **8** was detected by the high-resolution mass spectrometry analysis of the the reaction mixture (see Supplementary Information). Another possible pathway for base-free elimination of TEMPO-H from **8** by direct radical elimination with C–O bond homolysis is not considered as the stoichiometric base is necessary in our reaction system^47^,^48^,^49^. Taken together, although the calculation studies support the TEMPO-mediated HAT process as the likely mechanism for the transformation of C-centred radical intermediate **1c**-**C** into the final product **2c**, at present we cannot rule out the carbon radical recombination/elimination pathway (see Supplementary Figs 80 and 81 and Supplementary Notes 1-3 for details). More detailed mechanistic studies are curently underway in our laboratory.

**Figure 7:**
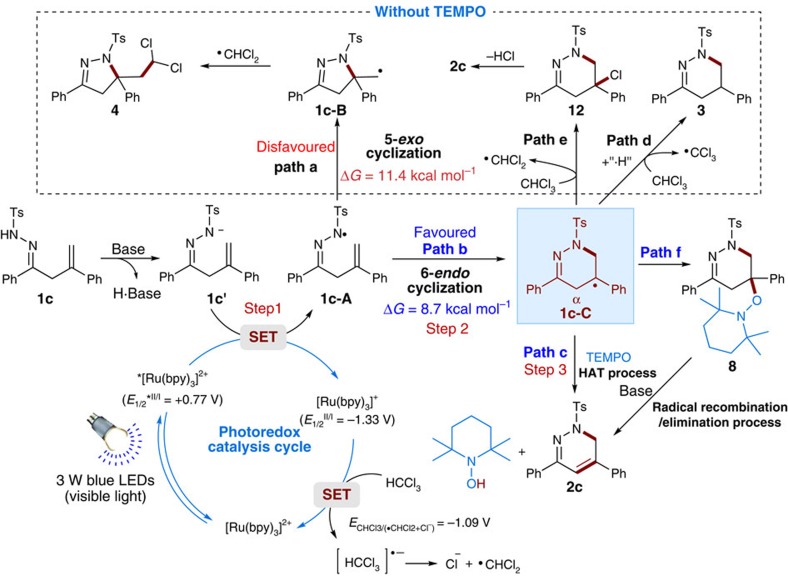
Proposed catalytic cycle. The plausible mechanism involves oxidative deprotonation electron transfer (ODET) activation of N–H bond into N-centred radical by visible light photoredox catalysis and TEMPO-mediated N-radical cyclization.

According to our blueprint for ODET activation of N–H bond, the addition of K_2_CO_3_ proved be critical for the reaction as a base and this phenomenon was indeed observed during the optimization study (Table 1, entry 14). To further evaluate the role of base in these reactions, we continue to study the mechanism of N-centred hydrazonyl radical formation by luminescence quenching experiments, NMR and electrochemical analysis with **1c** as a model substrate (see Supplementary Figs 82–86 for details). Stern–Volmer analysis demonstrated that hydrazone **1c** alone is unable to quench the excited state of *[Ru(bpy)_3_]^2+^ in dimethylformamide at 25 °C, implying that the excited state ruthenium complex does not oxidize the hydrazone **1c** directly. However, upon addition of K_2_CO_3_ as a base, a significant decrease of luminescence emission intensity was observed. In addition, the ^1^H NMR analysis of a solution containing both **1c** and K_2_CO_3_ exhibited that the addition of K_2_CO_3_ resulted in complete disappearance of the signal of N–H, suggesting that K_2_CO_3_ serve to abstract the proton of N–H bond to generate nitrogen anion intermediate **1c'** (Fig. 7 and Supplementary Information). Moreover, cyclic voltammetry data confirmed that the excited photocatalyst *Ru(bpy)_3_^2+^ (*E*_1/2_^*II/I^=+0.77 V versus SCE in CH_3_CN) is likely to be sufficiently oxidizing to oxidize the nitrogen anion **1c'** (*E*_p_^red^=0.56 V versus SCE) to generate the corresponding N-centred radical intermediate **1c-A** (Fig. 7). Taken together, although we could not completely exclude the concerted proton-coupled electron transfer mechanism at the current stage^24^,^38^,^39^, the above results are more consisted with an ODET activation mechanism involving sequential deprotonation of hydrazone substrates by the K_2_CO_3_ and visible-light photocatalytic single-electron transfer (SET) oxidation.

Ultimately, a plausible mechanism is outlined in Fig. 7 using **1c** as an example. Initially, the β,γ-unsaturated hydrazone **1c** is transformed into anionic intermediate **1c'** upon deprotonation, which is then oxidized to the N-centred radical **1c**-**A** by the excited photocatalyst *[Ru(bpy)_3_]^2+^ through a SET process. Then, the key intermediate **1c**-**A** undergoes a 6-*endo* radical cyclization to afford the C-centred benzylic radical intermediate **1c**-**C**, which can be conveniently transformed into the final product **2c** by an HAT process in the presence of TEMPO (path c). However, as for the transformation of C-centred radical intermediate **1c-C** into the final product **2c**, at the current stage, we cannot rule out the carbon radical recombination/elimination pathway that involves TEMPO-adduct **8** as the key intermediate (path f, see Supplementary Information). In the absence of TEMPO, the intermediate **1c**-**C** can abstract a hydrogen atom directly from CHCl_3_ to give 1,4,5,6-tetrahydropyridazine **3** (path d). Meanwhile, the intermediate **1c**-**C** can also abstract a chlorine radical from chloroform to give rise to dichloromethyl radical and labile tertiary chloride adduct **12** intermediate^50^, which can undergo facile elimination to give the product **2c**. Moreover, without addition of TEMPO, the intermediate N-centred radical **1c**-**A** could also undergo a 5-*exo* radical cyclization (path a) to furnish **1c**-**B**, partly because of the relatively small activation free energy difference between **1c**-**B** and **1c**-**C** (Fig. 2d). In the photocatalytic cycle, chloroform can regenerate the photocatalyst [Ru(bpy)_3_]^2+^ by an SET oxidation process with the concomitant release of the chloroform radical anion, which rapidly dechlorinated to give chloride ion and the dichloromethyl radical^51^,^52^,^53^,^54^. The formation of a dichloromethyl radical in the reaction was also confirmed by the isolation of side product **4**, resulting from the radical cross coupling between the dichloromethyl radical and **1c**-**B** intermediate.

### Synthetic application

**Figure 8:**
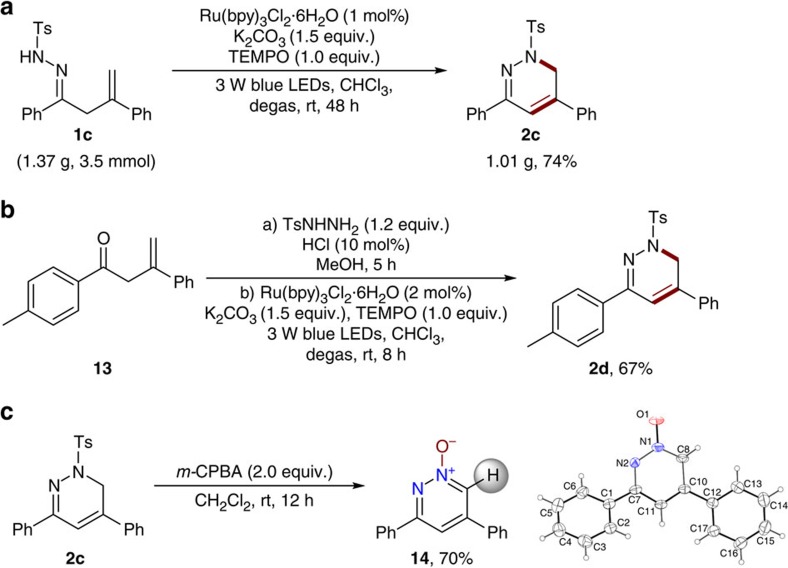
Synthetic application. (**a**) Gram-scale reaction. (**b**) One-pot process for synthesis of 1,6-dihydropyridazine **2d**. (**c**) Synthesis of pyridazine *N*-oxide **14**.

To further demonstrate the synthetic potential of this method, a gram-scale reaction of β,γ-unsaturated hydrazone **1c** was conducted in the presence of 1 mol% of photocatalyst under standard reaction conditions, and the desired product **2c** was still successfully obtained in 74% yield after 48 h (Fig. 8a). A key benefit of this photocatalytic radical cyclization strategy is that the β,γ-unsaturated hydrazone starting materials are easily accessed from the corresponding β,γ-unsaturated ketones and tosyl hydrazine. Thus, we examined the photocatalytic radical cyclization with β,γ-unsaturated ketone **13** and tosyl hydrazine in a two-step one-pot process (Fig. 8b). Pleasingly, the desired 1,6-dihydropyridazine **2d** was obtained in 67% overall yield. Recently, heteroaromatic *N*-oxides have been widely employed in transition-metal-catalyzed aromatic C–H activation/functionalization reactions to access various valuable heterocyclic molecules^55^. We found that the present method could provide a new approach to the synthesis of pyridazine *N*-oxides. For example, treatment of **2c** with *m*-CPBA as the oxidant resulted in the facile formation of pyridazine *N*-oxide **14** in a 70% yield that was also clearly determined by X-ray analysis (Fig. 8c; see Supplementary Fig. 79 for details).

**Figure 9:**
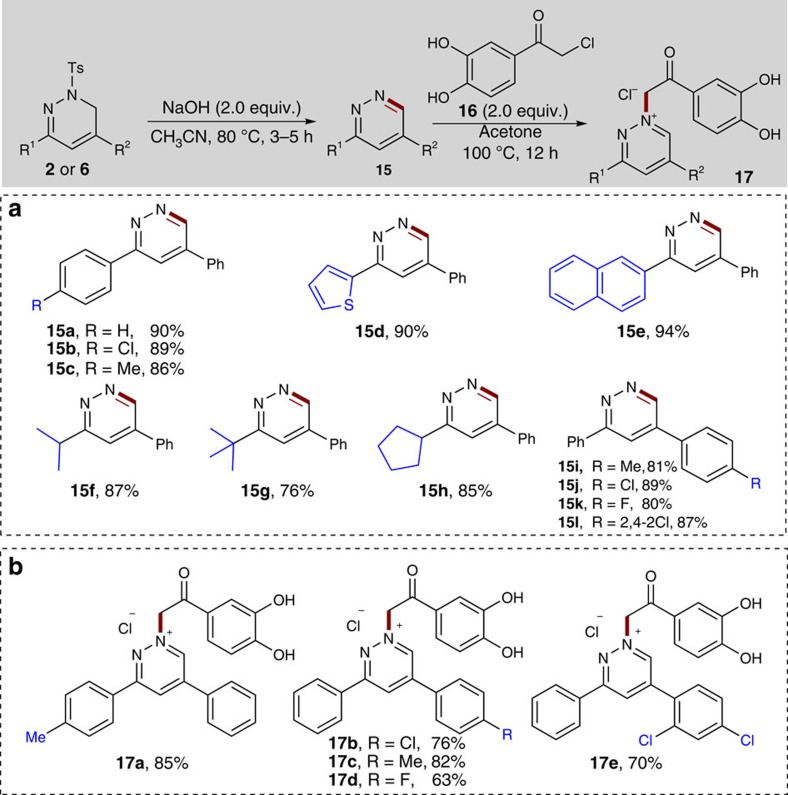
Application to the synthesis of pyridazines and diazinium salts. (**a**) Reactions were run with **2** or **6** (0.2 mmol), NaOH (0.6 mmol) and CH_3_CN (4.0 ml) at 80 °C for 3–5 h. (**b**) Reactions were run with **15** (0.3 mmol), **16** (0.6 mmol) and acetone (3.0 ml) at 100 °C for 12 h.

Moreover, it was then established that the 1,6-dihydropyridazine products can also be easily transformed into the corresponding biologically important pyridazines under mild conditions (2.0 equiv. NaOH in CH_3_CN at 80 °C). As highlighted in Fig. 9a, the electronic and steric properties of the substituents on both of the aromatic rings showed no significant effect on the reaction efficiency. A series of substrates with electron-rich or electron-poor substituents worked well to give the desired products in good yields (**15a**-**15d**, 86–90% yield; **15i**–**15l**, 81–89% yield). In addition, 2-thiophenyl and 2-naphthyl-substituted 1,6-dihydropyridazines reacted well to give the corresponding pyridazine products **15d** and **15e** in 90% and 94% yield, respectively. Remarkably, the 1,6-dihydropyridazines bearing alkyl groups such as isopropyl, *tert*-butyl and cyclohexyl substituents, were well tolerated to deliver the desired products **15f-15h** in high yields (76–87%).

It has recently been documented that the pyridazine derivatives, such as diazinium salts bearing a dihydroxyacetophenone core, showed promising biological activities against a variety of microorganisms (germs and fungi)^56^. Thus, we further attempted to transform a range of representative pyridazines **15** into the corresponding diazinium salts **17** and preliminarily explored their potential structure–activity relationships (Fig. 9b). By refluxing a mixture of pyridazines **15** and 2-chloro-3′,4′-dihydroxyacetophenone **16** in acetone for 12 h, a series of diazinium salts **17a**-**17e** were easily obtained in 63–85% yield after a simple filtration.

Over the past decades, the incidence of invasive fungal infections and the associated morbidity and mortality rates have risen remarkably due to the over-use of broad-spectrum antibiotics, serious medical interventions and immune deficiency disorders, such as AIDS^57^,^58^. Despite recent additions to the antifungal drug family, the limitations of the current antifungal drugs involve narrow activity spectra, detrimental drug–drug interactions and antifungal resistance, necessitating the development of new antifungal agents or leads. With diazinium salts **17a**-**17e** in hand, we evaluated the *in vitro* antifungal activities of these compounds against eight human pathogenic fungi, compared with commercially available fluconazole. In contrast to the antibacterial activities reported for related diazinium salts^56^, our results demonstrated that some of these compounds showed promising activities against four common clinical pathogenic fungi (*Candida albicans*, *C. parapsilosis*, *C. neoformans* and *C. glabrata*; see Supplementary Tables 3 and 4 for details). These results also confirmed that the substitution patterns and electronic properties of the substituents at both of the phenyl rings are critical to their *in vitro* antifungal activities. Gratifyingly, the MIC_80_ values of most of the compounds (**17b**-**17e**) against *C. parapsilosis*, *C. neoformans* and *C. glabrata* (0.5–4 μg ml^−1^) were comparable to those of fluconazole, which should be valuable for our future biological studies.

## Discussion

We have developed a novel ODET/HAT strategy, which we used to directly convert the N–H bond of β,γ-unsaturated hydrazones to the N-centred radical, and developed an efficient catalytic N-radical cascade reaction. This mild protocol represents the first, to our knowledge, broadly applicable synthesis of 1,6-dihydropyradazines with good regioselectivity and yield, achieved by the merge of visible-light photocatalysis and TEMPO mediation. The 1,6-dihydropyridazines could also be conveniently transformed into biologically important diazinium salts bearing dihydroxyacetophenone core, which showed promising antifungal *in vitro* activities against various fungal pathogens. Control experiments and DFT calculations have been performed to help explain the mechanism. Owing to the wide occurrence of various N–H bonds, we believe that this strategy may find wide use for generation of other various *N*-centred radicals and new reaction developments with these reactive species^59^.

## Methods

### Materials

Unless otherwise noted, materials were purchased from commercial suppliers and used without further purification. All the solvents were treated according to general methods. Flash column chromatography was performed using 200–300 mesh silica gel. The manipulations for photocatalytic N-radical cascade reactions were carried out with standard Schlenk techniques under Ar by visible-light irradiation. See Supplementary Methods for experimental details.

### General methods

^1^H NMR spectra were recorded on 400 or 600 MHz spectrophotometers. Chemical shifts are reported in delta (*δ*) units in parts per million (p.p.m.) relative to the singlet (0 p.p.m.) for tetramethylsilane. Data are reported as follows: chemical shift, multiplicity (s=singlet, d=doublet, t=triplet, dd=doublet of doublets, m=multiplet), coupling constants (Hz) and integration. ^13^C NMR spectra were recorded on 100 or 150 MHz with complete proton-decoupling spectrophotometers (CDCl_3_: 77.0 p.p.m. or DMSO-d^6^: 39.5 p.p.m.). ^19^F NMR spectra were recorded on 376 MHz with complete proton-decoupling spectrophotometers. Mass spectra were measured on MS spectrometer (EI) or liquid chromatography-mass spectrometry (LC/MS), or electrospray ionization mass spectrometry (ESI-MS). High-resolution mass spectrometry was recorded on Bruker ultrafleXtreme matrix-assisted laser desorption/ionization–time-of-flight (TOF)/TOF mass spectrometer. ^1^H NMR, ^13^C NMR and ^19^F NMR spectra are supplied for all compounds: see Supplementary Figs 1–77.

### General procedure for catalytic nitrogen radical cascade reaction of hydrazones

In a flame-dried Schlenk tube under Ar, **1c** (117.0 mg, 0.3 mmol), Ru(bpy)_3_Cl_2_.6H_2_O (0.006 mmol), TEMPO (46.9 mg, 0.3 mmol) and K_2_CO_3_ (61.2 mg, 0.45 mmol) were dissolved in CHCl_3_ (6.0 ml). Then, the resulting mixture was degassed via ‘freeze-pump-thaw' procedure (three times). After that, the solution was stirred at a distance of ∼5 cm from a 3-W blue light-emitting diodes (450–460 nm) at room temperature ∼5 h until the reaction was completed as monitored by thin-layer chromatography analysis. The crude product was purified by flash chromatography on silica gel (petroleum ether/ethyl acetate 20:1∼10:1) directly to give the desired product **2c** in 84% yield as a white solid. Full experimental details and characterization of new compounds can be found in the Supplementary Methods.

## Additional information

**Accession codes:** The X-ray crystallographic coordinates for structures reported in this Article have been deposited at the Cambridge Crystallographic Data Centre (CCDC), under deposition numbers CCDC 1407651 (2 f), 1407652 (4), 1407653 (14). These data can be obtained free of charge from the Cambridge Crystallographic Data Centre via http://www.ccdc.cam.ac.uk/data_request/cif.

**How to cite this article:** Hu, X.-Q. *et al*. Catalytic N-radical cascade reaction of hydrazones by oxidative deprotonation electron transfer and TEMPO mediation. *Nat. Commun.* 7:11188 doi: 10.1038/ncomms11188 (2016).

## Supplementary Material

Supplementary Information Supplementary Figures 1-86, Supplementary Tables 1-4, Supplementary Notes 1-3 and Supplementary References

## Figures and Tables

**Table 1:** Optimization of conditions for catalytic N-radical cascade reaction of unsaturated hydrazone **1c**.

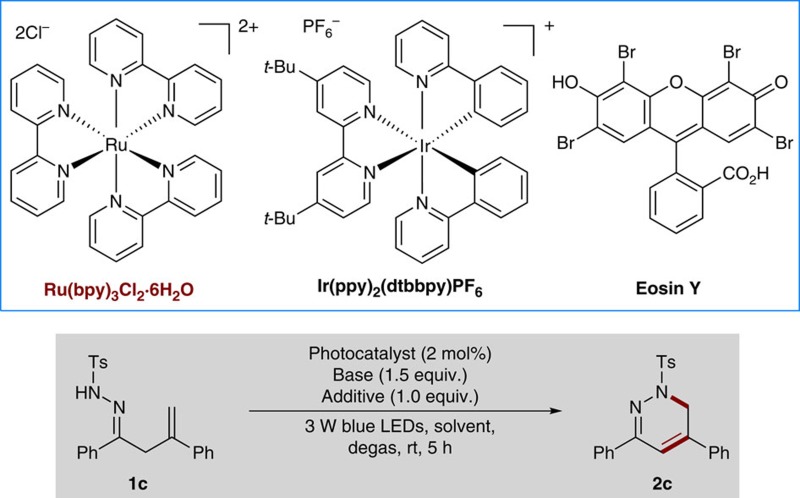					
**Entry**	**Photocatalyst**	**Base**	**Solvent**	**Additive**	Yield (%)*
1	Ru(bpy)_3_Cl_2_·6H_2_O	K_2_CO_3_	CHCl_3_	TEMPO	89
2	Ru(bpy)_3_Cl_2_·6H_2_O	NaOH	CHCl_3_	TEMPO	81
3	Ru(bpy)_3_Cl_2_·6H_2_O	Na_2_CO_3_	CHCl_3_	TEMPO	72
4	Ru(bpy)_3_Cl_2_·6H_2_O	Na_2_HPO_4_	CHCl_3_	TEMPO	8
5	Ru(bpy)_3_Cl_2_·6H_2_O	K_2_CO_3_	THF	TEMPO	51
6	Ru(bpy)_3_Cl_2_·6H_2_O	K_2_CO_3_	MeOH	TEMPO	23
7	Ru(bpy)_3_Cl_2_·6H_2_O	K_2_CO_3_	DMF	TEMPO	26
8	Ru(bpy)_3_Cl_2_·6H_2_O	K_2_CO_3_	CH_3_CN	TEMPO	48
9	Ir(ppy)_2_(dtbbpy)PF_6_	K_2_CO_3_	CHCl_3_	TEMPO	83
10	Eosin Y	K_2_CO_3_	CHCl_3_	TEMPO	Trace
11	Ru(bpy)_3_Cl_2_·6H_2_O	K_2_CO_3_	CHCl_3_	K_2_S_2_O_8_	Trace
12	Ru(bpy)_3_Cl_2_·6H_2_O	K_2_CO_3_	CHCl_3_	DDQ	Trace
13	—	K_2_CO_3_	CHCl_3_	TEMPO	0
14	Ru(bpy)_3_Cl_2_·6H_2_O	—	CHCl_3_	TEMPO	0
15	Ru(bpy)_3_Cl_2_·6H_2_O	K_2_CO_3_	CHCl_3_	—	11
16†	Ru(bpy)_3_Cl_2_·6H_2_O	K_2_CO_3_	CHCl_3_	TEMPO	0

DDQ, 2,3-dichloro-5,6-dicyano-1,4-benzoquinone; DMF, dimethylformamide; Eosin Y, tetrabromofluorescein; TEMPO, 2,2,6,6-tetramethylpiperidine-1-oxyl; THF, tetrahydrofuran.

Reaction conditions: **1c** (0.2 mmol), photocatalyst (0.004 mmol, 2.0 mol %), TEMPO (0.2 mmol), K_2_CO_3_ (0.3 mmol) and solvent (4.0 ml) at room temperature for 5 h under irradiation from a 3-W blue light-emitting diodes (450–460 nm).

^*^Isolated yields based on **1c**.

^†^Without visible-light irradiation.
