# Supplementary material for: Catalytic N-radical cascade reaction of hydrazones by oxidative deprotonation electron transfer and TEMPO mediation
Source: Nat Commun. 2016 Apr 6;7:11188. doi: 10.1038/ncomms11188 (PMC4823831; doi:10.1038/ncomms11188)
Supplement: Supplementary Information — Supplementary Figures 1-86, Supplementary Tables 1-4, Supplementary Notes 1-3 and Supplementary References [file ncomms11188-s1.pdf]

Supplementary Figures

Supplementary Figure 1. ^{1}H **NMR (400 MHz,** $CDCl_3$**) and** ^{13}C **NMR (100 MHz,** $CDCl_3$**) spectrum of hydrazone 1b**

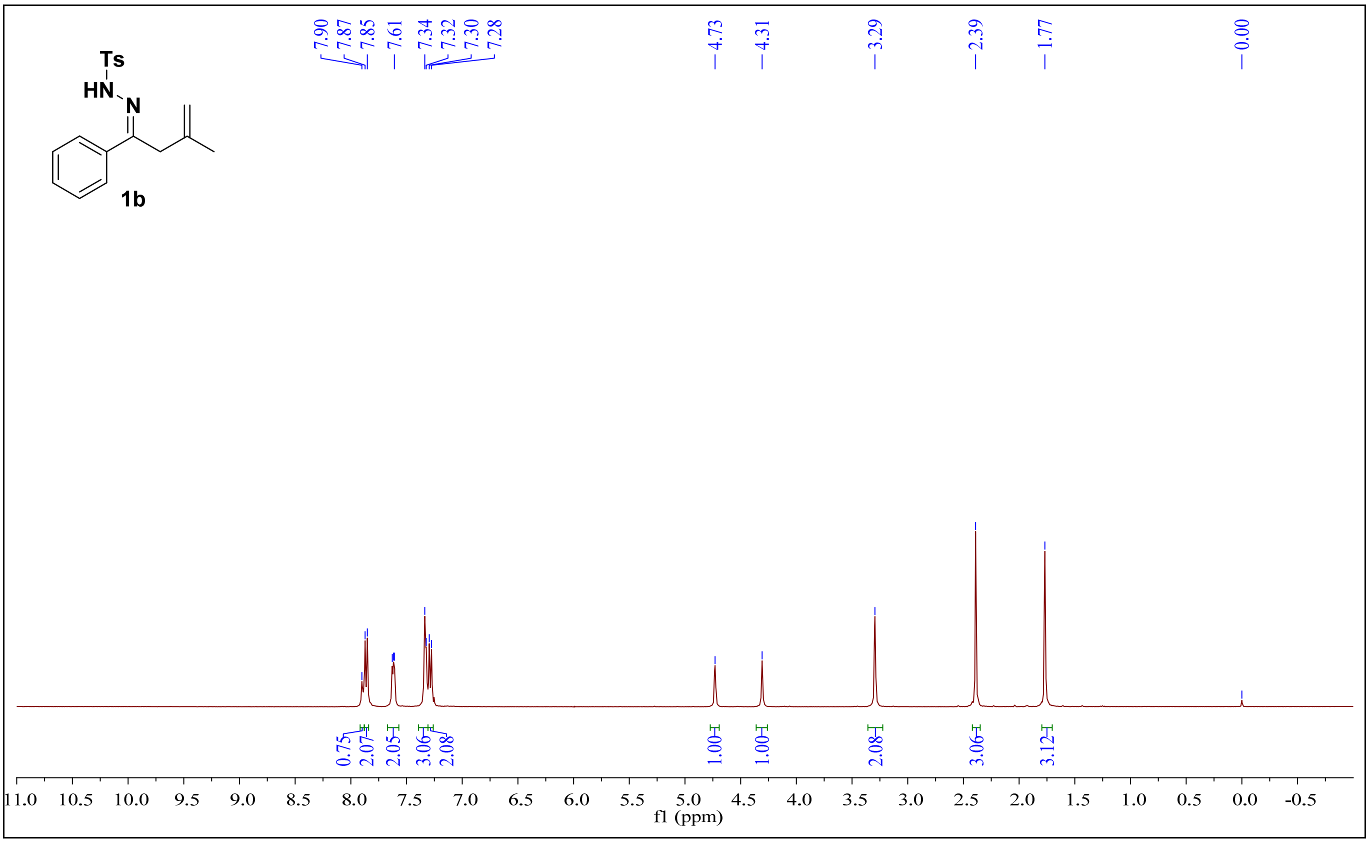

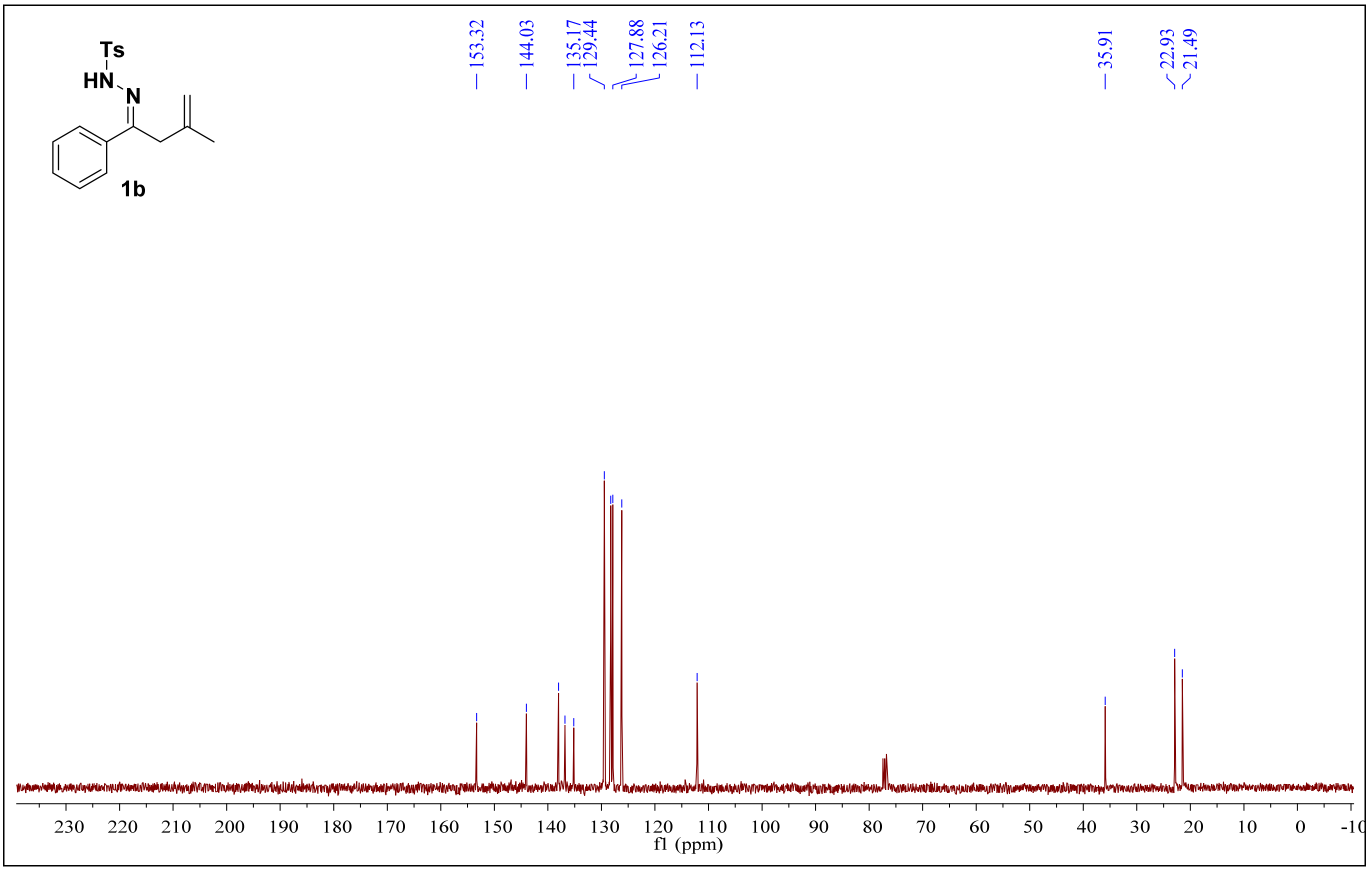

Supplementary Figure 2. ^{1}H **NMR (400 MHz, DMSO-d**^{6}**) and** ^{13}C **NMR (100 MHz, DMSO-d**^{6}**) spectra of hydrazone 1c**

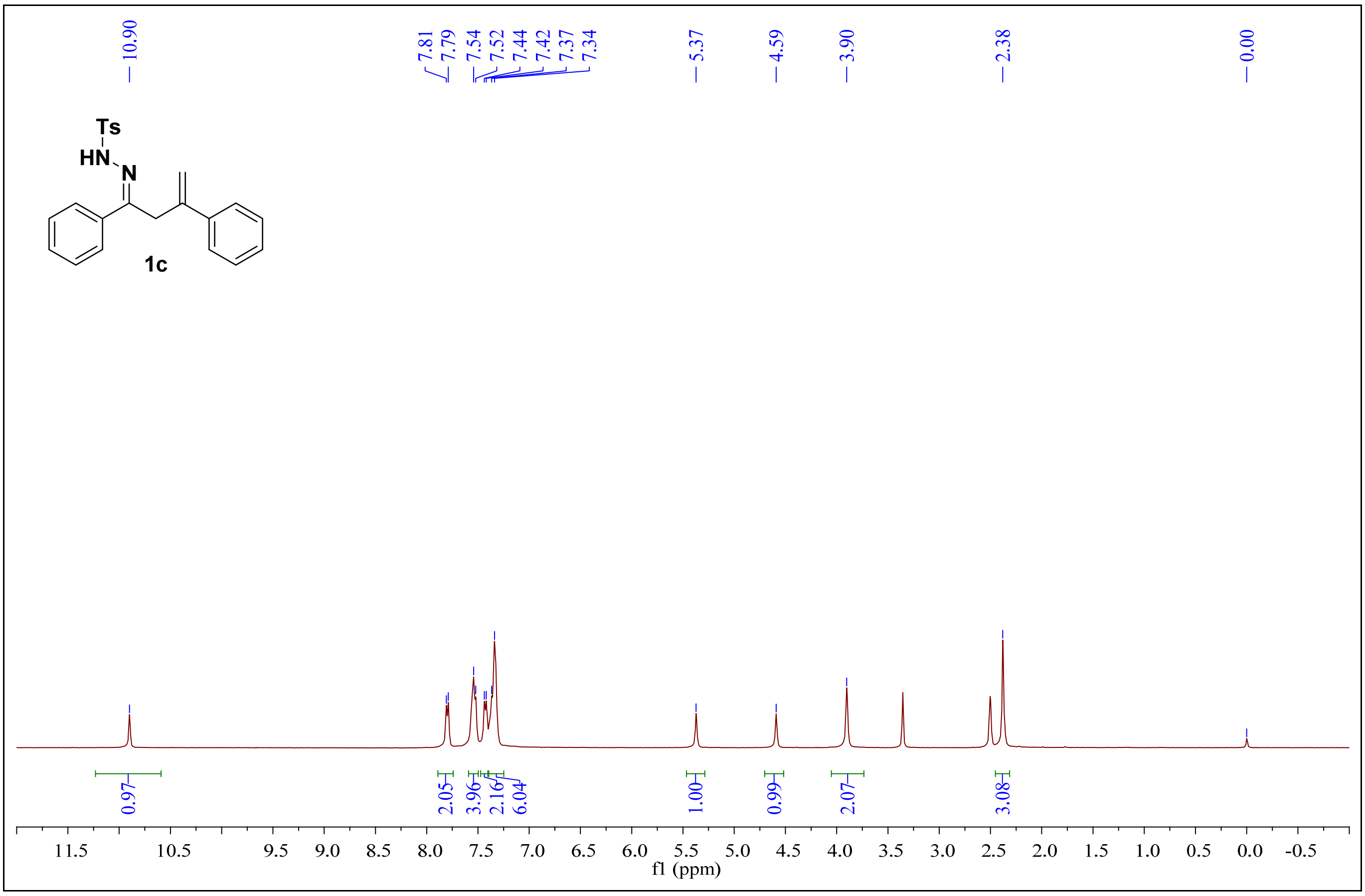

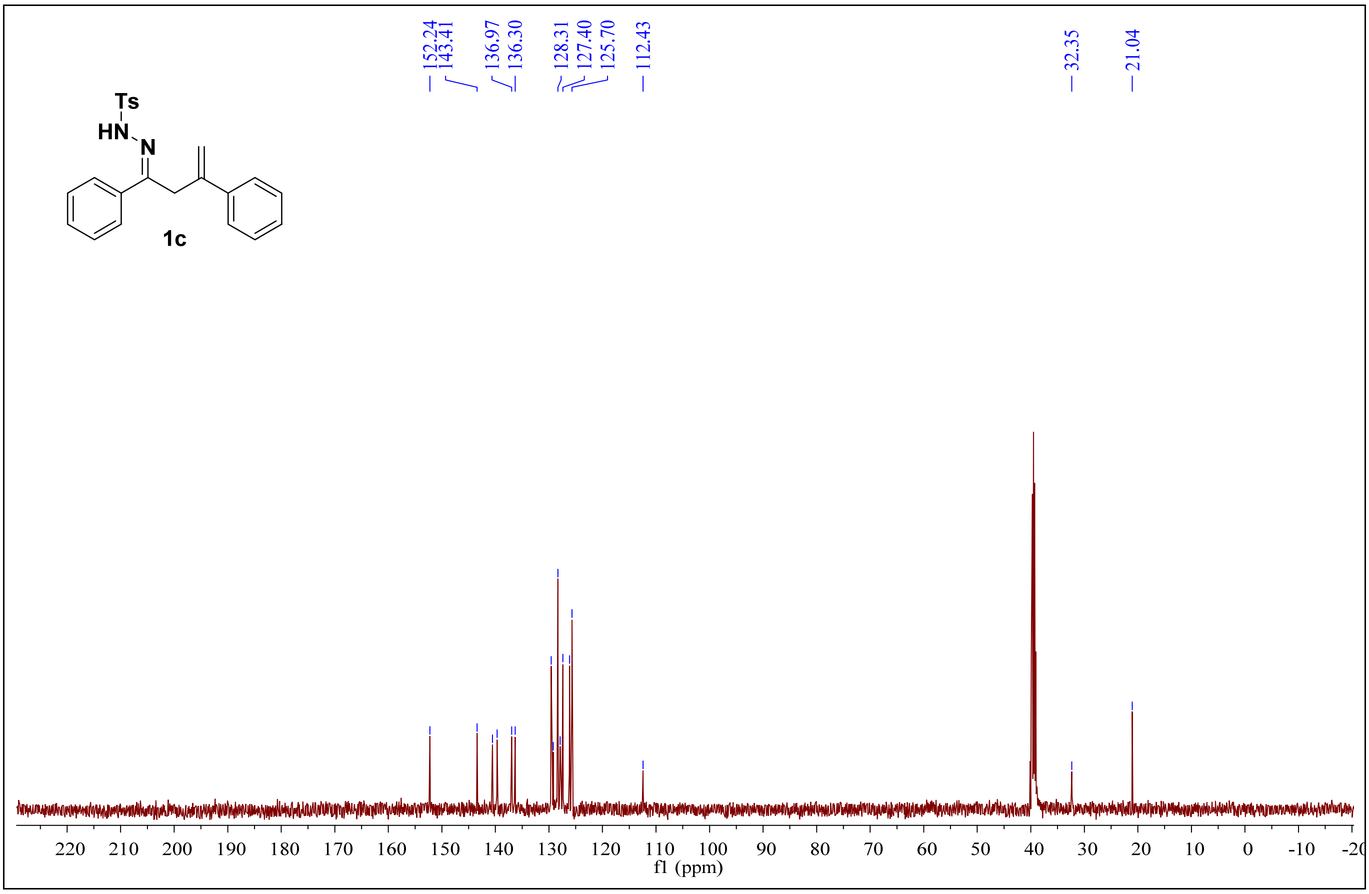

Supplementary Figure 3. ^{1}H NMR (600 MHz, $CDCl_3$) and ^{13}C NMR (100 MHz, $CDCl_3$) spectra of hydrazone 1d

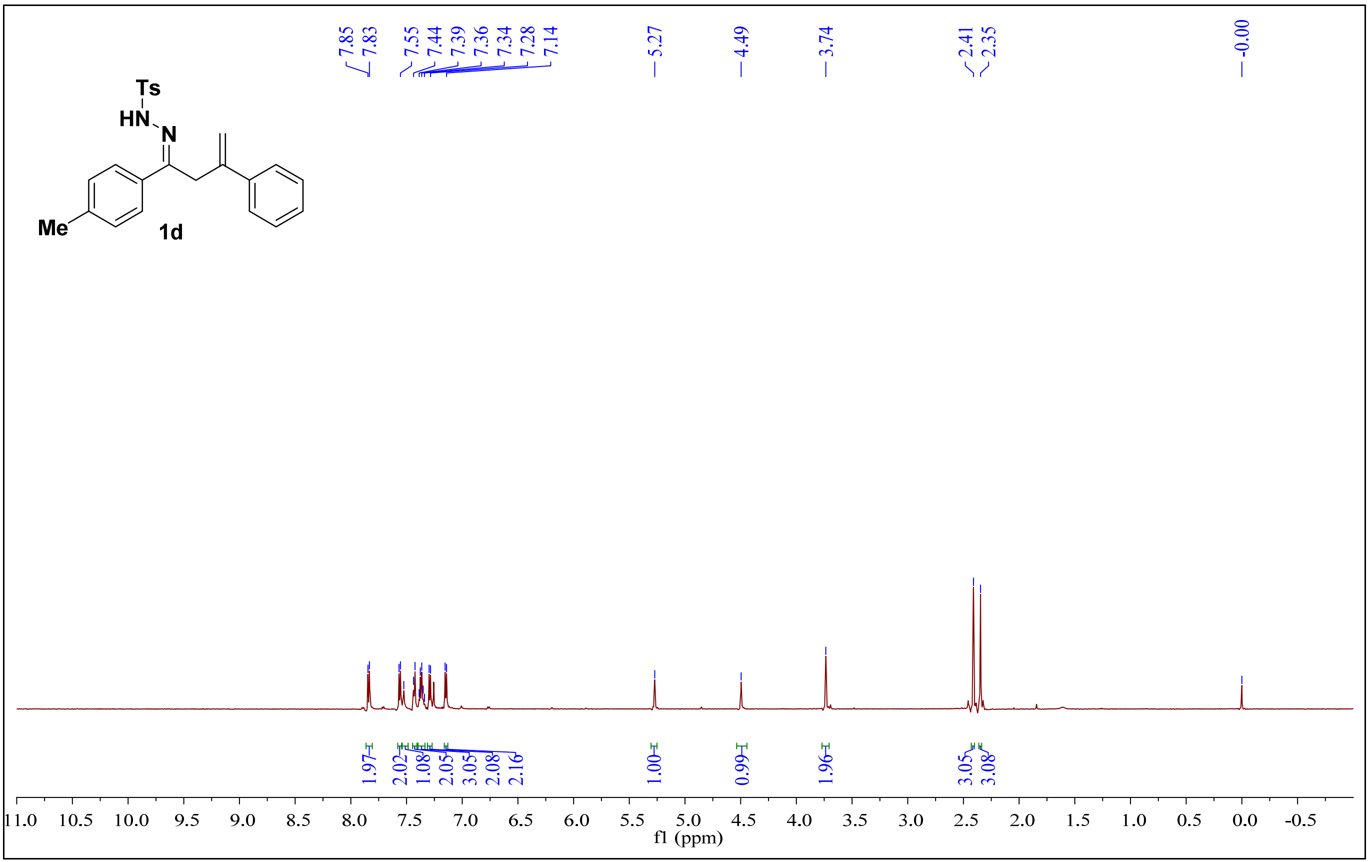

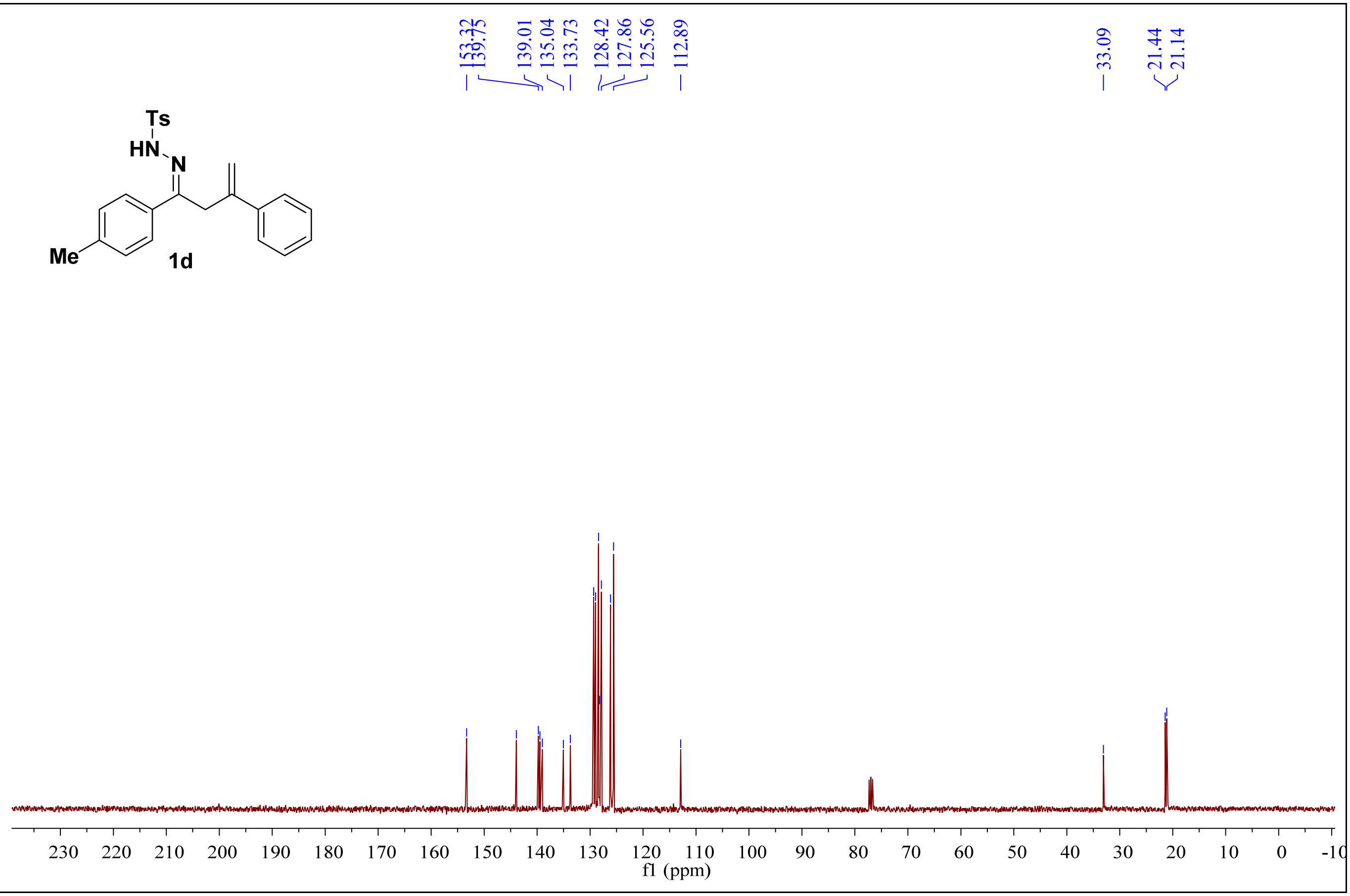

Supplementary Figure 4. ^{1}H **NMR (600 MHz,** $CDCl_3$**) and** ^{13}C **NMR (100 MHz,** $CDCl_3$**) spectra of hydrazone 1e**

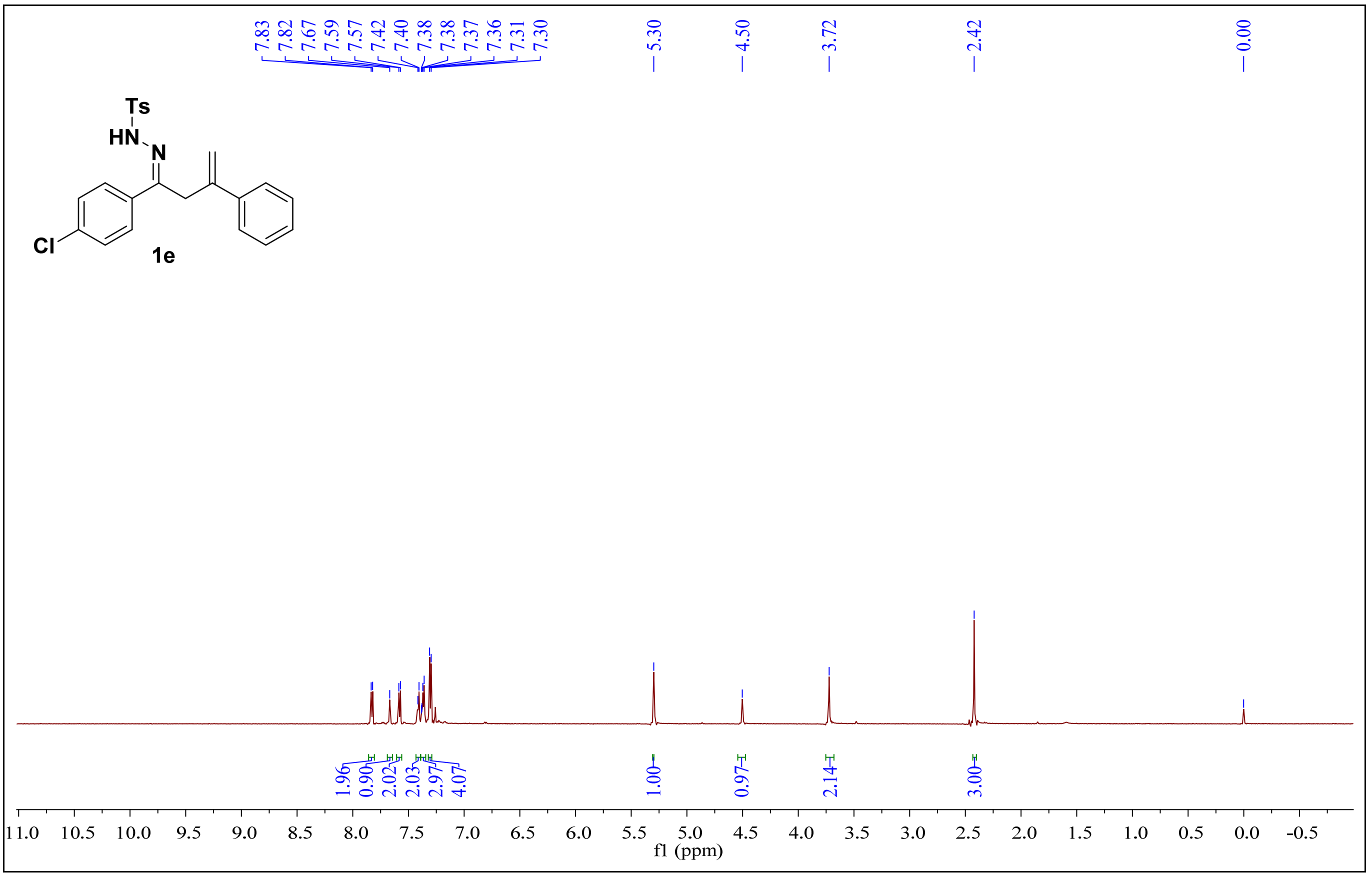

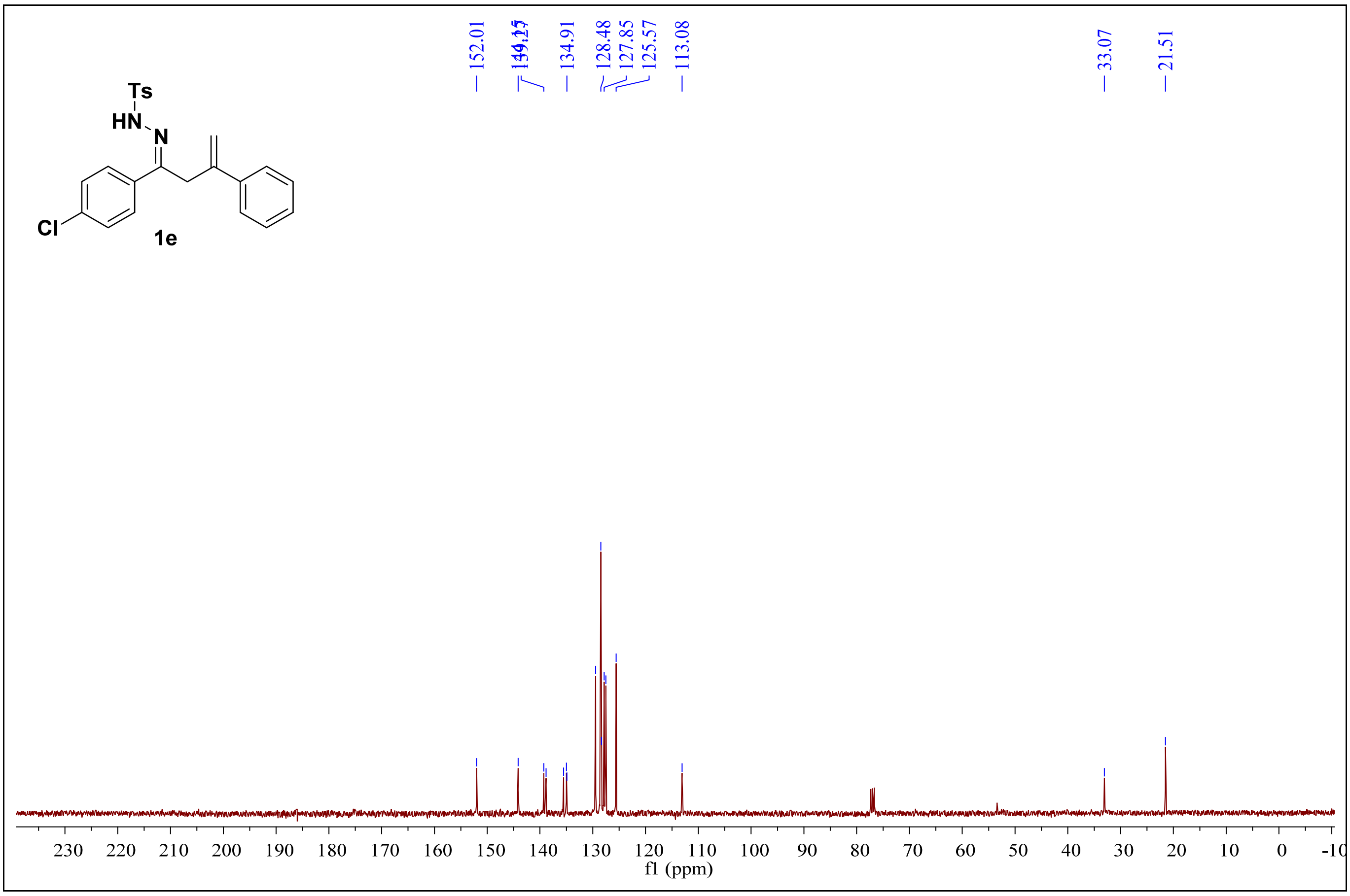

Supplementary Figure 5. ^{1}H NMR (600 MHz, $CDCl_3$) and ^{13}C NMR (100 MHz, $CDCl_3$) spectra of hydrazone 1f

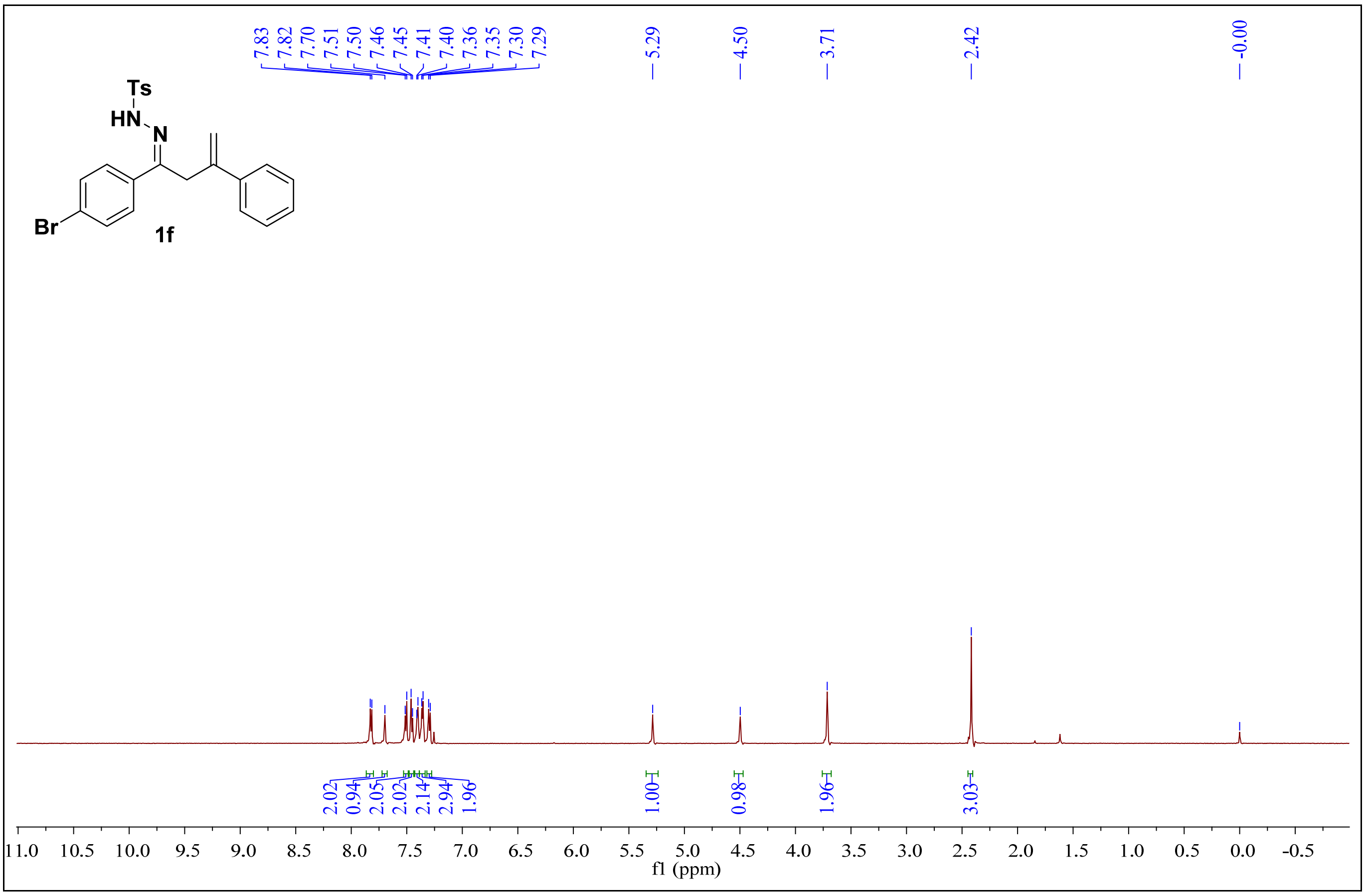

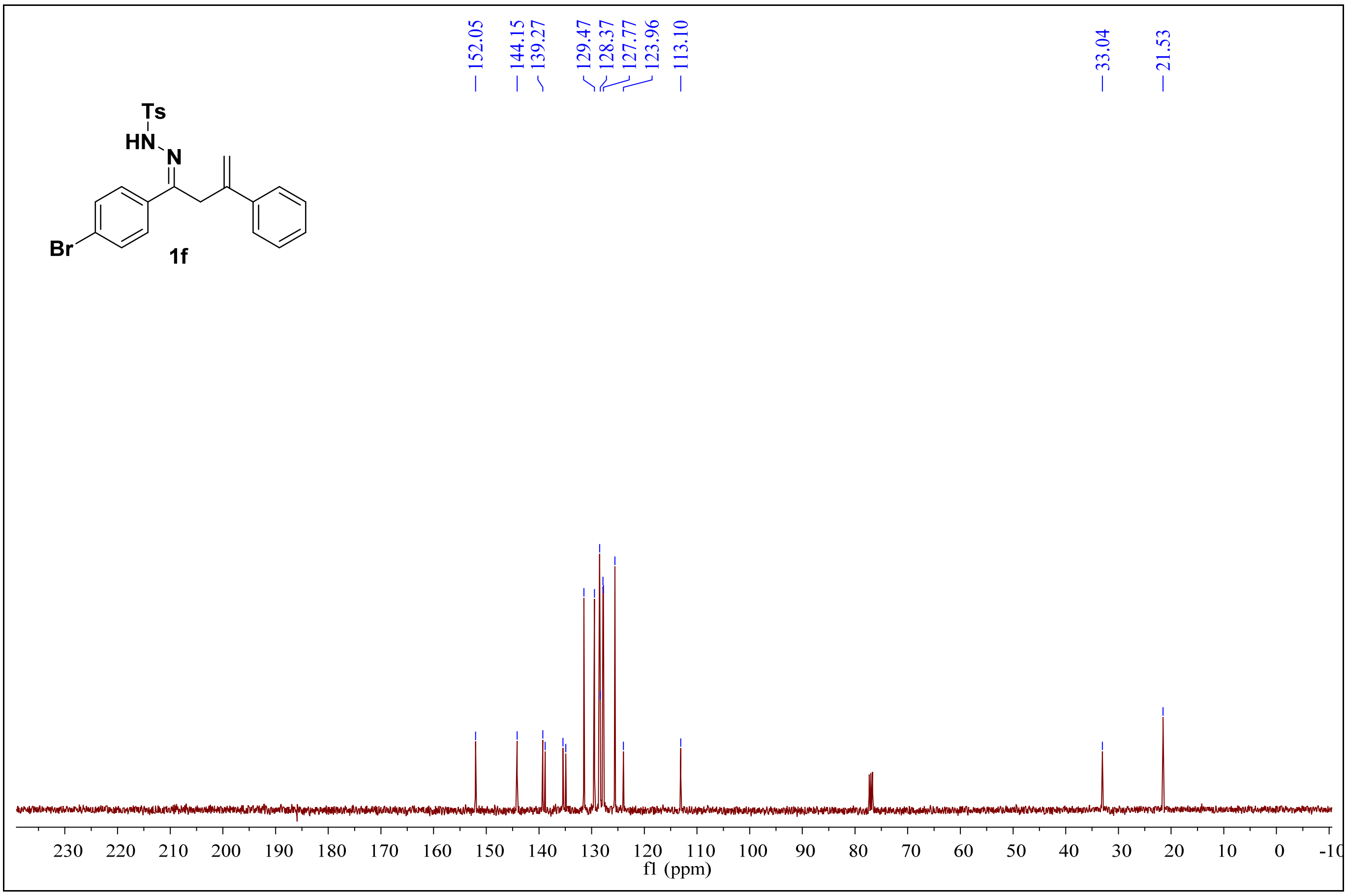

Supplementary Figure 6. **^{1}H NMR (400 MHz, $CDCl_3$), ^{13}C NMR (100 MHz, $CDCl_3$) and ^{19}F (376 MHz, $CDCl_3$) spectra of hydrazone 1g**

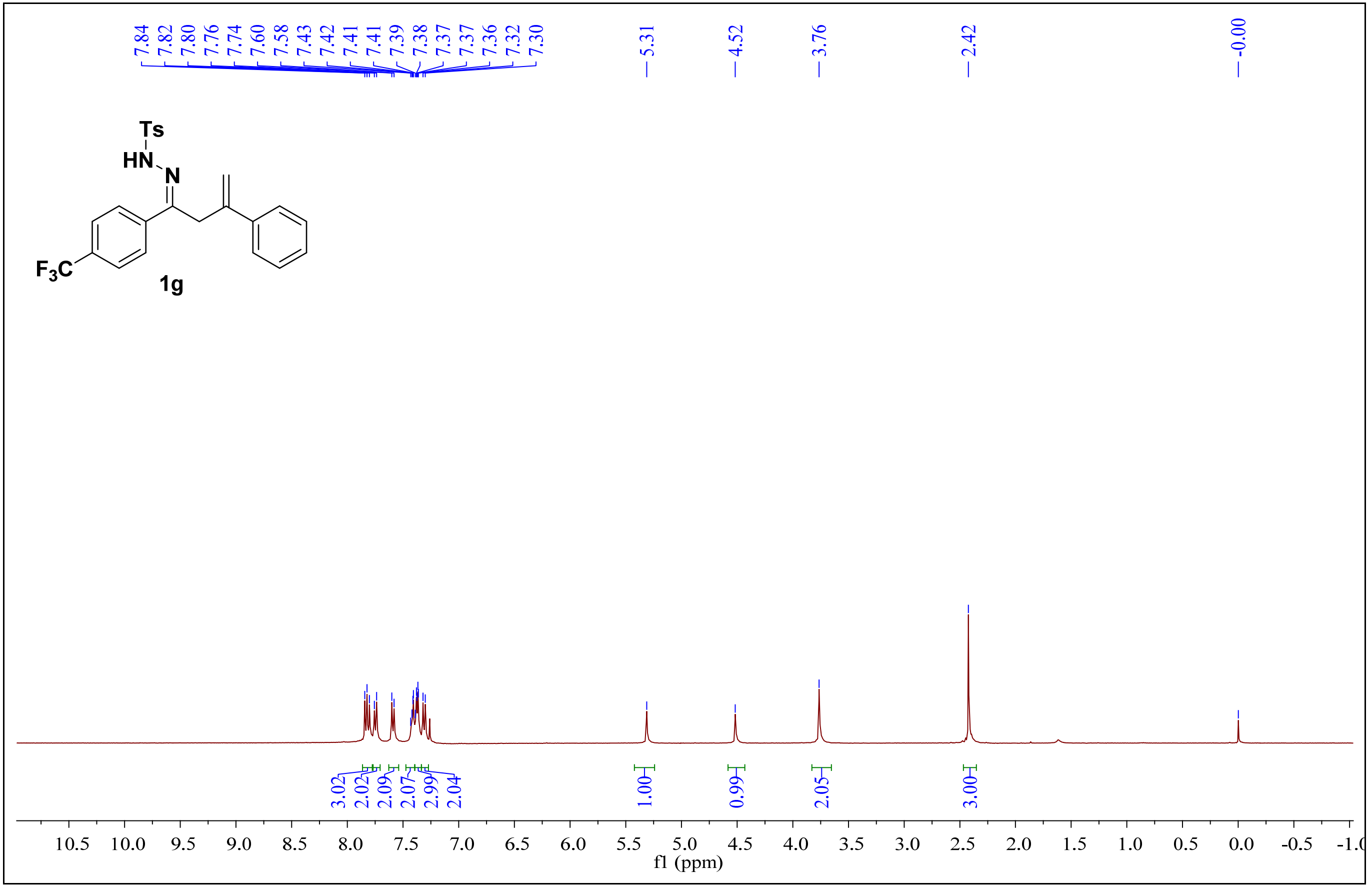

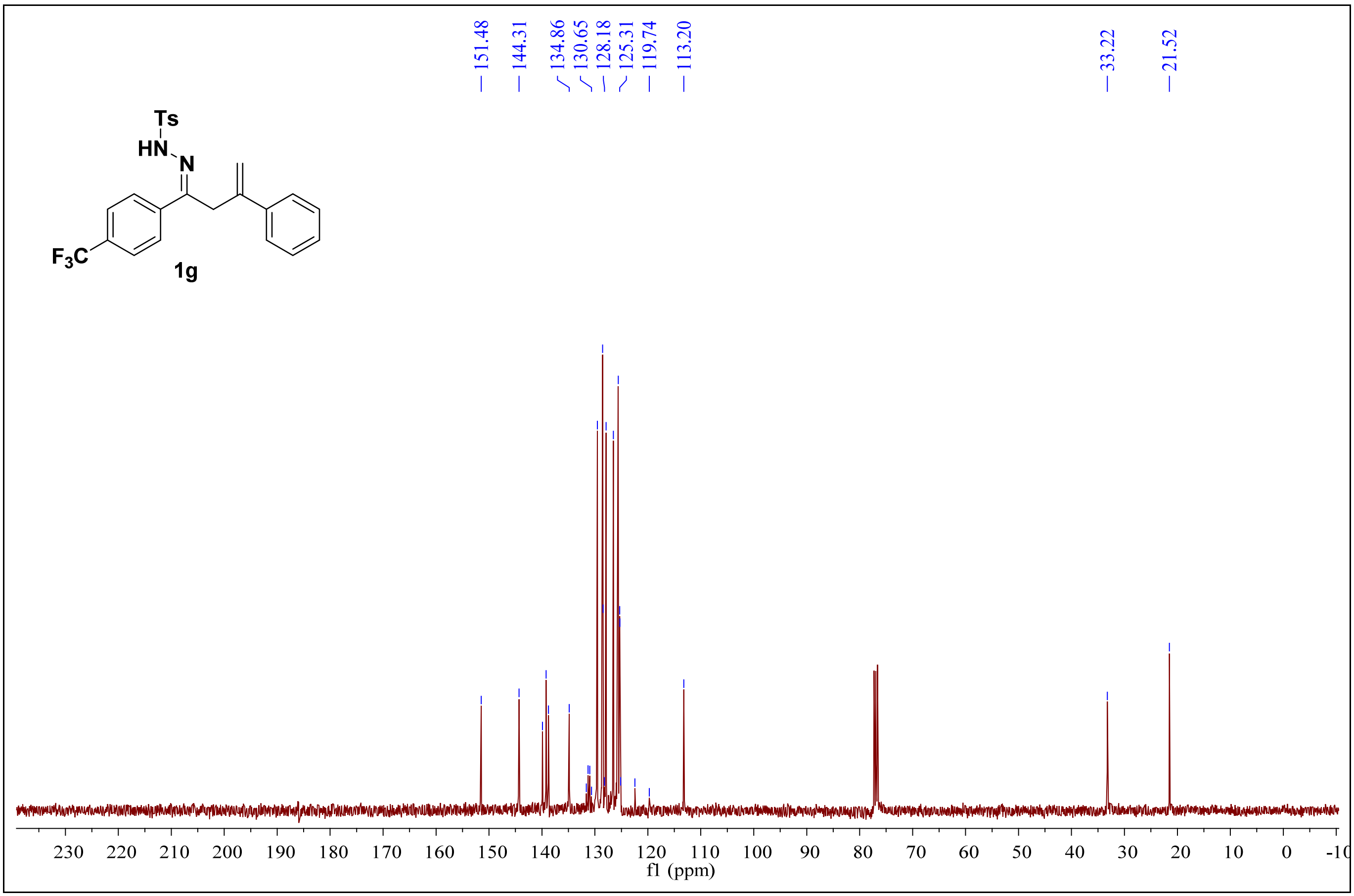

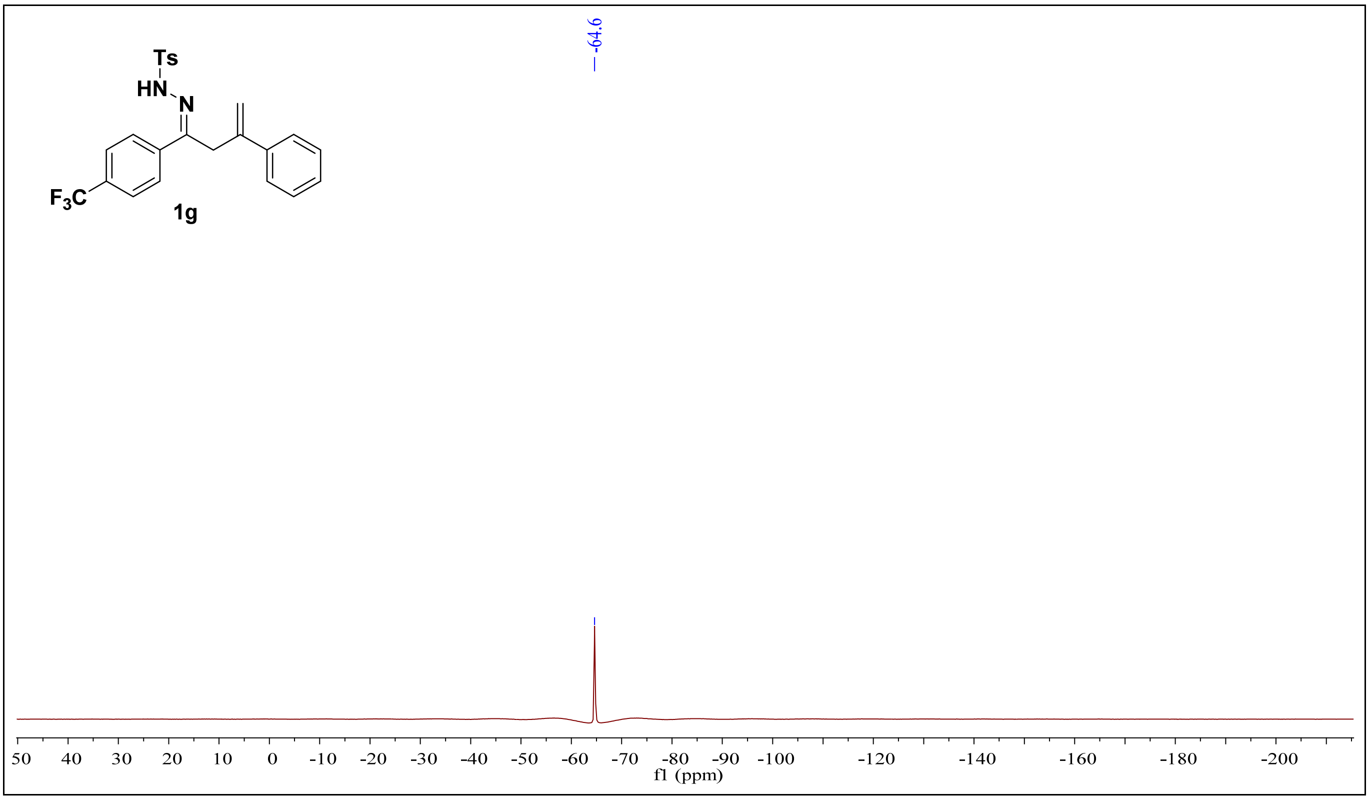
1g
-64.6
50
40
30
20
10
0
-10
-20
-30
-40
-50
-60
-70
-80
-90
-100
-120
-140
-160
-180
-200
f1 (ppm)

Supplementary Figure 7. ^{1}H NMR (600 MHz, $CDCl_3$) and ^{13}C NMR (100 MHz, $CDCl_3$) spectra of hydrazone 1h

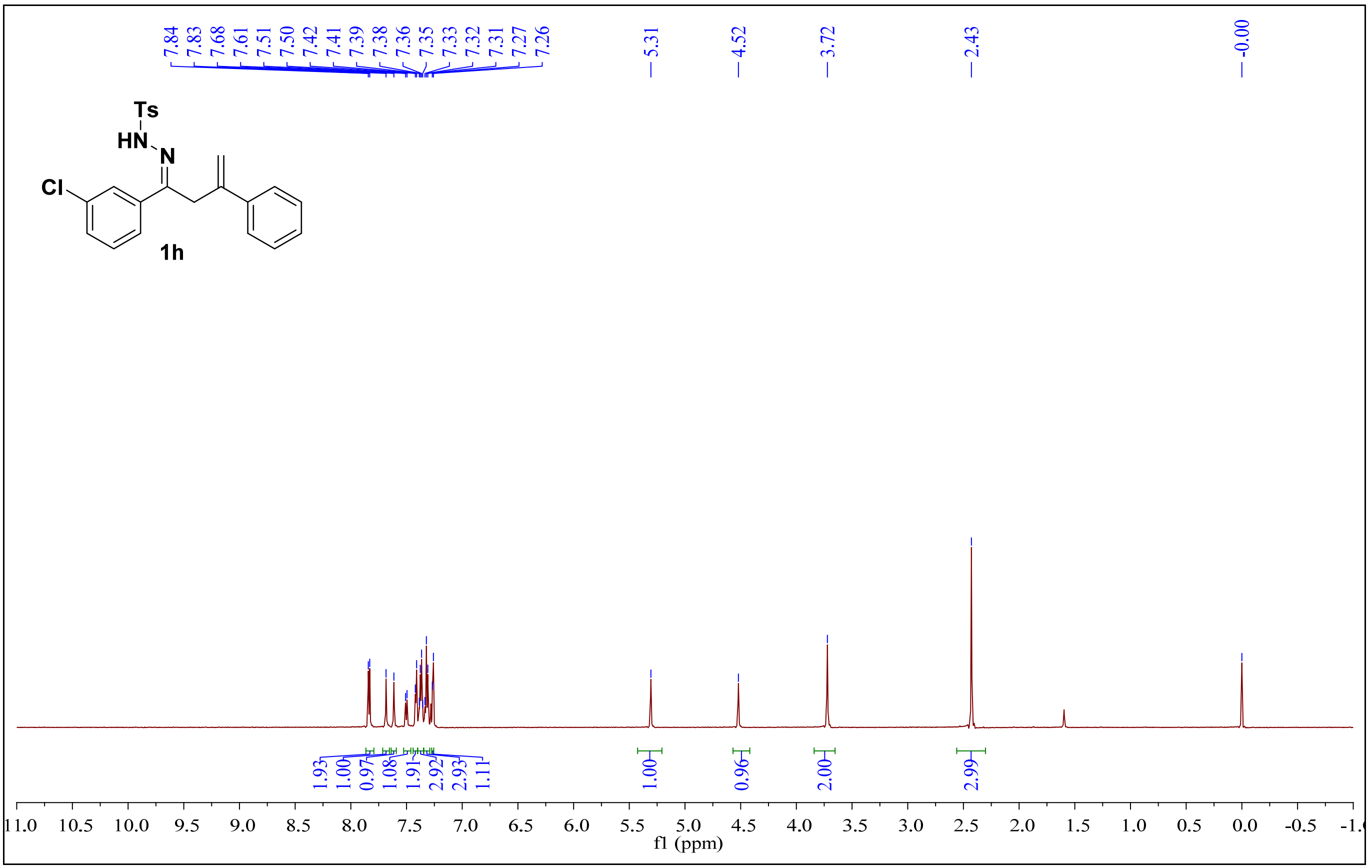

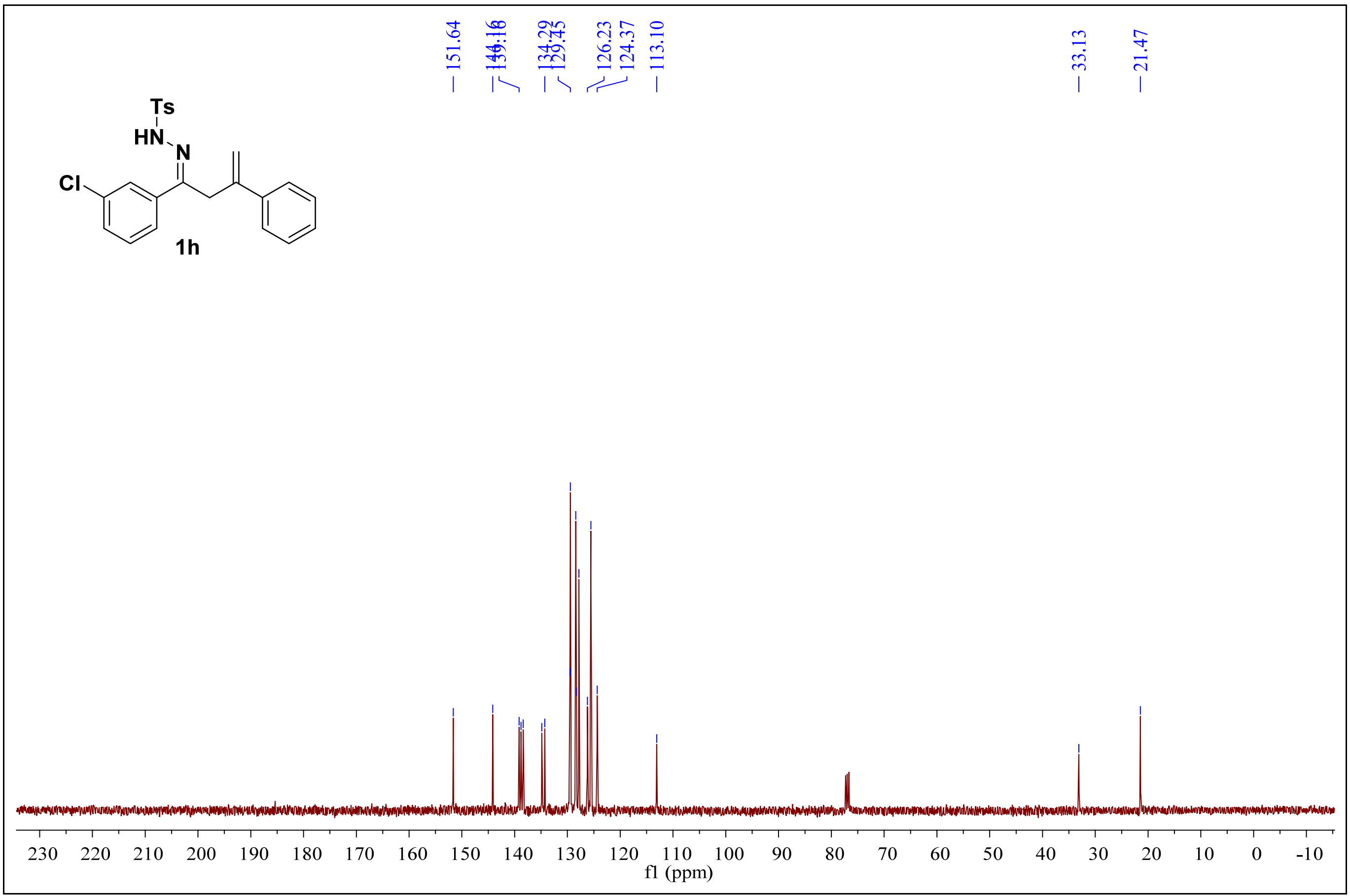

Supplementary Figure 8. ^{1}H NMR (400 MHz, $CDCl_3$) and ^{13}C NMR (100 MHz, $CDCl_3$) spectra of hydrazone 1i

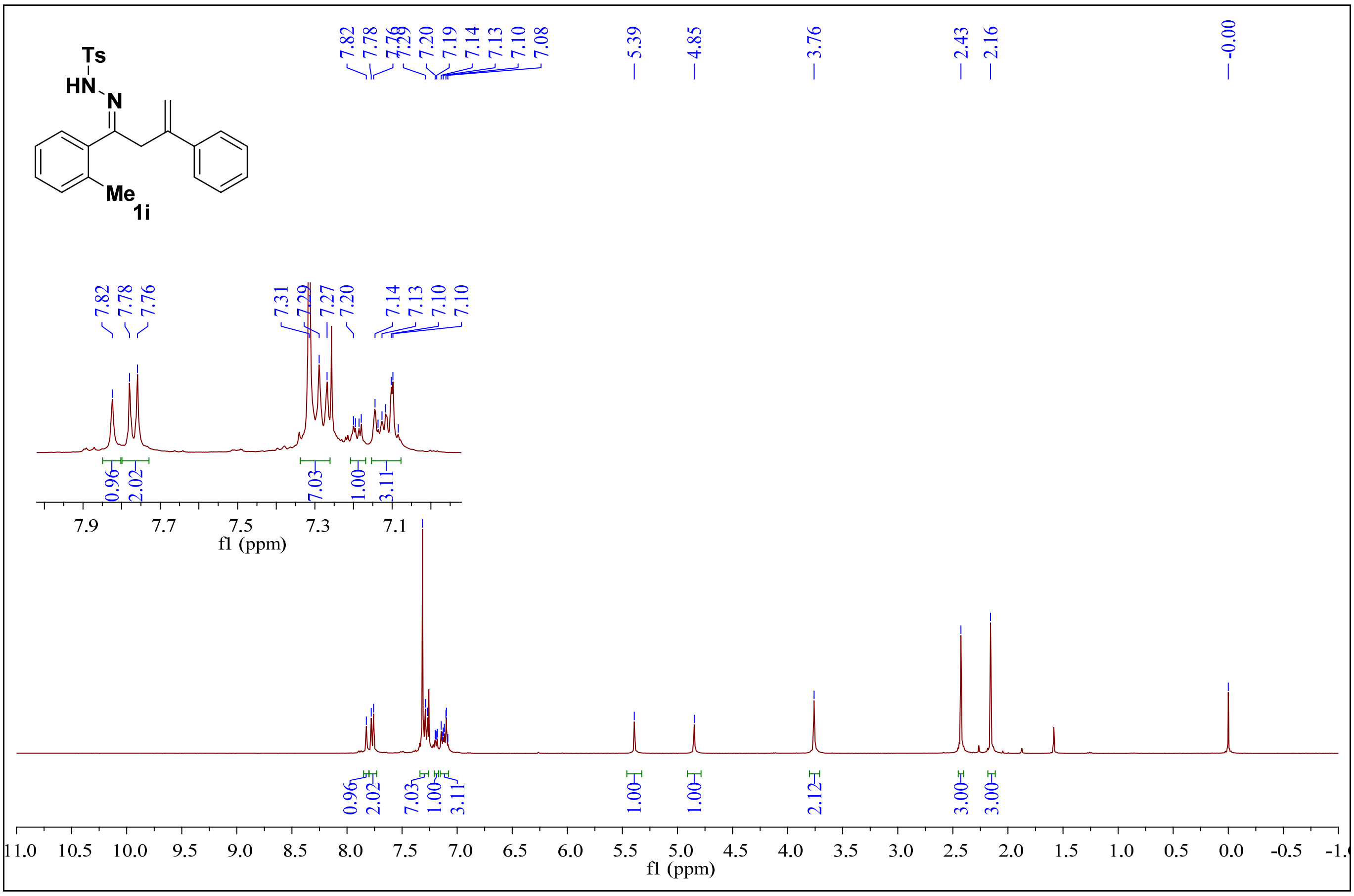

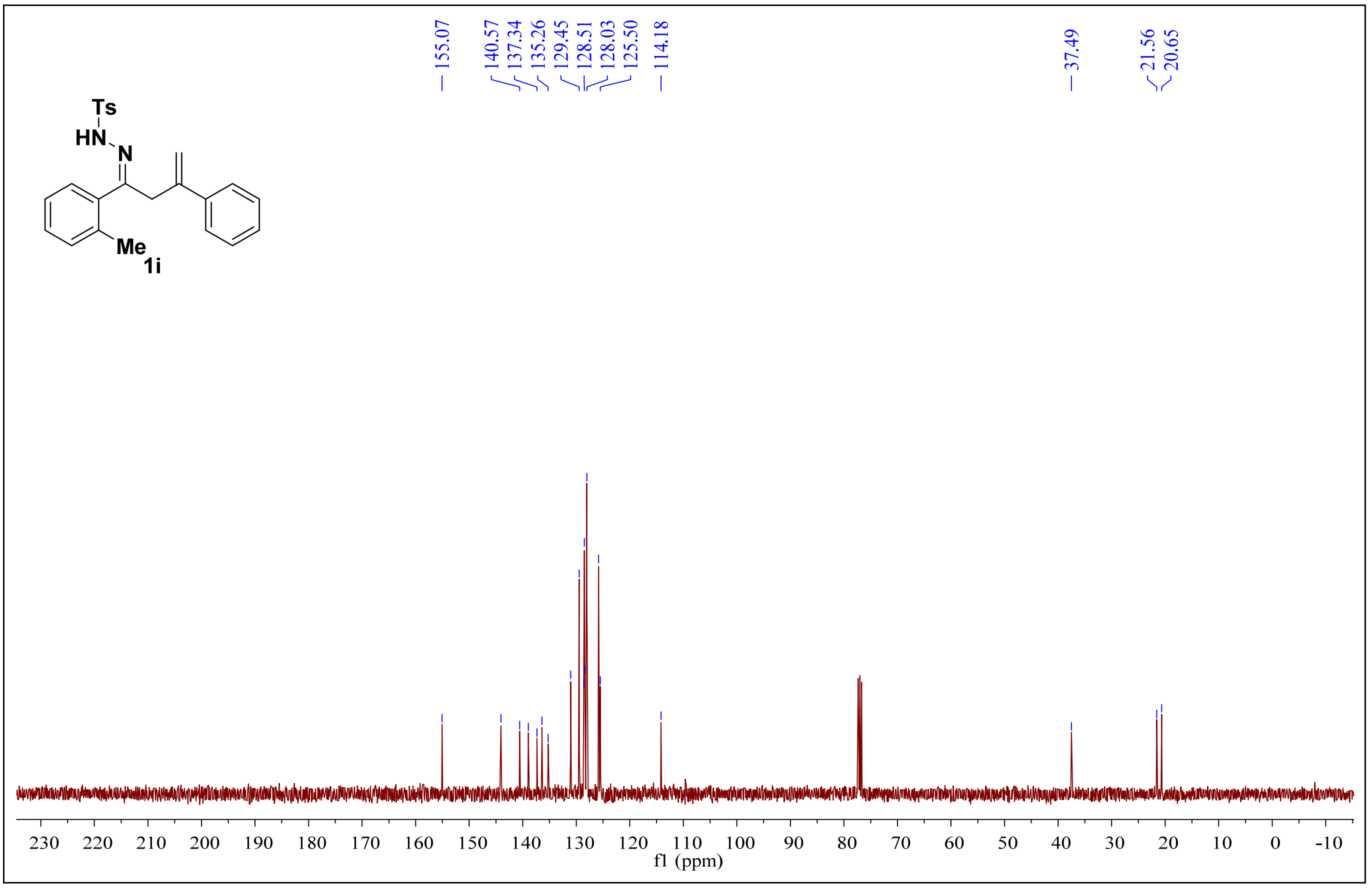

Supplementary Figure 9. ^{1}H NMR (600 MHz, $CDCl_3$) and ^{13}C NMR (100 MHz, $CDCl_3$) spectra of hydrazone 1j

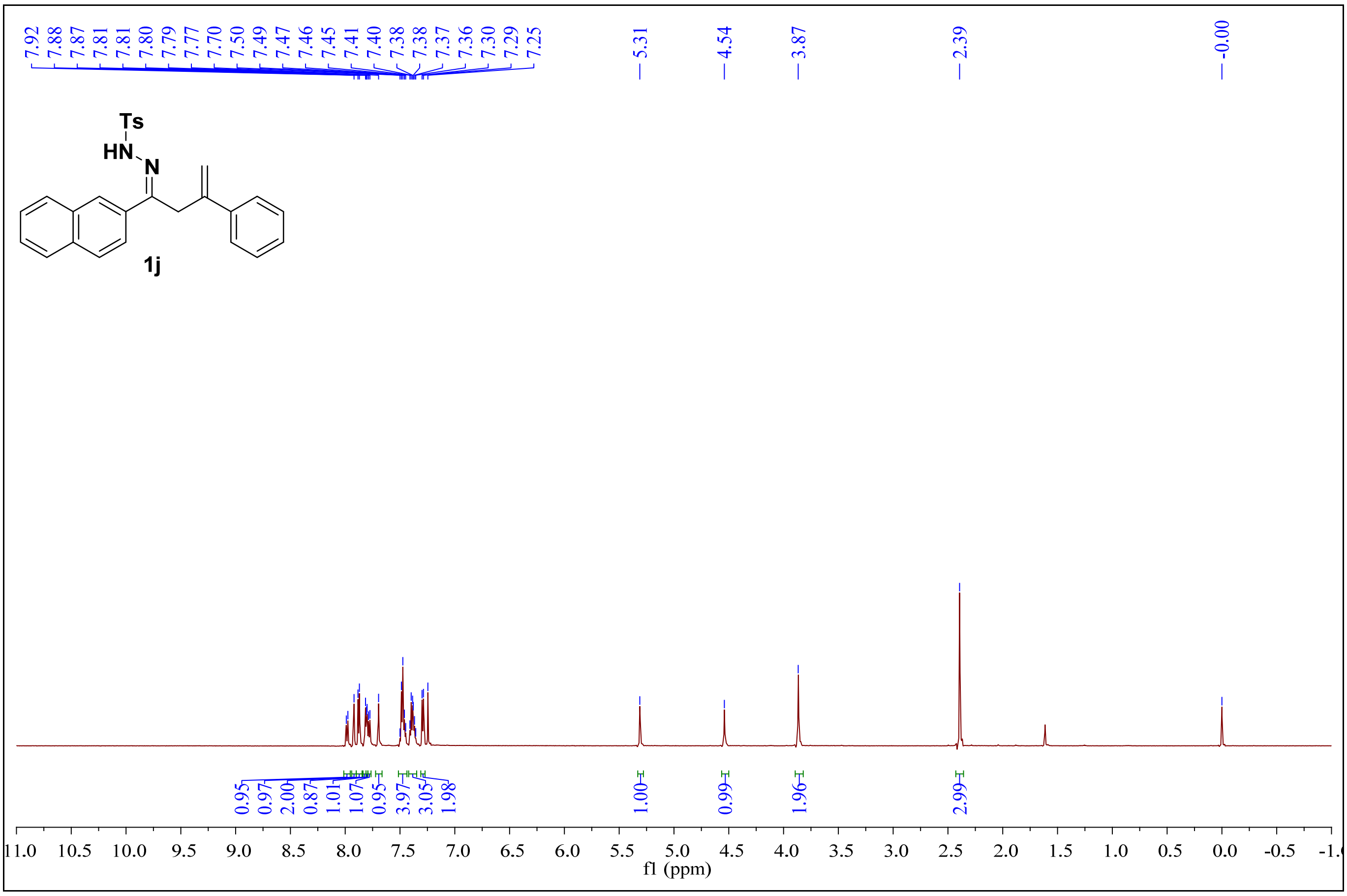

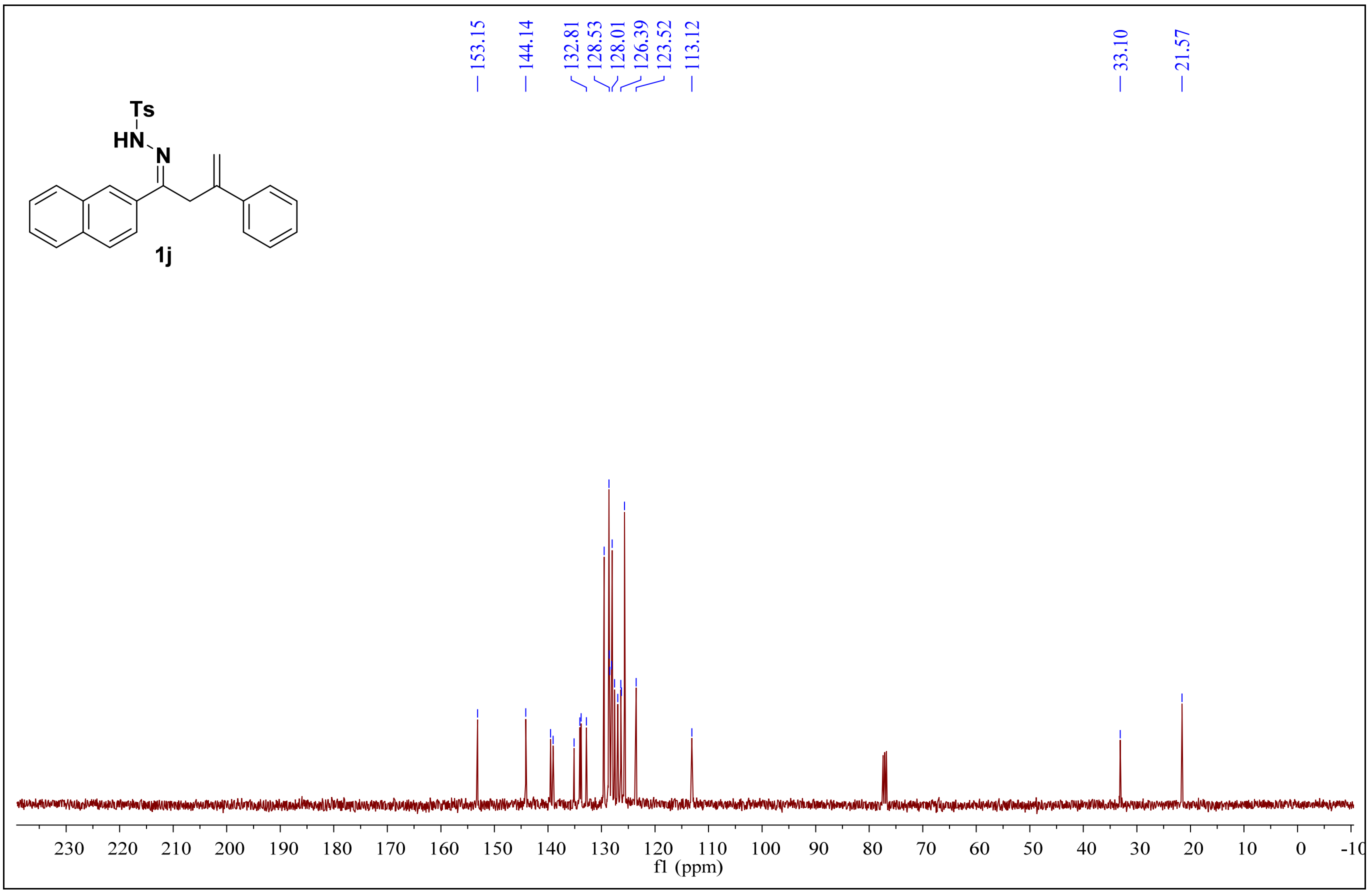

Supplementary Figure 10. **^{1}H NMR (400 MHz, $CDCl_3$) and ^{13}C NMR (100 MHz, $CDCl_3$) spectra of hydrazone 1k**

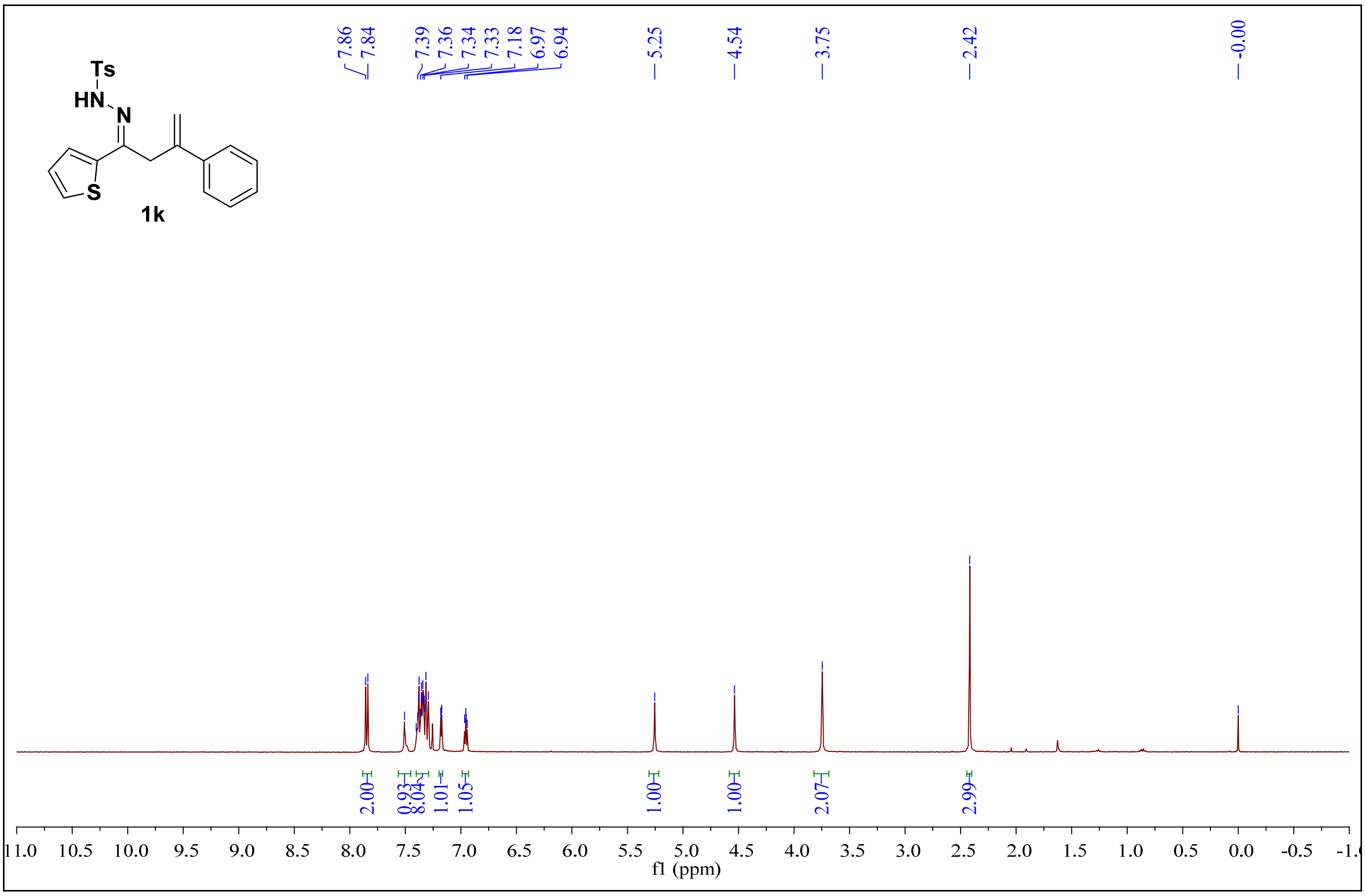

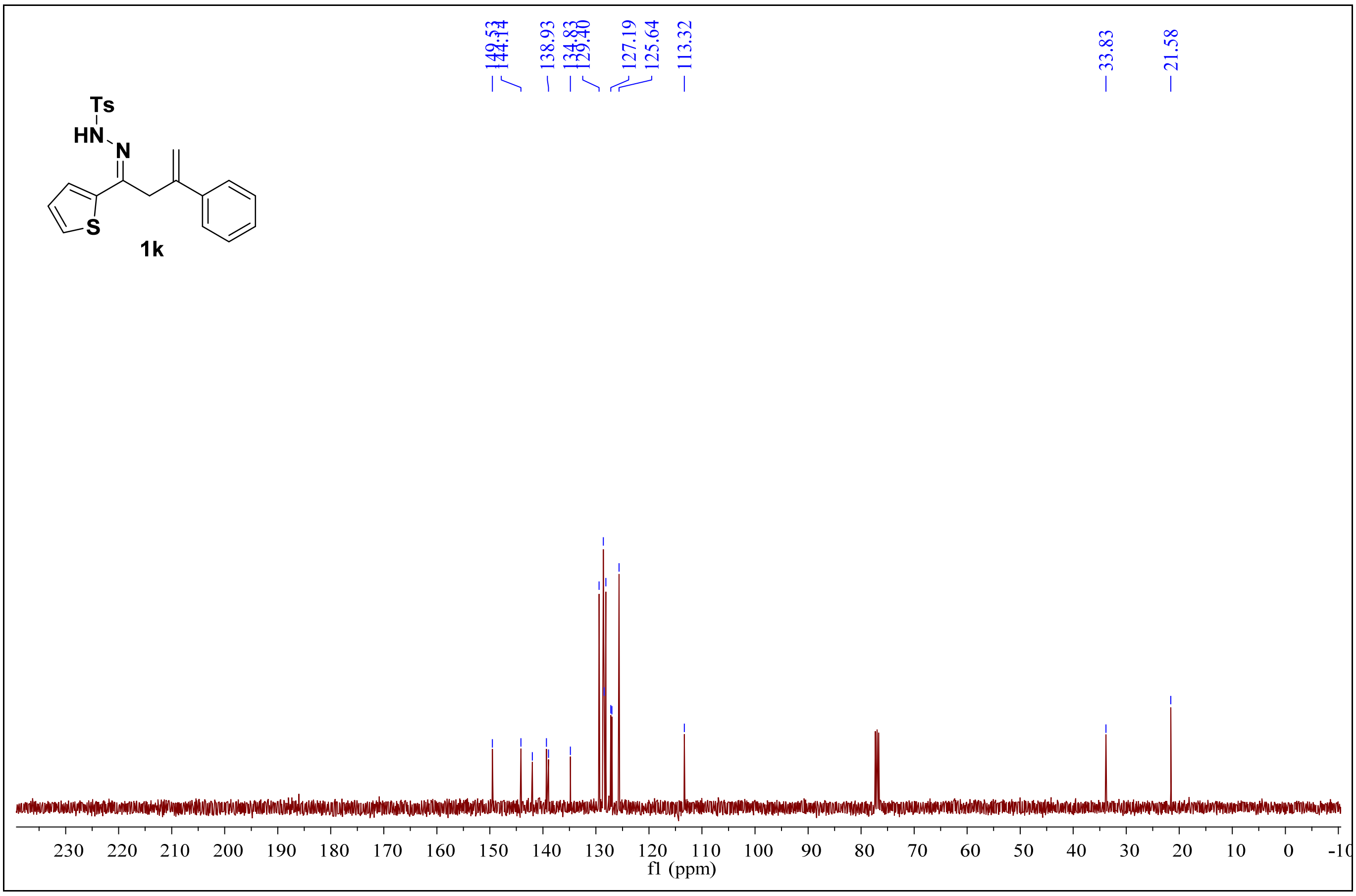

Supplementary Figure 11. **^{1}H NMR (600 MHz, $CDCl_3$) and ^{13}C NMR (100 MHz, $CDCl_3$) spectra of hydrazone 1l**

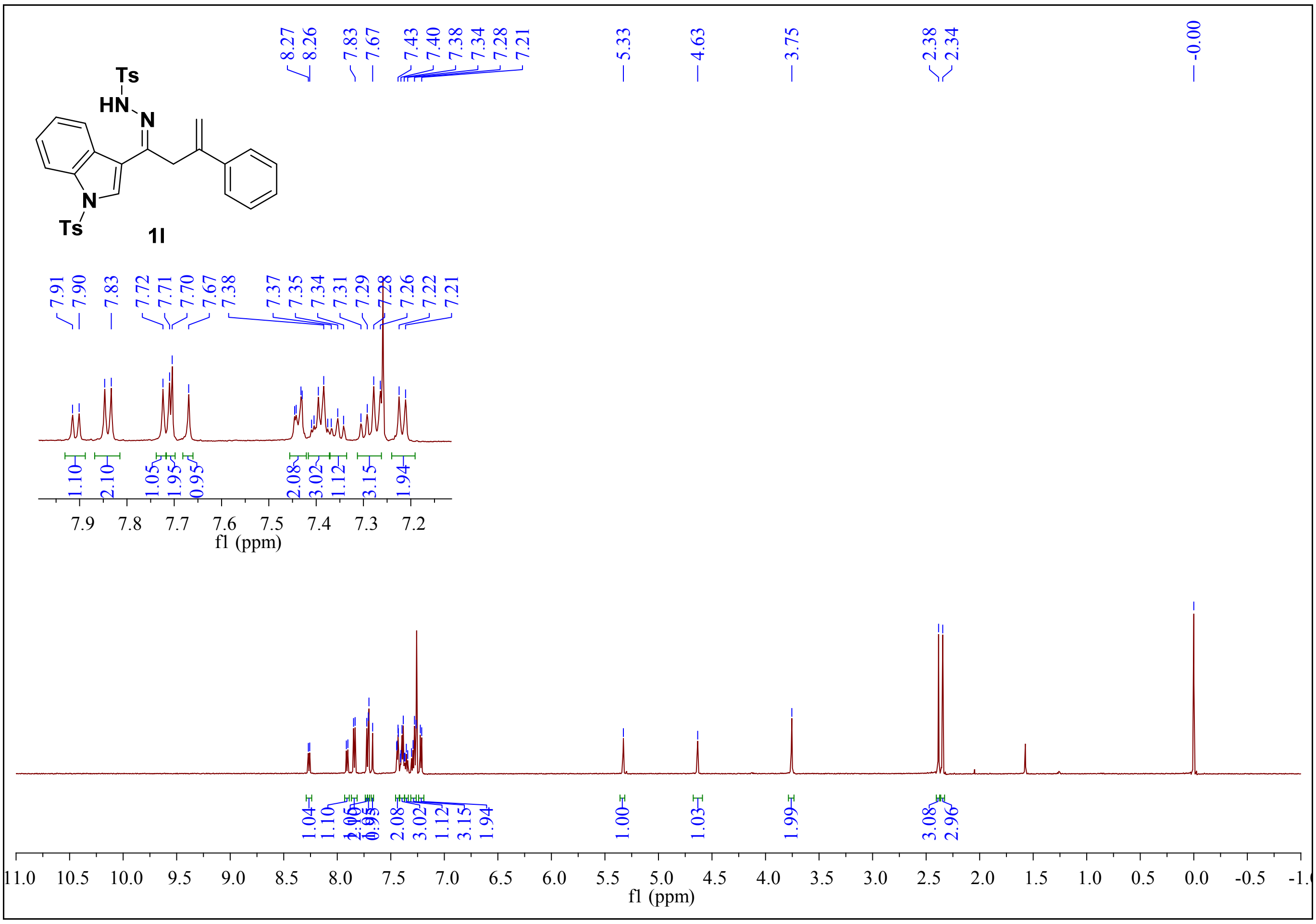

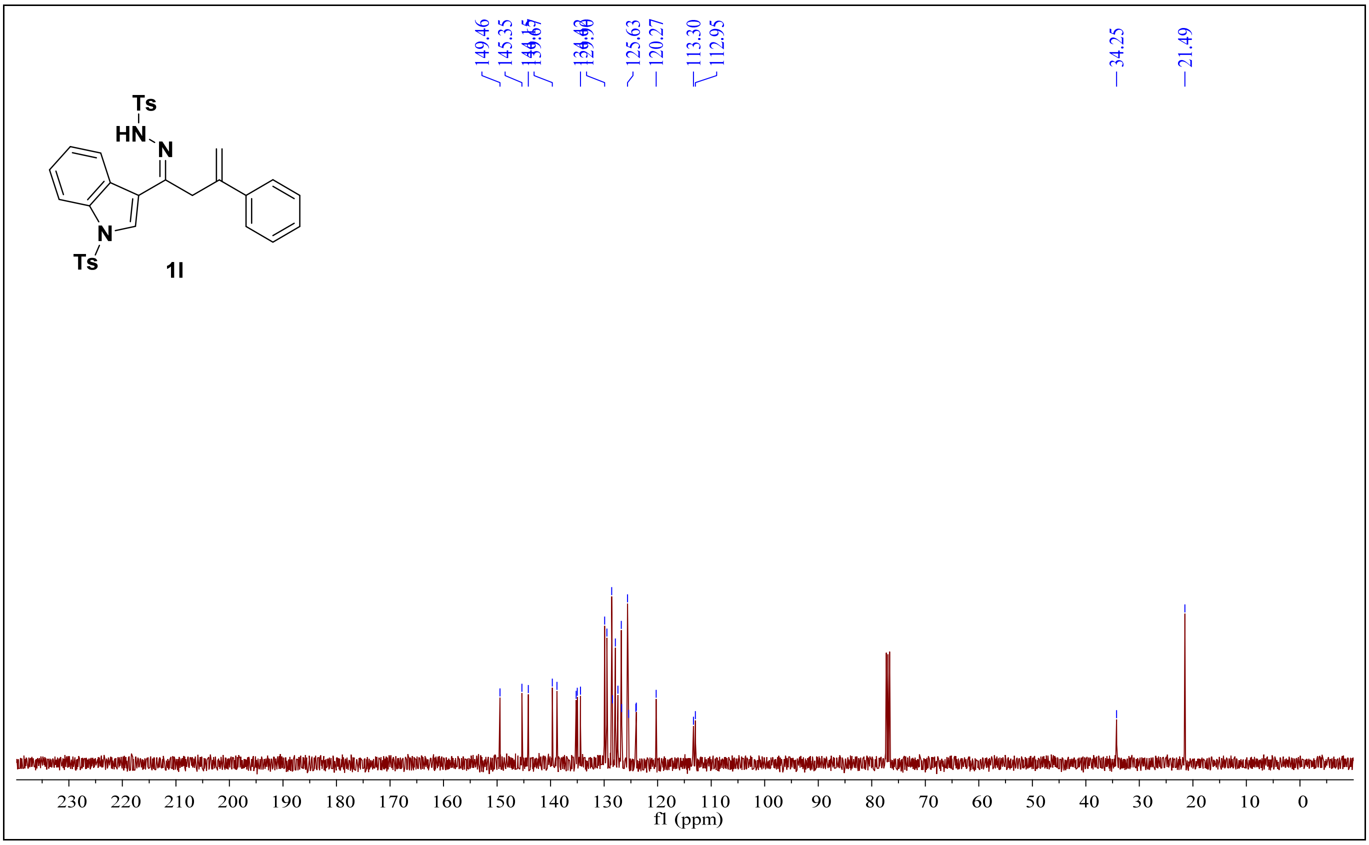

Supplementary Figure 12. **^{1}H NMR (600 MHz, $CDCl_3$) and ^{13}C NMR (100 MHz, $CDCl_3$) spectra of hydrazone 1m**

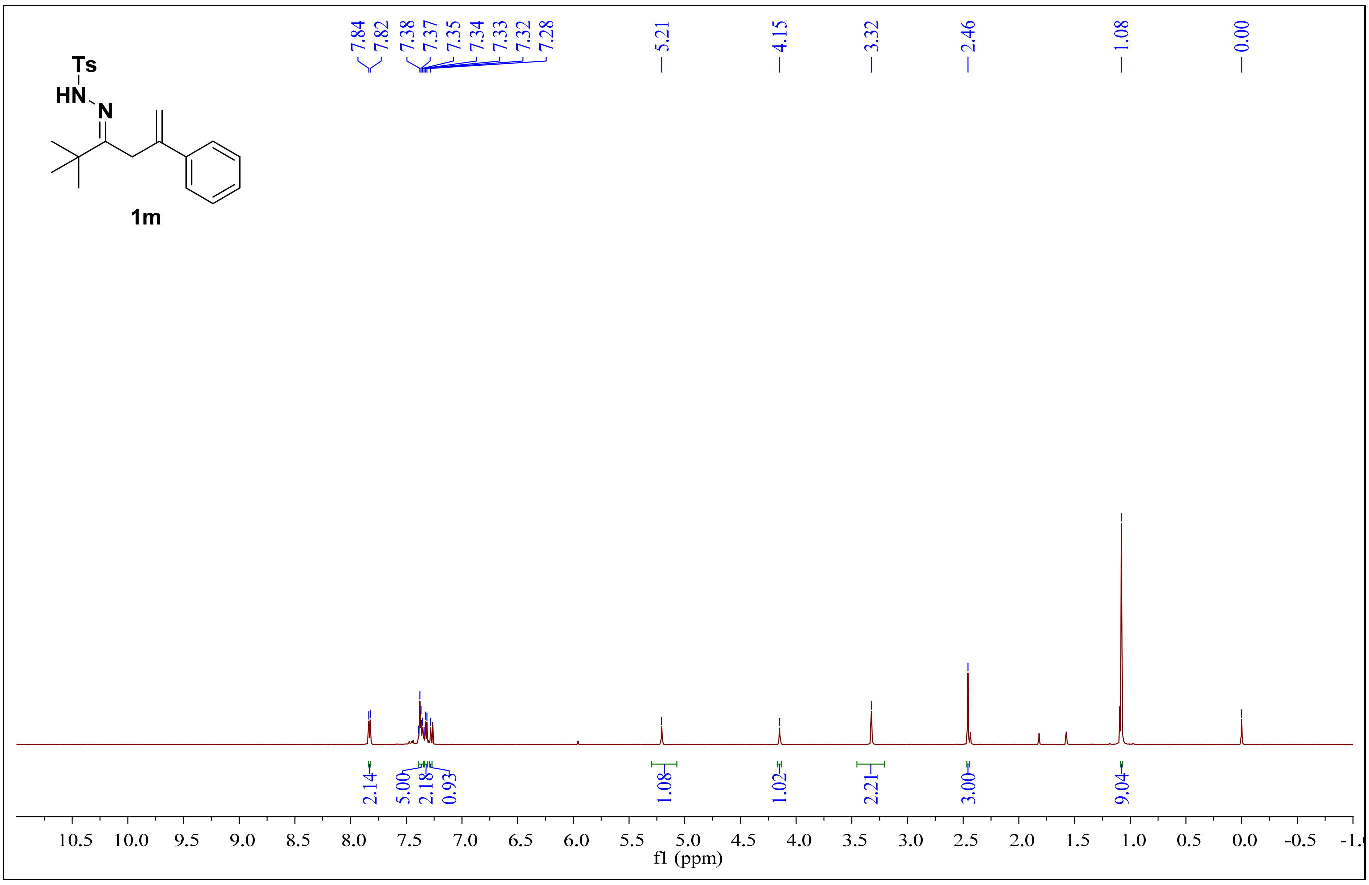

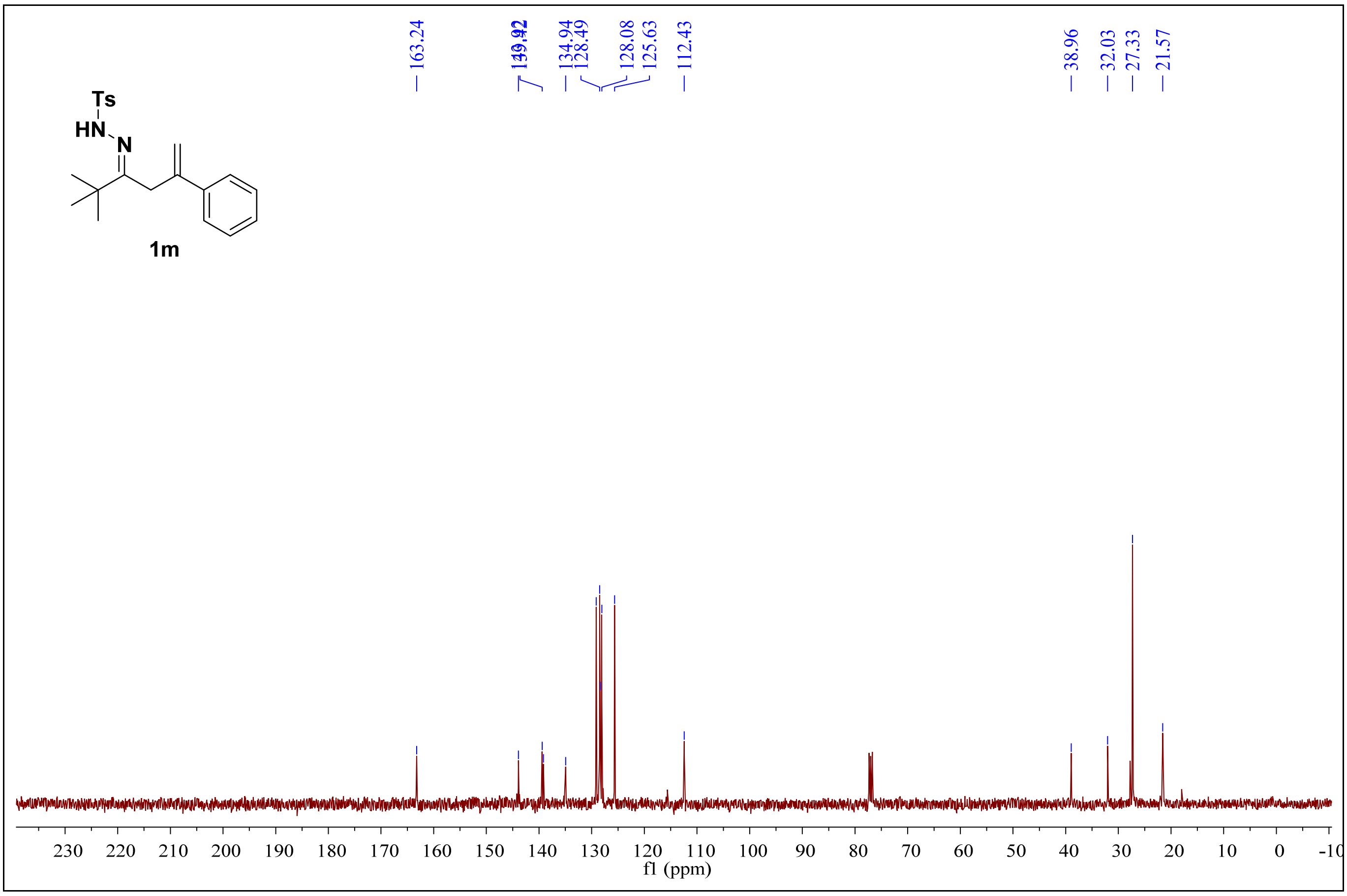

Supplementary Figure 13. **^{1}H NMR (400 MHz, DMSO-d^{6}) and ^{13}C NMR (100 MHz, $CDCl_3$) spectra of hydrazone 1n**

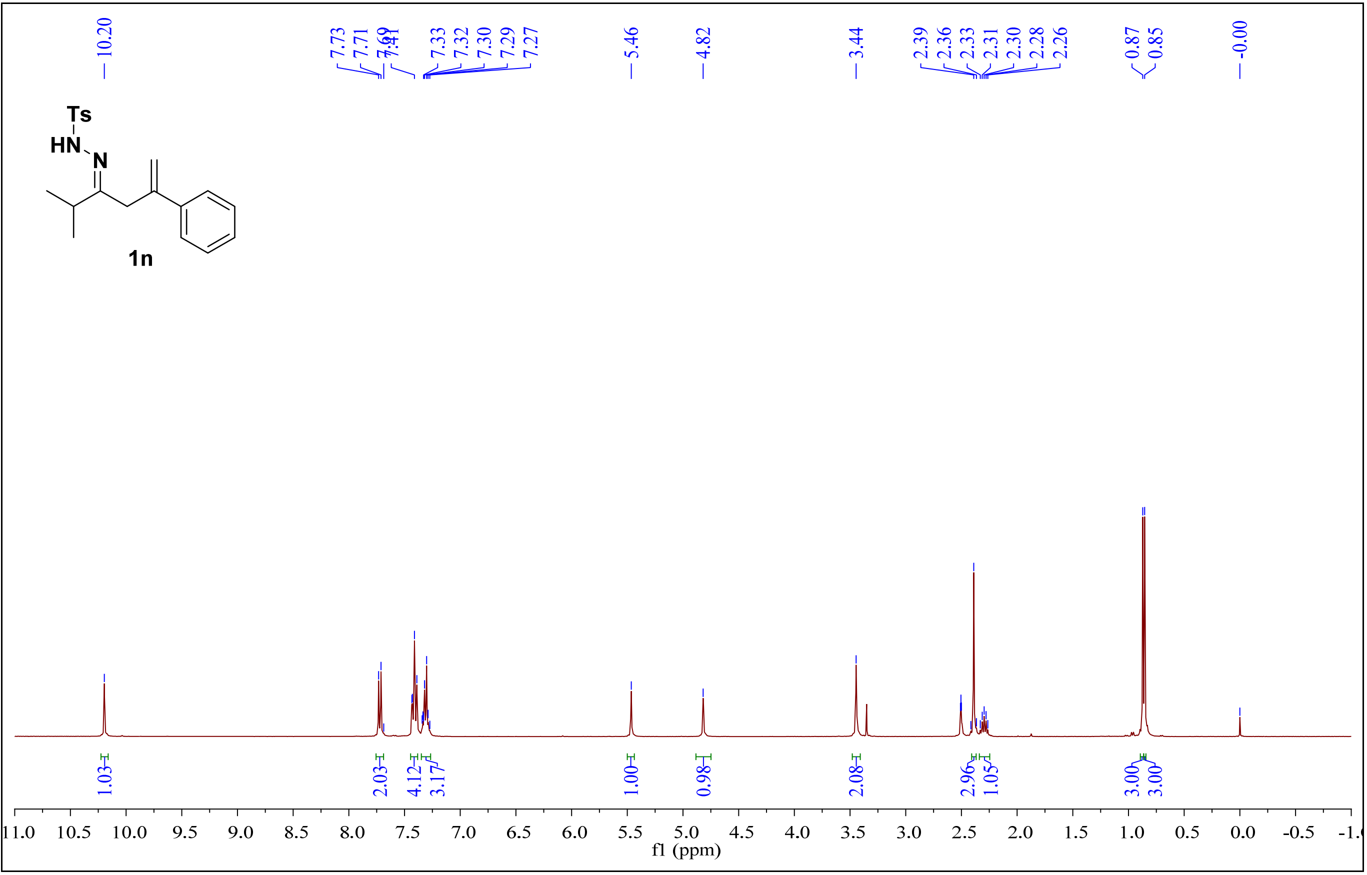

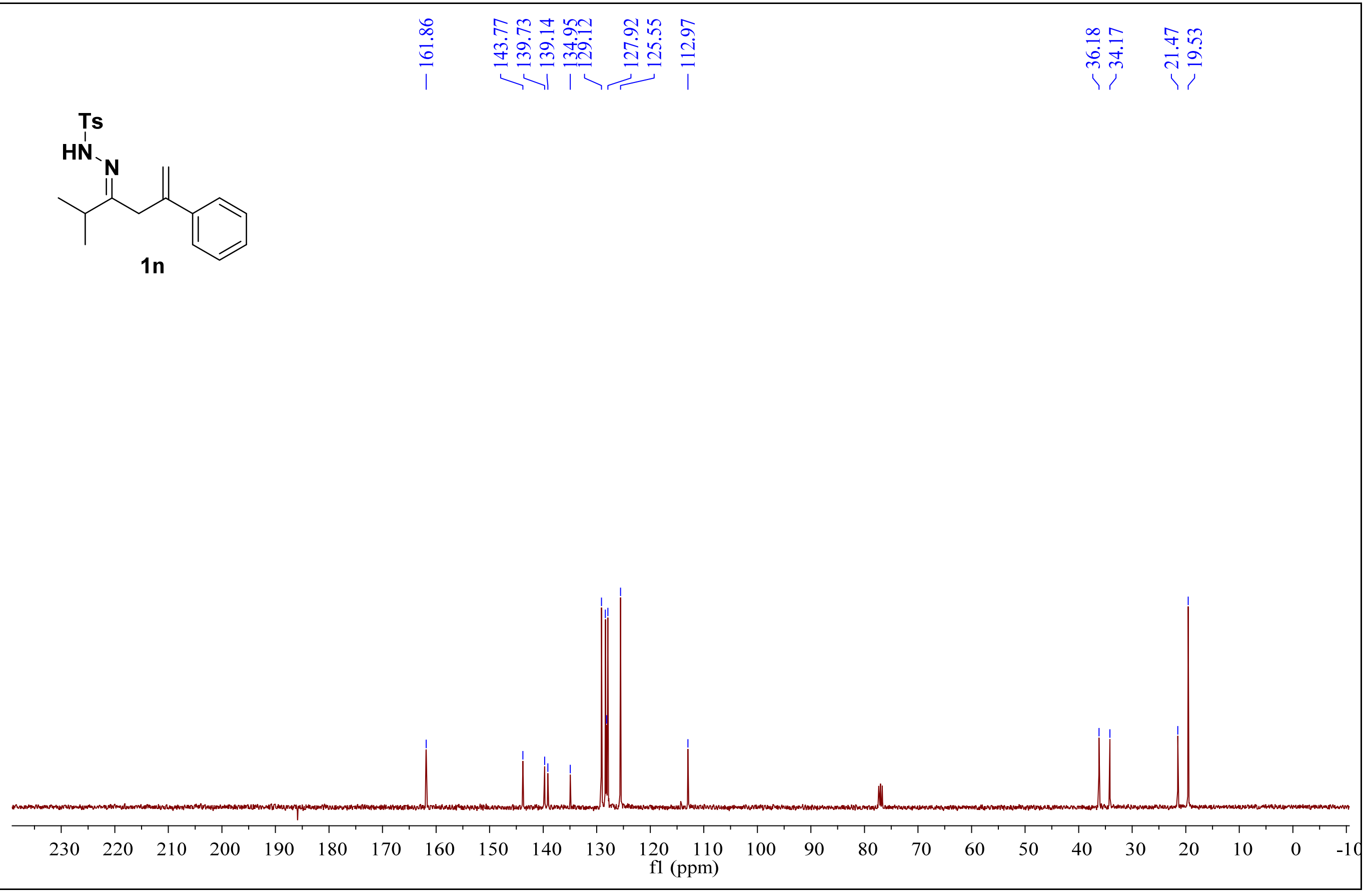

Supplementary Figure 14. ^{1}H NMR (400 MHz, DMSO-d^{6}) and ^{13}C NMR (100 MHz, $CDCl_3$) spectra of hydrazone **1o**

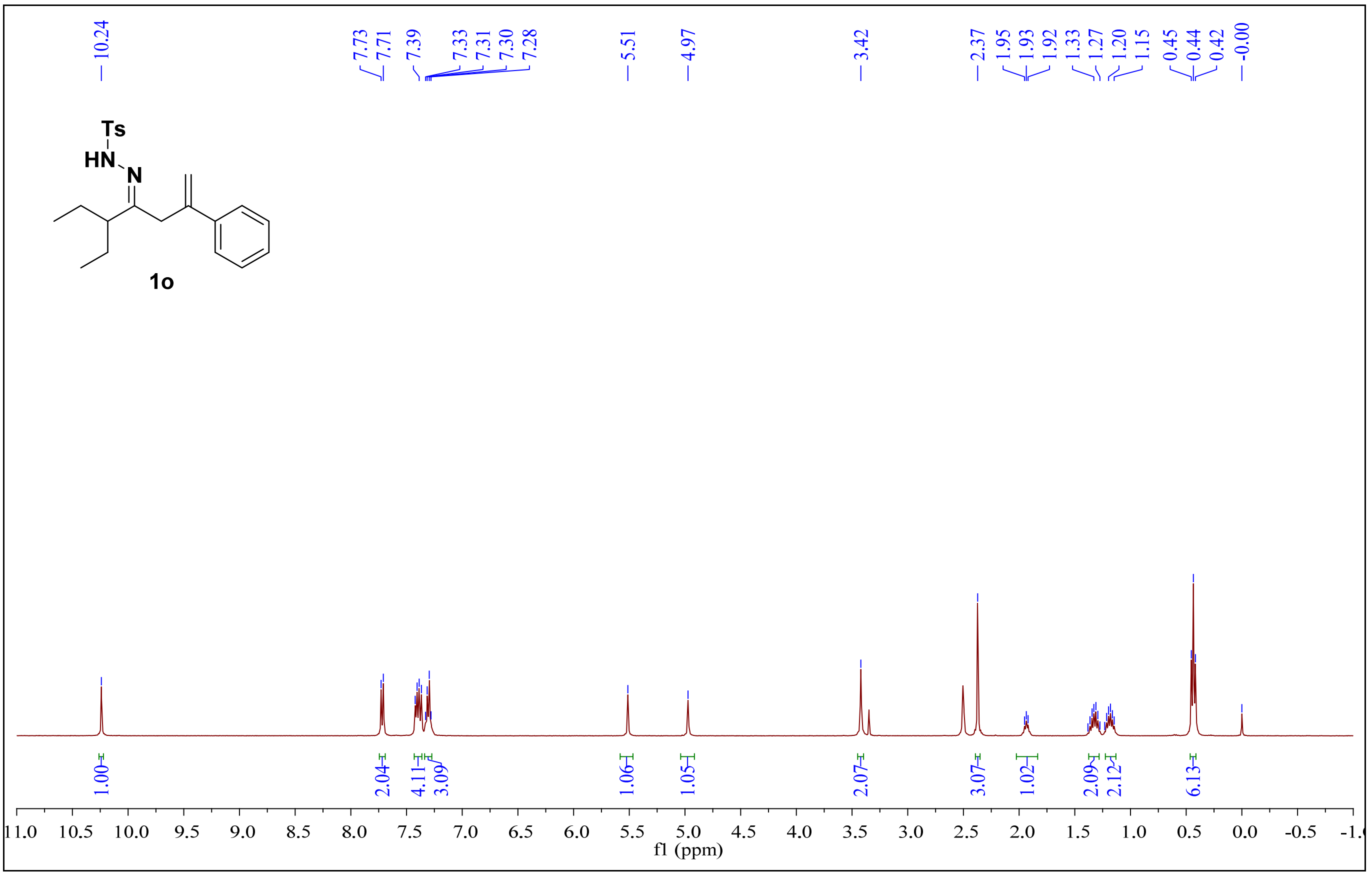

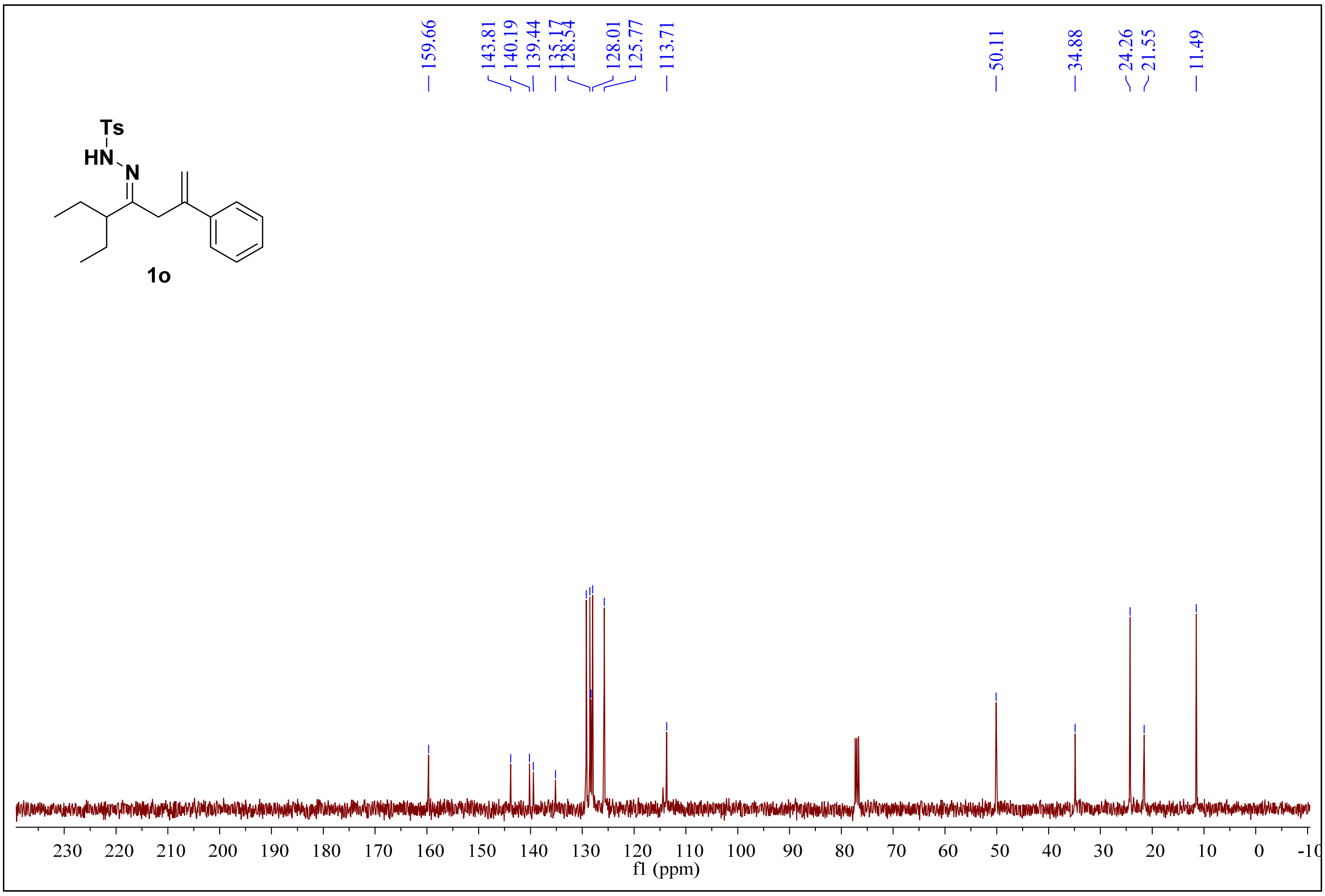

Supplementary Figure 15. ^{1}H **NMR (400 MHz,** $CDCl_3$**) and** ^{13}C **NMR (100 MHz,** $CDCl_3$**) spectra of hydrazone 1p**

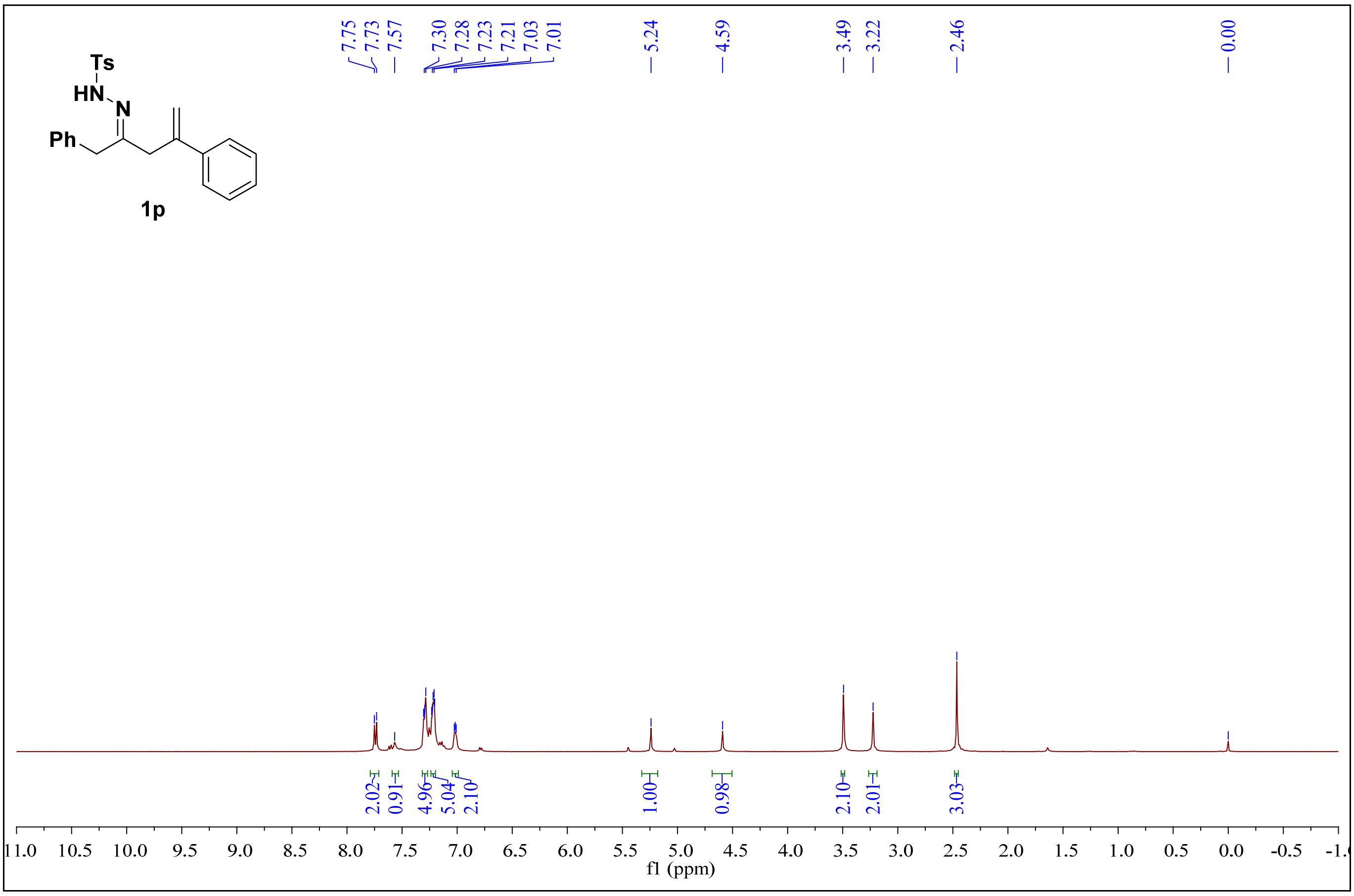

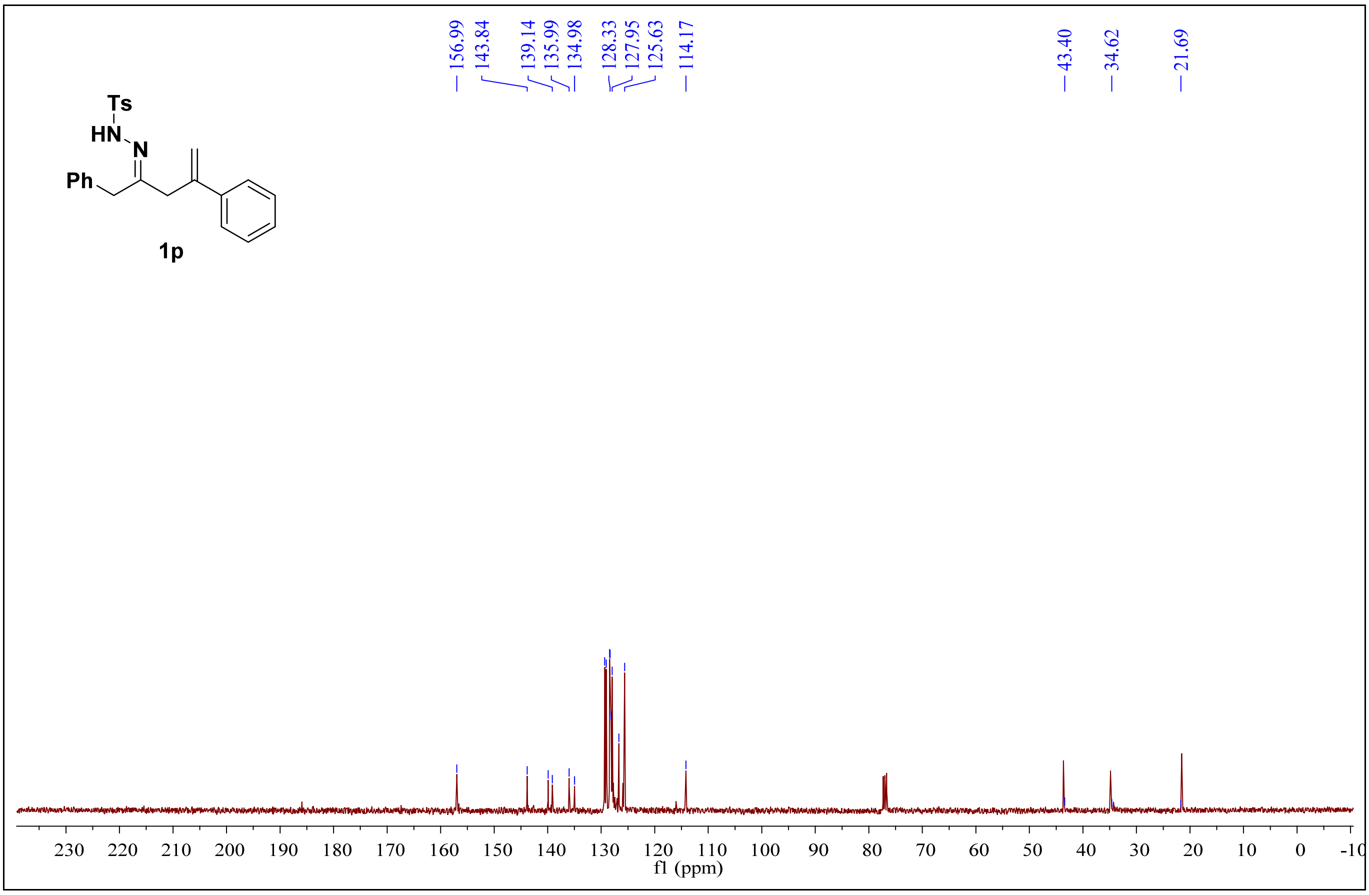

Supplementary Figure 16. ^{1}H NMR (400 MHz, $CDCl_3$) and ^{13}C NMR (100 MHz, $CDCl_3$) spectra of hydrazone 1q (major: minor = 1:0.6)

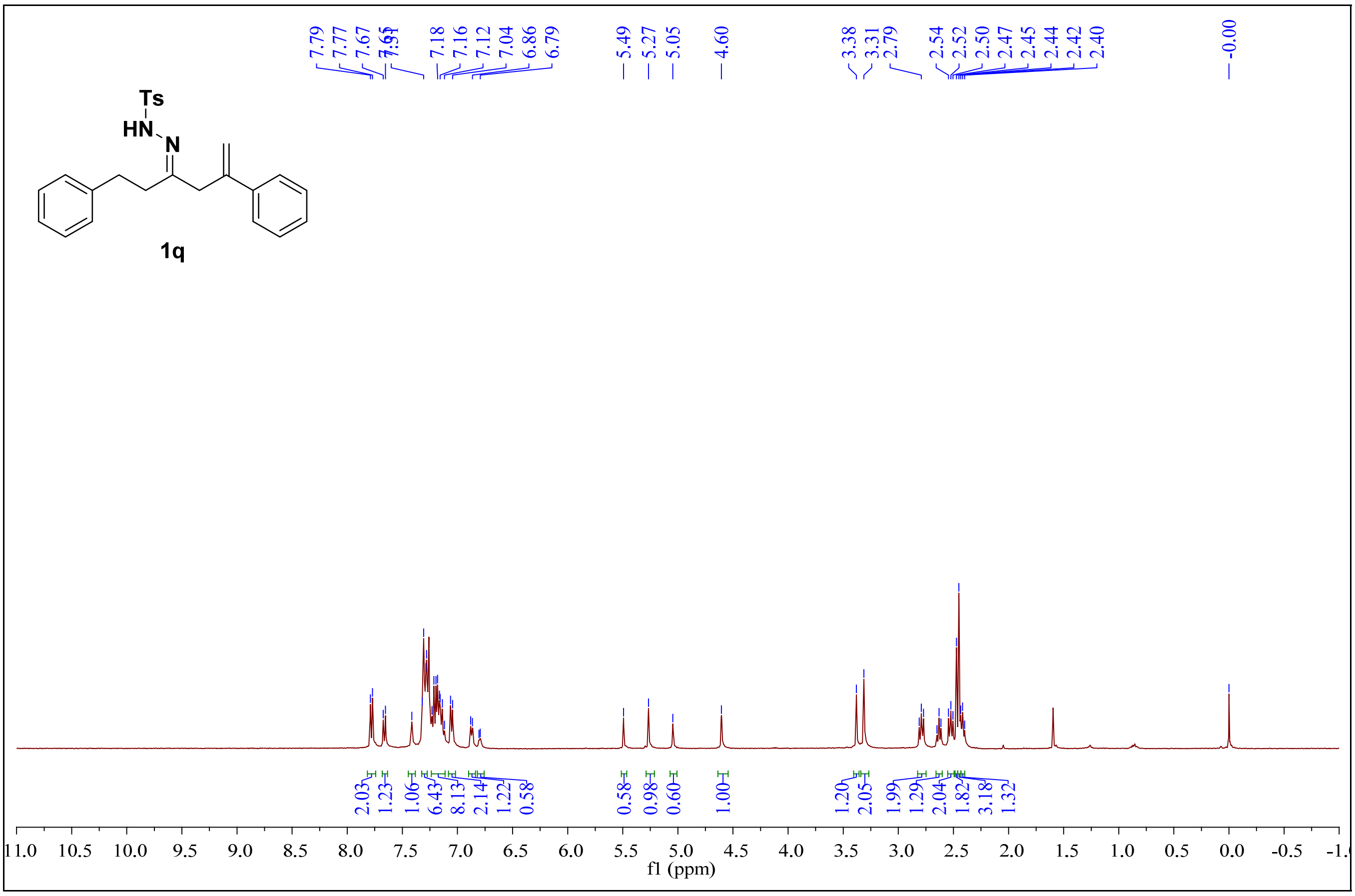

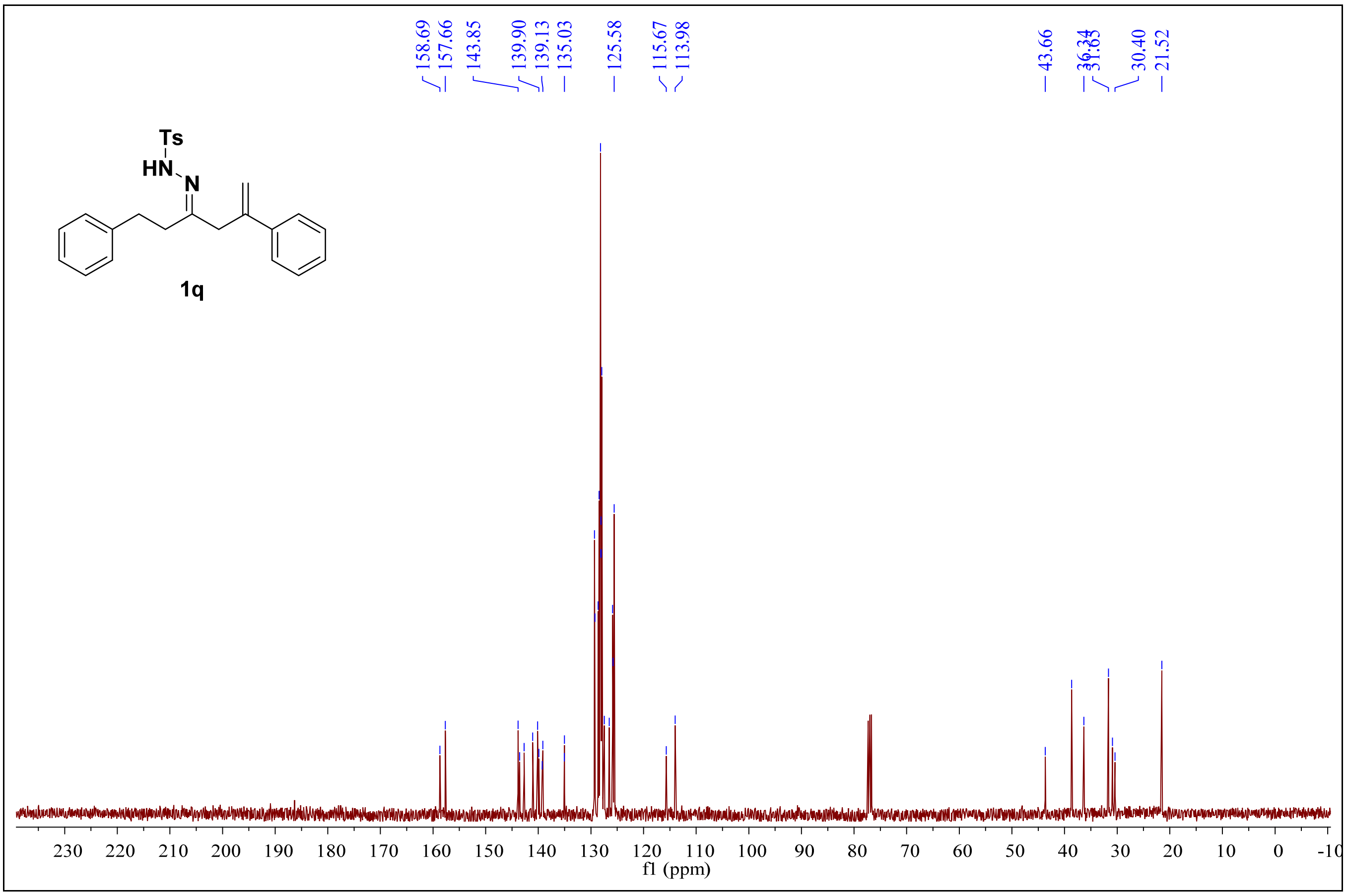

Supplementary Figure 17. ^{1}**H NMR (400 MHz, CDCl$_3$) and** ^{13}**C NMR (100 MHz, CDCl$_3$) spectra of hydrazone 1r**

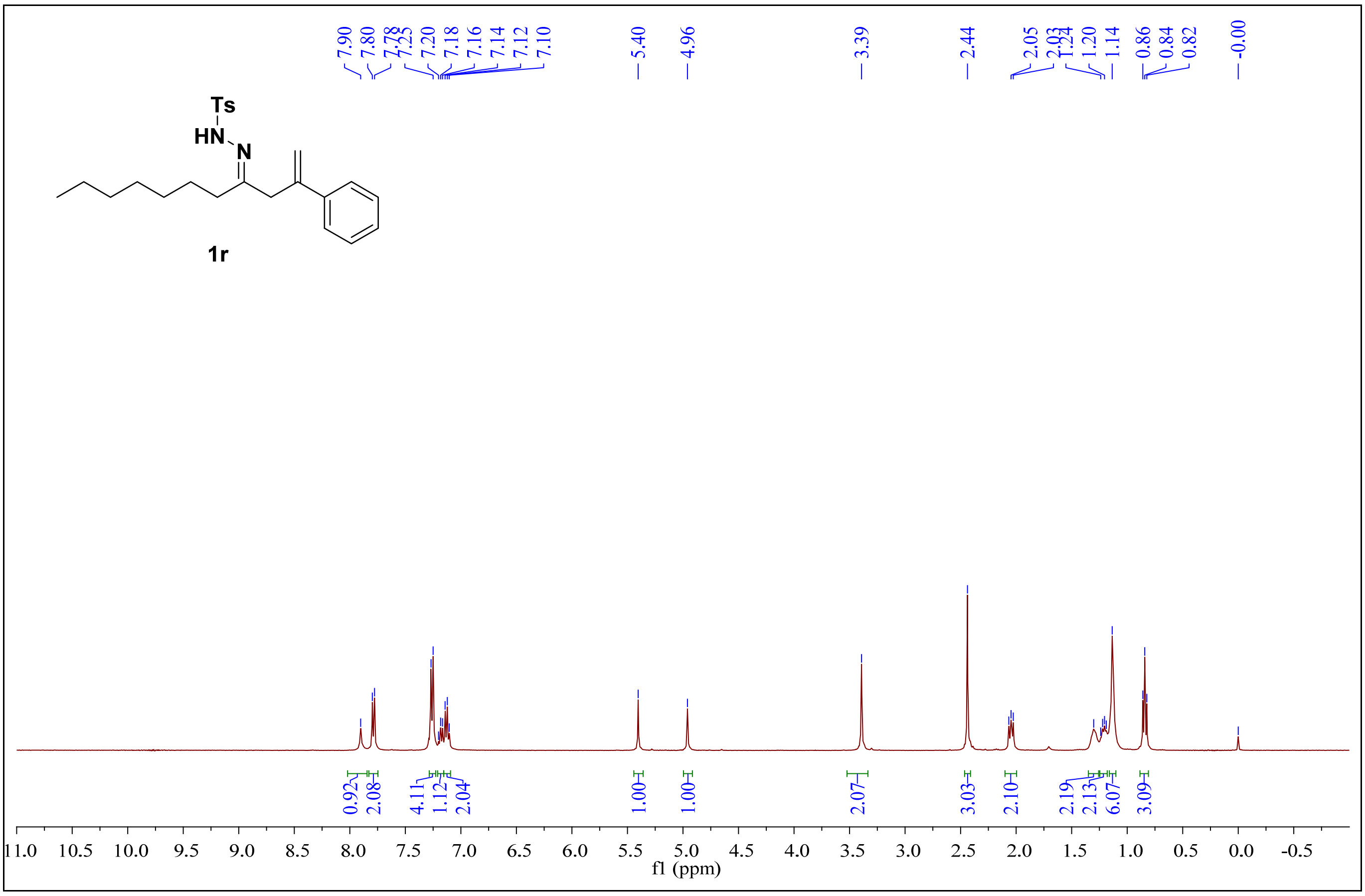

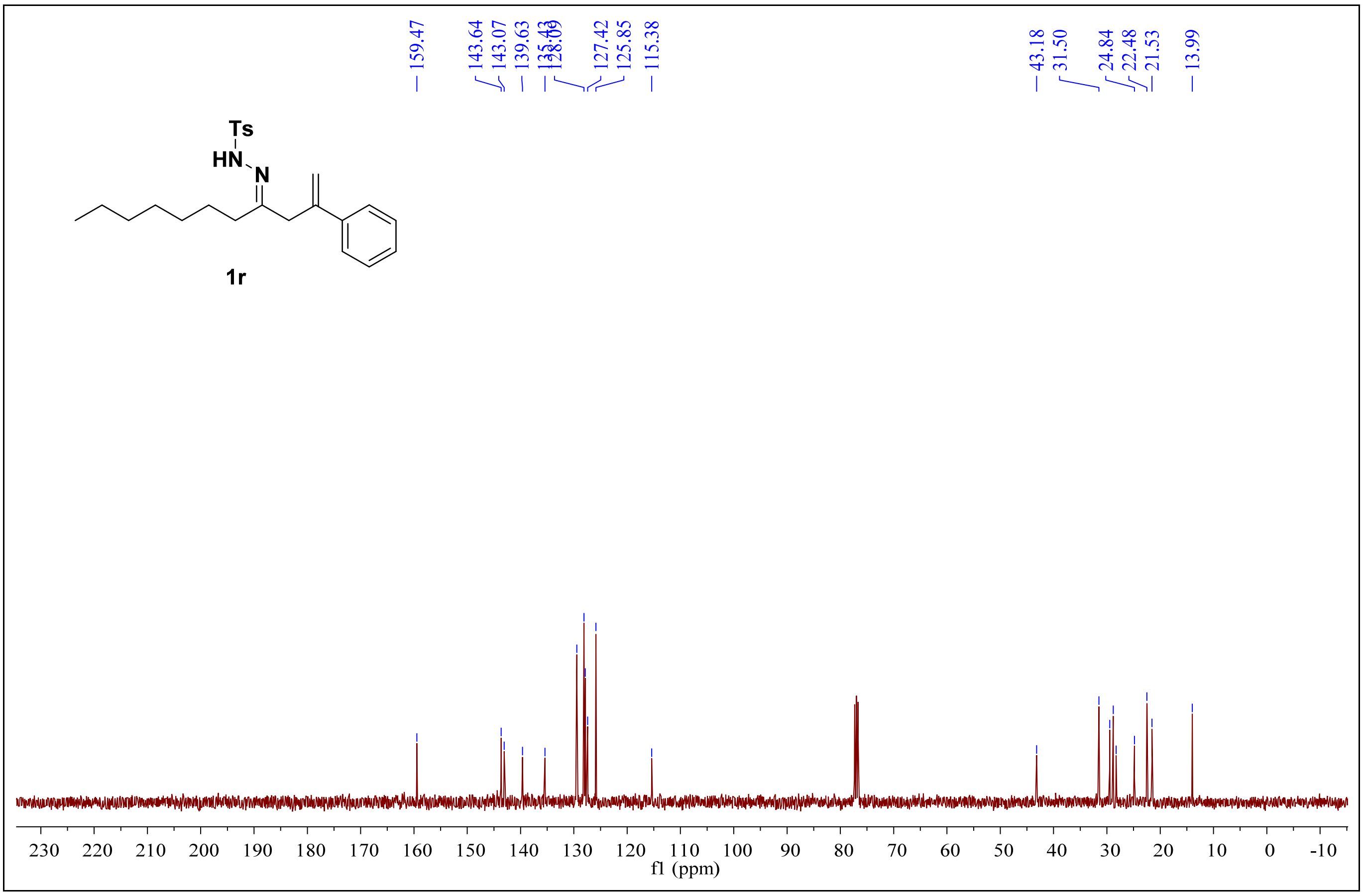

Supplementary Figure 18. ^{1}H **NMR (600 MHz,** $CDCl_3$**) and** ^{13}C **NMR (100 MHz,** $CDCl_3$**) spectra of hydrazone 1s**

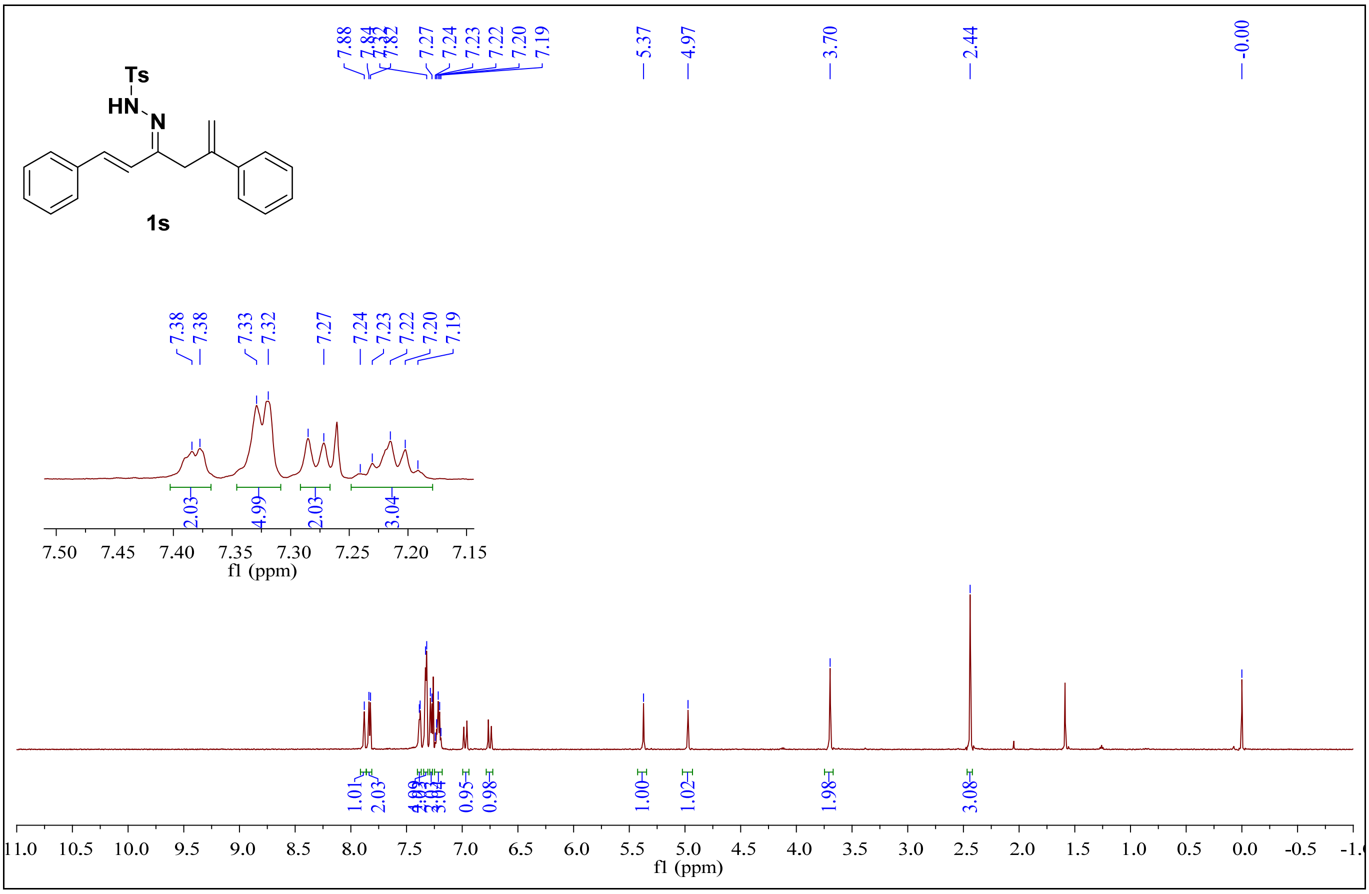

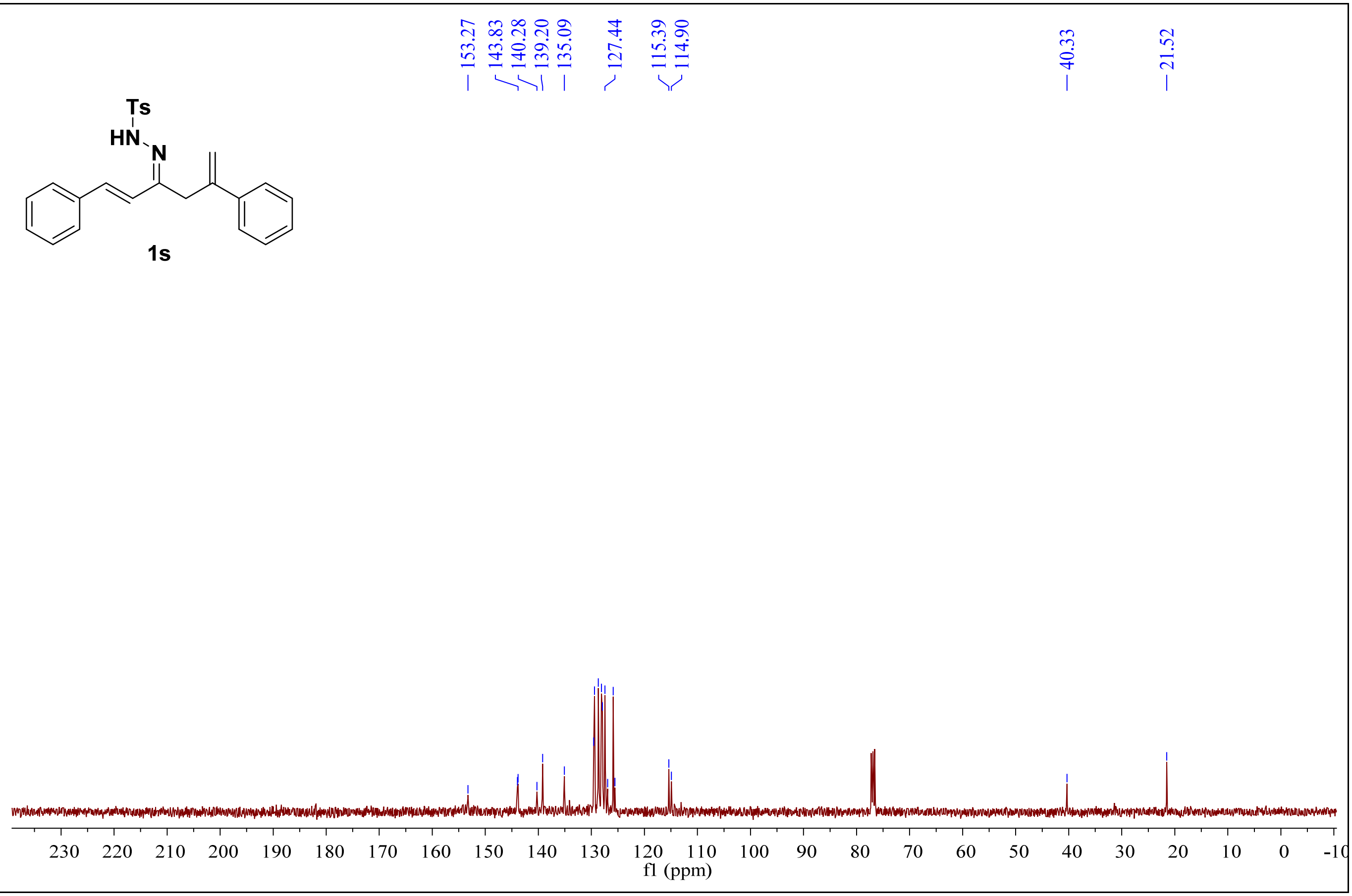

Supplementary Figure 19. **^{1}H NMR (600 MHz, $CDCl_3$) and ^{13}C NMR (100 MHz, $CDCl_3$) spectra of hydrazone 1t**

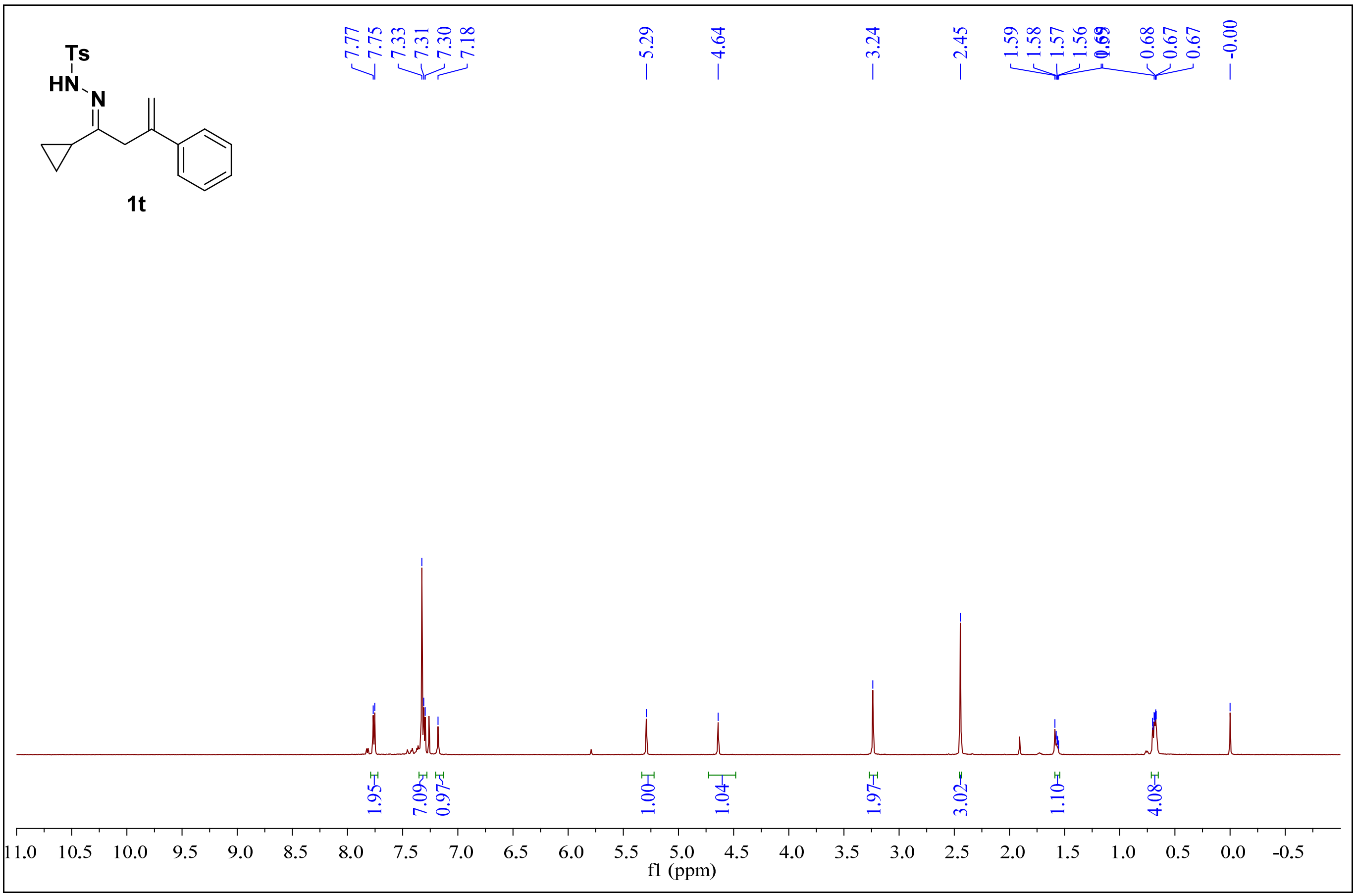

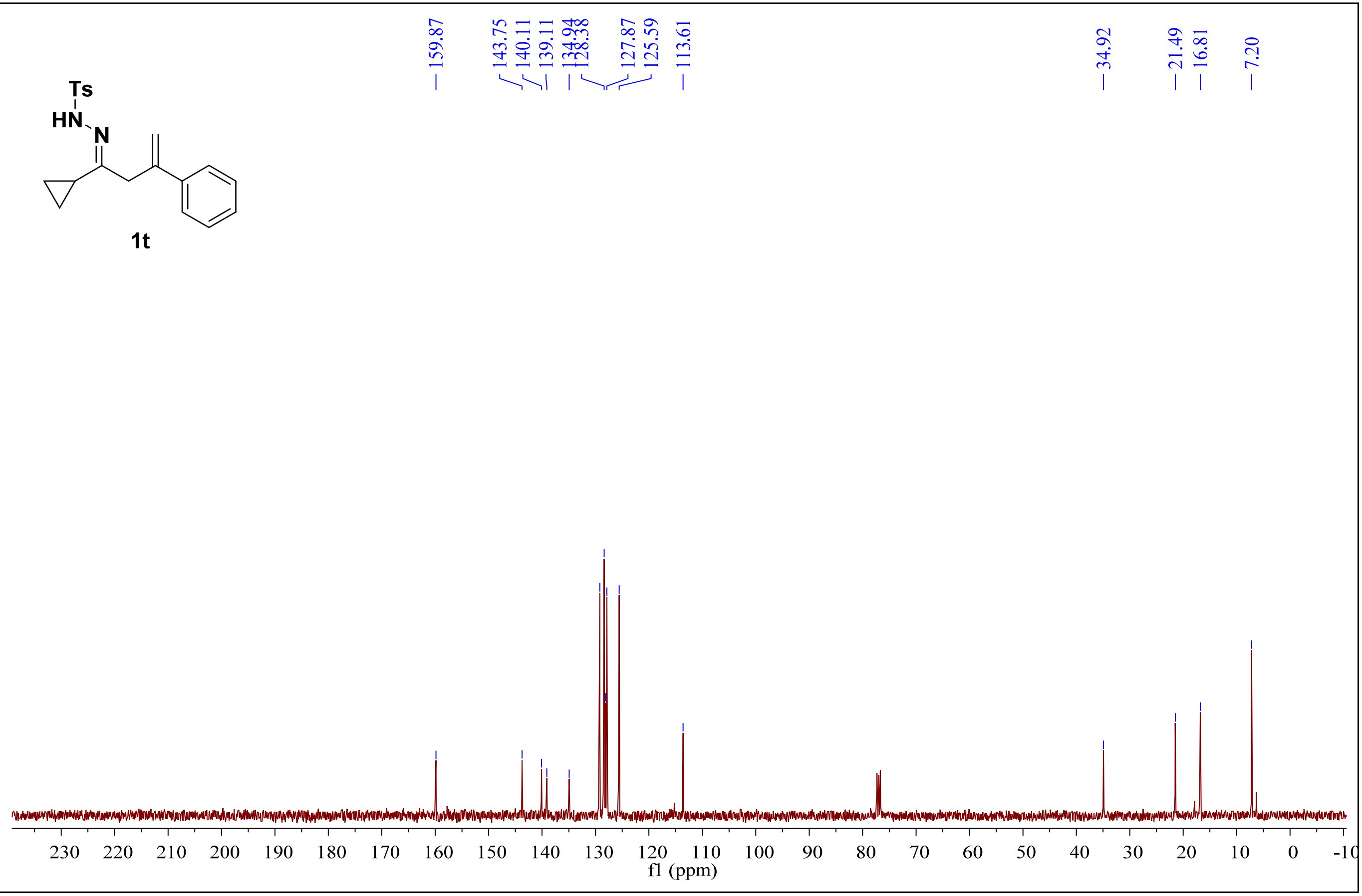

Supplementary Figure 20. ^{1}H **NMR (400 MHz, DMSO-d**^{6}**) and** ^{13}C **NMR (100 MHz,** $CDCl_3$**) spectra of hydrazone 1u**

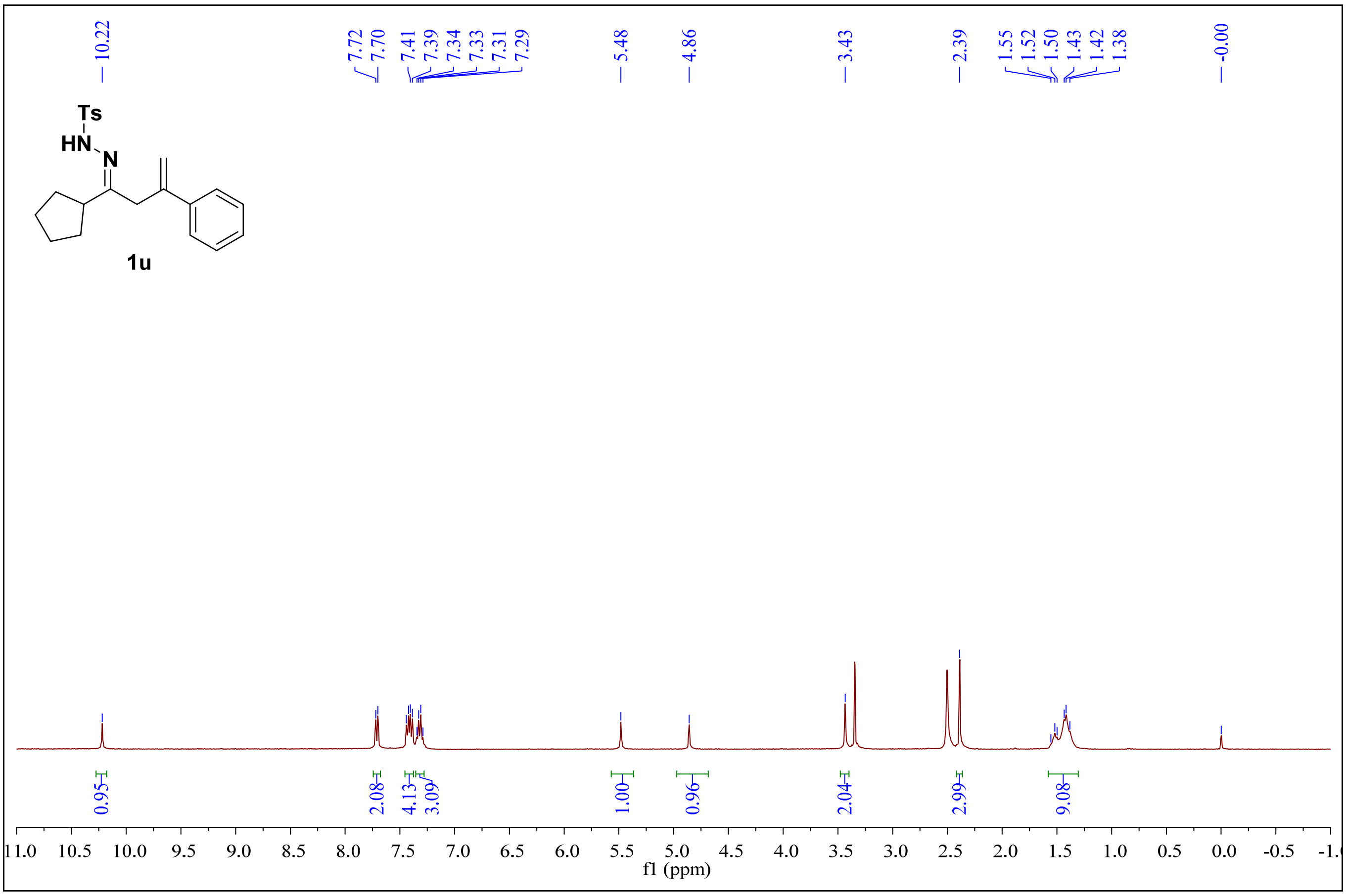

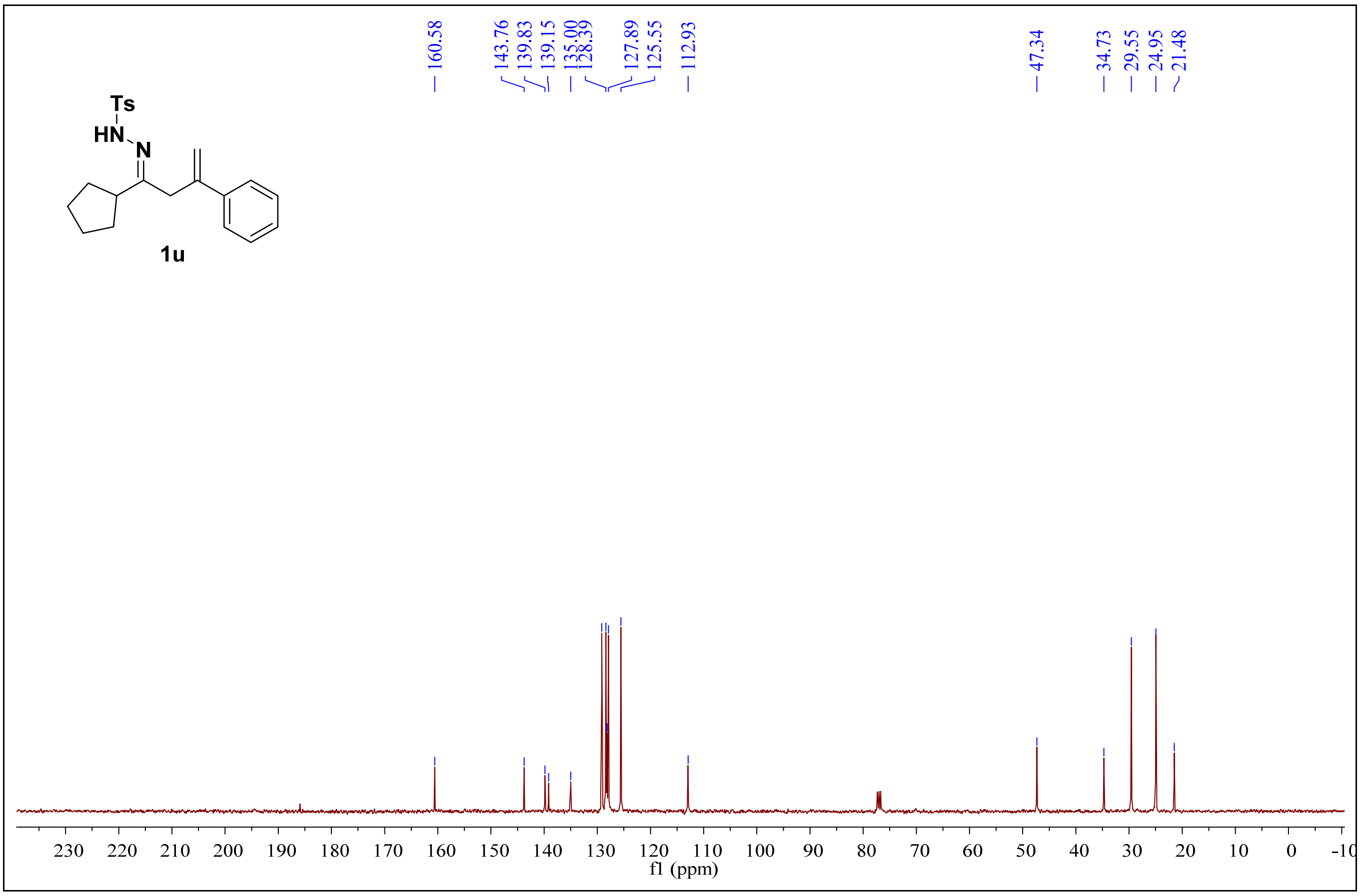

Supplementary Figure 21. **^{1}H NMR (400 MHz, $CDCl_3$) and ^{13}C NMR (100 MHz, $CDCl_3$) spectra of hydrazone 1v**

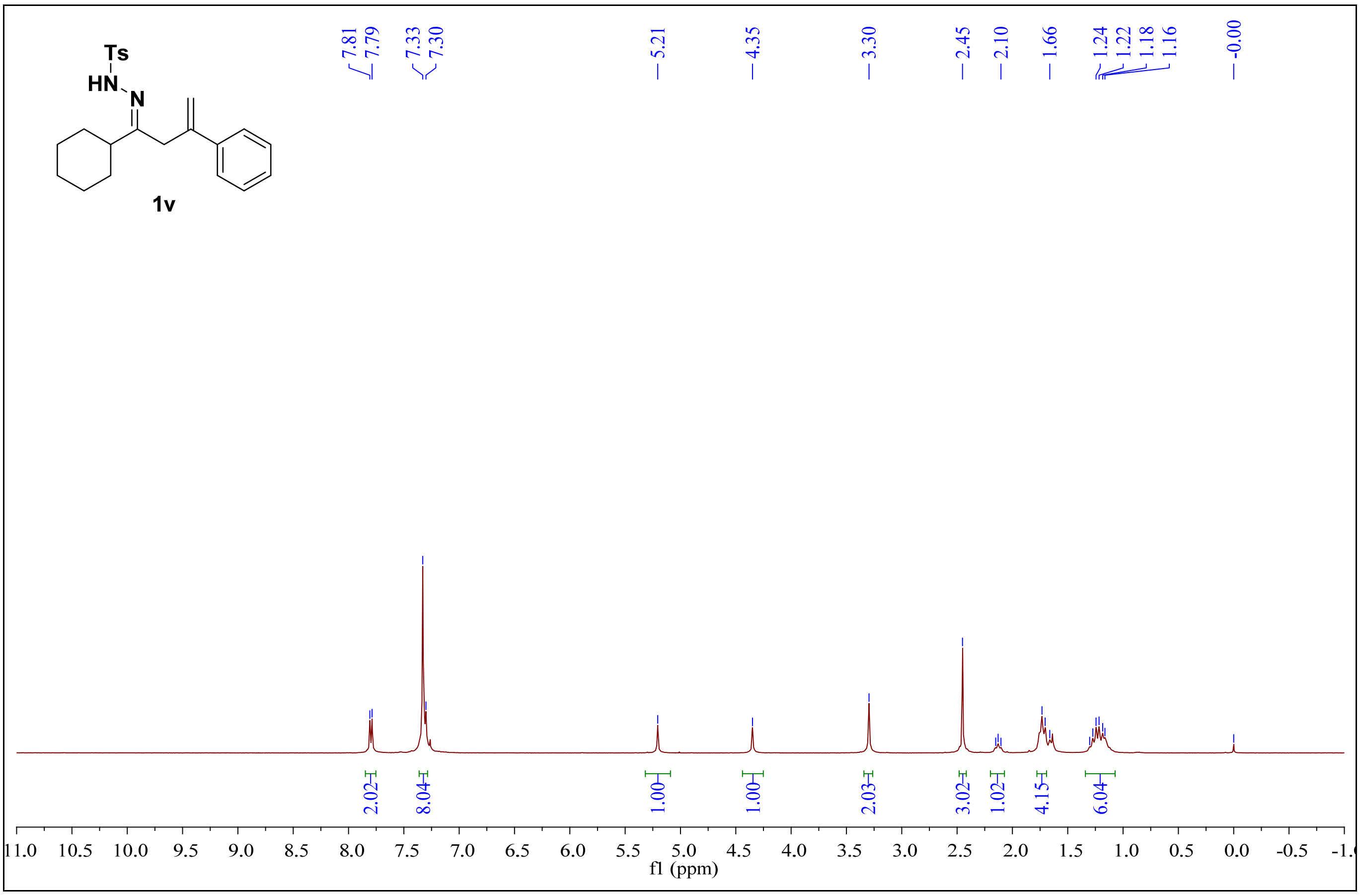

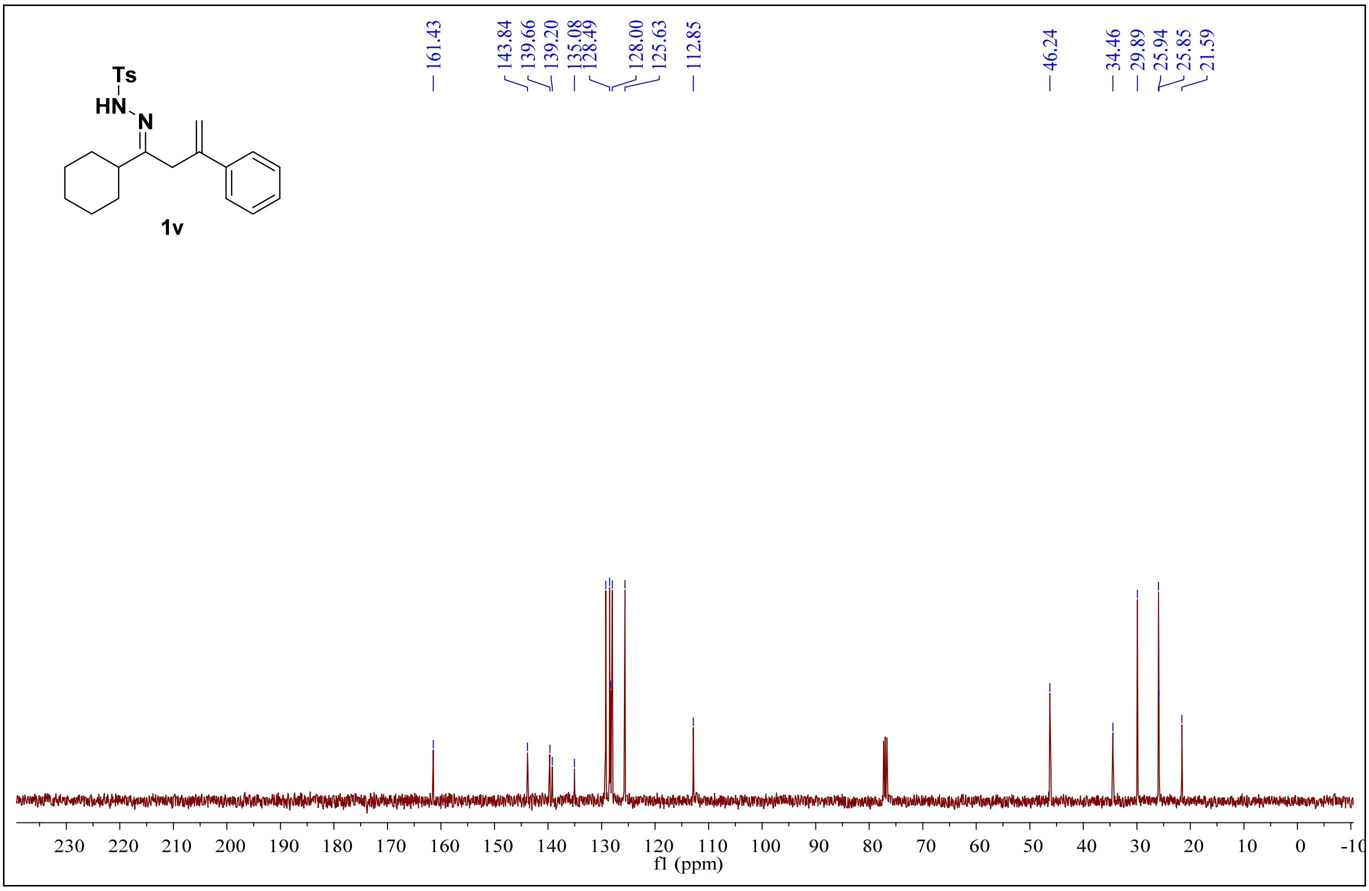

Supplementary Figure 22. ^{1}H NMR (600 MHz, $CDCl_3$) and ^{13}C NMR (100 MHz, $CDCl_3$) spectra of hydrazone 5a

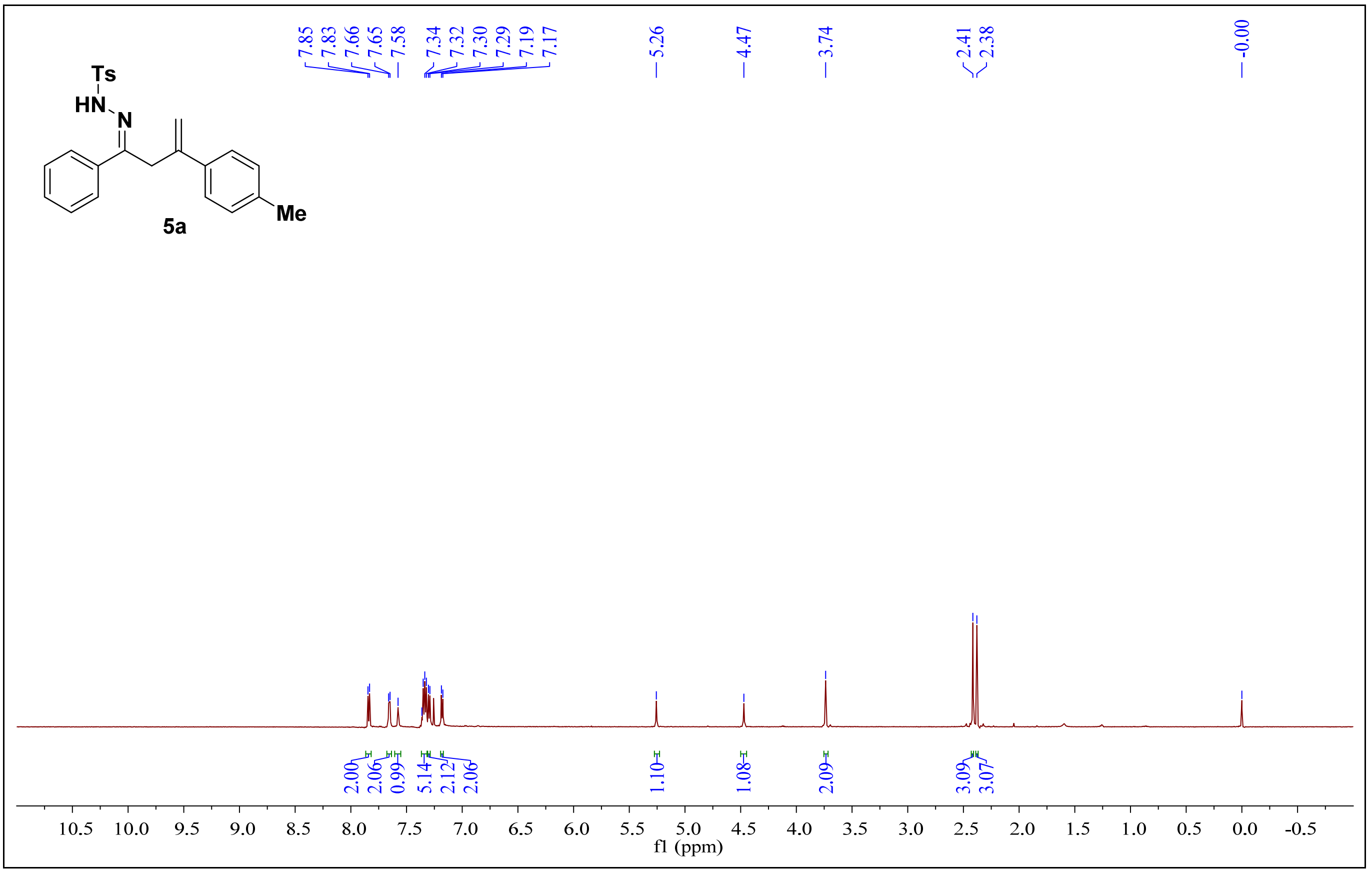

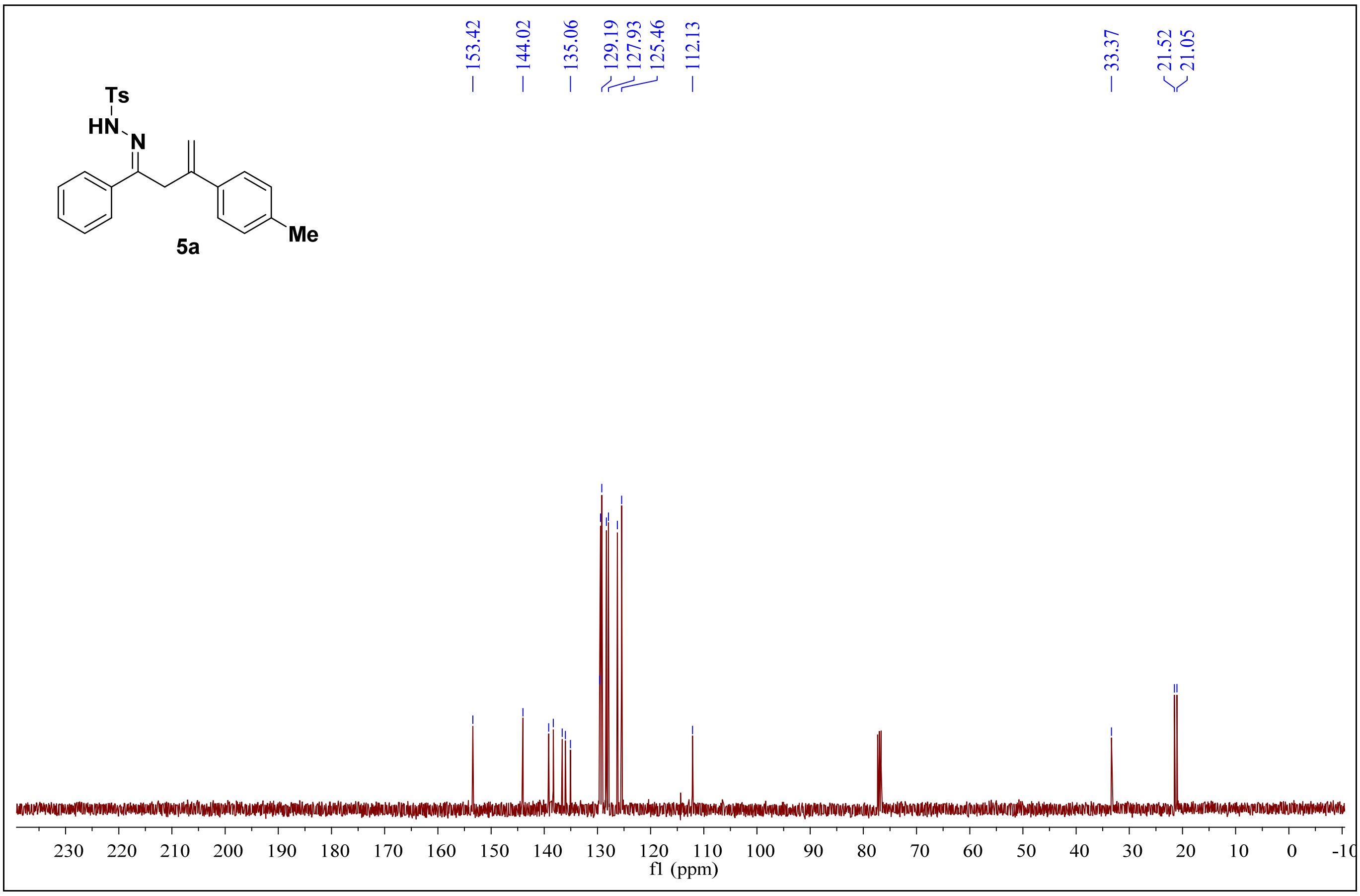

Supplementary Figure 23. ^{1}H **NMR (400 MHz, DMSO-d**^{6}**) and** ^{13}C **NMR (100 MHz,** $CDCl_3$**) spectra of hydrazone 5b**

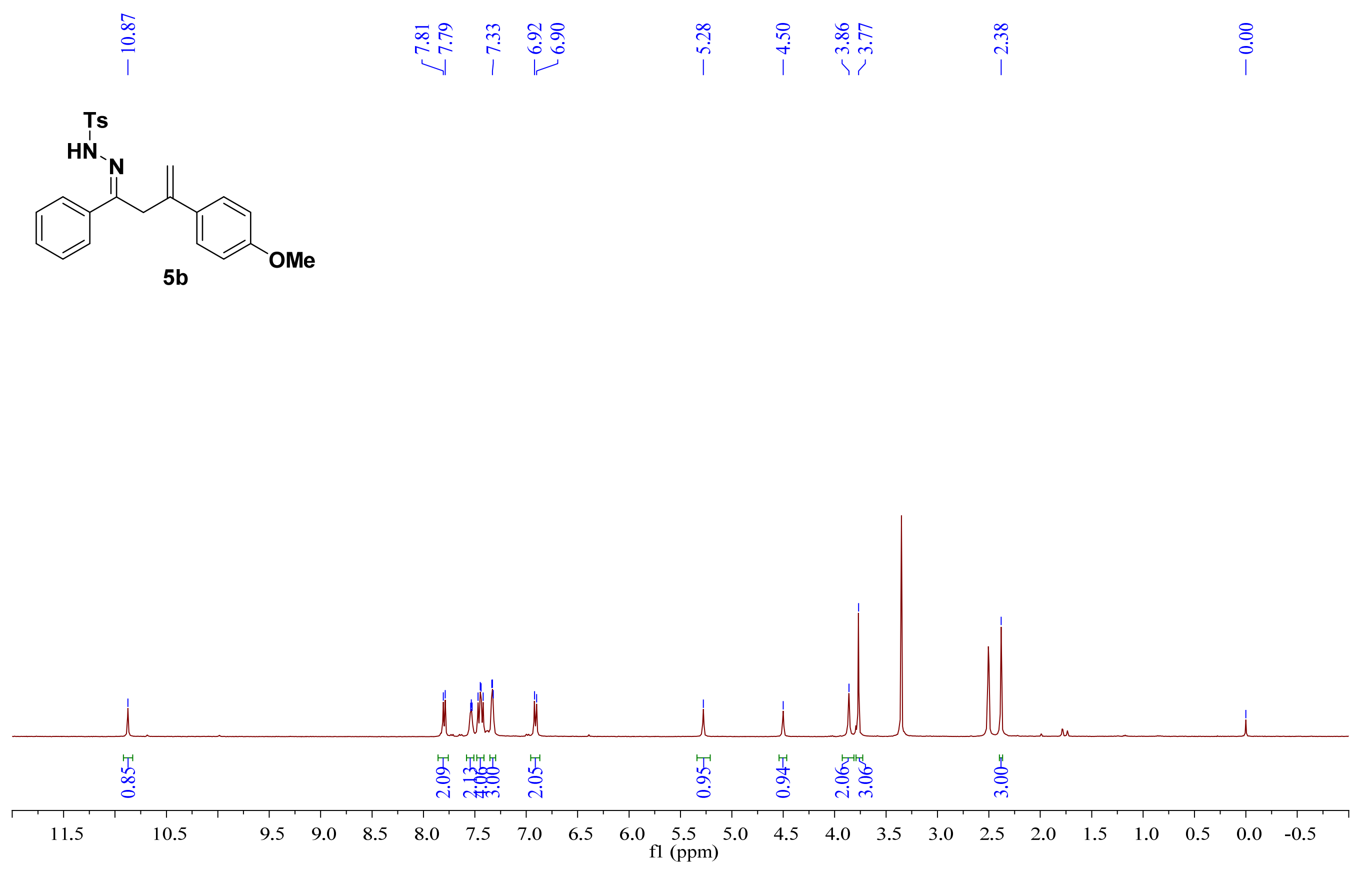

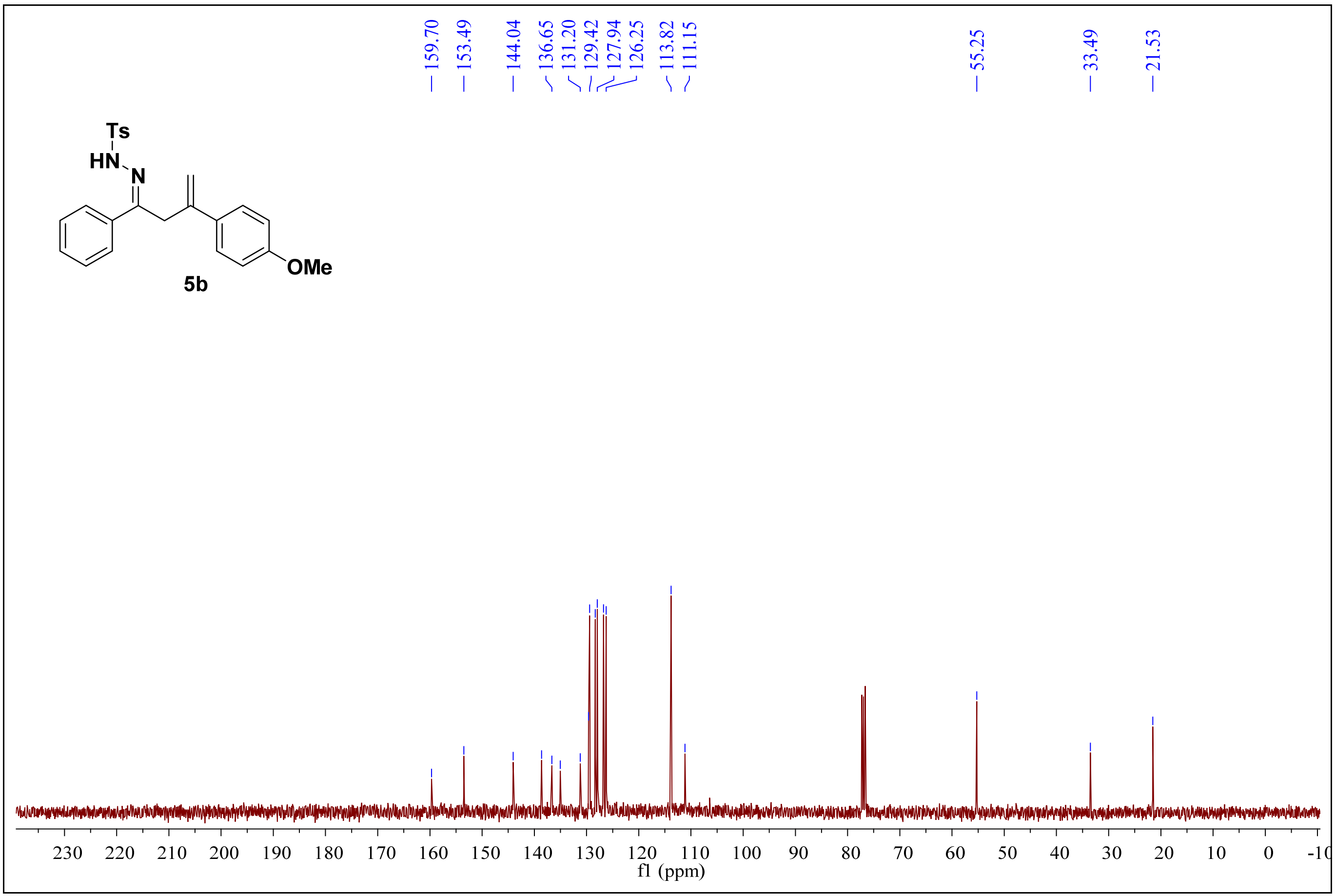

Supplementary Figure 24. ^{1}**H NMR (400 MHz, CDCl$_3$) and** ^{13}**C NMR (100 MHz, CDCl$_3$) spectra of hydrazone 5c**

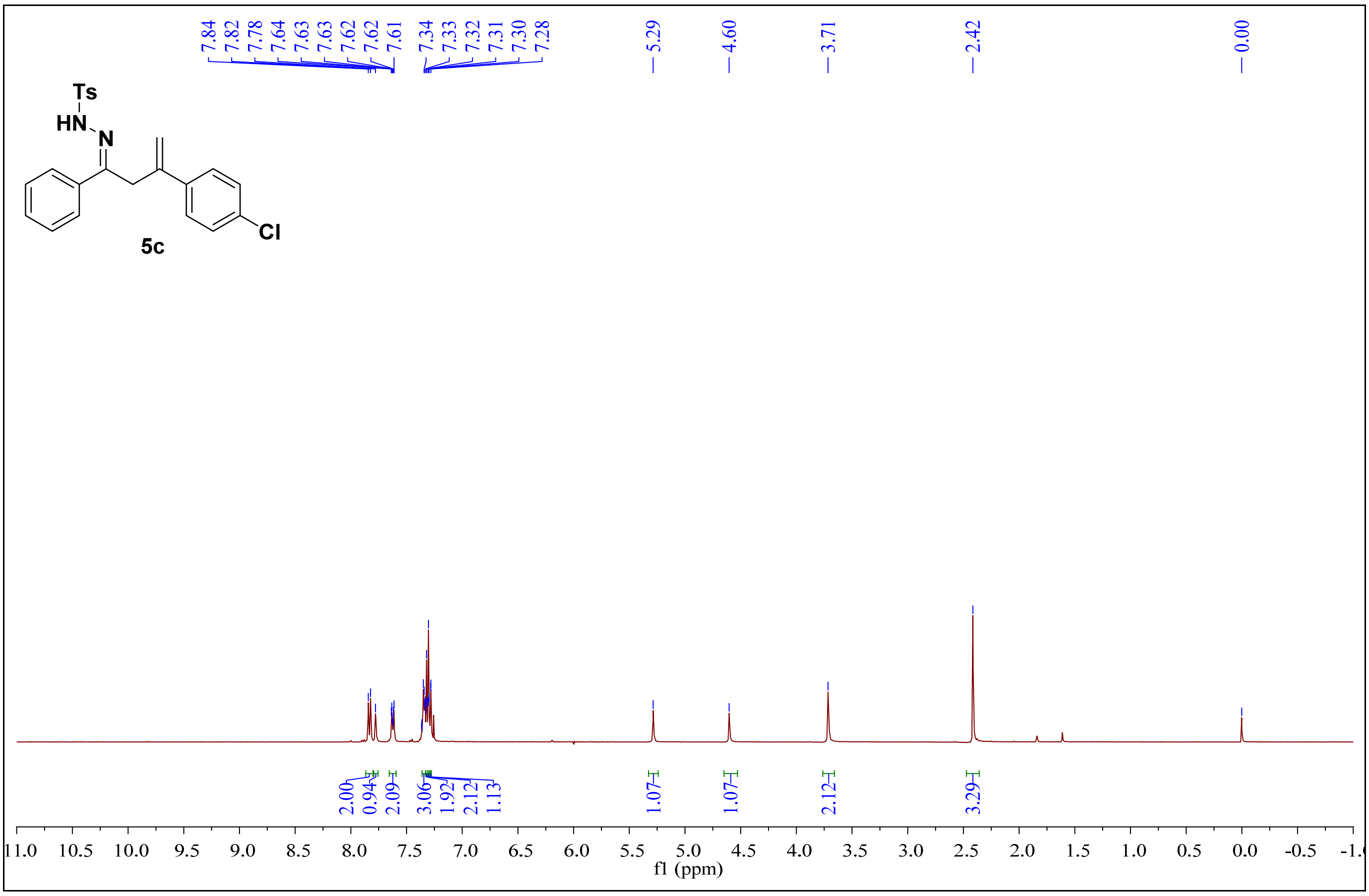

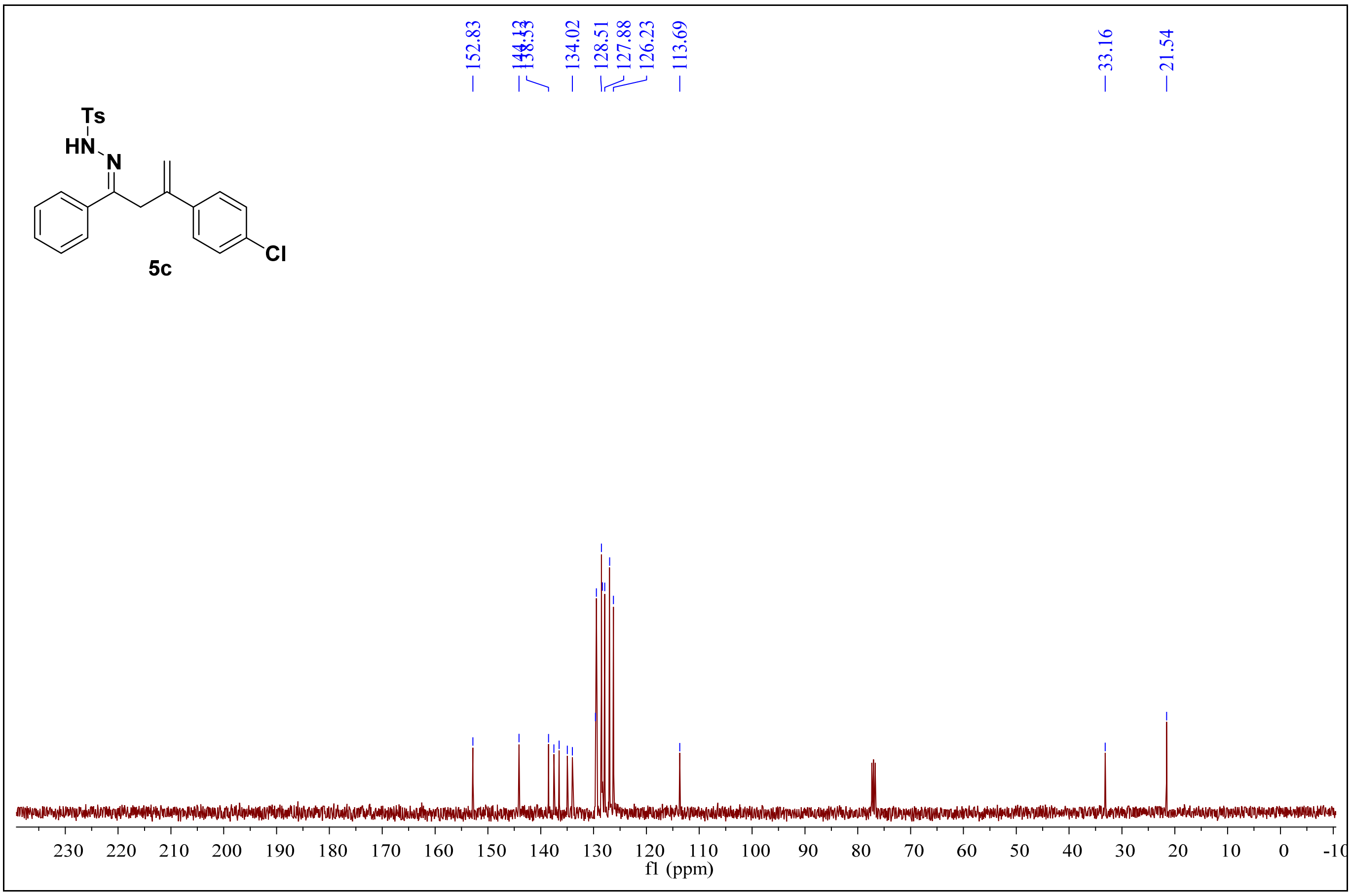

Supplementary Figure 25. ^{1}H NMR (400 MHz, $CDCl_3$) and ^{13}C NMR (100 MHz, $CDCl_3$) spectra of hydrazone 5d

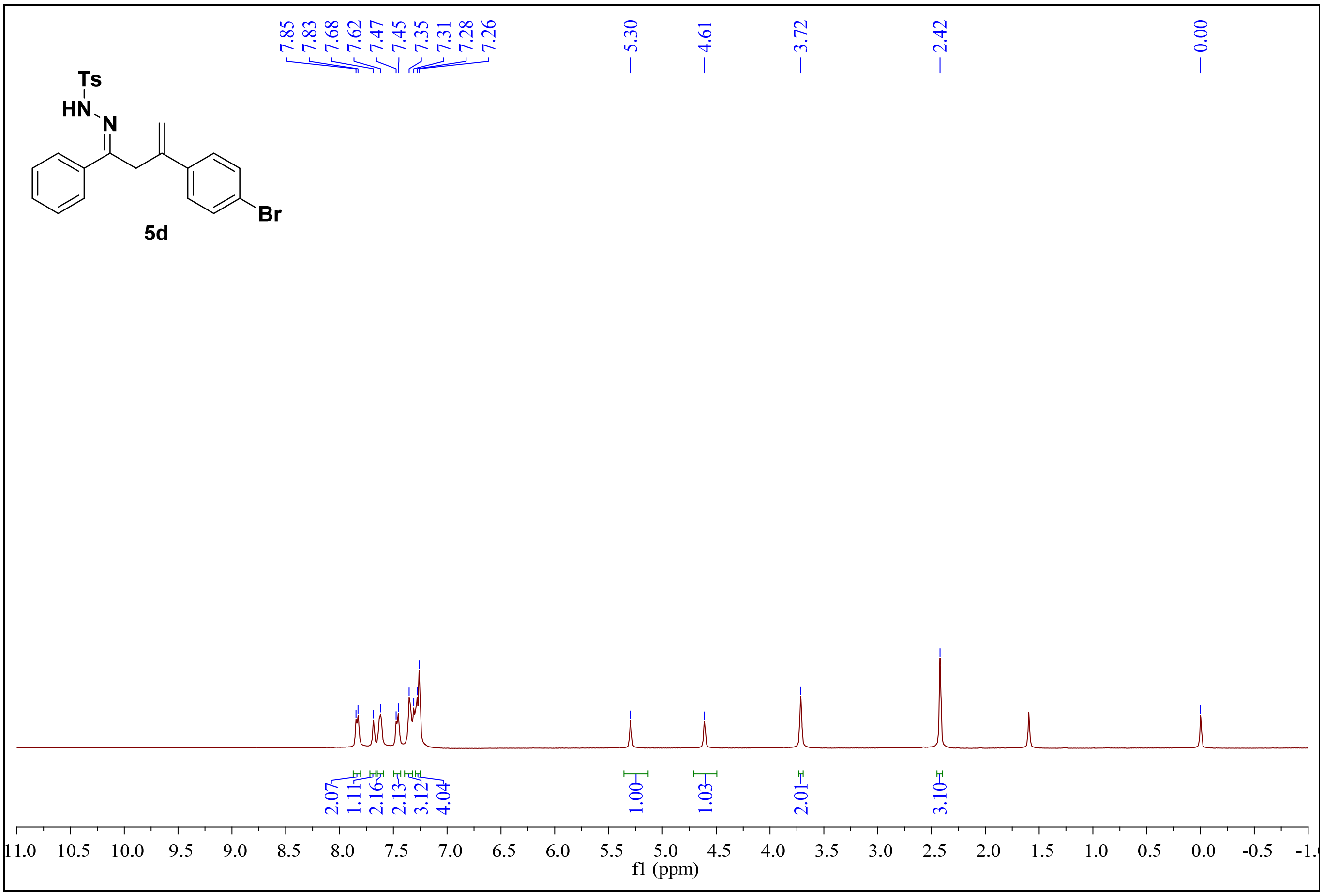

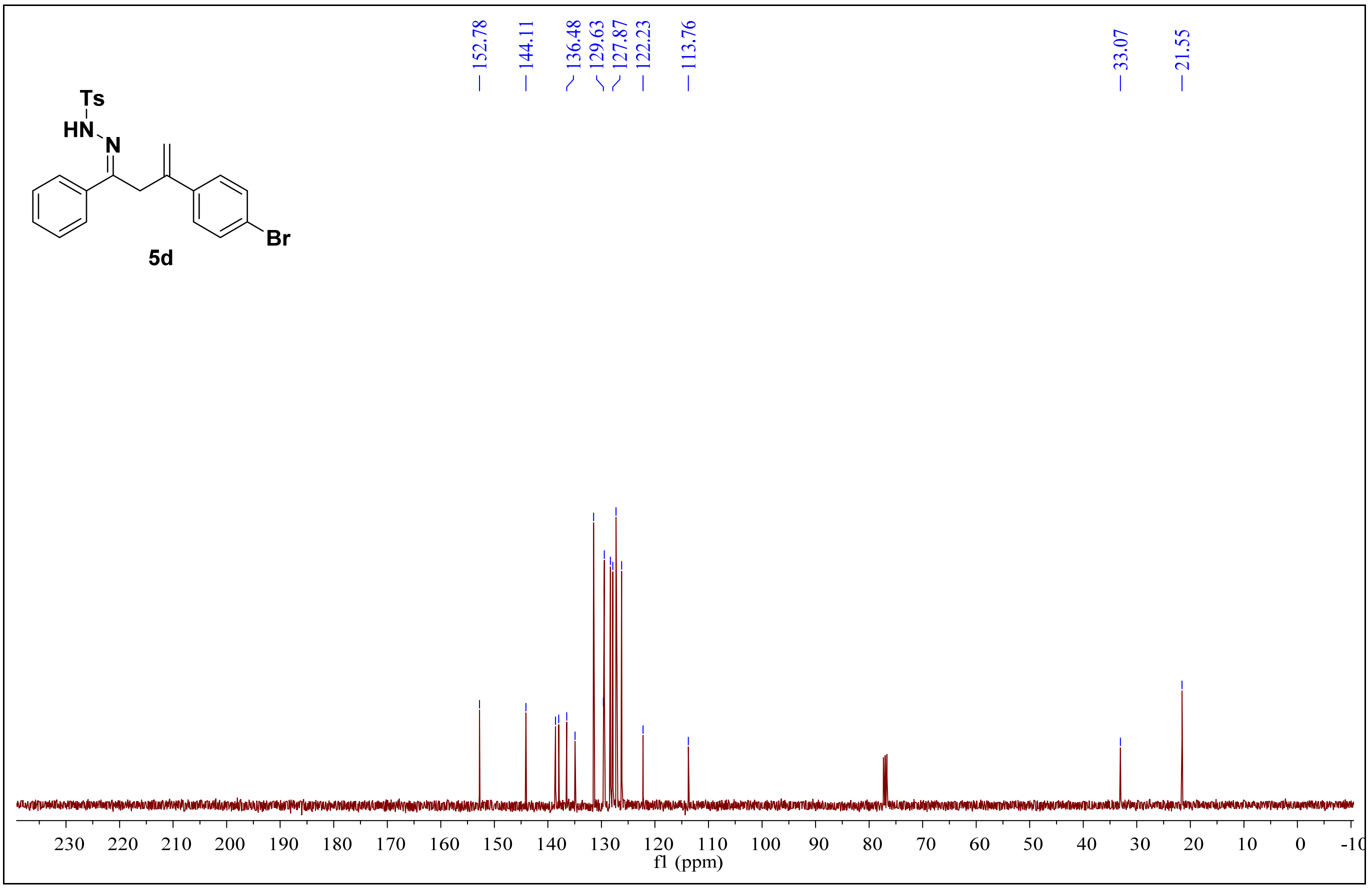

Supplementary Figure 26. ¹H NMR (600 MHz, $CDCl_3$), ¹³C NMR (100 MHz, $CDCl_3$) and ¹⁹F (376 MHz, $CDCl_3$) spectra of hydrazone 5e

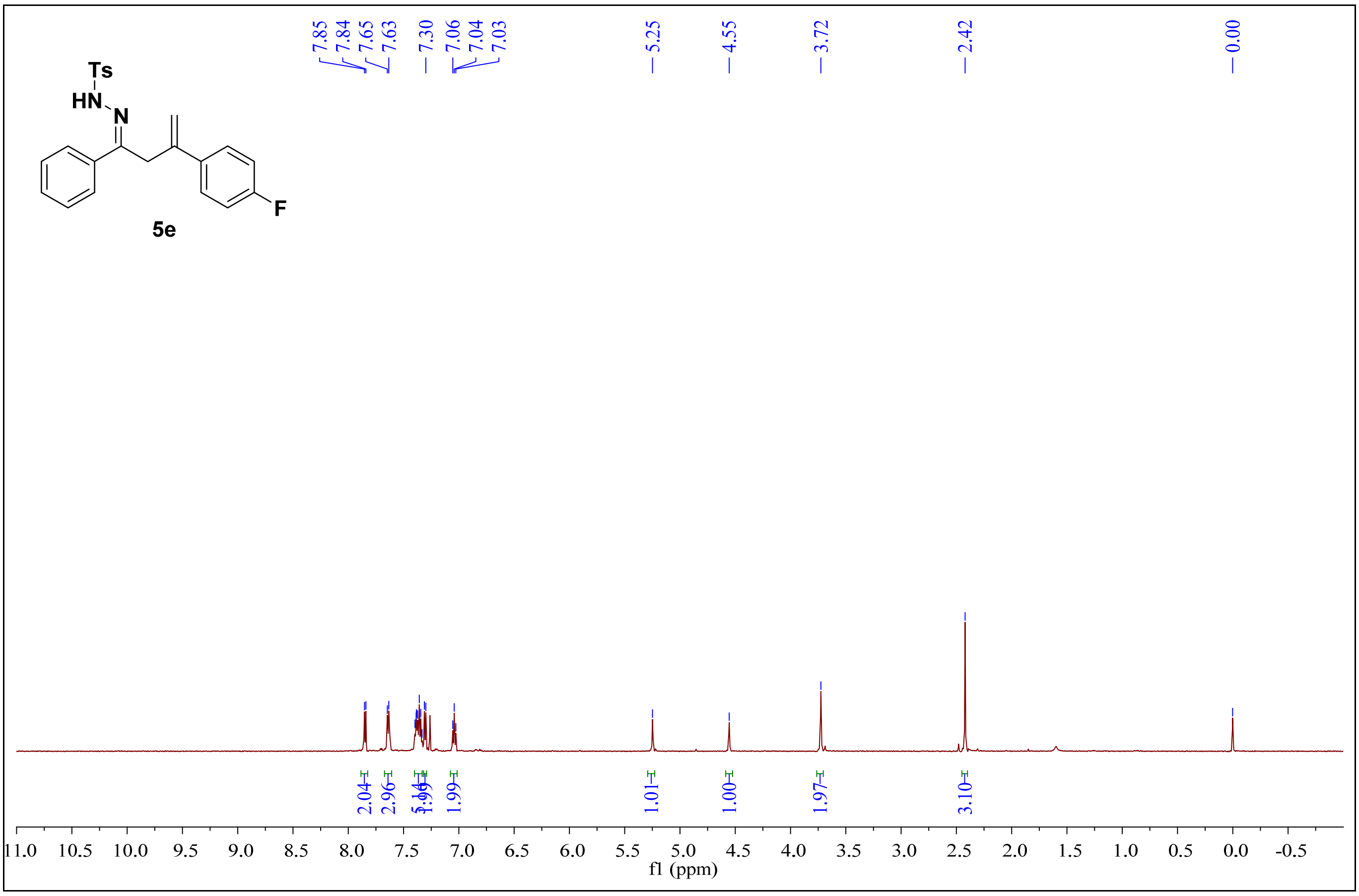

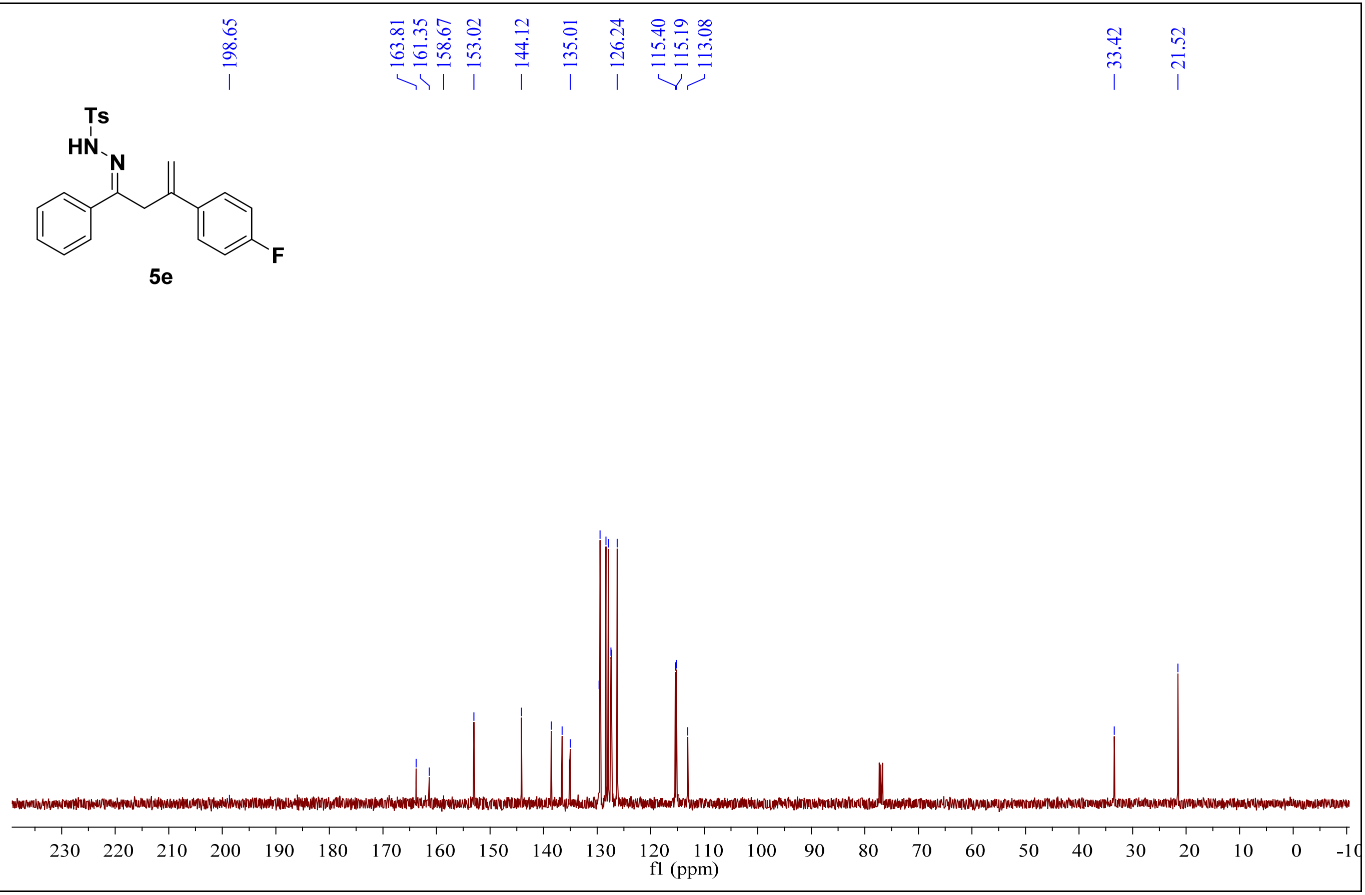

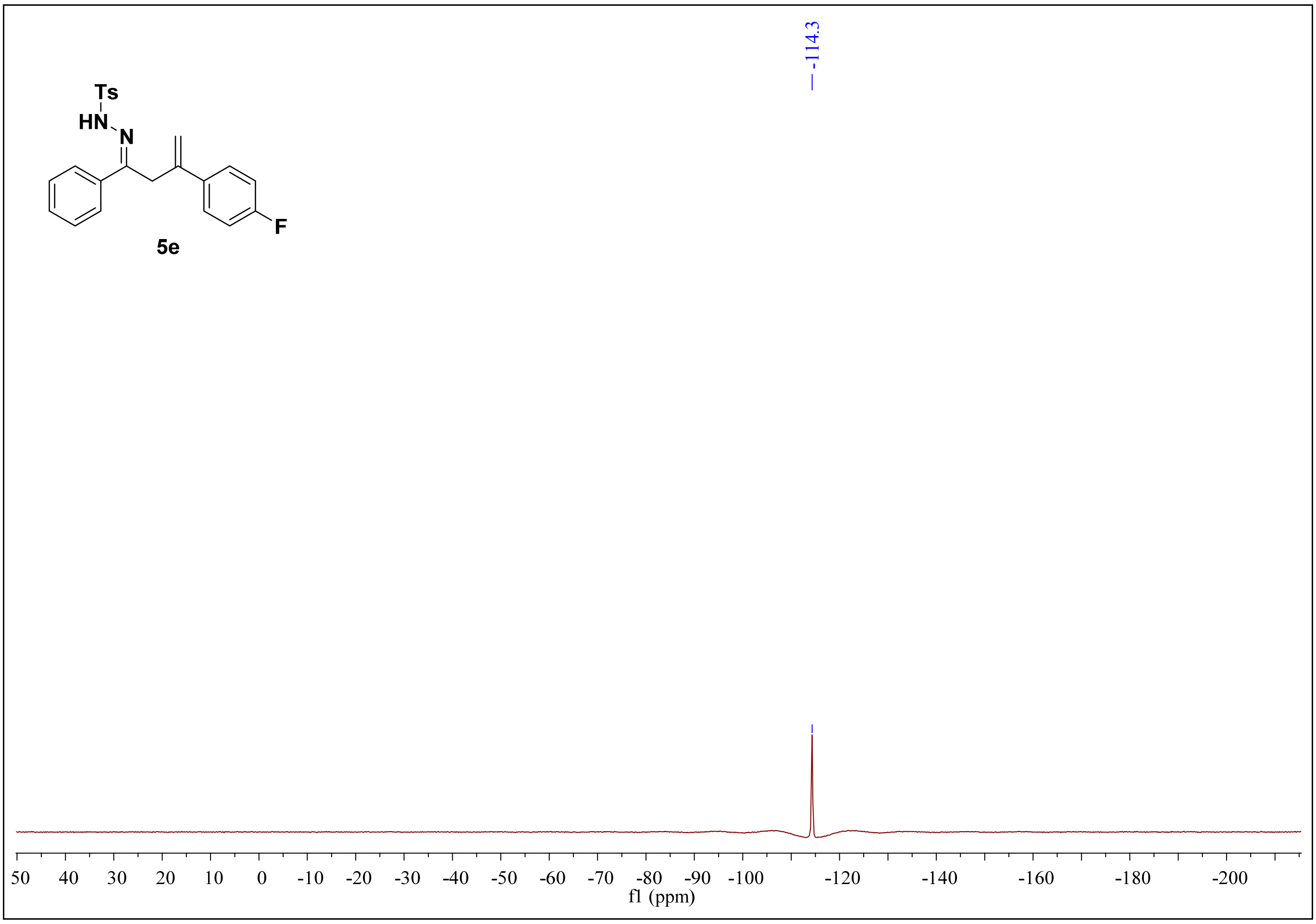
Ts
HN
N
F
5e
-114.3
50 40 30 20 10 0 -10 -20 -30 -40 -50 -60 -70 -80 -90 -100 -120 -140 -160 -180 -200
f1 (ppm)

Supplementary Figure 27. ^{1}H NMR (600 MHz, $CDCl_3$) and ^{13}C NMR (100 MHz, $CDCl_3$) spectra of hydrazone **5f**

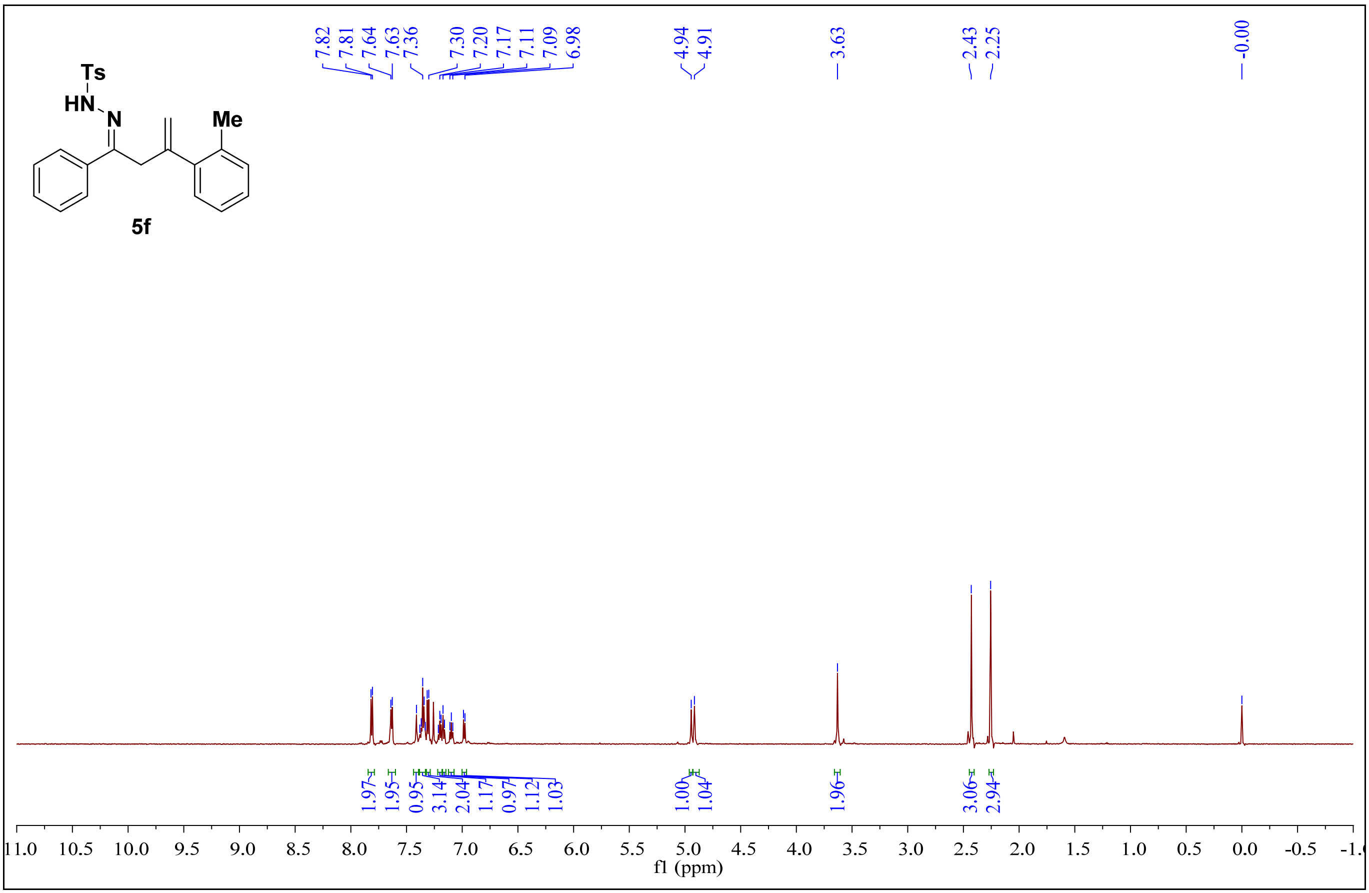

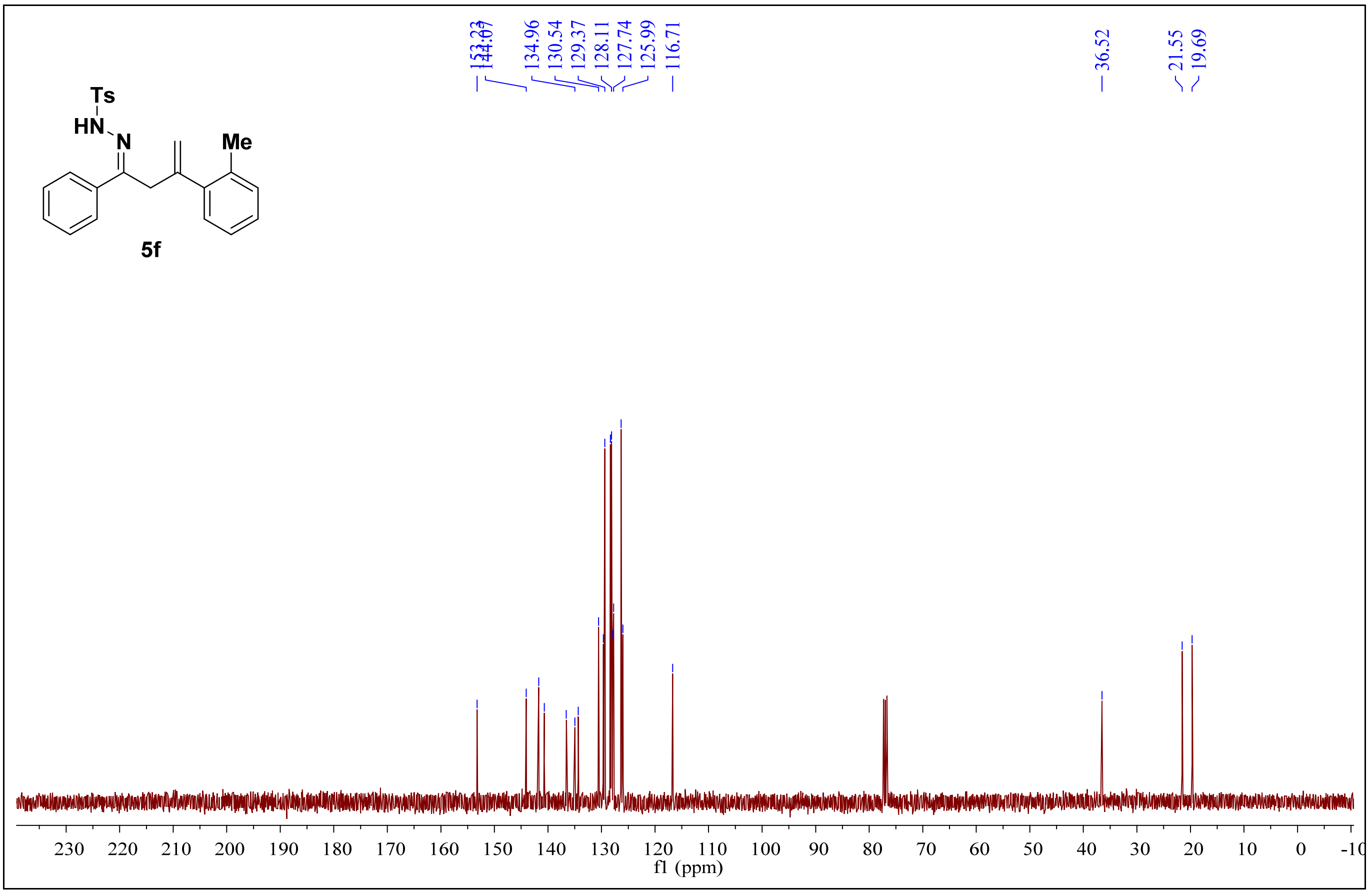

Supplementary Figure 28. ^{1}**H NMR (600 MHz, CDCl$_3$) and** ^{13}**C NMR (100 MHz, CDCl$_3$) spectra of hydrazone 5g (major : minor = 10:1)**

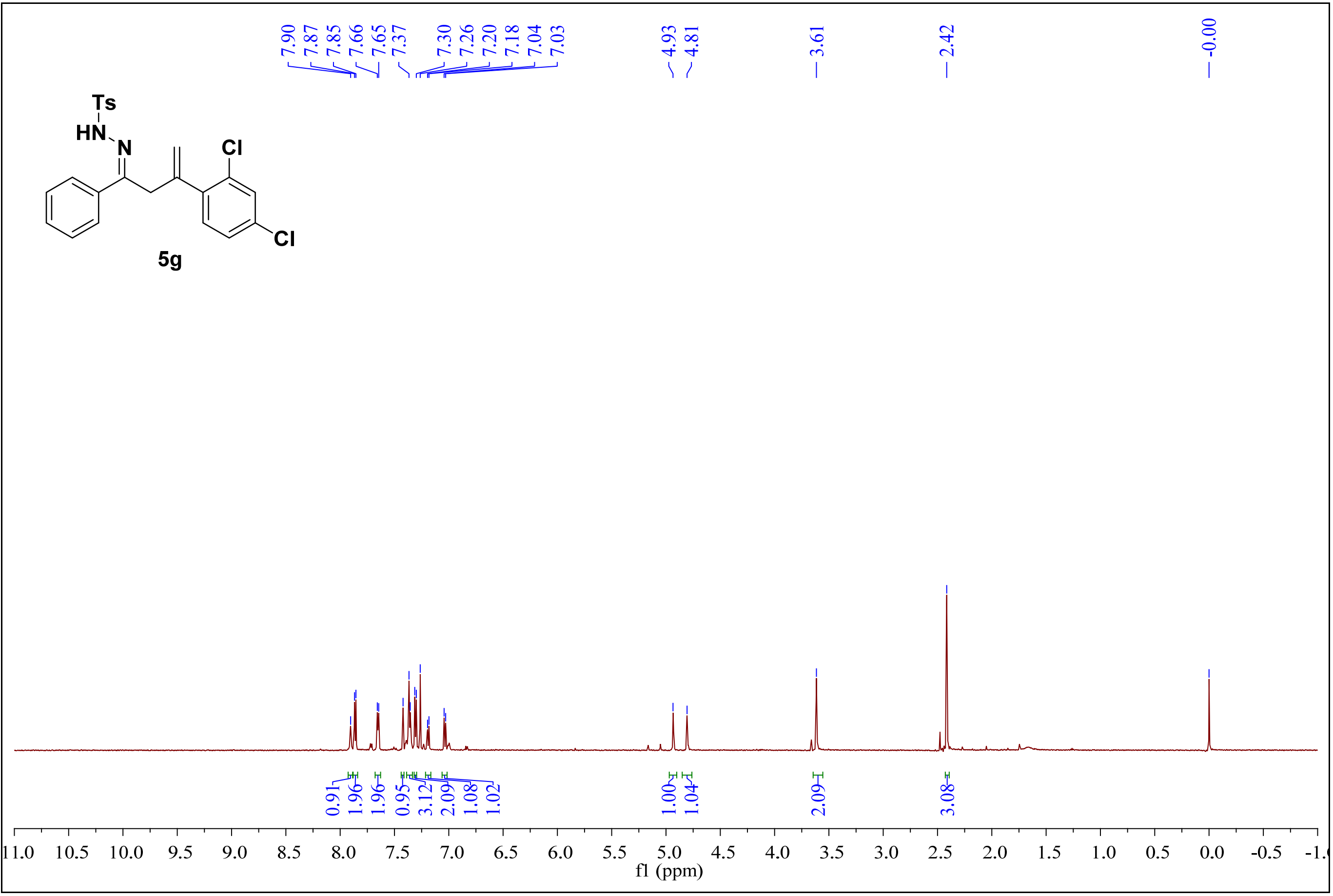

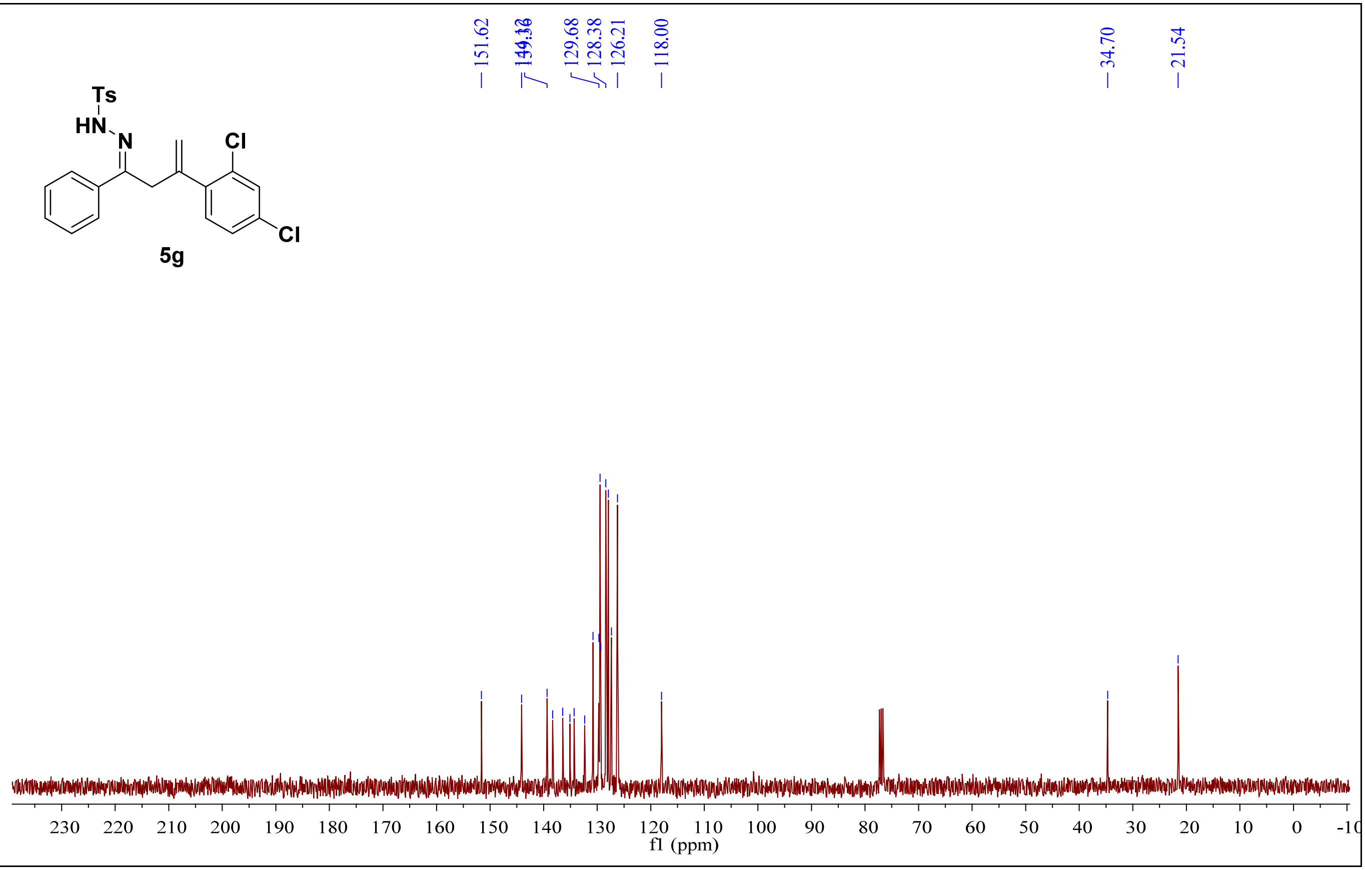

Supplementary Figure 29. ^{1}H NMR (400 MHz, $CDCl_3$) and ^{13}C NMR (100 MHz, $CDCl_3$) spectra of cyclic product **2b**

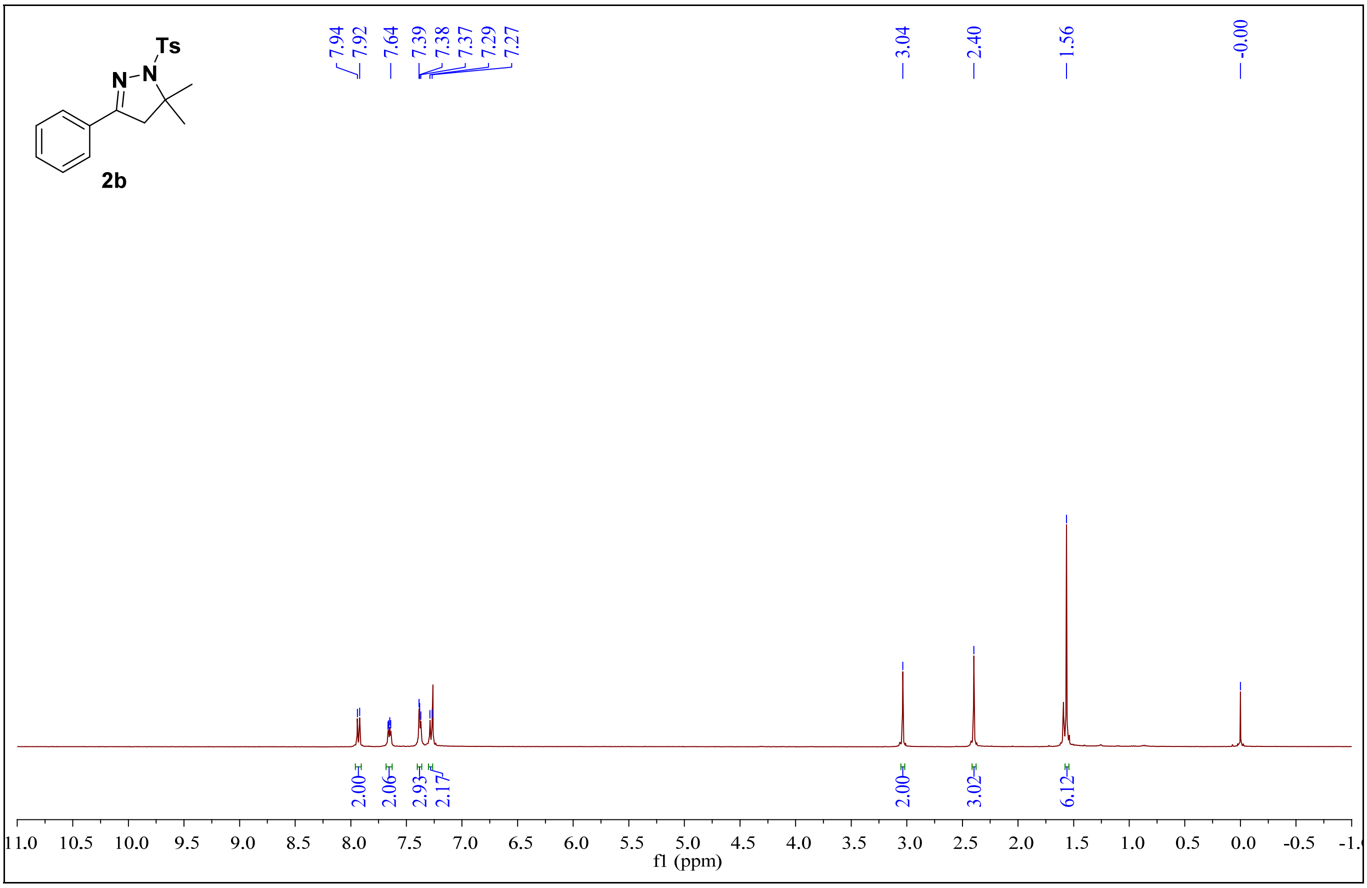

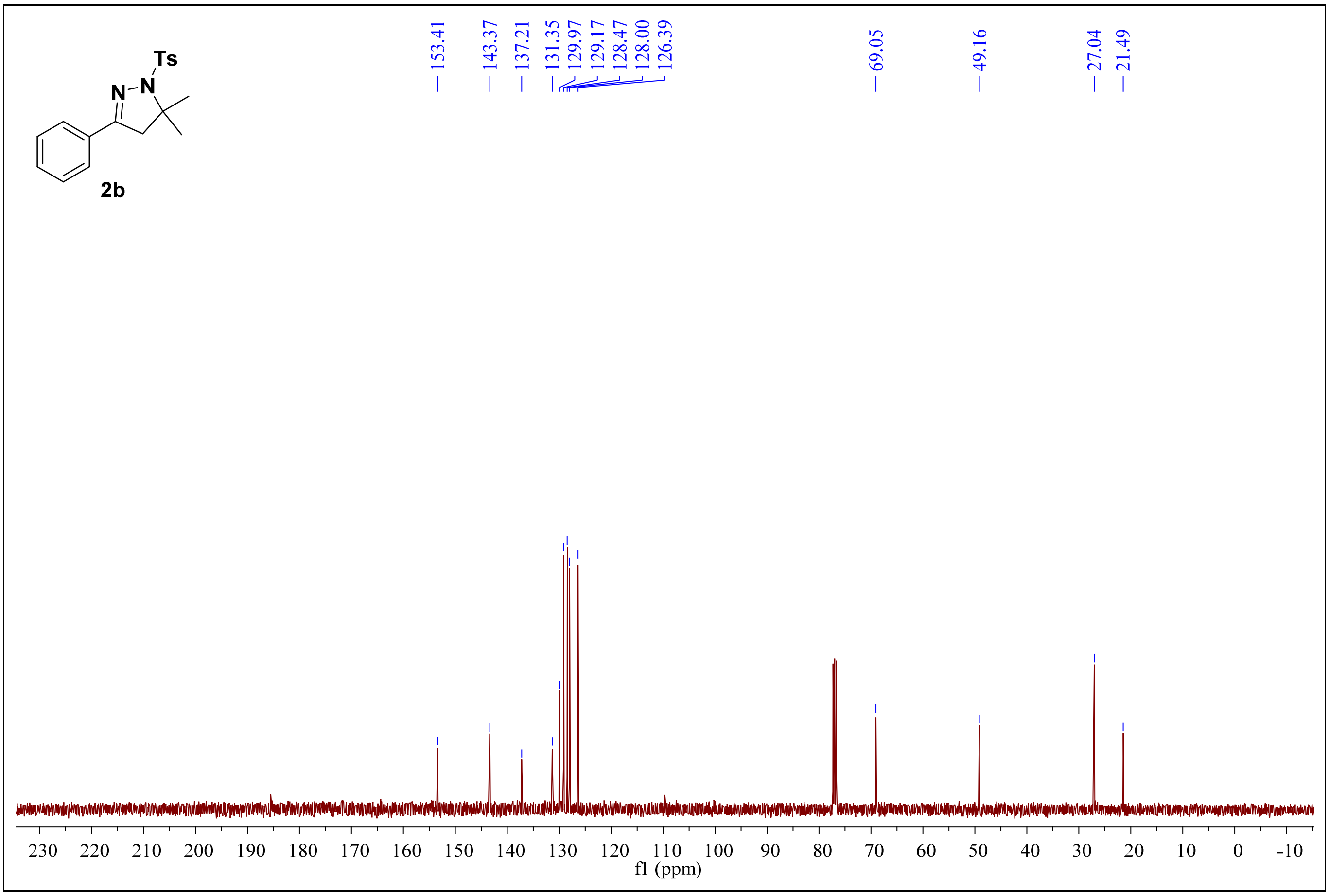

Supplementary Figure 30. **^{1}H NMR (600 MHz, $CDCl_3$) and ^{13}C NMR (100 MHz, $CDCl_3$) spectra of cyclic product 2c**

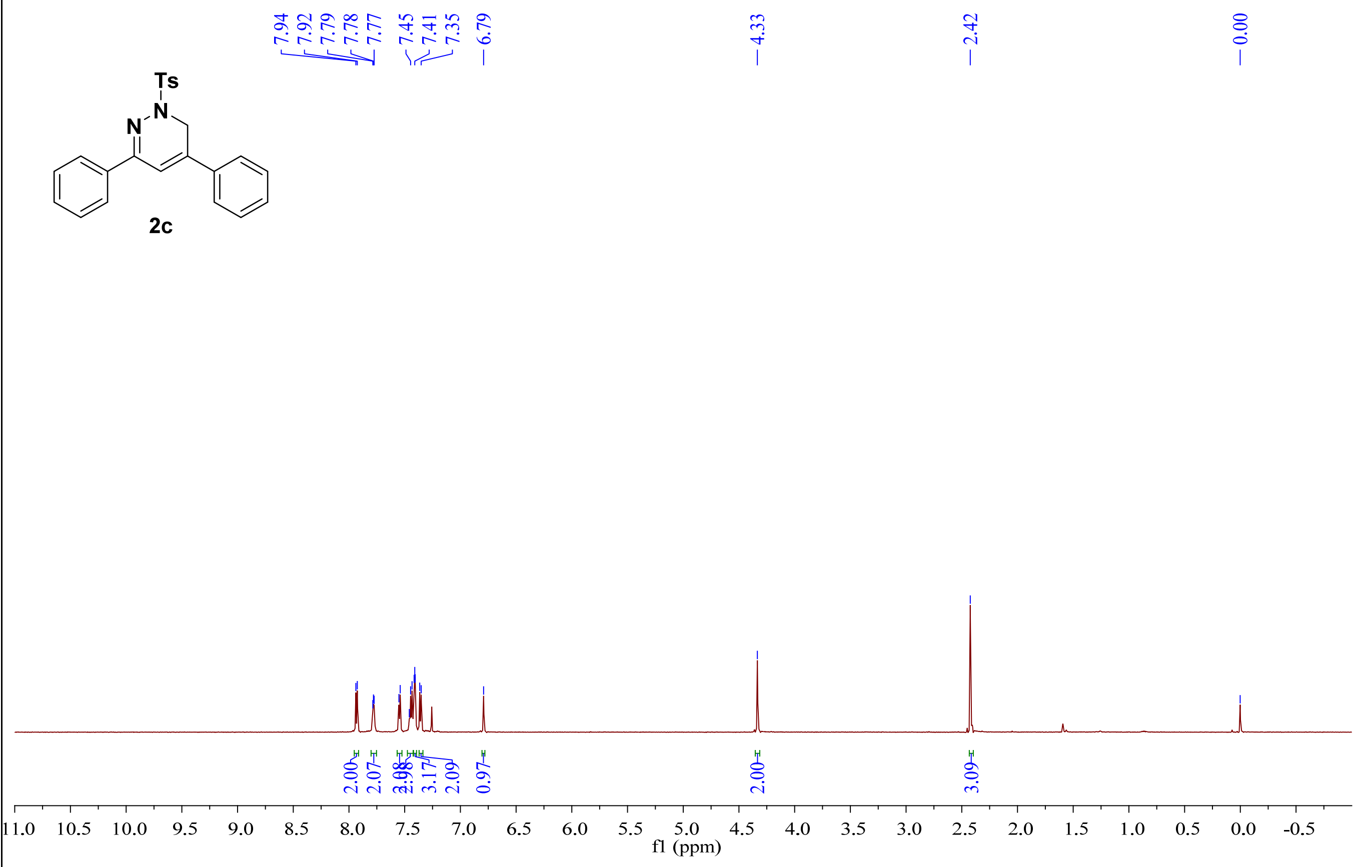

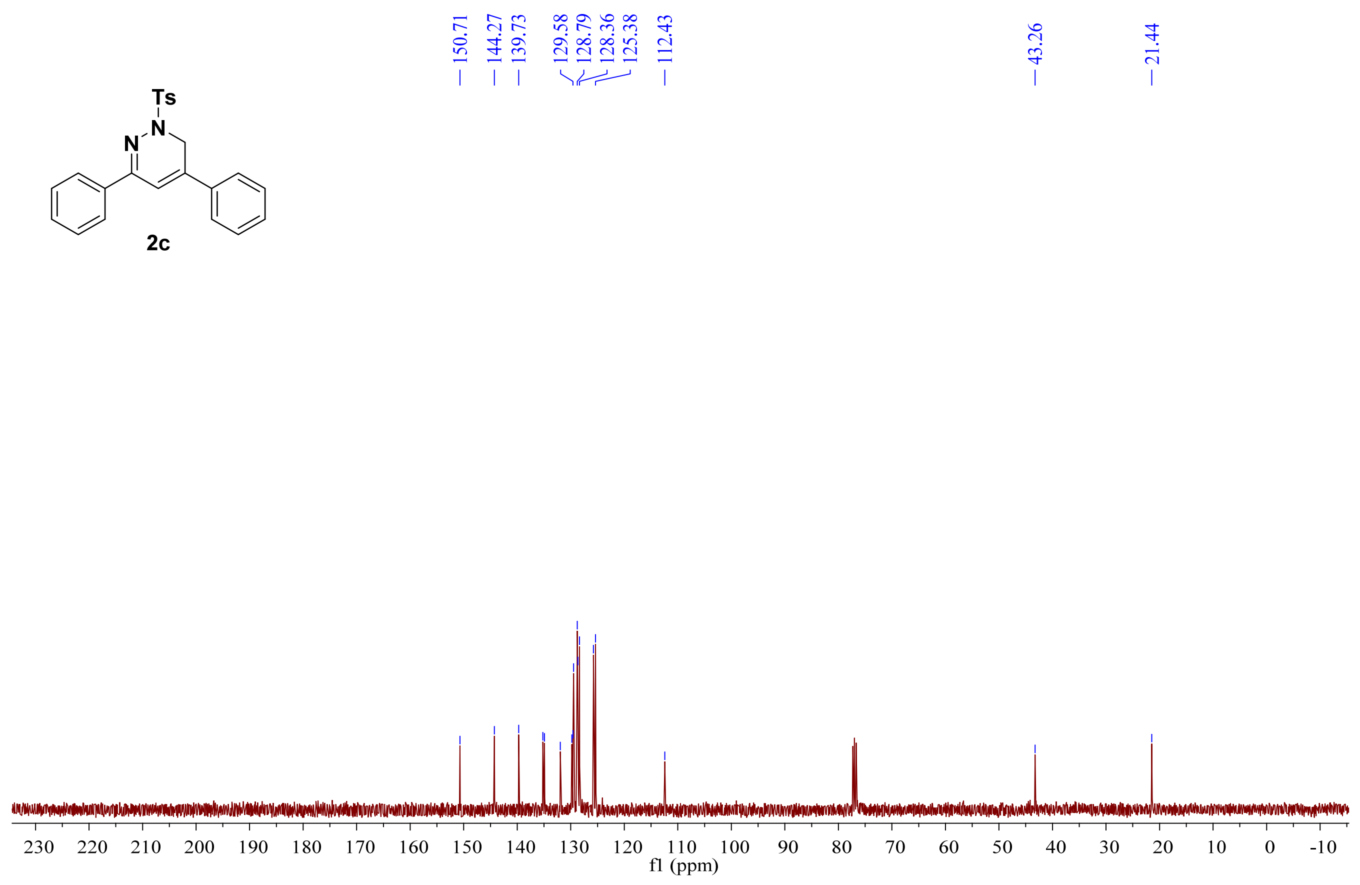

Supplementary Figure 31. **^{1}H NMR (600 MHz, $CDCl_3$) and ^{13}C NMR (100 MHz, $CDCl_3$) spectra of cyclic product 2d**

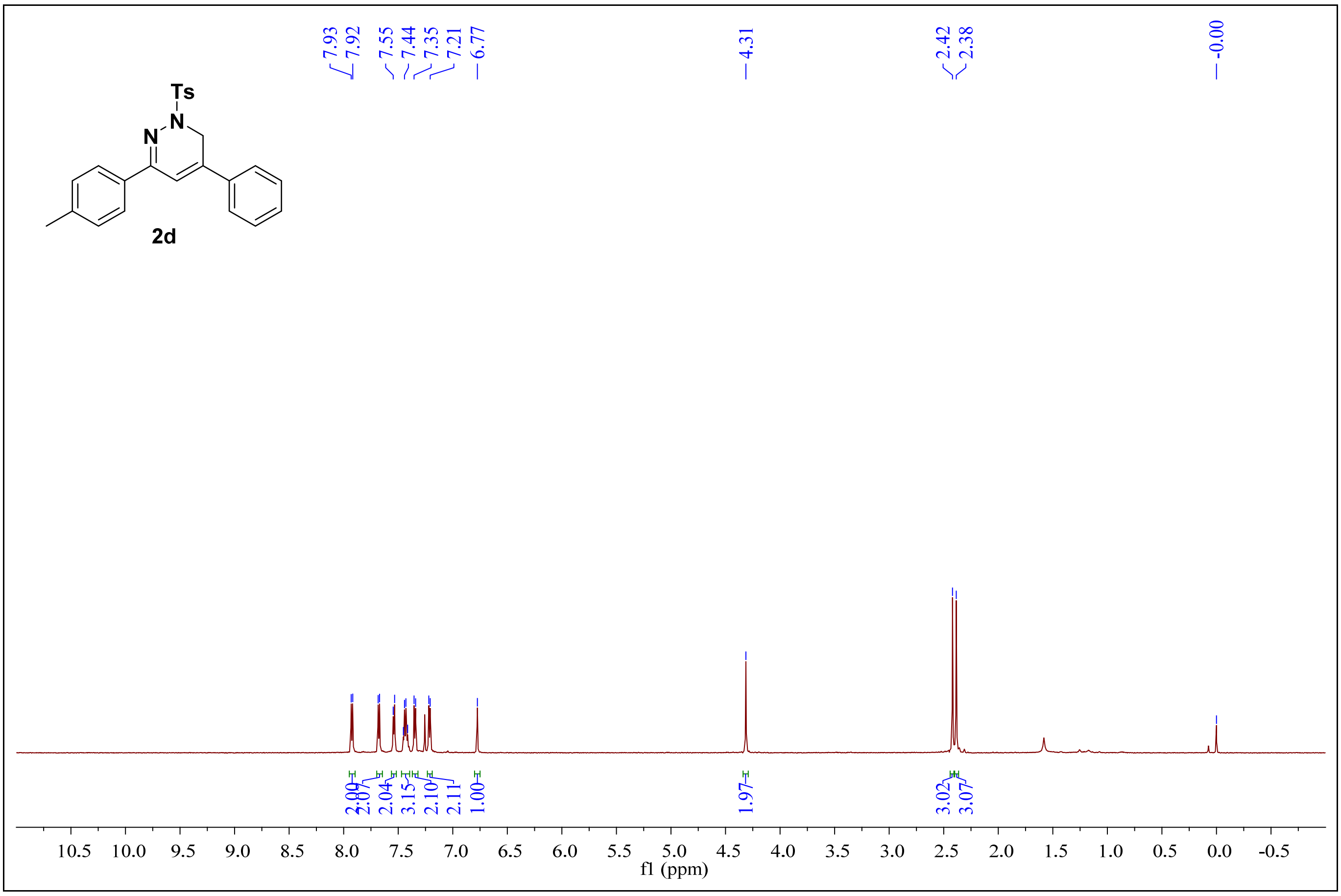

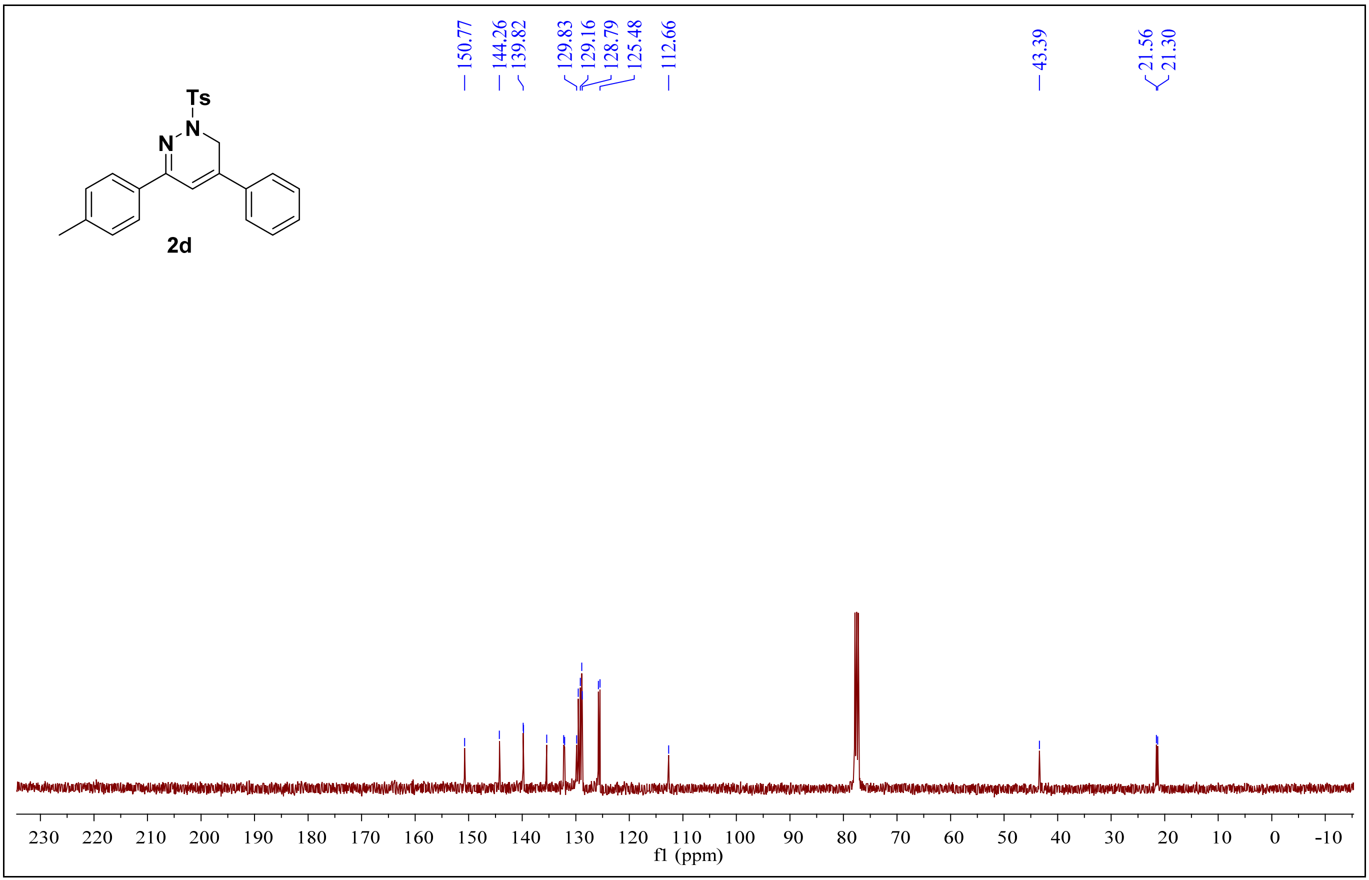

Supplementary Figure 32. **^{1}H NMR (600 MHz, $CDCl_3$) and ^{13}C NMR (100 MHz, $CDCl_3$) spectra of cyclic product 2e**

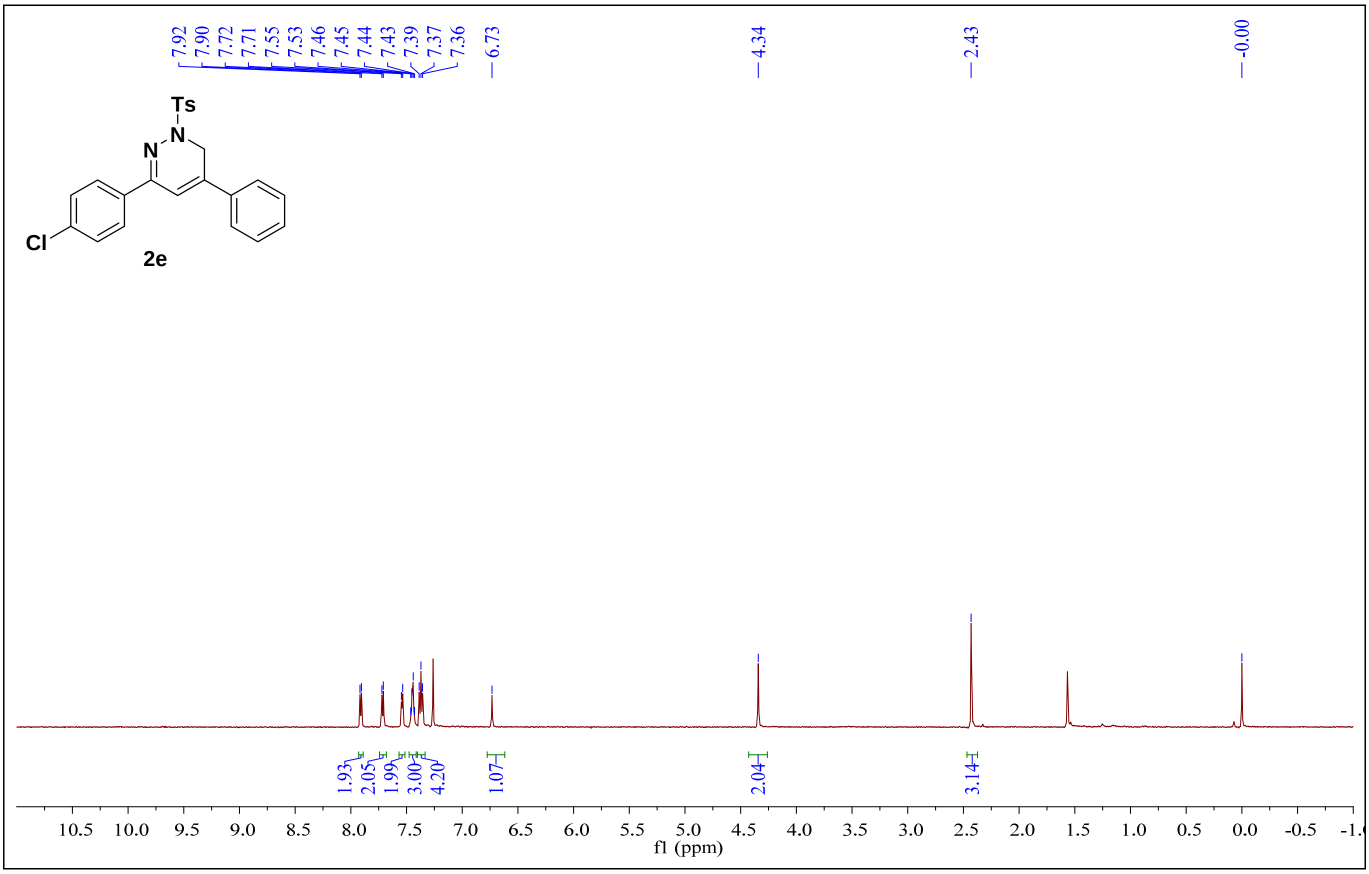

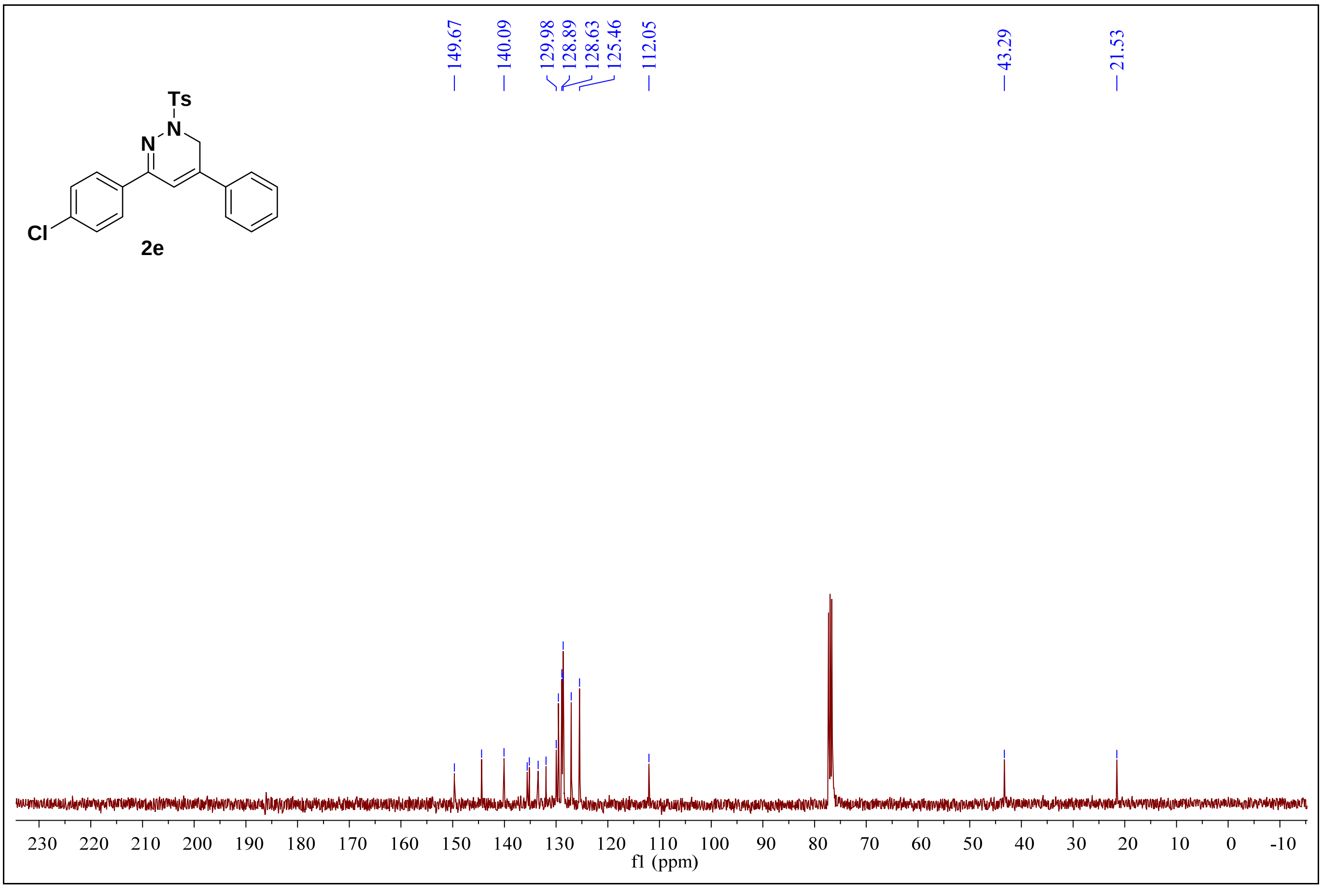

Supplementary Figure 33. ^{1}H NMR (600 MHz, $CDCl_3$) and ^{13}C NMR (100 MHz, $CDCl_3$) spectra of cyclic product **2f**

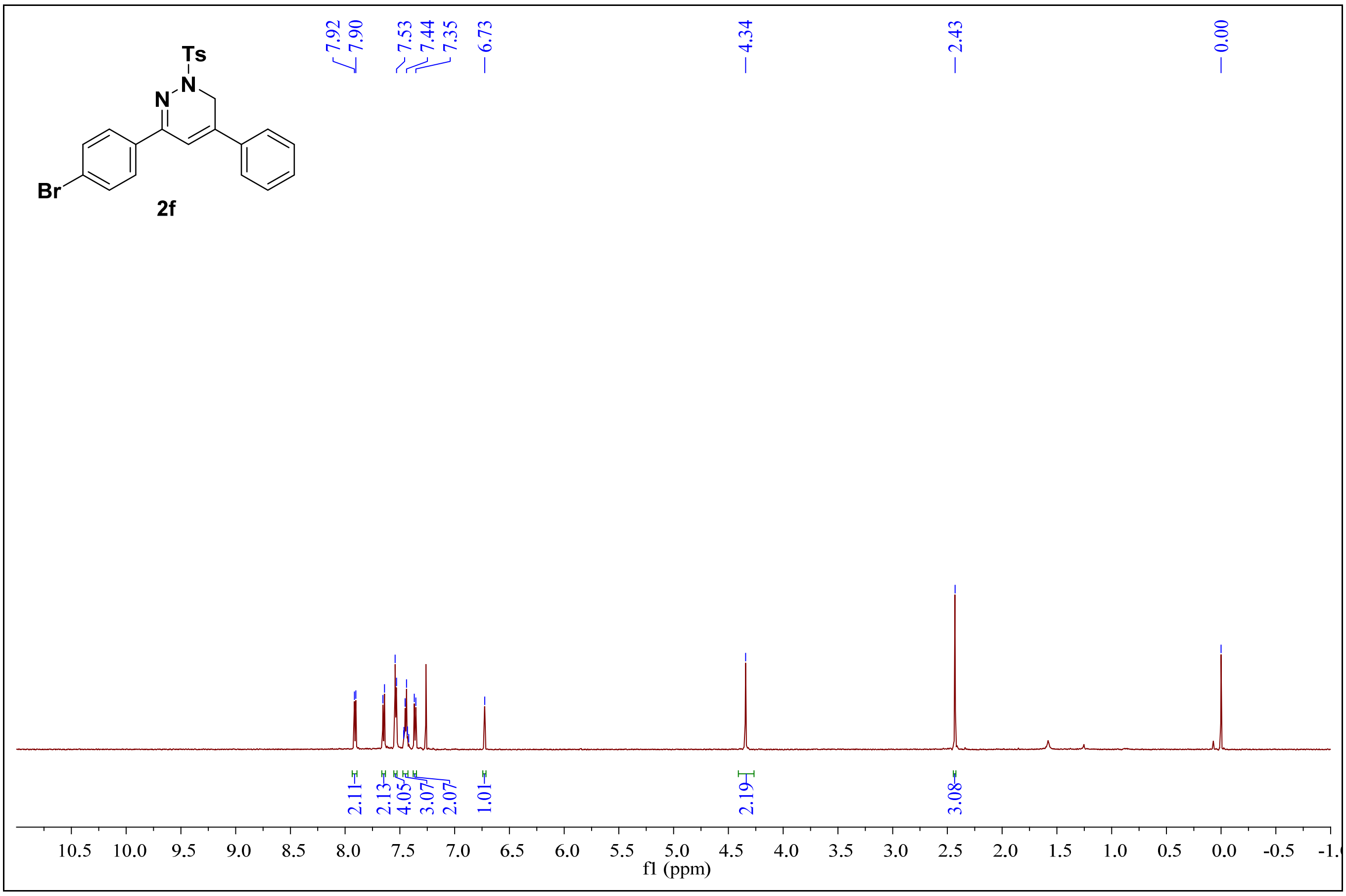

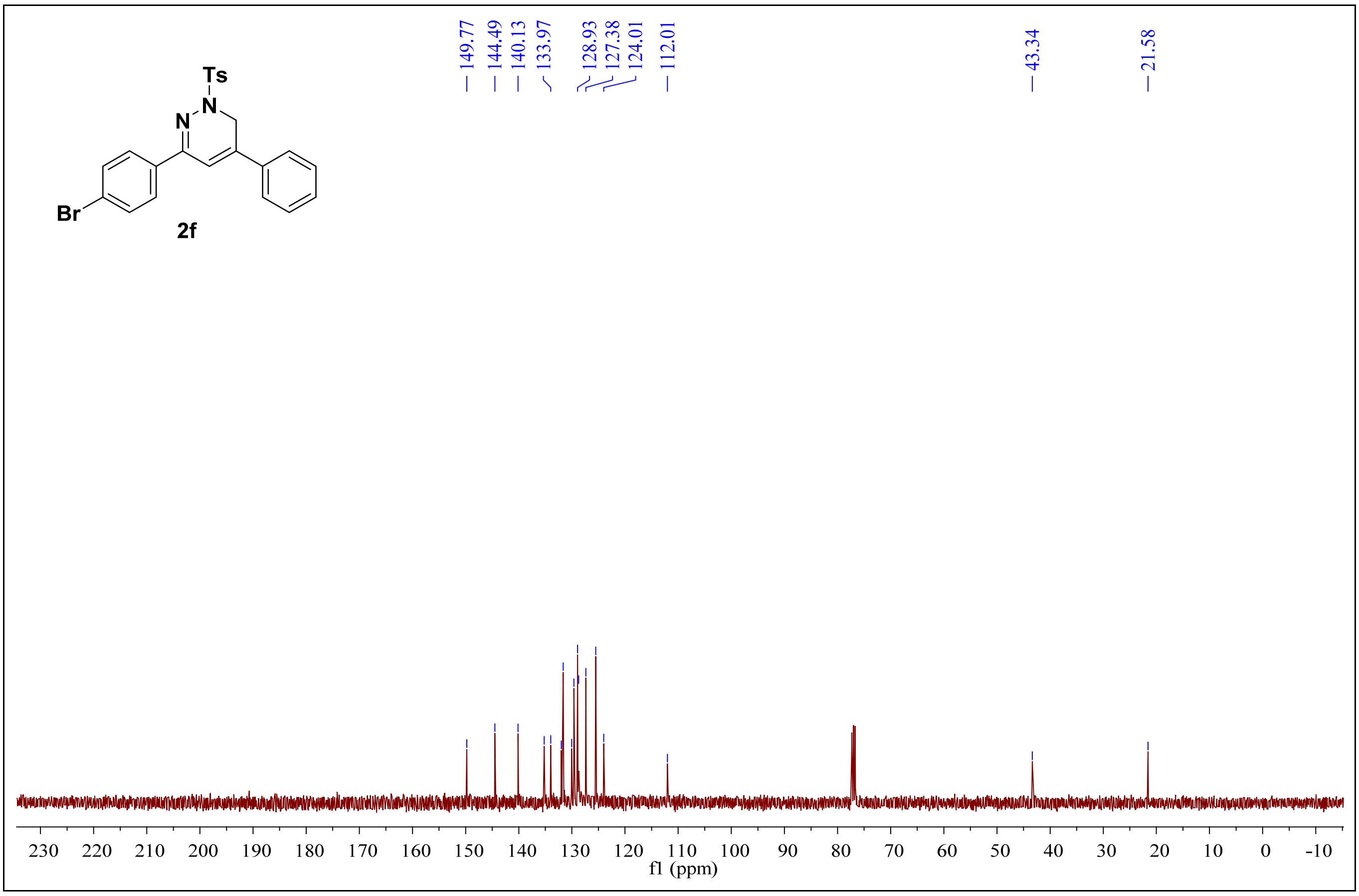

Supplementary Figure 34. **^{1}H NMR (600 MHz, $CDCl_3$), ^{13}C NMR (100 MHz, $CDCl_3$) and ^{19}F NMR (376 MHz, $CDCl_3$) spectra of cyclic product 2g**

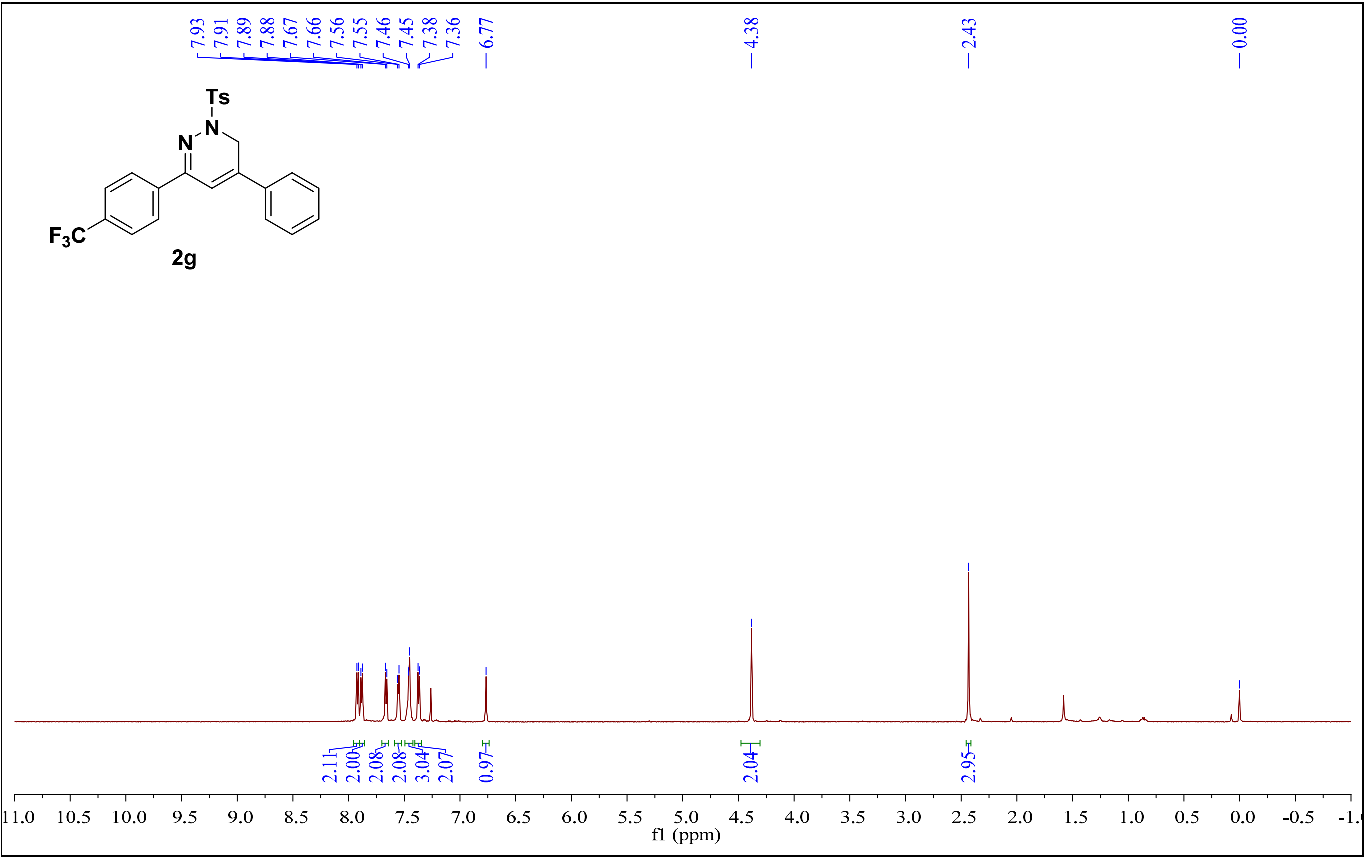

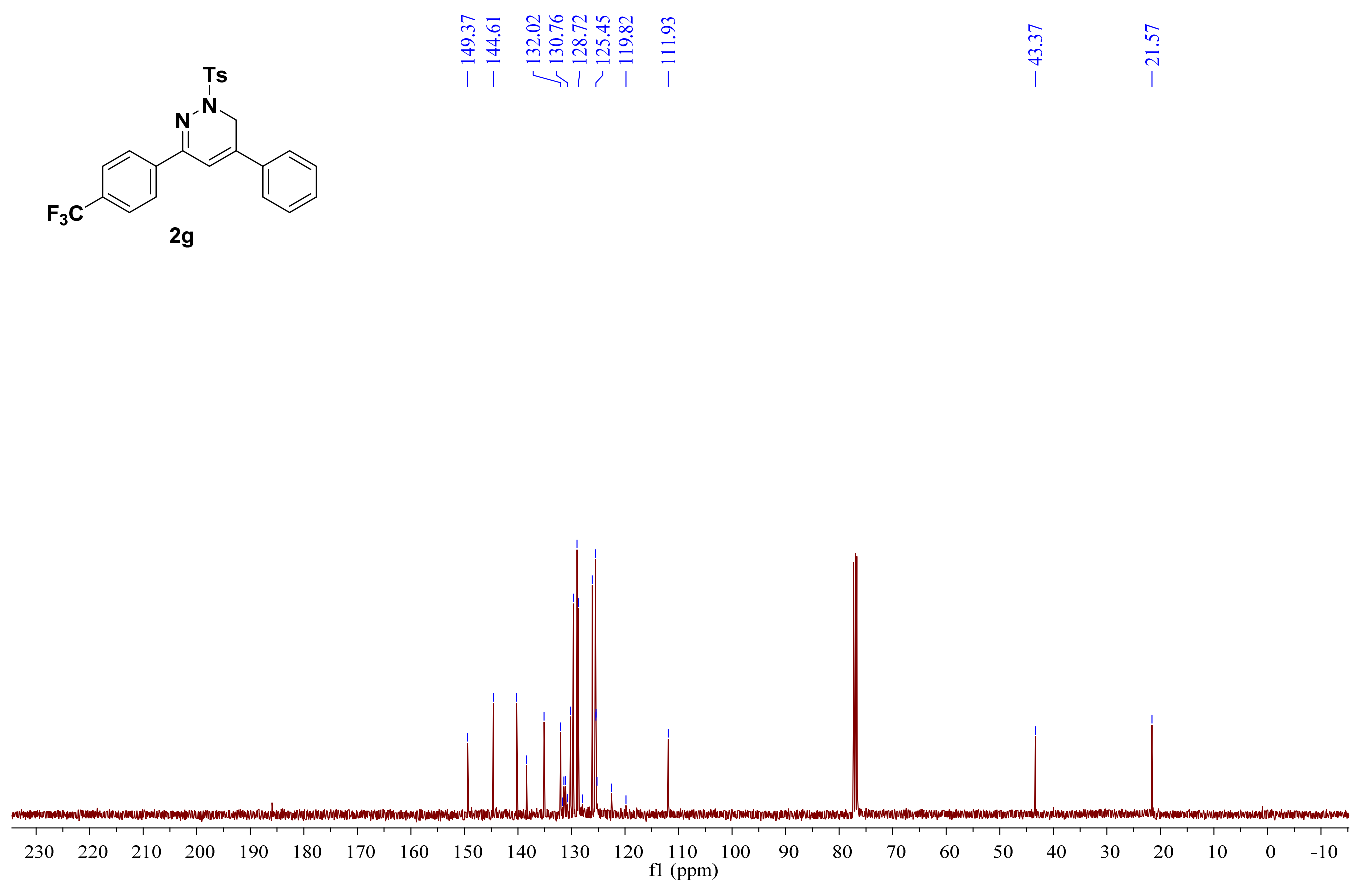

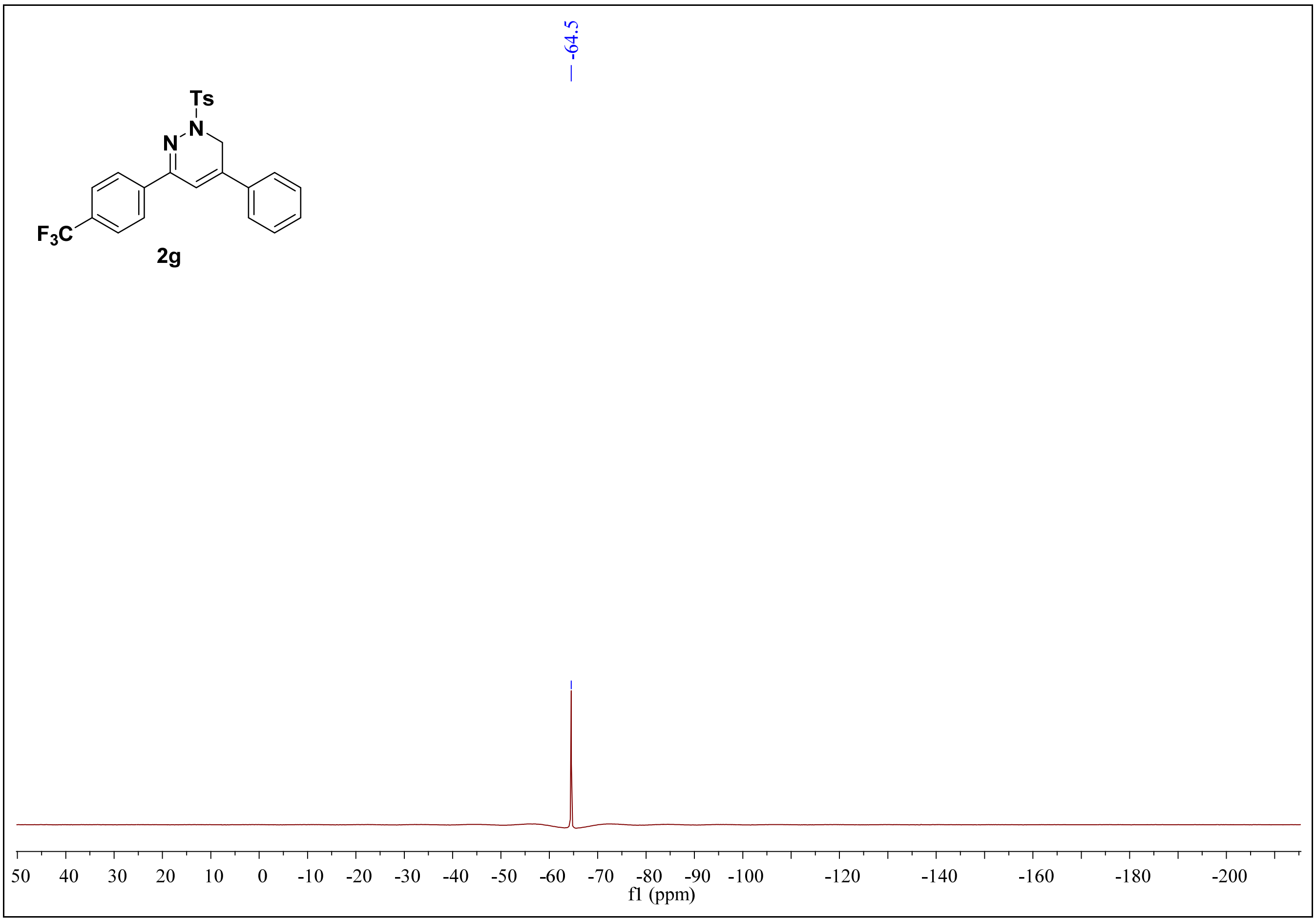

-64.5
Ts
N
N
F3C
2g
50 40 30 20 10 0 -10 -20 -30 -40 -50 -60 -70 -80 -90 -100 -120 -140 -160 -180 -200
f1 (ppm)

Supplementary Figure 35. **^{1}H NMR (400 MHz, $CDCl_3$) and ^{13}C NMR (100 MHz, $CDCl_3$) spectra of cyclic product 2h**

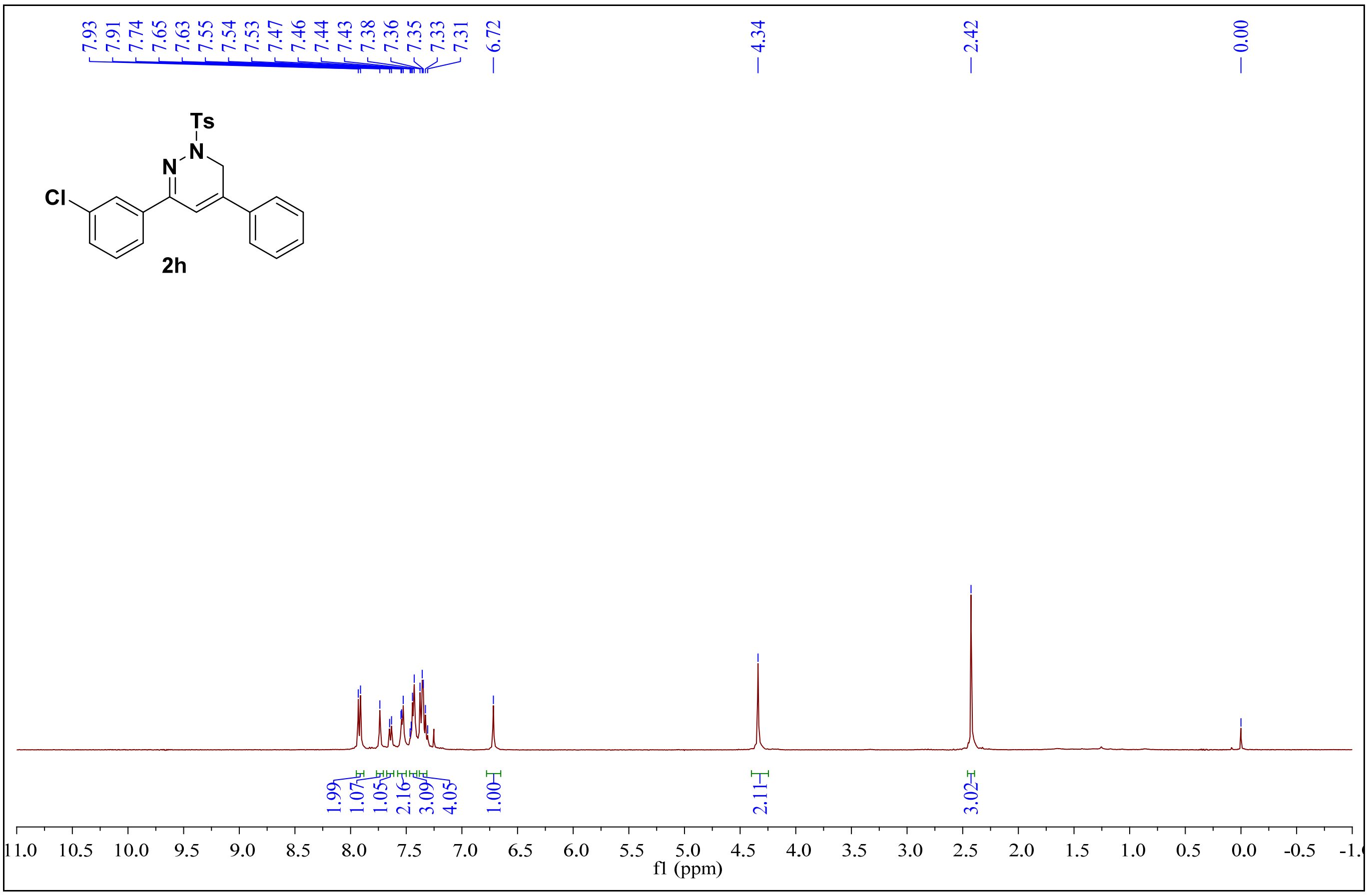

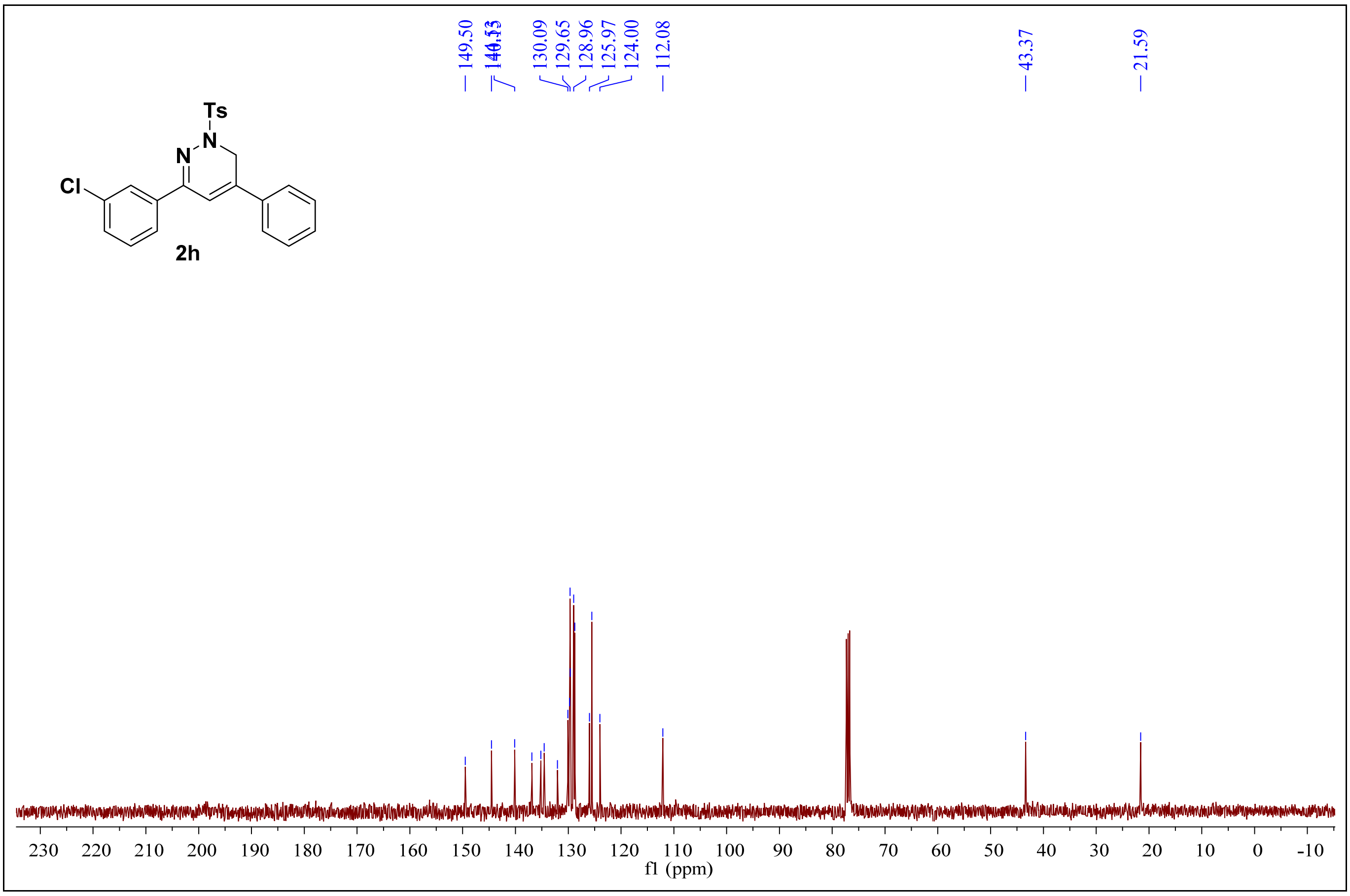

Supplementary Figure 36. ^{1}H NMR (400 MHz, $CDCl_3$) and ^{13}C NMR (100 MHz, $CDCl_3$) spectra of cyclic product **2i**

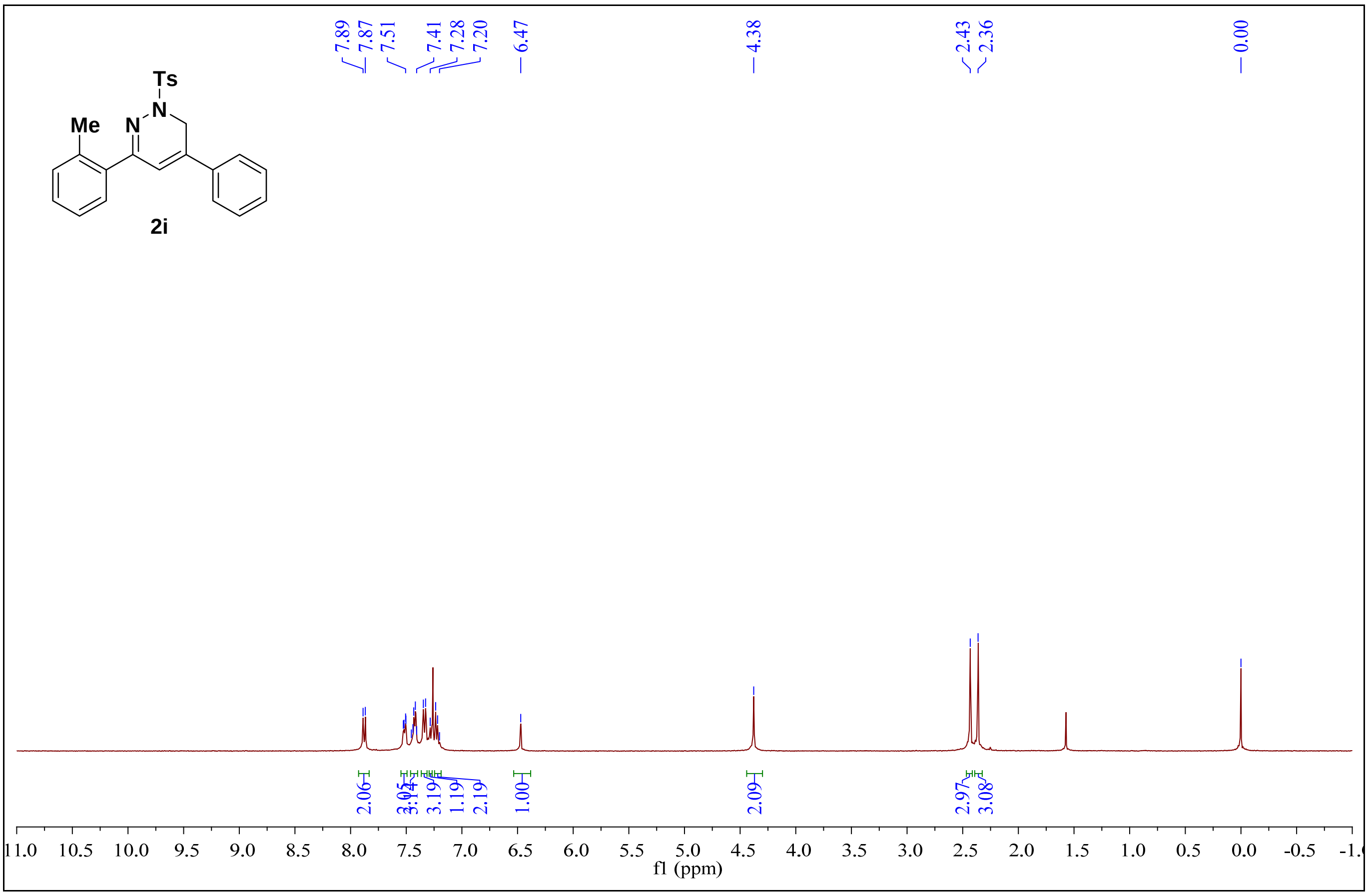

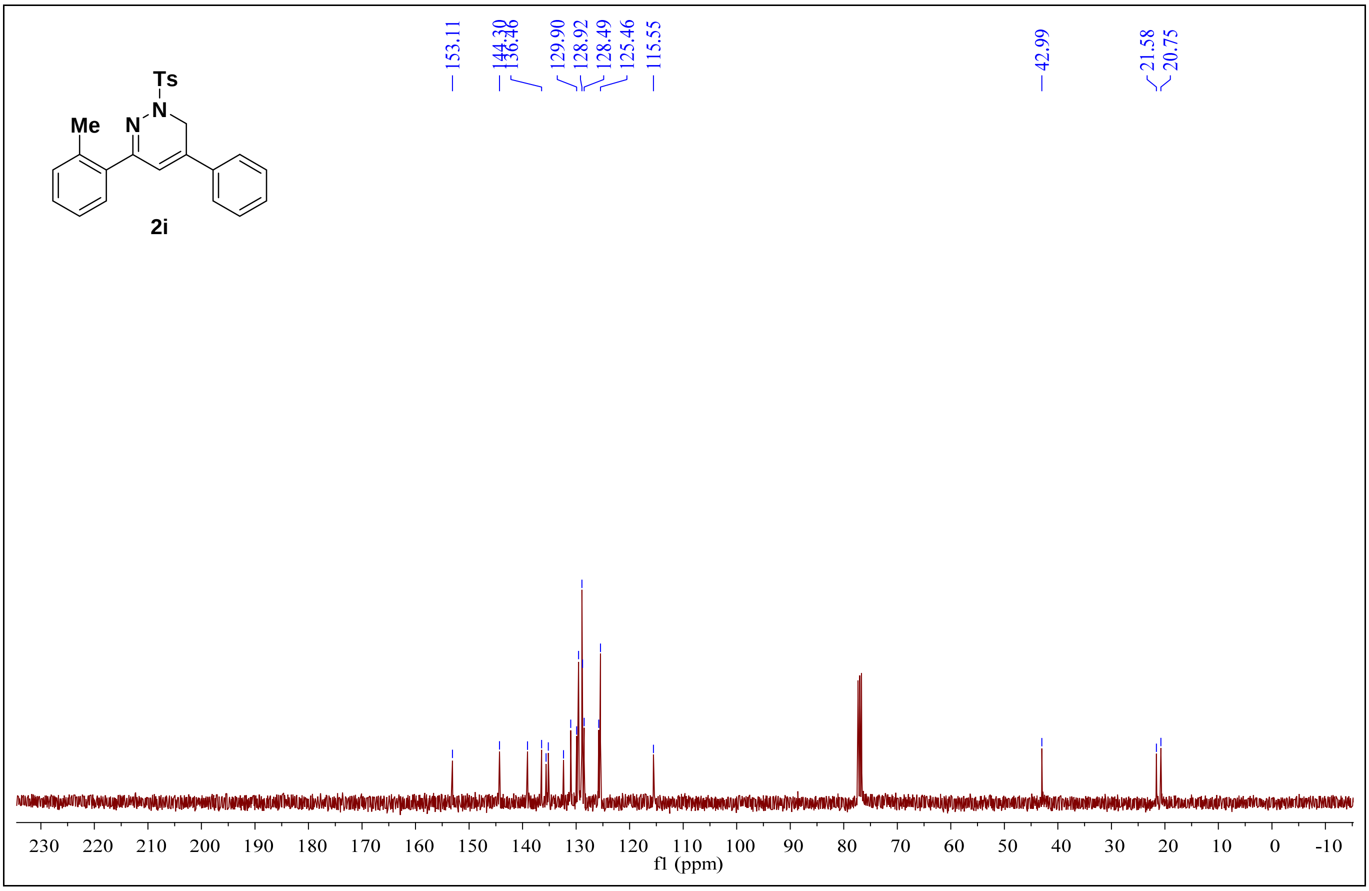

Supplementary Figure 37. ^{1}H **NMR (600 MHz, CDCl$_3$) and** ^{13}C **NMR (100 MHz, CDCl$_3$) spectra of cyclic product 2j**

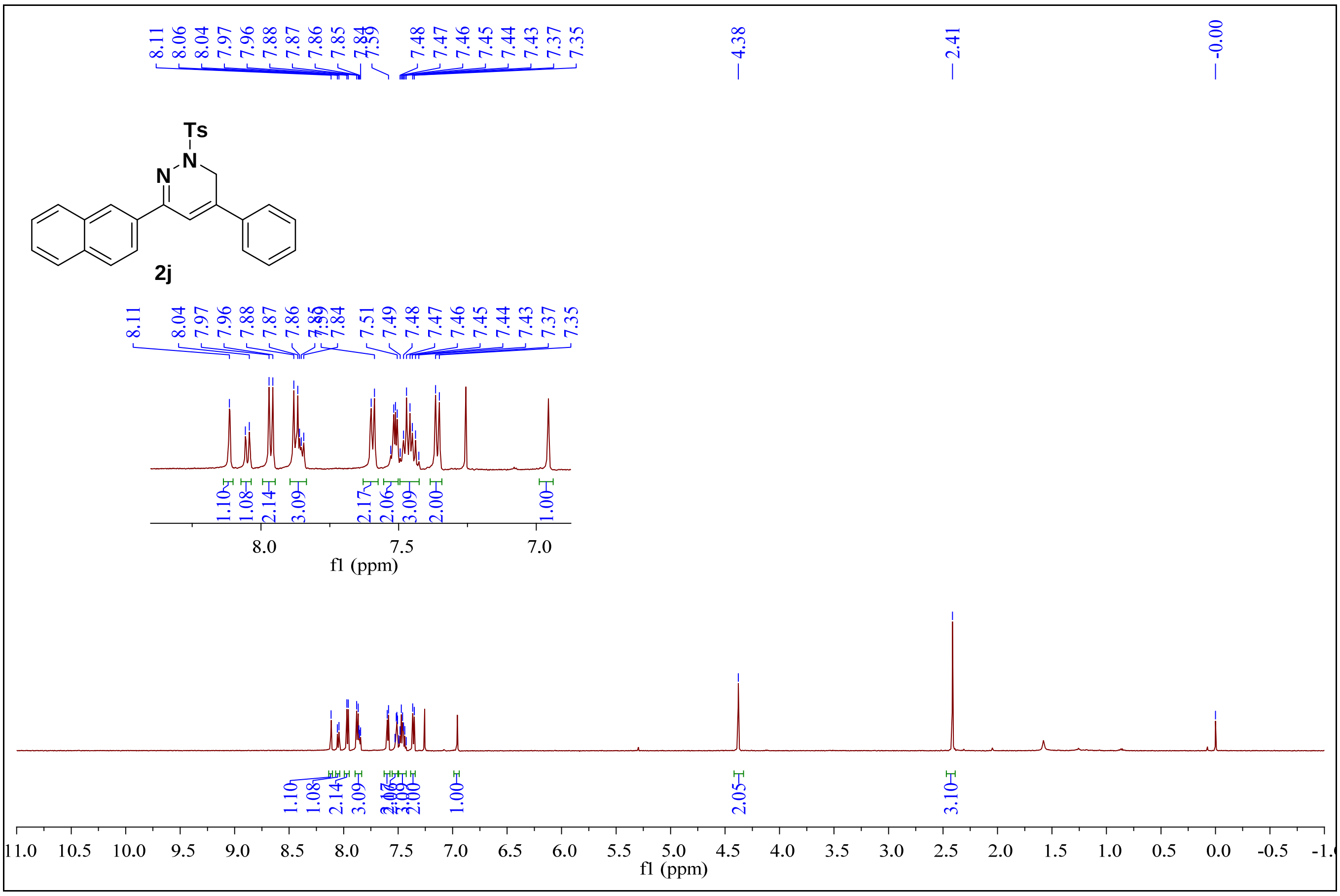

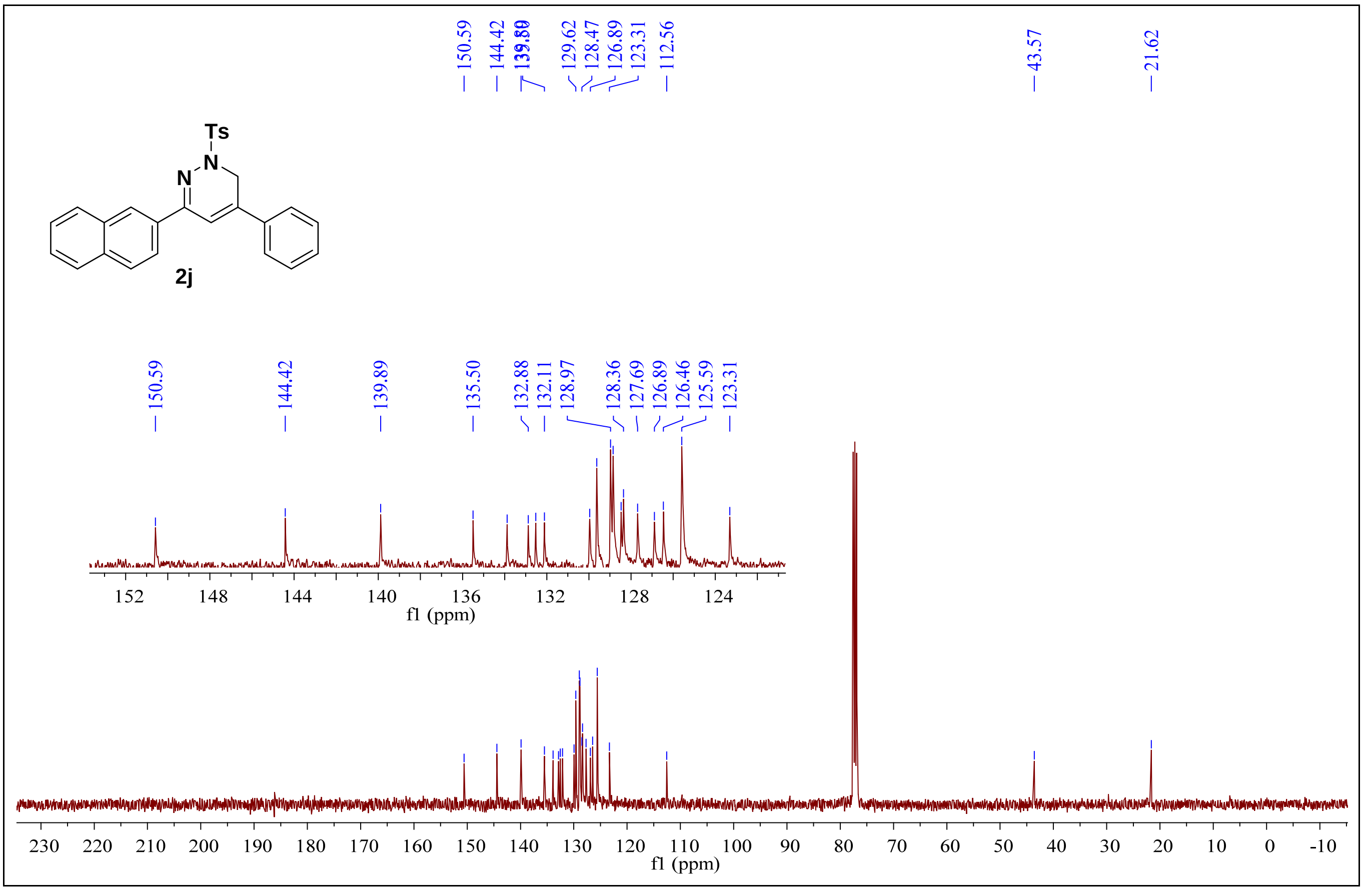

Supplementary Figure 38. ^{1}H NMR (600 MHz, $CDCl_3$) and ^{13}C NMR (100 MHz, $CDCl_3$) spectra of cyclic product 2k

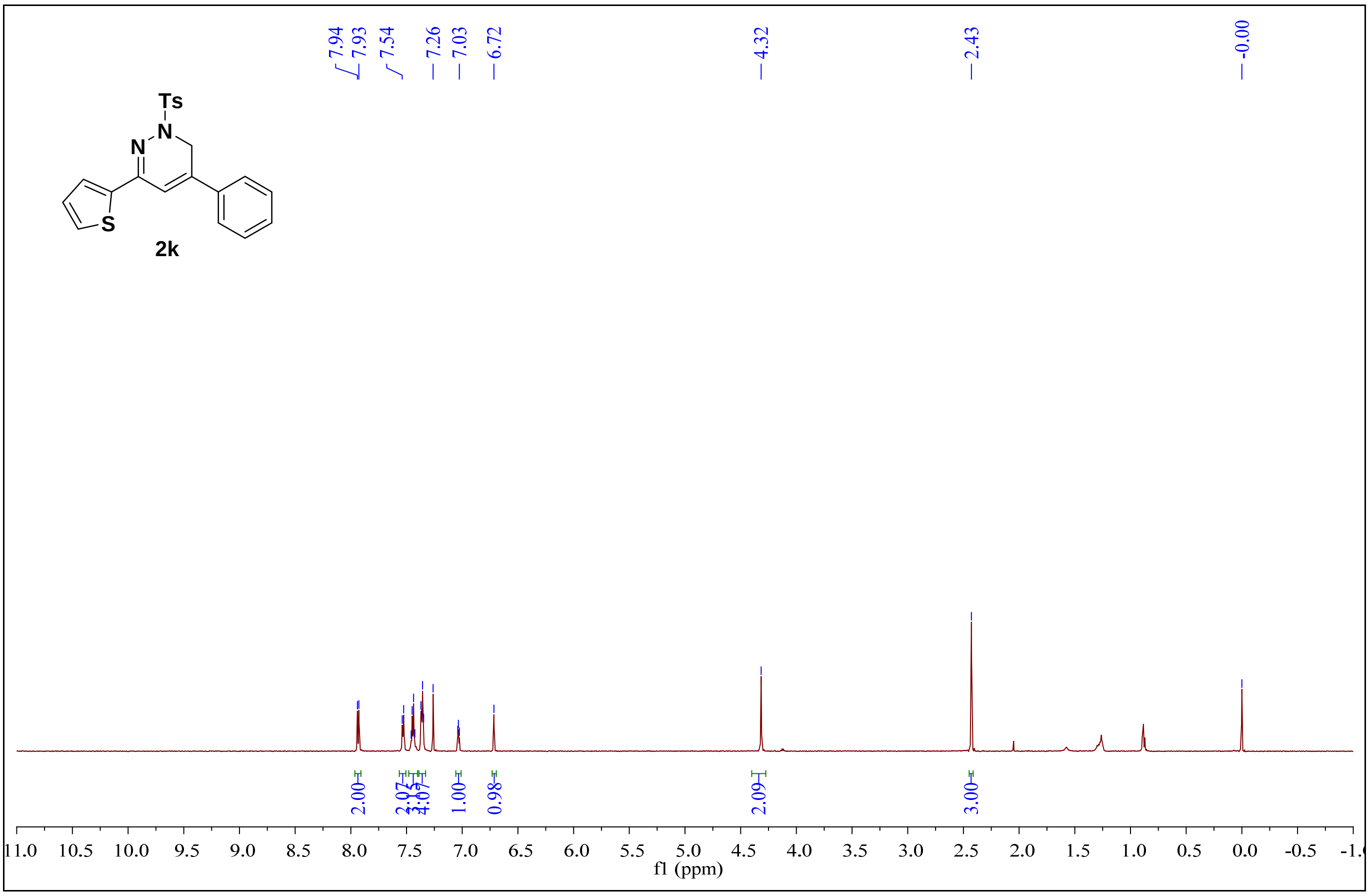

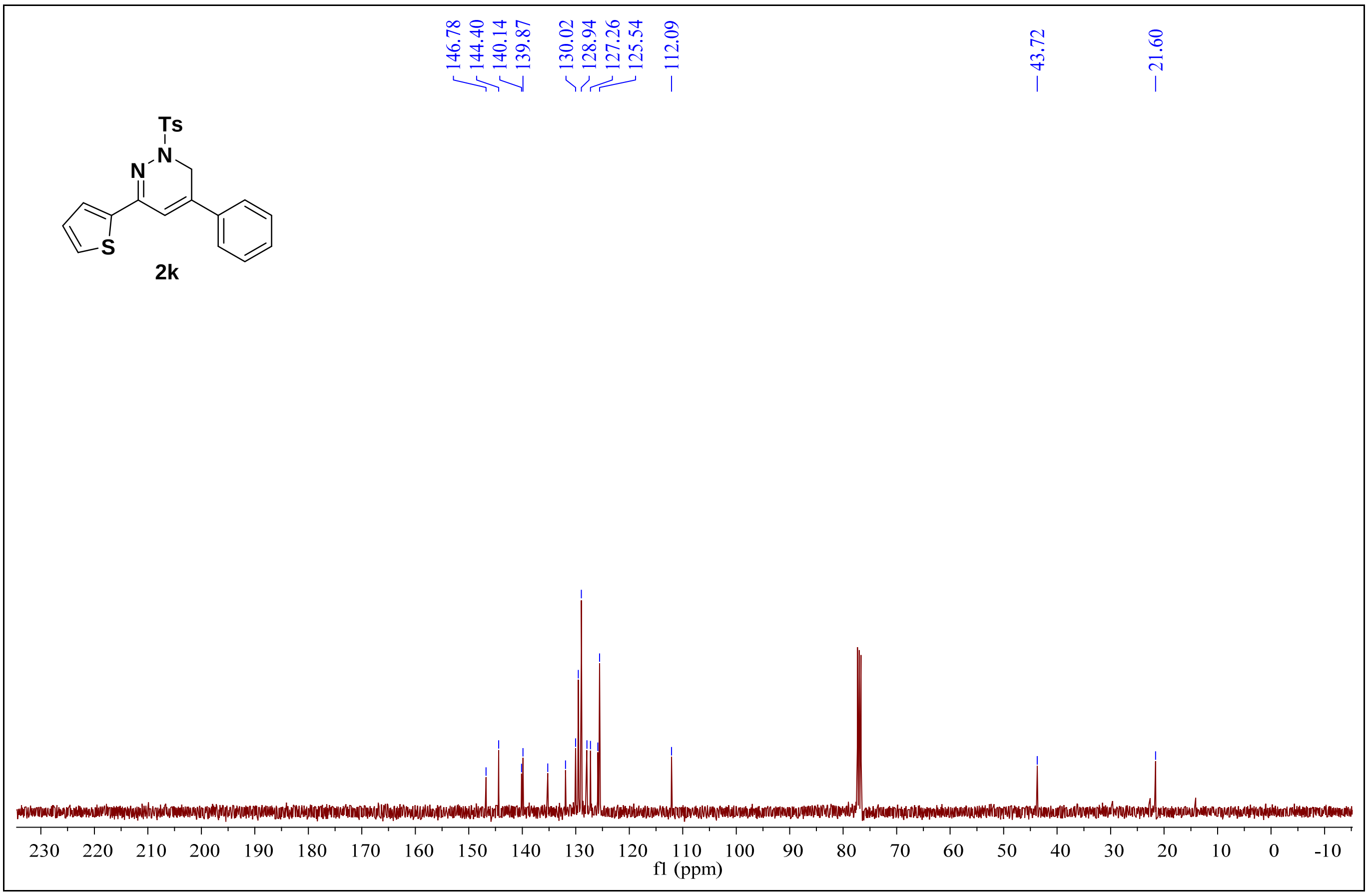

Supplementary Figure 39. ^{1}**H NMR (600 MHz, CDCl$_3$) and** ^{13}**C NMR (100 MHz, CDCl$_3$) spectra of cyclic product 2l**

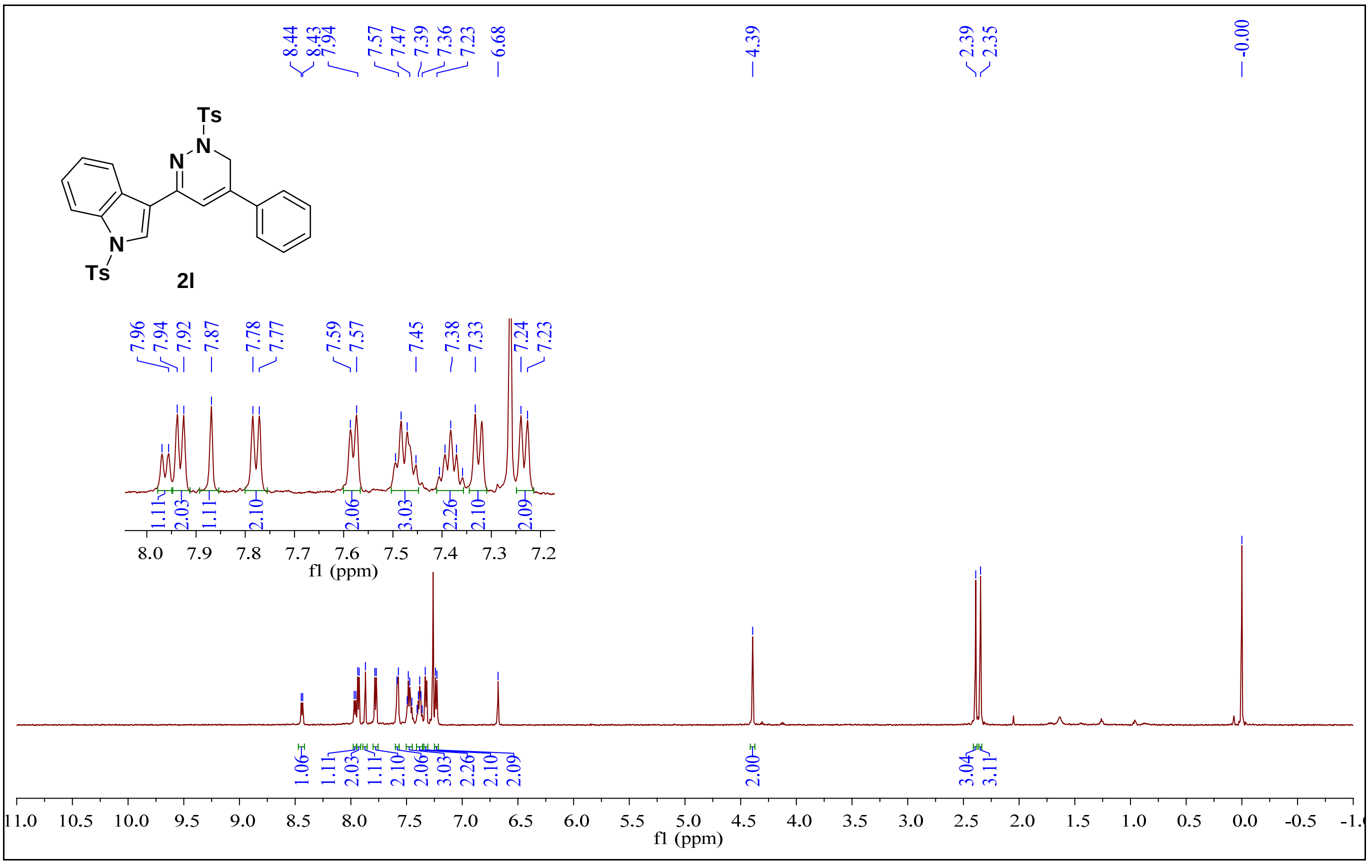

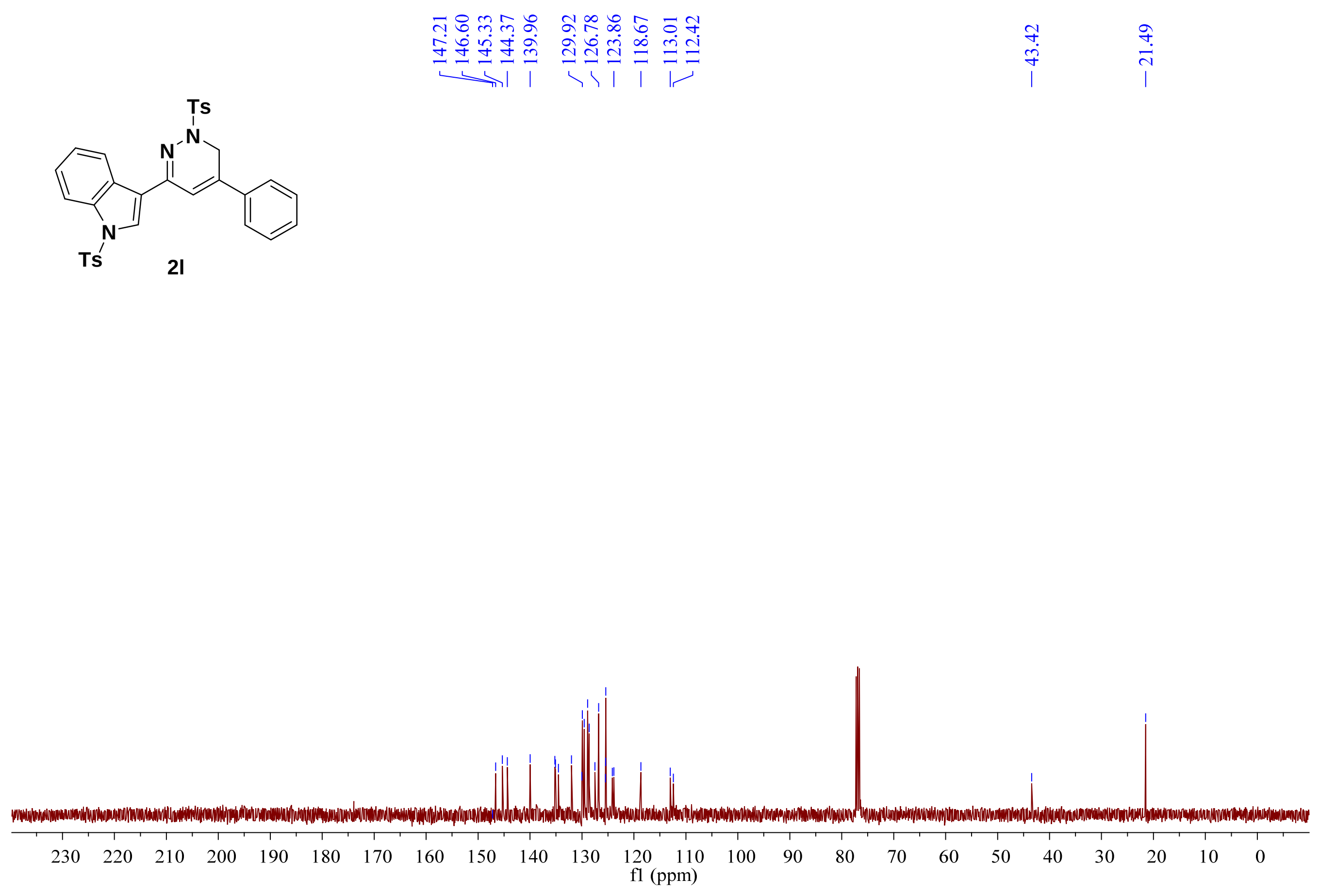

Supplementary Figure 40. ^{1}H **NMR (400 MHz, $CDCl_3$) and** ^{13}C **NMR (100 MHz, $CDCl_3$) spectra of cyclic product 2m**

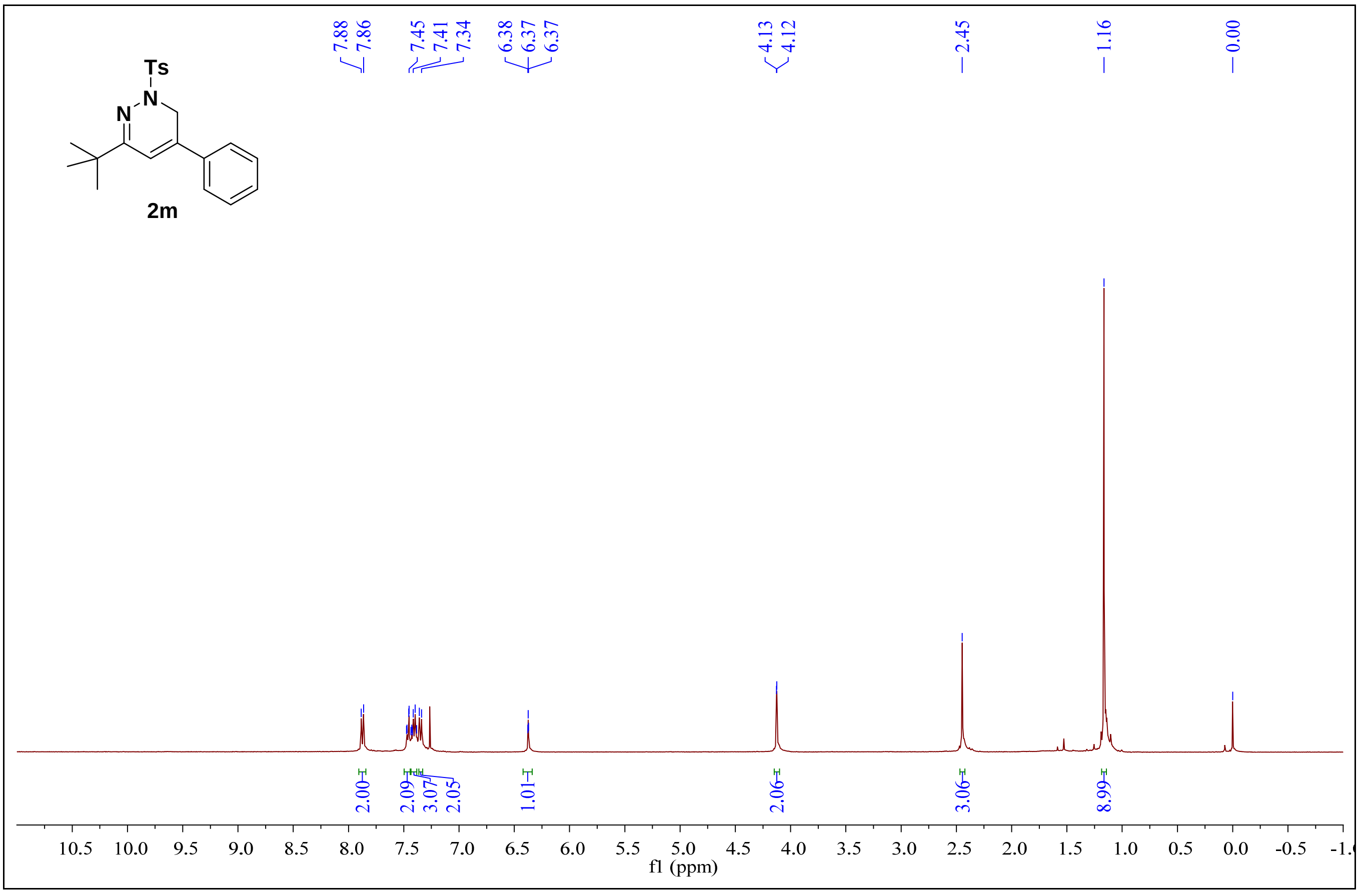

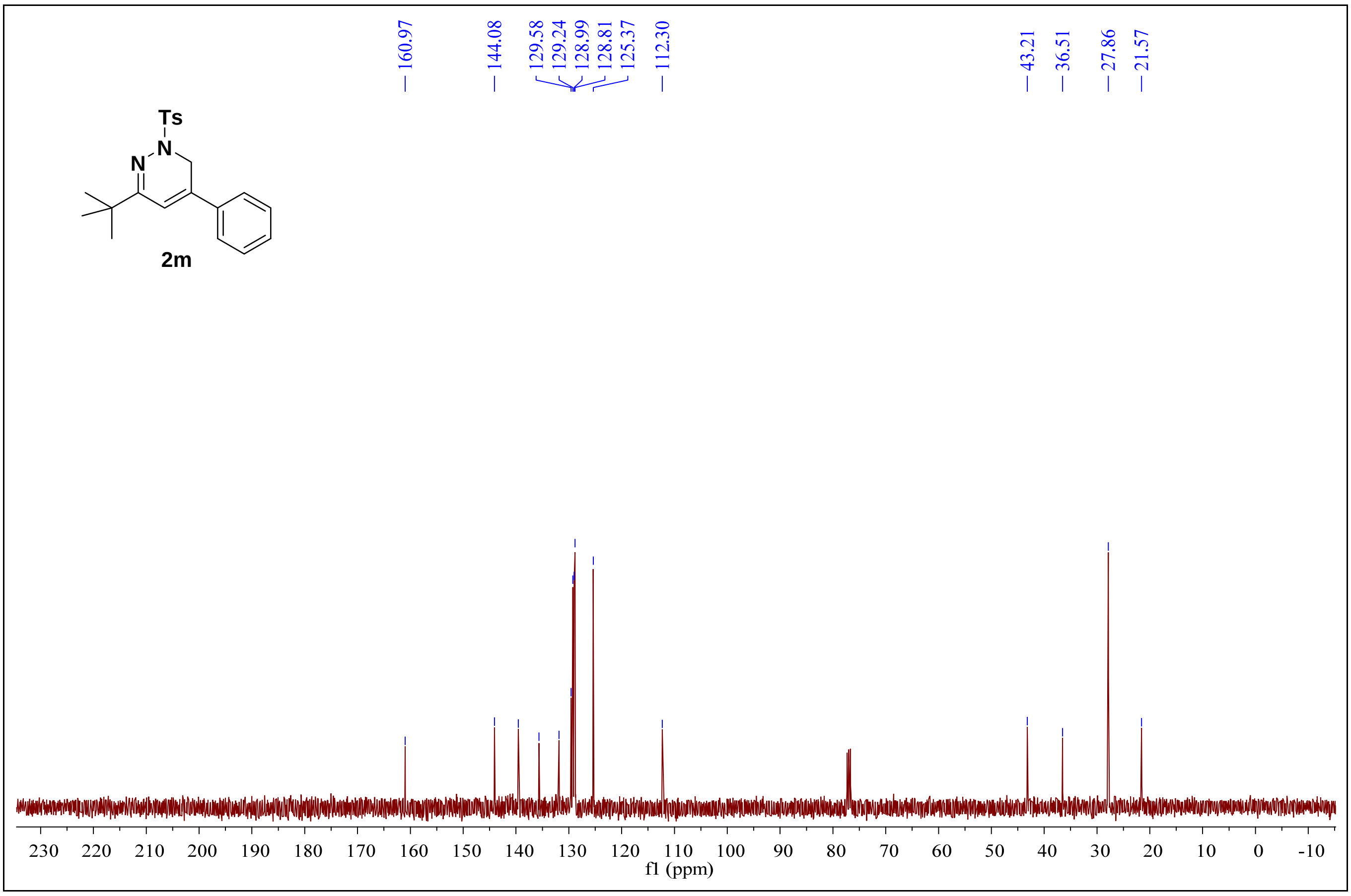

Supplementary Figure 41. ^{1}**H NMR (600 MHz,** $CDCl_3$**) and** ^{13}**C NMR (100 MHz,** $CDCl_3$**) spectra of cyclic product 2n**

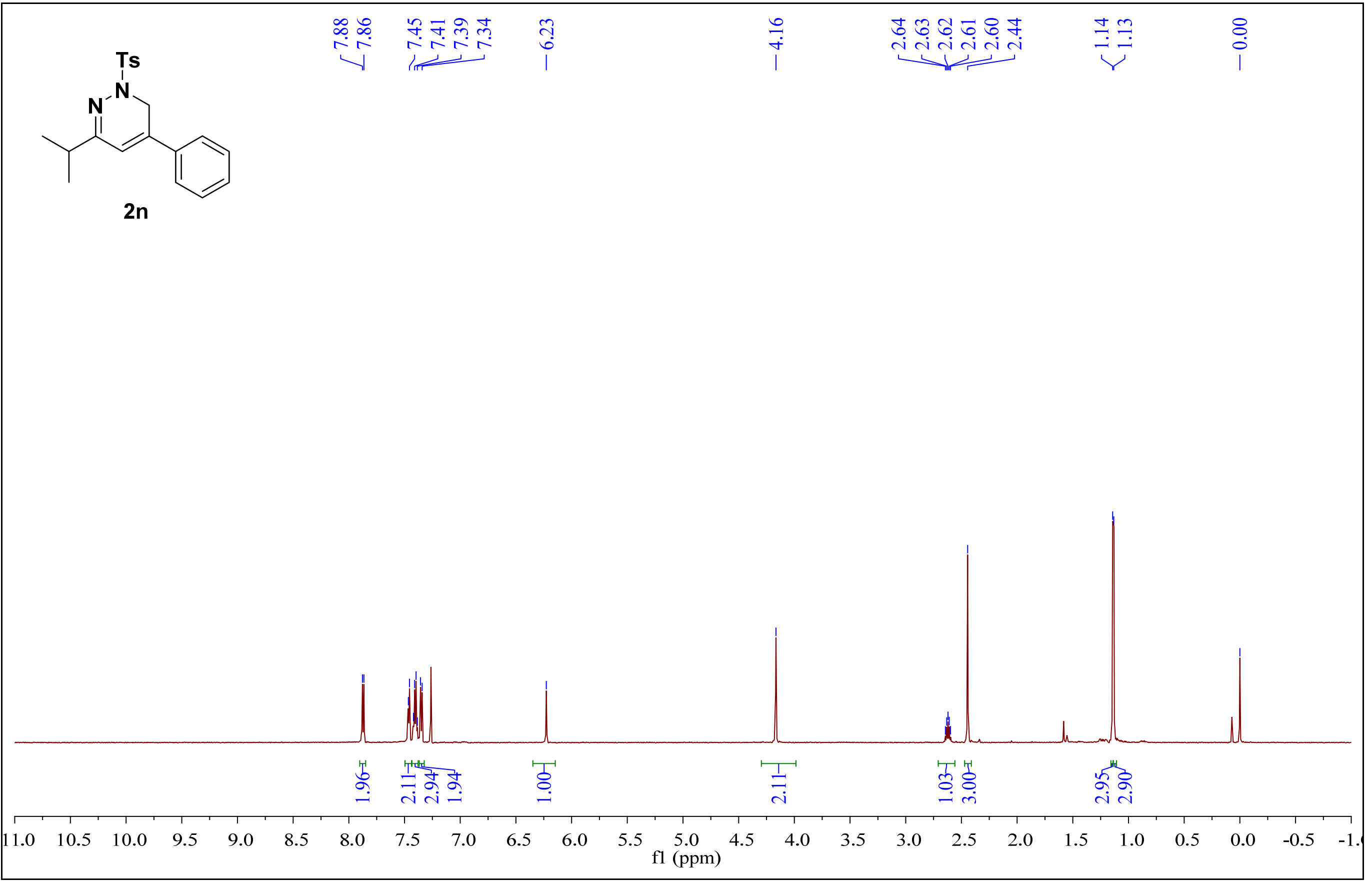

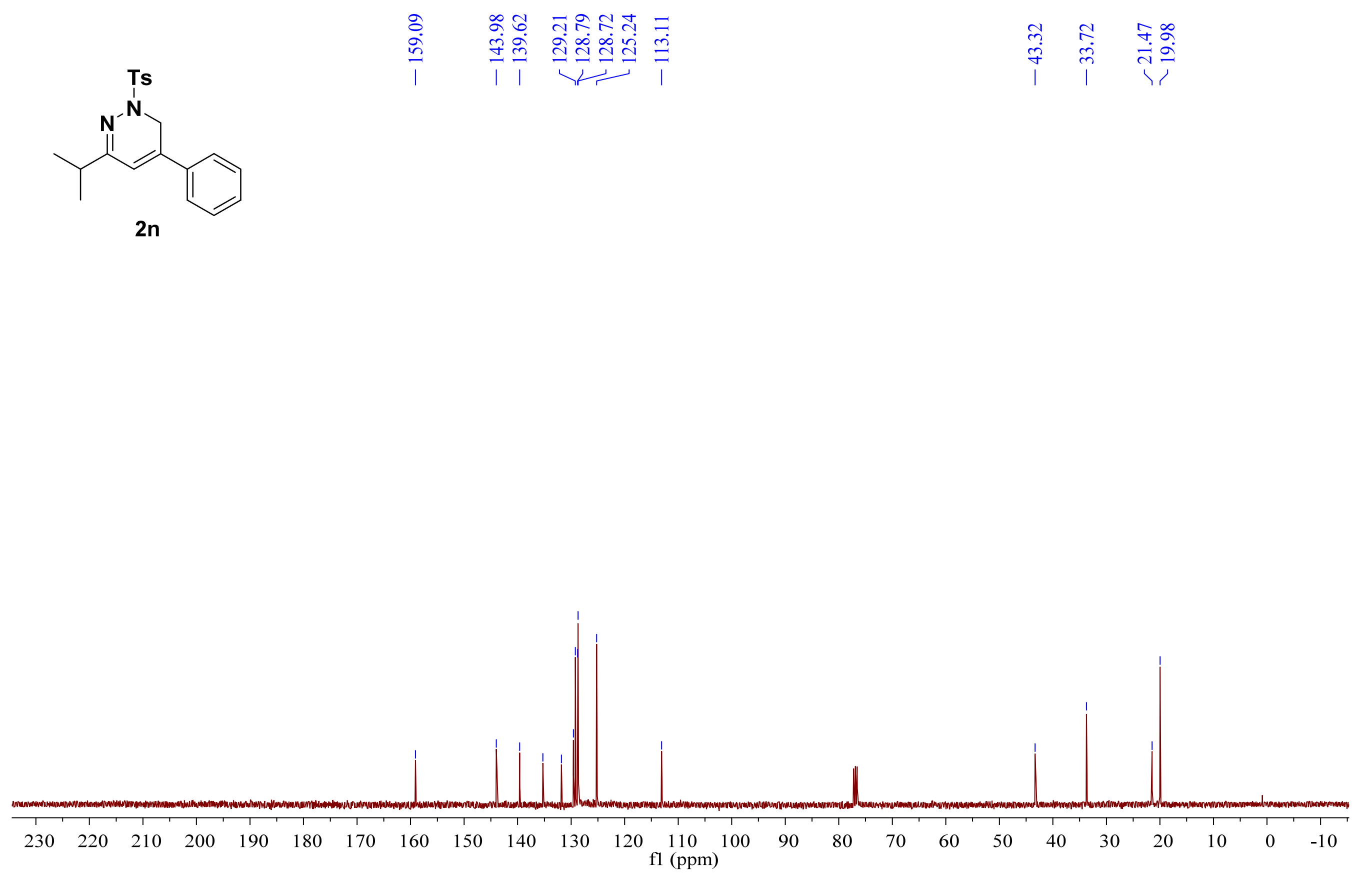

Supplementary Figure 42. ^{1}H **NMR (600 MHz,** $CDCl_3$**) and** ^{13}C **NMR (100 MHz,** $CDCl_3$**) spectra of cyclic product 2o**

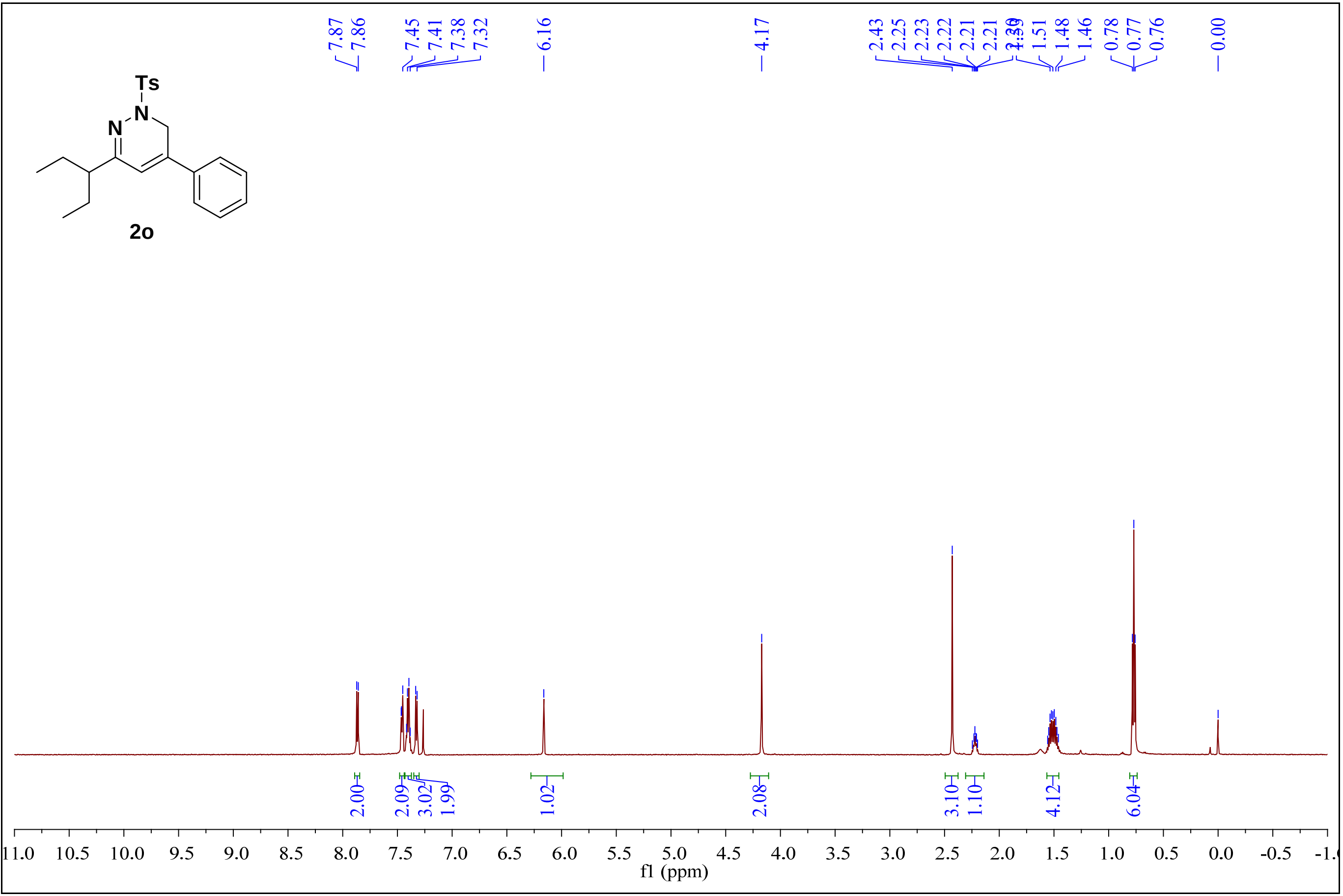

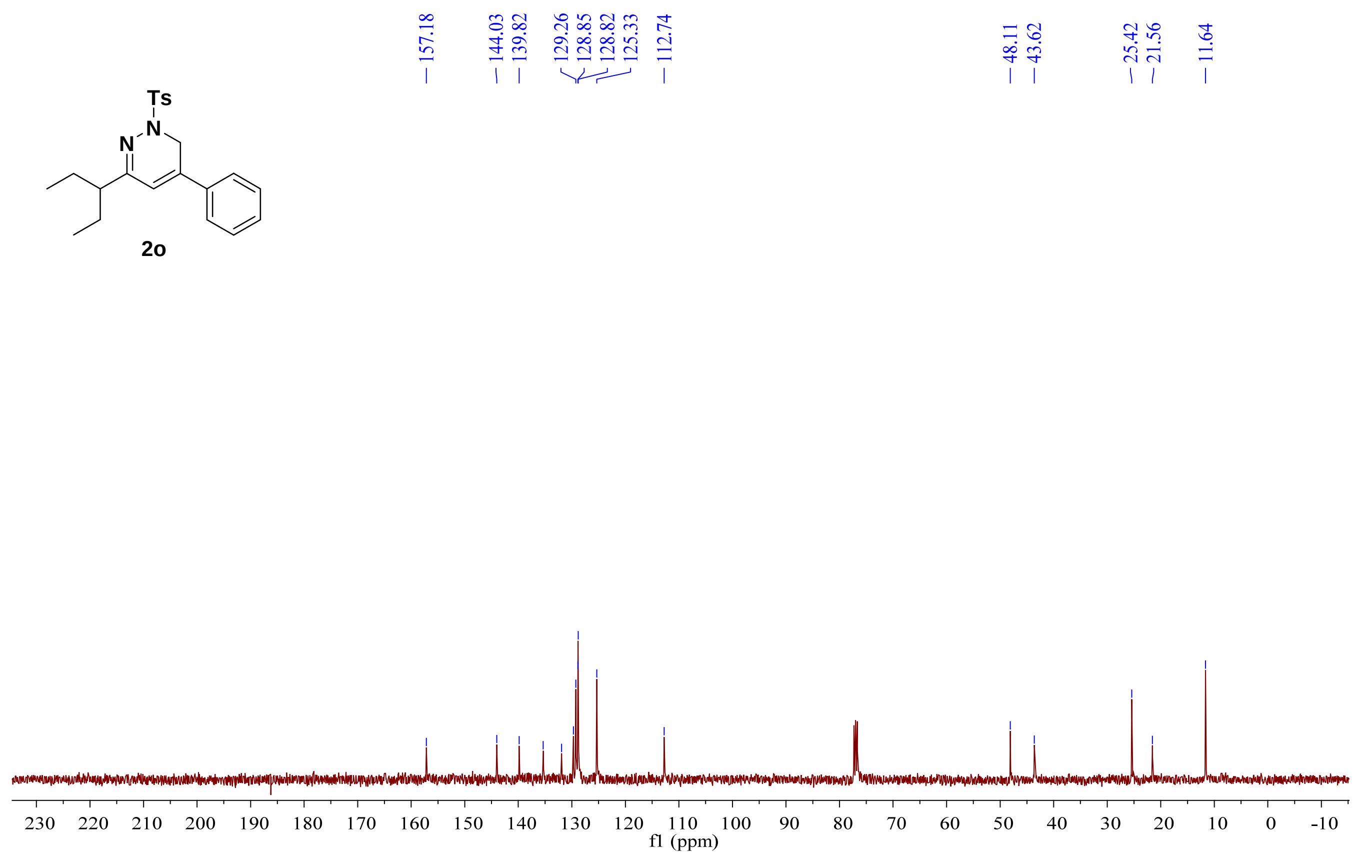

Supplementary Figure 43. ^{1}H NMR (600 MHz, $CDCl_3$) and ^{13}C NMR (100 MHz, $CDCl_3$) spectra of cyclic product **2p**

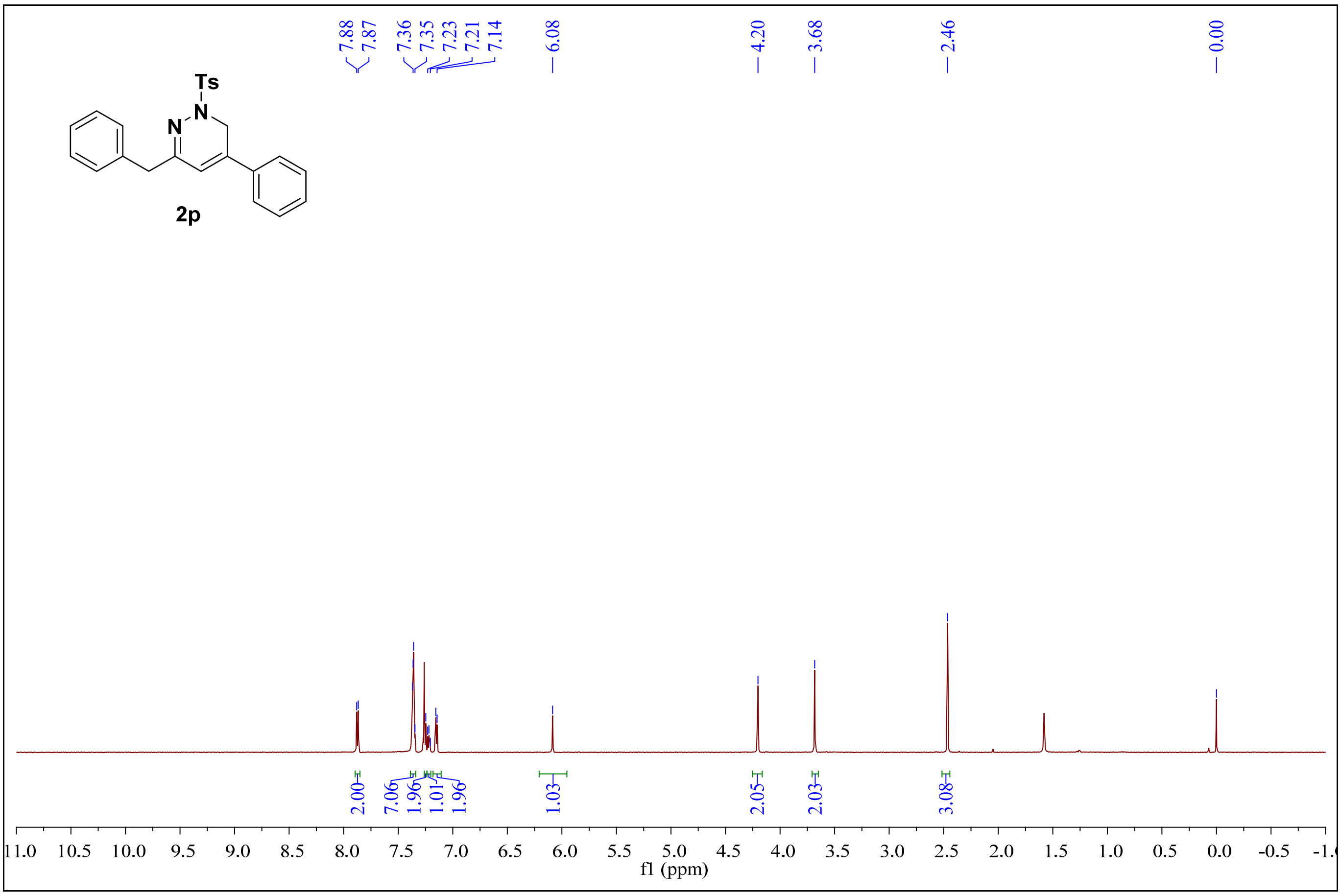

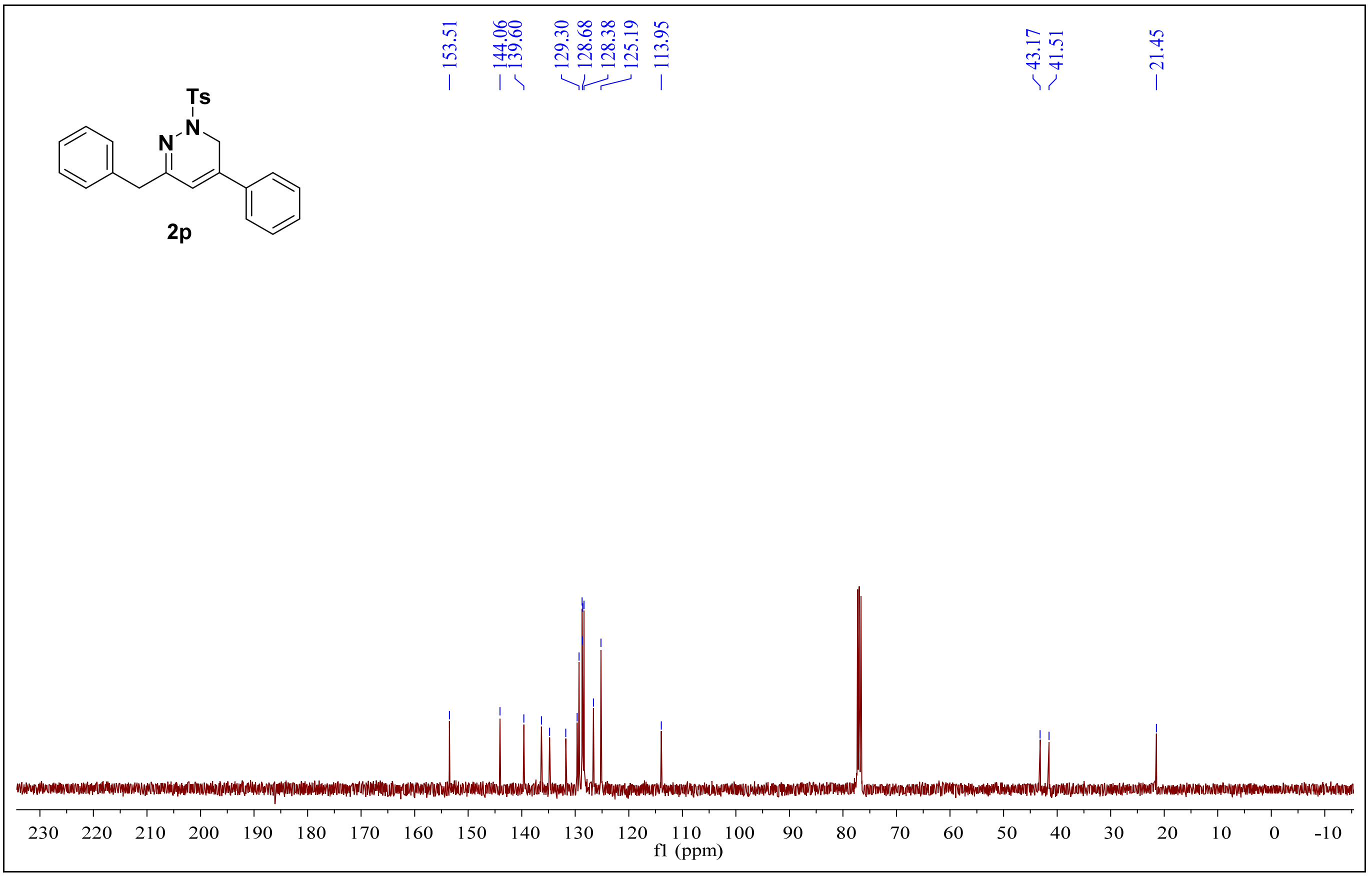

Supplementary Figure 44. **^{1}H NMR (600 MHz, $CDCl_3$) and ^{13}C NMR (100 MHz, $CDCl_3$) spectra of cyclic product 2q**

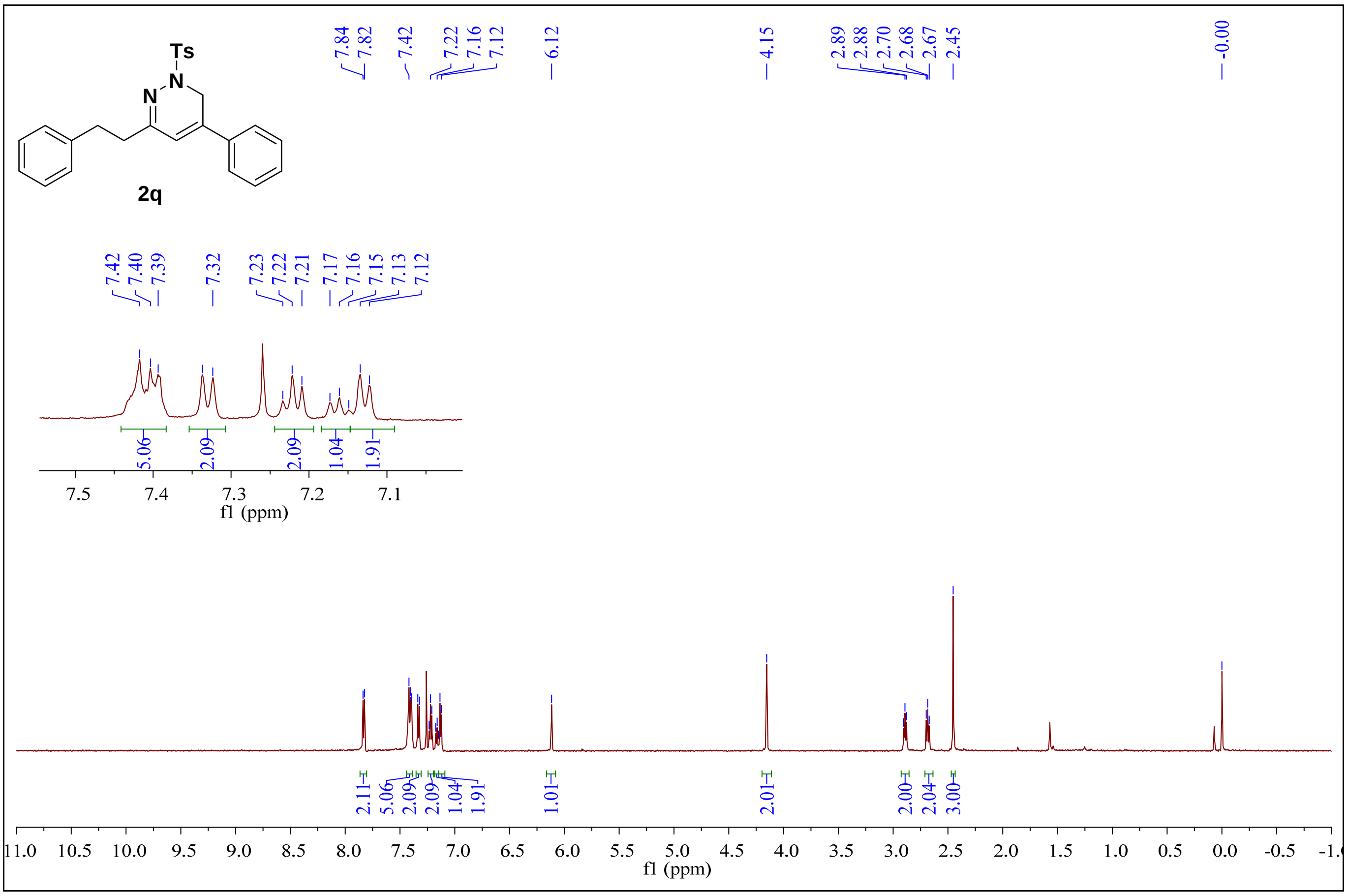

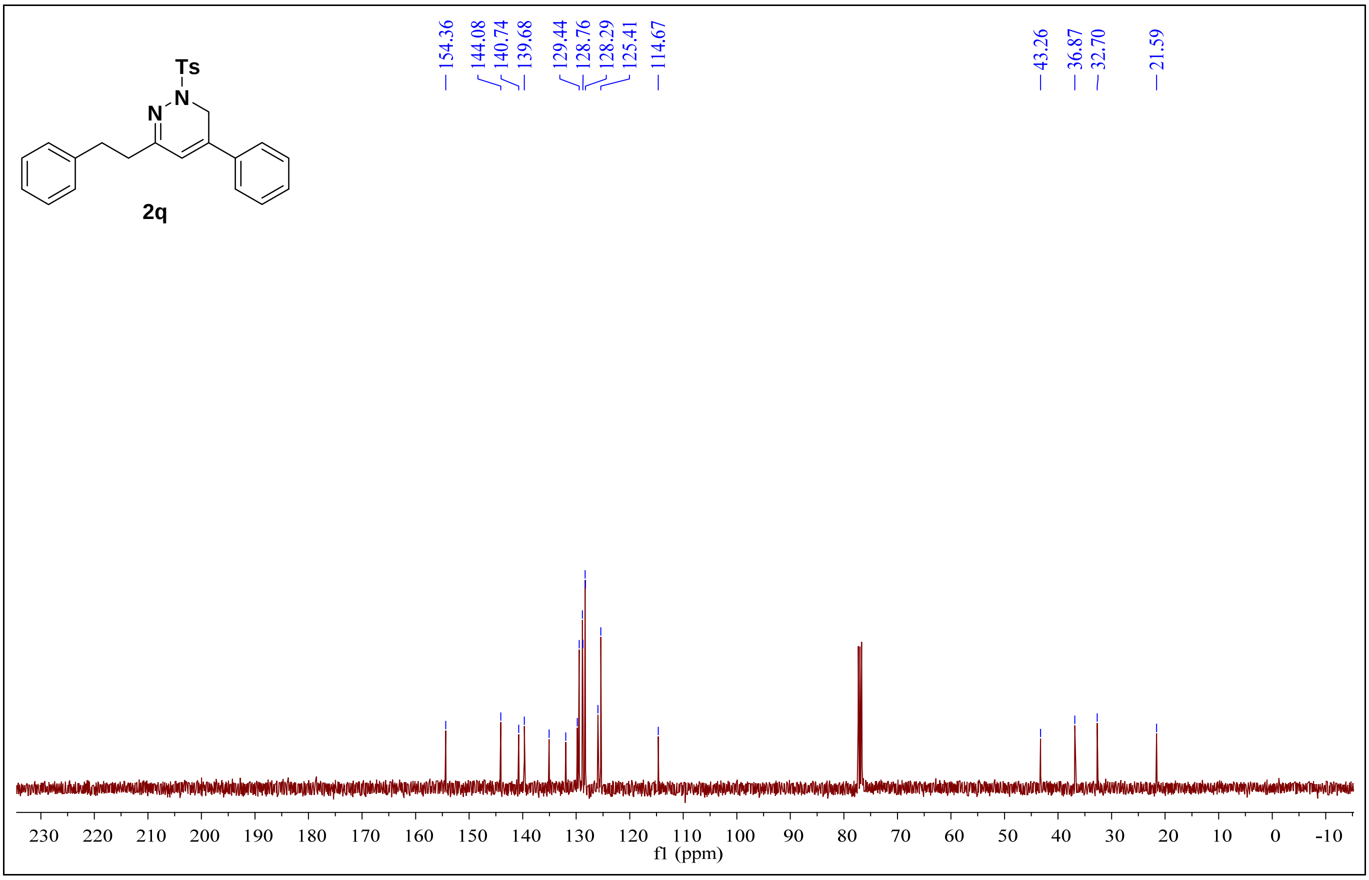

Supplementary Figure 45. ^{1}**H NMR (600 MHz, CDCl$_3$) and** ^{13}**C NMR (100 MHz, CDCl$_3$) spectra of cyclic product 2r**

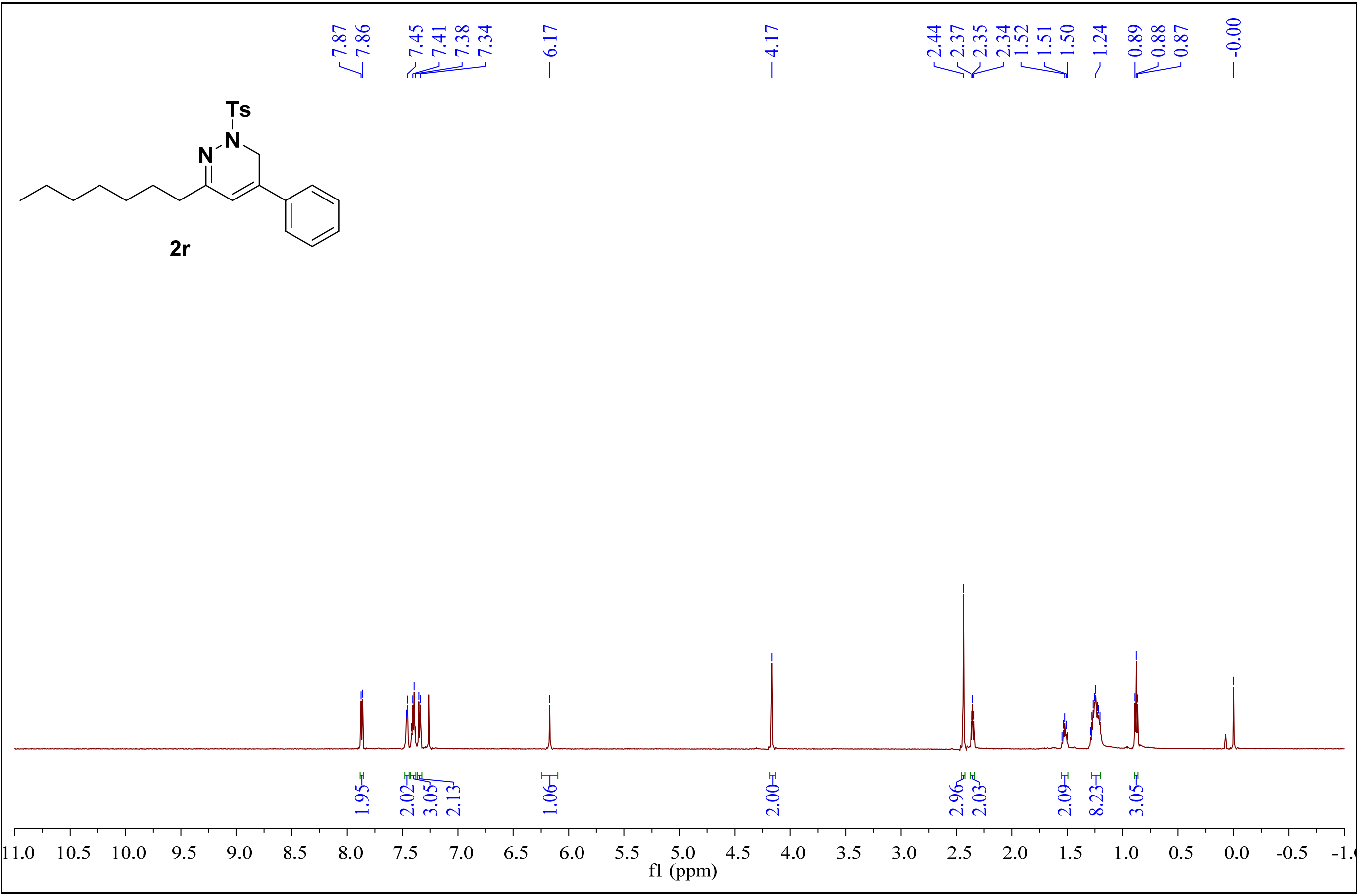

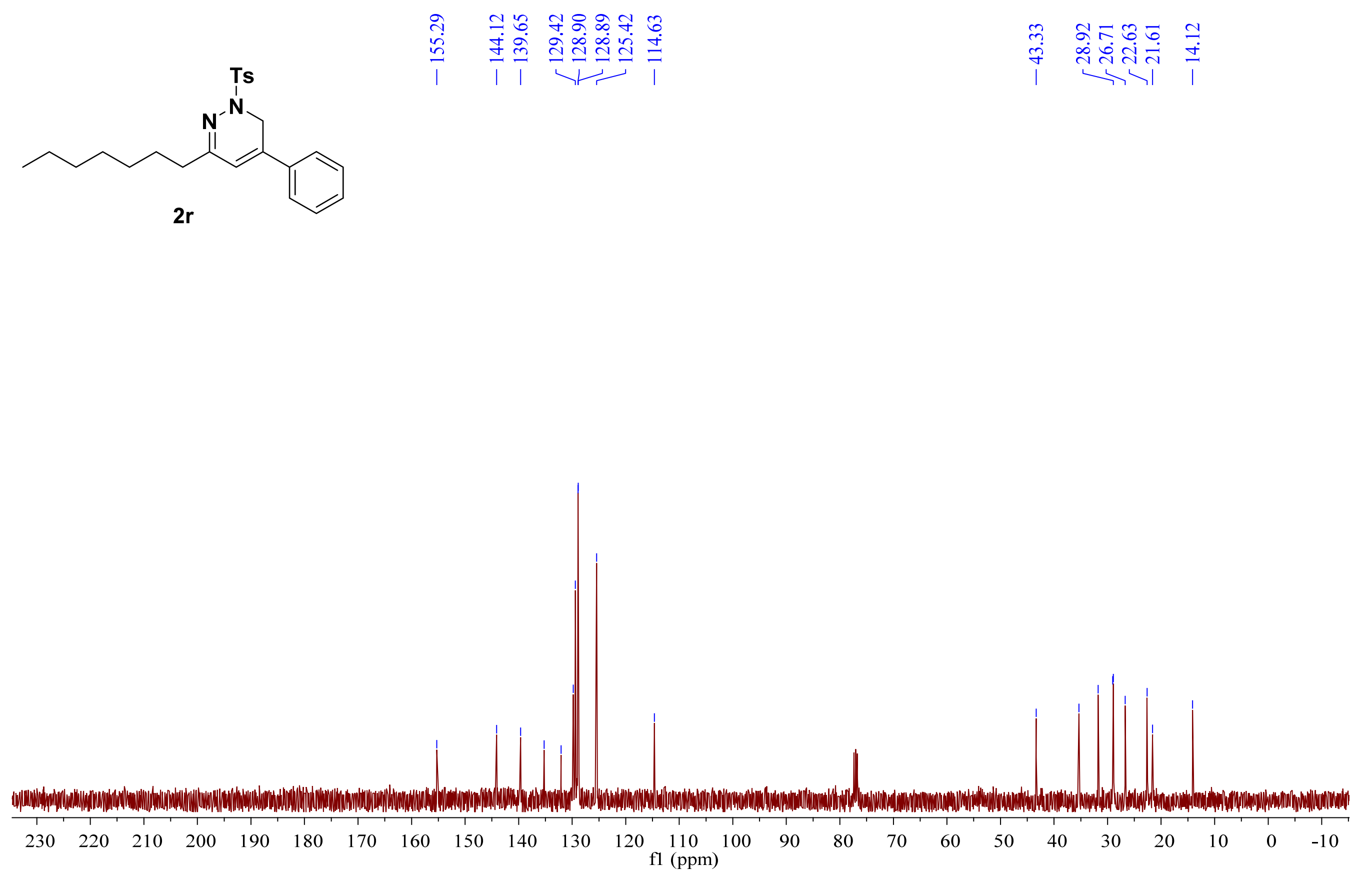

Supplementary Figure 46. ^{1}H NMR (400 MHz, $CDCl_3$) and ^{13}C NMR (100 MHz, $CDCl_3$) spectra of cyclic product **2s**

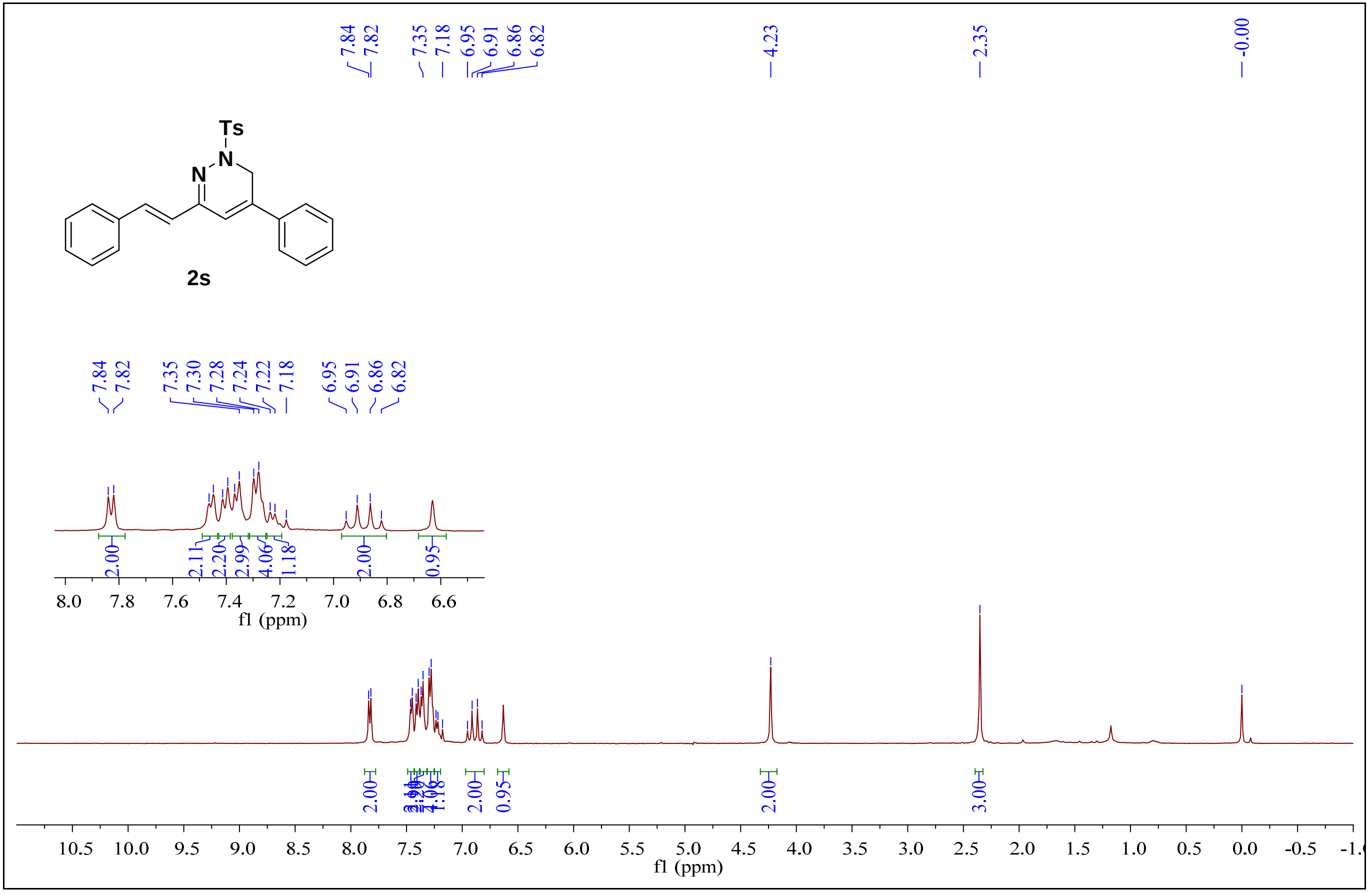

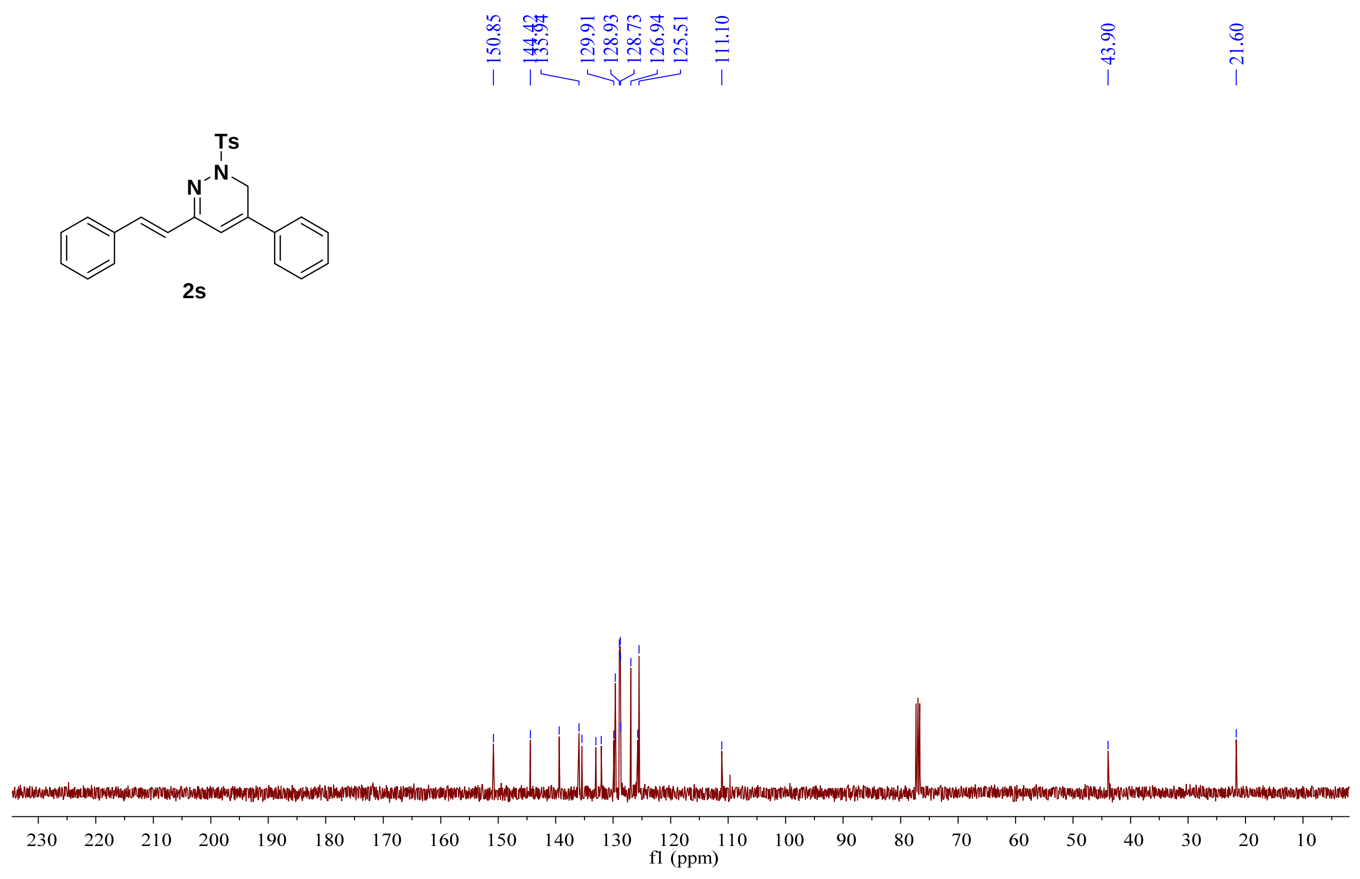

Supplementary Figure 47. ^{1}H NMR (600 MHz, $CDCl_3$) and ^{13}C NMR (100 MHz, $CDCl_3$) spectra of cyclic product 2t

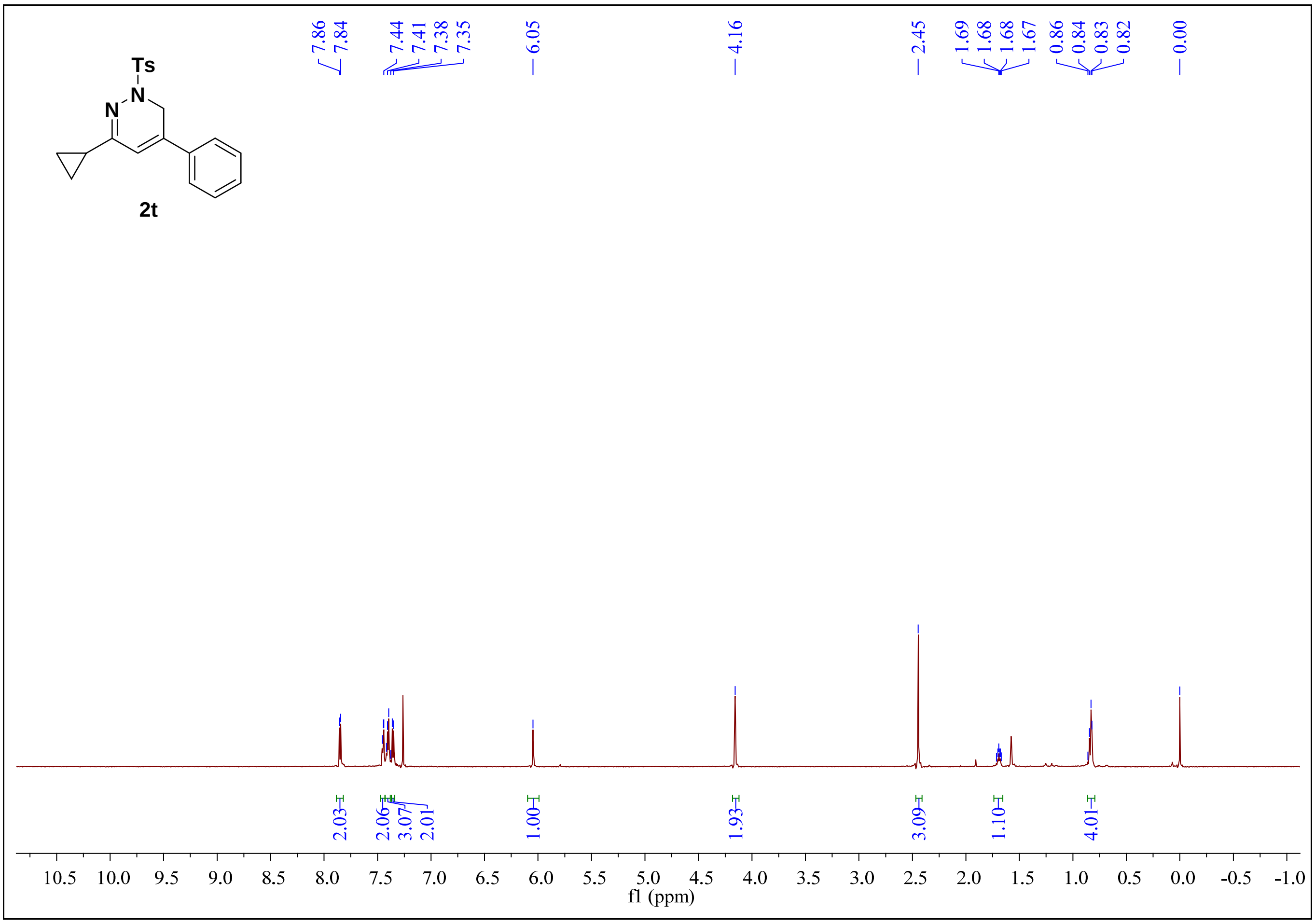

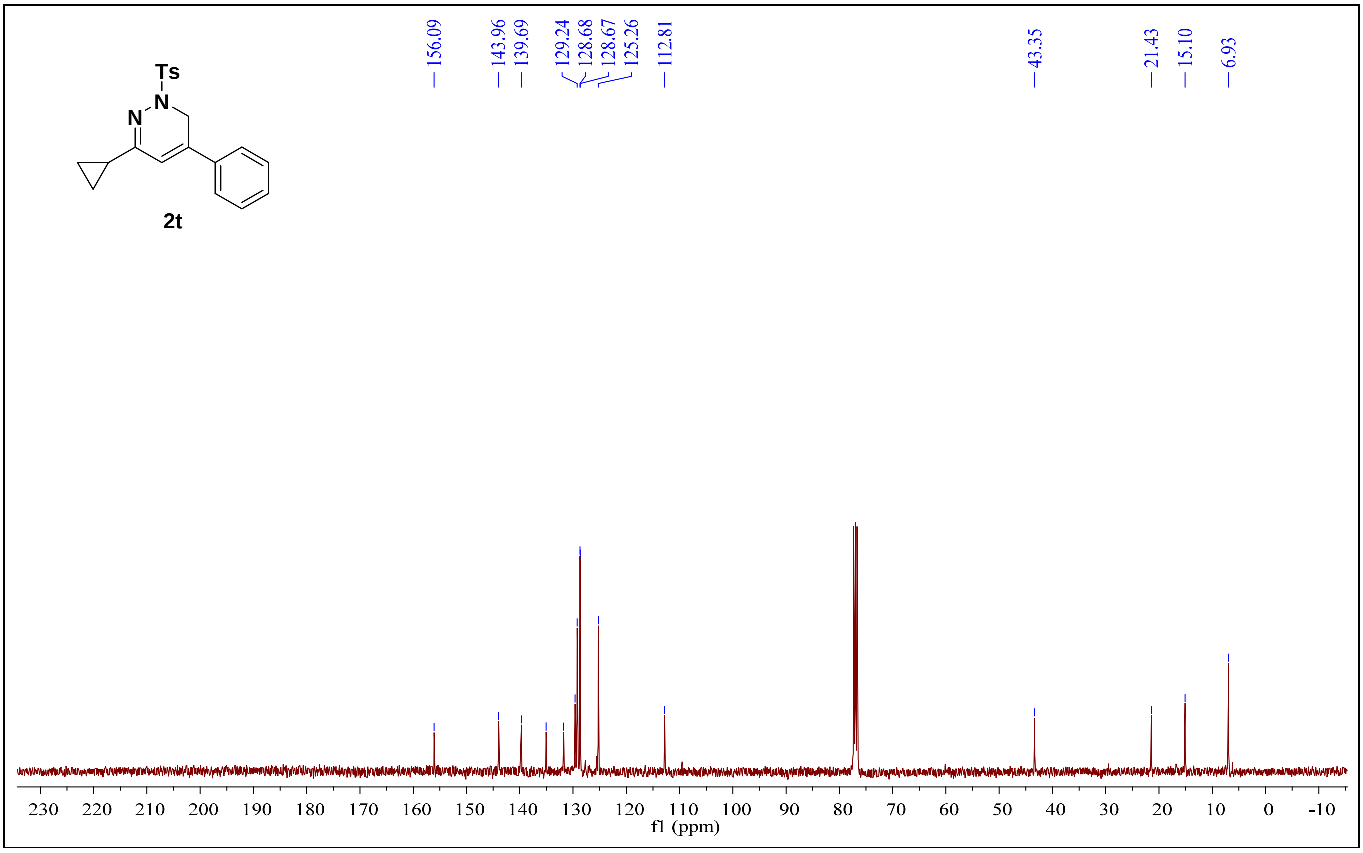

Supplementary Figure 48. **^{1}H NMR (600 MHz, $CDCl_3$) and ^{13}C NMR (100 MHz, $CDCl_3$) spectra of cyclic product 2u**

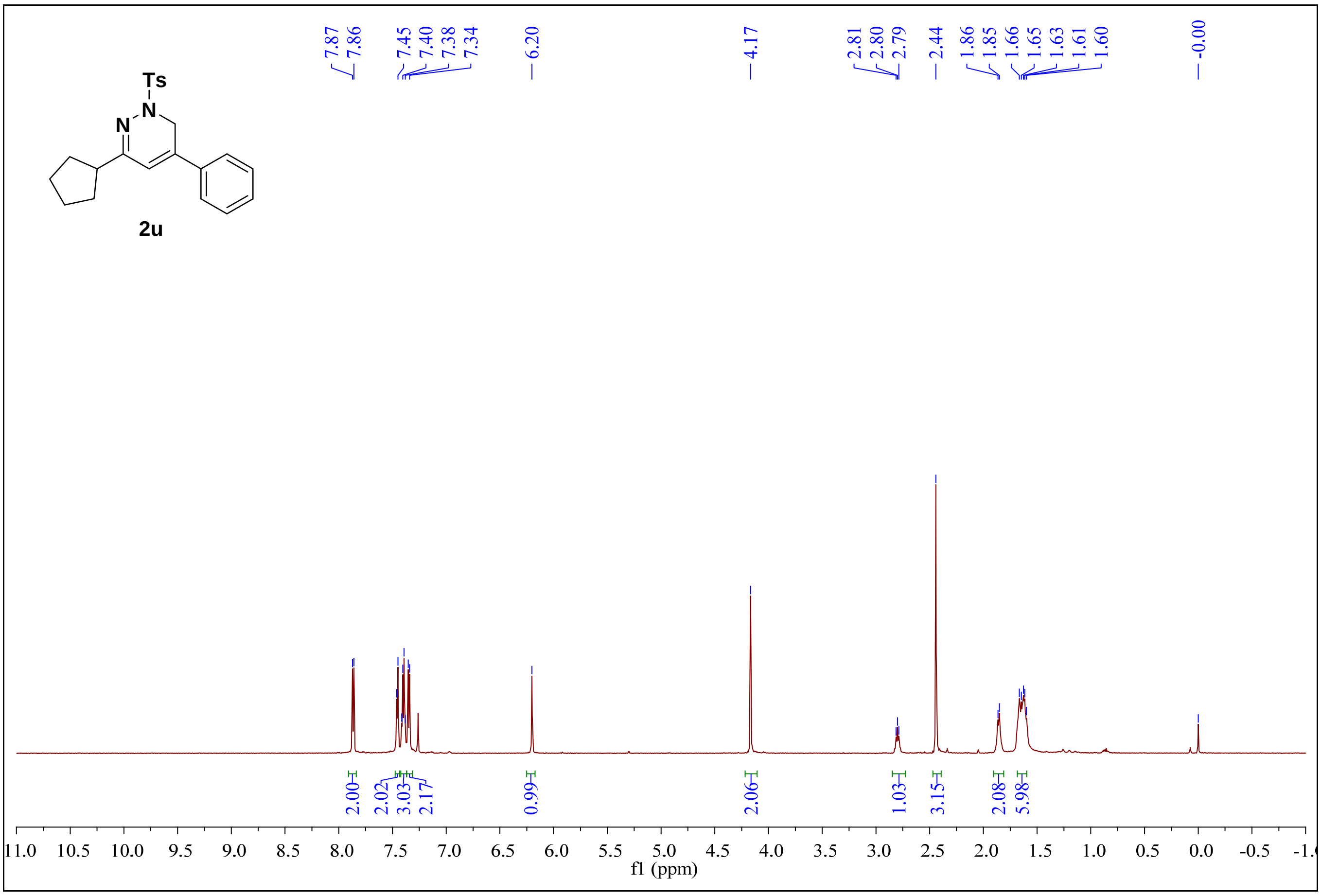

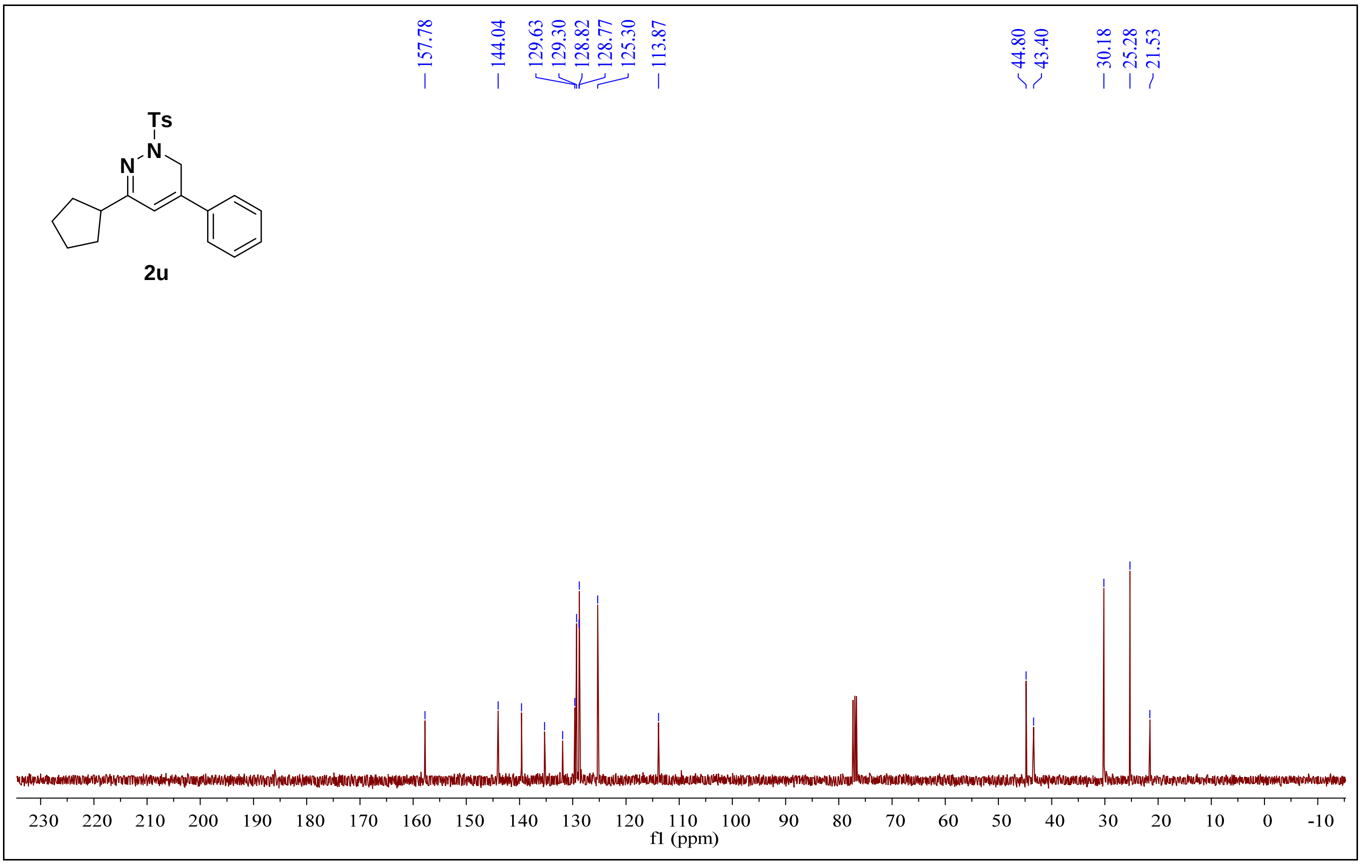

Supplementary Figure 49. ^{1}**H NMR (600 MHz, CDCl$_3$) and** ^{13}**C NMR (100 MHz, CDCl$_3$) spectra of cyclic product 2v**

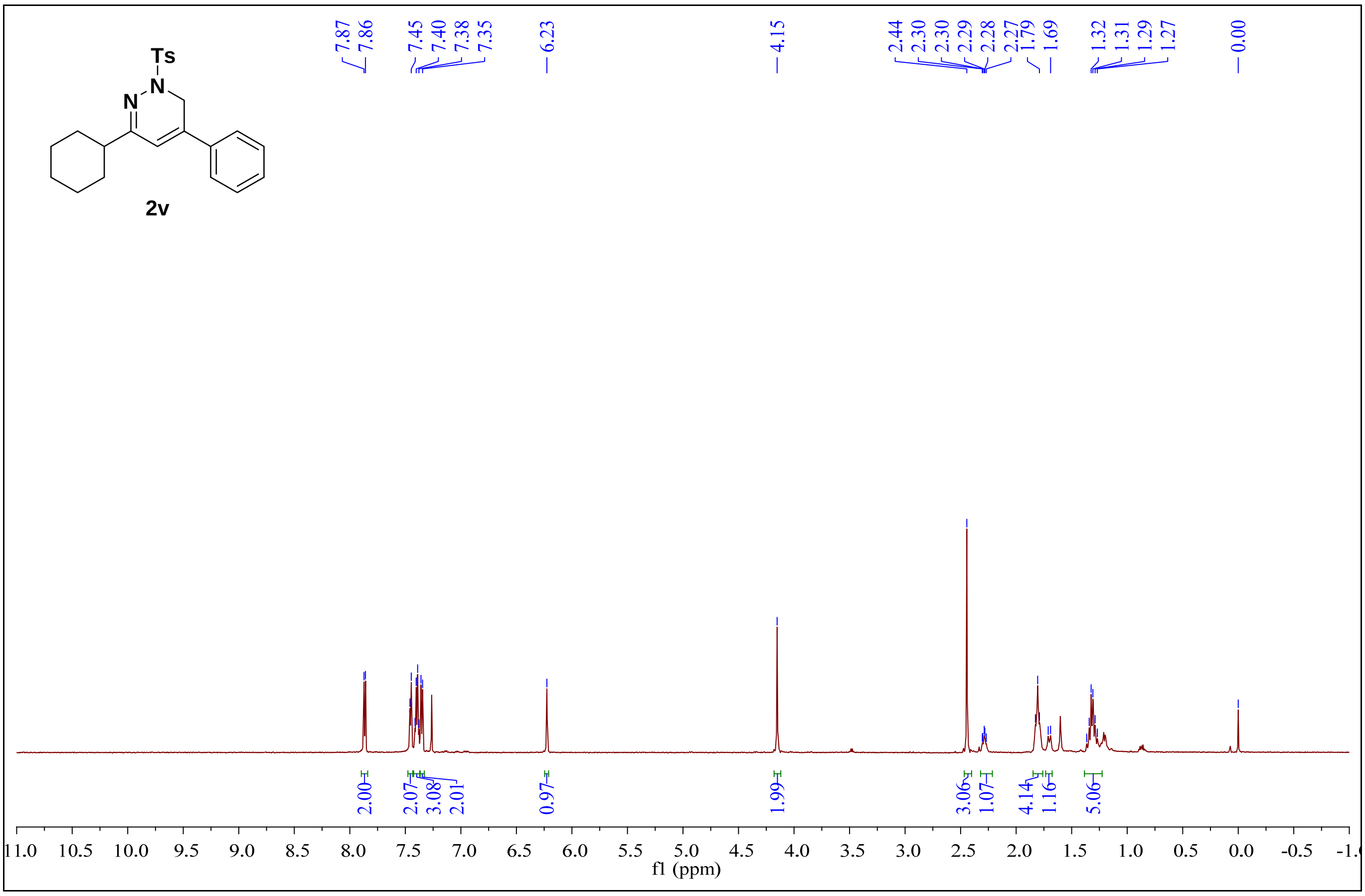

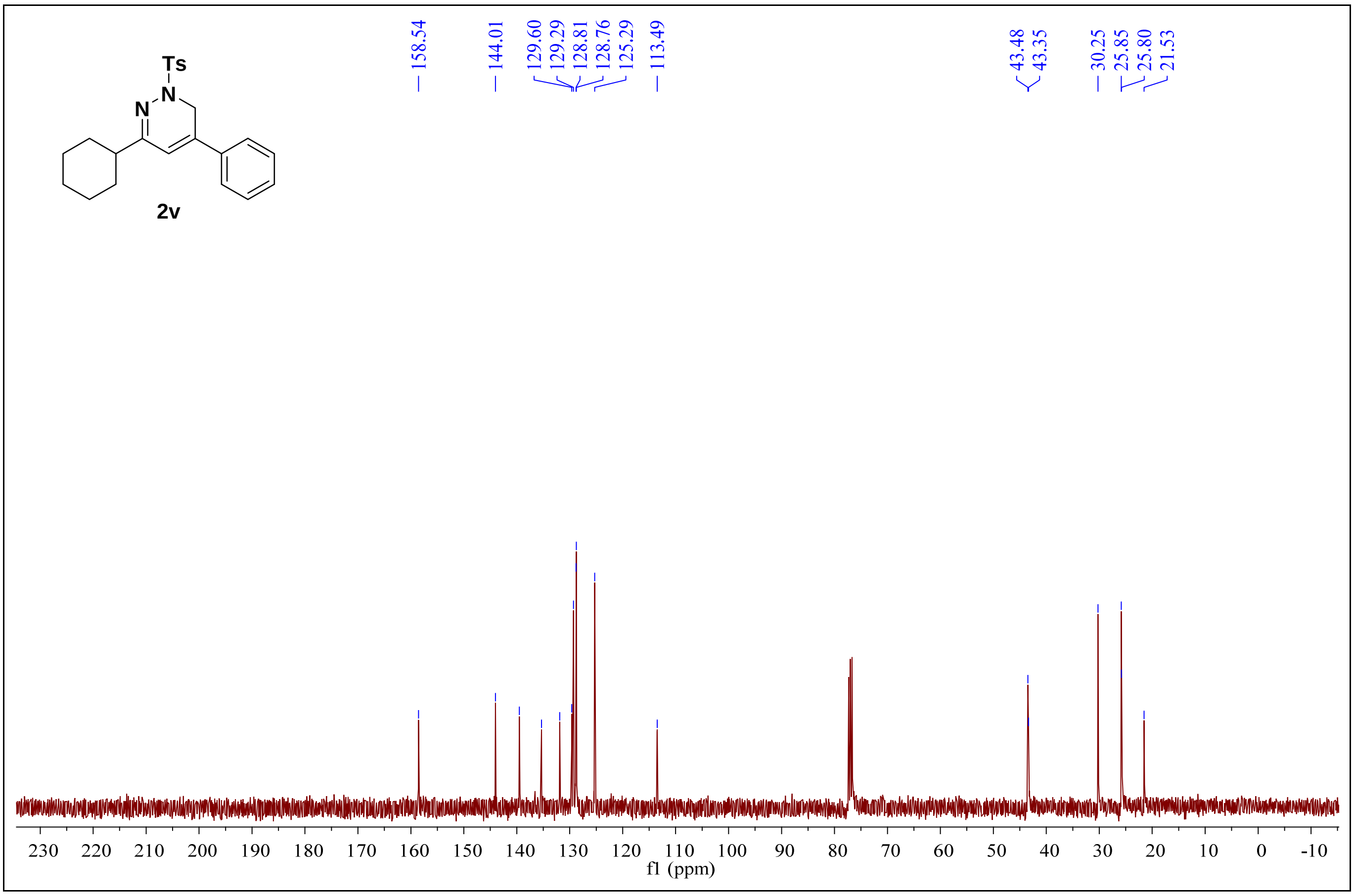

Supplementary Figure 50. ^{1}H NMR (400 MHz, $CDCl_3$) and ^{13}C NMR (100 MHz, $CDCl_3$) spectra of cyclic product 3

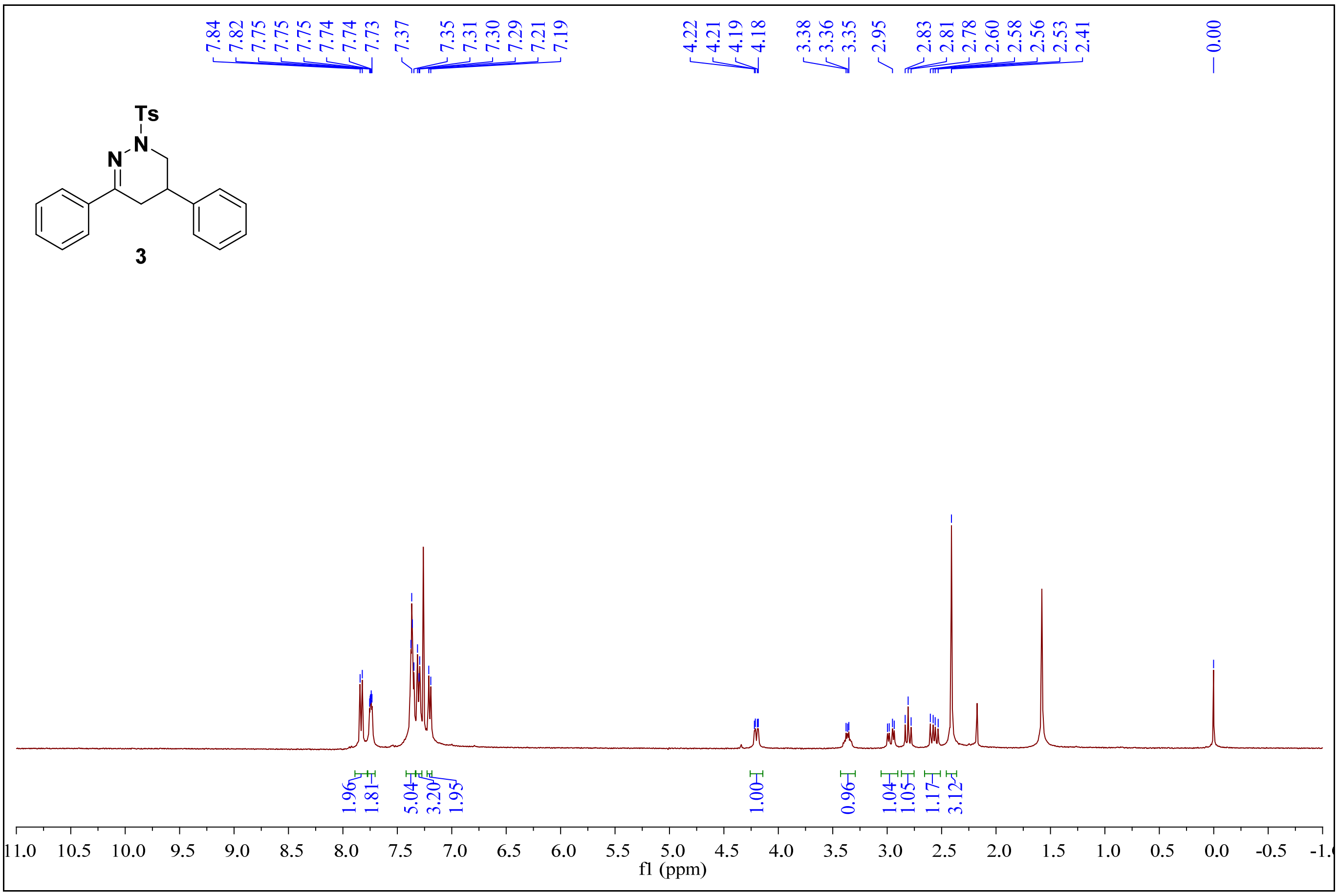

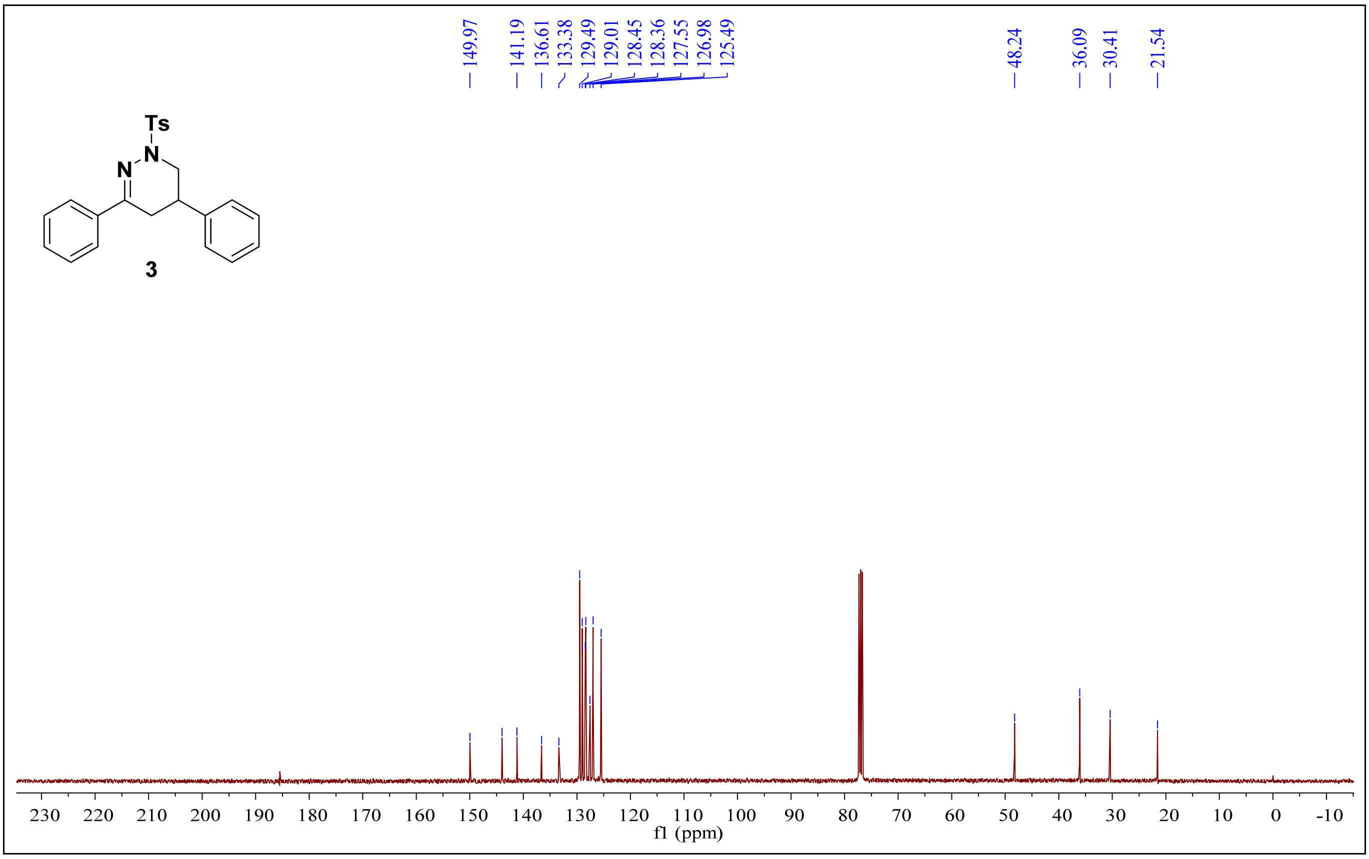

Supplementary Figure 51. ^{1}H NMR (400 MHz, $CDCl_3$) and ^{13}C NMR (100 MHz, $CDCl_3$) spectra of cyclic product **4**

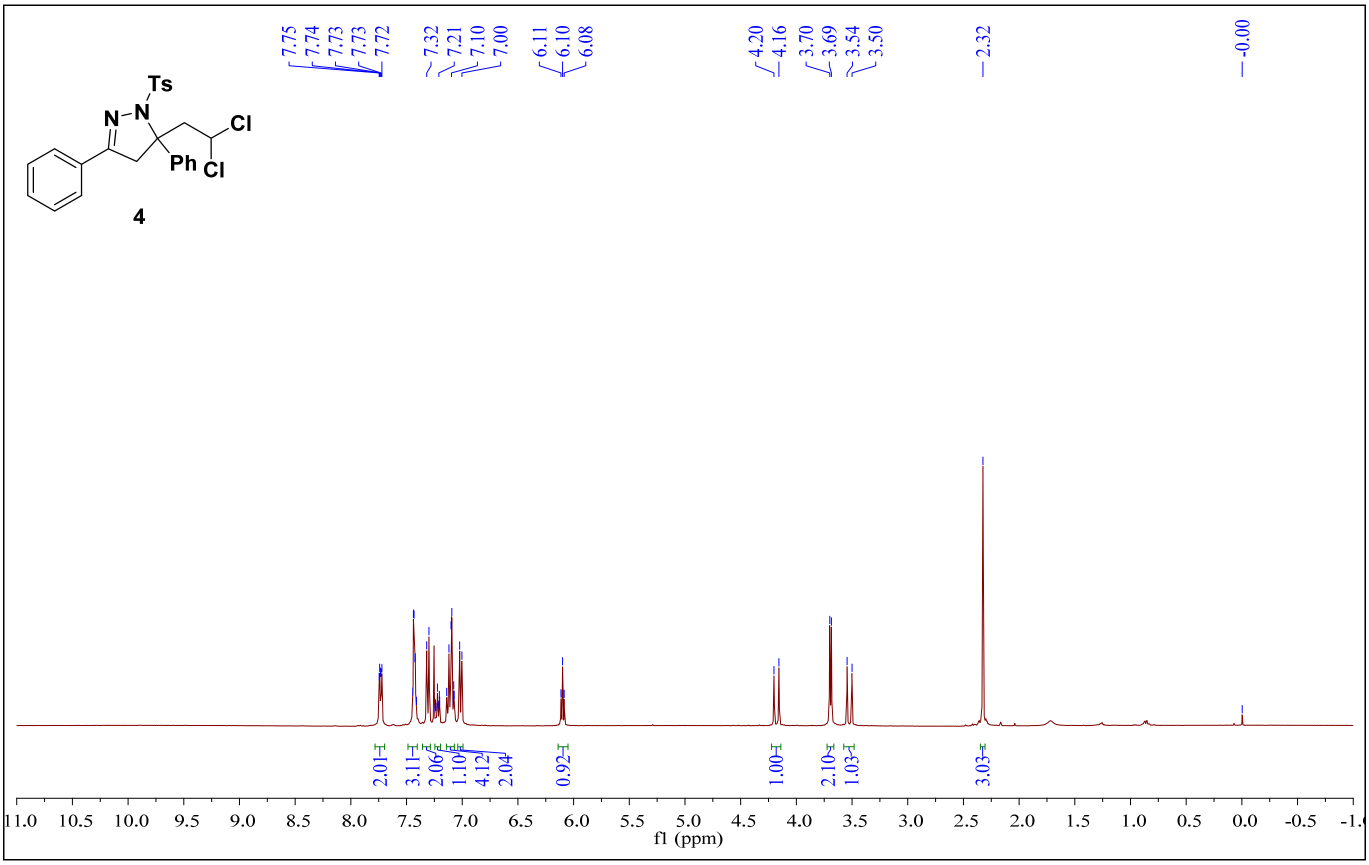

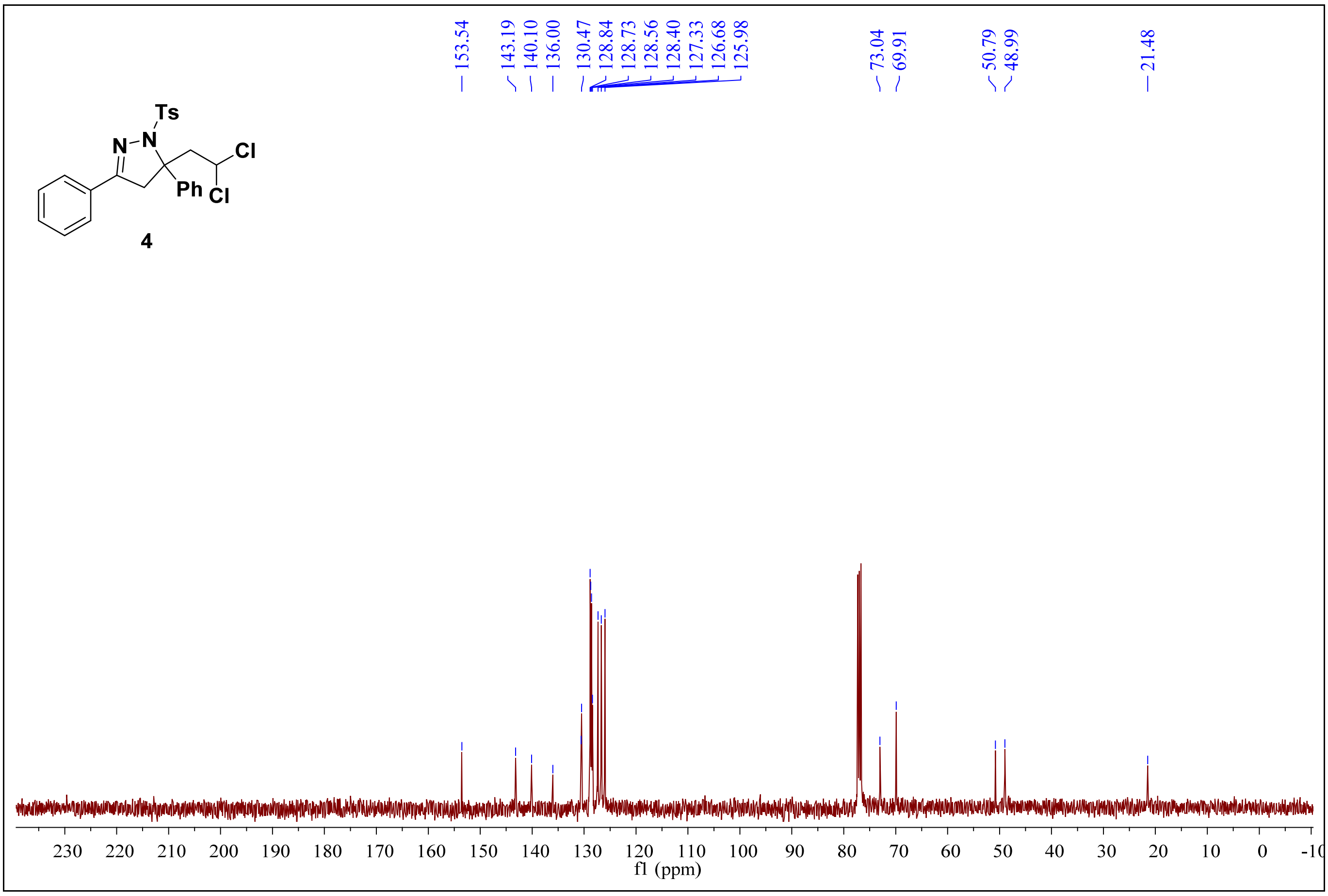

Supplementary Figure 52. ^{1}H **NMR (600 MHz, CDCl$_3$) and** ^{13}C **NMR (100 MHz, CDCl$_3$) spectra of cyclic product 6a**

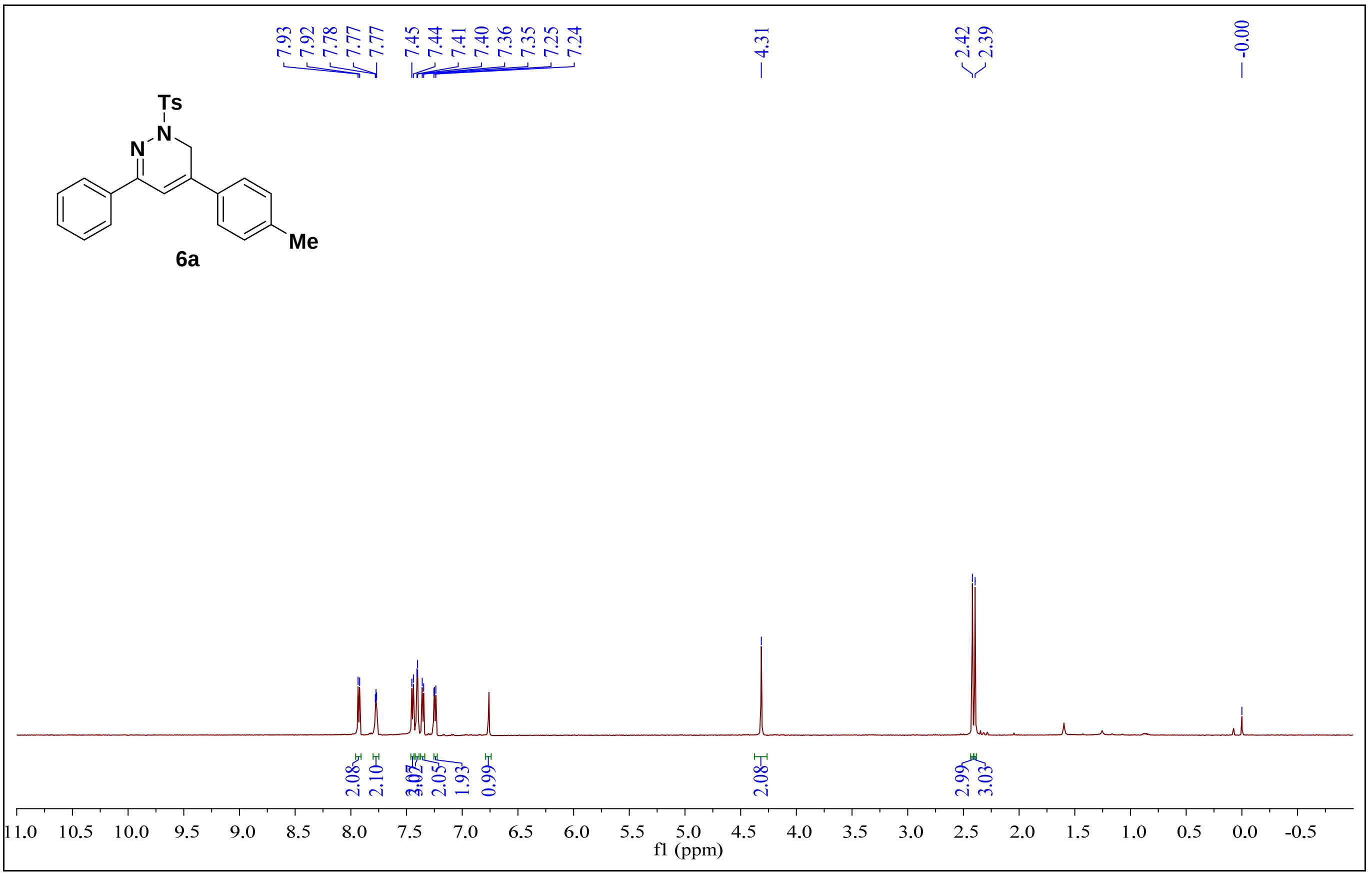

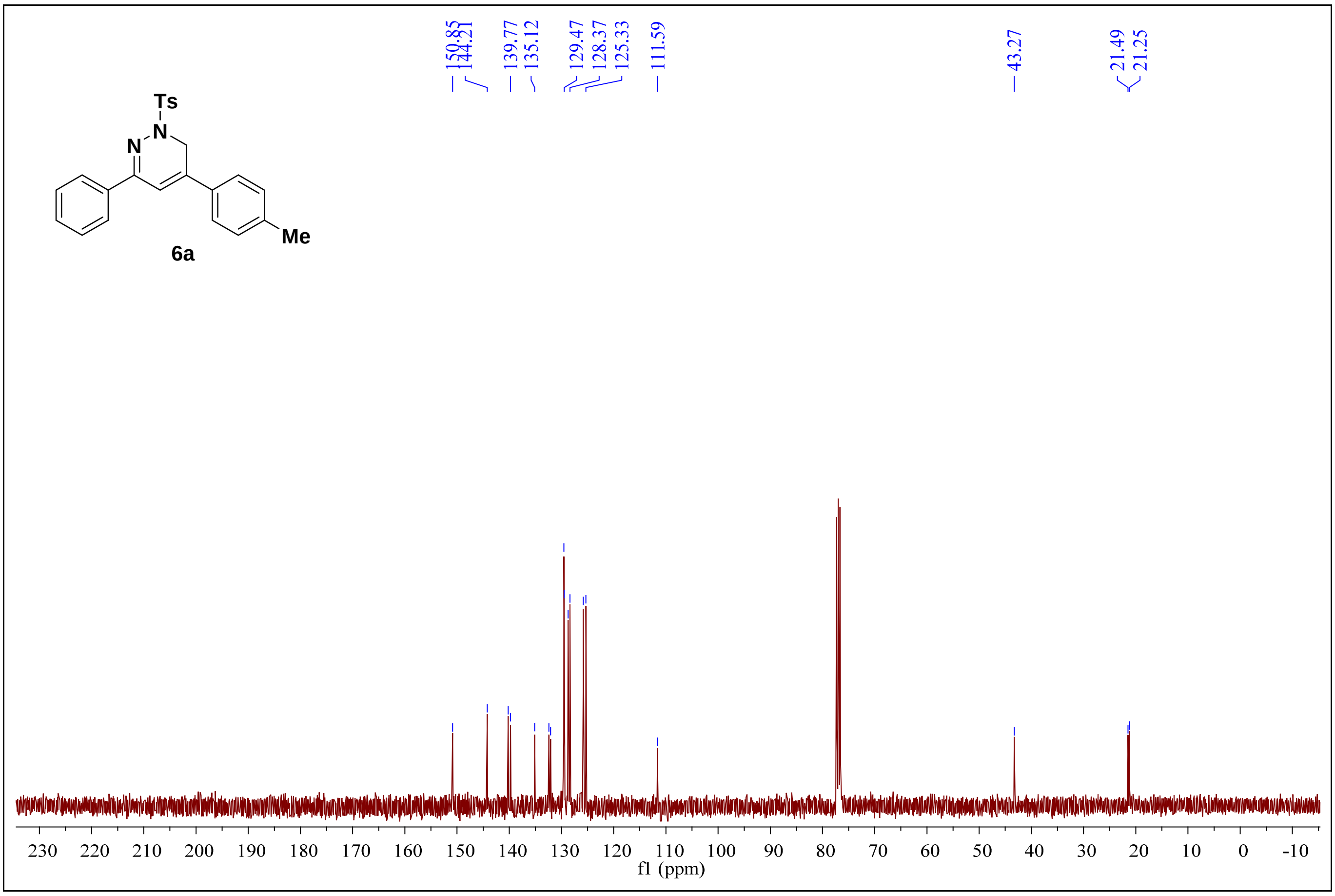

Supplementary Figure 53. ^{1}H NMR (600 MHz, $CDCl_3$) and ^{13}C NMR (100 MHz, $CDCl_3$) spectra of cyclic product **6b**

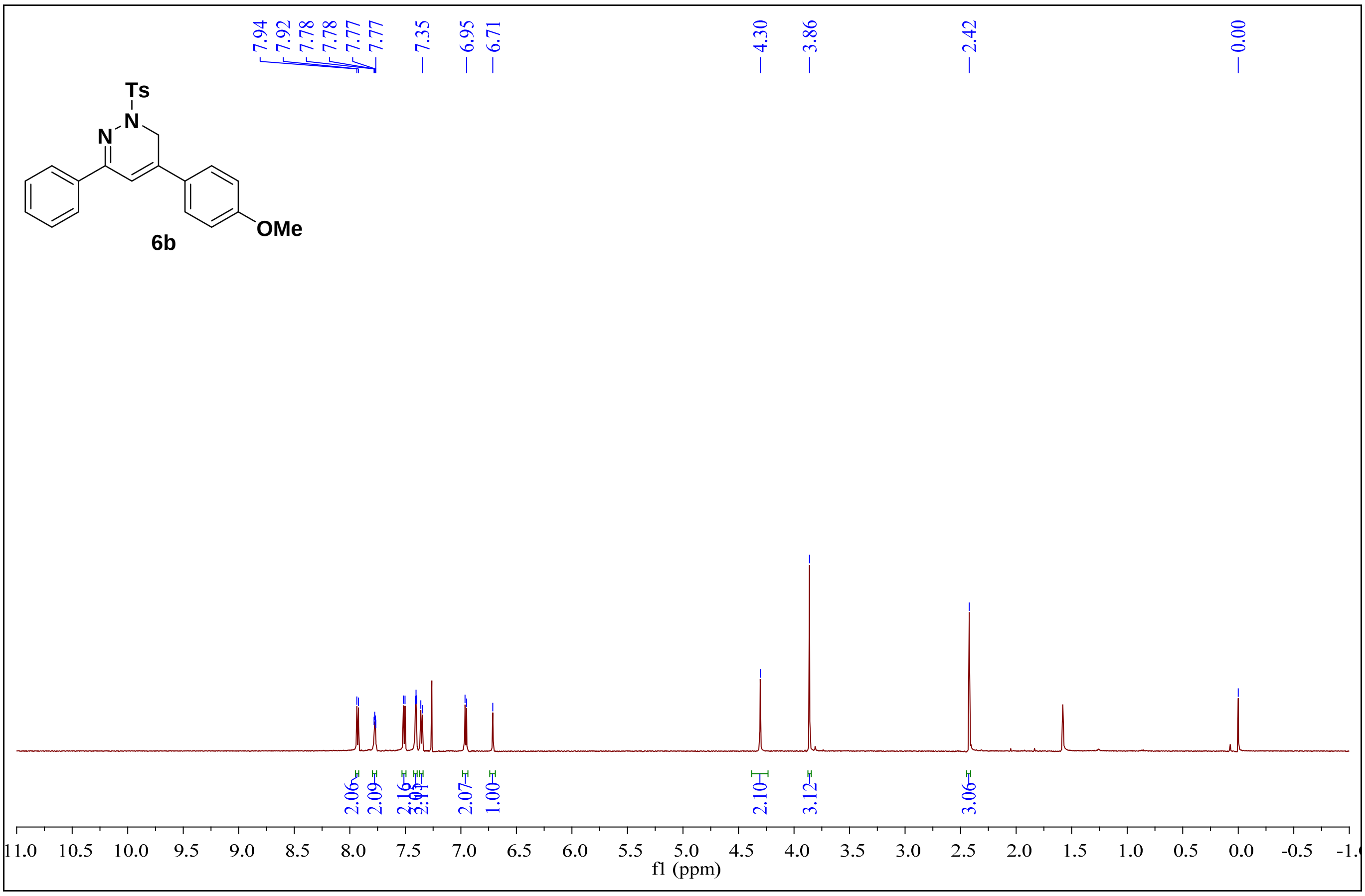

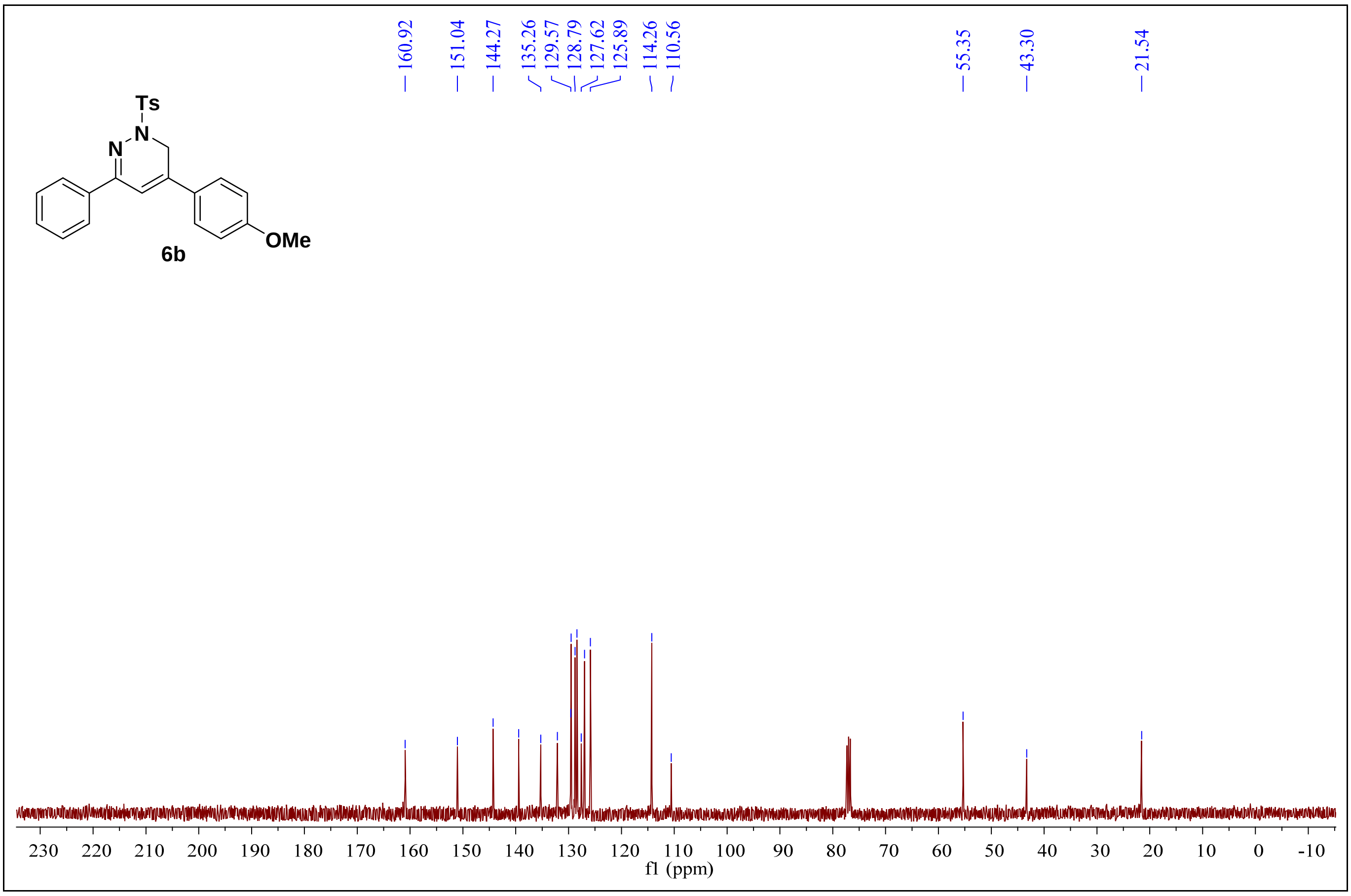

Supplementary Figure 54. ^{1}H NMR (600 MHz, $CDCl_3$) and ^{13}C NMR (100 MHz, $CDCl_3$) spectra of cyclic product **6c**

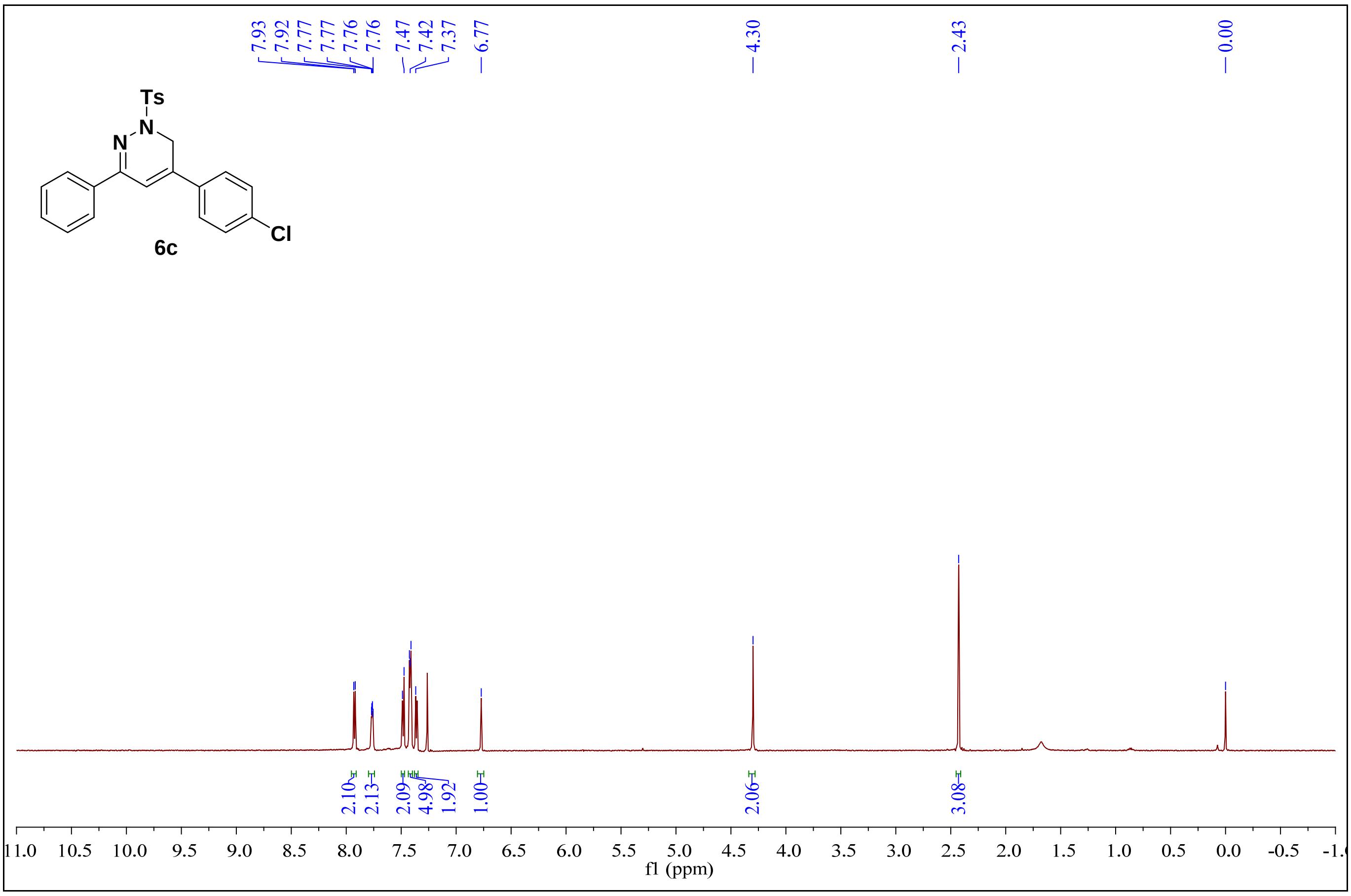

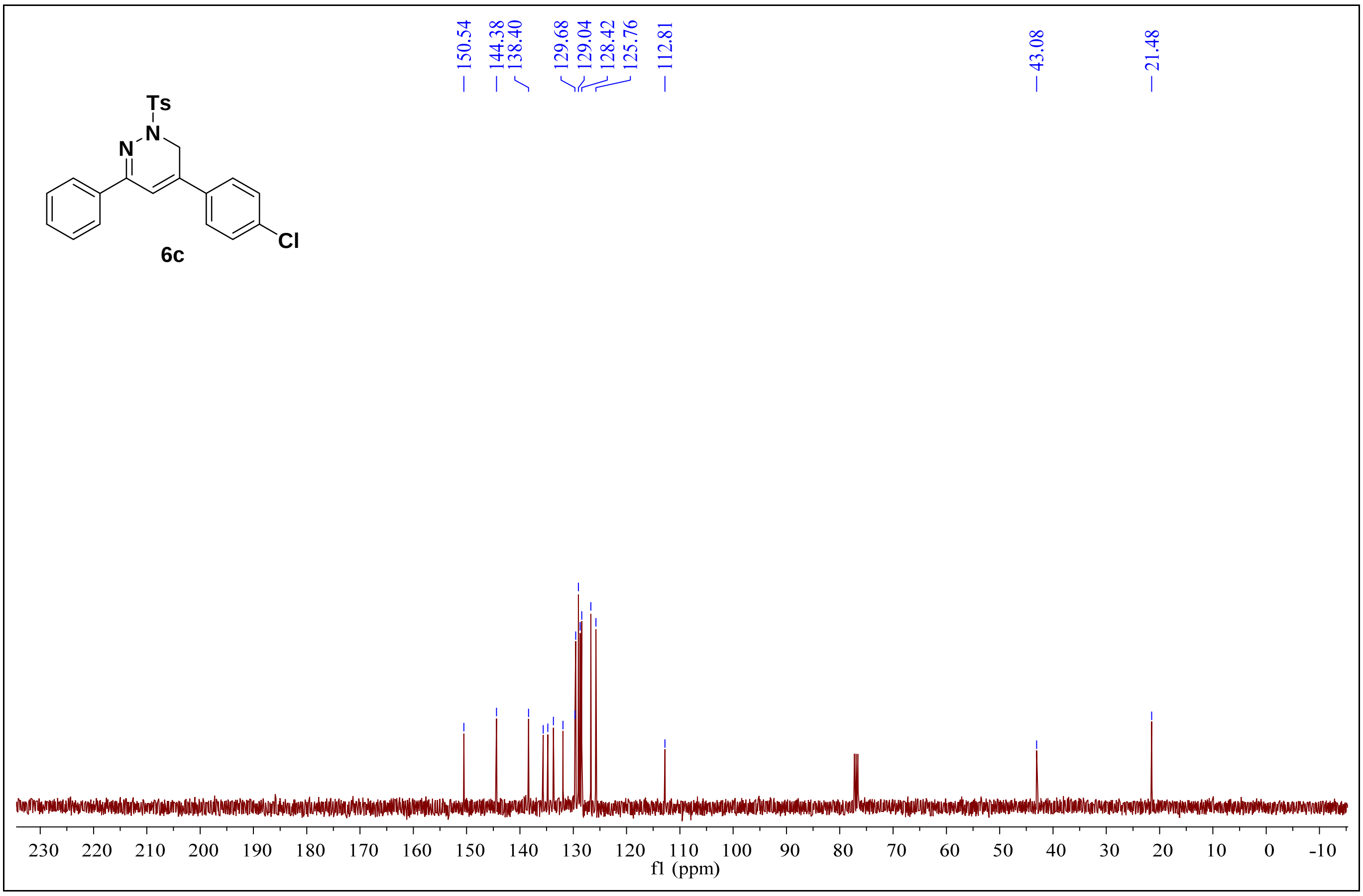

Supplementary Figure 55. ^{1}H **NMR (600 MHz,** $CDCl_3$**) and** ^{13}C **NMR (100 MHz,** $CDCl_3$**) spectra of cyclic product 6d**

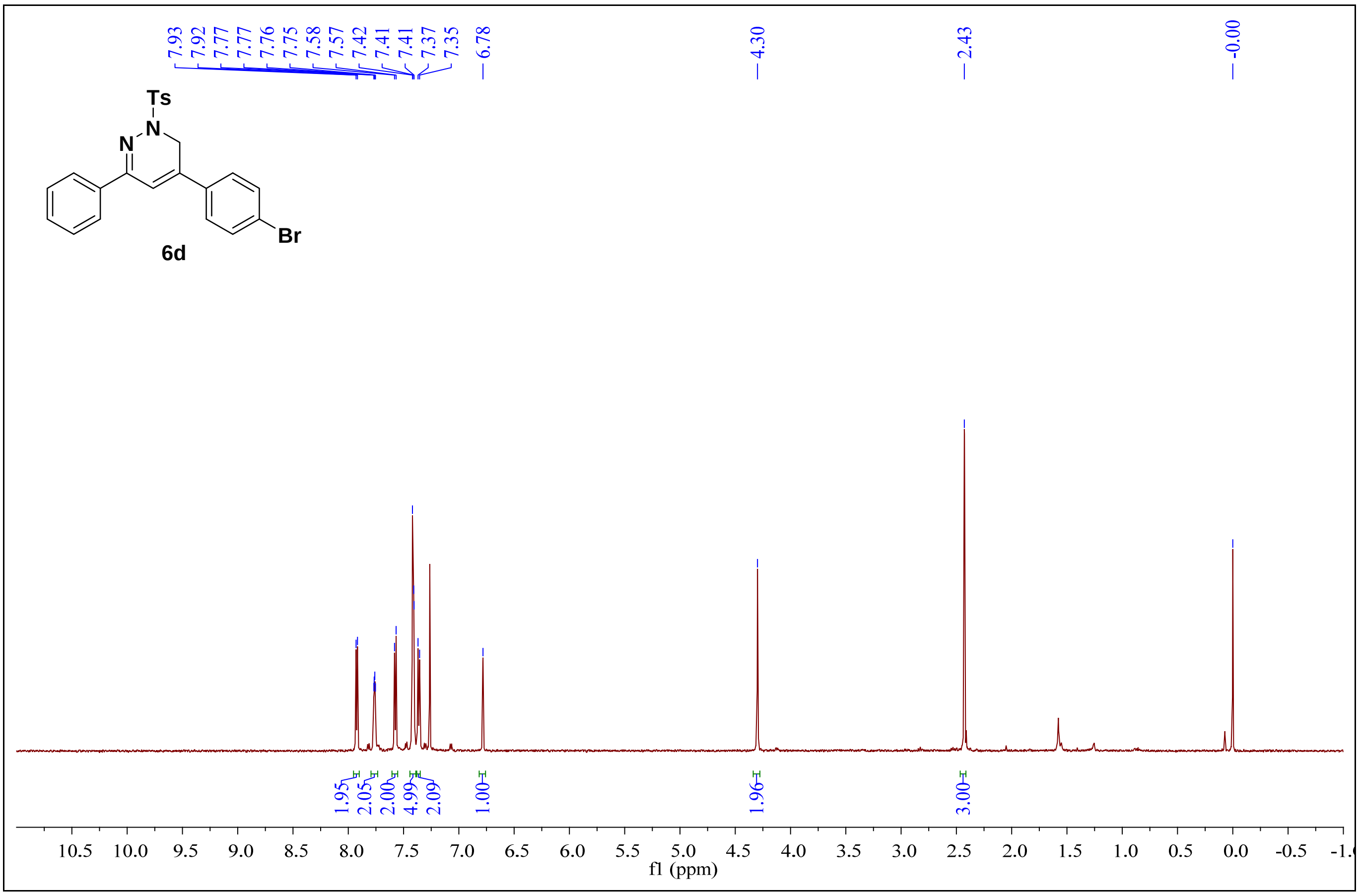

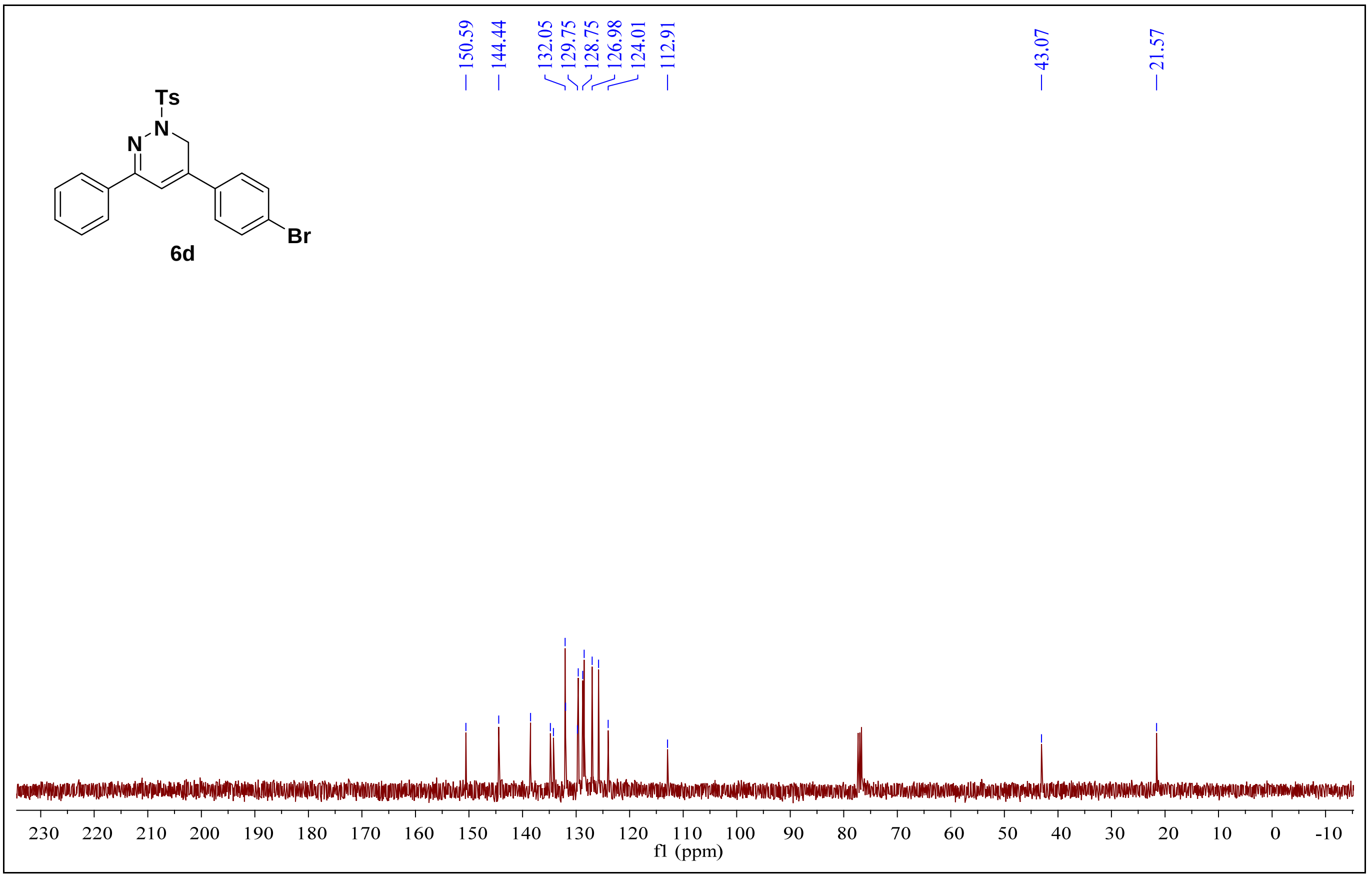

Supplementary Figure 56. ^{1}**H NMR (600 MHz, CDCl$_3$),** ^{13}**C NMR (100 MHz, CDCl$_3$) and** ^{19}**F (376 MHz, CDCl$_3$) spectra of cyclic product 6e**

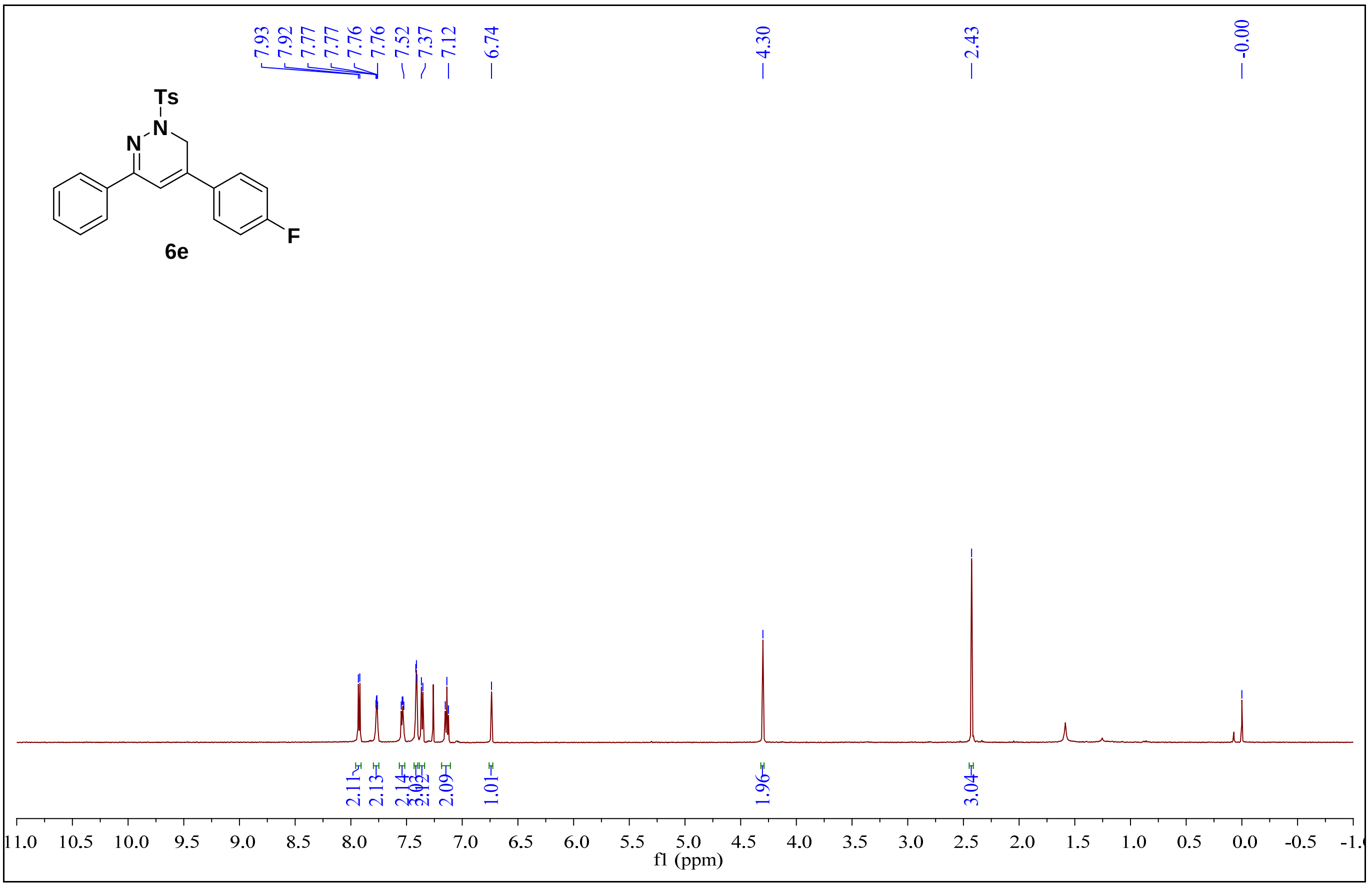

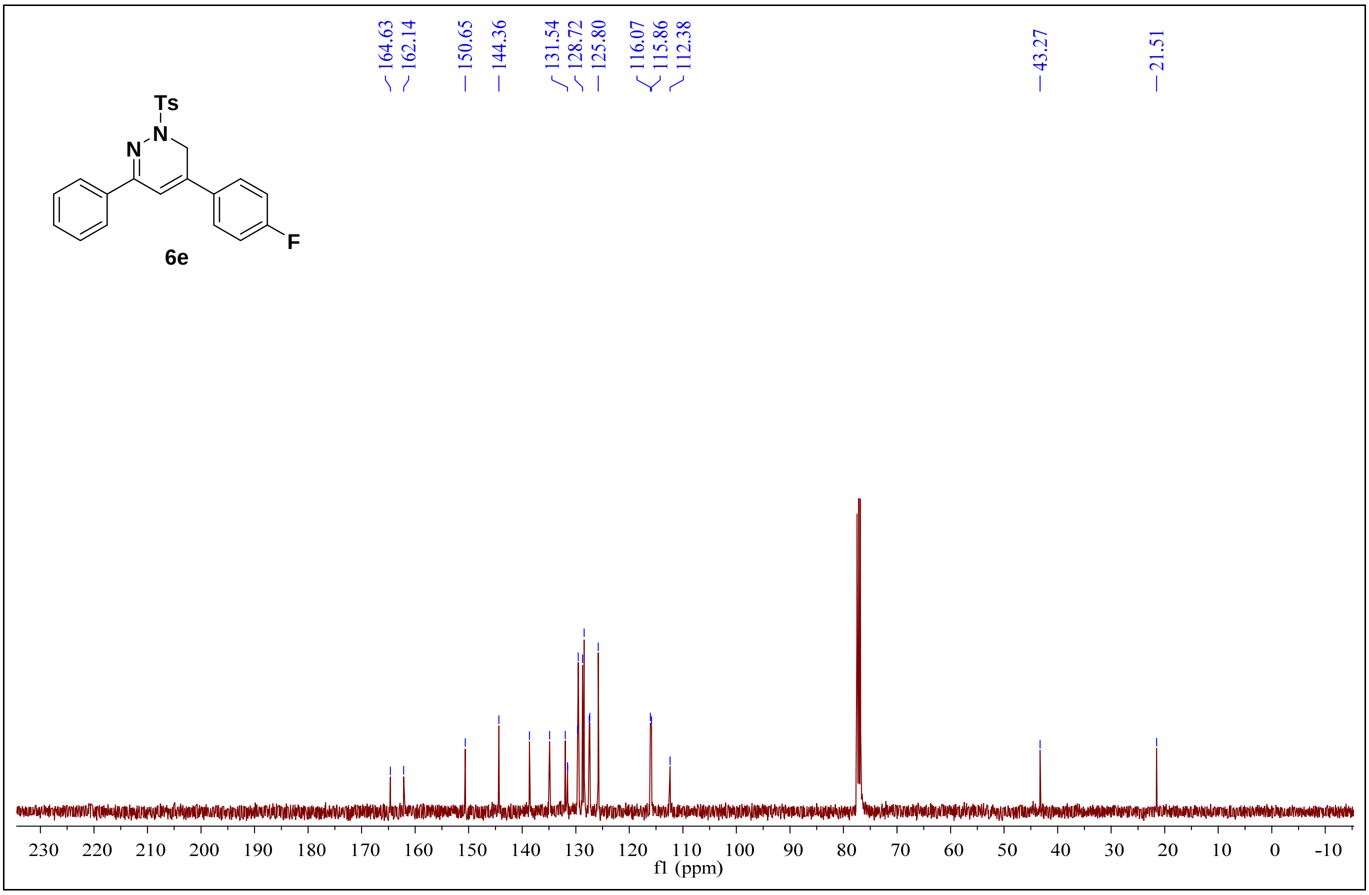

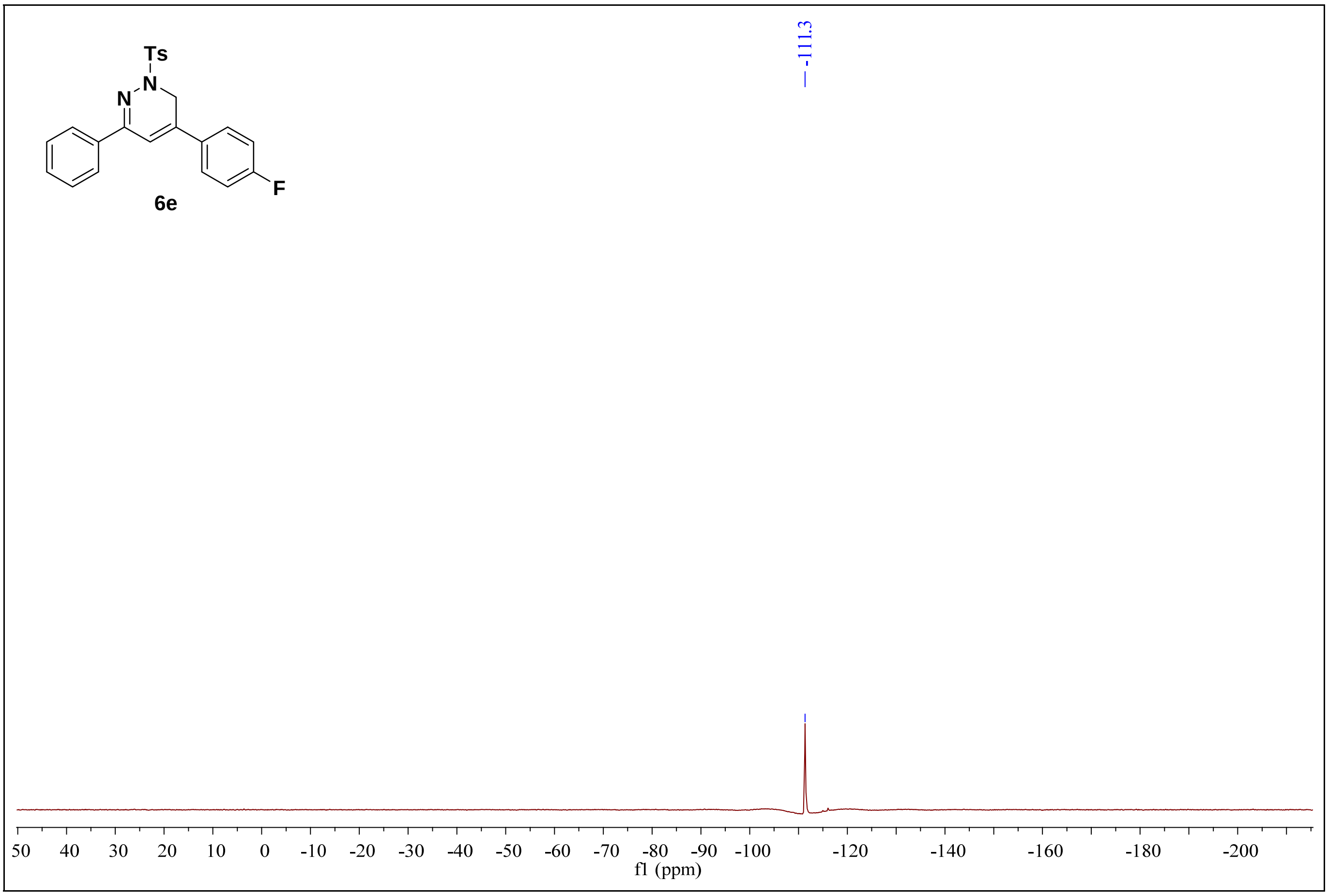
Ts
N
N
F
6e
-111.3
50
40
30
20
10
0
-10
-20
-30
-40
-50
-60
-70
-80
-90
-100
-120
-140
-160
-180
-200
f1 (ppm)

Supplementary Figure 57. ^{1}**H NMR (600 MHz, CDCl$_3$) and** ^{13}**C NMR (100 MHz, CDCl$_3$) spectra of cyclic product 6f**

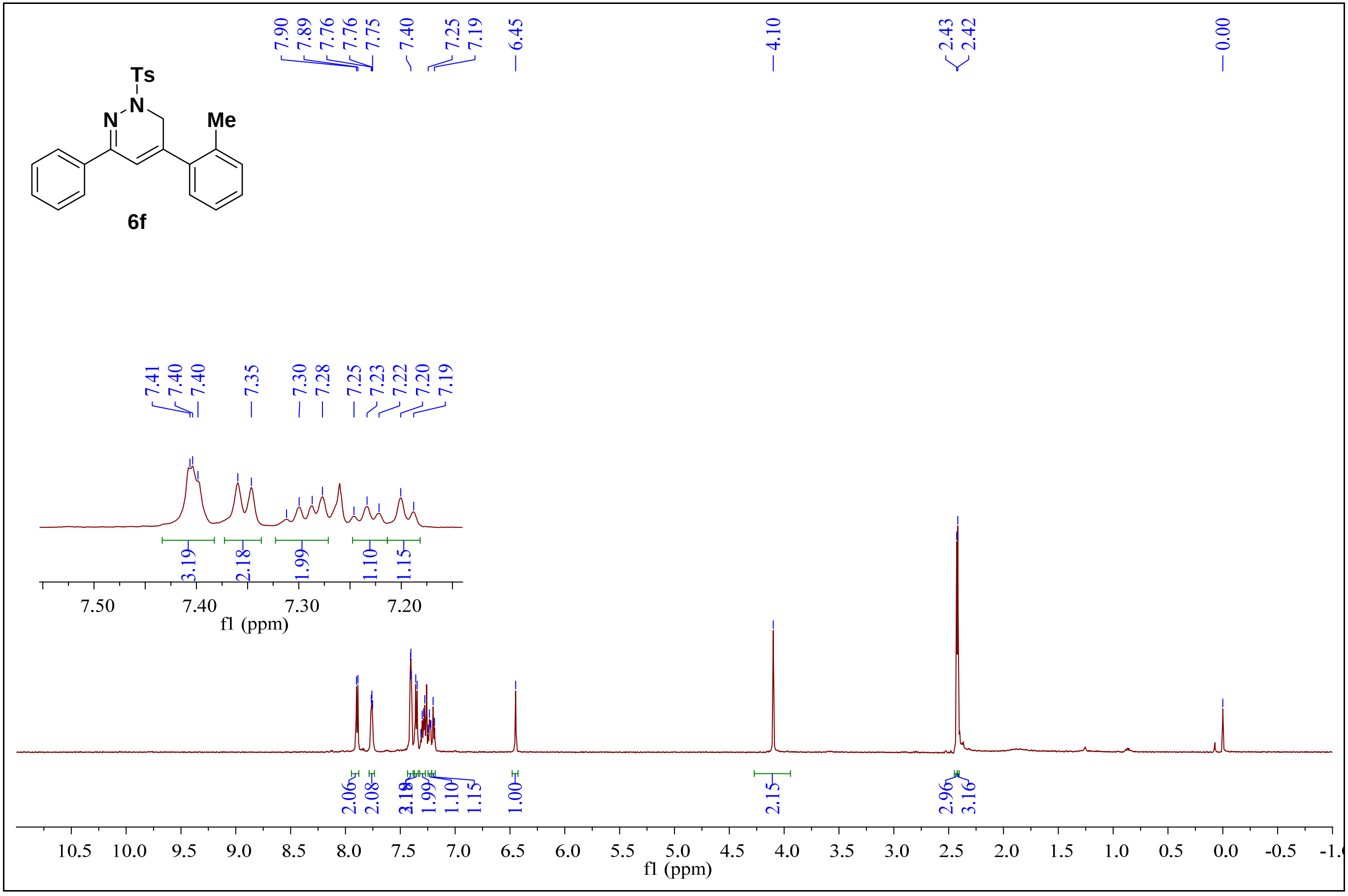

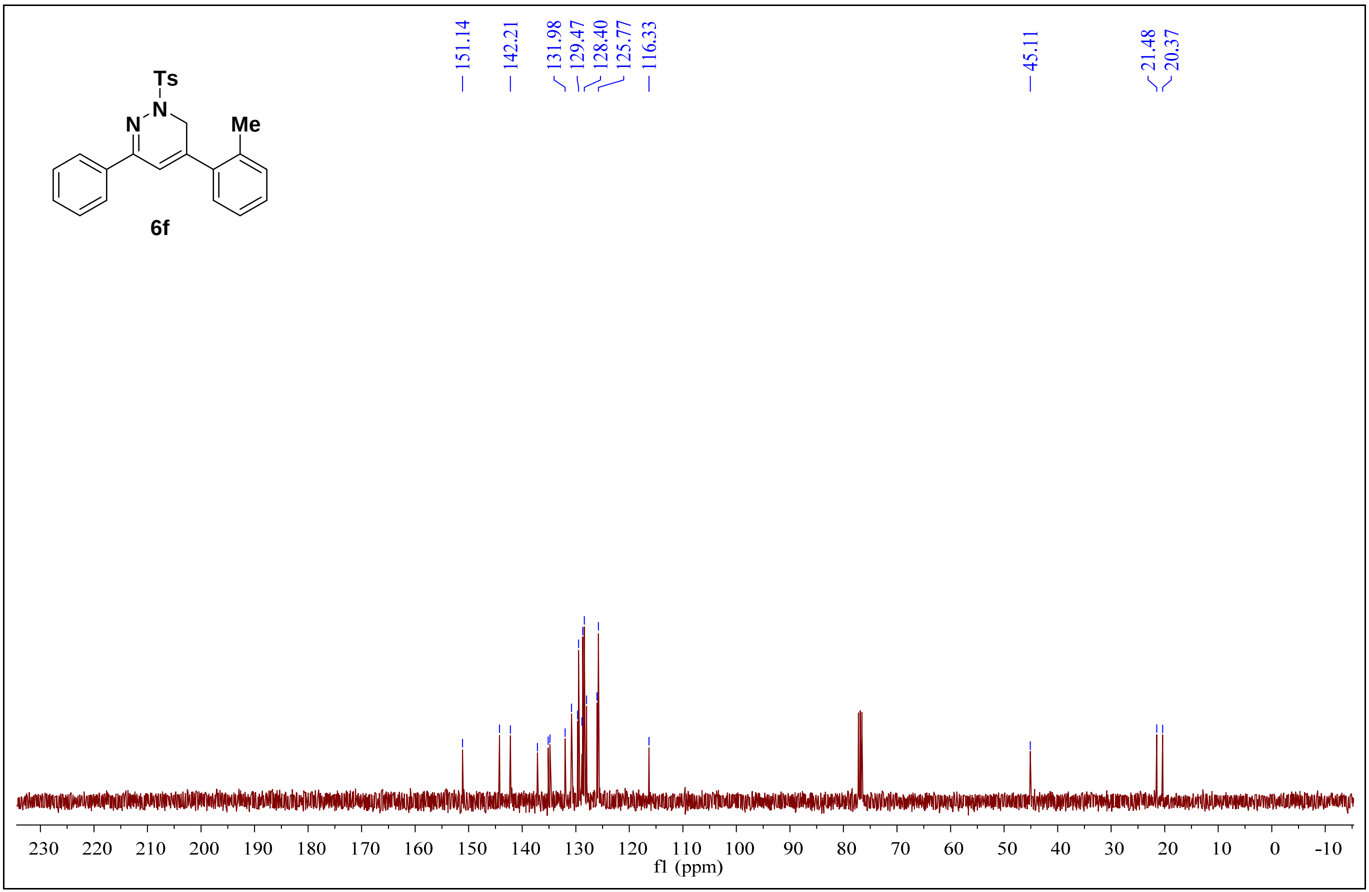

Supplementary Figure 58. **^{1}H NMR (600 MHz, $CDCl_3$) and ^{13}C NMR (100 MHz, $CDCl_3$) spectra of cyclic product 6g**

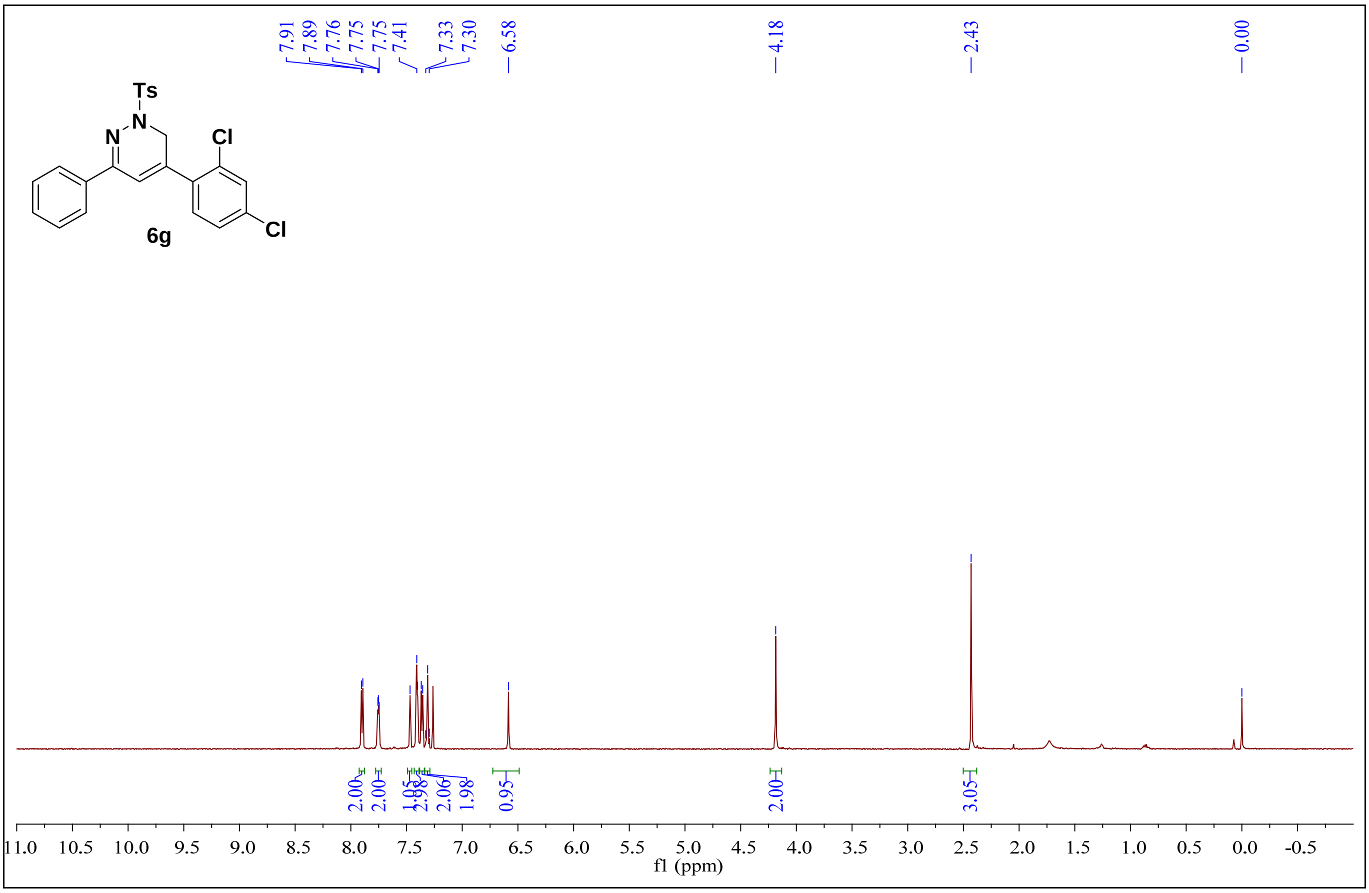

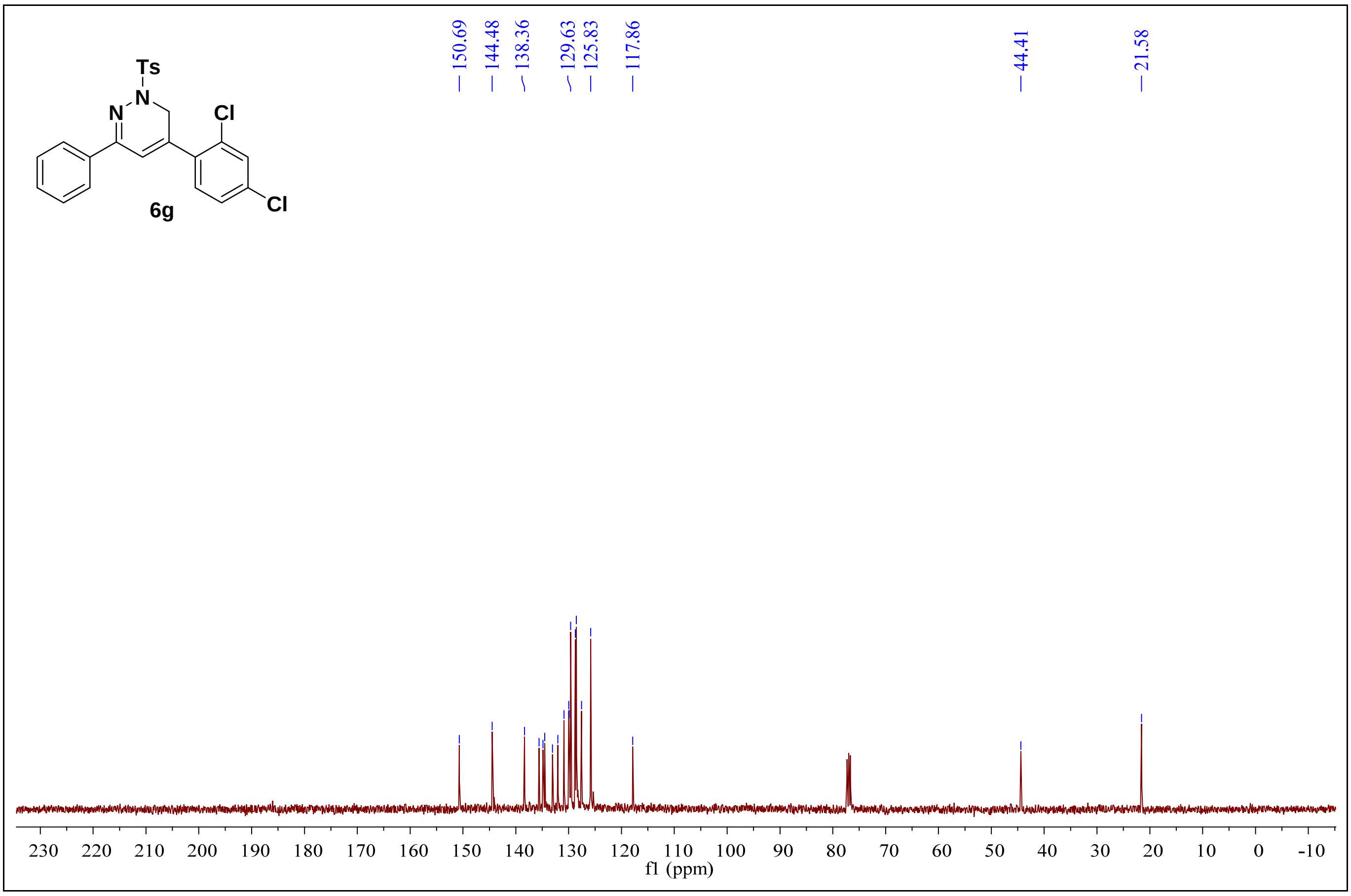

Supplementary Figure 59. ^{1}H **NMR (600 MHz, $CDCl_3$) and** ^{13}C **NMR (100 MHz, $CDCl_3$) spectra of pyridazine *N*-oxide 14**

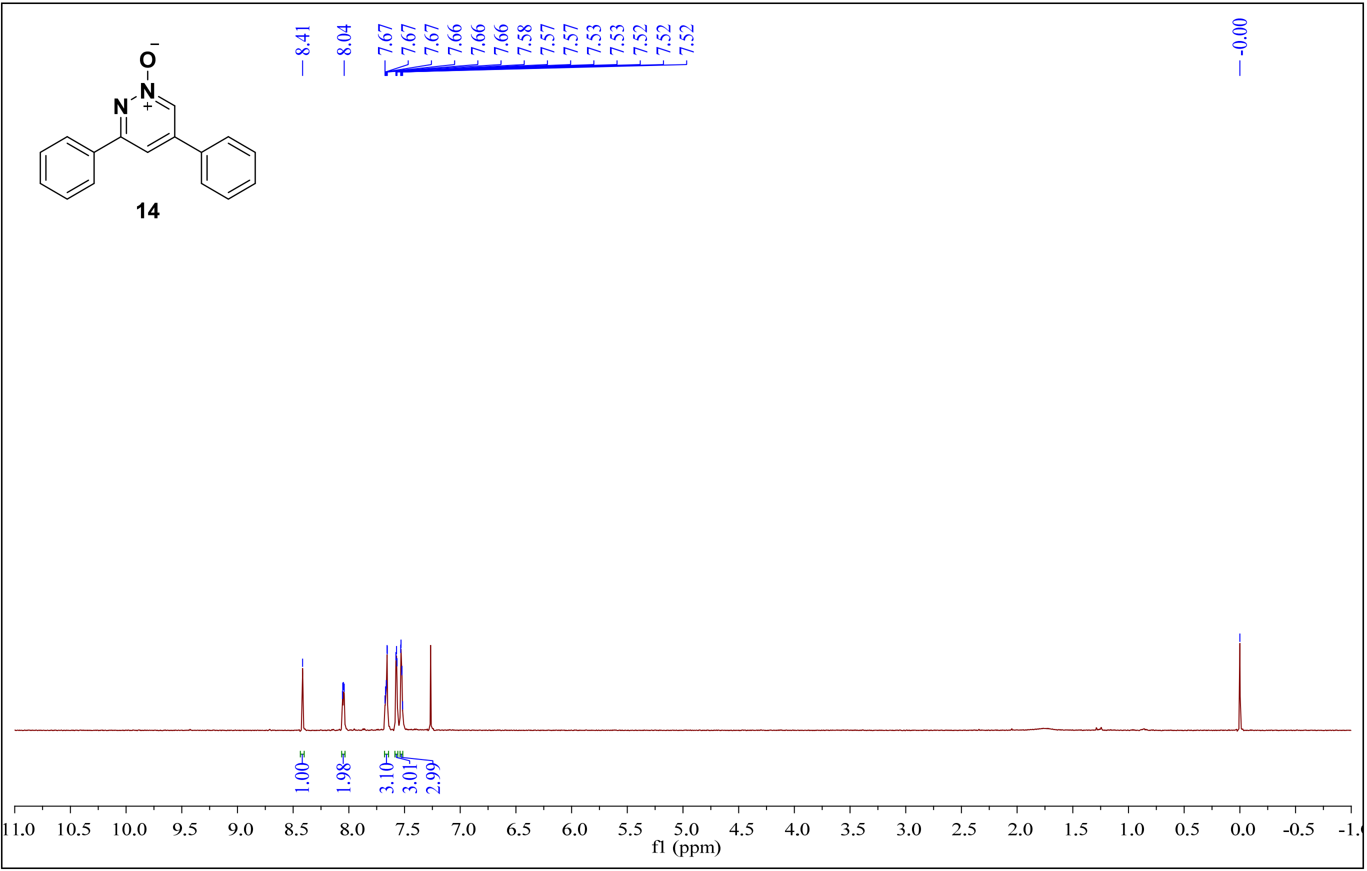

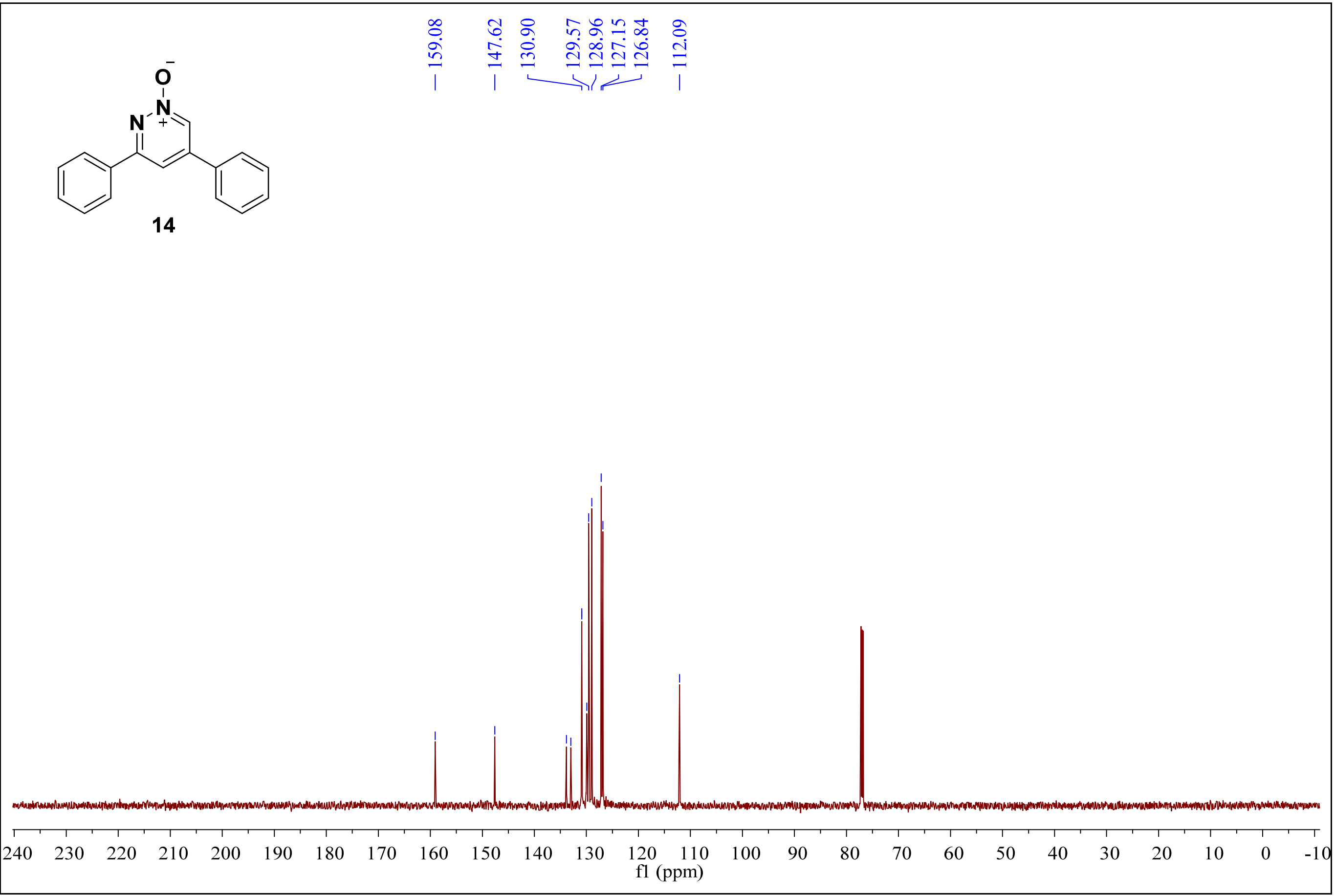

Supplementary Figure 60. ^{1}H **NMR (400 MHz,** $CDCl_3$**) and** ^{13}C **NMR (100 MHz,** $CDCl_3$**) spectra of cyclic product 15a**

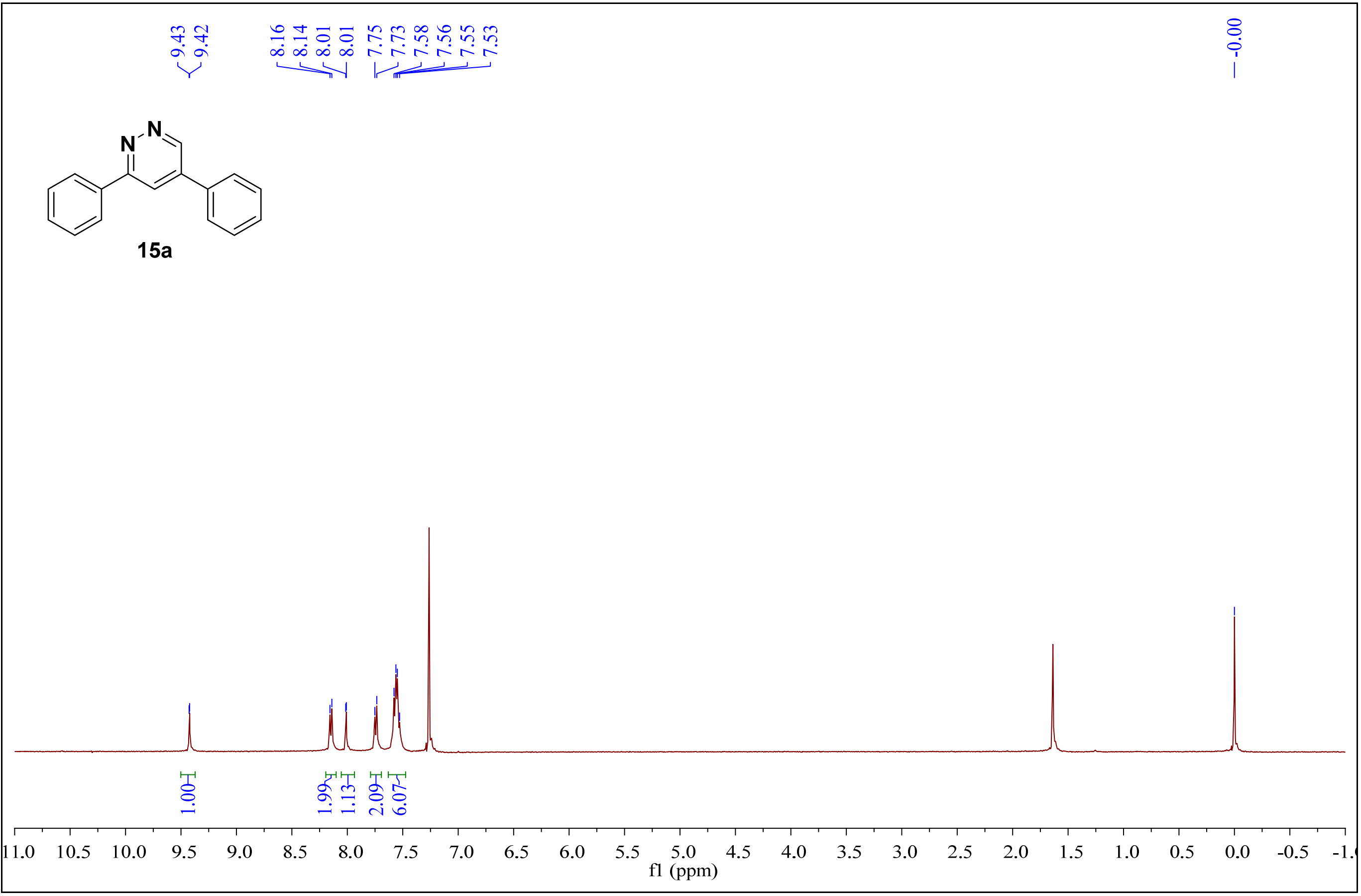

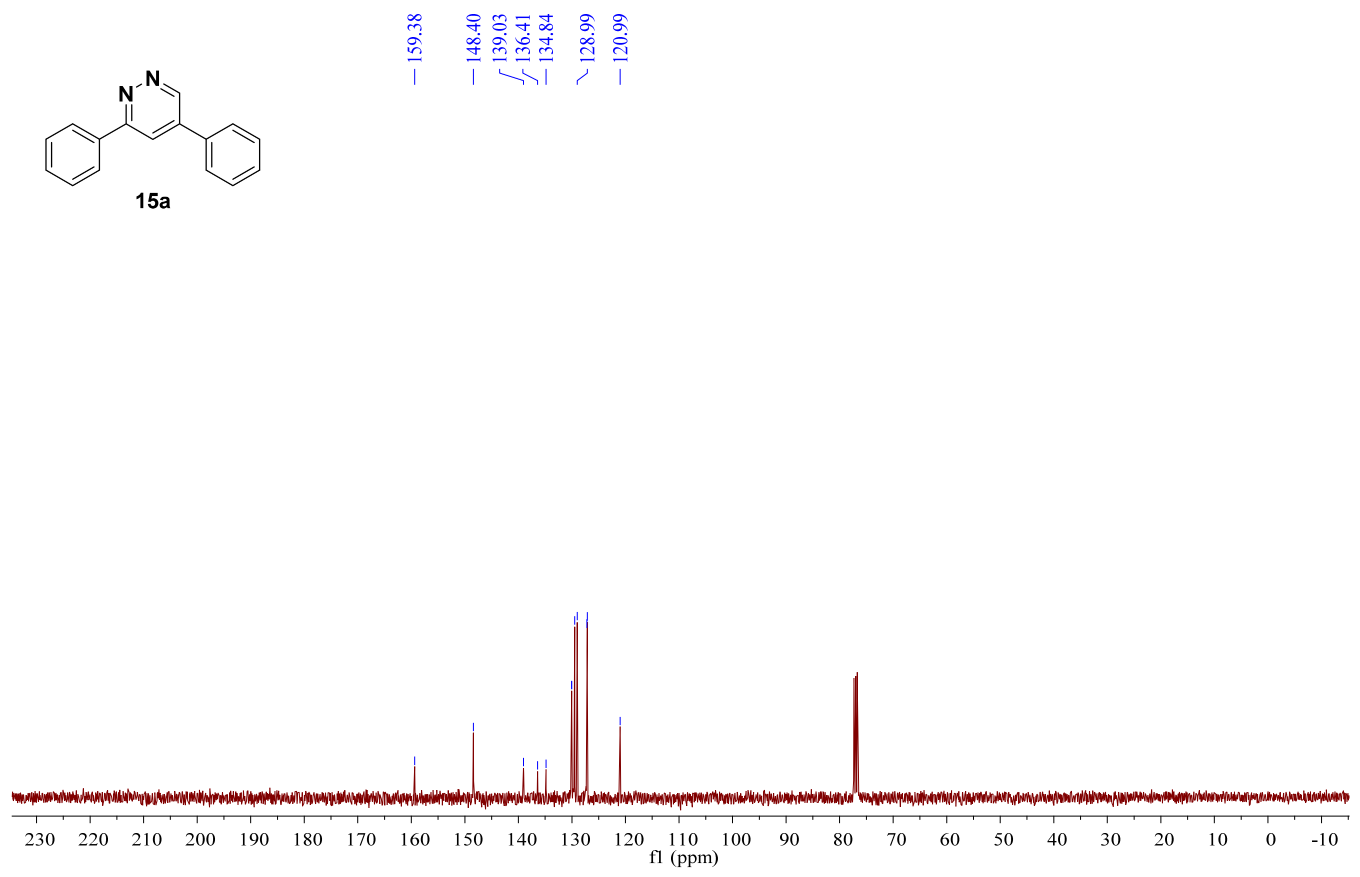

Supplementary Figure 61. ^{1}H NMR (600 MHz, $CDCl_3$) and ^{13}C NMR (100 MHz, $CDCl_3$) spectra of cyclic product **15b**

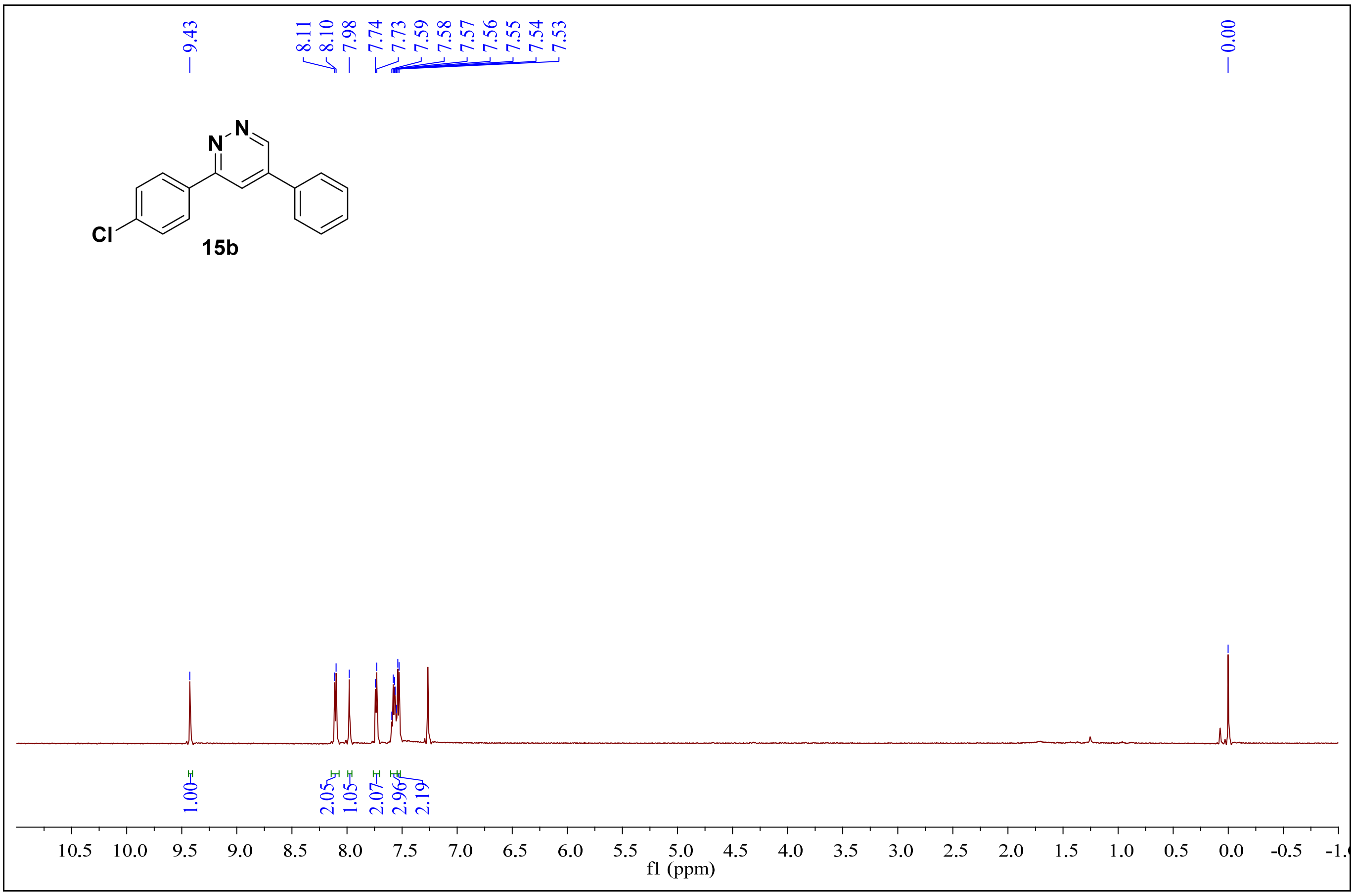

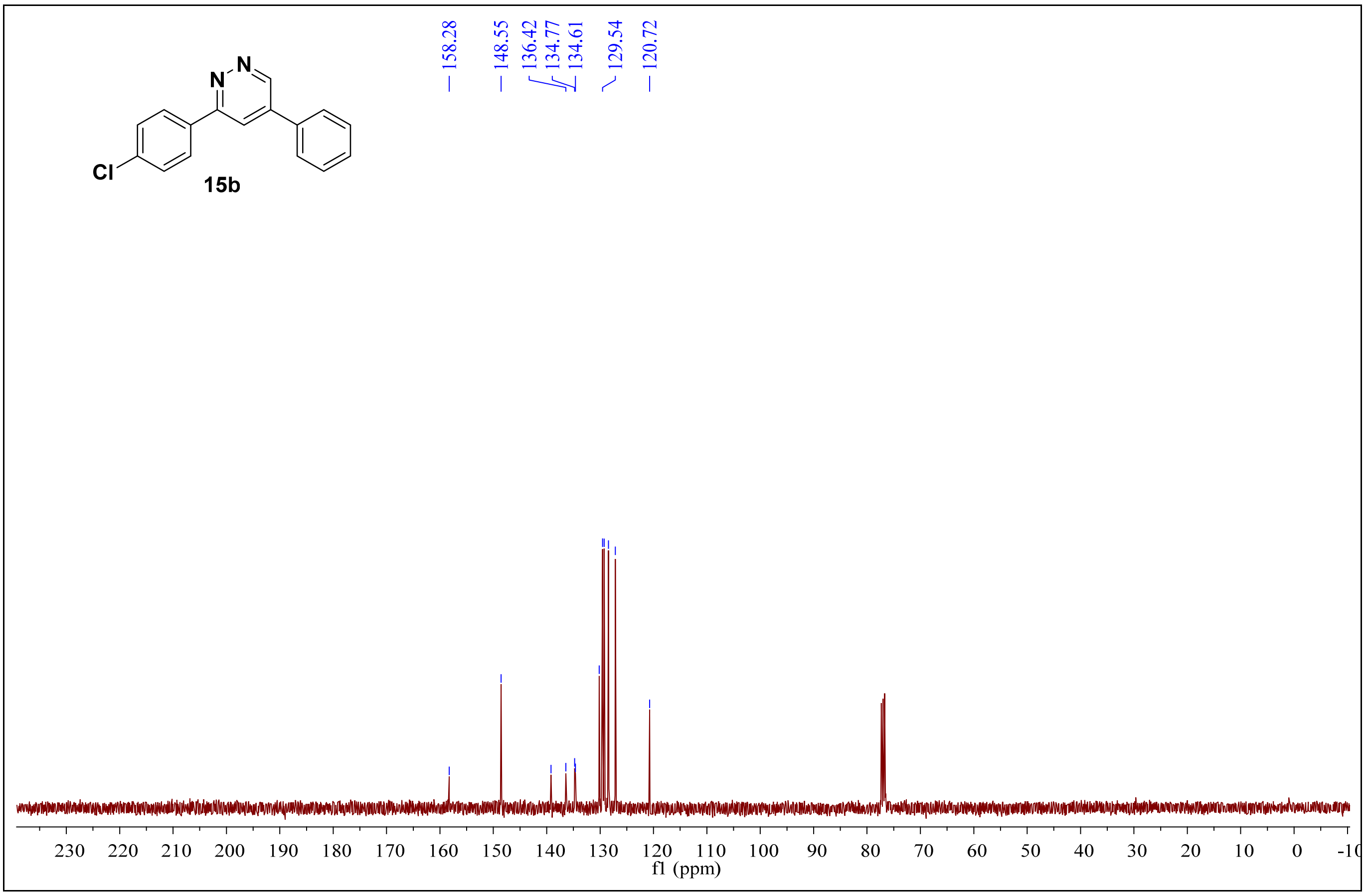

Supplementary Figure 62. ^{1}**H NMR (400 MHz, CDCl$_3$) and** ^{13}**C NMR (100 MHz, CDCl$_3$) spectra of cyclic product 15c**

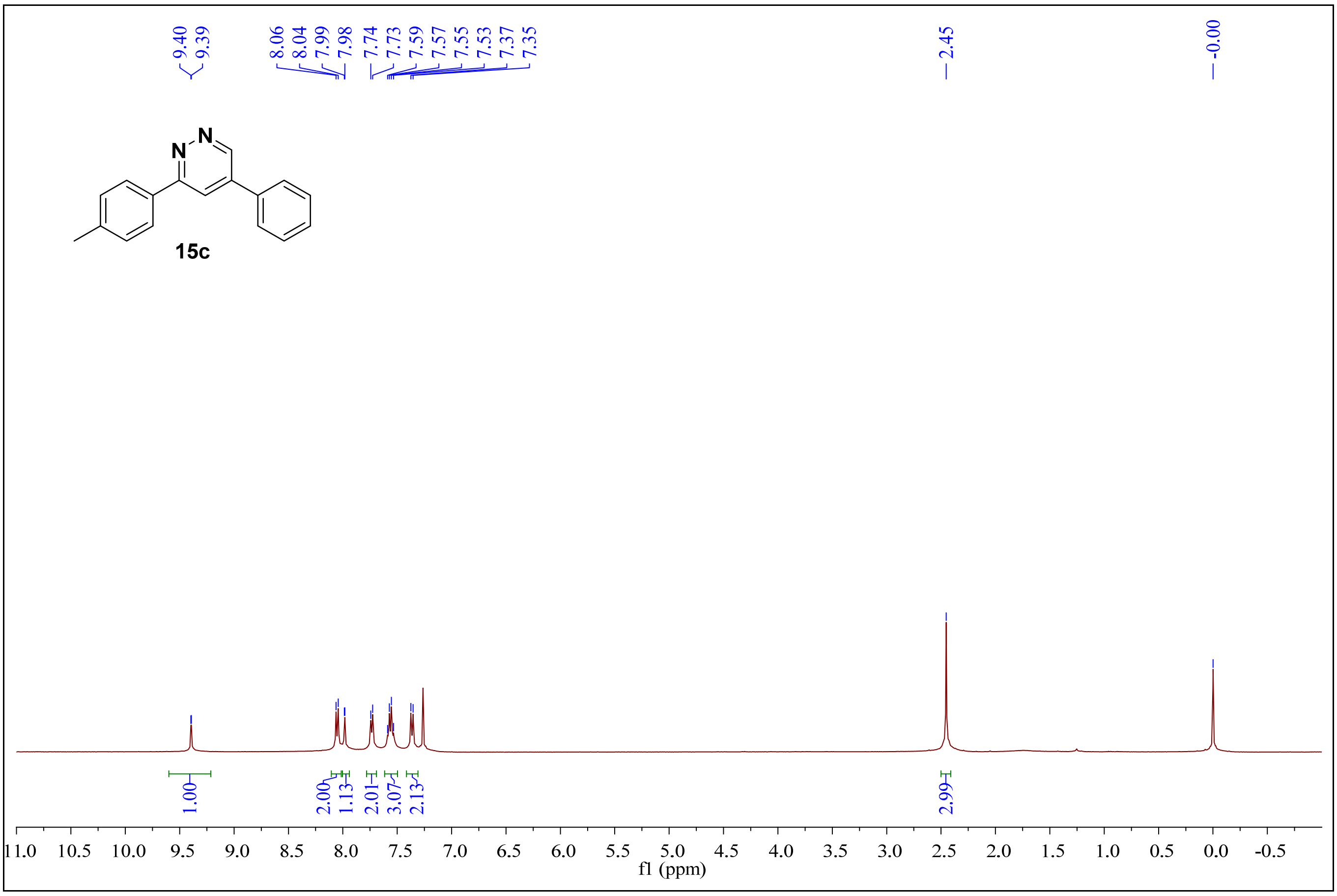

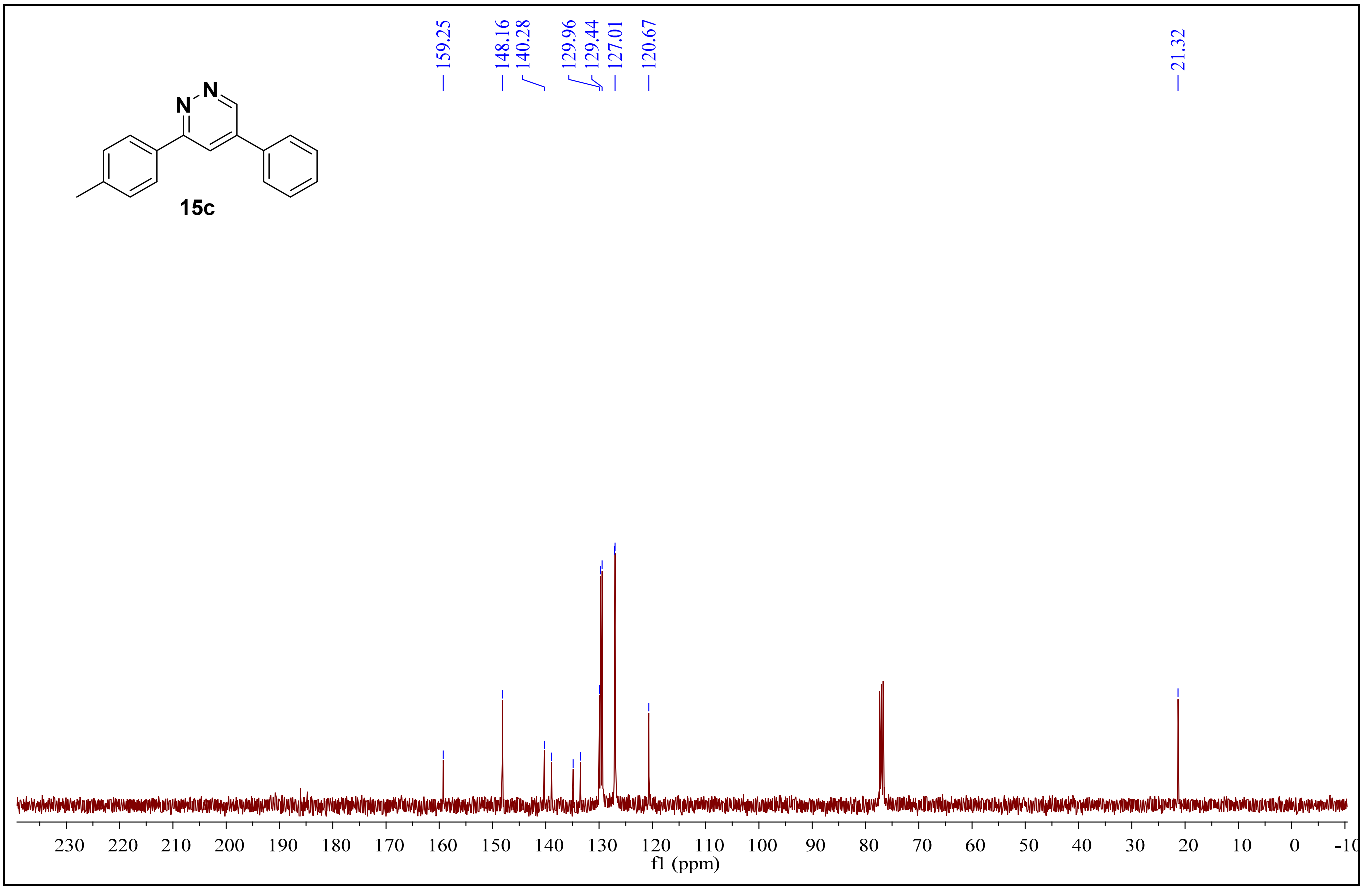

Supplementary Figure 63. ^{1}H NMR (600 MHz, $CDCl_3$) and ^{13}C NMR (100 MHz, $CDCl_3$) spectra of cyclic product **15d**

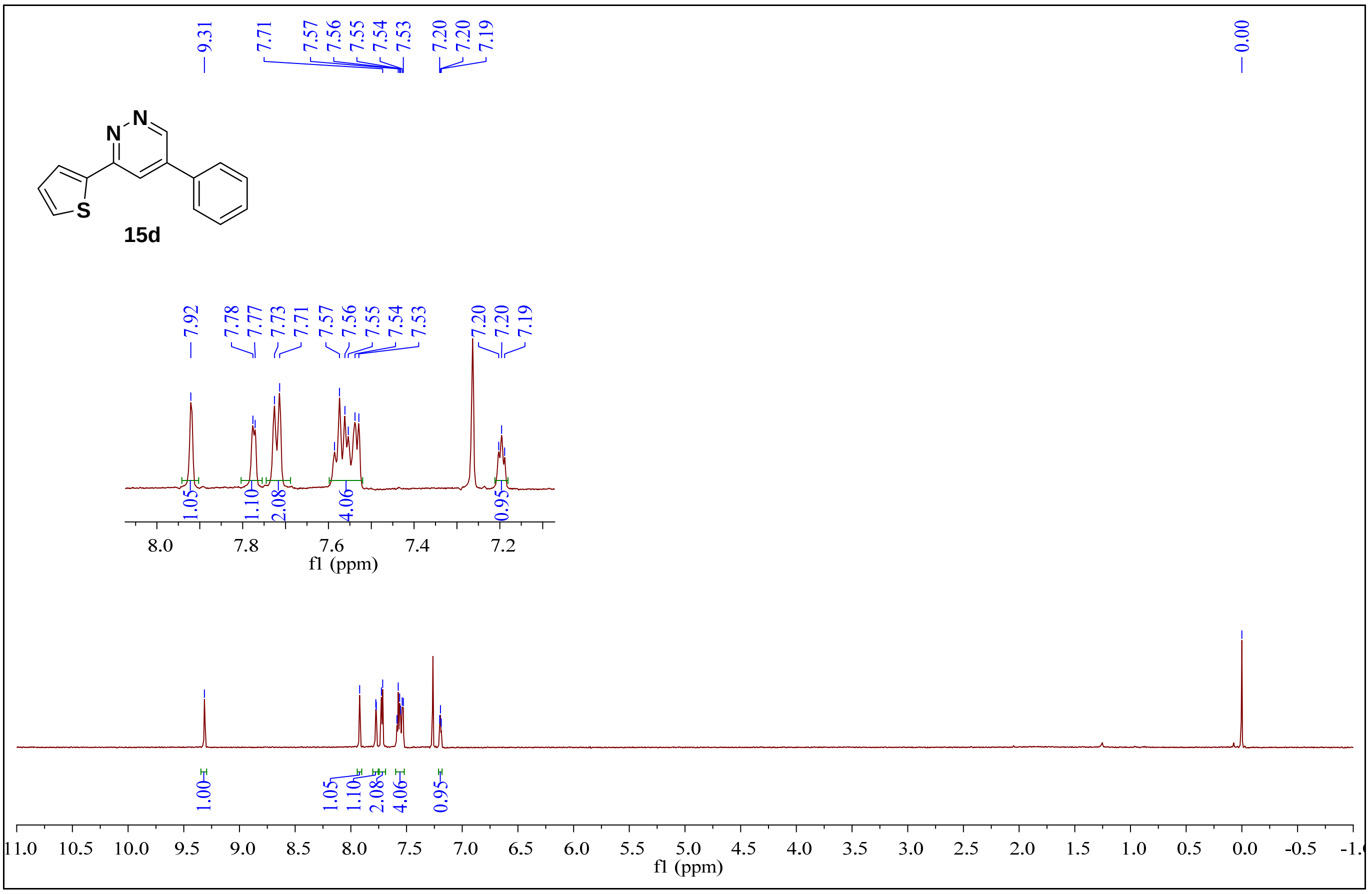

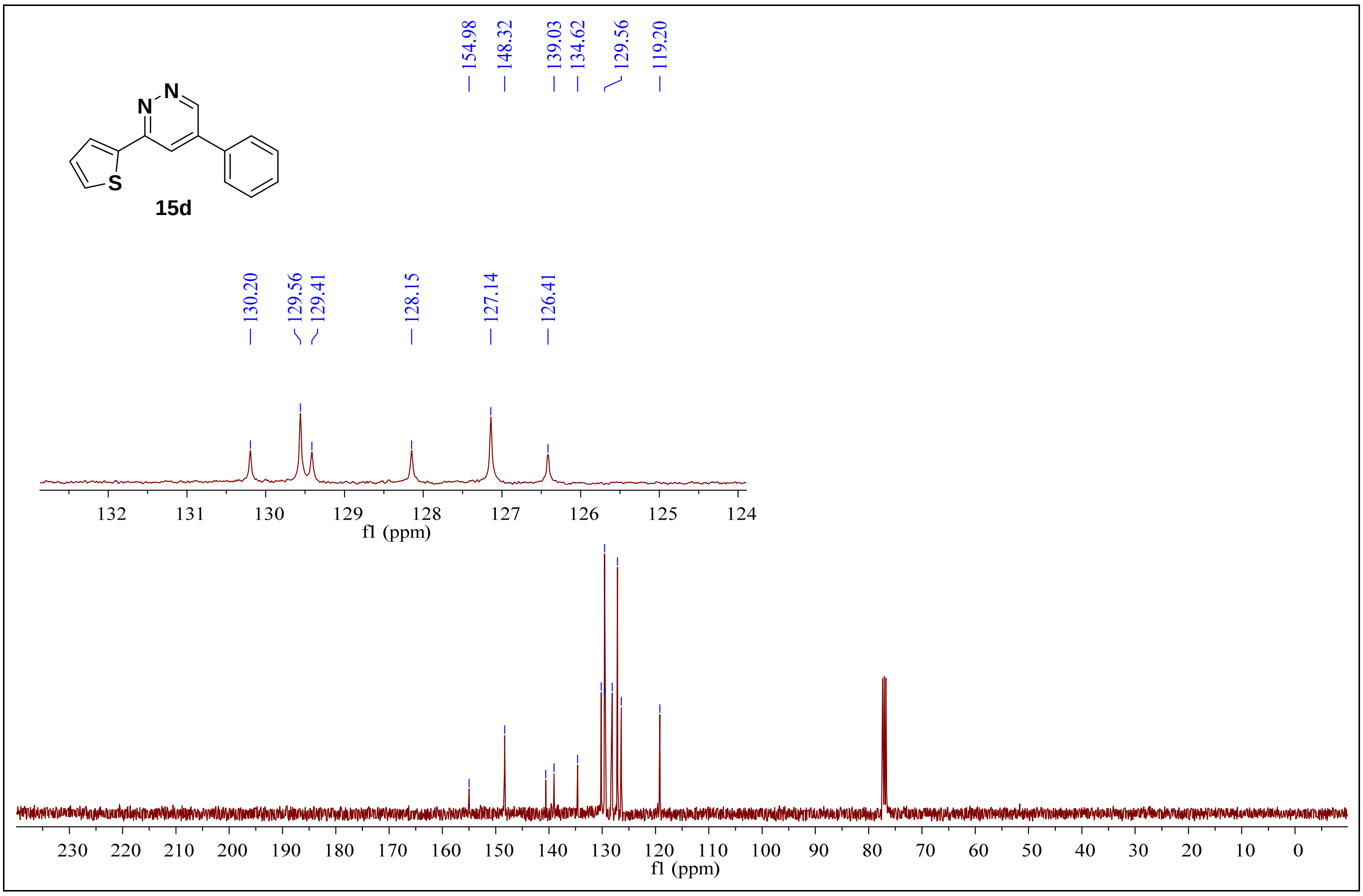

Supplementary Figure 64. ^{1}**H NMR (600 MHz, CDCl$_3$) and** ^{13}**C NMR (100 MHz, CDCl$_3$) spectra of cyclic product 15e**

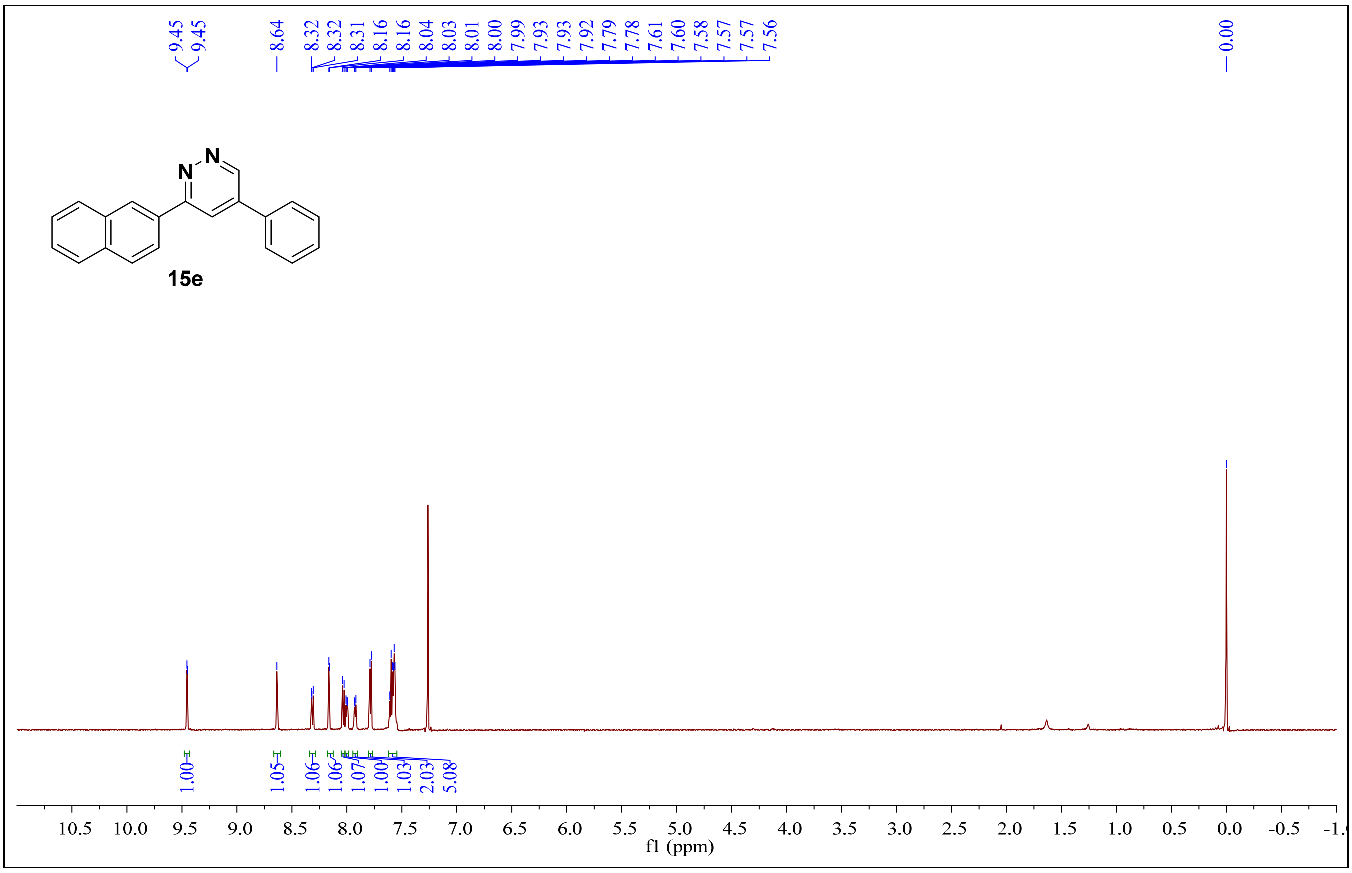

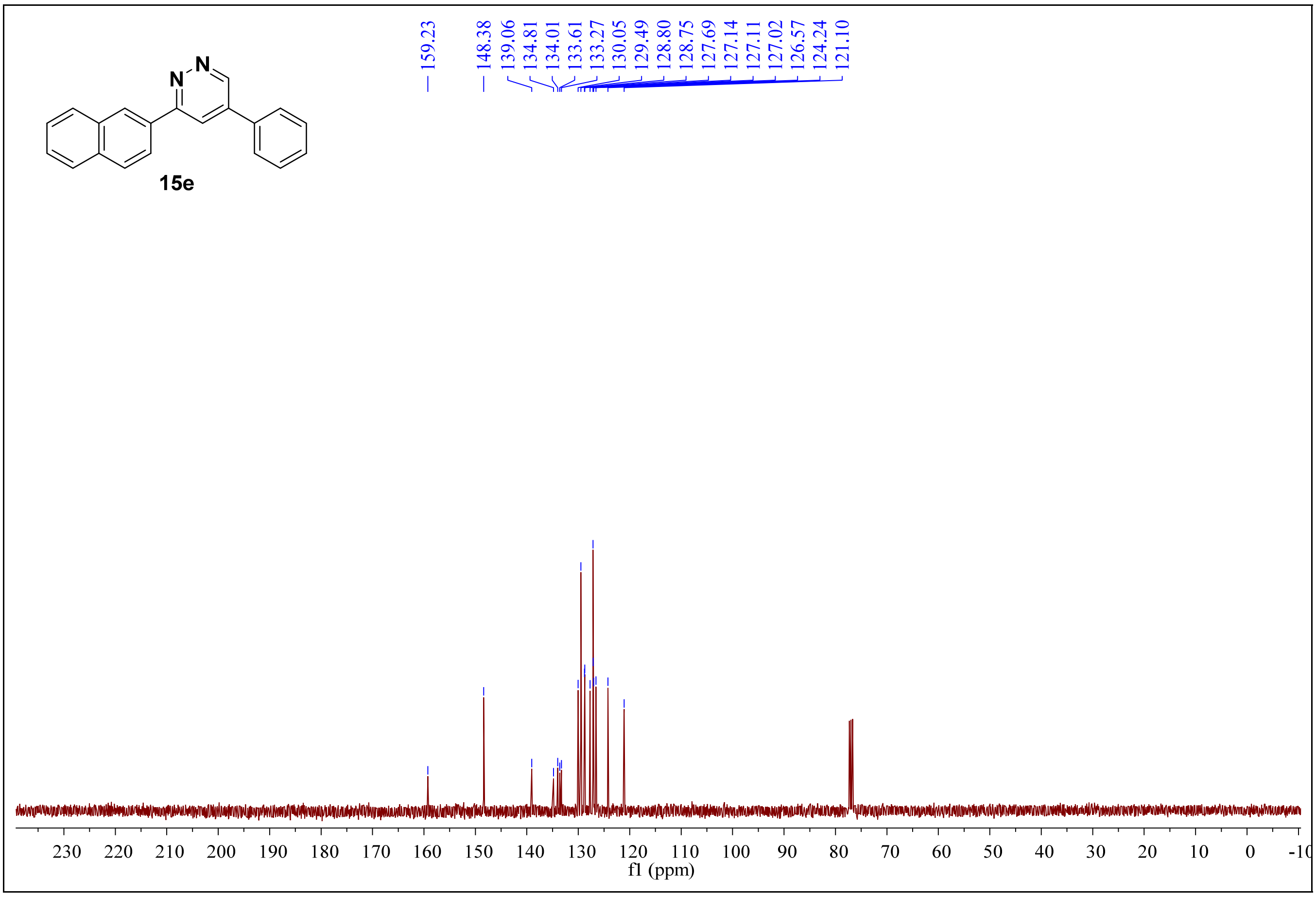

Supplementary Figure 65. ^{1}H NMR (400 MHz, $CDCl_3$) and ^{13}C NMR (100 MHz, $CDCl_3$) spectra of cyclic product **15f**

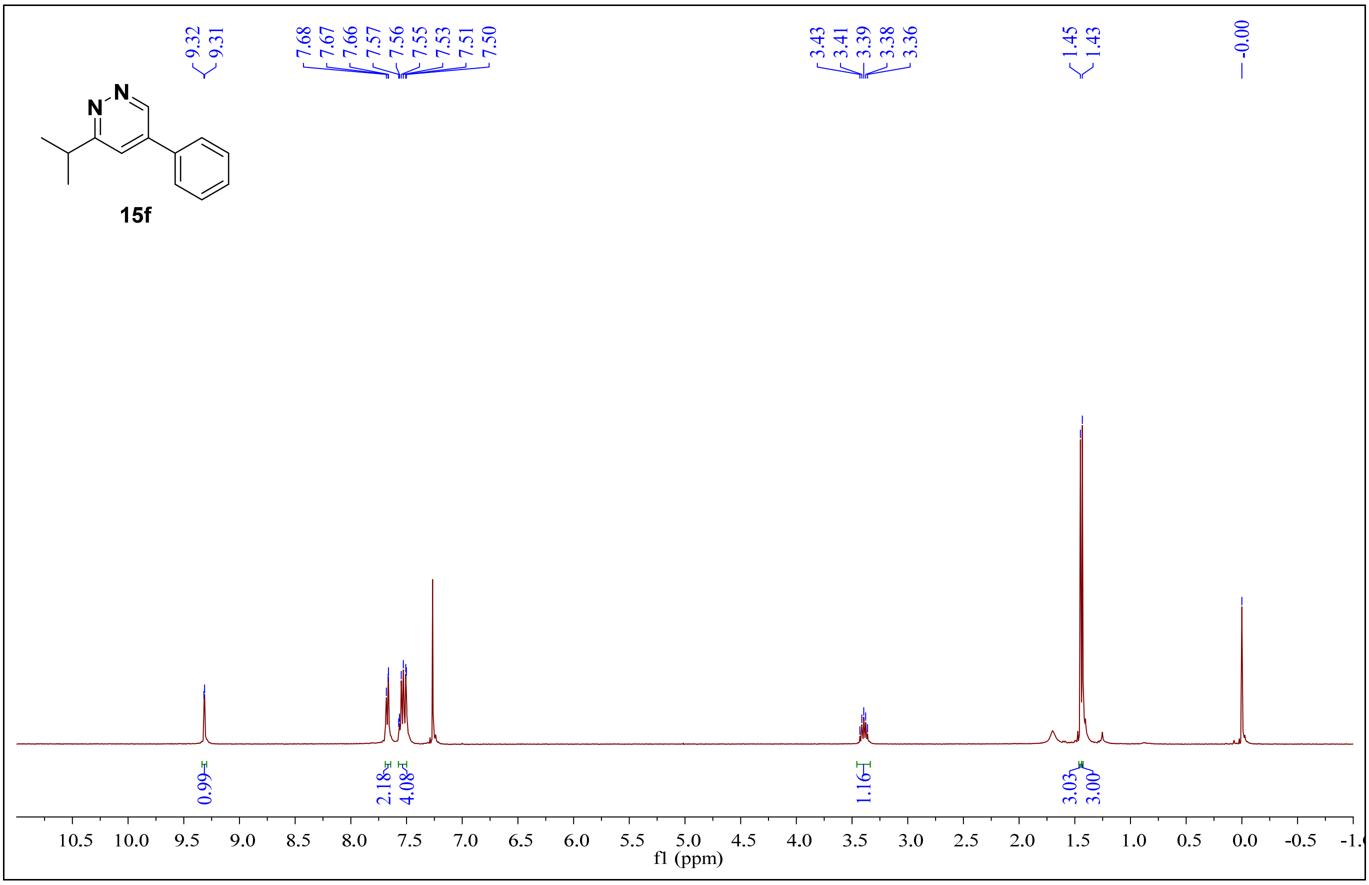

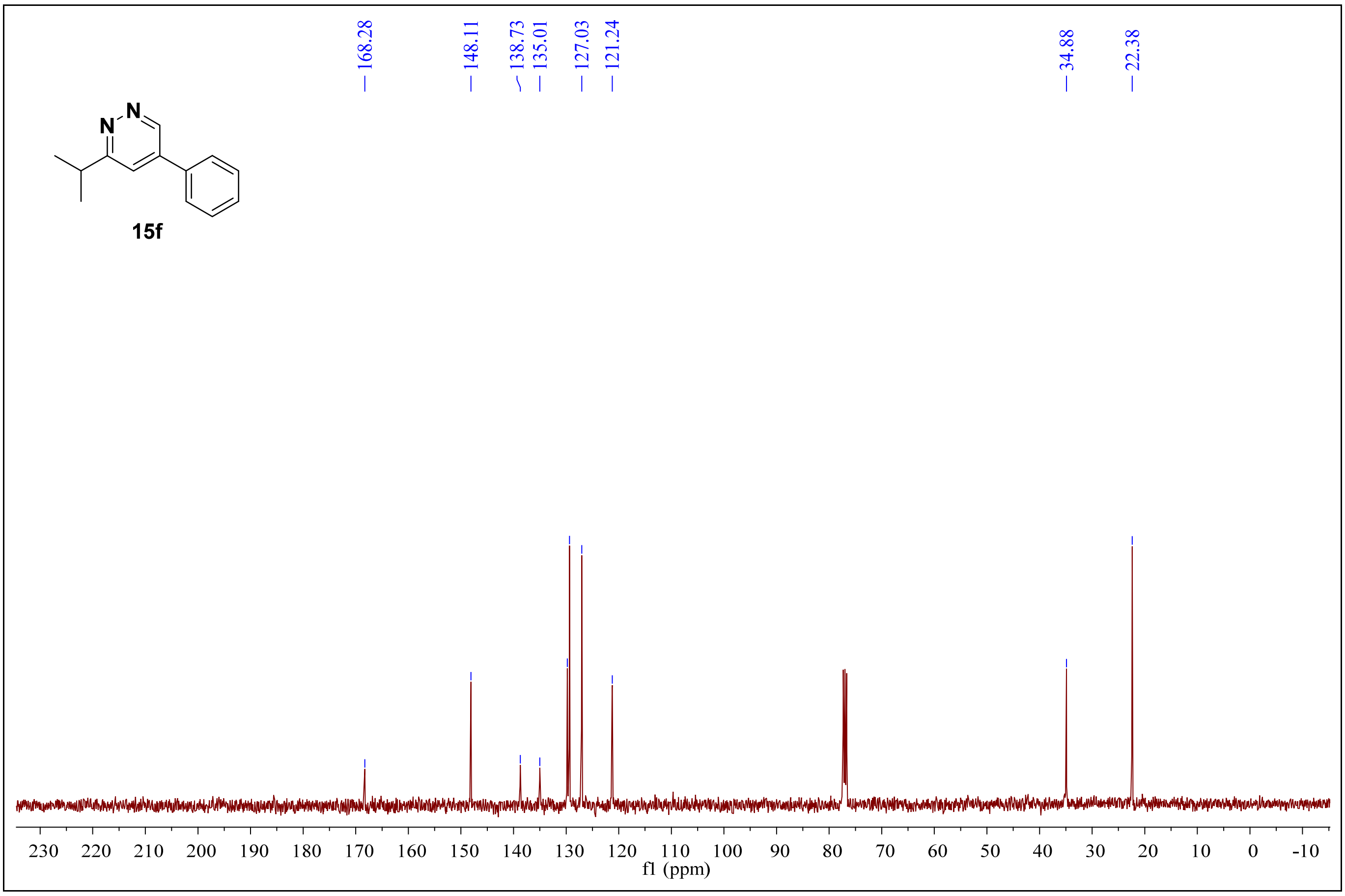

Supplementary Figure 66. ^{1}**H NMR (400 MHz, CDCl$_3$) and** ^{13}**C NMR (100 MHz, CDCl$_3$) spectra of cyclic product 15g**

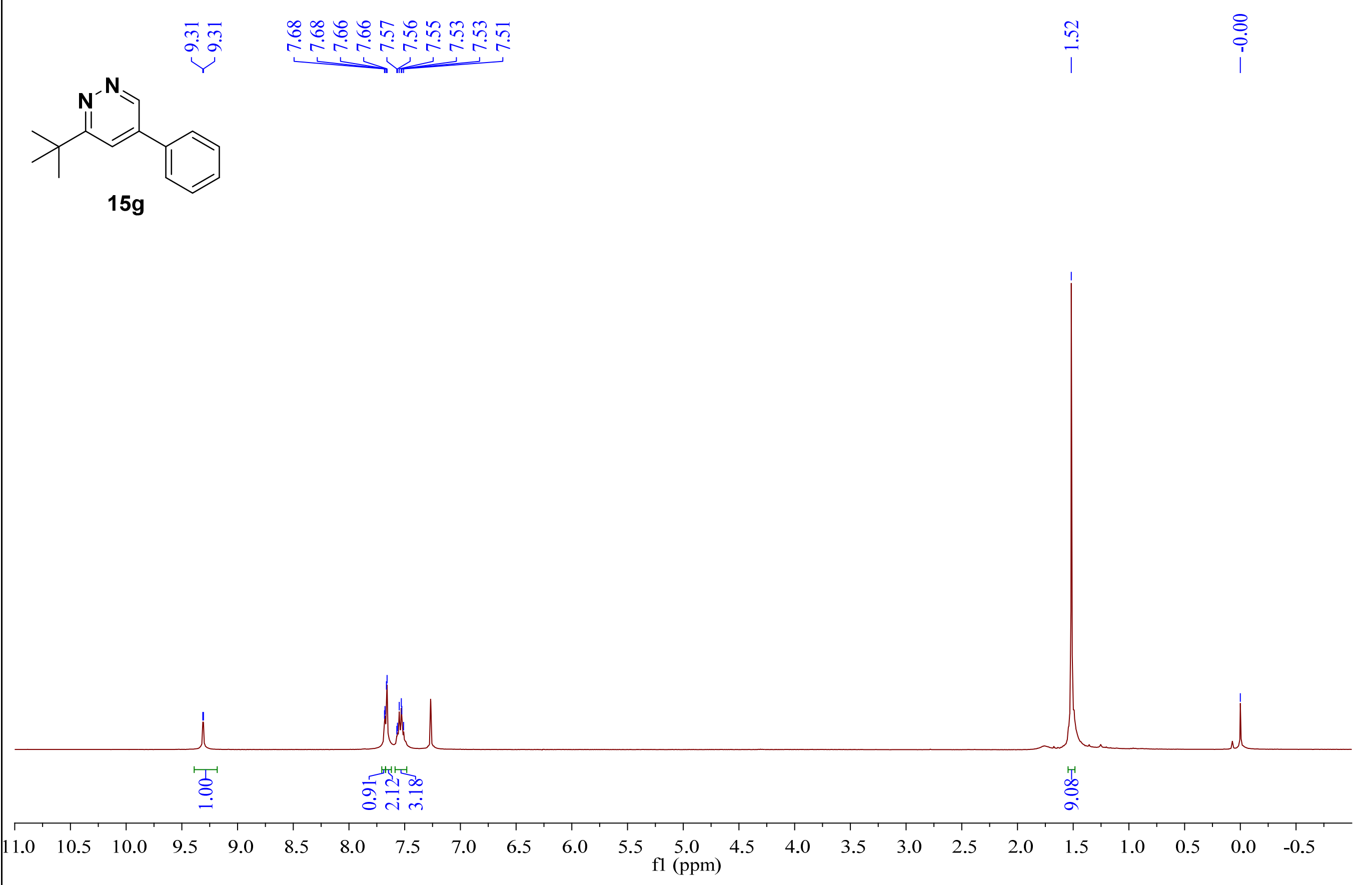

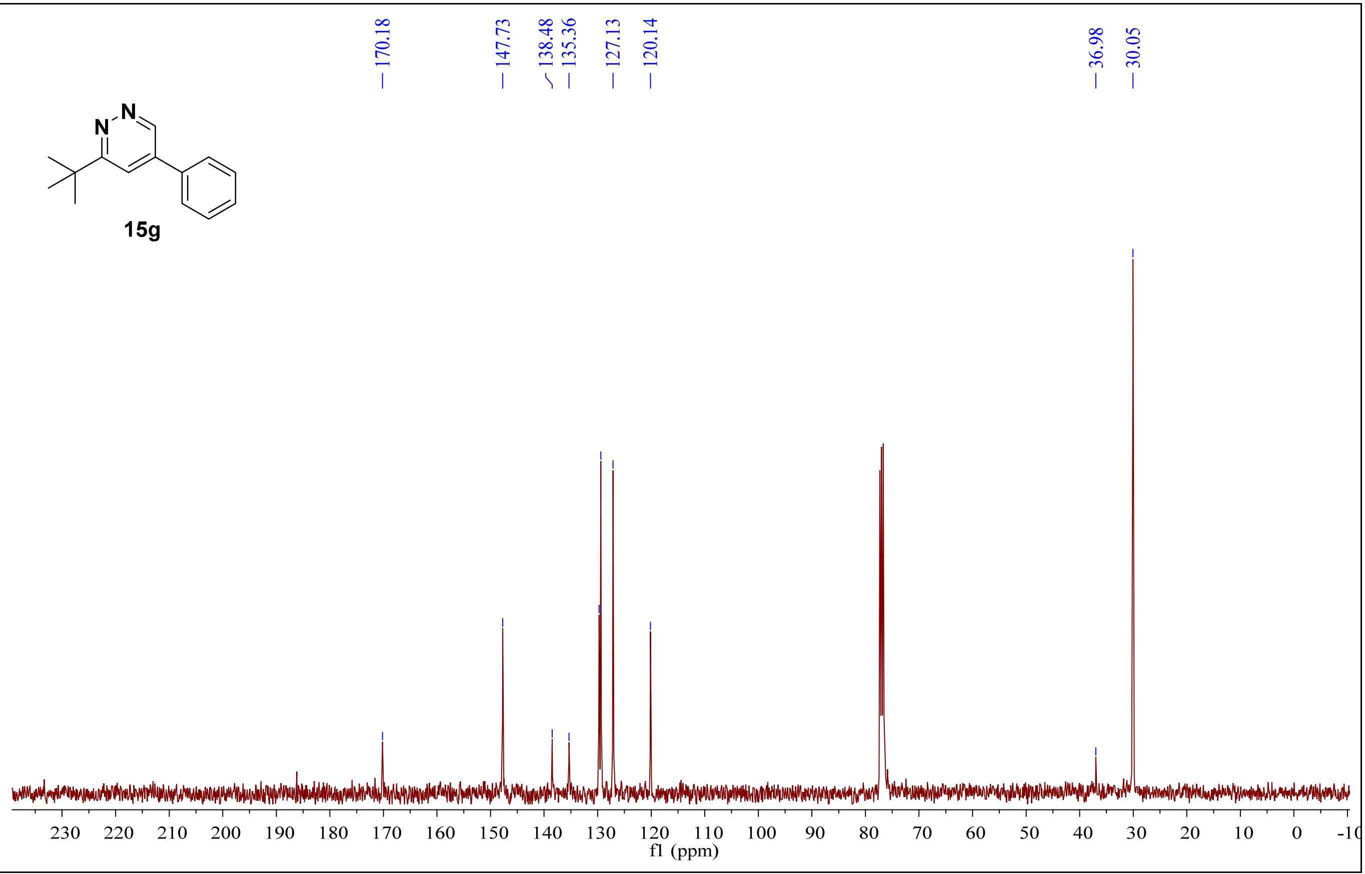

Supplementary Figure 67. ^{1}H **NMR (600 MHz,** $CDCl_3$**) and** ^{13}C **NMR (100 MHz,** $CDCl_3$**) spectra of cyclic product 15h**

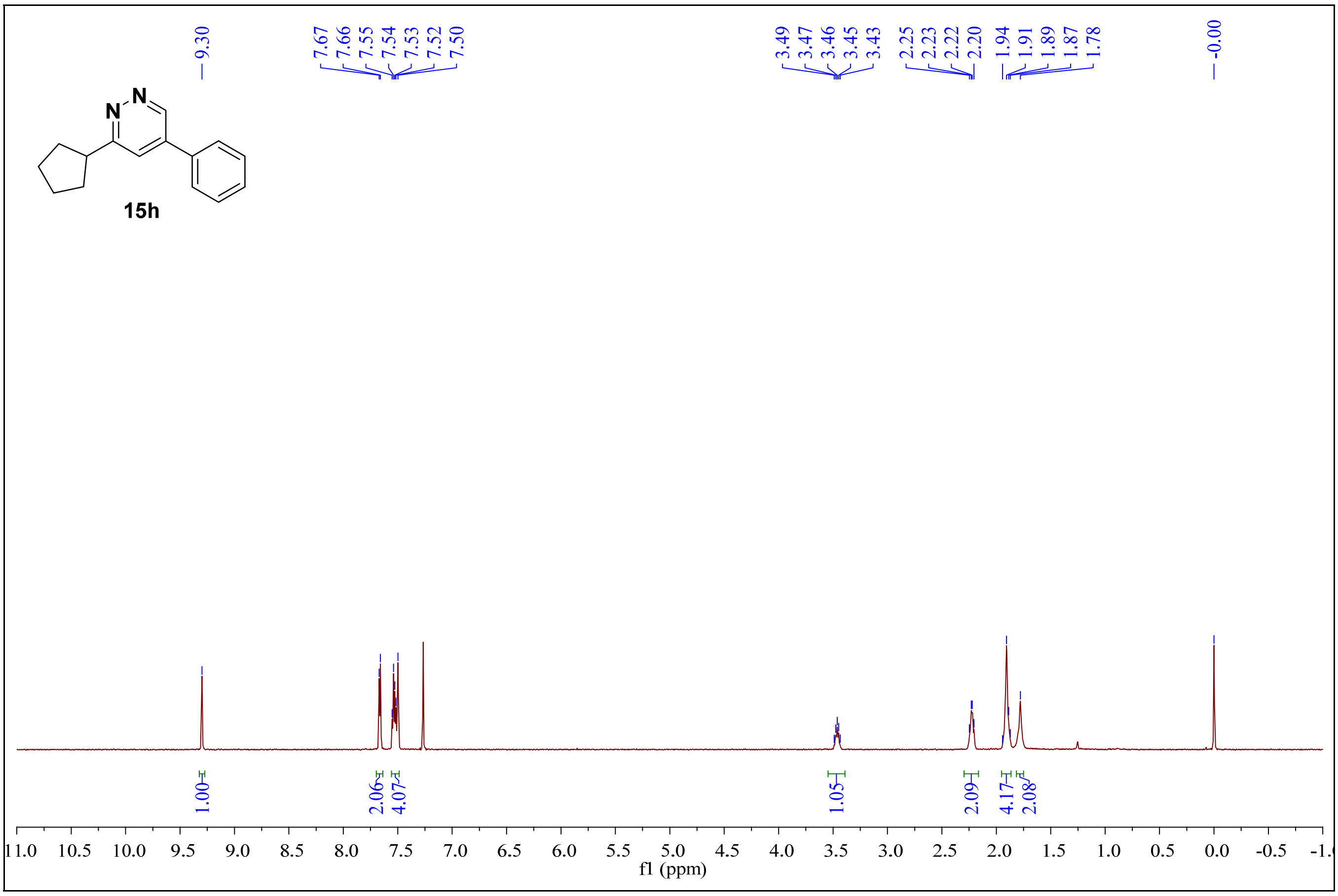

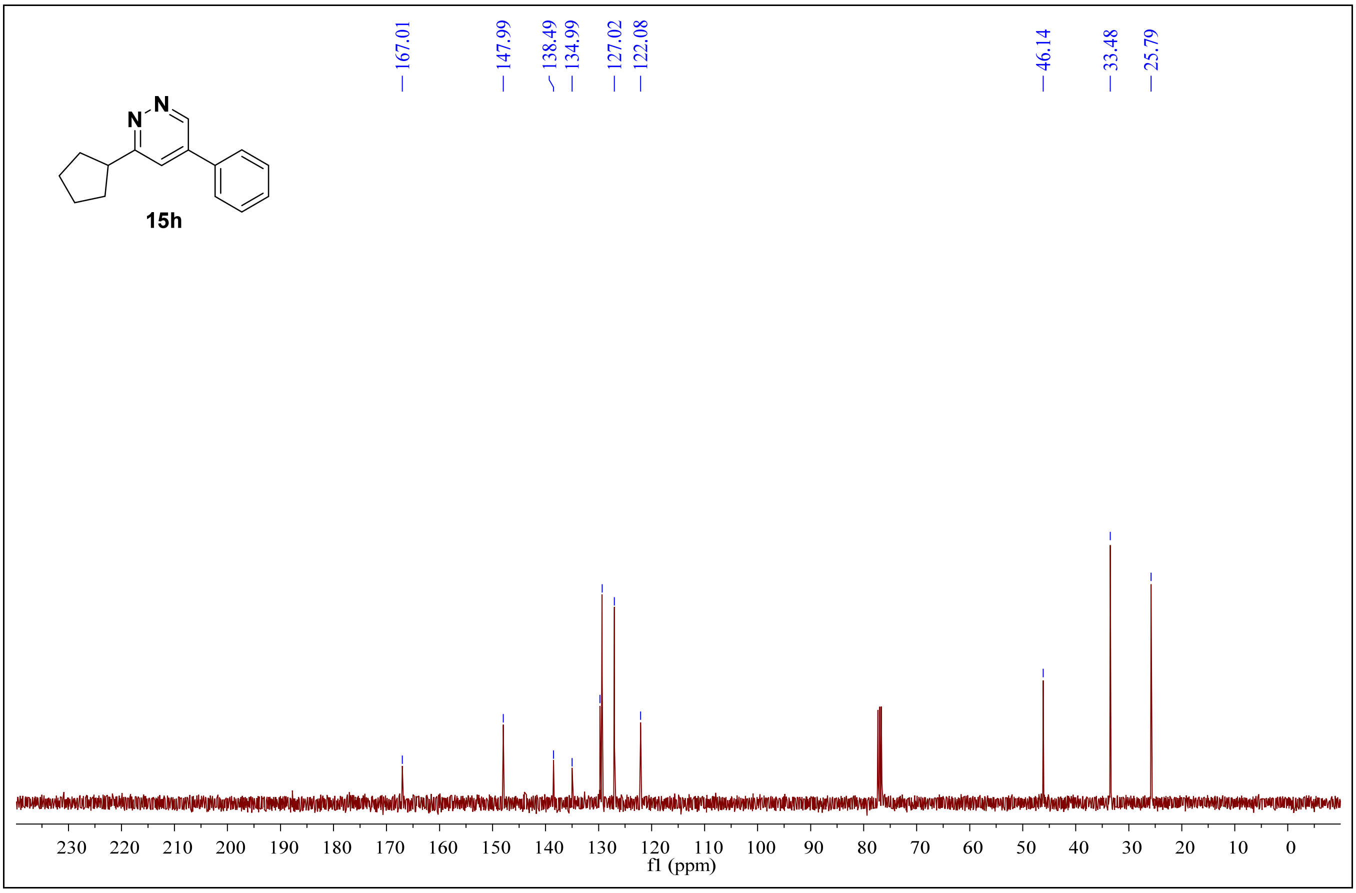

Supplementary Figure 68. ^{1}H NMR (600 MHz, $CDCl_3$) and ^{13}C NMR (150 MHz, $CDCl_3$) spectra of cyclic product **15i**

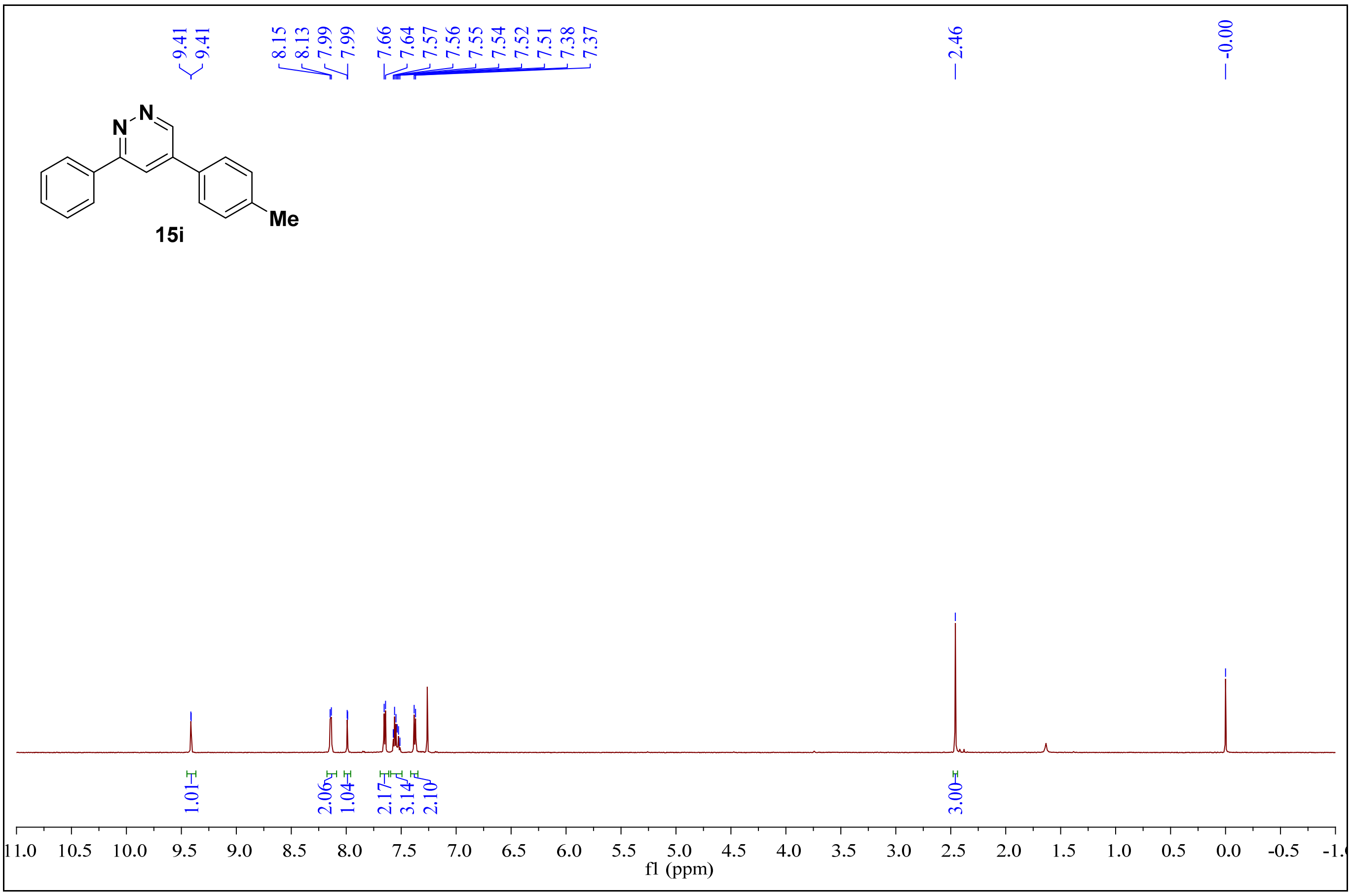

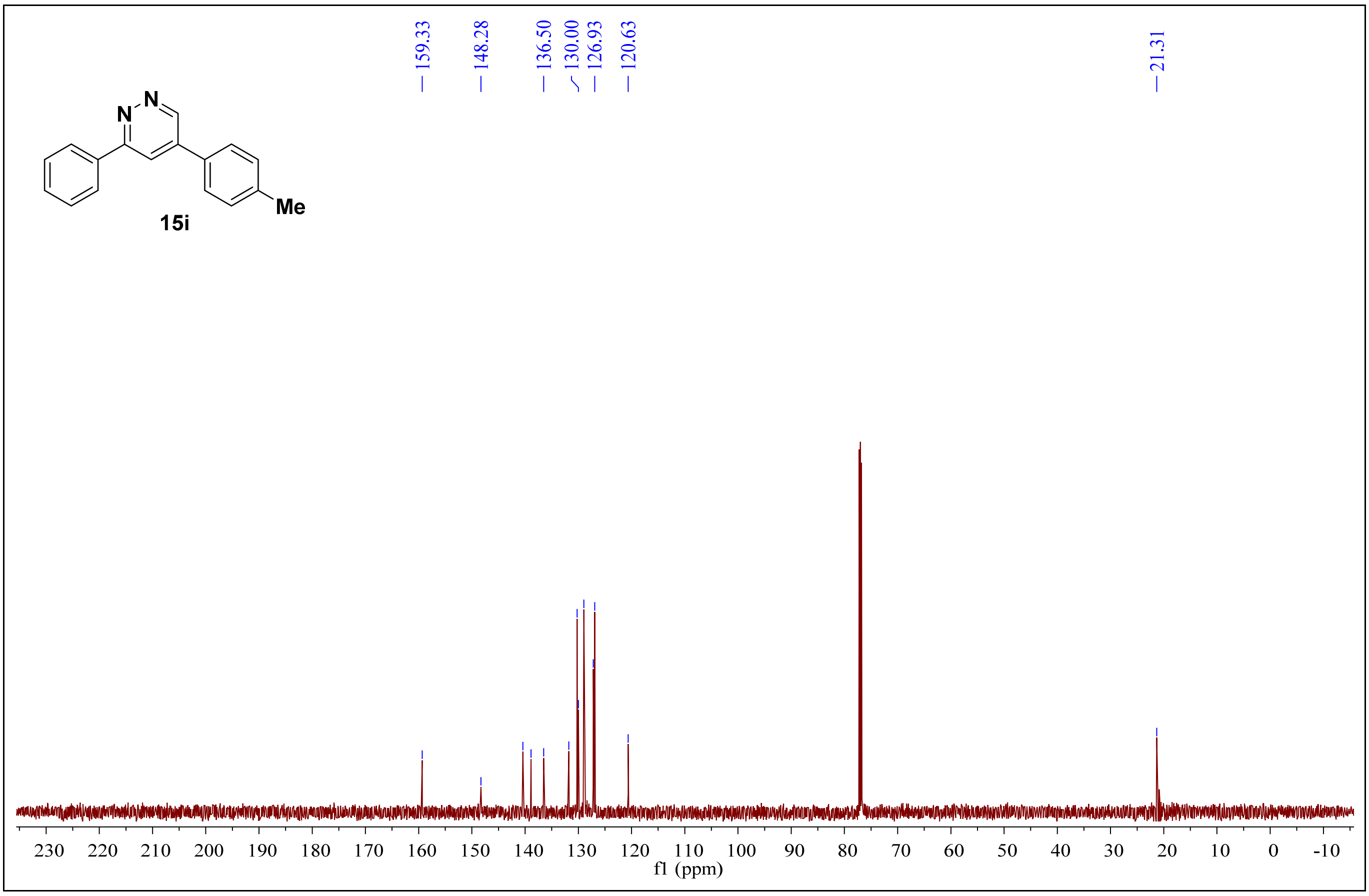

Supplementary Figure 69. ^{1}H **NMR (600 MHz,** $CDCl_3$**) and** ^{13}C **NMR (100 MHz,** $CDCl_3$**) spectra of cyclic product 15j**

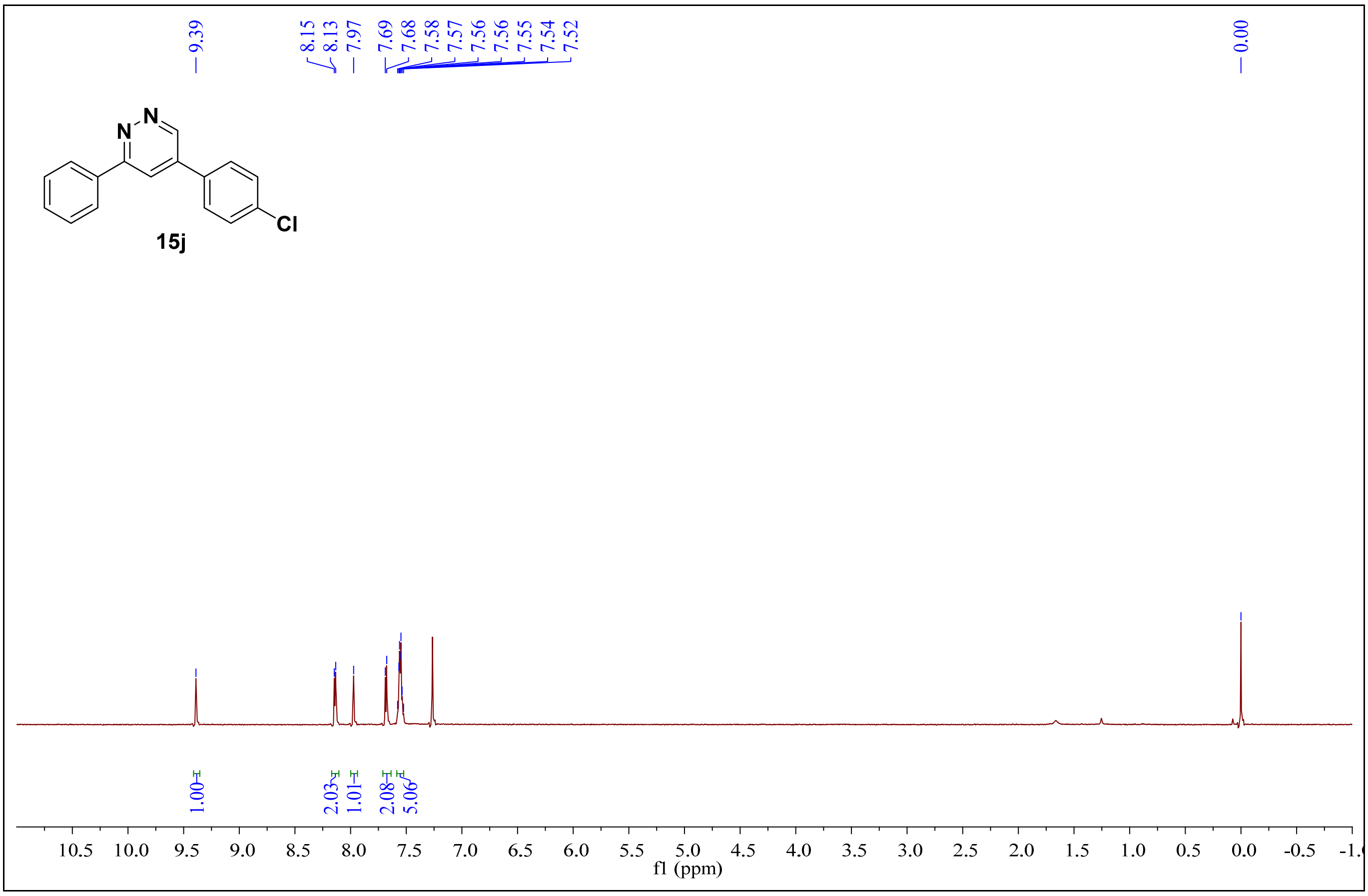

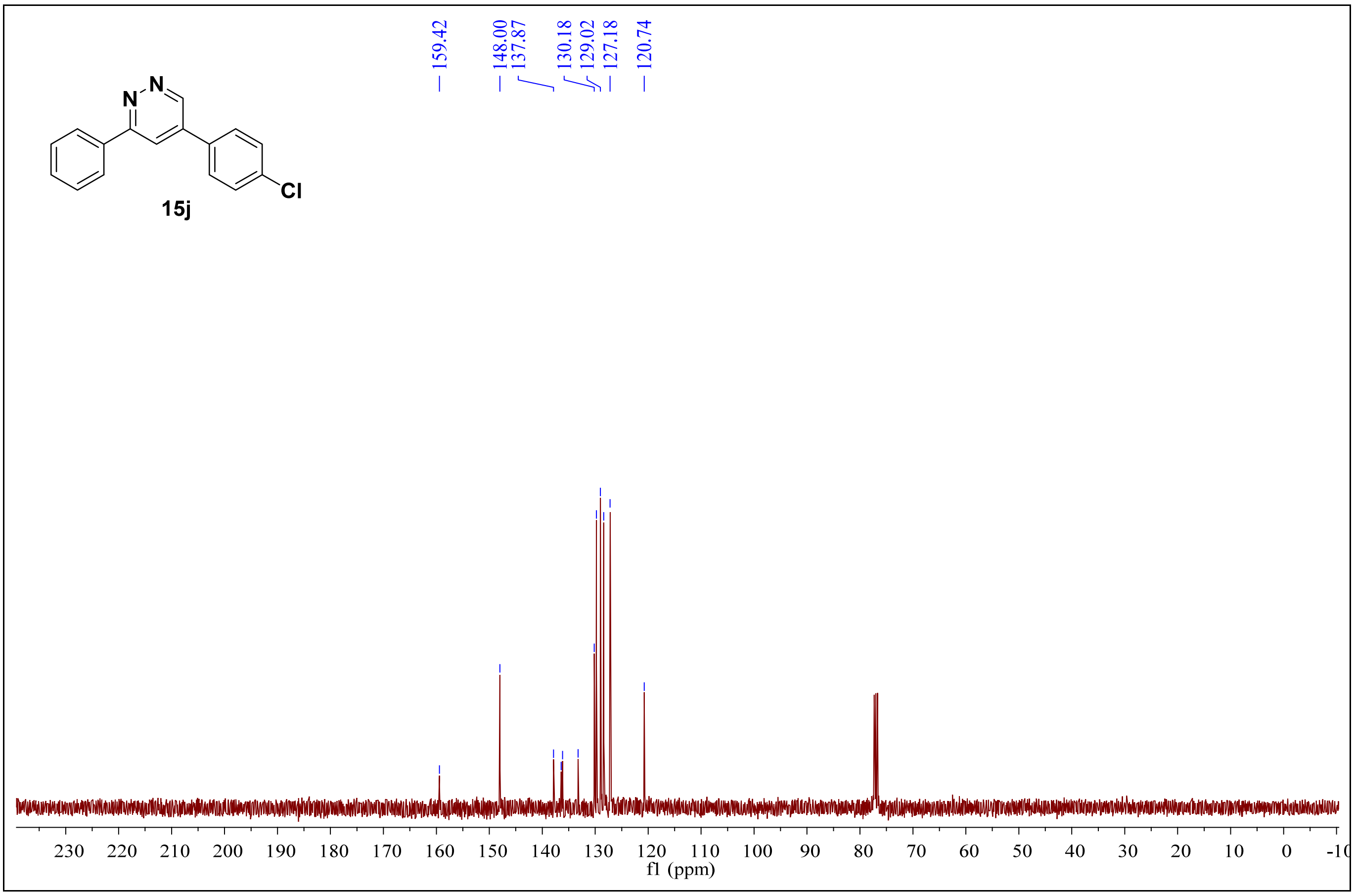

Supplementary Figure 70. ^{1}H NMR (600 MHz, $CDCl_3$), ^{13}C NMR (100 MHz, $CDCl_3$) and ^{19}F (376 MHz, $CDCl_3$) spectra of cyclic product 15k

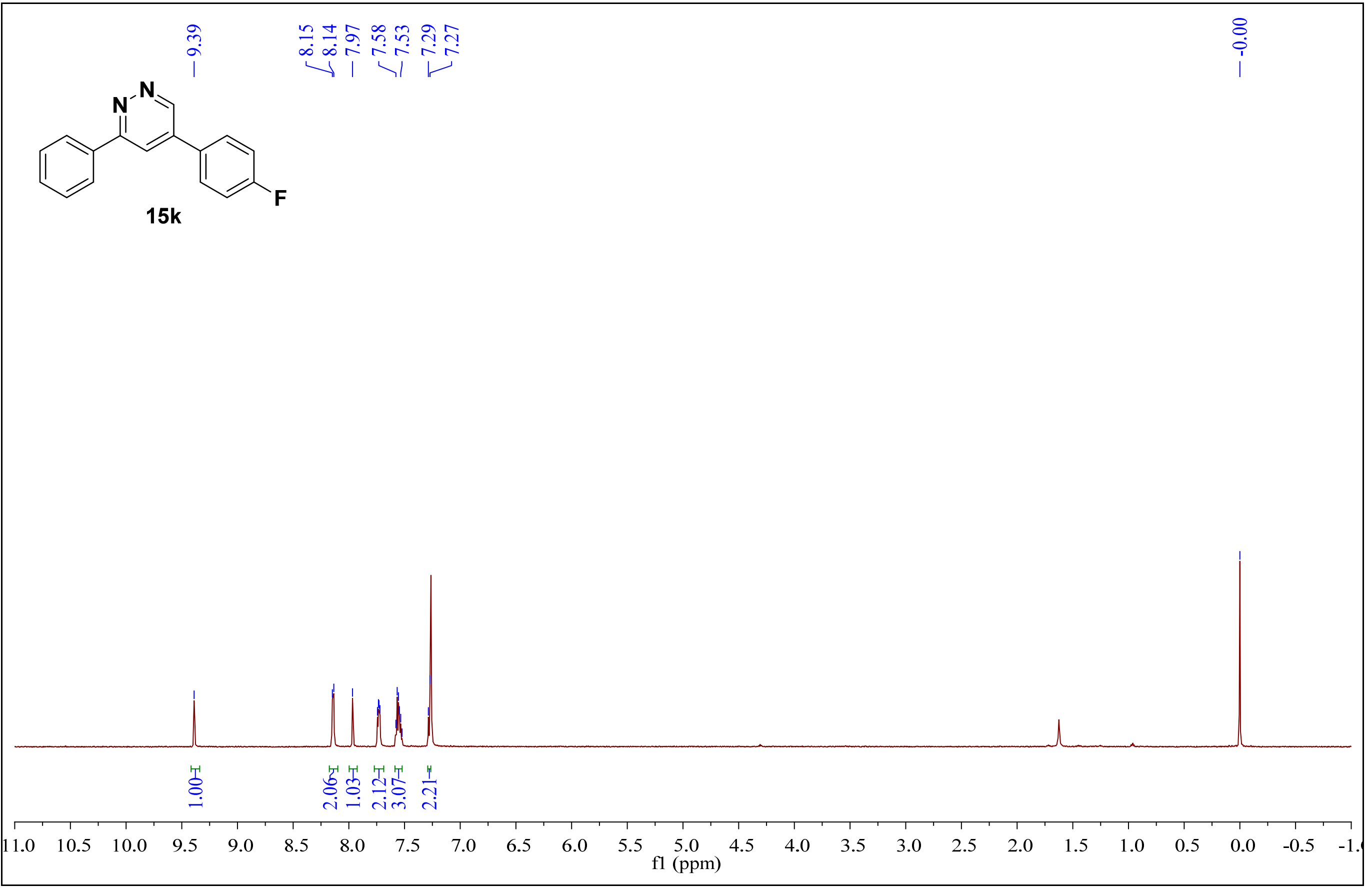

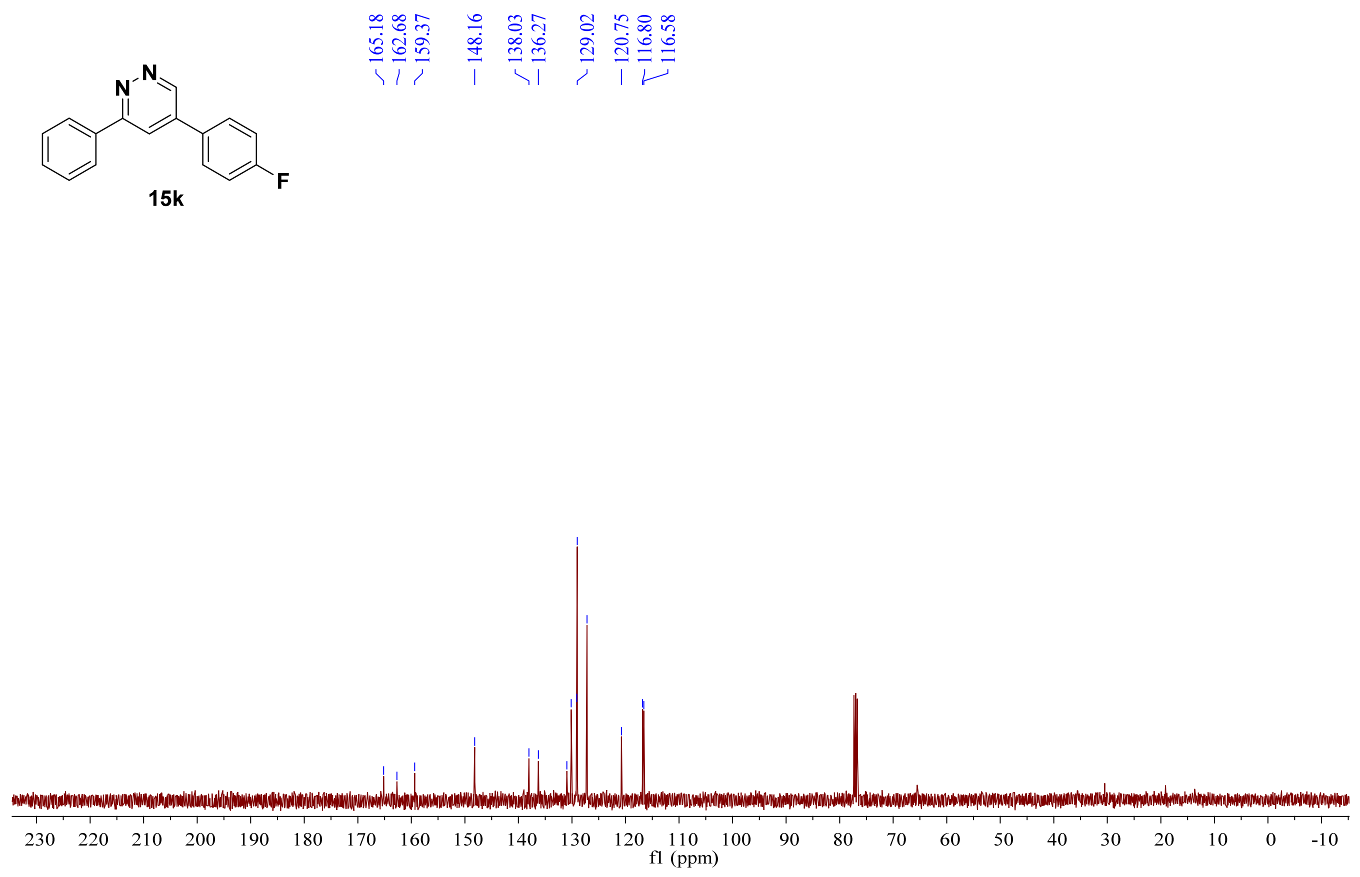

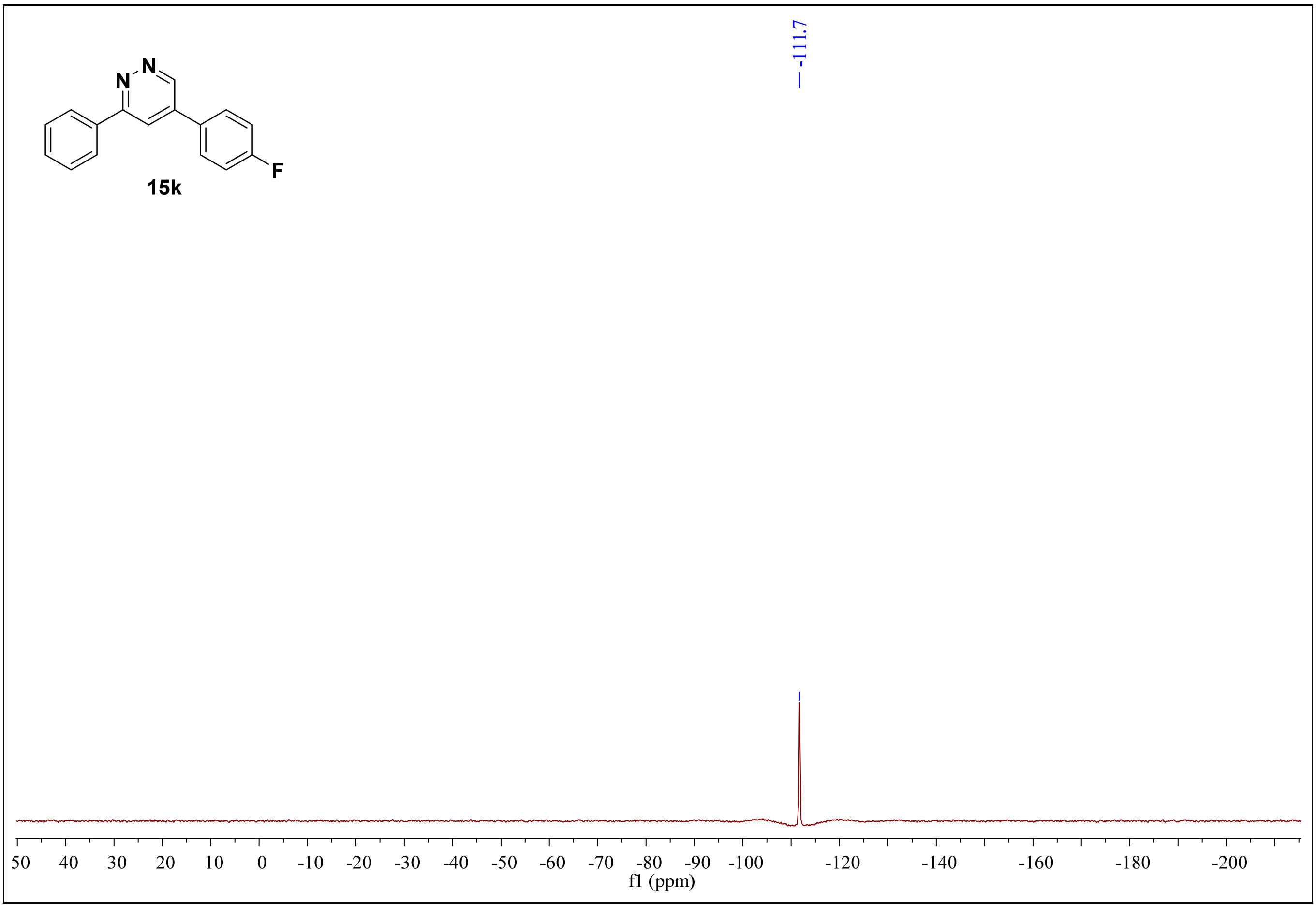
-111.7
15k
F
50 40 30 20 10 0 -10 -20 -30 -40 -50 -60 -70 -80 -90 -100 -120 -140 -160 -180 -200
f1 (ppm)

Supplementary Figure 71. **^{1}H NMR (600 MHz, $CDCl_3$) and ^{13}C NMR (100 MHz, $CDCl_3$) spectra of cyclic product 15l**

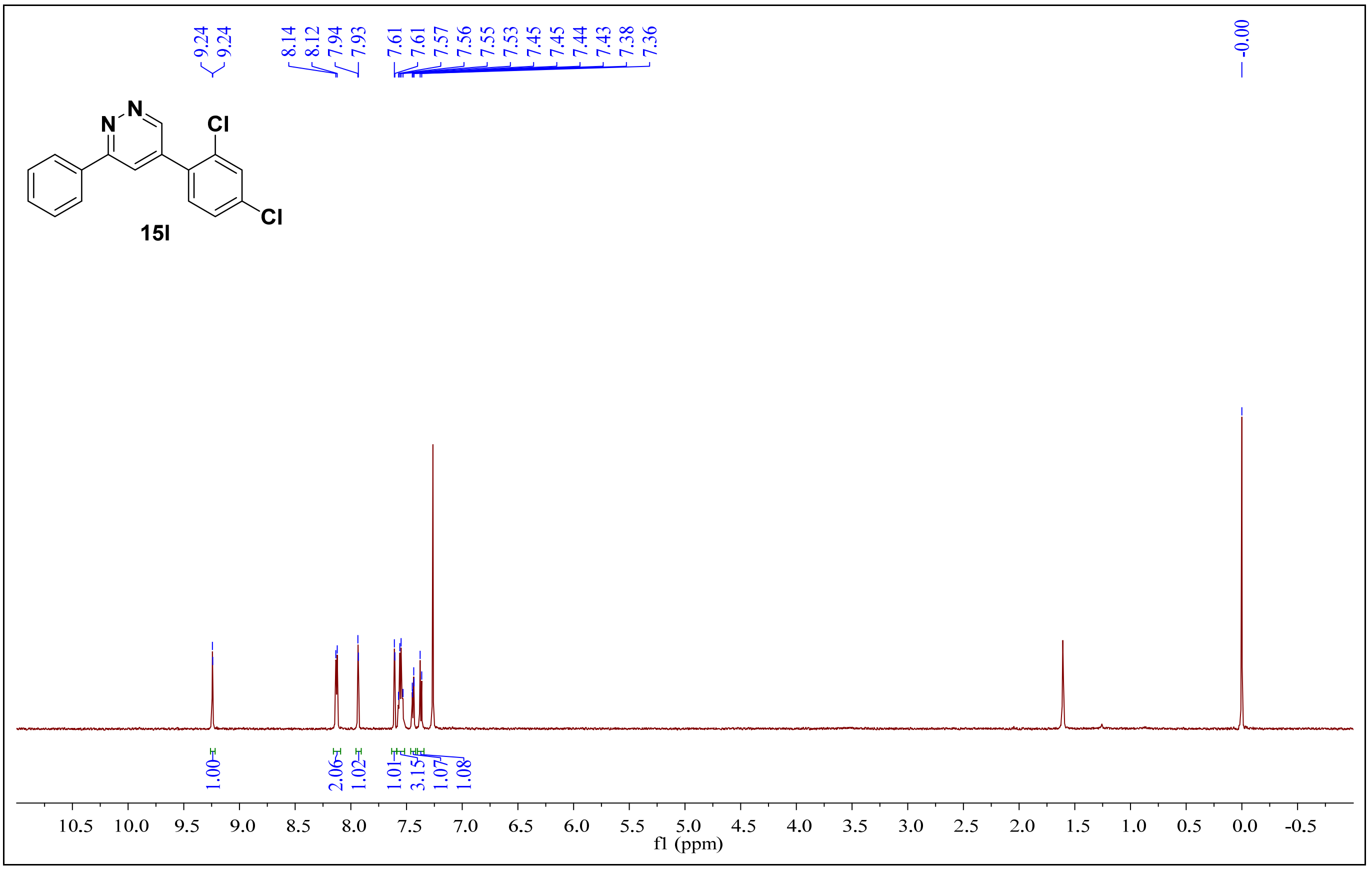

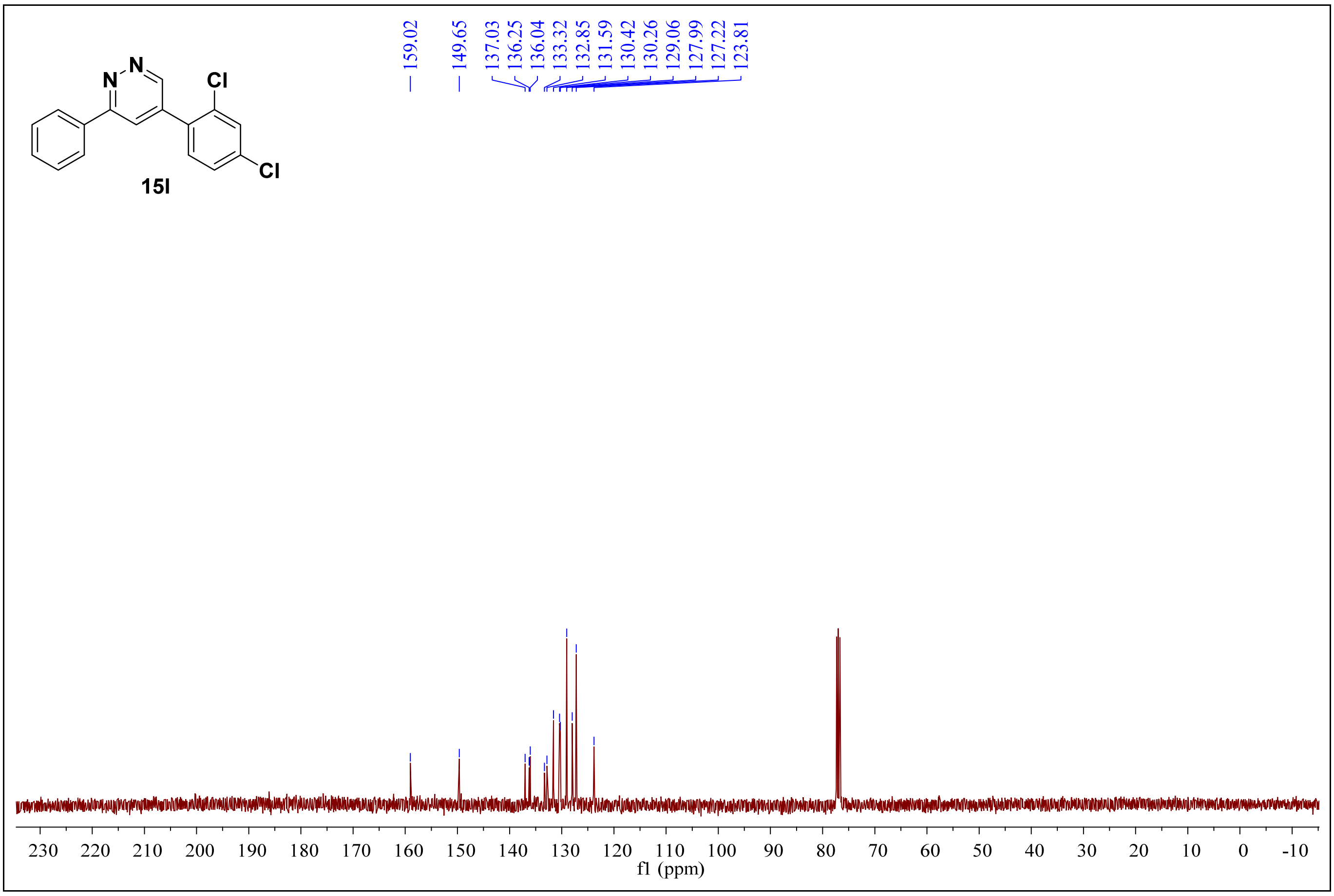

Supplementary Figure 72. **^{1}H NMR (400 MHz, DMSO-d^{6}) and ^{13}C NMR (100 MHz, DMSO-d^{6}) spectra of cyclic product 17a**

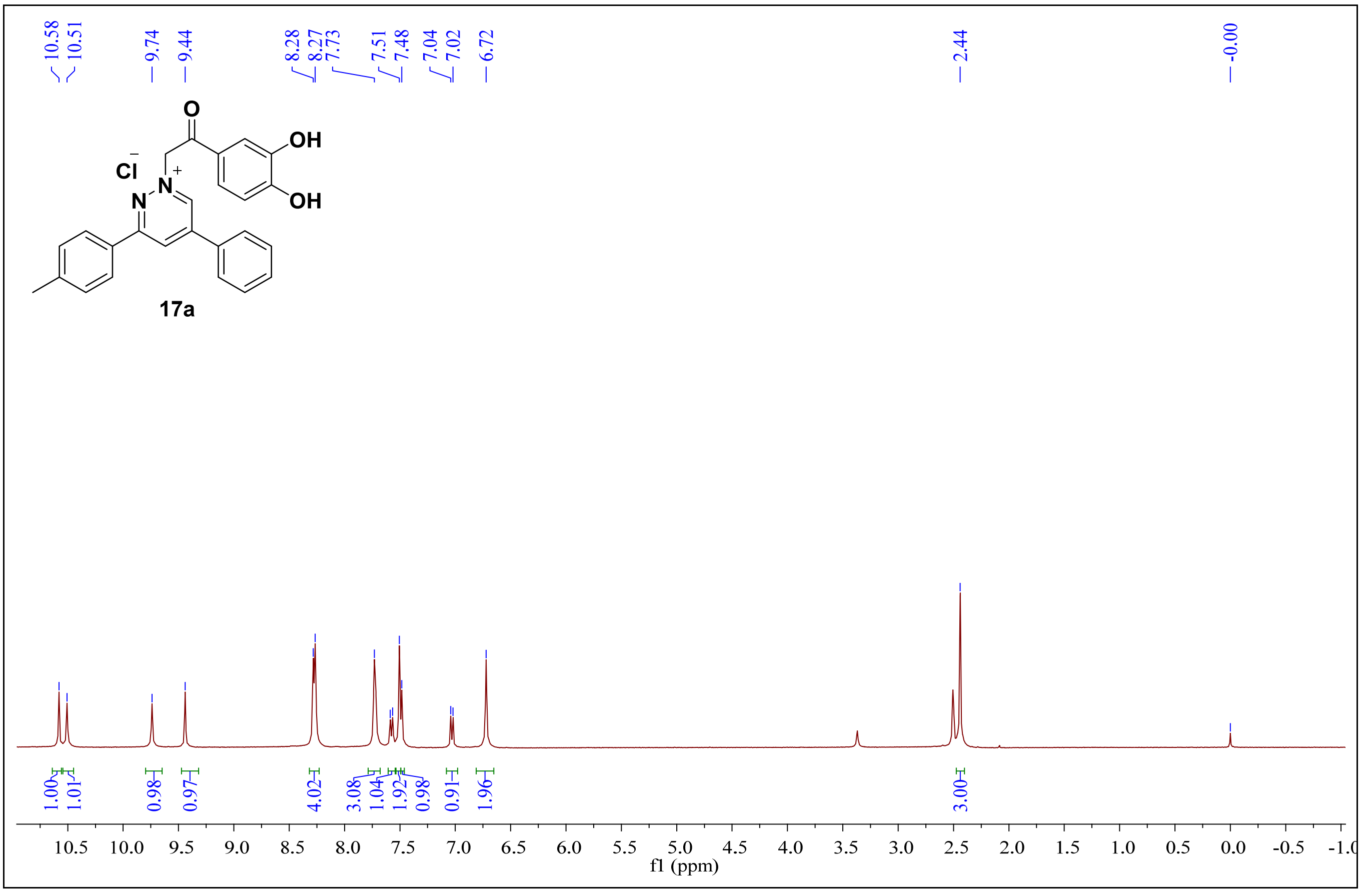

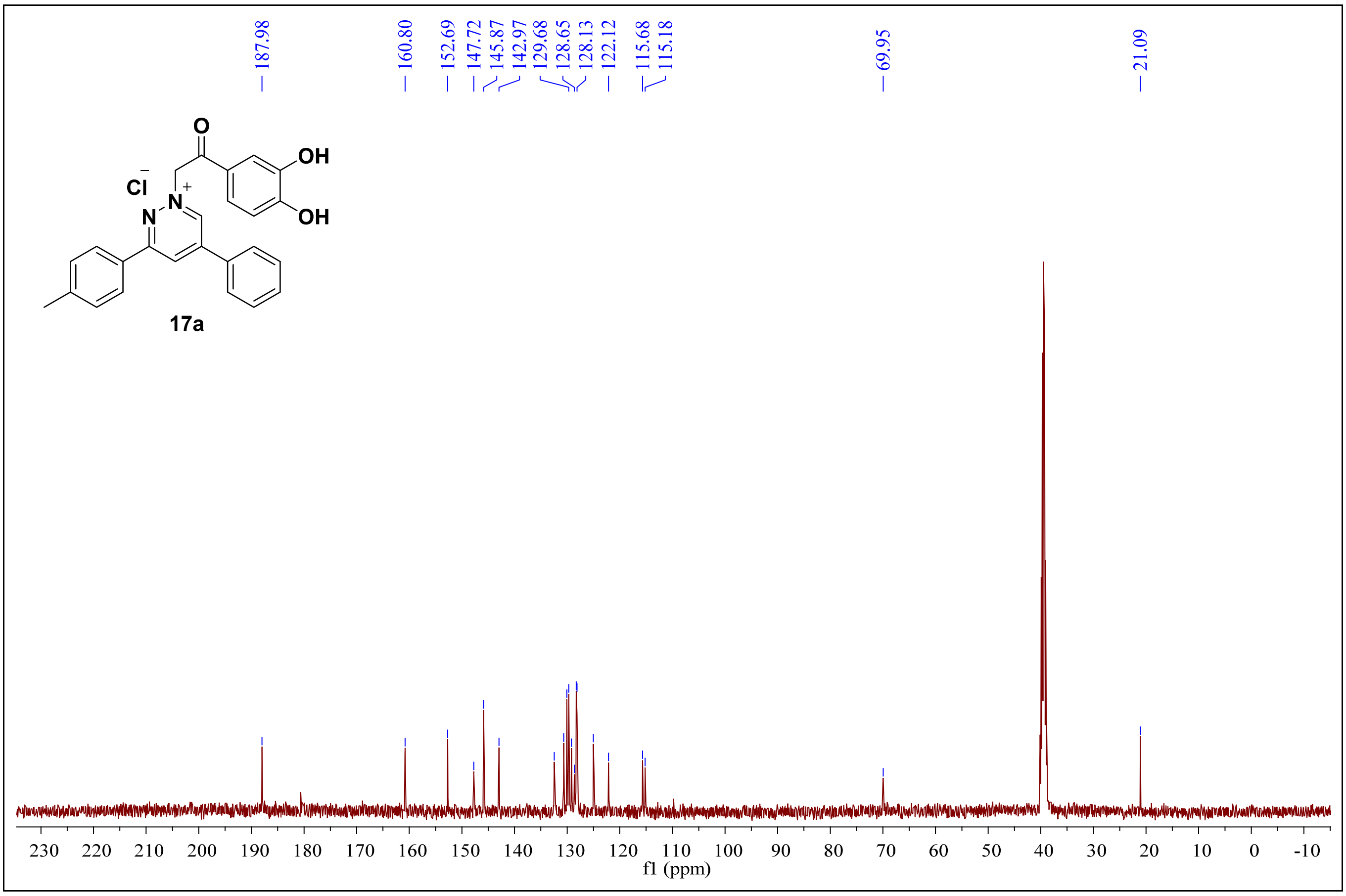

Supplementary Figure 73. ^{1}**H NMR (400 MHz, DMSO-d**^{6}**) and** ^{13}**C NMR (100 MHz, DMSO-d**^{6}**) spectra of cyclic product 17b**

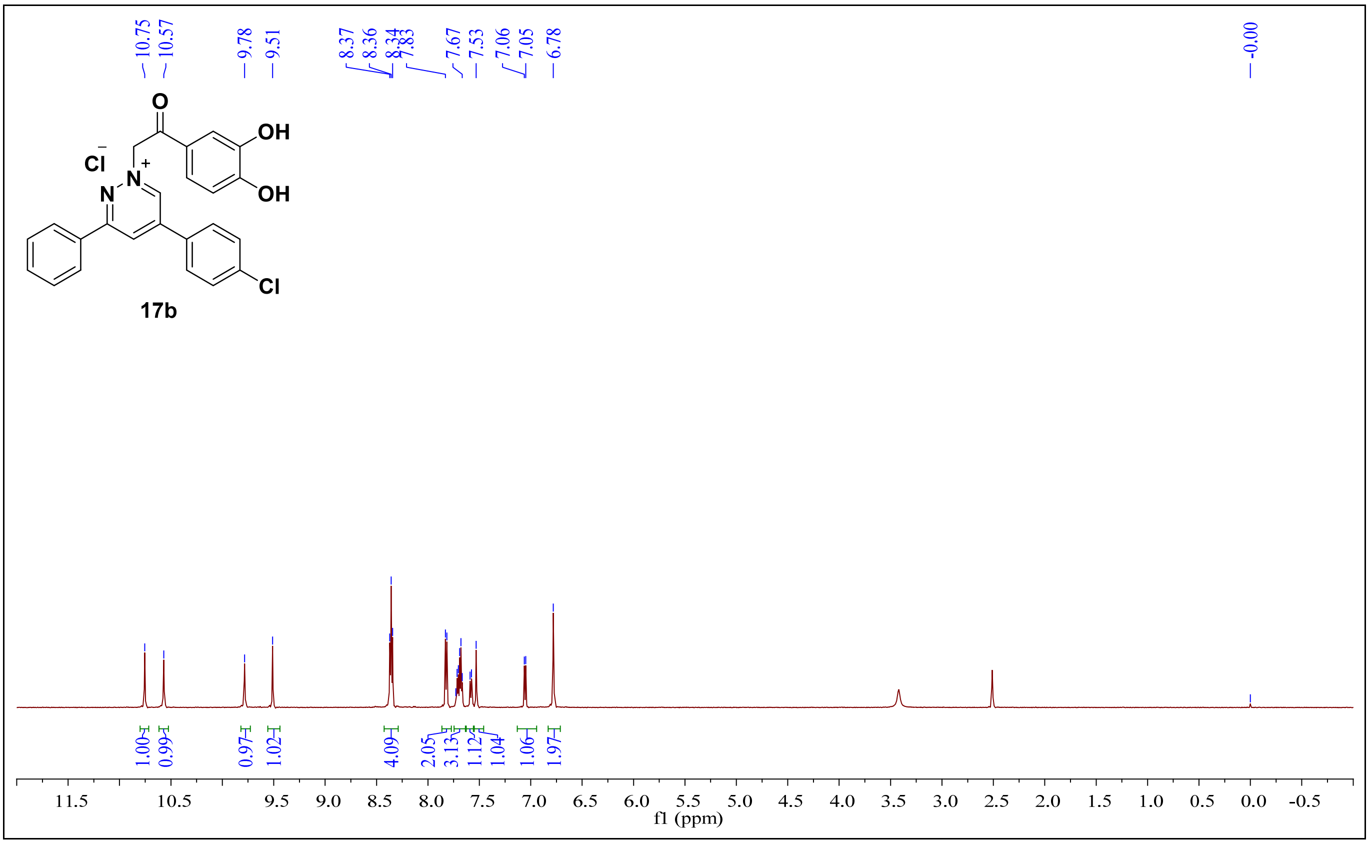

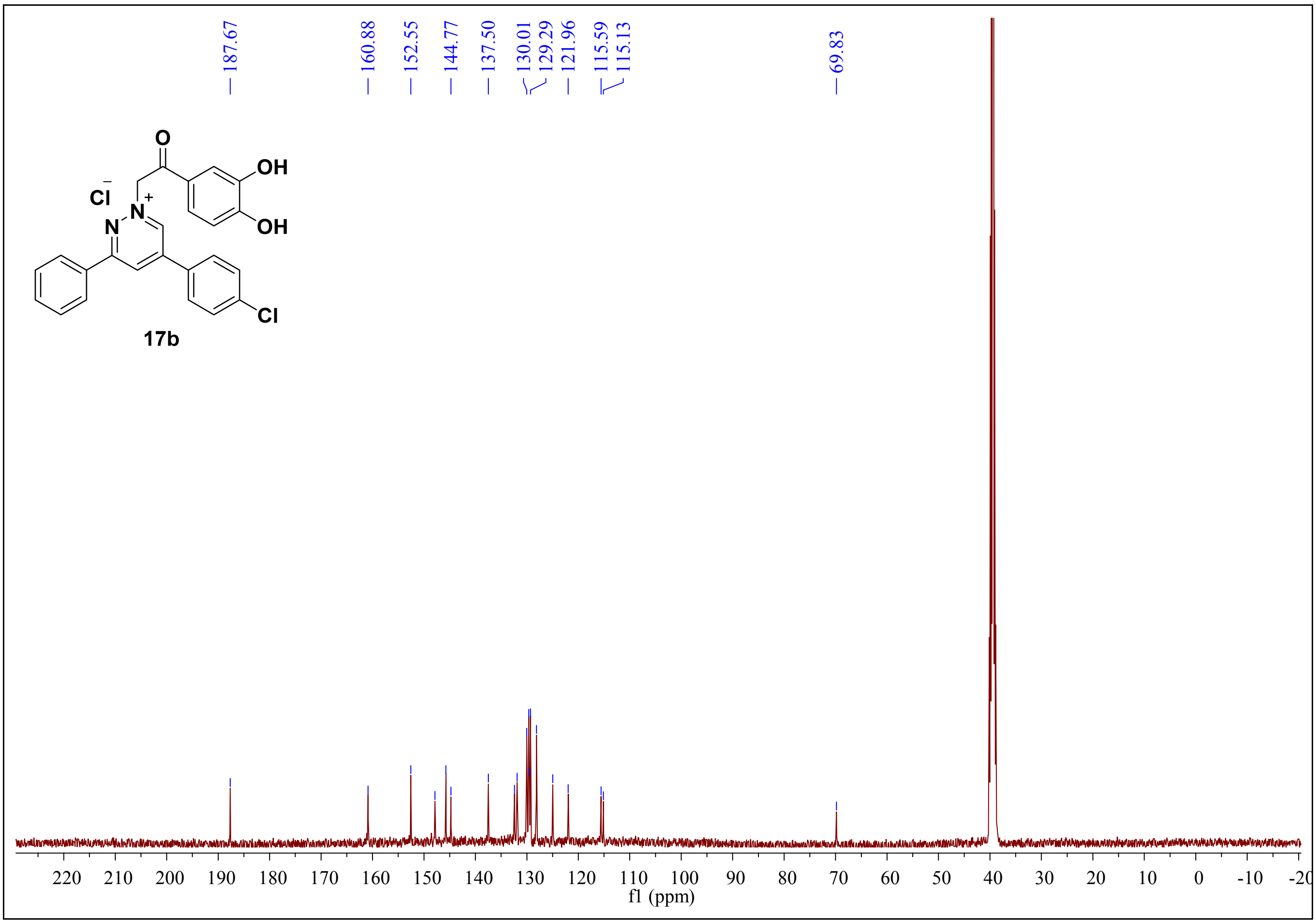

Supplementary Figure 74. ^{1}H NMR (400 MHz, DMSO-d^{6}) and ^{13}C NMR (100 MHz, DMSO-d^{6}) spectra of cyclic product 17c

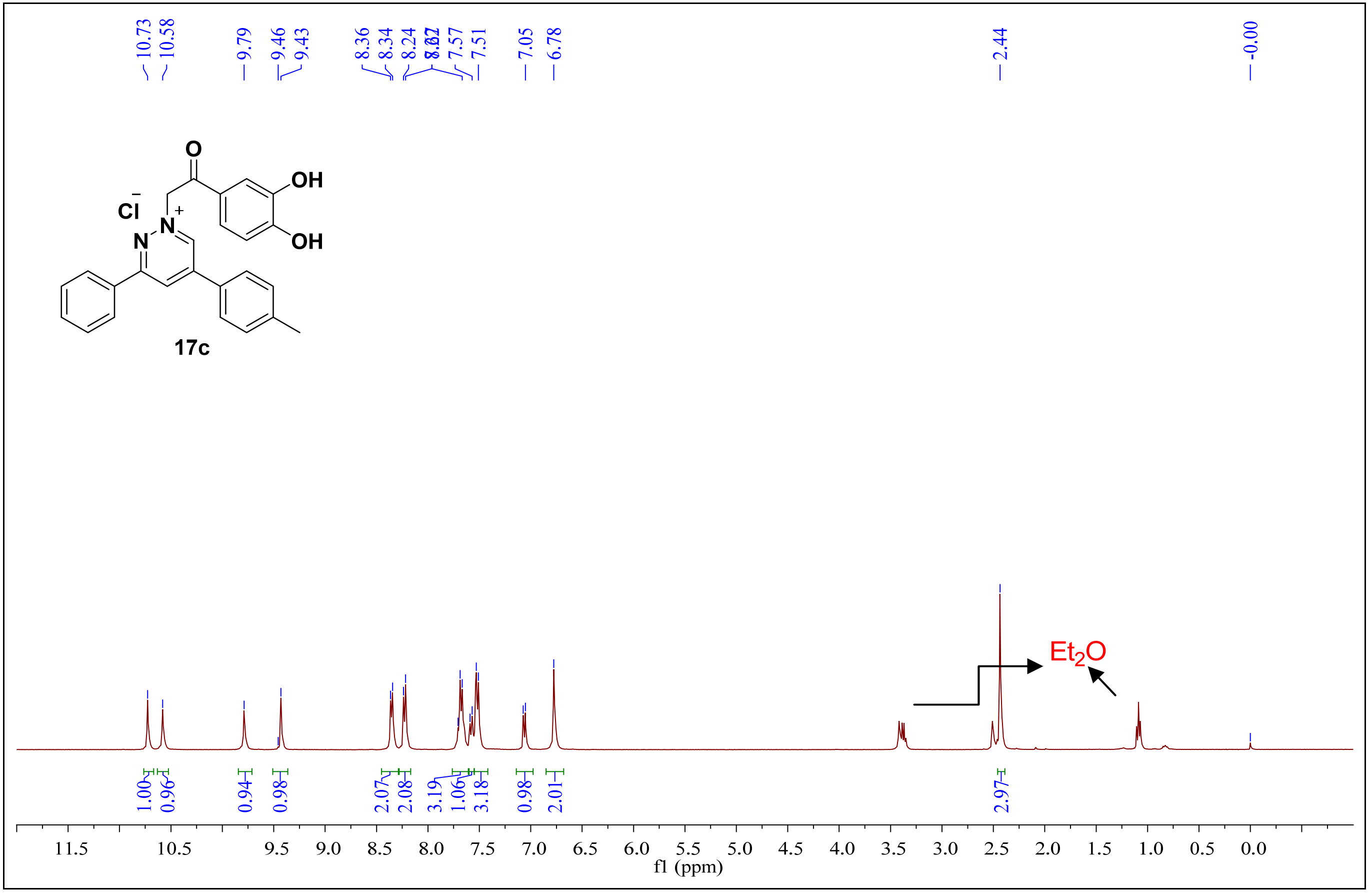

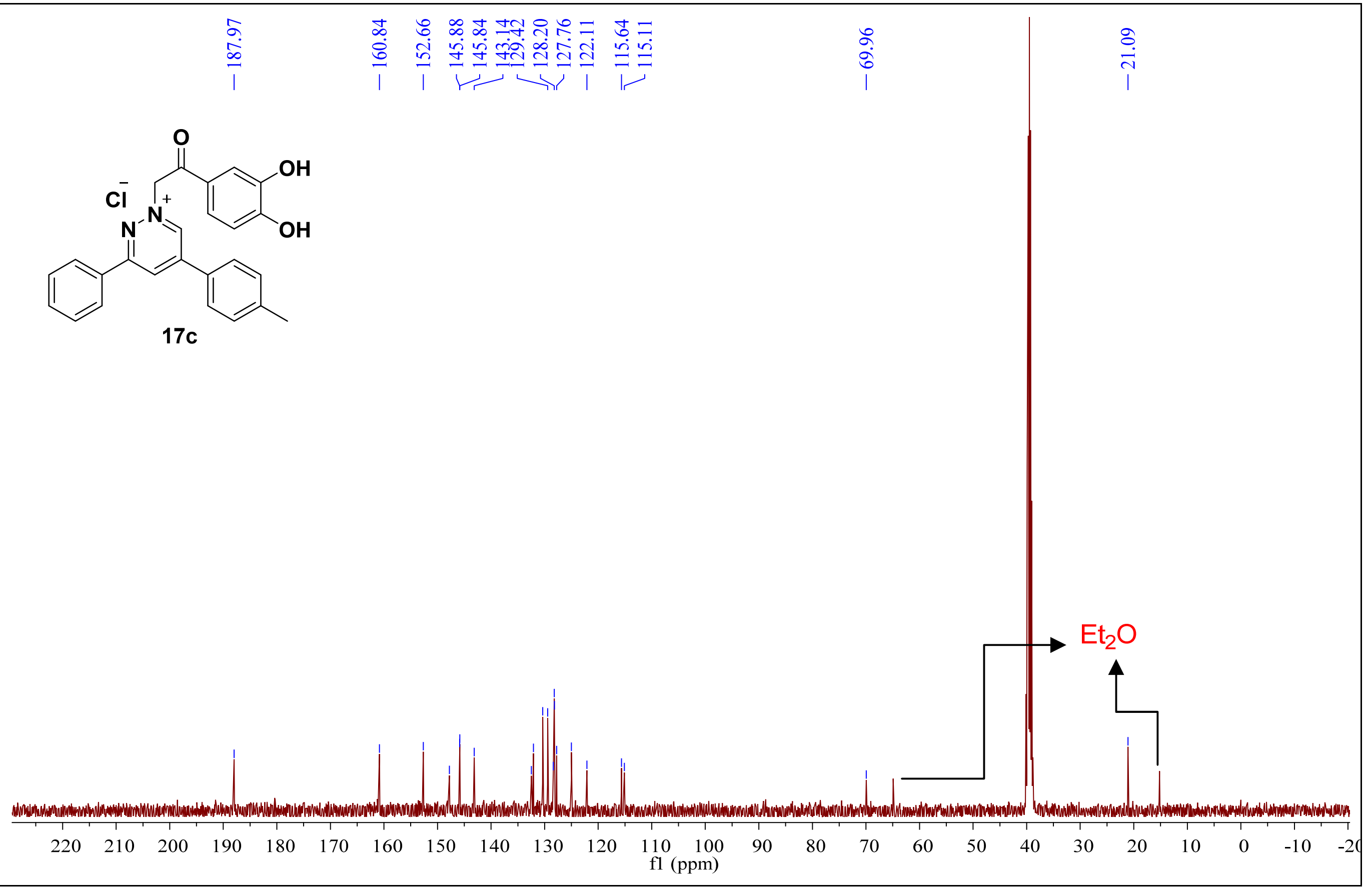

Supplementary Figure 75. **^{1}H NMR (600 MHz, DMSO-d[6]), ^{13}C NMR (100 MHz, DMSO-d[6]) and ^{19}F (376 MHz, $CDCl_3$) spectra of cyclic product 17d**

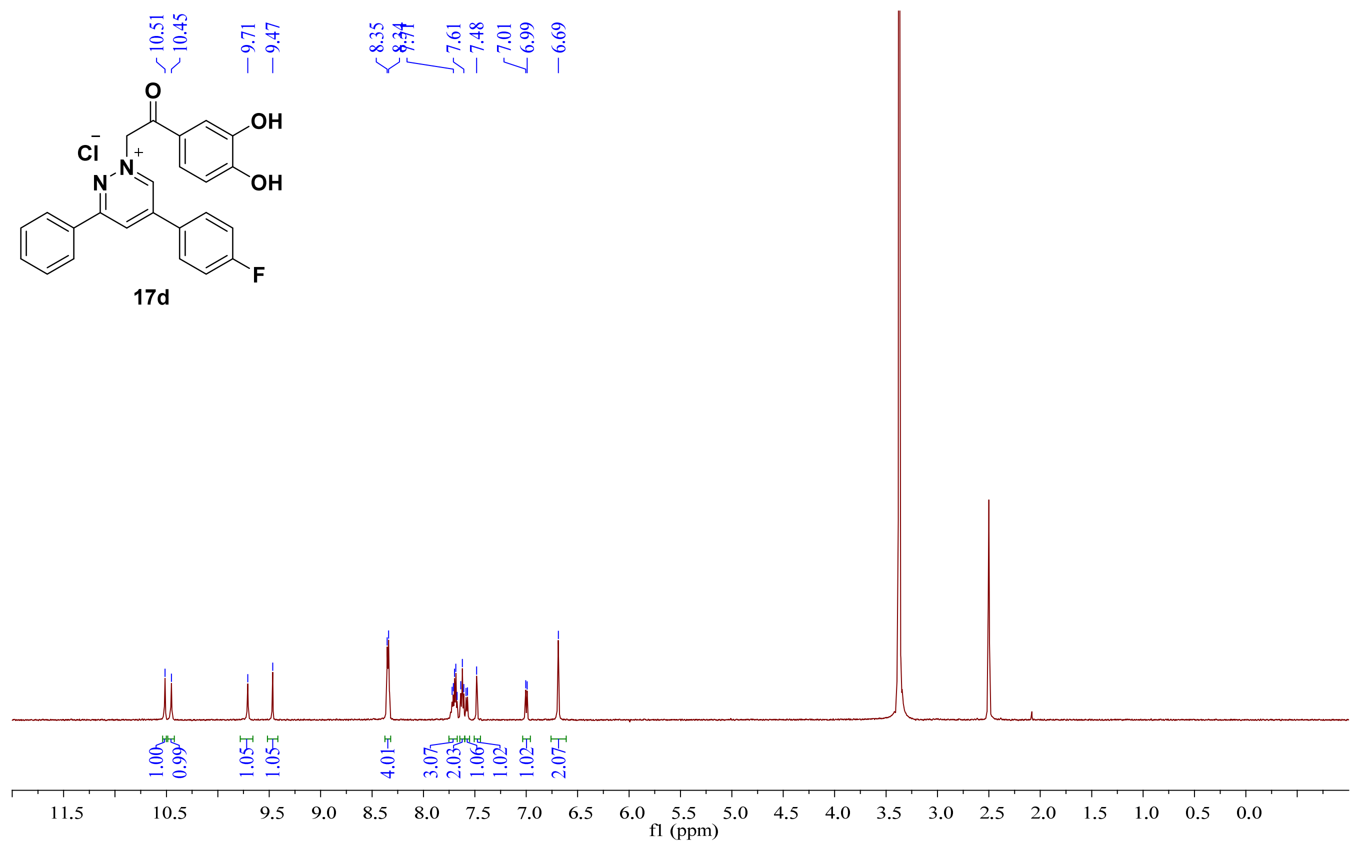

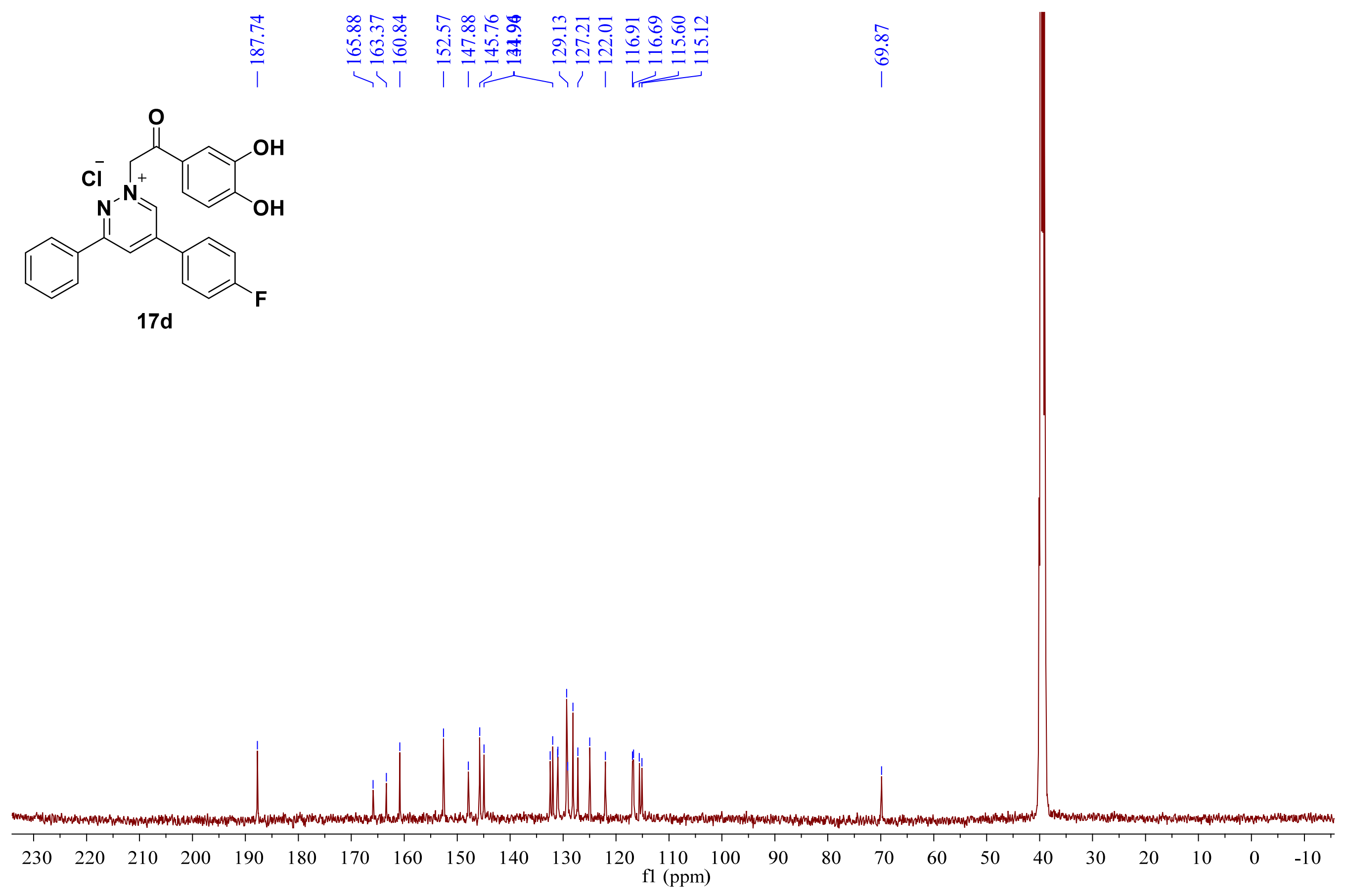

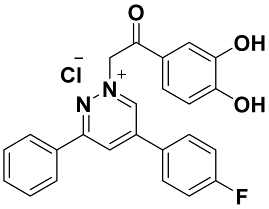
OH
OH
Cl
N
N
F

17d

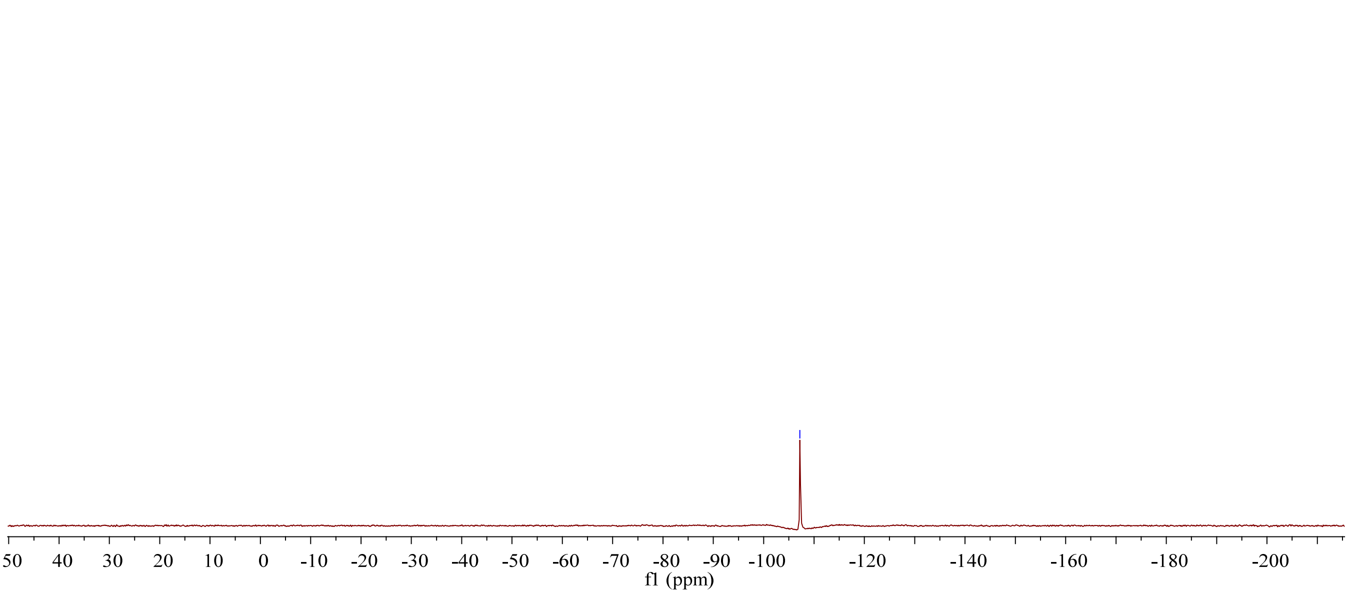
-107.2
50 40 30 20 10 0 -10 -20 -30 -40 -50 -60 -70 -80 -90 -100 -120 -140 -160 -180 -200
f1 (ppm)

Supplementary Figure 76. ^{1}H NMR (600 MHz, DMSO-d^{6}) and ^{13}C NMR (100 MHz, DMSO-d^{6}) spectra of cyclic product 17e

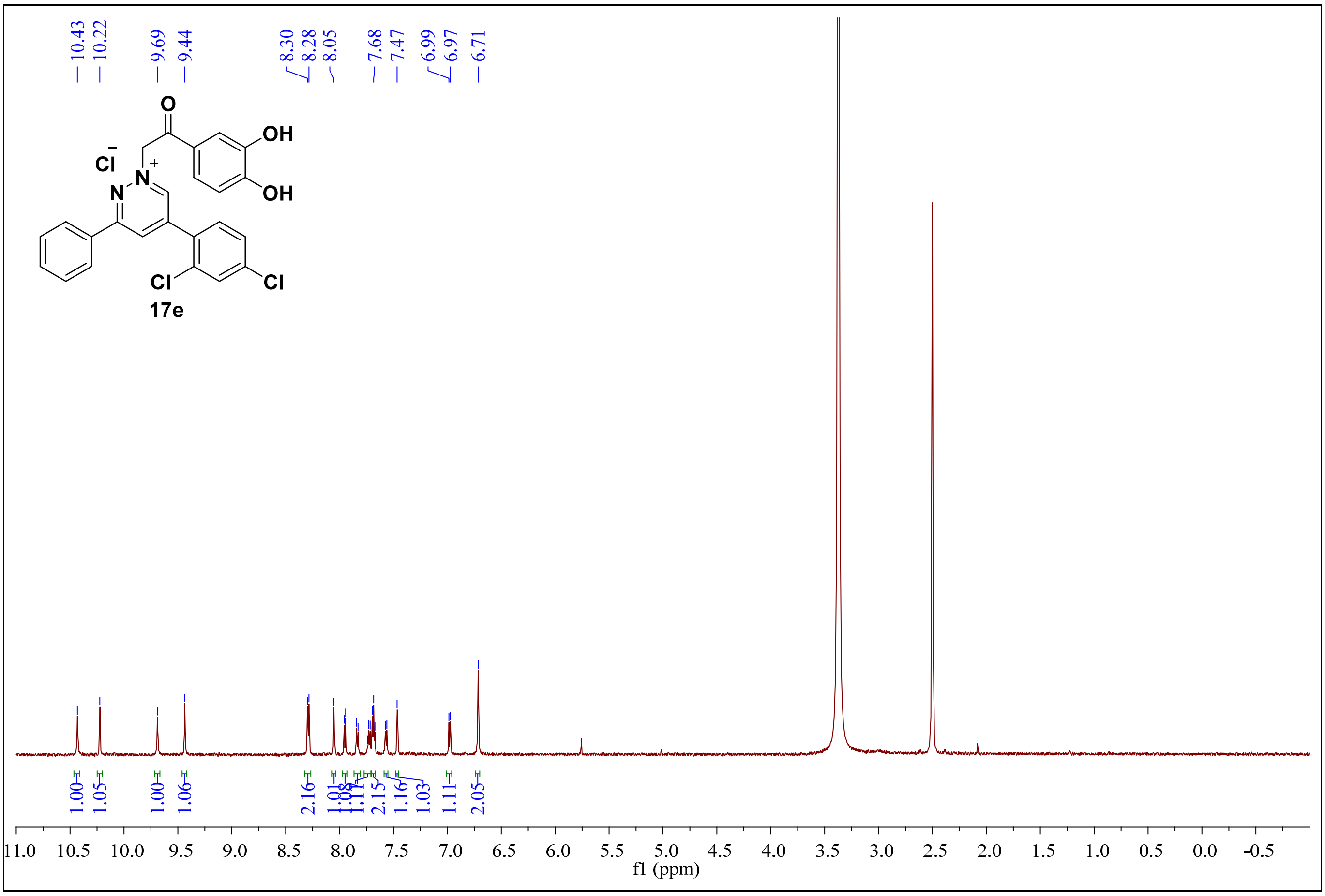

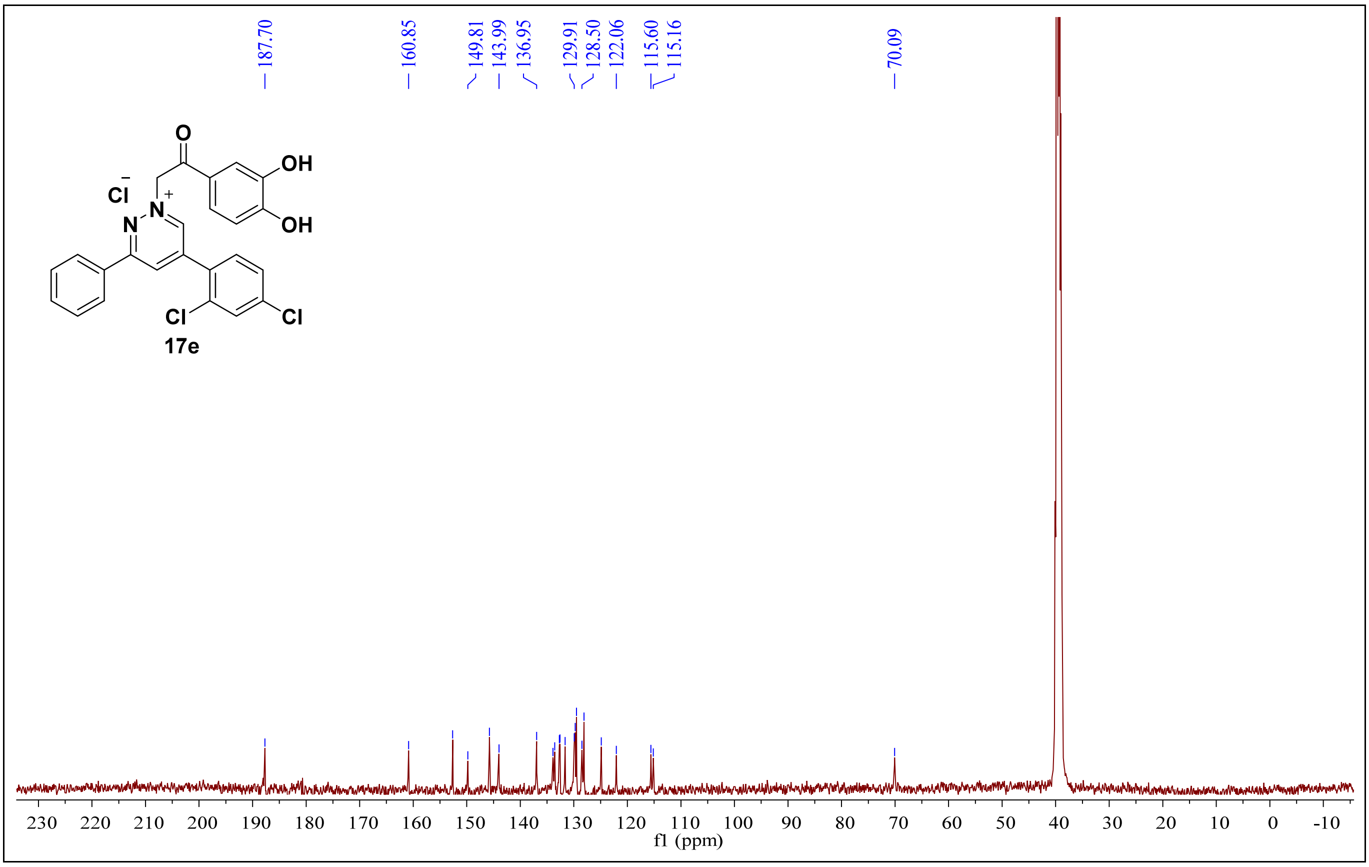

Supplementary Figure 77. ^{1}H NMR (400 MHz, $CDCl_3$) and ^{13}C NMR (100 MHz, $CDCl_3$) spectra of product 7

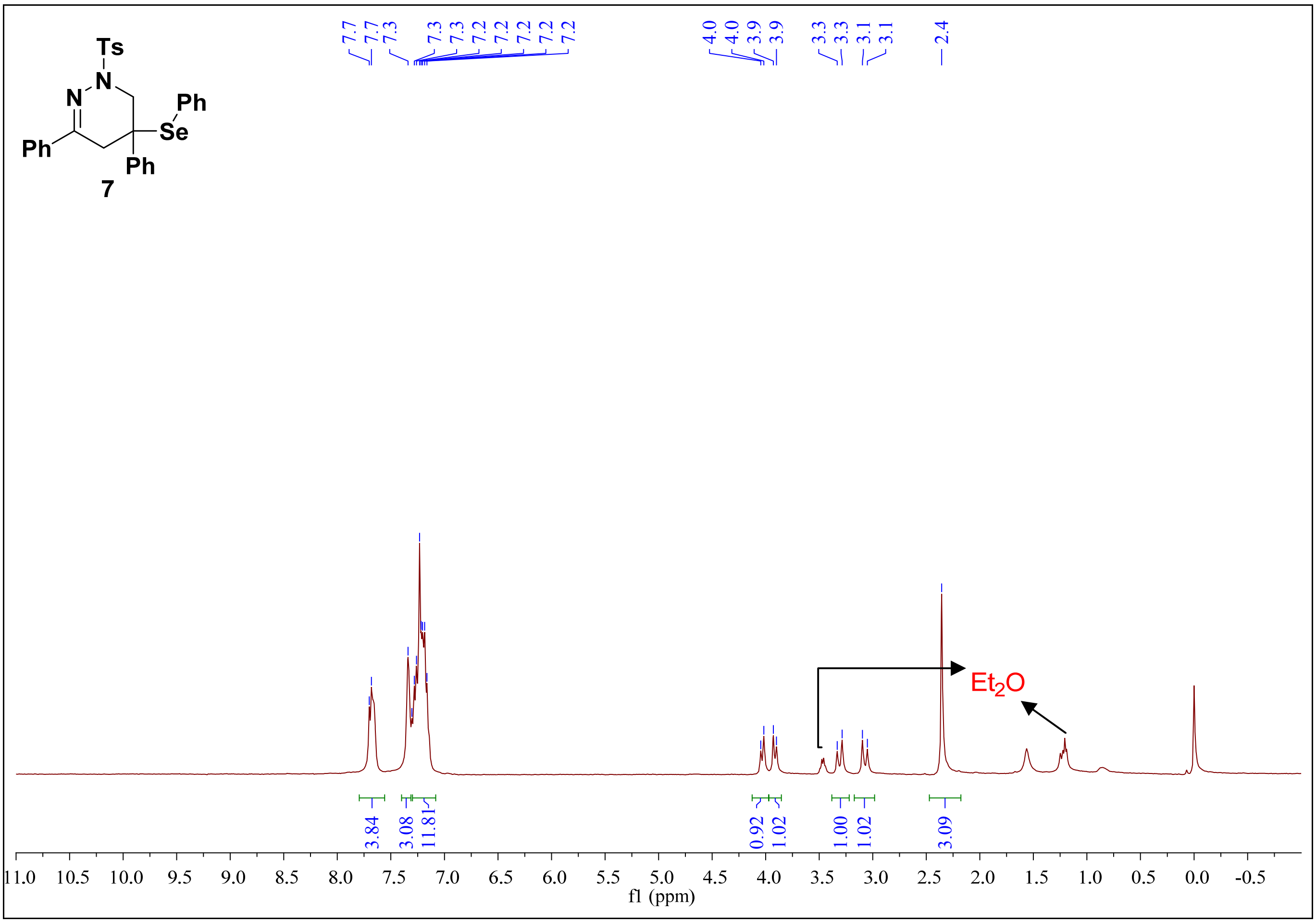

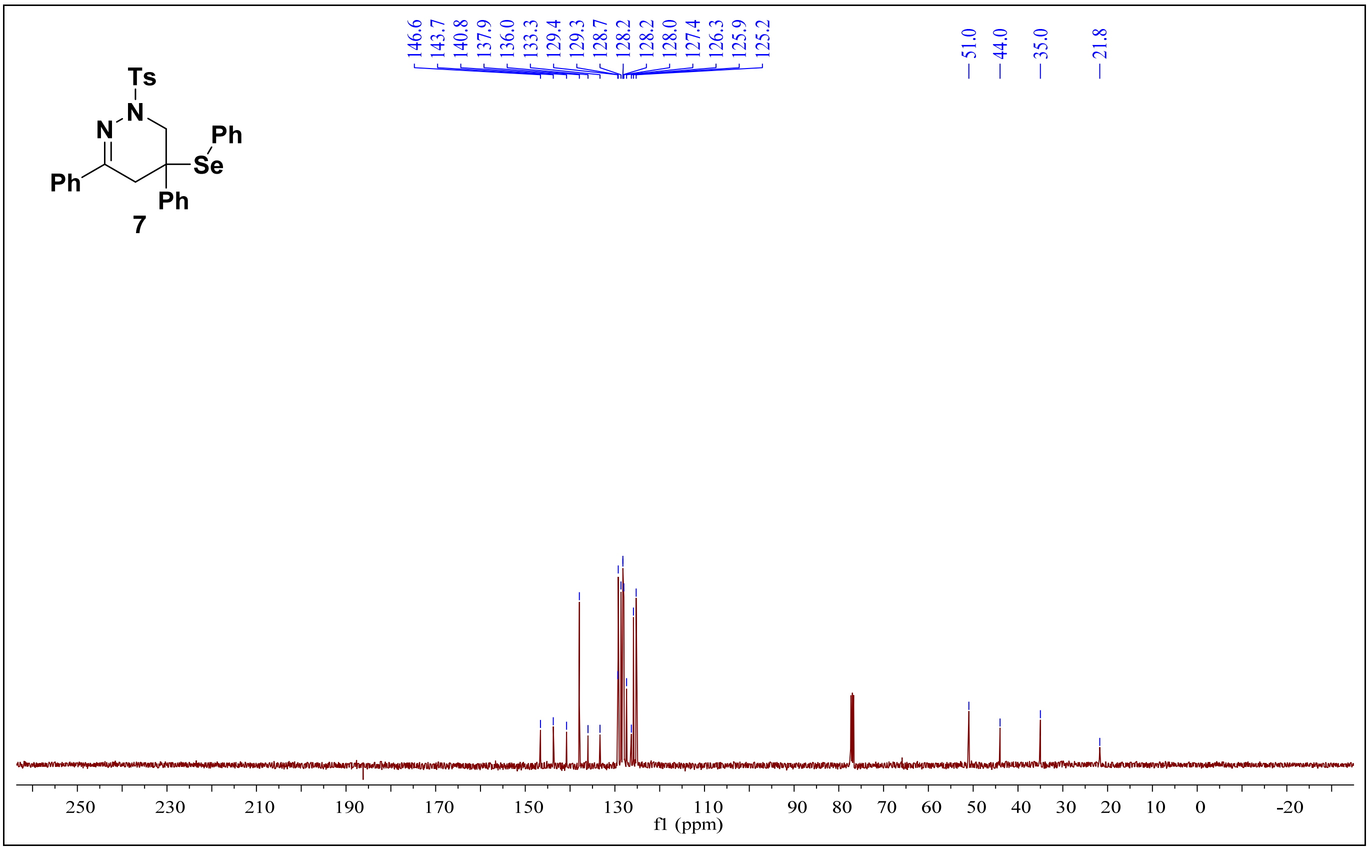

Supplementary Figure 78. ^{1}H NMR (400 MHz, $CDCl_3$) spectrum of a mixture of 2c and 7

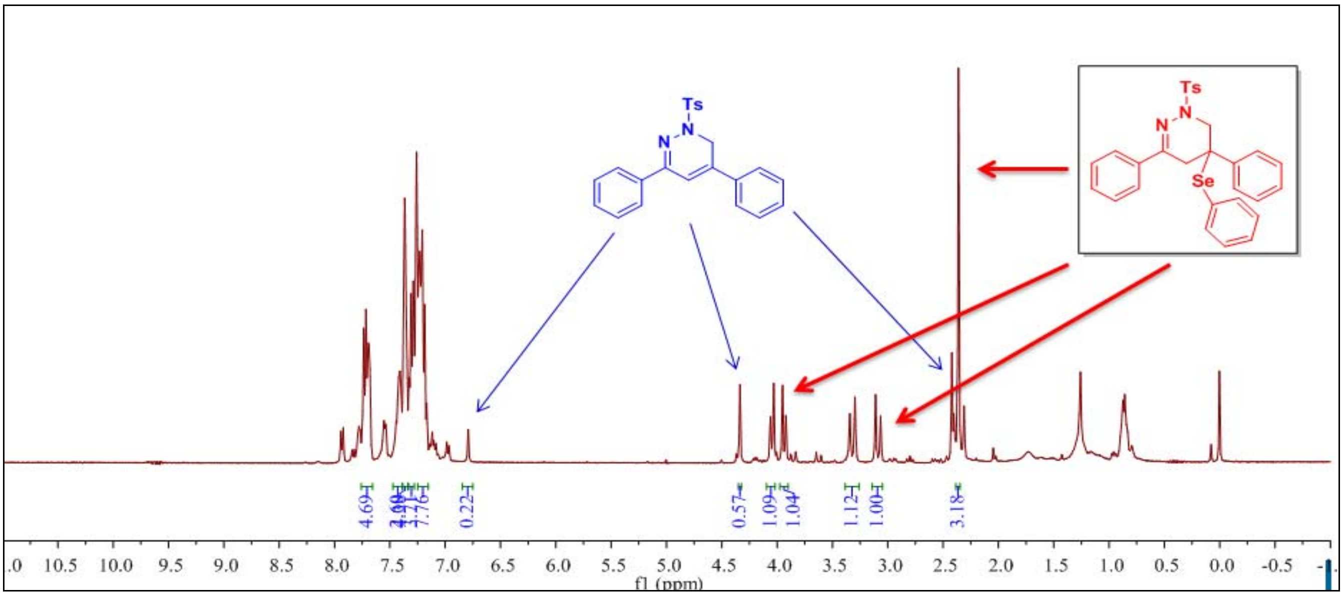

Supplementary Figure 79. X-Ray structure of compounds 2f, 4 and 14

The X-ray crystallographic coordinates for structures reported in this Article have been deposited at the Cambridge Crystallographic Data Centre (CCDC), under deposition numbers CCDC 1407651 (**2f**), 1407652 (**4**), 1407653 (**14**). These data can be obtained free of charge from The Cambridge Crystallographic Data Centre via http://www.ccdc.cam.ac.uk/data_request/cif.

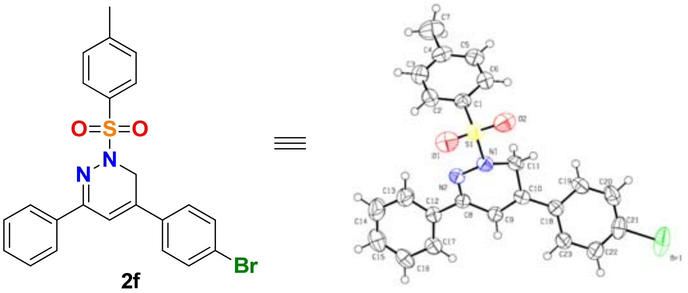

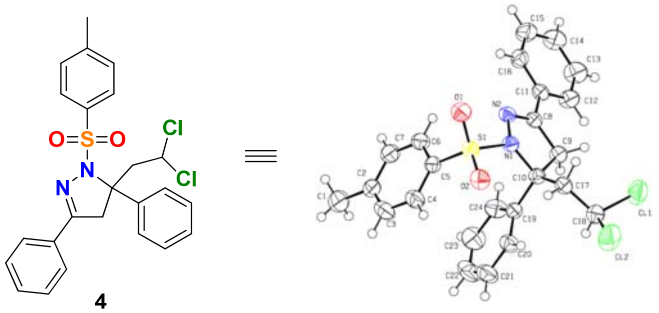

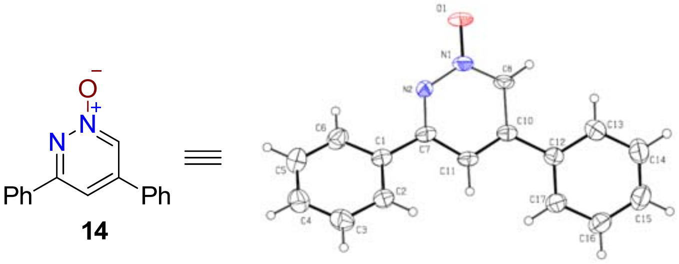

Supplementary Figure 80. HRMS analysis of reaction mixture upon about 50% conversion of model substrate 1c

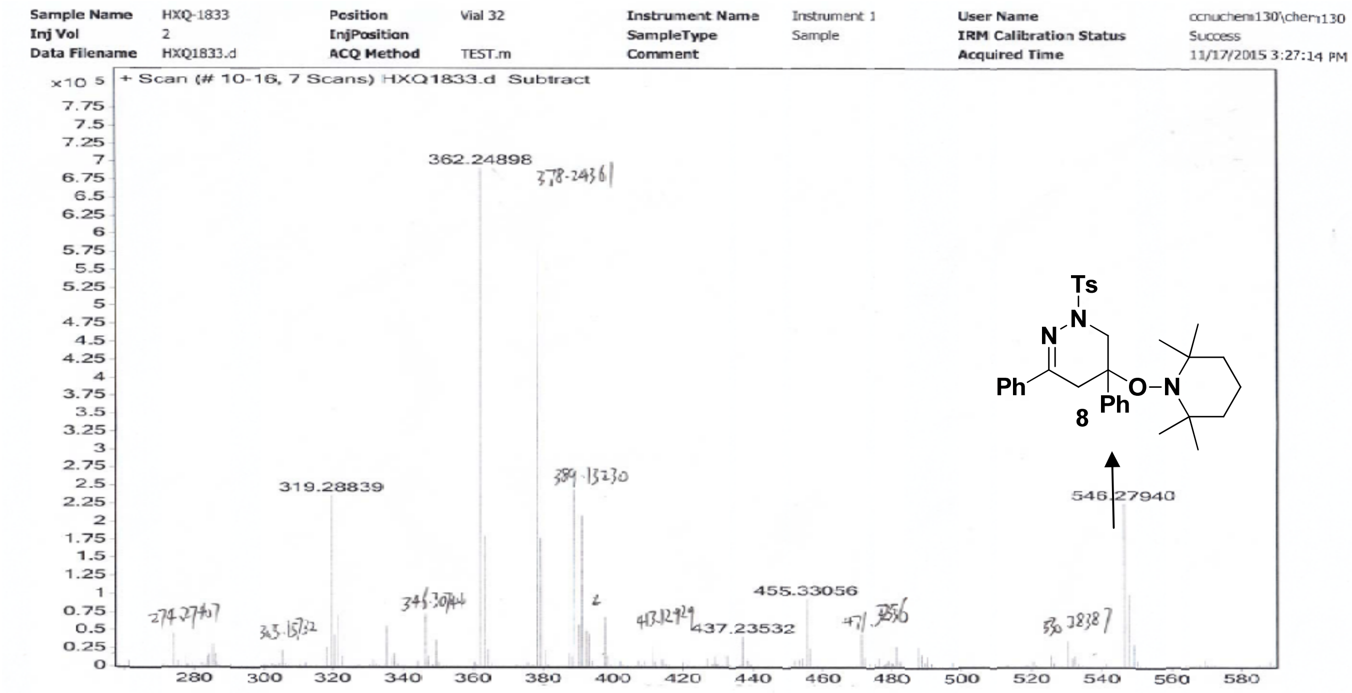

From the HRMS spectra, we can observe the intermediate 8. Unfortunately, all the attempts to isolate the possible intermediate 8 met failure. Therefore, at present we cannot rule out the carbon radical recombination/elimination pathway.

Supplementary Figure 81. Calculation studies on the role of TEMPO

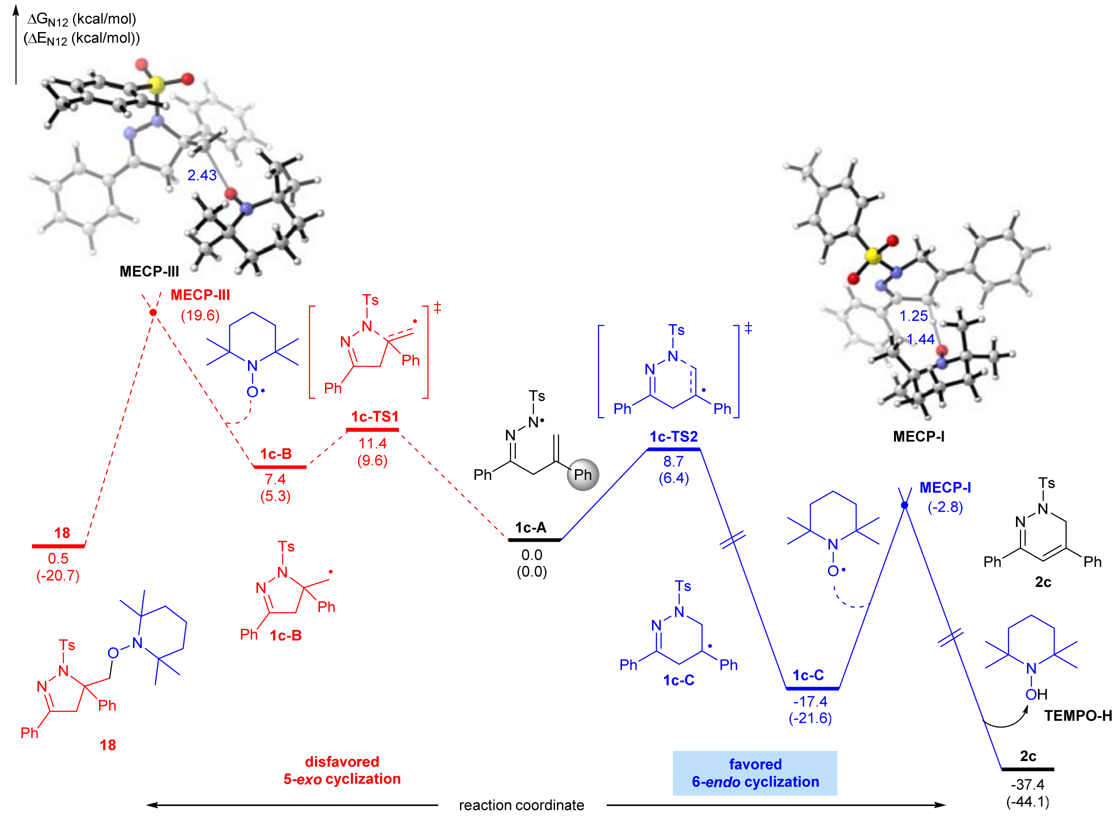

As shown in supplementary Fig 81 (red line), the energy barrier (ΔE) of MECP-III is 14.3 kcal/mol, and the formation of TEMPO-adduct **18** is endergonic by 0.5 kcal/mol (ΔG). For the combination of carbon radical **1c-C** with TEMPO, the energy barrier (ΔE) is 18.8 kcal/mol, and the formation of **2c** is exergonic by 20.0 kcal/mol compared with intermediate **1c-C**. These data indicate that the radical **1c-B** would be easily trapped by TEMPO to give the corresponding adduct **18** if it is generated from the intermediate **1c-A** via **1c-TS1** in the presence of TEMPO. However, it should be noted that the free energy barrier for the generation of radical **1c-C** is 2.7 kcal/mol lower than that of **1c-B**. Therefore, the 5-*exo* cyclization pathway through intermediate **1c-B** appears to be shut down in the presence of TEMPO, which is also in accordance with the experimental results.

Supplementary Figure 82. Luminescence quenching experiments with 1c

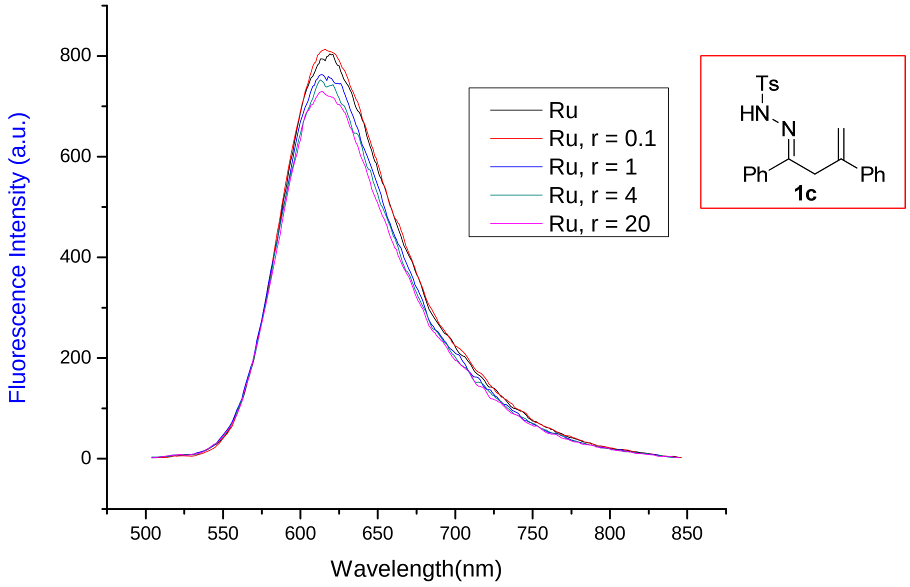

Supplementary Figure 83. Luminescence quenching experiments with 1c in the presence of base

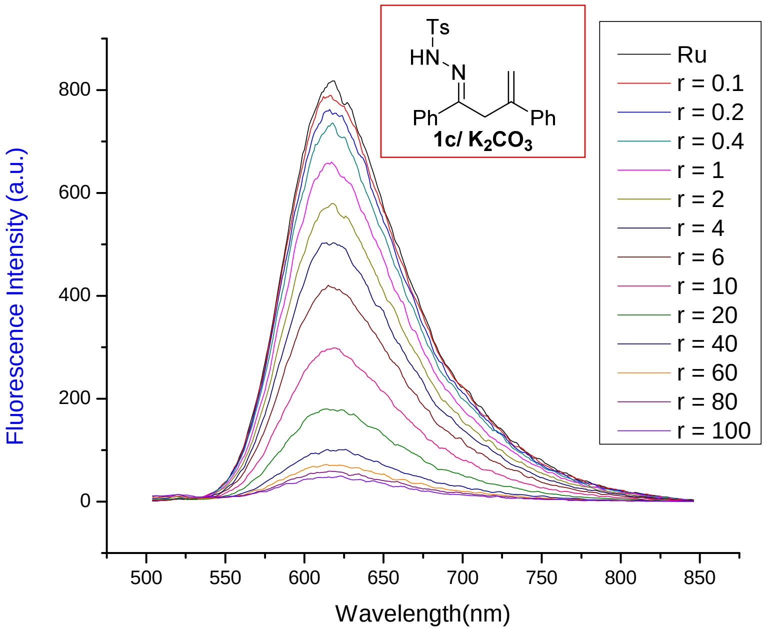

Fluorescence spectra were collected on Cary Eclipse Fluorescence Spectrophotometer. All Ru(bpy)$_3$Cl$_2$·6H$_2$O solutions were excited at 452 nm and the emission intensity at 616 nm was observed. In a typical experiment, the emission spectrum of a 1×10^{-5} M solution of Ru(bpy)$_3$Cl$_2$·6H$_2$O in DMF was collected. The decrease of Ru(bpy)$_3^{2+}$ luminescence couldn't be observed in the presence of substrate **1c** (Figure S3). Under basic condition (K_2CO_3 in DMF), a significant decrease of Ru(bpy)$_3^{2+}$ luminescence was successfully observed in the presence of **1c** (Figure S4).

These results suggested that it was the nitrogen anion of β,γ-unsaturated hydrazone that quenched the excited state photocatalyst *Ru(bpy)$_3^{2+}$.

Supplementary Figure 84. ^{1}H NMR Study on the effect of base with 1c

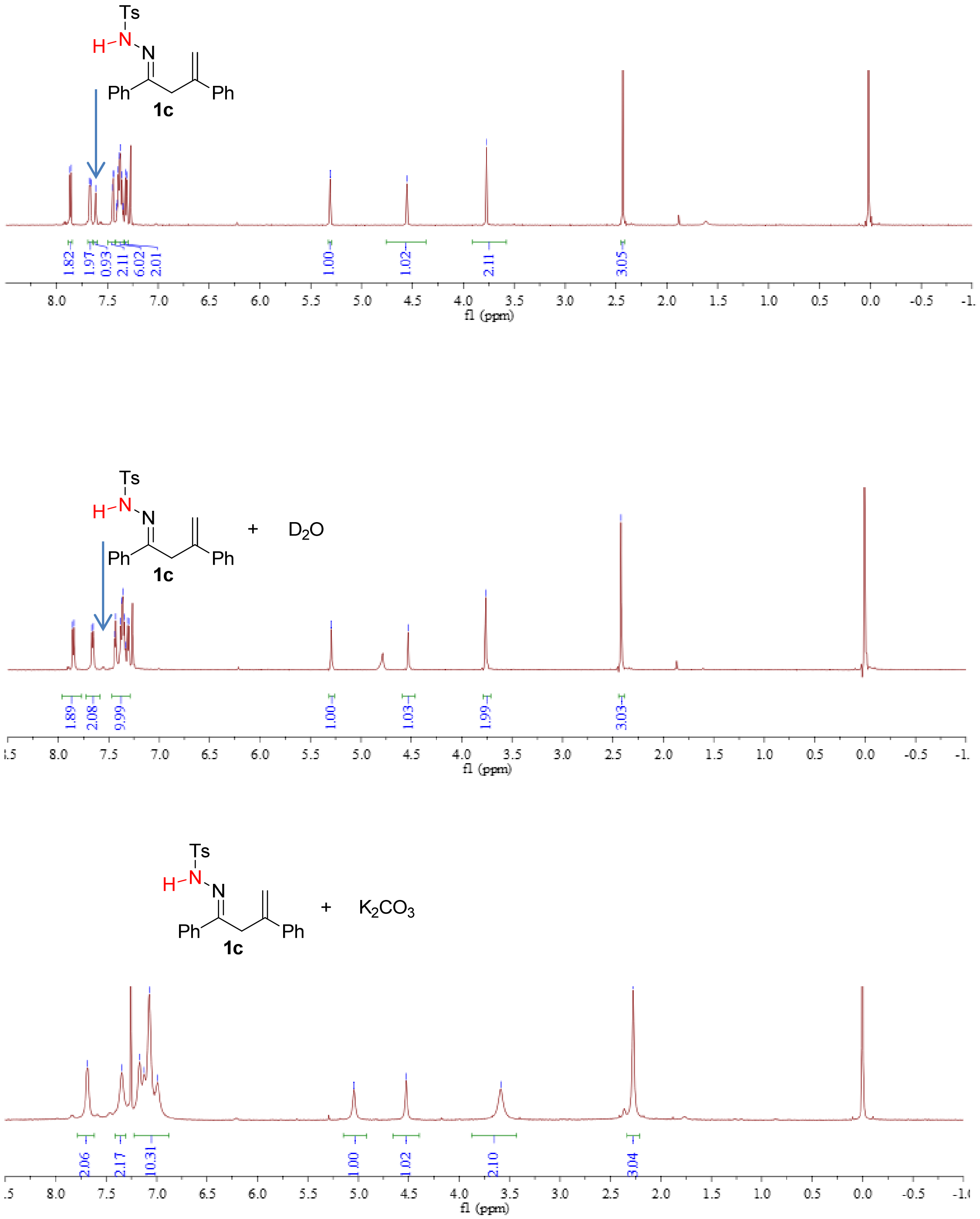

Under the basic condition (K_2CO_3 in $CDCl_3$), the signal of N-H disappeared in proton nuclear magnetic resonance (^{1}H NMR) spectra after stirring at room temperature for 30 min (Figure S2).

These results suggested that the K_2CO_3 is able to abstract the proton of N-H bond of β,γ-unsaturated hydrazone 1c to generate the corresponding nitrogen anion intermediate.

Supplementary Figure 85. Blank experiment-CV of 1c

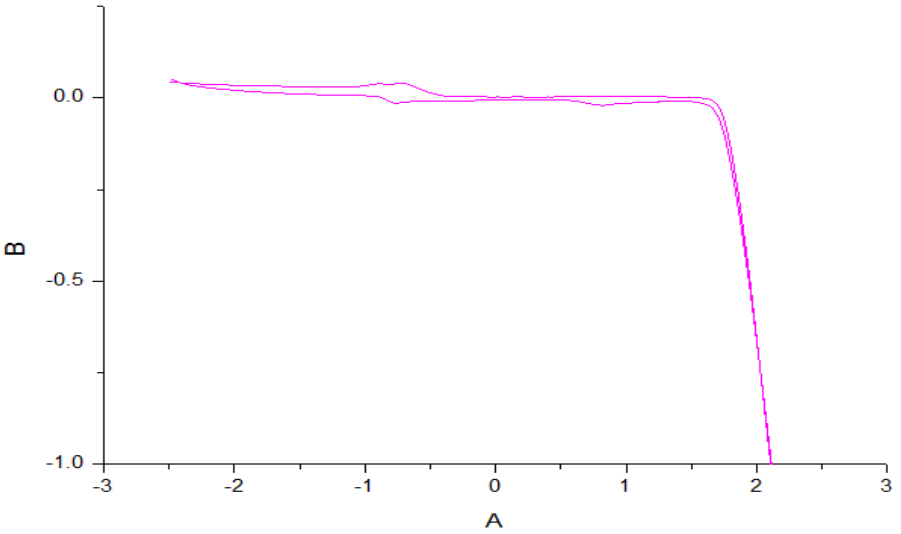

Supplementary Figure 86. CV of compound 1c under the basic condition

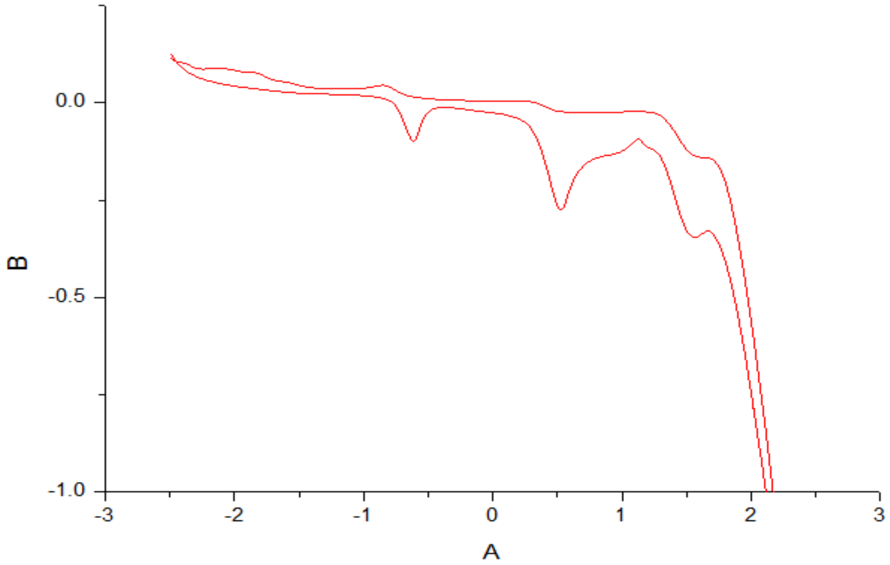

Electrochemical analysis and CV: Cyclic voltammetry (CV) was taken using a CHI660D potentiostation. Measurement of **1c** was carried out in 0.1 M of Bu_4NPF_6/DMF at a scan rate of 100 mV/s. The working electrode is a glassy carbon, the counter electrode is a Pt wire, and the reference electrode is SCE.

Measurement of **1c**, which was stirred at room temperature for 30 min under the basic condition (K_2CO_3 in DMF), was carried out in 0.1 M of Bu_4NPF_6/DMF at a scan rate of 100 mV/s. The working electrode is a glassy carbon, the counter electrode is a Pt wire, and the reference electrode is SCE.

These results suggested that the excited state photocatalyst $*Ru(bpy)_3^{2+}$ ($E_{1/2}*^{II/I}$ = +0.77 V vs SCE in CH_3CN) is likely to be sufficiently oxidizing to oxidize the nitrogen anion 1c' (E_p^{red} = 0.56 V vs SCE) to generate the corresponding N-centred radical intermediate.

Supplementary Tables

Supplementary Table 1. Screening of other oxidants[a]

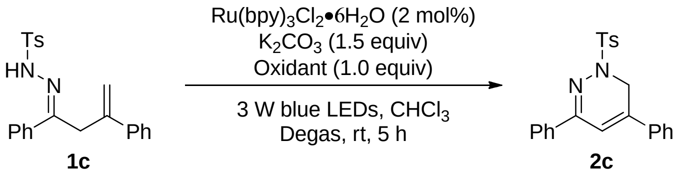

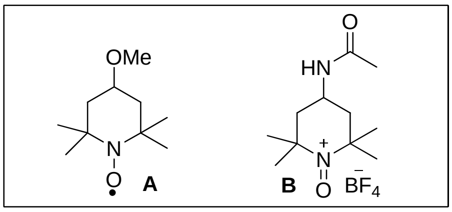

Entry	Solvent	Photocatalyst	Oxidants	Light source	Yield [%][b]
1	$CHCl_3$	$Ru(bpy)_3Cl_2·6H_2O$	**DDQ**	3 w blue LEDs	trace
2	$CHCl_3$	$Ru(bpy)_3Cl_2·6H_2O$	**MnO_2**	3 w blue LEDs	trace
3	$CHCl_3$	$Ru(bpy)_3Cl_2·6H_2O$	**$K_2S_2O_8$**	3 w blue LEDs	trace
4	$CHCl_3$	$Ru(bpy)_3Cl_2·6H_2O$	**$CBrCl_3$**	3 w blue LEDs	trace
5	$CHCl_3$	$Ru(bpy)_3Cl_2·6H_2O$	**A**	3 w blue LEDs	86
6	$CHCl_3$	$Ru(bpy)_3Cl_2·6H_2O$	**B**	3 w blue LEDs	trace

[a] Unless otherwise noted, the reaction conditions are as follows: **1c** (0.2 mmol), $Ru(bpy)_3Cl_2•6H_2O$ (2 mol%), Oxidant (0.2 mmol), K_2CO_3 (0.3 mmol), $CHCl_3$ (4.0 mL), 3 W blue LEDs (450-460 nm), at room temperature for 5 h. [b] Isolated yields.

Supplementary Table 2. Control experiments by using CH_3CN as the solvent[a]

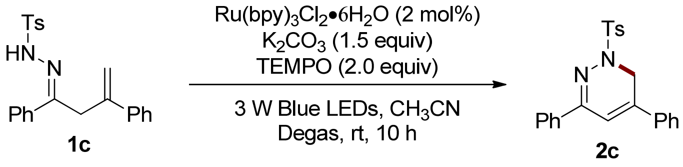

Entry	$Ru(bpy)_3Cl_2·6H_2O$	TEMPO	K_2CO_3	Light source	Yield [%][b]
1[c]	-	+	+	+	0
2[d]	+	-	+	+	0
3[e]	+	+	-	+	76
4[f]	+	+	+	-	0

[a] Unless otherwise noted, the reaction conditions are as follows: **1c** (0.2 mmol), $Ru(bpy)_3Cl_2•6H_2O$ (2 mol%), TEMPO (0.4 mmol), K_2CO_3 (0.3 mmol), CH_3CN (4.0 mL), 3W blue LEDs (450-460 nm), at room temperature for 10 h. [b] Isolated yields. [c] Without photocatalyst. [d] Without TEMPO. [e] Without base. [f] Without visible light irradiation.

The results of control experiments reveal that the 6-endo cyclization of β,γ-unsaturated hydrazones in CH_3CN is indeed a photocatalytic process.

Supplementary Table 3. Antifungal *in vitro* Activities of Diazinium Salts 17a-17e (MIC_{80}, μg/mL)

entry	Compd.	Candida Albicans Y0109	Candida Albicans SC5314	Candida Parapsilosis 22019	Cryptococcus neoformans 32609
1	**17a**	16	>64	16	8
2	**17b**	4	64	4	4
3	**17c**	4	64	4	4
4	**17d**	32	>64	4	2
5	**17e**	32	>64	4	2
6	**FCZ**	0.5	0.5	2	0.5

Supplementary Table 4. Antifungal *in vitro* Activities of Diazinium Salts 17a-17e (MIC_{80}, μg/mL)

entry	Compd.	Candida glabarata 537	Aspergillus fumigates 07544	Trichophyton rubrum	Microsporum gypseum
1	**17a**	4	>64	>64	>64
2	**17b**	1	>64	>64	>64
3	**17c**	0.5	>64	>64	>64
4	**17d**	4	>64	>64	>64
5	**17e**	2	>64	>64	>64
6	**FCZ**	0.5	>64	4	32

Experimental protocols for in vitro antifungal activity assay: The *in vitro* antifungal activities of all the target compounds were evaluated against four human pathogenic fungi- *C. alb.* - *Candida albicans*, *C. par.* - *Candida parapsilosis*, *C. neo.* - *Cryptococcus neoformans*, *C. gla.* - *Candida glabrata*, and were compared with FCZ (Fluconazole). Test fungal strains were obtained from the ATCC or were clinical isolates.

The in vitro antifungal activity was measured by means of the minimum inhibitory concentration (MIC) that achieved 80% inhibition of the tested fungi using the serial dilution method in 96-well micro test plates according to the methods defined by the National Committee for Clinical and Laboratory Standards Institute (NCCLSI) (NCCLSI-M27-A3[1] and NCCLSI-M38-A2[2]). The MIC80 was defined as the first well containing an approximate 80% reduction in growth compared with growth in the drug-free well. RPMI 1640 (Sigma) buffered with MOPS and YEPD were used as the test medium. For assays, the test compounds were dissolved in DMSO, serially diluted in growth medium, inoculated, and incubated at 35°C. The growth MIC80 was determined at 24 h for *C. albicans* and at 72 h for *C. neoformans*.

Supplementary Notes

Supplementary Note 1

Complete reference for Gaussian 09

Gaussian 09, Revision D.01, Frisch, M. J.; Trucks, G. W.; Schlegel, H. B.; Scuseria, G. E.; Robb, M. A.; Cheeseman, J. R.; Scalmani, G.; Barone, V.; Mennucci, B.; Petersson, G. A.; Nakatsuji, H.; Caricato, M.; Li, X.; Hratchian, H. P.; Izmaylov, A. F.; Bloino, J.; Zheng, G.; Sonnenberg, J. L.; Hada, M.; Ehara, M.; Toyota, K.; Fukuda, R.; Hasegawa, J.; Ishida, M.; Nakajima, T.; Honda, Y.; Kitao, O.; Nakai, H.; Vreven, T.; Montgomery, Jr., J. A.; Peralta, J. E.; Ogliaro, F.; Bearpark, M.; Heyd, J. J.; Brothers, E.; Kudin, K. N.; Staroverov, V. N.; Kobayashi, R.; Normand, J.; Raghavachari, K.; Rendell, A.; Burant, J. C.; Iyengar, S. S.; Tomasi, J.; Cossi, M.; Rega, N.; Millam, N. J.; Klene, M.; Knox, J. E.; Cross, J. B.; Bakken, V.; Adamo, C.; Jaramillo, J.; Gomperts, R.; Stratmann, R. E.; Yazyev, O.; Austin, A. J.; Cammi, R.; Pomelli, C.; Ochterski, J. W.; Martin, R. L.; Morokuma, K.; Zakrzewski, V. G.; Voth, G. A.; Salvador, P.; Dannenberg, J. J.; Dapprich, S.; Daniels, A. D.; Farkas, Ö.; Foresman, J. B.; Ortiz, J. V.; Cioslowski, J.; Fox, D. J. Gaussian, Inc., Wallingford CT, **2013**.

Supplementary Note 2

Computational methods

All the density functional theory (DFT) calculations were carried out with the GAUSSIAN 09 series of programs. DFT method B3-LYP[3,4] with a standard 6-31G(d) basis set (SDD basis set for Ru) was used for geometry optimizations. Harmonic frequency calculations were performed for all stationary points to confirm them as a local minima or transition structures and to derive the thermochemical corrections for the enthalpies and free energies. The minimum energy crossing point (MECP) in this work were located with the code developed by Harvey and co-workers at a B3-LYP/6-31G(d) level.[5-7] N12 functional,[8] which could give more accurate energetic information, is used to calculate single point energies. Solvent effects were considered by single point calculations on the gas-phase stationary points with a SMD continuum solvation model.[9-11] The larger basis set 6-311+G(d, p) (SDD for Ru) is used in the solvation single point calculations. The energies given in this work are the N12 calculated Gibbs free energies in chloroform solvent.

Geometry	$E_{(elec\text{-}B3LYP)}$[a]	$E_{(solv,\ N12)}$[b]	$G_{(corr\text{-}B3LYP)}$[c]	$H_{(corr\text{-}B3LYP)}$[d]	IF[e]
**1a-A**	-1316.011152	-1316.317491	0.250804	0.328203	-
**1a-TS1**	-1315.994271	-1316.306420	0.253780	0.326752	-405.8
**1a-B**	-1316.010032	-1316.324981	0.255267	0.328389	-
**1a-TS2**	-1315.986829	-1316.299558	0.254409	0.327192	-443.1
**1a-C**	-1316.013979	-1316.332667	0.255162	0.328911	-
**1b-A**	-1355.329862	-1355.639837	0.276941	0.357833	-
**1b-TS1**	-1355.313603	-1355.629773	0.278798	0.356020	-387.6
**1b-B**	-1355.326770	-1355.636938	0.279884	0.357377	-
**1b-TS2**	-1355.305803	-1355.624905	0.279847	0.356716	-451.2

**1b-C**	-1355.335371	-1355.659345	0.282756	0.359000	-
**1c-A**	-1547.061450	-1547.473492	0.325244	0.414194	-
**1c-TS1**	-1547.038843	-1547.458216	0.328214	0.412111	-374.7
**1c-B**	-1547.052214	-1547.465017	0.328506	0.413404	-
**1c-TS2**	-1547.045720	-1547.463360	0.328982	0.413286	-373.4
**1c-C**	-1547.081272	-1547.507939	0.331891	0.415819	-
**MECP-I**	-2030.771639	-2031.257959	-	-	-
**2c**	-1546.506720	-1546.937333	0.323015	0.405450	-
**MECP-II**	-2030.773116	-2031.258635	-	-	-
**8**	-2030.811844	-2031.286686	0.588262	0.696964	-
**MECP-III**	-2030.748311	-2031.222217	-	-	-
**9-TS**	-2294.557811	-2295.272831	0.593535	0.709031	-477.2
**10**	-2030.233935	-2030.765849	0.574046	0.681584	-
**11**	-483.698010	-483.846596	0.224834	0.272617	-
**18**	-2030.814408	-2031.286339	0.585753	0.696473	-

[a] The electronic energy calculated by B3LYP in gas phase. [b] The electronic energy calculated by N12 in chloroform solvent. [c] The thermal correction to Gibbs free energy calculated by B3LYP in gas phase. [d] The thermal correction to enthalpy calculated by B3LYP in gas phase. [e] The B3LYP calculated imaginary frequencies for the transition states.

Supplementary Note 3

Optimized Geometries for All the Compounds and Transition State

1a-A

C -3.92634800 2.77114700 -0.12577600
C -3.58972000 1.43311400 -0.29789100
C -2.24170100 1.00979800 -0.22556700
C -1.24781700 1.98779800 0.03421000
C -1.59256900 3.32216300 0.19851100
C -2.93174000 3.72302900 0.11855400
H -4.96823200 3.07399600 -0.17952900
H -4.37760800 0.70840800 -0.46589100
H -0.21304700 1.67329800 0.09768700
H -0.81615800 4.05759900 0.39016600
H -3.19783700 4.76839500 0.24844800
C -1.87478700 -0.38430300 -0.40626900
C -2.87294800 -1.45072400 -0.80473600
H -3.65320200 -1.02656500 -1.44302100
N -0.57770700 -0.68901400 -0.17355400
N -0.27310400 -1.93908900 -0.28312900
H -2.33250000 -2.20471900 -1.38870500
S 1.42149000 -2.25218000 0.05943600
O 1.88240200 -3.04751100 -1.08208600
O 1.44411200 -2.79585400 1.42216600
C 2.29975600 -0.68922000 0.05444000
C 2.42798200 0.03465000 1.24083700
C 2.86411800 -0.22849200 -1.13623100
C 3.13058700 1.23818000 1.22610600
H 1.99476900 -0.34971400 2.15797800
C 3.56321800 0.97684300 -1.13191800
H 2.76619300 -0.81529200 -2.04347400
C 3.70883800 1.72669600 0.04479100

H	3.23765500	1.80488900	2.14786200
H	4.00843700	1.33858100	-2.05560300
C	4.50037200	3.01307700	0.04578500
H	5.56919000	2.81538800	0.20197400
H	4.40515000	3.54360000	-0.90754200
H	4.17379200	3.68542400	0.84582200
C	-3.49404400	-2.12432800	0.40194900
C	-4.80274500	-2.17425100	0.65086600
H	-5.53487600	-1.71870700	-0.01372600
H	-5.19527600	-2.68818400	1.52391400
H	-2.78941900	-2.59894000	1.08284600

1a-TS1

C	-4.93991700	1.04138900	0.87588700
C	-3.83071700	0.22258600	1.08957600
C	-2.89195700	0.00584600	0.06583900
C	-3.10001100	0.62710200	-1.18205400
C	-4.20557900	1.44330100	-1.38984700
C	-5.13149800	1.65550400	-0.36187500
H	-5.65479400	1.19725300	1.67920800
H	-3.69991200	-0.24839400	2.05904900
H	-2.38355200	0.45303400	-1.97807700
H	-4.35124400	1.91370300	-2.35853000
H	-5.99651700	2.29155200	-0.52830000
C	-1.71848600	-0.84782500	0.28422600
C	-1.47877100	-1.60254200	1.59236500
H	-1.45826600	-0.88661700	2.42363100
N	-0.81606300	-0.98623600	-0.62942900
N	0.11014500	-1.91536000	-0.39379700
H	-2.32030800	-2.28161200	1.78062300
S	1.67674500	-1.53066700	-1.03349400
O	1.51675900	-1.45547900	-2.48680100
O	2.56843600	-2.50956800	-0.40884900
C	2.12007800	0.10639200	-0.45014800
C	3.03802200	0.23711400	0.59246400
C	1.55440300	1.23187400	-1.05561600
C	3.38540200	1.51297600	1.03529400
H	3.48028400	-0.64979800	1.03297300
C	1.90742200	2.49775500	-0.59388000
H	0.85992900	1.11267200	-1.87964500
C	2.82698100	2.65982100	0.45383800
H	4.10666800	1.61948500	1.84201500
H	1.46714700	3.37616900	-1.05966700
C	3.22821900	4.03968100	0.91907000
H	3.99592400	4.46827100	0.26126600
H	2.37643300	4.72849100	0.91289700
H	3.64126400	4.01758600	1.93256300
C	-0.18229900	-2.38619300	1.52572000
C	0.90309700	-2.02063200	2.31671400
H	0.95215200	-1.04018700	2.78275300
H	1.77573700	-2.65913800	2.39471800
H	-0.26533200	-3.43513700	1.26076300

1a-B

C	5.23039800	0.97315200	-0.79793900
C	4.14419700	0.10758900	-0.93310100
C	3.05721600	0.17855500	-0.04603100
C	3.08795900	1.13742800	0.98532500
C	4.17118100	1.99880800	1.11547000
C	5.24782700	1.92203900	0.22420800
H	6.06300900	0.90328500	-1.49266100
H	4.14646900	-0.62856600	-1.73159600
H	2.25422200	1.18748100	1.67773000
H	4.18076800	2.73262100	1.91698200
H	6.09351700	2.59600700	0.32990500
C	1.91589100	-0.73106400	-0.18723500
C	1.79887700	-1.83876100	-1.22305200
H	2.06079100	-1.51681000	-2.23494300
N	0.90659000	-0.69690400	0.61821700
N	0.02996700	-1.70909000	0.28259400
H	2.45697600	-2.67644000	-0.95728600
S	-1.53986300	-1.59515600	0.94595400
O	-1.36502700	-1.50544300	2.39173900
O	-2.26531000	-2.69837000	0.31066800
C	-2.25269000	-0.04594700	0.39336200
C	-3.21804300	-0.05491700	-0.61419600
C	-1.84465200	1.15079100	0.98971800
C	-3.77341200	1.15399500	-1.03220000
H	-3.53327900	-0.99725700	-1.04849300
C	-2.40456600	2.34822800	0.55184100
H	-1.10659800	1.13650700	1.78363600
C	-3.37636100	2.37069200	-0.46088800
H	-4.53057500	1.15118800	-1.81251800
H	-2.08742300	3.28249500	1.00894800
C	-4.00012500	3.67442000	-0.89963800
H	-4.79913700	3.97921800	-0.21083000
H	-3.26336600	4.48490500	-0.91820100
H	-4.44208700	3.59230800	-1.89768800

C	0.30478200	-2.23989600	-1.11598000
C	-0.54449200	-1.63096200	-2.17529200
H	-0.38959600	-0.59822800	-2.47281200
H	-1.40542300	-2.15890800	-2.56716700
H	0.16240100	-3.31975700	-1.07943500

1a-TS2

C	-4.93192500	1.12893100	0.61691200
C	-3.84735900	0.35549700	1.03438700
C	-2.80964700	0.02851900	0.14445700
C	-2.90312000	0.49071900	-1.18452300
C	-3.98117400	1.26588100	-1.59495900
C	-5.00278300	1.59189400	-0.69602000
H	-5.72273300	1.36606800	1.32348400
H	-3.82733400	-0.00062100	2.05895400
H	-2.11810500	0.22568100	-1.88469800
H	-4.02958700	1.61411700	-2.62330900
H	-5.84632900	2.19519600	-1.02004600
C	-1.65394800	-0.79727500	0.56517100
C	-1.51330400	-1.23993200	2.03512600
H	-1.89238500	-0.43997700	2.67679100
N	-0.78079000	-1.10646400	-0.33726100
N	0.18904900	-1.99567300	-0.20893300
H	-2.16158600	-2.11725400	2.19207600
S	1.63140800	-1.54676700	-1.08347100
O	1.26903000	-1.43464900	-2.49745700
O	2.63076600	-2.51165700	-0.61711800
C	2.10331700	0.08092400	-0.49844700
C	3.07223800	0.19310600	0.50052200
C	1.49086100	1.21555000	-1.03655200
C	3.42536400	1.45948400	0.96439200
H	3.55179600	-0.69928200	0.88832400
C	1.85409600	2.47215000	-0.55713500
H	0.75388800	1.11123400	-1.82506400
C	2.82431400	2.61557700	0.44644300
H	4.18528900	1.55163200	1.73660500
H	1.37969000	3.35785000	-0.97274600
C	3.23402500	3.98581700	0.93203500
H	3.65481100	3.94494400	1.94184400
H	3.99915000	4.42202200	0.27619000
H	2.38501900	4.67762800	0.94374900
C	-0.09010400	-1.56372600	2.35760000
C	0.48715800	-2.63095400	1.70360900
H	-0.11013200	-3.50447400	1.45826300
H	1.55774000	-2.80743100	1.73353500
H	0.53404400	-0.77662700	2.77344500

1a-C

C	-4.90885200	1.75282200	0.19744900
C	-4.16119900	0.59991500	0.44546400
C	-2.80255100	0.53141900	0.09420400
C	-2.21467300	1.65825500	-0.51203600
C	-2.96082000	2.80556100	-0.75672200
C	-4.31376000	2.86030700	-0.40424500
H	-5.95860400	1.78078800	0.47732300
H	-4.65091500	-0.24650800	0.91585700
H	-1.16557200	1.61469600	-0.78168500
H	-2.48649300	3.66398400	-1.22534500
H	-4.89491300	3.75798900	-0.59716100
C	-2.00026600	-0.69144600	0.35233200
C	-2.64693700	-1.86437700	1.07107000
H	-3.02916200	-1.52302500	2.04498400
N	-0.76600400	-0.67417500	-0.02231800
N	0.05810500	-1.74142200	0.22903700
H	-3.54793200	-2.17245900	0.50808600
S	1.46951500	-1.66939900	-0.78625900
O	1.09770200	-1.52928000	-2.19771700
O	2.27588500	-2.80907900	-0.33652300
C	2.22846900	-0.14673500	-0.24412100
C	2.95209700	-0.13289300	0.94967800
C	2.12581500	0.99263900	-1.04172400
C	3.56953600	1.04943400	1.34967600
H	3.03323900	-1.03447900	1.54753200
C	2.75257100	2.16593800	-0.62525500
H	1.56945600	0.94917200	-1.97143300
C	3.48164200	2.21397500	0.57143100
H	4.13281600	1.06853400	2.27941600
H	2.67547200	3.05855500	-1.24118500
C	4.18388800	3.48110400	0.99812200
H	4.23556800	3.56345000	2.08878600
H	5.21485800	3.50418400	0.62048300
H	3.67566200	4.37069000	0.61216900
C	-1.71031900	-3.00838200	1.24687800
C	-0.53991900	-3.09911800	0.33680400
H	-0.83919500	-3.44111000	-0.67261900
H	0.23123400	-3.77618200	0.70611200
H	-1.94949400	-3.82235200	1.92167400

1b-A

C	-3.50459000	3.16187800	-0.21190400
C	-3.28987300	1.79656400	-0.36508200
C	-1.98082700	1.26083600	-0.32347000
C	-0.89840500	2.15333900	-0.11461000
C	-1.12264600	3.51504300	0.03238800
C	-2.42472500	4.02828000	-0.01642000
H	-4.51772000	3.55275200	-0.24247200
H	-4.13903000	1.13592400	-0.49256600
H	0.10701100	1.75175100	-0.07442300
H	-0.28083900	4.18444600	0.18651200
H	-2.59575300	5.09491800	0.10017800
C	-1.74589700	-0.16290800	-0.49550300
C	-2.86073200	-1.13442100	-0.82097800
H	-3.49967600	-0.70477800	-1.59962300
N	-0.46325700	-0.56853900	-0.34735300
N	-0.24378000	-1.82817300	-0.51949600
H	-2.39092900	-2.03348100	-1.23552100
S	1.42565200	-2.27754600	-0.21176700
O	1.85876300	-2.95269300	-1.43862800
O	1.39176500	-2.98671500	1.07256200
C	2.39471400	-0.78249700	-0.01404500
C	2.55388900	-0.22633400	1.25627200
C	2.99618300	-0.20675300	-1.13412700
C	3.32640300	0.92450700	1.39873100
H	2.08986300	-0.69891100	2.11534900
C	3.76557000	0.94391200	-0.97250200
H	2.87181900	-0.66432700	-2.10987600
C	3.94415700	1.52559700	0.29142100
H	3.45730000	1.36042900	2.38622900
H	4.24032800	1.39455700	-1.84065700
C	4.81120000	2.75041300	0.46224300
H	5.85220200	2.46762400	0.66778100
H	4.81523300	3.36932000	-0.44104200
H	4.47231500	3.36942100	1.29965100
C	-3.71867800	-1.54523100	0.37240800
C	-5.05281300	-1.47847600	0.31225500
H	-5.57323500	-1.10846700	-0.56834600
H	-5.67522300	-1.80951600	1.13958000
C	-2.98489900	-2.07405000	1.57890600
H	-2.34715400	-1.30080900	2.02767400
H	-3.68184200	-2.42495400	2.34554100
H	-2.31827100	-2.90112000	1.30486500

1b-TS1

C	5.17600100	-1.82978200	-0.21350900
C	4.31269000	-0.73921400	-0.32444000
C	2.92923200	-0.89711500	-0.13299200
C	2.43379300	-2.18155800	0.16816500
C	3.29740000	-3.26465600	0.28028500
C	4.67369000	-3.09501500	0.09008500
H	6.24250100	-1.68764300	-0.36545800
H	4.72287800	0.23668900	-0.56526300
H	1.36612500	-2.30701100	0.31391500
H	2.89855300	-4.24753300	0.51661600
H	5.34632700	-3.94383400	0.17755000
C	2.01271200	0.24582900	-0.24195400
C	2.50019400	1.66048000	-0.55139100
H	3.31362300	1.91927700	0.13573100
N	0.74139800	0.09365300	-0.07951000
N	-0.00383100	1.16753900	-0.37253000
H	2.92342600	1.68174200	-1.56484200
S	-1.30868200	1.39767400	0.75827600
O	-1.90593500	2.67546500	0.35226300
O	-0.87333700	1.16967900	2.14232200
C	-2.42147100	0.07257400	0.29653000
C	-2.36088400	-1.14387600	0.97681500
C	-3.34196600	0.28094500	-0.73273100
C	-3.23579000	-2.16573900	0.61116300
H	-1.65254600	-1.27361000	1.78757600
C	-4.20870200	-0.75143500	-1.08219000
H	-3.38417700	1.24256900	-1.23289100
C	-4.16654200	-1.98956100	-0.42250700
H	-3.20030600	-3.11358800	1.14266300
H	-4.93439300	-0.59278500	-1.87630000
C	-5.09319800	-3.11028500	-0.83089600
H	-5.21469900	-3.84364900	-0.02742700
H	-6.08543900	-2.73200500	-1.09979600
H	-4.70185600	-3.64445400	-1.70694600
C	1.36008700	2.66803000	-0.43828600
C	1.22461000	3.35865400	0.76755600
H	0.51013700	4.16831500	0.86865400
H	1.72087900	3.01545500	1.67027500
C	0.88223700	3.32742500	-1.71649900
H	-0.07173400	3.83490300	-1.55183000
H	1.61913300	4.06925500	-2.05260800
H	0.74712100	2.59142100	-2.51367100

1b-B

C	-5.27969600	1.52970100	-0.40133200
C	-4.31494400	0.52915900	-0.52643300
C	-2.98665400	0.76235700	-0.13404800
C	-2.64686300	2.02648800	0.38533100
C	-3.61102100	3.02018400	0.50946300
C	-4.93247700	2.77719700	0.11695400
H	-6.30258000	1.33198400	-0.71018400
H	-4.59926500	-0.43665300	-0.93380900
H	-1.62049200	2.20836500	0.68600900
H	-3.33420300	3.99034000	0.91351200
H	-5.68332300	3.55651600	0.21482700
C	-1.97212500	-0.28998800	-0.26254100
C	-2.21667600	-1.67250300	-0.83203000
H	-3.09838600	-2.16267200	-0.40818100
N	-0.74029800	-0.09327300	0.06708900
N	0.00933900	-1.21753500	-0.24291700
H	-2.36074000	-1.61603300	-1.91958300
S	1.37551300	-1.38804600	0.80905500
O	2.02548700	-2.62631200	0.36305500
O	1.00489900	-1.19939400	2.21495400
C	2.36803100	0.00945500	0.30024200
C	2.30757800	1.19729500	1.02804100
C	3.21860100	-0.12427100	-0.79900100
C	3.10754900	2.26955300	0.63582400
H	1.65335000	1.26855000	1.88957500
C	4.01044100	0.95764300	-1.17445800
H	3.26681100	-1.06586500	-1.33541300
C	3.96435700	2.17033800	-0.46920700
H	3.06944200	3.19718500	1.20150600
H	4.68040200	0.85820200	-2.02505400
C	4.80792300	3.34515800	-0.90437200
H	4.96551100	4.05204700	-0.08370100
H	5.78894800	3.02127400	-1.26841500
H	4.32368100	3.89458800	-1.72265700
C	-0.90961600	-2.44136800	-0.48533700
C	-1.06900800	-3.25822500	0.75229000
H	-0.44978100	-4.13324500	0.91425400
H	-1.63110800	-2.86409300	1.59317800
C	-0.36343600	-3.26074700	-1.65651800
H	0.59019500	-3.72156700	-1.38912100
H	-1.07508100	-4.05161500	-1.92022600
H	-0.20974600	-2.62180700	-2.53188100

1b-TS2

C	4.46397400	2.00029600	-0.71316900
C	3.56502500	0.96602000	-0.97851300
C	2.66511900	0.52312500	0.00664400
C	2.70079100	1.14578800	1.27079300
C	3.59870200	2.17459800	1.53189600
C	4.48638600	2.60882400	0.54131000
H	5.14718500	2.32866400	-1.49188400
H	3.56435700	0.51465400	-1.96542200
H	2.01695600	0.80227200	2.03989800
H	3.61208700	2.63738200	2.51518000
H	5.18955000	3.41074000	0.74890300
C	1.69849000	-0.56621400	-0.26130600
C	1.85296500	-1.39546000	-1.55440000
H	1.56863600	-0.74697100	-2.39678700
N	0.75007200	-0.74243900	0.59933700
N	-0.12048900	-1.74030900	0.60504700
H	2.91464600	-1.63229700	-1.68878600
S	-1.63876900	-1.29596000	1.32779300
O	-1.37475600	-0.91420900	2.71717300
O	-2.52062700	-2.41687000	0.99073000
C	-2.22244900	0.15963100	0.45599300
C	-3.16715000	0.01403600	-0.56066500
C	-1.72194900	1.42010700	0.79724500
C	-3.61113900	1.14759400	-1.24307800
H	-3.56068800	-0.97020400	-0.79191300
C	-2.17347800	2.53808500	0.10180000
H	-1.00299300	1.51707600	1.60293700
C	-3.12247400	2.42177400	-0.92710200
H	-4.35538000	1.03979200	-2.02824700
H	-1.78814200	3.52020200	0.36564900
C	-3.60774400	3.64856900	-1.66277800
H	-4.10166800	4.35057500	-0.97961200
H	-2.77490300	4.18812200	-2.13018500
H	-4.32309700	3.38822400	-2.44874000
C	1.02484300	-2.64862300	-1.52670300
C	-0.32681700	-2.48889800	-1.31673600
H	-0.82604300	-1.57806600	-1.63816500
H	-0.97505200	-3.34030000	-1.13696000
C	1.71877600	-3.96141700	-1.31771600
H	2.29187200	-3.95760800	-0.37776000
H	1.00954000	-4.79310600	-1.27458300
H	2.43820700	-4.16293400	-2.12321900

1b-C

C	4.76874500	1.78414200	-0.74917200
C	3.87705000	0.71846300	-0.88607000
C	2.80449200	0.55669100	0.00676300
C	2.65882200	1.49177900	1.04981600
C	3.54933100	2.55125200	1.18489200
C	4.60925700	2.70559200	0.28464400
H	5.58927900	1.88998500	-1.45392800
H	4.02518300	0.01660900	-1.70062600
H	1.84440000	1.36131400	1.75394400
H	3.42189000	3.25789800	2.00086200
H	5.30495100	3.53321500	0.39356700
C	1.84797500	-0.57052300	-0.13161500
C	2.16023300	-1.69721100	-1.09958500
H	2.27924700	-1.27277300	-2.11561300
N	0.80553700	-0.54591300	0.62757700
N	-0.09615100	-1.57518700	0.60483400
H	3.15025900	-2.11419300	-0.85693000
S	-1.55565500	-1.14927900	1.42874400
O	-1.16523700	-0.60937700	2.72550400
O	-2.39792300	-2.34087900	1.29528200
C	-2.29079700	0.16761200	0.46321300
C	-3.29129400	-0.13757500	-0.46189100
C	-1.84145800	1.48116500	0.63066800
C	-3.84438500	0.88952500	-1.22663500
H	-3.64103800	-1.15960200	-0.56093900
C	-2.40410800	2.49109900	-0.14483900
H	-1.07221300	1.70056800	1.36199000
C	-3.41194100	2.21455900	-1.08235200
H	-4.62846900	0.65733200	-1.94315500
H	-2.05810500	3.51411900	-0.01828900
C	-4.03257300	3.32737700	-1.89325400
H	-4.78103000	3.87305000	-1.30358100
H	-3.28015500	4.05634200	-2.21364100
H	-4.53561900	2.94183100	-2.78561100
C	1.12615800	-2.77547800	-1.09956700
C	-0.23394000	-2.39605000	-0.61892700
H	-0.78514100	-1.81529100	-1.38812200
H	-0.83718000	-3.26968000	-0.36735700
C	1.32834600	-4.01579700	-1.90414000
H	0.67290900	-4.82736500	-1.56645900
H	1.11478300	-3.85822600	-2.97749900
H	2.36661400	-4.36664100	-1.84337600

1c-A

C	1.73859600	3.92319900	0.04236500
C	1.75450900	2.54159700	0.19780300
C	0.57103700	1.78299900	0.04172600
C	-0.62646600	2.46820500	-0.28054100
C	-0.63398000	3.84863500	-0.42934600
C	0.54618500	4.58421100	-0.26892100
H	2.65911200	4.48754000	0.16159300
H	2.69247800	2.04584300	0.42245000
H	-1.53736000	1.89448700	-0.40342800
H	-1.56263100	4.35791000	-0.67163700
H	0.53673000	5.66426100	-0.38706300
C	0.57816300	0.33733100	0.20432800
C	1.80956600	-0.40140300	0.66765800
H	2.26787300	0.16071200	1.48742900
N	-0.57731100	-0.29977800	-0.07854100
N	-0.55050100	-1.58769700	0.04500700
H	1.49305600	-1.36823800	1.07351100
S	-2.07832800	-2.31515300	-0.43860100
O	-2.21983400	-3.46995900	0.45085800
O	-2.00404300	-2.47651100	-1.89585800
C	-3.38292800	-1.14642500	-0.05915000
C	-3.81113000	-0.24381900	-1.03350700
C	-3.97520500	-1.17708400	1.20531900
C	-4.84257000	0.64318900	-0.72763700
H	-3.35614100	-0.25709900	-2.01795000
C	-5.00362000	-0.28287400	1.49282400
H	-3.64618900	-1.90563500	1.93883800
C	-5.44948100	0.64250100	0.53665800
H	-5.18875300	1.34096300	-1.48622100
H	-5.47530600	-0.30959000	2.47220300
C	-6.55075500	1.62102500	0.86982300
H	-7.34443600	1.14566100	1.45663500
H	-6.16587200	2.45871100	1.46648300
H	-7.00186800	2.04129400	-0.03446700
C	2.84856400	-0.63267600	-0.43597100
C	2.47731000	-0.84876200	-1.70646900
H	1.43609600	-0.83775300	-2.01258700
H	3.20492500	-1.08619500	-2.47624400
C	4.27613200	-0.65159500	-0.01909900
C	4.65774300	-1.13511700	1.24535900
C	5.28719400	-0.18624100	-0.87987400
C	5.99934100	-1.17379300	1.62528200
H	3.90282500	-1.50950200	1.93104500

C 6.62741500 -0.22235000 -0.49957400
H 5.01264500 0.22614900 -1.84644100
C 6.99073500 -0.71811300 0.75480400
H 6.26921100 -1.56415400 2.60299300
H 7.38909500 0.14802500 -1.18077400
H 8.03533000 -0.74269600 1.05284400

1c-TS1

C -0.61170800 5.18941600 -1.03474900
C -0.07405000 3.90304400 -1.08666100
C -0.32873500 2.97752800 -0.05956800
C -1.13253100 3.38003300 1.02600900
C -1.66760000 4.66192600 1.07247300
C -1.41054600 5.57389100 0.04224100
H -0.40308700 5.89111600 -1.83773800
H 0.54827800 3.62305600 -1.93112000
H -1.32308100 2.67179700 1.82560300
H -2.28466400 4.95587000 1.91731200
H -1.82755000 6.57636900 0.08309100
C 0.22803100 1.62087400 -0.11051300
C 1.15370100 1.15258200 -1.22940800
H 0.64109700 1.26973600 -2.19159200
N -0.05394300 0.74831700 0.80052500
N 0.64731600 -0.38495100 0.73019000
H 2.04352200 1.79138100 -1.26640900
S -0.20139000 -1.77408300 1.32698500
O -0.44360500 -1.52083000 2.74834900
O 0.59753600 -2.91420600 0.87535100
C -1.79790000 -1.82862900 0.50877100
C -2.00449700 -2.73387900 -0.53230000
C -2.82246300 -0.97934400 0.93679300
C -3.25095500 -2.77785300 -1.15712200
H -1.20494400 -3.40429700 -0.82829200
C -4.05753200 -1.03249200 0.29576200
H -2.65375200 -0.30369400 1.76762500
C -4.29135600 -1.92661500 -0.76084000
H -3.42022400 -3.49097300 -1.96023600
H -4.85806500 -0.37537400 0.62722500
C -5.63204600 -1.95958700 -1.45583200
H -5.75132600 -2.86760100 -2.05499100
H -6.45670200 -1.91719100 -0.73548100
H -5.74847000 -1.10074700 -2.12995500
C 1.56689800 -0.30927600 -1.02613500
C 0.91904000 -1.26270600 -1.83124600
H 1.21869100 -2.30345100 -1.81589800
H 0.00367900 -1.00952400 -2.35656700
C 2.99055800 -0.55275600 -0.60523900
C 3.81739400 -1.40675500 -1.34865800
C 3.54488300 0.12146200 0.49524300
C 5.15477900 -1.59587500 -0.99517200
H 3.42040100 -1.91498300 -2.22186400
C 4.87947100 -0.06514000 0.84716900
H 2.91841900 0.77217600 1.09676200
C 5.69097100 -0.92673800 0.10414800
H 5.77600600 -2.26428900 -1.58533500
H 5.28468500 0.45745200 1.70943800
H 6.73144800 -1.07343400 0.38104800

1c-B

C -1.13875200 5.31696300 -0.90179300
C -0.42484600 4.11801000 -0.91161900
C -0.73667600 3.09153300 -0.00466200
C -1.77943800 3.29923100 0.91907400
C -2.48912500 4.49459700 0.92454200
C -2.17327500 5.50948200 0.01379300
H -0.88331000 6.10038100 -1.61012800
H 0.38039600 3.98386800 -1.62800600
H -2.01387000 2.51314600 1.62915200
H -3.29013500 4.64066900 1.64431100
H -2.72850900 6.44342400 0.02262700
C 0.01219500 1.83038100 -0.01764600
C 1.20842400 1.53723300 -0.89911500
H 1.03358900 1.76206400 -1.95565400
N -0.26588200 0.86051300 0.78736900
N 0.64115300 -0.16383900 0.61012900
H 2.07636200 2.12347500 -0.57505000
S 0.10093700 -1.68236700 1.21418900
O -0.14731300 -1.47042200 2.63703200
O 1.09213200 -2.64565400 0.73289100
C -1.47476600 -2.04128300 0.43588000
C -1.53328800 -3.00773400 -0.56931300
C -2.62806500 -1.38116700 0.86859200
C -2.76335500 -3.30307600 -1.15537500
H -0.63085900 -3.53056700 -0.86633100
C -3.84586800 -1.68425200 0.26433000
H -2.56765200 -0.65409500 1.67006000
C -3.93409700 -2.64288000 -0.75686300
H -2.81516500 -4.06457400 -1.92976100

H	-4.74651500	-1.17472900	0.59819600
C	-5.25722900	-2.94401300	-1.42002000
H	-5.27502600	-3.95517900	-1.83924400
H	-6.08951800	-2.85276200	-0.71415000
H	-5.45214300	-2.24462900	-2.24407700
C	1.46026800	0.00720700	-0.67598600
C	0.87710800	-0.79817200	-1.78999900
H	1.24774100	-1.79386500	-1.99945000
H	-0.05307600	-0.47906100	-2.25013300
C	2.94040000	-0.28742200	-0.43510700
C	3.78434200	-0.49473200	-1.53448200
C	3.49790600	-0.27406900	0.84918000
C	5.15430500	-0.69292000	-1.35547200
H	3.36828900	-0.50088600	-2.53874900
C	4.86704900	-0.47561900	1.02789600
H	2.85558700	-0.12001600	1.70943200
C	5.70099500	-0.68593600	-0.07167900
H	5.79158900	-0.85531800	-2.22084800
H	5.28010200	-0.47411200	2.03306900
H	6.76640600	-0.84562200	0.07091600

1c-TS2

C	5.10080300	0.80825100	0.95630900
C	3.99235800	1.13676900	0.17420600
C	2.98576100	0.18915600	-0.08434800
C	3.12990000	-1.10135700	0.46464700
C	4.23291100	-1.42328900	1.24659300
C	5.22581300	-0.47010300	1.49863500
H	5.86885900	1.55564200	1.13629700
H	3.93182900	2.13270900	-0.25301100
H	2.35713600	-1.83742800	0.27265000
H	4.31997100	-2.42263700	1.66483600
H	6.08730400	-0.72437300	2.10994500
C	1.81363800	0.51729900	-0.92428300
C	1.47044700	1.97665600	-1.25285200
H	1.89875900	2.63265500	-0.49263600
N	1.07932800	-0.46540900	-1.34602400
N	0.12741200	-0.34929600	-2.24898900
H	1.94193100	2.24172900	-2.21105200
S	-1.11561500	-1.56836500	-2.08671700
O	-0.54888800	-2.82508700	-2.58327700
O	-2.28791400	-0.96032300	-2.72491600
C	-1.45065800	-1.77331200	-0.33731200
C	-2.34389700	-0.90988900	0.29926900
C	-0.80847600	-2.79329600	0.36763400
C	-2.58866600	-1.07290100	1.66123200
H	-2.84147600	-0.12779200	-0.26303400
C	-1.06894400	-2.94309700	1.72836700
H	-0.13210000	-3.46240000	-0.15300400
C	-1.95976000	-2.08910900	2.39518100
H	-3.27878300	-0.39807300	2.16112100
H	-0.57509100	-3.73831300	2.28163200
C	-2.25860200	-2.27889700	3.86352200
H	-2.51957400	-1.33081400	4.34527000
H	-3.10696100	-2.96141800	4.00687700
H	-1.40293300	-2.70802100	4.39521100
C	-0.02356400	2.15397200	-1.37206300
C	-0.56646800	1.62720000	-2.52934500
H	0.01215300	1.61176000	-3.44569900
H	-1.63312500	1.47835100	-2.65598100
C	-0.82948900	2.53166900	-0.21282700
C	-0.30759900	2.50334400	1.09991000
C	-2.16950000	2.95679900	-0.37335900
C	-1.09128900	2.86266100	2.19383300
H	0.70987500	2.16752700	1.27271000
C	-2.94990700	3.31201800	0.72150500
H	-2.58859400	3.03257200	-1.37221200
C	-2.41628900	3.26675200	2.01405600
H	-0.66523100	2.82179700	3.19276000
H	-3.97436700	3.63977200	0.56614800
H	-3.02364100	3.55305200	2.86822500

1c-C

C	0.12971000	5.17863100	0.82373900
C	0.60886300	3.87182500	0.71199400
C	-0.04570900	2.93094300	-0.10028900
C	-1.19128000	3.34436500	-0.80759800
C	-1.66532400	4.64669200	-0.69608200
C	-1.00887600	5.57216500	0.12256300
H	0.65183300	5.88770700	1.46064100
H	1.49829200	3.59460400	1.26849400
H	-1.68977200	2.62879400	-1.45200100
H	-2.54801700	4.94561000	-1.25539400
H	-1.38014500	6.58990700	0.20687200
C	0.44974300	1.53614300	-0.22753300
C	1.80974100	1.18080100	0.34561400
C	2.18498400	-0.26489300	0.17817000
H	2.55189900	1.83264900	-0.13619800

C	1.02575000	-1.18092500	-0.06742000
H	0.45269900	-1.33002100	0.86829600
C	3.52193800	-0.72628800	0.36718100
C	4.56398600	0.17394800	0.74136000
C	3.89135300	-2.09473500	0.20800600
C	5.86739800	-0.26200800	0.93229000
H	4.33901300	1.22472000	0.88943600
C	5.19829000	-2.51909600	0.39903500
H	3.14596800	-2.82889000	-0.07724700
C	6.20050600	-1.61103200	0.76244900
H	5.44174500	-3.56946100	0.26238800
H	7.22194900	-1.94902200	0.91091100
H	6.63328200	0.45451600	1.21771700
N	-0.28932200	0.71296900	-0.88927300
N	0.13833200	-0.57342100	-1.08281200
H	1.83315100	1.46186300	1.41274300
S	-1.14200200	-1.57173000	-1.68978800
O	-0.53588200	-2.90224800	-1.79229100
O	-1.70176200	-0.87663500	-2.84242800
C	-2.37809500	-1.61684800	-0.39546600
C	-3.31322900	-0.58188600	-0.29566400
C	-2.38353600	-2.68076500	0.50880300
C	-4.25534100	-0.62001900	0.72829400
H	-3.30554400	0.22452500	-1.01973500
C	-3.33735700	-2.70111800	1.52649200
H	-1.66898500	-3.48906400	0.39615700
C	-4.28097500	-1.67347500	1.65641100
H	-4.98905900	0.17869800	0.80602200
H	-3.35307700	-3.53375400	2.22534400
C	-5.29769800	-1.68741000	2.77306600
H	-5.37875200	-2.67856900	3.22979800
H	-5.02151900	-0.97958800	3.56567300
H	-6.29077000	-1.39631900	2.41305300
H	1.31687300	-2.15709100	-0.44650800

MECP-I

C	-0.46494700	-3.44553100	3.63060300
C	-0.57987600	-2.34930600	2.77488900
C	0.52380000	-1.91804700	2.01873300
C	1.74198200	-2.60759600	2.14386700
C	1.85289000	-3.69738500	3.00313700
C	0.75041500	-4.12034600	3.75177500
H	-1.33063500	-3.77361200	4.19961100
H	-1.53936700	-1.85325800	2.66625400
H	2.59555900	-2.26812400	1.56659100
H	2.80383700	-4.21531400	3.09591700
H	0.83991700	-4.96839000	4.42520800
C	0.40978100	-0.76314300	1.09690000
C	-0.60881200	0.30833000	1.35152100
C	-0.22847200	1.61539800	0.88981900
H	-1.66445100	-0.10391900	0.81944000
C	0.88414300	1.68145200	-0.12367400
H	1.83299300	1.97931700	0.36623900
C	-0.82535400	2.82621700	1.38593100
C	-1.97121800	2.79738800	2.22780600
C	-0.29100400	4.10271100	1.06451000
C	-2.53370800	3.96902400	2.71746000
H	-2.43207000	1.84623600	2.47235400
C	-0.85570400	5.26868600	1.56742500
H	0.58430700	4.17777300	0.42730700
C	-1.98109700	5.21402100	2.39643300
H	-0.41550300	6.22890300	1.31139400
H	-2.42332100	6.12789600	2.78329700
H	-3.41464900	3.91349200	3.35212900
N	1.19108500	-0.73765900	0.06838000
N	1.02788000	0.36396300	-0.76823600
H	-0.97173100	0.29647600	2.38334300
S	2.09176700	0.27432600	-2.12071800
O	1.89991100	1.54995700	-2.81831800
O	1.82817800	-1.00883100	-2.76371000
C	3.75431000	0.23950500	-1.45789800
C	4.30413000	-0.97775300	-1.04187300
C	4.47716600	1.42876400	-1.35167800
C	5.58941700	-0.99003100	-0.50802500
H	3.73101100	-1.89131500	-1.15010100
C	5.76495700	1.39441700	-0.81519200
H	4.04442100	2.35817700	-1.70616200
C	6.33894800	0.19155000	-0.38450400
H	6.02370100	-1.93393600	-0.18684600
H	6.33455000	2.31709400	-0.73725600
C	7.73990000	0.15397200	0.17891700
H	8.12233400	1.16082600	0.37233900
H	7.77688300	-0.40919200	1.11870600
H	8.43236200	-0.33629800	-0.51748200
H	0.68140600	2.40171900	-0.91794900
C	-5.14237200	-0.10464400	-2.15140300
H	-6.06227400	-0.45688400	-1.66458100
H	-5.41435500	0.78574800	-2.73153100

C -4.61652600 -1.21471800 -3.06151100
H -3.74839700 -0.86924500 -3.63627000
H -5.38359900 -1.48867700 -3.79619100
C -4.25255100 -2.43067300 -2.20909200
H -3.88164100 -3.25214100 -2.83405600
H -5.16154000 -2.79633900 -1.71149100
C -3.18548900 -2.13122500 -1.13168000
C -4.14123600 0.32752500 -1.05407400
N -3.57103100 -0.88467100 -0.40402900
C -4.90379500 1.12715800 0.01886100
H -4.22564400 1.55358200 0.75968400
H -5.44855400 1.94763700 -0.46210600
H -5.62582300 0.48651900 0.53588200
C -3.02519300 1.22335200 -1.64648600
H -2.49887400 0.74692400 -2.47748000
H -3.46361600 2.15798000 -2.01573700
H -2.29167300 1.47455900 -0.87637200
C -3.17643600 -3.28534300 -0.11314000
H -3.02814300 -4.23211000 -0.64473500
H -2.37365900 -3.17291500 0.61754700
H -4.12951100 -3.33470600 0.42417700
C -1.78178500 -2.01018400 -1.76738900
H -1.03733100 -1.70760100 -1.02907100
H -1.47630100 -2.98603700 -2.16141300
H -1.74816900 -1.29537400 -2.59300100
O -2.97982000 -0.68330100 0.75768800

2c

C 0.76575400 5.09453700 0.98294700
C 1.05024200 3.74068700 0.79908900
C 0.32911400 2.97681500 -0.13215100
C -0.68686600 3.60276600 -0.87587900
C -0.96776200 4.95338900 -0.69168600
C -0.24239500 5.70626500 0.23762000
H 1.33122600 5.66874700 1.71204000
H 1.82501600 3.27306400 1.40012800
H -1.23856400 3.01525600 -1.60217300
H -1.74994200 5.42458400 -1.28109000
H -0.46128700 6.76156400 0.37659600
C 0.62322400 1.53632100 -0.32647400
C 1.94274100 0.98433200 -0.01576500
C 2.12657700 -0.35235500 0.04315700
C 0.88549700 -1.20737400 -0.13283900
H 0.38489900 -1.35518500 0.84282600
C 3.42544600 -0.99069300 0.33277300
C 3.77521400 -2.23353800 -0.22715500
C 4.35746400 -0.36036300 1.18028800
C 5.01710300 -2.81129600 0.03329900
H 3.08955300 -2.74059000 -0.89907600
C 5.59532000 -0.94099400 1.44331700
H 4.09222900 0.57907000 1.65619800
C 5.93236300 -2.16939300 0.86907600
H 6.29425200 -0.43896600 2.10691300
H 6.89683900 -2.62437900 1.07679500
H 5.26972800 -3.76504400 -0.42181500
N -0.31199800 0.80247000 -0.85253300
N 0.00479300 -0.51566200 -1.08663400
H 2.77770100 1.66865800 0.08946700
S -1.34264200 -1.39765400 -1.70722300
O -0.82468200 -2.75664600 -1.89053700
O -1.88360900 -0.61178400 -2.80912600
C -2.54476700 -1.42837100 -0.38188800
C -3.36505500 -0.31532100 -0.16819800
C -2.63738000 -2.55810300 0.43224600
C -4.28152100 -0.34513700 0.87828900
H -3.28681500 0.54733200 -0.81959100
C -3.56602800 -2.56873600 1.47386000
H -2.00871900 -3.41950500 0.23409600
C -4.39577000 -1.46673500 1.71631600
H -4.92553600 0.51492300 1.04562900
H -3.64985400 -3.45089000 2.10363700
C -5.38849900 -1.47353600 2.85445500
H -6.38183100 -1.15375600 2.51899100
H -5.48502500 -2.46957200 3.29712100
H -5.08026800 -0.78377100 3.65073900
H 1.10532700 -2.18830000 -0.54858300

MECP-II

C -1.50862100 4.97228300 1.92590900
C -0.91796100 3.73705300 1.65137500
C -1.57723900 2.78305700 0.85734000
C -2.85016600 3.10624700 0.35070100
C -3.43792000 4.33597900 0.62791000
C -2.77067400 5.27844800 1.41756100
H -0.97795000 5.69566800 2.53968700
H 0.06830500 3.52733800 2.05406100
H -3.36957200 2.36907100 -0.25144200
H -4.42612500 4.55861600 0.23367800

H	-3.23397500	6.23700400	1.63688600
C	-0.96497300	1.46319100	0.56316500
C	0.21735900	1.00054000	1.38798300
C	0.59436600	-0.44352900	1.18956600
H	1.07046000	1.65931800	1.16543800
C	-0.30691900	-1.26385800	0.31703600
H	-1.08506800	-1.76160500	0.92714300
C	1.38655900	-1.11035400	2.18828000
C	2.10293900	-0.37932800	3.17528100
C	1.47689500	-2.52879100	2.25677100
C	2.85470800	-1.02051700	4.15383400
H	2.06874000	0.70537300	3.16801000
C	2.22793900	-3.16140000	3.24099800
H	0.94330600	-3.13868900	1.53495900
C	2.92478000	-2.41680800	4.19921300
H	2.26412800	-4.24785100	3.26698300
H	3.50756500	-2.91506500	4.96857400
H	3.38529700	-0.42509900	4.89269300
N	-1.47674500	0.78376100	-0.40648400
N	-0.94874200	-0.43499100	-0.71581600
H	-0.01170700	1.18973900	2.44822500
S	-1.87399200	-1.24443100	-1.91883400
O	-1.24525000	-2.56227500	-2.04259700
O	-1.99466800	-0.33064200	-3.04969400
C	-3.49807300	-1.46097900	-1.19577700
C	-4.45615000	-0.45356400	-1.33780700
C	-3.77427000	-2.61744300	-0.46350400
C	-5.69633000	-0.60779300	-0.72316400
H	-4.22830100	0.42249600	-1.93470900
C	-5.02170900	-2.75259400	0.14529800
H	-3.03028000	-3.40396600	-0.39418300
C	-5.99516300	-1.75009400	0.03591000
H	-6.44744600	0.17057600	-0.83616800
H	-5.24304800	-3.65445700	0.71085300
C	-7.32861800	-1.88479500	0.73233200
H	-7.55974100	-2.93026500	0.95981800
H	-7.33209000	-1.33439200	1.68259500
H	-8.14328900	-1.48006800	0.12206300
H	0.25980300	-2.03163000	-0.20996500
C	4.27929800	1.43444000	-2.71591600
H	3.88926200	0.91911200	-3.60394000
H	4.43774800	2.48193000	-3.00141200
C	5.59515400	0.77590000	-2.30231400
H	6.06044100	1.32014000	-1.47071000
H	6.30936000	0.81976400	-3.13376500
C	5.33554700	-0.68039800	-1.91770000
H	6.26571900	-1.17940400	-1.61929900
H	4.95799100	-1.21575600	-2.79970400
C	4.31096200	-0.85320800	-0.77009900
C	3.19156500	1.39764000	-1.61469300
N	3.11663800	0.01278400	-1.04249700
C	1.83449500	1.73588900	-2.25108500
H	1.03985000	1.83172300	-1.51048800
H	1.92713100	2.68846300	-2.78530700
H	1.53020200	0.96413100	-2.96309500
C	3.51075600	2.43828100	-0.51526500
H	4.50668900	2.30942800	-0.08378000
H	3.45864000	3.44643700	-0.94259400
H	2.78039300	2.37797100	0.29621700
C	3.85160300	-2.31867800	-0.74505800
H	4.73918600	-2.95971400	-0.69319200
H	3.22306200	-2.53278400	0.12047200
H	3.29096300	-2.56857100	-1.65075600
C	4.94346600	-0.50346900	0.59716200
H	4.19242800	-0.55603900	1.38935500
H	5.73474400	-1.22666500	0.82752900
H	5.39240900	0.49340800	0.61155900
O	1.95603700	-0.45130100	-0.73081300

8

C	-2.12745100	5.32449700	0.30332800
C	-1.41491200	4.14314200	0.51907500
C	-1.62335300	3.01772300	-0.29554600
C	-2.56245500	3.11526900	-1.34048900
C	-3.26924800	4.29347600	-1.55407400
C	-3.05743300	5.40568500	-0.73189500
H	-1.95098500	6.18149700	0.94792800
H	-0.69714200	4.10694700	1.33212400
H	-2.71441200	2.25568600	-1.98386700
H	-3.98480500	4.34915300	-2.37036700
H	-3.60928200	6.32618800	-0.90215700
C	-0.87517900	1.75322100	-0.07429300
C	0.26647800	1.73968800	0.92271400
C	1.07075800	0.40979100	1.01903500
H	0.97576500	2.54082100	0.68785900
C	0.14330200	-0.73962600	0.61470600
H	-0.60142500	-0.87073000	1.41131600
C	1.56516400	0.29141500	2.47765100

C	0.96278900	-0.51278500	3.45441500
C	2.64744100	1.10011000	2.86706100
C	1.43229500	-0.51770500	4.77177000
H	0.12648400	-1.15876400	3.21018300
C	3.11207600	1.10006500	4.18081000
H	3.13530300	1.72148900	2.12418000
C	2.50611000	0.28859600	5.14283500
H	3.95212700	1.73420700	4.45220000
H	2.86855300	0.28469700	6.16717200
H	0.95168500	-1.15946500	5.50558900
N	-1.25189700	0.72750700	-0.76024800
N	-0.56037100	-0.44257200	-0.63414500
H	-0.14385900	1.97942100	1.91133000
S	-1.36035200	-1.74979500	-1.42187200
O	-0.50206300	-2.91141300	-1.17178400
O	-1.67804700	-1.29342600	-2.77034900
C	-2.89264800	-1.98207600	-0.52356200
C	-3.99541700	-1.17329900	-0.81505600
C	-2.95909900	-2.95684200	0.47398100
C	-5.17033100	-1.34707200	-0.08887100
H	-3.93034900	-0.43528800	-1.60617900
C	-4.14667100	-3.11664800	1.18805100
H	-2.10186800	-3.59387500	0.66384700
C	-5.26491100	-2.31437000	0.92449500
H	-6.03282400	-0.72518800	-0.31614600
H	-4.20709900	-3.88349700	1.95639400
C	-6.54016700	-2.47344400	1.71786800
H	-6.57937800	-3.44243700	2.22522500
H	-6.62441300	-1.69457100	2.48731200
H	-7.42382100	-2.38907100	1.07582800
O	2.26485100	0.56993300	0.19880700
C	3.38079700	-1.84163700	-2.97162800
H	3.87593700	-2.40443900	-3.77246300
H	2.30611400	-2.03242400	-3.06696900
C	3.87902900	-2.32598400	-1.60461600
H	4.95144200	-2.55315800	-1.66087900
H	3.37513600	-3.26480800	-1.35094600
C	3.65491300	-0.34845700	-3.10812400
H	3.41258700	0.00922900	-4.11559500
H	4.72786700	-0.16206100	-2.96868900
C	2.82181400	0.48024400	-2.09272100
C	3.67539800	-1.29722500	-0.44030700
C	5.00196100	-0.58270800	-0.08366400
H	5.50346000	-0.15428300	-0.95432400
H	4.82690200	0.21300800	0.64647100
H	5.69574100	-1.30344000	0.36516900
C	3.26193300	-2.11275700	0.79706900
H	3.13801900	-1.49499700	1.68797700
H	2.34502600	-2.68028000	0.61352800
H	4.05193100	-2.84289900	1.00792900
N	2.55738500	-0.37662600	-0.86279900
C	1.46851800	0.81604800	-2.74038500
H	0.92142000	-0.08129500	-3.03772800
H	0.83365800	1.38680400	-2.06220400
H	1.63659700	1.43234500	-3.63195200
C	3.54734200	1.81527100	-1.80754000
H	4.49374500	1.68235000	-1.28059000
H	3.76362000	2.30358800	-2.76550400
H	2.92300300	2.49245100	-1.22042200
H	0.68837700	-1.66473400	0.47176600

MECP-III

C	2.18087300	0.97648200	5.17760100
C	1.56121500	1.01983500	3.92714200
C	2.32224200	0.94422600	2.74854700
C	3.72236900	0.83028800	2.85473800
C	4.33555600	0.79013900	4.10140800
C	3.56826200	0.86136700	5.27021300
H	1.57692400	1.03361400	6.07954400
H	0.48009300	1.11213300	3.86889200
H	4.31100400	0.77545300	1.94486200
H	5.41752000	0.70541200	4.16526700
H	4.05134100	0.82863800	6.24316300
C	1.67527600	0.96599700	1.43196200
C	0.18630000	1.10018200	1.18719600
H	-0.36008300	0.23817600	1.58511500
N	2.35312500	0.80561100	0.34498200
N	1.50564800	0.85019800	-0.73036900
H	-0.24574300	1.99382200	1.64541400
S	2.18401700	0.45044500	-2.22978300
O	3.28338000	1.37511200	-2.51150200
O	1.04637300	0.32551700	-3.14345200
C	2.91095400	-1.16234600	-1.94145800
C	2.11436600	-2.30565000	-2.01698900
C	4.26508000	-1.24775500	-1.61403000
C	2.68623300	-3.54767100	-1.74303300
H	1.06824200	-2.22107600	-2.29420800
C	4.81867200	-2.49688000	-1.34585300

H	4.86556700	-0.34598100	-1.57758700
C	4.04022800	-3.66255800	-1.39505200
H	2.07049700	-4.44218300	-1.79749100
H	5.87267600	-2.56764300	-1.08795200
C	4.65038700	-5.00230200	-1.05629900
H	4.01531600	-5.82964300	-1.38635600
H	5.63477700	-5.12443200	-1.52187600
H	4.79308900	-5.10569900	0.02774400
C	0.05293900	1.16417300	-0.37849900
C	-0.87066200	0.11074400	-0.90260400
H	-1.34861900	0.26781400	-1.86022500
H	-0.66877000	-0.91283500	-0.60596200
C	-0.32389200	2.57421200	-0.85476800
C	-1.66250300	2.98122500	-0.74145000
C	0.61697000	3.48131000	-1.35605800
C	-2.05290600	4.25781400	-1.14673500
H	-2.39366400	2.29317200	-0.32748700
C	0.22074900	4.75855700	-1.76382200
H	1.65915300	3.19472500	-1.44794200
C	-1.11382500	5.15144500	-1.66667700
H	-3.09570000	4.55263500	-1.05893900
H	0.96406800	5.44253500	-2.16588600
H	-1.42004000	6.14189700	-1.99448100
C	-6.12140400	-1.27864300	-0.17458100
H	-6.64307000	-0.59602100	0.51046700
H	-6.76095500	-1.38879000	-1.05871700
C	-5.92189400	-2.62097700	0.52959100
H	-5.47070400	-3.35388400	-0.15108100
H	-6.89240700	-3.03568500	0.82880000
C	-5.04500900	-2.41290100	1.76443700
H	-4.88538100	-3.35641600	2.30026400
H	-5.57222400	-1.74322300	2.45717200
C	-3.65968300	-1.80204100	1.45302400
C	-4.80021800	-0.61380000	-0.62048200
N	-3.82207400	-0.64587900	0.51426900
C	-5.07310900	0.86064700	-0.96320300
H	-4.19942700	1.33840300	-1.40978300
H	-5.90210800	0.91609300	-1.67757100
H	-5.35230500	1.42060200	-0.06491200
C	-4.21731500	-1.32077800	-1.86537100
H	-4.14491800	-2.40484100	-1.74039800
H	-4.86158700	-1.12783800	-2.73056200
H	-3.22176800	-0.93257100	-2.09167700
C	-3.05725300	-1.26186800	2.76130800
H	-3.05725000	-2.05829900	3.51373700
H	-2.03158300	-0.91689000	2.62138700
H	-3.65018600	-0.42377200	3.14088600
C	-2.70740000	-2.86221600	0.85658700
H	-1.76493500	-2.39676200	0.55775700
H	-2.48564800	-3.62584300	1.61091300
H	-3.13394000	-3.36587000	-0.01522200
O	-2.85825300	0.21973100	0.48477200

9-ts

C	0.36028600	4.82533900	0.98317100
C	0.39057200	3.42970100	0.89344000
C	-0.37926600	2.77776800	-0.08936500
C	-1.15267900	3.56026300	-0.97199800
C	-1.17889300	4.94816400	-0.86747600
C	-0.42253800	5.59195900	0.11911700
H	0.96294000	5.30863300	1.74976000
H	1.03759500	2.86613200	1.57524000
H	-1.71916400	3.05508900	-1.74751500
H	-1.78452600	5.53054100	-1.56181400
H	-0.44073400	6.67847200	0.20455500
C	-0.37633700	1.28847500	-0.21201700
C	0.48945600	0.50998500	0.68462100
C	0.60841300	-0.96479800	0.34123500
H	1.47556600	0.96896600	0.79710200
C	-0.71316700	-1.45024700	-0.30532000
H	-1.46515800	-1.50432900	0.49564200
C	0.80667000	-1.97475700	1.50071500
C	0.55119800	-1.67868800	2.84663200
C	1.16224200	-3.29500200	1.16156200
C	0.66518200	-2.68332800	3.81615100
H	0.30762700	-0.65440400	3.16036700
C	1.26865900	-4.29082600	2.13156400
H	1.37963600	-3.53346700	0.12488400
C	1.01698200	-3.98801900	3.47274200
H	1.55106900	-5.30163400	1.83862900
H	1.10266500	-4.75986700	4.23725100
H	0.48471900	-2.42136100	4.85684500
N	-1.21084400	0.83176600	-1.11468300
N	-1.16119700	-0.54686900	-1.37688800
H	0.21444700	0.77253000	1.84716000
S	-2.55724500	-1.03637200	-2.20993900
O	-2.43053000	-2.49004200	-2.41550300
O	-2.74003300	-0.15012000	-3.36271600

C	-3.93256300	-0.77051300	-1.09194100
C	-4.31937500	0.53726200	-0.76533100
C	-4.54696000	-1.86592900	-0.47790200
C	-5.33227400	0.72932200	0.17276600
H	-3.82268900	1.37770700	-1.23361000
C	-5.56740200	-1.65314200	0.44632900
H	-4.22318200	-2.86811700	-0.73830200
C	-5.97096800	-0.35327500	0.79258800
H	-5.62578500	1.74369200	0.43533100
H	-6.05231700	-2.50766300	0.91534200
C	-7.03825300	-0.12959300	1.83880800
H	-6.61138200	-0.13873400	2.85146800
H	-7.53379500	0.83904700	1.70625900
H	-7.80699800	-0.91179500	1.80502100
O	1.69979600	-1.28780000	-0.62720500
C	5.23773300	-0.37780100	-2.51861700
H	5.55930600	-1.17297900	-3.20566800
H	6.05525800	0.35536800	-2.49980500
C	3.96474200	0.30492100	-3.05023200
H	3.94578700	0.27346800	-4.14778200
H	3.96735500	1.36557700	-2.78123400
C	5.01504300	-0.96707500	-1.11235300
H	5.95084200	-0.93847700	-0.53817300
H	4.73959800	-2.02647600	-1.18675900
C	3.91179200	-0.24467300	-0.30091200
C	2.64511400	-0.32128500	-2.54712500
C	2.52658600	-1.76775600	-3.09549500
H	3.28344000	-2.44121900	-2.68199900
H	1.54537000	-2.18382800	-2.86430700
H	2.64601600	-1.75079900	-4.18752400
C	1.48776900	0.50832600	-3.13007500
H	0.52173500	0.09679600	-2.83942900
H	1.53936600	1.53950500	-2.76765000
H	1.54217300	0.51704700	-4.22855800
N	2.58274000	-0.23092400	-1.04462900
C	4.29699700	1.22865300	-0.00410300
H	4.22686400	1.87716000	-0.88190100
H	3.65047100	1.62830400	0.78632200
H	5.33408400	1.26736300	0.36059900
C	3.80719900	-0.93328400	1.06674100
H	3.50976100	-1.97976200	0.98353000
H	4.80006000	-0.89225300	1.53450600
H	3.12662100	-0.39482300	1.73262300
H	-0.58481300	-2.44225300	-0.73676100

O	0.27129500	1.21329300	3.28054100
C	1.52449100	1.58046600	3.61688100
O	2.36074400	1.81511600	2.64803000
O	1.81529100	1.68746700	4.83609900

10

C	-2.24377100	5.24605100	-0.61040000
C	-1.53468200	4.11787100	-0.19433900
C	-1.54519200	2.93471700	-0.95063700
C	-2.30454500	2.91558100	-2.13373700
C	-3.01705300	4.04007100	-2.54808900
C	-2.98927200	5.21508700	-1.79085000
H	-2.22062000	6.14959700	-0.00380900
H	-0.98040700	4.13938700	0.73917200
H	-2.31343000	1.99956400	-2.71514300
H	-3.59303400	4.00211200	-3.47102200
H	-3.54356300	6.09352600	-2.11545700
C	-0.78970800	1.71740000	-0.52323800
C	0.37117600	1.84196100	0.24273500
C	0.99220100	0.66814200	0.89770700
H	0.74566800	2.82749100	0.49138400
C	0.03291000	-0.52127500	0.74909800
H	-0.79443200	-0.39562800	1.46491800
C	1.31319700	0.99502200	2.36205600
C	0.76866600	0.31791800	3.46424000
C	2.22106600	2.03969900	2.63103200
C	1.08674100	0.68985300	4.77544600
H	0.08799100	-0.51313500	3.31598500
C	2.54481400	2.40596900	3.93362600
H	2.68251800	2.55787700	1.79699300
C	1.97306500	1.73576400	5.02142200
H	3.25062400	3.21681300	4.10362900
H	2.22310100	2.02156100	6.04078700
H	0.63937600	0.14665400	5.60567400
N	-1.34014600	0.57860100	-0.95750200
N	-0.51312900	-0.53117300	-0.61712500
S	-1.22733400	-1.96984200	-1.11473800
O	-0.38596500	-3.07121300	-0.61097400
O	-1.51453100	-1.86421900	-2.54698200
C	-2.80721600	-2.09330300	-0.25557300
C	-3.84326900	-1.20176000	-0.56061800
C	-2.96844900	-3.05182900	0.74540500
C	-5.04256900	-1.29044100	0.14352200
H	-3.69211700	-0.45155900	-1.32604300

C	-4.18045600	-3.13184900	1.43324300
H	-2.15136900	-3.73105000	0.96445600
C	-5.23214600	-2.25215000	1.14729900
H	-5.84845800	-0.59763100	-0.09131300
H	-4.31004400	-3.88952500	2.20387600
C	-6.53201400	-2.31700100	1.91667800
H	-6.65493500	-3.28459100	2.41581300
H	-6.57597700	-1.54022900	2.69250400
H	-7.39649200	-2.16312800	1.25991100
O	2.41957600	0.34507500	0.42908300
C	3.50467300	-2.27231000	-2.59303600
H	4.03078300	-2.91584200	-3.31163600
H	2.43464700	-2.36289900	-2.80840000
C	3.78385900	-2.74359800	-1.15885300
H	4.81867200	-3.10593600	-1.08140800
H	3.13543900	-3.59694200	-0.93167000
C	3.93272800	-0.81684100	-2.74734900
H	3.84464600	-0.48801300	-3.79060300
H	4.99576300	-0.72102900	-2.48534500
C	3.06396300	0.12539700	-1.87139600
C	3.58099300	-1.63656600	-0.07392400
C	4.93919500	-1.04856700	0.39337300
H	5.56030800	-0.70080600	-0.43690700
H	4.76302000	-0.20924700	1.07202700
H	5.51422600	-1.81364900	0.93146600
C	2.94130600	-2.31586700	1.15104700
H	2.77639700	-1.60602500	1.96547700
H	1.99224400	-2.78361800	0.87515400
H	3.61167000	-3.10661100	1.51293300
N	2.63239200	-0.61115100	-0.61901000
C	1.79552900	0.49185400	-2.66479100
H	1.21621400	-0.39442200	-2.93628500
H	1.14830400	1.13888800	-2.07248300
H	2.07883800	1.02581300	-3.58306600
C	3.83991600	1.43133100	-1.59414700
H	4.72740600	1.27103600	-0.97563200
H	4.16749900	1.85999900	-2.55089800
H	3.19871900	2.15843800	-1.09208400
H	0.52775000	-1.46884400	0.92755900

11

C	-1.24861000	1.44749800	-0.40853900
H	-1.25874300	1.54291900	-1.50500800
H	-2.16485800	1.93214700	-0.03695300
C	0.00000300	2.16041700	0.11965600
H	0.00000400	2.16230600	1.21825000
H	0.00000500	3.21791500	-0.18956100
C	1.24861400	1.44749500	-0.40854100
H	2.16486300	1.93214100	-0.03695600
H	1.25874600	1.54291500	-1.50501000
C	1.27461500	-0.05422500	-0.05259100
C	-1.27461500	-0.05422100	-0.05259000
N	-0.00000100	-0.70787900	-0.47580800
C	-2.35991200	-0.75632700	-0.89514500
H	-2.26453900	-1.82880500	-0.70673200
H	-3.37160300	-0.40369700	-0.64393200
H	-2.17528100	-0.58141400	-1.96286700
C	-1.61174900	-0.30016800	1.43961600
H	-1.10543600	0.39765800	2.11665100
H	-2.69134800	-0.23339500	1.63920600
H	-1.25566400	-1.32134800	1.62981600
C	2.35991200	-0.75633300	-0.89514300
H	3.37160400	-0.40371200	-0.64392500
H	2.26452900	-1.82881100	-0.70673800
H	2.17528700	-0.58141200	-1.96286500
C	1.61174900	-0.30016800	1.43961500
H	1.25566000	-1.32134600	1.62982100
H	2.69134800	-0.23339800	1.63920300
H	1.10543800	0.39766100	2.11664800
O	-0.00000500	-2.05462300	-0.05955400

18

C	-3.50539100	0.54100200	-4.55421100
C	-2.61025900	0.88610700	-3.54064100
C	-2.93885300	0.67084700	-2.19186300
C	-4.19123700	0.10575700	-1.88300900
C	-5.07958000	-0.23752900	-2.89567300
C	-4.74116600	-0.02239600	-4.23677900
H	-3.23457500	0.71468200	-5.59209100
H	-1.65226900	1.32533900	-3.80228800
H	-4.45198700	-0.05228600	-0.84186100
H	-6.04301800	-0.67140500	-2.64141300
H	-5.43868200	-0.29028100	-5.02568000
C	-1.99347000	1.02945800	-1.12855700
C	-0.66686900	1.72733700	-1.33621500
H	-0.03595400	1.28388100	-2.10760000
N	-2.25914500	0.82854200	0.11865300
N	-1.21475300	1.31037400	0.88947000

H	-0.83455500	2.77762200	-1.60398000
S	-1.25496700	0.76850900	2.51564600
O	-2.46808500	1.33462500	3.09855600
O	0.07498200	1.07619100	3.04848000
C	-1.45910800	-1.01403000	2.48217400
C	-0.36715300	-1.82781800	2.78575100
C	-2.71467800	-1.56649500	2.20978600
C	-0.53435700	-3.21297900	2.79662100
H	0.58880200	-1.37914100	3.03284900
C	-2.85896200	-2.95071400	2.21897600
H	-3.55828100	-0.91784100	2.00569300
C	-1.77482100	-3.79561100	2.50644800
H	0.31187000	-3.84910900	3.04379600
H	-3.83350800	-3.38493000	2.00896600
C	-1.94585700	-5.29601100	2.49297800
H	-1.12550300	-5.79824800	3.01500800
H	-2.88621200	-5.59563700	2.96878400
H	-1.96752800	-5.68022000	1.46464400
C	0.00930100	1.62359600	0.06502100
C	0.97462300	0.40195900	0.13371200
H	1.53870600	0.46116200	1.06528300
H	0.37193100	-0.50988400	0.13156700
C	0.64725700	2.95518800	0.46709500
C	1.88599300	3.32462900	-0.07887000
C	-0.01719100	3.86495900	1.29987500
C	2.45304000	4.56499500	0.21894800
H	2.40186700	2.63964100	-0.74174900
C	0.55306000	5.10402400	1.59624500
H	-0.98264000	3.60674900	1.72002400
C	1.79092000	5.45997300	1.06017400
H	3.41514700	4.82982500	-0.21230900
H	0.02407000	5.79000900	2.25253400
H	2.23352700	6.42472100	1.29371200
C	4.92009500	-1.66271700	-1.04010600
H	5.33864400	-1.01553600	-1.82153300
H	5.73269200	-1.89331700	-0.33968300
C	4.38756400	-2.93974700	-1.69383400
H	4.06090900	-3.65897200	-0.93158200
H	5.19188900	-3.43417900	-2.25312800
C	3.23433700	-2.59611200	-2.63919400
H	2.82734600	-3.50319100	-3.10371900
H	3.61988800	-1.96233000	-3.44816100
C	2.07256600	-1.83294200	-1.94857500
C	3.83212200	-0.85582200	-0.28273600
N	2.70909700	-0.69632000	-1.23838500
C	4.41088500	0.53882300	0.01974500
H	3.75835600	1.13301700	0.66431900
H	5.37340900	0.42748200	0.53181500
H	4.57327700	1.09321900	-0.90956900
C	3.52517400	-1.56475200	1.06659800
H	3.28316600	-2.62323300	0.94260900
H	4.40990700	-1.50819400	1.71227300
H	2.70063500	-1.09313600	1.60482400
C	1.16403700	-1.27029100	-3.05755000
H	0.90537200	-2.07401000	-3.75654400
H	0.22822200	-0.86907800	-2.65989400
H	1.67754500	-0.47821200	-3.61076700
C	1.23638000	-2.82854400	-1.09924100
H	0.39569300	-2.34221800	-0.59870900
H	0.81692000	-3.60030000	-1.75650400
H	1.83105100	-3.33621800	-0.33703300
O	1.85266900	0.42350600	-1.00703300

Supplementary Discussion

Trapping of *C*-centered radical intermediate

Trapping the C-centred intermediate by addition of PhSeSePh or BHT under the standard conditions

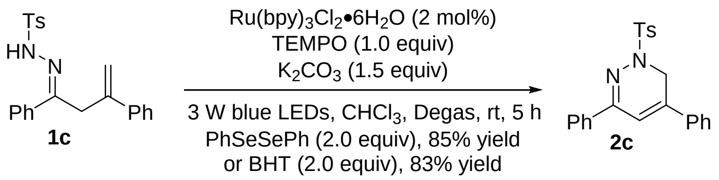

1c (117.0 mg, 0.3 mmol), $Ru(bpy)_3Cl_2{\cdot}6H_2O$ (0.006 mmol), TEMPO (46.9 mg, 0.3 mmol), K_2CO_3 (61.2 mg, 0.45 mmol) and PhSeSePh (2.0 equiv) or BHT (2.0 equiv) were dissolved in $CHCl_3$ (6.0 mL). Then, the resulting mixture was degassed via 'freeze-pump-thaw' procedure (3 times). After that, the solution was stirred at a distance of ~5 cm from a 3W blue LEDs (450-460 nm) at room temperature about 5 h until the reaction was completed as monitored by TLC analysis. The crude product was purified by flash chromatography on silica gel (petroleum ether/ethyl acetate 20:1~10:1) directly to give the product **2c** in 85% and 83% yield, respectively.

Trapping the C-centred intermediate by addition of PhSeSePh under the standard conditions in the absence of TEMPO

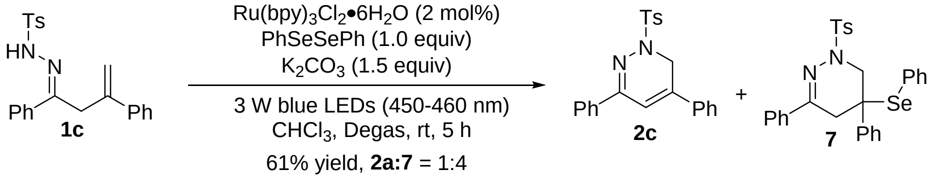

1c (117.0 mg, 0.3 mmol), $Ru(bpy)_3Cl_2{\cdot}6H_2O$ (0.006 mmol), K_2CO_3 (61.2 mg, 0.45 mmol) and PhSeSePh (2.0 equiv) were dissolved in $CHCl_3$ (6.0 mL). Then, the resulting mixture was degassed via 'freeze-pump-thaw' procedure (3 times). After that, the solution was stirred at a distance of ~5 cm from a 3 W blue LEDs (450-460 nm) at room temperature about 48 h until the reaction was completed as monitored by TLC analysis. The crude product was purified by flash chromatography on silica gel (petroleum ether/ethyl acetate 20:1~10:1) directly to give the mixture of **2c** and **7** in 61% yield with 1:4 ratio.

Control experiment with selenide-adduct 7 under the standard conditions

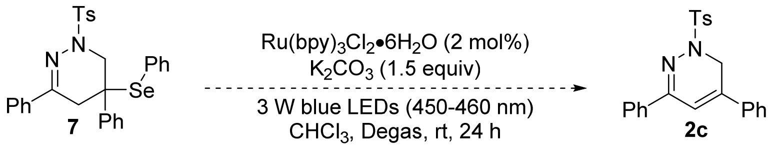

Compound **7** (27.1 mg, 0.05 mmol), $Ru(bpy)_3Cl_2{\cdot}6H_2O$ (0.001 mmol), K_2CO_3 (1.5 eq) were dissolved in $CHCl_3$ (6.0 mL). Then, the resulting mixture was degassed via 'freeze-pump-thaw' procedure (3 times). After that, the solution was stirred at a distance of ~5 cm from a 3W blue LEDs (450-460 nm) at room temperature. However, the desired product **2c** can not be detected from ^1H NMR spectra by using 1,3,5-trrimethoxybenzene as internal standard after 24 h.

The result suggested that the selenide-adduct 7 should not be the possible intermediate for formation of 1,6-dihydropyridazine 2c.

HRMS analysis of reaction mixture upon about 50% conversion of model substrate 1c

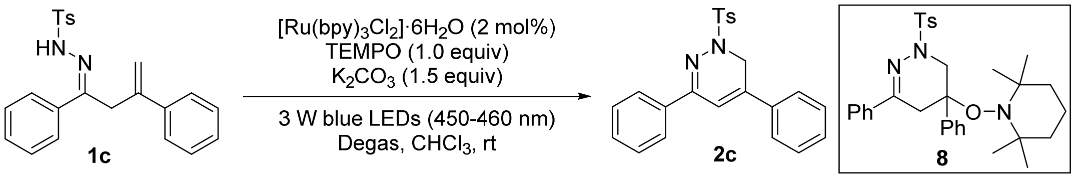

1c (117.0 mg, 0.3 mmol), $Ru(bpy)_3Cl_2{\cdot}6H_2O$ (0.006 mmol), TEMPO (46.9 mg, 0.3 mmol) and K_2CO_3 (61.2 mg, 0.45 mmol) were dissolved in $CHCl_3$ (6.0 mL). Then, the resulting mixture was degassed via 'freeze-pump-thaw' procedure (3 times). After that, the solution was stirred at a distance of ~5 cm from a 3W blue LEDs (450-460 nm) at room temperature, the crude mixture was dectected by HRMS analysis upon about 50% conversion of model substrate **1c**.

Unfortunately, all the attempts to isolate the possible intermediate 8 met failure.

Supplementary Methods

General Information

Unless otherwise noted, materials were purchased from commercial suppliers and used without further purification. All the solvents were treated according to general methods. Flash column chromatography was performed using 200-300 mesh silica gel. ^{1}H NMR spectra were recorded on 400 or 600 MHz spectrophotometers. Chemical shifts are reported in delta (δ) units in parts per million (ppm) relative to the singlet (0 ppm) for tetramethylsilane (TMS). Data are reported as follows: chemical shift, multiplicity (s = ^{13}C NMR spectra were recorded on 100 or 150 MHz with complete proton-decoupling spectrophotometers ($CDCl_3$: 77.0 ppm, or DMSO-d^{6}: 39.5 ppm). ^{19}F NMR spectra were recorded on 376 MHz with complete proton-decoupling spectrophotometers. Mass spectra were measured on MS spectrometer (EI) or LC/MS/MS (ESI-MS). HRMS was recorded on Bruker ultrafleXtreme MALDI-TOF/TOF mass spectrometer.

Experimental Section

General procedure for the preparation of β,γ-unsaturated hydrazones 1b-1v and 5a-5g.[12,13]

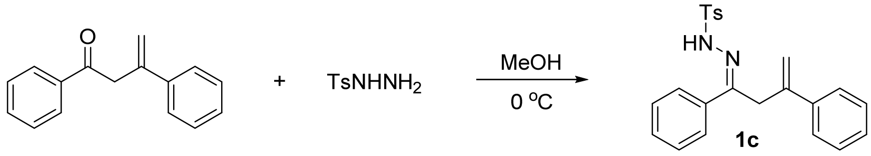

To a stirred solution of β,γ-unsaturated ketone (20 mmol, 1.0 equiv) in MeOH (10 mL), *p*-toluenesulfonyl hydrazide (30 mmol, 1.5 equiv) was added. The mixture was stirred at room temperature until the reaction was completed, as monitored by TLC. Then, the solvent was removed and the residue was purified by flash column chromatography to give compound **1c** as a white solid (3.2 g, 49% yield).

The other β,γ-unsaturated hydrazones were prepared according to the above procedure. **1a** is a kown product.[14] Other substrates were prepared based on the above procedure.

Spectral data of the β,γ-unsaturated hydrazones 1b-1v and 5a-5g

β,γ-Unsaturated hydrazone 1b

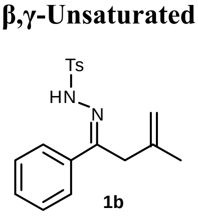

^{1}H NMR (400 MHz, $CDCl_3$) δ (ppm) = 7.90 (s, 1H), 7.86 (d, *J* = 8.2 Hz, 2H), 7.63 – 7.51 (m, 2H), 7.34 – 7.30 (m, 3H), 7.29 (d, *J* = 8.1 Hz, 2H), 4.73 (s, 1H), 4.31 (s, 1H), 3.29 (s, 2H), 2.39 (s, 3H), 1.77 (s, 3H). ^{13}C NMR (100 MHz, $CDCl_3$) δ (ppm) = 153.3, 144.0, 138.0, 136.8, 135.2, 129.5, 129.4, 128.3, 127.9, 126.2, 112.1, 35.9, 22.9, 21.5. M.P.: 145.2 – 146.1 °C. IR (in KBr): 3199, 1596, 1449, 1384, 1350, 1167 cm^{-1}. HRMS (EI): m/z [M + H]$^{+}$ calcd for $C_{18}H_{21}N_2O_2S$: 329.1318; found: 329.1312.

β,γ-Unsaturated hydrazone 1c

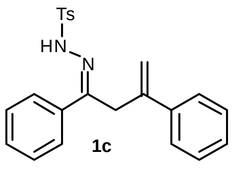

^{1}H NMR (400 MHz, DMSO-d^{6}) δ (ppm) =10.90 (s, 1H), 7.80 (d, *J* = 7.5 Hz, 2H), 7.53 (d, *J* = 8.0 Hz, 4H), 7.43 (d, *J* = 7.4 Hz, 2H), 7.37 – 7.33 (m, 6H), 5.37 (s, 1H), 4.59 (s, 1H), 3.90 (s, 2H), 2.38 (s, 3H). ^{13}C NMR (100 MHz, DMSO-d^{6}) δ (ppm) = 152.2, 143.4, 140.6, 139.7, 137.0, 136.3, 129.6, 129.2, 128.3, 128.3, 127.9, 127.4, 126.1, 125.7, 112.4, 32.4, 21.0. M.P.: 126.9 – 127.7 °C.

IR (in KBr): 3060, 1624, 1474, 1443, 1352, 1166 cm^{-1}. HRMS (EI): m/z $[M + H]^+$ calcd for $C_{23}H_{23}N_2O_2S$: 391.1475; found: 391.1475.

β,γ-Unsaturated hydrazone 1d

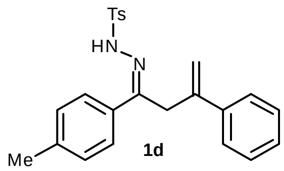

1d

^1H NMR (600 MHz, $CDCl_3$) δ (ppm) = 7.84 (d, *J* = 8.0 Hz, 2H), 7.56 (d, *J* = 8.0 Hz, 2H), 7.52 (s, 1H), 7.43 (d, *J* = 7.4 Hz, 2H), 7.39 – 7.34 (m, 3H), 7.29 (d, *J* = 8.0 Hz, 2H), 7.15 (d, *J* = 7.9 Hz, 2H), 5.27 (s, 1H), 4.49 (s, 1H), 3.74 (s, 2H), 2.41 (s, 3H), 2.35 (s, 3H). ^{13}C NMR (100 MHz, $CDCl_3$) δ (ppm) = 153.3, 143.9, 139.8, 139.5, 139.0, 135.0, 133.7, 129.3, 129.0, 128.4, 128.2, 127.9, 126.1, 125.6, 112.9, 33.1, 21.4, 21.1. M.P.: 151.1 – 152.3 ^oC. IR (in KBr): 3206, 1626, 1595, 1393, 1332, 1166 cm^{-1}. HRMS (EI): m/z $[M + H]^+$ calcd for $C_{24}H_{25}N_2O_2S$: 405.1631; found: 405.1633.

β,γ-Unsaturated hydrazone 1e

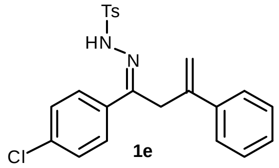

1e

^1H NMR (600 MHz, $CDCl_3$) δ (ppm) = 7.83 (d, *J* = 8.0 Hz, 2H), 7.67 (s, 1H), 7.58 (d, *J* = 8.2 Hz, 2H), 7.41 (d, *J* = 6.6 Hz, 2H), 7.38 – 7.36 (m, 3H), 7.30 (d, *J* = 8.1 Hz, 4H), 5.30 (s, 1H), 4.50 (s, 1H), 3.72 (s, 2H), 2.42 (s, 3H). ^{13}C NMR (100 MHz, $CDCl_3$) δ (ppm) = 152.0, 144.2, 139.3, 138.8, 135.5, 135.0, 134.9, 129.5, 128.5, 128.4, 127.8, 127.5, 125.6, 113.1, 33.1, 21.5. M.P.: 134.4 – 134.7 ^oC. IR (in KBr): 3209, 1624, 1472, 1390, 1330, 1164 cm^{-1}. HRMS (EI): m/z $[M + H]^+$ calcd for $C_{23}H_{22}ClN_2O_2S$: 425.1085; found: 425.1084.

β,γ-Unsaturated hydrazone 1f

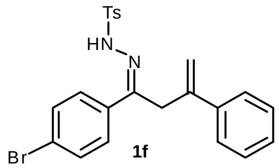

1f

^1H NMR (600 MHz, $CDCl_3$) δ (ppm) = 7.82 (d, *J* = 8.0 Hz, 2H), 7.70 (s, 1H), 7.51 (d, *J* = 8.4 Hz, 2H), 7.45 (d, *J* = 8.4 Hz, 2H), 7.40 (d, *J* = 6.5 Hz, 2H), 7.36 (d, *J* = 6.7 Hz, 3H), 7.30 (d, *J* = 8.0 Hz, 2H), 5.29 (s, 1H), 4.50 (s, 1H), 3.71 (s, 2H), 2.42 (s, 3H). ^{13}C NMR (100 MHz, $CDCl_3$) δ (ppm) = 152.0, 144.2, 139.3, 138.8, 135.4, 134.9, 131.5, 129.5, 128.5, 128.4, 127.9, 127.8, 125.6, 124.0, 113.1, 33.0, 21.5. M.P.: 134.1 – 134.7 ^oC. IR (in KBr): 3201, 1628, 1492, 1385, 1342, 1164 cm^{-1}. HRMS (EI): m/z $[M + H]^+$ calcd for $C_{23}H_{22}BrN_2O_2S$: 469.0580; found: 469.0567.

β,γ-Unsaturated hydrazone 1g

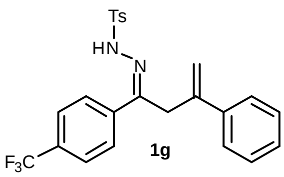

1g

^1H NMR (400 MHz, $CDCl_3$) δ (ppm) = 7.86 – 7.77 (m, 3H), 7.75 (d, *J* = 8.3 Hz, 2H), 7.59 (d, *J* = 8.3 Hz, 2H), 7.43 – 7.41 (m, 2H), 7.39 – 7.36 (m, 3H), 7.31 (d, *J* = 8.2 Hz, 2H), 5.31 (s, 1H), 4.52 (s, 1H), 3.76 (s, 2H), 2.42 (s, 3H). ^{13}C NMR (100 MHz, $CDCl_3$) δ (ppm) = 151.5, 144.3, 140.0, 139.2, 138.9, 134.8, 131.1 (q, *J* = 32.2 Hz), 129.5, 128.6, 128.5, 127.9, 126.5, 125.6, 125.3 (q, *J* = 3.2 Hz), 123.8 (q, *J* = 270.5 Hz), 113.2, 33.2, 21.5. ^{19}F NMR (376 MHz, $CDCl_3$) δ(ppm) = -64.6 (s, 3F). M.P.: 150.3 – 151.3 ^oC. IR (in KBr): 3196, 1626, 1338, 1328, 1167, 1113 cm^{-1}. HRMS (EI): m/z $[M + H]^+$ calcd for $C_{24}H_{22}F_3N_2O_2S$: 459.1349; found: 459.1349.

β,γ-Unsaturated hydrazone 1h

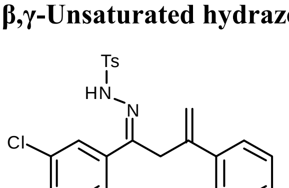

1h

^1H NMR (600 MHz, $CDCl_3$) δ (ppm) = 7.84 (d, *J* = 8.1 Hz, 2H), 7.68 (s, 1H), 7.61 (s, 1H), 7.50 (d, *J* = 7.8 Hz, 1H), 7.42 (d, *J* = 6.6 Hz, 2H), 7.39 – 7.35 (m, 3H), 7.32 (t, *J* = 7.8 Hz,

3H), 7.26 (d, *J* = 6.1 Hz, 1H), 5.31 (s, 1H), 4.52 (s, 1H), 3.72 (s, 2H), 2.43 (s, 3H). ^{13}C NMR (100 MHz, $CDCl_3$) δ (ppm) = 151.6, 144.2, 139.2, 138.8, 138.4, 134.8, 134.3, 129.6, 129.4, 128.4, 128.3, 127.8, 126.2, 125.6, 124.4, 113.1, 33.1, 21.5. M.P.: 151.6 – 152.8 °C. IR (in KBr): 3049, 1624, 1592, 1393, 1343, 1164 cm^{-1}. HRMS (EI): m/z [M + H]$^+$ calcd for $C_{23}H_{22}ClN_2O_2S$: 425.1085; found: 425.1085.

β,γ-Unsaturated hydrazone 1i

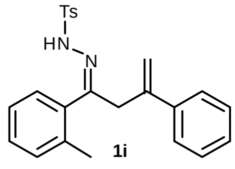

^{1}H NMR (400 MHz, $CDCl_3$) δ (ppm) = 7.82 (s, 1H), 7.77 (d, *J* = 8.3 Hz, 2H), 7.31 – 7.27 (m, 7H), 7.20 – 7.18 (m, 1H), 7.14 – 7.12 (m, 3H), 5.39 (s, 1H), 4.85 (s, 1H), 3.76 (s, 2H), 2.43 (s, 3H), 2.16 (s, 3H). ^{13}C NMR (100 MHz, $CDCl_3$) δ (ppm) = 155.1, 144.0, 140.6, 139.0, 137.3, 136.4, 135.3, 131.0, 129.4, 128.6, 128.5, 128.4, 128.0, 125.8, 125.5, 114.2, 37.5, 21.6, 20.6. M.P.: 138.4 – 139.3 °C. IR (in KBr): 3220, 1623, 1494, 1397, 1333, 1162 cm^{-1}. HRMS (EI): m/z [M + H]$^+$ calcd for $C_{24}H_{25}N_2O_2S$: 405.1631; found: 405.1630.

β,γ-Unsaturated hydrazone 1j

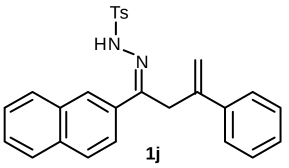

^{1}H NMR (600 MHz, $CDCl_3$) δ (ppm) = 7.98 (d, *J* = 8.7 Hz, 1H), 7.92 (s, 1H), 7.88 (d, *J* = 8.1 Hz, 2H), 7.81 (s, 1H), 7.80 (d, *J* = 3.0 Hz, 1H), 7.78 (d, *J* = 7.9 Hz, 1H), 7.70 (s, 1H), 7.51 – 7.44 (m, 4H), 7.41 – 7.36 (m, 3H), 7.29 (d, *J* = 8.0 Hz, 2H), 5.31 (s, 1H), 4.54 (s, 1H), 3.87 (s, 2H), 2.39 (m, 3H). ^{13}C NMR (100 MHz, $CDCl_3$) δ (ppm) = 153.2, 144.1, 139.5, 139.0, 135.1, 134.0, 133.8, 132.8, 129.5, 128.6, 128.5, 128.4, 128.1, 128.0, 127.6, 127.0, 126.4, 126.3, 125.7, 123.5, 113.1, 33.1, 21.6. M.P.: 150.4 – 151.8 °C. IR (in KBr): 3202, 1596, 1477, 1383, 1339, 1166 cm^{-1}. HRMS (EI): m/z [M + H]$^+$ calcd for $C_{27}H_{25}N_2O_2S$: 441.1631; found: 441.1630.

β,γ-Unsaturated hydrazone 1k

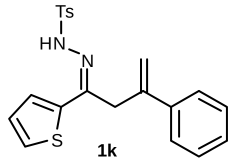

^{1}H NMR (400 MHz, $CDCl_3$) δ (ppm) = 7.85 (d, *J* = 8.3 Hz, 2H), 7.51 (s, 1H), 7.40 – 7.29 (m, 8H), 7.18 – 7.17 (m, 1H), 6.97 – 6.94 (m, 1H), 5.25 (s, 1H), 4.54 (s, 1H), 3.75 (s, 2H), 2.42 (s, 3H). ^{13}C NMR (100 MHz, $CDCl_3$) δ (ppm) = 149.5, 144.1, 142.0, 139.3, 138.9, 134.8, 129.4, 128.6, 128.6, 128.4, 128.1, 127.2, 127.0, 125.6, 113.3, 33.8, 21.6. M.P.: 145.3 – 146.7 °C. IR (in KBr): 3204, 1596, 1494, 1389, 1348, 1166 cm^{-1}. HRMS (EI): m/z [M + H]$^+$ calcd for $C_{21}H_{21}N_2O_2S_2$: 397.1039; found: 397.1039.

β,γ-Unsaturated hydrazone 1l

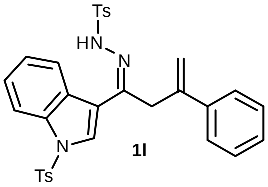

^{1}H NMR (600 MHz, $CDCl_3$) δ (ppm) = 8.26 (d, *J* = 7.9 Hz, 1H), 7.91 (d, *J* = 8.3 Hz, 1H), 7.84 (d, *J* = 8.3 Hz, 2H), 7.72 (s, 1H), 7.71 (d, *J* = 3.4 Hz, 2H), 7.67 (s, 1H), 7.45 – 7.43 (m, 2H), 7.41 – 7.38 (m, 3H), 7.35 – 7.34 (m, 1H), 7.31 – 7.26 (m, 3H), 7.22 (d, *J* = 8.2 Hz, 2H), 5.33 (s, 1H), 4.63 (s, 1H), 3.75 (s, 2H), 2.38 (s, 3H), 2.34 (s, 3H). ^{13}C NMR (100 MHz, $CDCl_3$) δ (ppm) = 149.5, 145.4, 144.2, 139.7, 138.8, 135.2, 135.0, 134.4, 129.9, 129.5, 128.6, 128.4, 127.9, 127.4, 126.8, 126.7, 125.6, 125.4, 124.1, 124.0, 120.3, 113.3, 113.0, 34.2, 21.5. M.P.: 158.4 – 159.6 °C. IR (in KBr): 3214, 1597, 1555, 1443, 1379, 1171 cm^{-1}. HRMS (EI): m/z [M + H]$^+$ calcd for $C_{32}H_{30}N_3O_4S_2$: 584.1672; found: 584.1663.

β,γ-Unsaturated hydrazone 1m

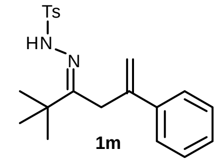

1m

^{1}H NMR (600 MHz, $CDCl_3$) δ (ppm) = 7.83 (d, *J* = 7.8 Hz, 2H), 7.39 – 7.34 (m, 5H), 7.32 (d, *J* = 8.1 Hz, 2H), 7.28 (s, 1H), 5.21 (s, 1H), 4.15 (s, 1H), 3.32 (s, 2H), 2.46 (s, 3H), 1.08 (s, 9H). ^{13}C NMR (100 MHz, $CDCl_3$) δ (ppm) = 163.2, 143.9, 139.4, 139.2, 134.9, 129.2, 128.5, 128.4, 128.1, 125.6, 112.4, 39.0, 32.0, 27.3, 21.6. M.P.: 138.4 – 138.7 ^{o}C. IR (in KBr): 3201, 1626, 1597, 1476, 1400, 1340, 1172 cm^{-1}. HRMS (EI): m/z [M + H]$^{+}$ calcd for $C_{21}H_{27}N_2O_2S$: 371.1788; found: 371.1788.

β,γ-Unsaturated hydrazone 1n

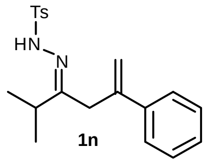

1n

^{1}H NMR (400 MHz, DMSO-d^{6}) δ (ppm) = 10.19 (s, 1H), 7.71 (d, *J* = 8.2 Hz, 2H), 7.43 – 7.38 (m, 4H), 7.33 – 7.27 (m, 3H), 5.46 (s, 1H), 4.81 (s, 1H), 3.44 (s, 2H), 2.38 (s, 3H), 2.29 (m, 1H), 0.86 (s, 3H), 0.85 (s, 3H). ^{13}C NMR (100 MHz, $CDCl_3$) δ (ppm) = 161.9, 143.8, 139.7, 139.1, 135.0, 129.1, 128.4, 128.2, 127.9, 125.6, 113.0, 36.2, 34.2, 21.5, 19.5. M.P.: 106.5 – 107.7 ^{o}C. IR (in KBr): 2971, 1617, 1595, 1495, 1390, 1164 cm^{-1}. HRMS (EI): m/z [M + H]$^{+}$ calcd for $C_{20}H_{25}N_2O_2S$: 357.1631; found: 357.1630.

β,γ-Unsaturated hydrazone 1o

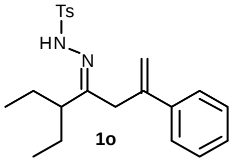

1o

^{1}H NMR (400 MHz, DMSO-d^{6}) δ (ppm) = 10.24 (s, 1H), 7.72 (d, *J* = 8.2 Hz, 2H), 7.42 – 7.37 (m, 4H), 7.33 – 7.28 (m, 3H), 5.51 (s, 1H), 4.97 (s, 1H), 3.42 (s, 2H), 2.37 (s, 3H), 1.95 – 1.92 (m, 1H), 1.38 – 1.27 (m, 2H), 1.23 – 1.15 (m, 2H), 0.44 (t, *J* = 7.3 Hz, 6H). ^{13}C NMR (100 MHz, $CDCl_3$) δ (ppm) = 159.7, 143.8, 140.2, 139.4, 135.2, 129.2, 128.5, 128.3, 128.0, 125.8, 113.7, 50.1, 34.9, 24.3, 21.6, 11.5. M.P.: 104.2 – 104.6 ^{o}C. IR (in KBr): 3218, 1622, 1495, 1458, 1331, 1158 cm^{-1}. HRMS (EI): m/z [M + H]$^{+}$ calcd for $C_{22}H_{29}N_2O_2S$: 385.1944; found: 385.1944.

β,γ-Unsaturated hydrazone 1p

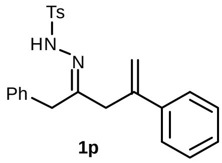

1p

^{1}H NMR (400 MHz, $CDCl_3$) δ (ppm) = 7.74 (d, *J* = 8.2 Hz, 2H), 7.57 (s, 1H), 7.30 – 7.28 (m, 5H), 7.24 – 7.20 (m, 5H), 7.03 – 7.01 (m, 2H), 5.24 (s, 1H), 4.59 (s, 1H), 3.49 (s, 2H), 3.22 (s, 2H), 2.46 (s, 3H). ^{13}C NMR (100 MHz, $CDCl_3$) δ (ppm) = 157.0, 143.8, 139.9, 139.1, 136.0, 135.0, 129.3, 129.1, 128.4, 128.3, 128.2, 128.0, 126.7, 125.6, 114.2, 43.4, 34.6, 21.7. M.P.: 92.9 – 93.6 ^{o}C. IR (in KBr): 3223, 1622, 1493, 1451, 1396, 1130, 1163 cm^{-1}. HRMS (EI): m/z [M + H]$^{+}$ calcd for $C_{24}H_{25}N_2O_2S$: 405.1631; found: 405.1641.

β,γ-Unsaturated hydrazone 1q

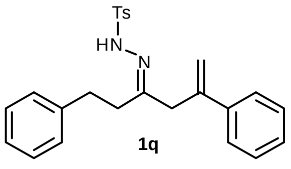

1q

^{1}H NMR (400 MHz, $CDCl_3$) (major : minor = 1:0.6) δ (ppm) = 7.78 (d, *J* = 8.1 Hz, 2H, major), 7.66 (d, *J* = 8.0 Hz, 2H, minor), 7.41 (s, 1H, major), 7.32 – 7.28 (m, 8 H, major + minor), 7.23 – 7.12 (m, 10H, major + minor), 7.08 (d, *J* = 7.3 Hz, 2H, major), 6.87 (d, *J* = 6.7 Hz, 2H, minor), 6.80 (d, *J* = 4.4 Hz, 1H, minor), 5.49 (s, 1H, minor), 5.27 (s, 1H, major), 5.05 (s, 1H, minor), 4.60 (s, 1H, major), 3.38 (s, 2H, minor), 3.31 (s, 2H, major), 2.83 – 2.75 (m, 2H, major), 2.65 – 2.61 (m, 2H, minor), 2.54 – 2.50 (m, 2H, major), 2.47 (s, 3H, minor), 2.44 (s, 3H, major), 2.44 – 2.40 (m, 2H, minor). ^{13}C NMR (100 MHz, $CDCl_3$) δ (ppm) = 158.7, 157.7, 143.9, 143.6, 142.7, 141.1, 140.1, 139.9, 139.2, 139.1, 135.1, 135.0, 129.3, 129.2, 128.6, 128.4, 128.2, 128.1, 128.1, 128.0, 127.9, 127.5, 126.5, 125.9, 125.8, 125.6, 115.7, 114.0, 43.7, 38.6, 36.3, 31.6,

30.9, 30.4, 21.5. M.P.: 122.0 – 122.7 °C. IR (in KBr): 3223, 1597, 1495, 1403, 1339, 1166 cm^{-1}. HRMS (EI): m/z $[M + H]^+$ calcd for $C_{25}H_{27}N_2O_2S$: 419.1788; found: 419.1789.

β,γ-Unsaturated hydrazone 1r

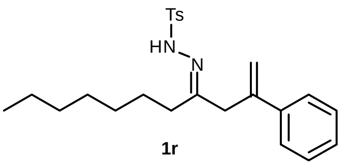

1r

^{1}H NMR (400 MHz, $CDCl_3$) δ (ppm) = 7.90 (s, 1H), 7.79 (d, *J* = 8.2 Hz, 2H), 7.26 (d, *J* = 7.7 Hz, 4H), 7.20 – 7.16 (m, 1H), 7.12 (t, *J* = 7.3 Hz, 2H), 5.40 (s, 1H), 4.96 (s, 1H), 3.39 (s, 2H), 2.44 (s, 3H), 2.10 – 2.00 (m, 2H), 1.33 – 1.27 (m, 2H), 1.24 – 1.19 (m, 2H), 1.14 (s, 6H), 0.84 (t, *J* = 7.1 Hz, 3H). ^{13}C NMR (100 MHz, $CDCl_3$) δ (ppm) = 159.5, 143.6, 143.0, 139.6, 135.4, 129.4, 128.1, 127.8, 127.4, 125.8, 115.4, 43.2, 31.5, 29.5, 28.8, 28.3, 24.8, 22.5, 21.5, 14.0. M.P.: 110.4 – 110.7 °C. IR (in KBr): 3228, 1619, 1463, 1392, 1333, 1166 cm^{-1}. HRMS (EI): m/z $[M + H]^+$ calcd for $C_{24}H_{33}N_2O_2S$: 413.2257; found: 413.2254.

β,γ-Unsaturated hydrazone 1s

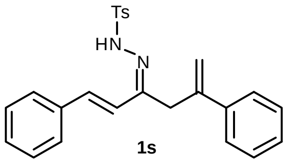

1s

^{1}H NMR (600 MHz, $CDCl_3$) δ (ppm) = 7.88 (s, 1H), 7.83 (d, *J* = 8.1 Hz, 2H), 7.38 (d, *J* = 4.1 Hz, 2H), 7.32 (d, *J* = 5.9 Hz, 5H), 7.28 (d, *J* = 8.0 Hz, 2H), 7.24 – 7.19 (m, 3H), 6.97 (d, *J* = 16.2 Hz, 1H), 6.75 (d, *J* = 16.3 Hz, 1H), 5.37 (s, 1H), 4.97 (s, 1H), 3.70 (s, 2H), 2.44 (s, 3H). ^{13}C NMR (100 MHz, $CDCl_3$) δ (ppm) = 153.3, 145.0, 143.8, 140.3, 139.2, 135.1, 129.6, 129.4, 128.7, 128.1, 127.9, 127.4, 126.9, 125.9, 125.6, 115.4, 114.9, 40.3, 21.5. M.P.: 148.5 – 149.5 °C. IR (in KBr): 3223, 1625, 1493, 1342, 1304, 1164 cm^{-1}. HRMS (EI): m/z $[M + H]^+$ calcd for $C_{25}H_{25}N_2O_2S$: 417.1631; found: 417.1632.

β,γ-Unsaturated hydrazone 1t

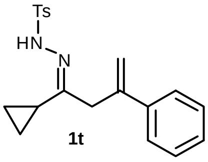

1t

^{1}H NMR (600 MHz, $CDCl_3$) δ (ppm) = 7.76 (d, *J* = 8.1 Hz, 2H), 7.35 – 7.28 (m, 7H), 7.18 (s, 1H), 5.29 (s, 1H), 4.64 (s, 1H), 3.24 (s, 2H), 2.45 (s, 3H), 1.59 – 1.55 (m, 1H), 0.70 – 0.67 (m, 4H). ^{13}C NMR (100 MHz, $CDCl_3$) δ (ppm) = 159.9, 143.8, 140.1, 139.1, 134.9, 129.2, 128.4, 128.1, 127.9, 125.6, 113.6, 34.9, 21.5, 16.8, 7.2. M.P.: 105.6 – 106.2 °C. IR (in KBr): 3209, 1632, 1493, 1373, 1337, 1165 cm^{-1}. HRMS (EI): m/z $[M + H]^+$ calcd for $C_{20}H_{23}N_2O_2S$: 355.1475; found: 355.1466.

β,γ-Unsaturated hydrazone 1u

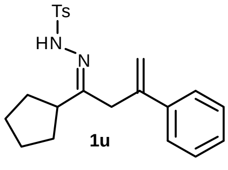

1u

^{1}H NMR (400 MHz, DMSO-d^6) δ (ppm) = 10.22 (s, 1H), 7.71 (d, *J* = 7.4 Hz, 2H), 7.44 – 7.39 (m, 4H), 7.34 – 7.29 (m, 3H), 5.48 (s, 1H), 4.86 (s, 1H), 3.43 (s, 2H), 2.39 (s, 3H), 1.55 – 1.38 (m, 9H). ^{13}C NMR (100 MHz, $CDCl_3$) δ (ppm) = 160.6, 143.8, 139.8, 139.2, 135.0, 129.2, 128.4, 128.2, 127.9, 125.6, 112.9, 47.3, 34.7, 29.6, 25.0, 21.5. M.P.: 104.7 – 105.2 °C. IR (in KBr): 3204, 1628, 1492, 1454, 1386, 1337, 1164 cm^{-1}. HRMS (EI): m/z $[M + H]^+$ calcd for $C_{22}H_{27}N_2O_2S$: 383.1788; found: 383.1788.

β,γ-Unsaturated hydrazone 1v

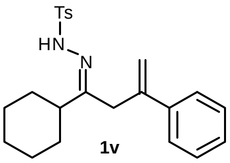

1v

^{1}H NMR (400 MHz, $CDCl_3$) δ (ppm) = 7.80 (d, *J* = 7.9 Hz, 2H), 7.33 – 7.31 (m, 8H), 5.21 (s, 1H), 4.35 (s, 1H), 3.30 (s, 2H), 2.45 (s, 3H), 2.15 – 2.11 (m, 1H), 1.73 – 1.66 (m, 4H), 1.30 – 1.16 (m, 6H). ^{13}C NMR (100 MHz, $CDCl_3$) δ (ppm) = 161.4, 143.8, 139.7, 139.2, 135.1, 129.2, 128.5,

128.3, 128.0, 125.6, 112.8, 46.2, 34.5, 29.9, 25.9, 25.8, 21.6. M.P.: 104.5 – 104.7 °C. IR (in KBr): 3199, 1626, 1596, 1493, 1392, 1338, 1158 cm^{-1}. HRMS (EI): m/z [M + H]$^+$ calcd for $C_{23}H_{29}N_2O_2S$: 397.1944; found: 397.1946.

β,γ-Unsaturated hydrazone 5a

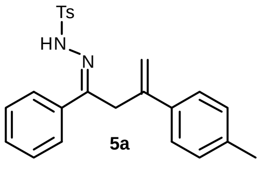

^{1}H NMR (600 MHz, CDCl$_3$) δ (ppm) = 7.84 (d, *J* = 8.1 Hz, 2H), 7.65 (d, *J* = 6.9 Hz, 2H), 7.58 (s, 1H), 7.36 – 7.32 (m, 5H), 7.30 (d, *J* = 8.1 Hz, 2H), 7.18 (d, *J* = 7.9 Hz, 2H), 5.26 (s, 1H), 4.47 (s, 1H), 3.74 (s, 2H), 2.41 (s, 3H), 2.38 (s, 3H). ^{13}C NMR (100 MHz, CDCl$_3$) δ (ppm) = 153.4, 144.0, 139.2, 138.3, 136.6, 136.0, 135.1, 129.6, 129.4, 129.2, 128.3, 127.9, 126.2, 125.5, 112.1, 33.4, 21.5, 21.0. M.P.: 145.3 – 145.9 °C. IR (in KBr): 3214, 1596, 1479, 1396, 1362, 1166 cm^{-1}. HRMS (EI): m/z [M + H]$^+$ calcd for $C_{24}H_{25}N_2O_2S$: 405.1631; found: 405.1631.

β,γ-Unsaturated hydrazone 5b

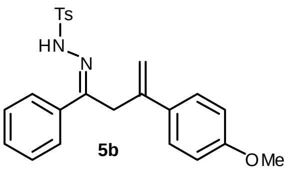

^{1}H NMR (400 MHz, CDCl$_3$) δ (ppm) = 10.87 (s, 1H), 7.80 (d, *J* = 8.0 Hz, 2H), 7.54 – 7.53 (m, 2H), 7.47 – 7.44 (m, 4H), 7.33 – 7.32 (m, 3H), 6.91 (d, *J* = 8.5 Hz, 2H), 5.28 (s, 1H), 4.50 (s, 1H), 3.86 (s, 2H), 3.77 (s, 3H), 2.38 (s, 3H). ^{13}C NMR (100 MHz, CDCl$_3$) δ (ppm) = 159.7, 153.5, 144.0, 138.6, 136.6, 135.0, 131.2, 129.6, 129.4, 128.3, 127.9, 126.8, 126.2, 113.8, 111.2, 55.2, 33.5, 21.5. M.P.: 132.6 – 133.0 °C. IR (in KBr): 3161, 1599, 1511, 1330, 1237, 1171 cm^{-1}. HRMS (EI): m/z [M + H]$^+$ calcd for $C_{24}H_{25}N_2O_3S$: 421.1580; found: 421.1581.

β,γ-Unsaturated hydrazone 5c

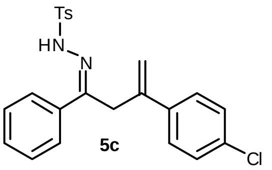

^{1}H NMR (400 MHz, CDCl$_3$) δ (ppm) = 7.83 (d, *J* = 8.3 Hz, 2H), 7.78 (s, 1H), 7.64 – 7.61 (m, 2H), 7.37 – 7.35 (m, 3H), 7.33 (t, *J* = 2.1 Hz, 2H), 7.31 (t, *J* = 2.0 Hz, 2H), 7.30 – 7.28 (m, 1H), 5.29 (s, 1H), 4.60 (s, 1H), 3.71 (s, 2H), 2.42 (s, 3H). ^{13}C NMR (100 MHz, CDCl$_3$) δ (ppm) = 152.8, 144.1, 138.5, 137.5, 136.5, 135.0, 134.0, 129.6, 129.5, 128.5, 128.3, 127.9, 127.0, 126.2, 113.7, 33.2, 21.5. M.P.: 155.2 – 155.7 °C. IR (in KBr): 3201, 1628, 1595, 1385, 1343, 1164 cm^{-1}. HRMS (EI): m/z [M + H]$^+$ calcd for $C_{23}H_{22}ClN_2O_2S$: 425.1085; found: 425.1086.

β,γ-Unsaturated hydrazone 5d

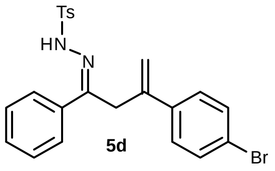

^{1}H NMR (400 MHz, CDCl$_3$) δ (ppm) = 7.84 (d, *J* = 7.6 Hz, 2H), 7.68 (s, 1H), 7.62 – 7.60 (s, 2H), 7.46 (d, *J* = 7.7 Hz, 2H), 7.35 -7.33 (m, 3H), 7.31 – 7.26 (m, 4H), 5.30 (s, 1H), 4.61 (s, 1H), 3.72 (s, 2H), 2.42 (s, 3H). ^{13}C NMR (100 MHz, CDCl$_3$) δ (ppm) = 152.9, 144.1, 138.6, 138.0, 136.5, 134.9, 131.4, 129.6, 129.5, 128.3, 127.9, 127.3, 126.2, 122.2, 113.8, 33.1, 21.6. M.P.: 159.4 – 159.7 °C. IR (in KBr): 3201, 1628, 1595, 1388, 1333, 1163 cm^{-1}. HRMS (EI): m/z [M + H]$^+$ calcd for $C_{23}H_{22}BrN_2O_2S$: 469.0580; found: 469.0580.

β,γ-Unsaturated hydrazone 5e

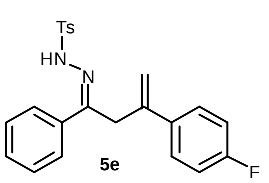

^{1}H NMR (600 MHz, CDCl$_3$) δ (ppm) = 7.85 (d, *J* = 7.8 Hz, 2H), 7.64 (d, *J* = 7.9 Hz, 3H), 7.40 – 7.37 (m, 5H), 7.30 (d, *J* = 8.0 Hz, 2H), 7.04 (t, *J* = 8.3 Hz, 2H), 5.25 (s, 1H), 4.55 (s, 1H), 3.72 (s, 2H), 2.42 (s, 3H). ^{13}C NMR (100 MHz, CDCl$_3$) δ (ppm) δ = 163.6 (d, *J* = 246.4 Hz),

153.0, 144.1, 138.6, 136.6, 135.1 (d, J = 3.3 Hz), 135.0, 129.6, 129.5, 128.4, 127.9, 127.4 (d, J = 8.1 Hz), 126.2, 115.3 (d, J = 21.3 Hz), 113.1, 33.4, 21.5. ^{19}F NMR (376 MHz, $CDCl_3$) δ(ppm) = -114.3 (s, 1F). M.P.: 152.7 – 153.2 °C. IR (in KBr): 3212, 1625, 1598, 1506, 1393, 1329, 1166 cm^{-1}. HRMS (EI): m/z [M + H]$^+$ calcd for $C_{23}H_{22}FN_2O_2S$: 409.1381; found: 409.1381.

β,γ-Unsaturated hydrazone 5f

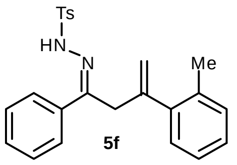

5f

^{1}H NMR (600 MHz, $CDCl_3$) δ (ppm) = 7.81 (d, J = 8.1 Hz, 2H), 7.63 (d, J = 7.6 Hz, 2H), 7.41 (s, 1H), 7.38 – 7.33 (m, 3H), 7.31 (d, J = 8.2 Hz, 2H), 7.20 (t, J = 7.4 Hz, 1H), 7.17 (d, J = 7.5 Hz, 1H), 7.10 (t, J = 7.4 Hz, 1H), 6.98 (d, J = 7.5 Hz, 1H), 4.94 (s, 1H), 4.91 (s, 1H), 3.63 (s, 2H), 2.43 (s, 3H), 2.25 (s, 3H). ^{13}C NMR (100 MHz, $CDCl_3$) δ (ppm) = 153.2, 144.0, 141.7, 140.7, 136.6, 135.0, 134.4, 130.5, 129.6, 129.4, 128.3, 128.1, 127.8, 127.7, 126.3, 126.0, 116.7, 36.5, 21.6, 19.7. M.P.: 119.5 – 120.1 °C. IR (in KBr): 3213, 1598, 1489, 1375, 1320, 1165 cm^{-1}. HRMS (EI): m/z [M + H]$^+$ calcd for $C_{24}H_{25}N_2O_2S$: 405.1631; found: 405.1631.

β,γ-Unsaturated hydrazone 5g

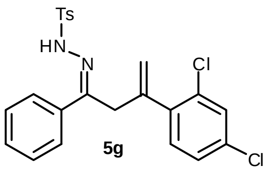

5g

(major : minor = 10:1). ^{1}H NMR (600 MHz, $CDCl_3$) δ (ppm) = 7.90 (s, 1H), 7.86 (d, J = 8.2 Hz, 2H), 7.65 (d, J = 7.7 Hz, 2H), 7.41 (s, 1H), 7.35 (d, J = 7.1 Hz, 3H), 7.30 (d, J = 8.1 Hz, 2H), 7.19 (d, J = 8.2 Hz, 1H), 7.04 (d, J = 8.1 Hz, 1H), 4.93 (s, 1H), 4.81 (s, 1H), 3.61 (s, 2H), 2.42 (s, 3H). ^{13}C NMR (100 MHz, $CDCl_3$) δ (ppm) = 151.6, 144.1, 139.4, 138.3, 136.4, 135.1, 134.3, 132.3, 130.8, 129.7, 129.5, 129.3, 128.4, 127.9, 127.4, 126.2, 118.0, 34.7, 21.5. M.P.: 157.4 – 158.0 °C. IR (in KBr): 3203, 1593, 1468, 1383, 1344, 1165 cm^{-1}. HRMS (EI): m/z [M + H]$^+$ calcd for $C_{23}H_{21}Cl_2N_2O_2S$: 459.0695; found: 459.0698.

General procedure for the 5-*exo*-cyclization of β,γ-unsaturated hydrazones 1a and 1b

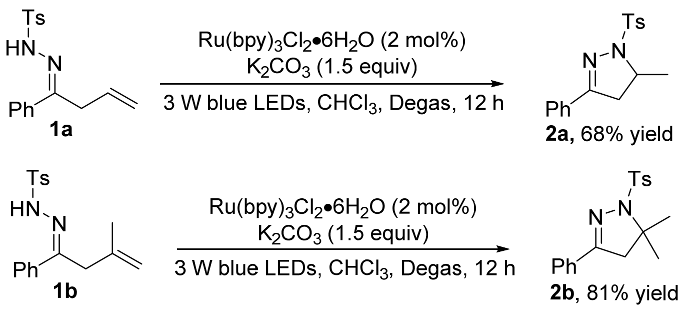

1a or **1b** (0.2 mmol), $Ru(bpy)_3Cl_2{\cdot}6H_2O$ (0.004 mmol), K_2CO_3 (41.5 mg, 0.3 mmol) were dissolved in $CHCl_3$ (4.0 mL). Then, the resulting mixture was degassed via 'freeze-pump-thaw' procedure (3 times). After that, the solution was stirred at a distance of ~5 cm from a 3 W blue LEDs (450-460 nm) at room temperature about 12 h until the reaction was completed as monitored by TLC analysis. The crude product was purified by flash chromatography on silica gel (petroleum ether/ethyl acetate 20:1~10:1) directly to give the the corresponding product **2a** and **2b** in 68% yield and 81% yield, respectively. Product **2a** is a kown compound.[14]

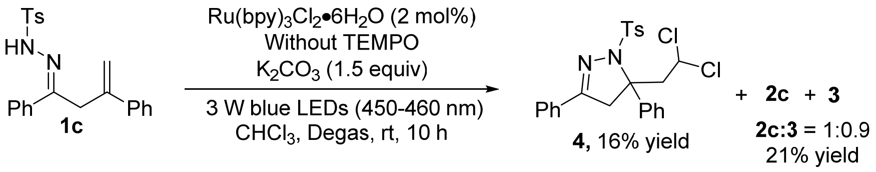

1c (0.2 mmol, 78.0 mg), Ru(bpy)$_3$Cl$_2$·6H$_2$O (0.004 mmol), K_2CO_3 (41.5 mg, 0.3 mmol) were dissolved in $CHCl_3$ (4.0 mL). Then, the resulting mixture was degassed via 'freeze-pump-thaw' procedure (3 times). After that, the solution was stirred at a distance of ~5 cm from a 3W blue LEDs (450-460 nm) at room temperature about 10h until the reaction was completed as monitored by TLC analysis. The crude product was purified by flash chromatography on silica gel (petroleum ether/ethyl acetate 30:1~20:1) directly to give the product **4** in 16% yield and a mixture of **2c** and **3** in 21% yield with 1:0.9 ratio.

General procedure for the synthesis of 1,6-dihydropyridazines 2c-2v and 6a-6g

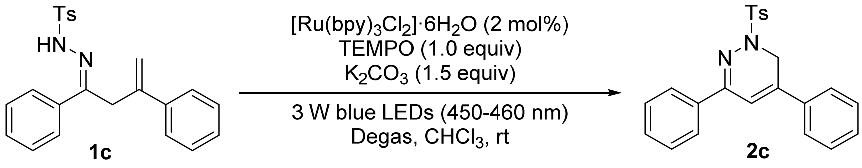

1c (117.0 mg, 0.3 mmol), Ru(bpy)$_3$Cl$_2$·6H$_2$O (0.006 mmol), TEMPO (46.9 mg, 0.3 mmol) and K_2CO_3 (61.2 mg, 0.45 mmol) were dissolved in $CHCl_3$ (6.0 mL). Then, the resulting mixture was degassed via 'freeze-pump-thaw' procedure (3 times). After that, the solution was stirred at a distance of ~5 cm from a 3W blue LEDs (450-460 nm) at room temperature about 5 h until the reaction was completed as monitored by TLC analysis. The crude product was purified by flash chromatography on silica gel (petroleum ether/ethyl acetate 20:1~10:1) directly to give the desired product **2c** in 84% yield as a white solid.

General procedure for the synthesis of pyridazines 15a-15l

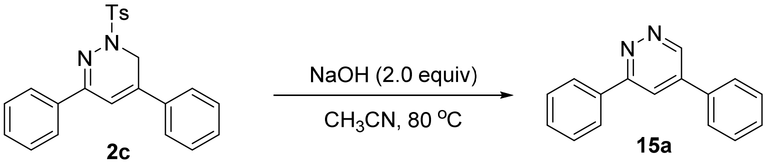

2c (77.6 mg, 0.2 mmol) and NaOH (16 mg, 0.4 mmol) were dissolved in CH_3CN (4.0 mL). Then, the resulting mixture was sitrred at 80 ^{o}C until the reaction was completed as monitored by TLC analysis. The crude product was purified by flash chromatography on silica gel (petroleum ether/ethyl acetate 10:1~5:1) directly to give the product **15a** in 90% yield as a white solid.

Gram-scale experiment for the synthesis of 2c

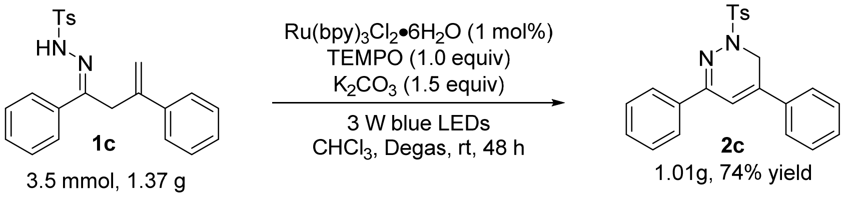

1c (1.37 g, 3.5 mmol), Ru(bpy)$_3$Cl$_2$·6H$_2$O (0.035 mmol), TEMPO (546.9 mg, 3.5 mmol) and K_2CO_3 (732.5 mg, 5.3 mmol) were dissolved in $CHCl_3$ (15.0 mL). Then, the resulting mixture was degassed via 'freeze-pump-thaw' procedure

(3 times). After that, the solution was stirred at a distance of ~5 cm from a 3W blue LEDs (450-460 nm) at room temperature about 24 h until the reaction was completed as monitored by TLC analysis. The crude product was purified by flash chromatography on silica gel (petroleum ether/ethyl acetate 20:1~10:1) directly to give the desired product **2c** in 74% yield as a white solid.

One-pot process for the synthesis of 1,6-dihydropyridazine 2d

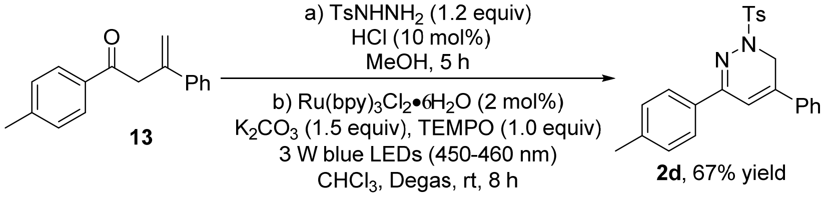

To a stirred solution of β,γ-unsaturated ketone **13** (70.8 mg, 0.3 mmol) in MeOH (3 mL), *p*-toluenesulfonyl hydrazide (0.45 mmol, 1.5 equiv) and HCl (10 mol%) was added. The solution was stirred at at room temperature for about 5 h until the reaction was completed as monitored by TLC analysis. After that, MeOH was removed and $Ru(bpy)_3Cl_2{\cdot}6H_2O$ (0.006 mmol), TEMPO (0.3 mmol, 46.9 mg) and K_2CO_3 (61.2 mg, 0.45 mmol) in $CHCl_3$ (6.0 mL) was added to the reaction system. Then, the resulting mixture was degassed via 'freeze-pump-thaw' procedure (3 times). After that, the solution was stirred at a distance of ~5 cm from a 3W blue LEDs (450-460 nm) at room temperature about 8 h until the reaction was completed as monitored by TLC analysis. The crude product was purified by flash chromatography on silica gel (petroleum ether/ethyl acetate 20:1~10:1) directly to give the desired adduct **2d** in 67% yield as a white solid.

Procedure for the synthesis of pyridazine *N*-oxide 14

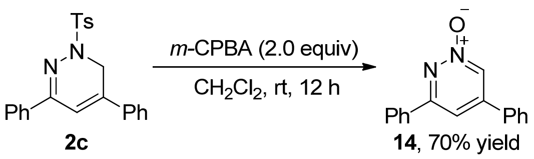

2c (116.4 mg, 0.3 mmol) were dissolved in CH_2Cl_2 (3.0 mL). Then, *m*-CPBA (2.0 equiv) was addded to the mixture. The solution was stirred at at room temperature for about 12 h until the reaction was completed as monitored by TLC analysis. The crude product was purified by flash chromatography on silica gel (petroleum ether/ethyl acetate 10:1~3:1) to give the desired product **14** in 70% yield as a white solid.

General procedure for the synthesis of diazinium salts 17a-17e

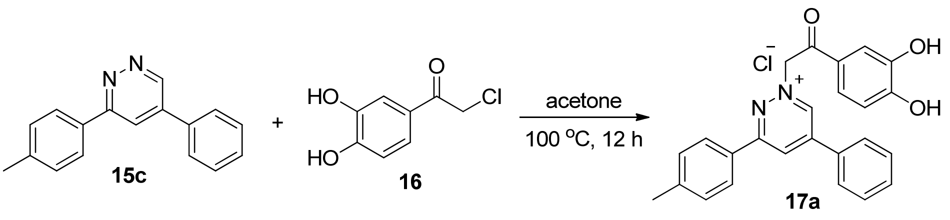

15c (73.8 mg, 0.3 mmol) were dissolved in acetone (3.0 mL). Then, **16** (2.0 equiv) was addded to the mixture and stirred at 100 ^{o}C for about 12 h. The crude product were filtered off, washed 2 times with 10 mL of Et_2O and dried in vacuo to give the desired product **17a** in 85% yield as a white solid.

The other diazinium salts were prepared according to the above procedure.

Spectral data of the products

Product 2b

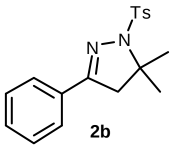

2b

Yield of **2b** : 81% as a white solid. ^{1}H NMR (400 MHz, $CDCl_3$) δ (ppm) = 7.93 (d, *J* = 8.3 Hz, 2H), 7.66 – 7.64 (m, 2H), 7.39 – 7.37 (m, 3H), 7.29 – 7.27 (d, *J* = 8.0 Hz, 2H), 3.04 (s, 2H), 2.42 (s, 3H), 1.56 (s, 6H). ^{13}C NMR (100 MHz, $CDCl_3$) δ (ppm) = 153.4, 143.4, 137.2, 131.4, 130.0, 129.2, 128.5, 128.0, 126.4, 69.0, 49.2, 27.0, 21.5. M.P.: 162.7 – 163.5 °C. IR (in KBr): 1597, 1493, 1449, 1368, 1157, 702 cm^{-1}. HRMS (EI): m/z $[M + H]^+$ calcd for $C_{18}H_{21}N_2O_2S$: 329.1318; found: 329.1311.

Product 2c

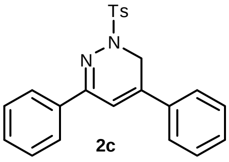

2c

Yield of **2c** : 84% as a white solid. ^{1}H NMR (600 MHz, $CDCl_3$) δ (ppm) δ 7.95 (d, *J* = 8.2 Hz, 2H), 7.79 – 7.77 (m, 2H), 7.55 (d, *J* = 7.0 Hz, 2H), 7.46 – 7.43 (m, 3H), 7.41 – 7.40 (m, 3H), 7.36 (d, *J* = 8.1 Hz, 2H), 6.79 (s, 1H), 4.33 (s, 2H), 2.42 (s, 3H). ^{13}C NMR (100 MHz, $CDCl_3$) δ (ppm) = 150.7, 144.3, 139.7, 135.2, 134.9, 132.0, 129.8, 129.6, 129.5, 128.8, 128.7, 128.4, 125.8, 125.4, 112.4, 43.3, 21.4. M.P.: 133.6 – 134.9 °C. IR (in KBr): 1622, 1530, 1494, 1446, 1359, 1171 cm^{-1}. HRMS (ESI): m/z $[M+H]^+$ calcd for $C_{23}H_{21}N_2O_2S$: 389.1318; found: 389.1340.

Product 2d

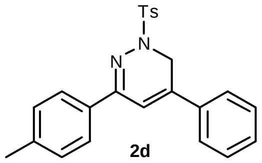

2d

Yield of **2d** : 85% as a white solid. ^{1}H NMR (600 MHz, $CDCl_3$) δ (ppm) = 7.92 (d, *J* = 8.1 Hz, 2H), 7.68 (d, *J* = 8.0 Hz, 2H), 7.54 (d, *J* = 7.7 Hz, 2H), 7.45 – 7.41 (m, 3H), 7.35 (d, *J* = 8.0 Hz, 2H), 7.21 (d, *J* = 8.0 Hz, 2H), 6.77 (s, 1H), 4.31 (s, 2H), 2.42 (s, 3H), 2.38 (s, 3H). ^{13}C NMR (100 MHz, $CDCl_3$) δ (ppm) = 150.8, 144.3, 139.8, 139.8, 135.4, 132.3, 132.1, 129.8, 129.5, 129.2, 128.9, 128.8, 125.8, 125.5, 112.7, 43.4, 21.6, 21.3. M.P.: 168.2 – 168.8 °C. IR (in KBr): 1628, 1492, 1445, 1356, 1170, 1094 cm^{-1} HRMS (EI): m/z $[M + H]^+$ calcd for $C_{24}H_{23}N_2O_2S$: 403.1475; found: 403.1475.

Product 2e

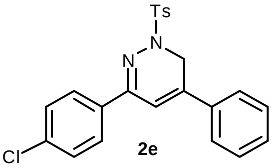

2e

Yield of **2e** : 74% as a white solid. ^{1}H NMR (600 MHz, $CDCl_3$) δ (ppm) = 7.91 (d, *J* = 7.9 Hz, 2H), 7.71 (d, *J* = 8.2 Hz, 2H), 7.54 (d, *J* = 6.7 Hz, 2H), 7.46 – 7.43 (m, 3H), 7.37 (*t*, *J* = 9.1 Hz, 4H), 4.34 (s, 2H), 2.43 (s, 3H). ^{13}C NMR (100 MHz, $CDCl_3$) δ (ppm) = 149.7, 144.4, 140.1, 135.6, 135.2, 133.5, 132.0, 130.0, 129.6, 128.9, 128.7, 128.6, 127.1, 125.5, 112.0, 43.3, 21.5. M.P.: 151.1 – 151.3 °C. IR (in KBr): 1628, 1493, 1400, 1358, 1168, 1093 cm^{-1}. HRMS (EI): m/z $[M + H]^+$ calcd for $C_{23}H_{20}ClN_2O_2S$: 423.0929; found: 423.0929.

Product 2f

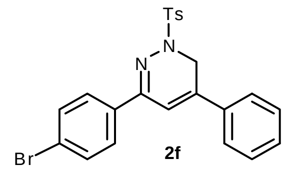

2f

Yield of **2f** : 70% as a white solid. ^{1}H NMR (600 MHz, $CDCl_3$) δ (ppm) = 7.91 (d, *J* = 8.2 Hz, 2H), 7.65 (d, *J* = 8.4 Hz, 2H), 7.54 (d, *J* = 7.1 Hz, 4H), 7.47 – 7.42 (m, 3H), 7.36 (d, *J* = 8.1 Hz, 2H), 6.73 (s, 1H), 4.34 (s, 2H), 2.43 (s, 3H). ^{13}C NMR (100 MHz, $CDCl_3$) δ (ppm) = 149.8, 144.5, 140.2, 135.2, 134.0, 132.0, 131.6, 130.0, 129.6, 128.9, 128.7, 127.4, 125.5, 124.0, 112.0, 43.3, 21.6. M.P.: 142.9 – 143.7 °C. IR (in KBr): 1627, 1591, 1491, 1358, 1167, 1094 cm^{-1}. HRMS (EI): m/z $[M + H]^+$ calcd for $C_{23}H_{20}BrN_2O_2S$: 467.0423; found: 467.0418.

Product 2g

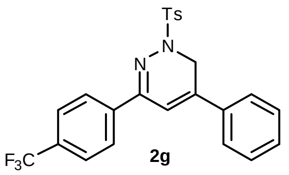

Yield of **2g** : 61% as a white solid. ^{1}H NMR (600 MHz, $CDCl_3$) δ (ppm) = 7.92 (d, *J* = 7.7 Hz, 2H), 7.88 (d, *J* = 8.0 Hz, 2H), 7.66 (d, *J* = 7.9 Hz, 2H), 7.55 (d, *J* = 7.4 Hz, 2H), 7.46 (d, *J* = 6.6 Hz, 3H), 7.37 (d, *J* = 7.8 Hz, 2H), 6.77 (s, 1H), 4.38 (s, 2H), 2.43 (s, 3H). ^{13}C NMR (100 MHz, $CDCl_3$) δ (ppm) = 149.4, 144.6, 140.2, 138.4, 135.1, 132.0, 131.2 (q, *J* = 32.3 Hz), 130.2, 129.7, 129.0, 128.7, 126.1, 125.5, 125.4 (q, *J* = 3.7 Hz), 121.2 (q, *J* = 270.6 Hz), 111.9, 43.4, 21.6. ^{19}F NMR (376 MHz, $CDCl_3$) δ(ppm) = -64.5 (s, 3F). M.P.: 177.5 – 178.3 °C. IR (in KBr): 1630, 1530, 1362, 1323, 1168, 1115 cm^{-1}. HRMS (EI): m/z [M + H]$^+$ calcd for $C_{24}H_{20}F_3N_2O_2S$: 457.1192; found: 457.1190.

Product 2h

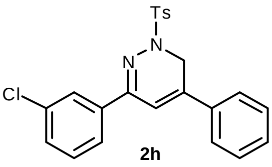

Yield of **2h** : 78% as a white solid. ^{1}H NMR (400 MHz, $CDCl_3$) δ (ppm) = 7.92 (d, *J* = 8.2 Hz, 2H), 7.74 (s, 1H), 7.64 (d, *J* = 7.1 Hz, 1H), 7.55 – 7.53 (m, 2H), 7.47 - 7.43 (m, 3H), 7.38 – 7.31 (m, 4H), 6.72 (s, 1H), 4.34 (s, 2H), 2.42 (s, 3H). ^{13}C NMR (100 MHz, $CDCl_3$) δ (ppm) = 149.5, 144.5, 140.2, 136.9, 135.2, 134.6, 132.0, 130.1, 129.8, 129.6, 129.6, 129.0, 128.8, 126.0, 125.5, 124.0, 112.1, 43.4, 21.6. M.P.: 136.4 – 136.7 °C. IR (in KBr): 1631, 1594, 1446, 1359, 1168, 1091 cm^{-1}. HRMS (EI): m/z [M + H]$^+$ calcd for $C_{23}H_{20}ClN_2O_2S$: 423.0929; found: 423.0936.

Product 2i

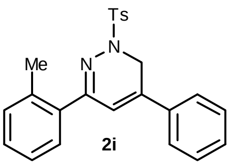

Yield of **2i** : 72% as a white solid. ^{1}H NMR (400 MHz, $CDCl_3$) δ (ppm) = 7.88 (d, *J* = 8.2 Hz, 2H), 7.53 – 7.50 (m, 2H), 7.45 – 7.41 (m, 3H), 7.34 (d, *J* = 8.2 Hz, 3H), 7.28 (s, 1H), 7.24 – 7.20 (m, 2H), 6.47 (s, 1H), 4.38 (s, 2H), 2.43 (s, 3H), 2.36 (s, 3H). ^{13}C NMR (100 MHz, $CDCl_3$) δ (ppm) = 153.1, 144.3, 139.1, 136.5, 135.6, 135.2, 132.4, 131.0, 129.9, 129.5, 128.9, 128.8, 128.5, 125.8, 125.5, 115.6, 43.0, 21.6, 20.8. M.P.: 148.9 – 149.6 °C. IR (in KBr): 1625, 1597, 1348, 1170, 1096, 955 cm^{-1}. HRMS (EI): m/z [M + H]$^+$ calcd for $C_{24}H_{23}N_2O_2S$: 403.1475; found: 403.1464.

Product 2j

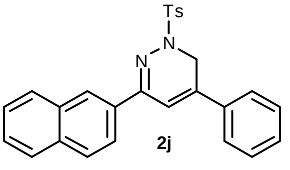

Yield of **2j** : 86% as a white solid. ^{1}H NMR (600 MHz, $CDCl_3$) δ (ppm) = 8.11 (s, 1H), 8.05 (d, *J* = 8.7 Hz, 1H), 7.96 (d, *J* = 8.3 Hz, 2H), 7.88 – 7.84 (m, 3H), 7.59 (d, *J* = 7.0 Hz, 2H), 7.53 – 7.47 (m, 2H), 7.46 – 7.43 (m, 3H), 7.36 (d, *J* = 8.1 Hz, 2H), 6.96 (s, 1H), 4.38 (s, 2H), 2.41 (s, 3H). ^{13}C NMR (100 MHz, $CDCl_3$) δ (ppm) = 150.6, 144.4, 139. 9, 135.5, 133.9, 132.9, 132.5, 132.1, 130.0, 129.6, 129.0, 128.8, 128.5, 128.4, 127.7, 126.9, 126.5, 125.6, 123.3, 112.6, 43.6, 21.6. M.P.: 169.6 – 170.8 °C. IR (in KBr): 1629, 1494, 1446, 1362, 1171, 1096 cm^{-1}. HRMS (EI): m/z [M + H]$^+$ calcd for $C_{27}H_{23}N_2O_2S$: 439.1475; found: 439.1475.

Product 2k

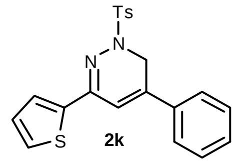

Yield of **2k** : 59% as a white solid. ^{1}H NMR (600 MHz, $CDCl_3$) δ (ppm) = 7.93 (d, *J* = 8.1 Hz, 2H), 7.53 (d, *J* = 7.9 Hz, 2H), 7.45 – 7.43 (m, 3H), 7.37 – 7.35 (m, 4H), 7.04 – 7.03 (m, 1H), 6.72 (s, 1H), 4.32 (s, 2H), 2.43 (s, 3H). ^{13}C NMR (100 MHz, $CDCl_3$) δ (ppm) = 146.8, 144.4, 140.1, 139.9, 135.2, 131.9, 130.0, 129.5, 129.0, 128.9, 127.9, 127.3, 125.9, 125.5, 112.1, 43.7, 21.6.

M.P.: 155.8 – 156.4 °C. IR (in KBr): 1627, 1493, 1436, 1352, 1170, 1098 cm^{-1}. HRMS (EI): m/z [M + H]$^{+}$ calcd for $C_{21}H_{19}N_2O_2S_2$: 395.0882; found: 395.0884.

Product 2l

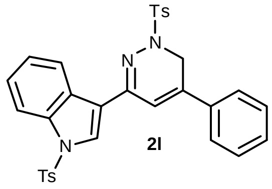

2l

Yield of **2l** : 53% as a green solid. ^{1}H NMR (600 MHz, $CDCl_3$) δ (ppm) = 8.44 (d, *J* = 7.3 Hz, 1H), 7.96 (d, *J* = 8.0 Hz, 1H), 7.93 (d, *J* = 7.9 Hz, 2H), 7.87 (s, 1H), 7.78 (d, *J* = 7.8 Hz, 2H), 7.58 (d, *J* = 7.6 Hz, 2H), 7.49 – 7.45 (m, 3H), 7.41 – 7.36 (m, 2H), 7.33 (s, 2H), 7.23 (d, *J* = 7.9 Hz, 2H), 4.39 (s, 2H), 2.39 (s, 3H), 2.35 (s, 3H). ^{13}C NMR (100 MHz, $CDCl_3$) δ (ppm) = 147.2, 146.6, 145.3, 144.4, 140.0, 135.2, 135.1, 134.5, 132.0, 130.0, 129.9, 129.6, 129.5, 128.9, 128.6, 127.5, 126.8, 125.5, 125.4, 124.2, 123.9, 118.7, 113.0, 112.4, 43.4, 21.5. M.P.: 186.2 – 186.9 °C. IR (in KBr): 1641, 1444, 1378, 1358, 1269, 1171 cm^{-1}. HRMS (EI): m/z [M + H]$^{+}$ calcd for $C_{32}H_{28}N_3O_4S$: 582.1516; found: 582.1528.

Product 2m

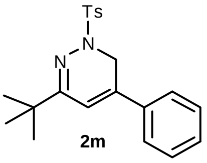

2m

Yield of **2m** : 63% as a white solid. ^{1}H NMR (400 MHz, $CDCl_3$) δ (ppm) = 7.87 (d, *J* = 8.3 Hz, 2H), 7.48 – 7.45 (m, 2H), 7.44 – 7.38 (m, 3H), 7.35 (d, *J* = 8.2 Hz, 2H), 6.37 (t, *J* = 1.2 Hz, 1H), 4.13 (d, *J* = 1.3 Hz, 2H), 2.45 (s, 3H), 1.16 (s, 9H). ^{13}C NMR (100 MHz, $CDCl_3$) δ (ppm) = 161.0, 144.1, 139.6, 135.6, 131.9, 129.6, 129.2, 129.0, 128.8, 125.4, 112.3, 43.2, 36.5, 27.9, 21.6. M.P.: 153.2 – 154.1 °C. IR (in KBr): 1633, 1554, 1495, 1449, 1352, 1171 cm^{-1}. HRMS (EI): m/z [M + H]$^{+}$ calcd for $C_{21}H_{25}N_2O_2S$: 369.1631; found: 369.1642.

Product 2n

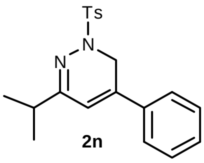

2n

Yield of **2n** : 72% as a white solid. ^{1}H NMR (600 MHz, $CDCl_3$) δ (ppm) = 7.87 (d, *J* = 8.1 Hz, 2H), 7.46 (d, *J* = 6.6 Hz, 2H), 7.42 – 7.39 (m, 3H), 7.35 (d, *J* = 8.0 Hz, 2H), 6.23 (s, 1H), 4.16 (s, 2H), 2.64 – 2.60 (m, 1H), 2.44 (s, 3H), 1.14 (s, 3H), 1.13 (s, 3H).. ^{13}C NMR (100 MHz, $CDCl_3$) δ (ppm) = 159.1, 144.0, 139.6, 135.3, 131.8, 129.6, 129.2, 128.8, 128.7, 125.2, 113.1, 43.3, 33.7, 21.5, 20.0. M.P.: 122.5 – 123.8 °C. IR (in KBr): 1631, 1594, 1494, 1447, 1351, 1170 cm^{-1}. HRMS (EI): m/z [M + H]$^{+}$ calcd for $C_{20}H_{23}N_2O_2S$: 355.1475; found: 355.1475.

Product 2o

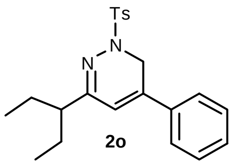

2o

Yield of **2o**: 71% as a white solid. ^{1}H NMR (600 MHz, $CDCl_3$) δ (ppm) = 7.87 (d, *J* = 8.2 Hz, 2H), 7.46 – 7.45 (m, 2H), 7.40 (q, *J* = 6.1 Hz, 3H), 7.33 (d, *J* = 8.1 Hz, 2H), 6.16 (s, 1H), 4.17 (s, 2H), 2.43 (s, 3H), 2.25 – 2.20 (m, 1H), 1.56 – 1.46 (m, 4H), 0.77 (t, *J* = 7.4 Hz, 6H). ^{13}C NMR (100 MHz, $CDCl_3$) δ (ppm) = 157.2, 144.0, 139.8, 135.3, 131.9, 129.7, 129.3, 128.8, 128.8, 125.3, 112.7, 48.1, 43.6, 25.4, 21.6, 11.6. M.P.: 105.7 – 106.3 °C. IR (in KBr): 1632, 1596, 1494, 1382, 1353, 1169 cm^{-1}. HRMS (EI): m/z [M + H]$^{+}$ calcd for $C_{22}H_{27}N_2O_2S$: 383.1788; found: 383.1783.

Product 2p

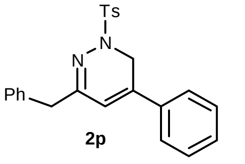

2p

Yield of **2p** : 83% as a white solid. ^{1}H NMR (600 MHz, $CDCl_3$) δ (ppm) = 7.87 (d, *J* = 8.2 Hz, 2H), 7.37 – 7.35 (m, 7H), 7.25 (s, 2H), 7.23 -7.22 (m, 1H), 7.15 (d, *J* = 7.1 Hz, 2H), 6.08 (s, 1H), 4.20 (s, 2H), 3.68 (s, 2H), 2.46 (s, 3H). ^{13}C NMR (100 MHz, $CDCl_3$) δ (ppm) = 153.5, 144.1, 139.6, 136.3, 134.8, 131.8, 129.7, 129.3, 128.7, 128.7, 128.6, 128.4, 126.6, 125.2, 114.0, 43.2, 41.5, 21.4. M.P.: 138.5 – 139.2 ^{o}C. IR (in KBr): 1636, 1495, 1448, 1355, 1172, 1094 cm^{-1}. HRMS (EI): m/z [M + H]$^{+}$ calcd for $C_{24}H_{23}N_2O_2S$: 403.1475; found: 403.1474.

Product 2q

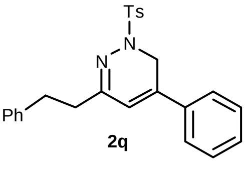

2q

Yield of **2q** : 70% as a white solid. ^{1}H NMR (600 MHz, $CDCl_3$) δ (ppm) = 7.83 (d, *J* = 8.2 Hz, 2H), 7.42 – 7.39 (m, 5H), 7.33 (d, *J* = 8.0 Hz, 2H), 7.22 (t, *J* = 7.3 Hz, 2H), 7.17 – 7.15 (m, 1H), 7.13 (d, *J* = 7.1 Hz, 2H), 6.12 (s, 1H), 4.15 (s, 2H), 2.89 (t, *J* = 7.8 Hz, 2H), 2.68 (t, *J* = 7.8 Hz, 2H), 2.45 (s, 3H). ^{13}C NMR (100 MHz, $CDCl_3$) δ (ppm) = 154.4, 144.1, 140.7, 139.7, 135.1, 131.9, 129.8, 129.4, 128.9, 128.8, 128.3, 128.2, 125.9, 125.4, 114.7, 43.3, 36.9, 32.7, 21.6. M.P.: 143.2 – 144.7 ^{o}C. IR (in KBr): 1632, 1494, 1420, 1382, 1170, 1096 cm^{-1}. HRMS (EI): m/z [M + H]$^{+}$ calcd for $C_{25}H_{25}N_2O_2S$: 417.1631; found: 417.1632.

Product 2r

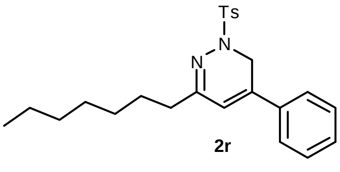

2r

Yield of **2r** : 74% as a white solid. ^{1}H NMR (600 MHz, $CDCl_3$) δ (ppm) = 7.87 (d, *J* = 8.1 Hz, 2H), 7.46 (d, *J* = 6.7 Hz, 2H), 7.42 – 7.38 (m, 3H), 7.34 (d, *J* = 8.0 Hz, 2H), 6.17 (s, 1H), 4.17 (s, 2H), 2.44 (s, 3H), 2.44 – 2.34 (m, 2H), 1.54 – 1.50 (m, 2H), 1.28 – 1.20 (m, 8H), 0.89 – 0.88 (t, *J* = 7.1 Hz, 3H). ^{13}C NMR (100 MHz, $CDCl_3$) δ (ppm) = 155.3, 144.1, 139.6, 135.2, 132.1, 129.8, 129.4, 128.9, 128.8, 125.4, 114.6, 43.3, 35.4, 31.8, 29.0, 28.9, 26.7, 22.6, 21.6, 14.1. M.P.: 101.7 – 102.0 ^{o}C. IR (in KBr): 1636, 1496, 1354, 1170, 1094, 896 cm^{-1}. HRMS (EI): m/z [M + Na]$^{+}$ calcd for $C_{24}H_{31}N_2O_2S$: 433.1920; found: 433.1930.

Product 2s

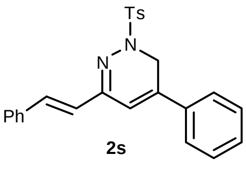

2s

Yield of **2s** : 70% as a white solid. ^{1}H NMR (400 MHz, $CDCl_3$) δ (ppm) = 7.83 (d, *J* = 8.1 Hz, 2H), 7.46 (d, *J* = 6.4 Hz, 2H), 7.40 (d, *J* = 7.5 Hz, 2H), 7.36 (d, *J* = 6.9 Hz, 3H), 7.29 (d, *J* = 7.4 Hz, 4H), 7.23 (d, *J* = 7.1 Hz, 1H), 6.95 – 6.82 (m, 2H), 6.63 (s, 1H), 4.23 (s, 2H), 2.35 (s, 3H). ^{13}C NMR (100 MHz, $CDCl_3$) δ (ppm) = 150.9, 144.4, 139.4, 135.9, 135.4, 133.0, 132.1, 129.9, 129.6, 128.9, 128.8, 128.7, 128.6, 126.9, 125.7, 125.5, 111.1, 43.9, 21.6. M.P.: 166.8 – 167.2 ^{o}C. IR (in KBr): 1621, 1596, 1445, 1358, 1170, 1093 cm^{-1}. HRMS (EI): m/z [M + H]$^{+}$ calcd for $C_{25}H_{23}N_2O_2S$: 415.1475; found: 415.1480.

Product 2t

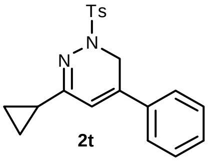

2t

Yield of **2t** : 83% as a white solid. ^{1}H NMR (600 MHz, $CDCl_3$) δ (ppm) = 7.97 (d, *J* = 8.2 Hz, 2H), 7.58 – 7.56 (m, 2H), 7.54 – 7.52 (m, 3H), 7.48 (d, *J* = 8.0 Hz, 2H), 6.17 (s, 1H), 4.28 (s, 2H), 2.57 (s, 3H), 1.81 – 1.79 (m, 1H), 0.98 – 0.94 (m, 4H). ^{13}C NMR (100 MHz, $CDCl_3$) δ (ppm) = 156.1, 144.0, 139.7, 135.1, 131.8, 129.6, 129.2, 128.7, 128.6, 125.3, 112.8, 43.4, 21.4, 15.1, 6.9. M.P.: 128.5 –

129.7 °C . IR (in KBr): 1631, 1494, 1445, 1405, 1171, 1094 cm^{-1}. HRMS (EI): m/z [M + H]$^+$ calcd for $C_{20}H_{21}N_2O_2S$: 353.1318; found: 353.1319.

Product 2u

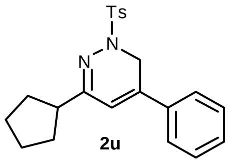

Yield of **2u** : 86% as a white solid. ^{1}H NMR (600 MHz, $CDCl_3$) δ (ppm) = 7.87 (d, *J* = 8.1 Hz, 2H), 7.45 (d, *J* = 6.9 Hz, 2H), 7.41 – 7.38 (m, 3H), 7.35 (d, *J* = 8.0 Hz, 2H), 6.20 (s, 1H), 4.17 (s, 2H), 2.82 – 2.78 (m, 1H), 2.44 (s, 3H), 1.86 – 1.85 (m, 2H), 1.66 – 1.60 (m, 6H). ^{13}C NMR (100 MHz, $CDCl_3$) δ (ppm) = 157.8, 144.0, 139.6, 135.3, 131.9, 129.6, 129.3, 128.8, 128.8, 125.3, 113.9, 44.8, 43.4, 30.2, 25.3, 21.5. M.P.: 131.7 – 132.8 °C. IR (in KBr): 1629, 1494, 1445, 1353, 1171, 1093 cm^{-1}. HRMS (EI): m/z [M + H]$^+$ calcd for $C_{22}H_{25}N_2O_2S$: 381.1631; found: 381.1639.

Product 2v

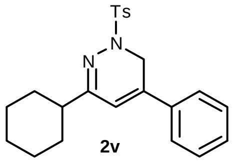

Yield of **2v** : 96% as a white solid. ^{1}H NMR (600 MHz, $CDCl_3$) δ (ppm) = 7.87 (d, *J* = 8.1 Hz, 2H), 7.45 (d, *J* = 6.7 Hz, 2H), 7.41 – 7.38 (m, 3H), 7.35 (d, *J* = 8.0 Hz, 2H), 6.23 (s, 1H), 4.15 (s, 2H), 2.44 (s, 3H), 2.30 – 2.27 (s, 1H), 1.83 – 1.79 (m, 4H), 1.70 (d, *J* = 12.9 Hz, 1H), 1.36 – 1.27 (m, 5H). ^{13}C NMR (100 MHz, $CDCl_3$) δ (ppm) = 158.5, 144.0, 139.5, 135.3, 131.9, 129.6, 129.3, 128.8, 128.7, 125.3, 113.5, 43.5, 43.4, 30.2, 25.8, 25.8, 21.5. M.P.: 136.1 – 137.1 °C. IR (in KBr): 1633, 1495, 1447, 1384, 1347, 1169, 1094 cm^{-1}. HRMS (EI): m/z [M + H]$^+$ calcd for $C_{23}H_{27}N_2O_2S$: 395.1788; found: 395.1788.

Product 3

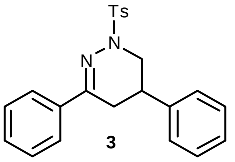

Yield of **3** : 11% as a white solid. ^{1}H NMR (400 MHz, $CDCl_3$) δ (ppm) = 7.83 (d, *J* = 8.0 Hz, 2H), 7.75 – 7.73 (m, 2H), 7.38 – 7.35 (m, 5H), 7.31 – 7.29 (m, 3H), 7.20 (d, *J* = 7.7 Hz, 2H), 4.22 – 4.18 (m, 1H), 3.38 – 3.35 (m, 1H), 2.97 (dd, *J* = 18.3 Hz, 6.1 Hz, 1H), 2.81 (t, *J* = 10.8 Hz, 1H), 2.57 (dd, *J* = 18.4 Hz, 10.5 Hz, 1H), 2.41 (s, 3H). ^{13}C NMR (100 MHz, $CDCl_3$) δ (ppm) = 150.0, 144.0, 141.2, 136.6, 133.4, 129.5, 129.0, 128.4, 128.4, 127.6, 127.0, 125.5, 48.2, 36.1, 30.4, 21.5. M.P.: 193.7 – 194.2 °C. IR (in KBr): 1597, 1494, 1453, 1166, 1089, 1004 cm^{-1}. HRMS (EI): m/z [M + H]$^+$ calcd for $C_{23}H_{23}N_2O_2S$: 391.1475; found: 391.1474.

Product 4

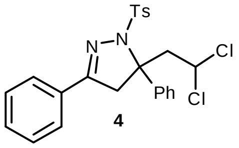

Yield of **4** : 16% as a white solid. ^{1}H NMR (400 MHz, $CDCl_3$) δ (ppm) = 7.75 – 7.72 (m, 2H), 7.44 – 7.41 (m, 3H), 7.31 (d, *J* = 8.3 Hz, 2H), 7.24 – 7.20 (m, 1H), 7.14 – 7.07 (m, 4H), 7.01 (d, *J* = 8.1 Hz, 2H), 6.10 (t, *J* = 5.8 Hz, 1H), 4.18 (d, *J* = 17.8 Hz, 1H), 3.69 (d, *J* = 5.9 Hz, 2H), 3.52 (d, *J* = 17.8 Hz, 1H), 2.32 (s, 3H). ^{13}C NMR (100 MHz, $CDCl_3$) δ (ppm) = 153.5, 143.2, 140.1, 136.0, 130.6, 130.5, 128.8, 128.7, 128.6, 128.4, 127.3, 126.7, 126.0, 73.0, 69.9, 50.8, 49.0, 21.5. M.P.: 195.3 – 195.8 °C. IR (in KBr): 1597, 1495, 1447, 1354, 1166, 1088, 758 cm^{-1}. HRMS (EI): m/z [M + H]$^+$ calcd for $C_{24}H_{23}Cl_2N_2O_2S$: 473.0852; found: 473.0852.

Product 6a

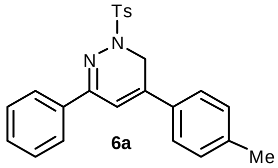

Yield of **6a** : 81% as a white solid. ^{1}H NMR (600 MHz, $CDCl_3$) δ (ppm) = 7.93 (d, *J* = 8.1 Hz, 2H), 7.80 – 7.75 (m, 2H), 7.45 (d, *J* = 8.0 Hz, 2H), 7.41 – 7.40 (m, 3H), 7.35 (d, *J* = 8.1 Hz, 2H), 7.24 (s, 2H), 6.76 (s, 1H), 4.31 (s, 2H), 2.42 (s, 3H), 2.39 (s, 3H). ^{13}C NMR (100 MHz, $CDCl_3$) δ (ppm) = 150.9, 144.2, 140.2, 139.8, 135.1, 132.4, 132.1, 129.5, 129.5, 128.7, 128.4, 125.8, 125.3, 111.6, 43.3, 21.5, 21.2. M.P.: 167.2 – 167.8 ^{o}C. IR (in KBr): 1599, 1513, 1358, 1172, 1091, 903 cm^{-1}. HRMS (EI): m/z $[M + H]^+$ calcd for $C_{24}H_{23}$ N_2O_2S: 403.1475; found: 403.1467.

Product 6b

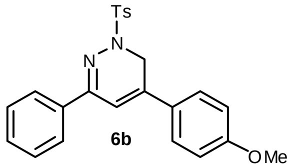

Yield of **6b** : 76% as a white solid. ^{1}H NMR (600 MHz, $CDCl_3$) δ (ppm) = 7.93 (d, *J* = 8.2 Hz, 2H), 7.78 – 7.77 (m, 2H), 7.51 (d, *J* = 8.8 Hz, 2H), 7.41 – 7.39 (m, 3H), 7.35 (d, *J* = 8.1 Hz, 2H), 6.96 (d, *J* = 8.8 Hz, 2H), 6.71 (s, 1H), 4.30 (s, 2H), 3.86 (s, 3H), 2.42 (s, 3H). ^{13}C NMR (100 MHz, $CDCl_3$) δ (ppm) = 160.9, 151.0, 144.3, 139.4, 135.3, 132.1, 129.6, 129.5, 128.8, 128.4, 127.6, 127.0, 125.9, 114.3, 110.6, 55.4, 43.3, 21.5. M.P.: 149.6 – 150.7 ^{o}C. IR (in KBr): 1602, 1512, 1367, 1252, 1174, 1093 cm^{-1}. HRMS (EI): m/z $[M + H]^+$ calcd for $C_{24}H_{23}N_2O_3S$: 419.1424; found: 419.1424.

Product 6c

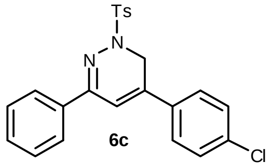

Yield of **6c** : 75% as a white solid. ^{1}H NMR (600 MHz, $CDCl_3$) δ (ppm) = 7.94 (d, *J* = 8.2 Hz, 2H), 7.79 – 7.77 (m, 2H), 7.50 (d, *J* = 8.5 Hz, 2H), 7.45 – 7.42 (m, 5H), 7.39 (s, 2H), 6.79 (s, 1H), 4.32 (s, 2H), 2.45 (s, 3H). ^{13}C NMR (100 MHz, $CDCl_3$) δ (ppm) = 150.5, 144.4, 138.4, 135.6, 134.8, 133.7, 131.9, 129.7, 129.5, 129.0, 128.7, 128.4, 126.7, 125.8, 112.8, 43.1, 21.5. M.P.: 159.0 – 159.7 ^{o}C. IR (in KBr): 1626, 1493, 1442, 1355, 1174, 1093 cm^{-1}. HRMS (EI): m/z $[M + H]^+$ calcd for $C_{23}H_{20}ClN_2O_2S$: 423.0929; found: 423.0929.

Product 6d

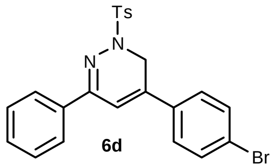

Yield of **6d** : 81% as a green solid. ^{1}H NMR (600 MHz, $CDCl_3$) δ (ppm) = 7.92 (d, *J* = 8.2 Hz, 2H), 7.77 – 7.75 (m, 2H), 7.57 (d, *J* = 8.5 Hz, 2H), 7.42 – 7.41 (m, 5H), 7.36 (d, *J* = 8.2 Hz, 2H), 6.78 (s, 1H), 4.30 (s, 2H), 2.43 (s, 3H). ^{13}C NMR (100 MHz, $CDCl_3$) δ (ppm) = 150.6, 144.4, 138.5, 134.8, 134.2, 132.0, 132.0, 129.8, 129.6, 128.8, 128.5, 127.0, 125.8, 124.0, 112.9, 43.1, 21.6. M.P.: 164.3 – 165.5 ^{o}C. IR (in KBr): 1628, 1490, 1442, 1355, 1173, 1097 cm^{-1}. HRMS (EI): m/z $[M + Na]^+$ calcd for $C_{23}H_{19}BrN_2O_2SNa$: 489.0243; found: 489.0250.

Product 6e

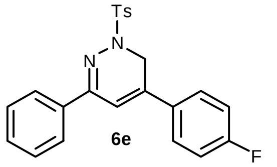

Yield of **6e** : 72% as a white solid. ^{1}H NMR (600 MHz, $CDCl_3$) δ (ppm) = 7.93 (d, *J* = 8.2 Hz, 2H), 7.77 – 7.76 (m, 2H), 7.55 – 7.52 (m, 2H), 7.42 – 7.40 (m, 3H), 7.36 (d, *J* = 8.1 Hz, 2H), 7.14 (t, *J* = 8.5 Hz, 2H), 6.74 (s, 1H), 4.30 (s, 2H), 2.43 (s, 3H). ^{13}C NMR (100 MHz, $CDCl_3$) δ (ppm) = 163.4 (d, *J* = 249.7 Hz), 150.6, 144.4, 138.6, 134.9, 132.0, 131.5 (d, *J* = 3.2 Hz),

129.7, 129.5, 128.7, 128.4, 127.4 (d, *J* = 8.3 Hz), 125.8, 116.0 (d, *J* = 21.7 Hz), 112.4, 43.3, 21.5. ^{19}F NMR (376 MHz, $CDCl_3$) δ(ppm) = -111.3 (s, 1F). M.P.: 161.4 – 162.5 °C. IR (in KBr): 1622, 1510, 1342, 1229, 1168, 1093 cm^{-1}. HRMS (EI): m/z [M + H]$^+$ calcd for $C_{23}H_{20}FN_2O_2S$: 407.1224; found: 407.1224.

Product 6f

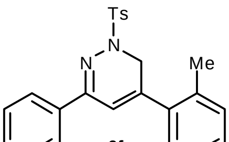

6f

Yield of **6f** : 51% as a white solid. ^{1}H NMR (600 MHz, $CDCl_3$) δ (ppm) = 7.89 (d, *J* = 8.1 Hz, 2H), 7.77 – 7.74 (m, 2H), 7.41 – 7.40 (m, 3H), 7.35 (d, *J* = 8.0 Hz, 2H), 7.30 - 7.28 (m, 2H), 7.23 (t, *J* = 7.3 Hz, 1H), 7.19 (d, *J* = 7.5 Hz, 1H), 6.45 (s, 1H), 4.10 (s, 2H), 2.43 (s, 3H), 2.42 (s, 3H). ^{13}C NMR (100 MHz, $CDCl_3$) δ (ppm) = 151.1, 144.2, 142.2, 137.2, 135.2, 134.8, 132.0, 130.8, 129.7, 129.5, 128.9, 128.7, 128.4, 128.0, 126.0, 125.8, 116.3, 45.1, 21.5, 20.4. M.P.: 139.5 – 140.3 °C. IR (in KBr): 1640, 1594, 1442, 1361, 1175, 1090 cm^{-1}. HRMS (EI): m/z [M + H]$^+$ calcd for $C_{24}H_{23}$ N_2O_2S: 403.1475; found: 403.1476.

Product 6g

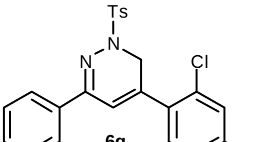

6g

Yield of **6g** : 57% as a white solid. ^{1}H NMR (600 MHz, $CDCl_3$) δ (ppm) = 7.90 (d, *J* = 8.0 Hz, 2H), 7.76 – 7.75 (m, 2H), 7.47 (s, 1H), 7.42 – 7.40 (m, 3H), 7.36 (d, *J* = 8.0 Hz, 2H), 7.33 – 7.30 (m, 2H), 6.58 (s, 1H), 4.18 (s, 2H), 2.43 (s, 3H). ^{13}C NMR (100 MHz, $CDCl_3$) δ (ppm) = 150.7, 144.5, 138.4, 135.6, 134.9, 134.5, 133.1, 132.0, 130.9, 130.0, 129.9, 129.6, 128.7, 128.5, 127.6, 125.8, 117.9, 44.4, 21.6. M.P.: 162.6 – 163.3 °C. IR (in KBr): 1643, 1587, 1470, 1368, 1170, 1096 cm^{-1}. HRMS (EI): m/z [M + H]$^+$ calcd for $C_{23}H_{19}Cl_2N_2O_2S$: 457.0539; found: 457.0541.

Product 14

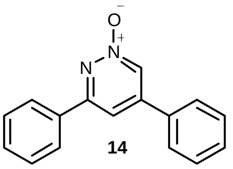

14

Yield of **14** : 70% as a white solid. ^{1}H NMR (600 MHz, $CDCl_3$) δ (ppm) = 8.41 (s, 1H), 8.06 – 8.04 (m, 2H), 7.68 – 7.64 (m, 3H), 7.59 – 7.56 (m, 3H), 7.54 – 7.52 (m, 3H). ^{13}C NMR (150 MHz, $CDCl_3$) δ (ppm) = 159.1, 147.6, 133.8, 133.0, 130.9, 130.9, 129.9, 129.6, 129.0, 127.2, 126.8, 112.1. M.P.: 144.1 – 145.7 °C. IR (in KBr): 1642, 1574, 1532, 1376, 1230, 1103 cm^{-1}. HRMS (EI): m/z [M + H]$^+$ calcd for $C_{16}H_{13}N_2O$: 249.1022; found: 249.1025.

Product 15a

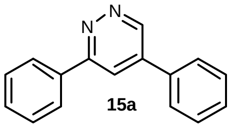

15a

Yield of **15a** : 90% as a white solid. ^{1}H NMR (400 MHz, $CDCl_3$) δ (ppm) = 9.42 (d, *J* = 2.0 Hz, 1H), 8.15 (d, *J* = 6.8 Hz, 2H), 8.01 (d, *J* = 2.0 Hz, 1H), 7.74 (d, *J* = 6.8 Hz, 2H), 7.58 – 7.53 (m, 6H). ^{13}C NMR (100 MHz, $CDCl_3$) δ (ppm) = 159.4, 148.4, 139.0, 136.4, 134.8, 130.1, 130.0, 129.5, 129.0, 127.2, 127.1, 121.0. M.P.: 143.1 – 143.5 °C. IR (in KBr): 1591, 1493, 1448, 1402, 1078, 693 cm^{-1}. HRMS (EI): m/z [M + H]$^+$ calcd for $C_{16}H_{13}N_2$: 233.1073; found: 233.1068.

Product 15b

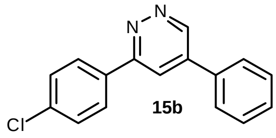

15b

Yield of **15b** : 89% as a white solid. ^{1}H NMR (600 MHz, $CDCl_3$) δ (ppm) = 9.43 (s, 1H), 8.11 (d, *J* = 7.2 Hz, 2H), 7.98 (s, 1H), 7.74 (d, *J* = 7.6 Hz, 2H), 7.59 – 7.55 (m, 3H), 7.53 (d, *J* =

7.1 Hz, 2H). ^{13}C NMR (100 MHz, $CDCl_3$) δ (ppm) = 158.3, 148.6, 139.2, 136.4, 134.8, 134.6, 130.2, 129.5, 129.2, 128.4, 127.1, 120.7. M.P.: 202.7 – 203.6 ^{o}C. IR (in KBr): 1674, 1593, 1488, 1446, 1386, 1089 cm^{-1}. HRMS (EI): m/z $[M + H]^+$ calcd for $C_{16}H_{12}ClN_2$: 267.0684; found: 267.0677.

Product 15c

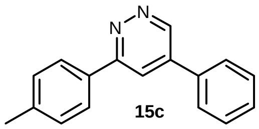

Yield of **15c** : 86% as a white solid. ^{1}H NMR (400 MHz, $CDCl_3$) δ (ppm) = 9.39 (d, *J* = 1.9 Hz, 1H), 8.05 (d, *J* = 8.1 Hz, 2H), 7.98 (d, *J* = 1.9 Hz, 1H), 7.73 (d, *J* = 6.6 Hz, 2H), 7.59 – 7.53 (m, 3H), 7.36 (d, *J* = 8.0 Hz, 2H), 2.45 (s, 3H). ^{13}C NMR (100 MHz, $CDCl_3$) δ (ppm) = 159.3, 148.2, 140.3, 138.9, 134.9, 133.5, 130.0, 129.7, 129.4, 127.1, 127.0, 120.7, 21.3. M.P.: 150.3 – 151.8 ^{o}C. IR (in KBr): 1588, 1517, 1494, 1391, 1182, 1110 cm^{-1}. HRMS (EI): m/z $[M + H]^+$ calcd for $C_{17}H_{15}N_2$: 247.1230; found: 247.1230.

Product 15d

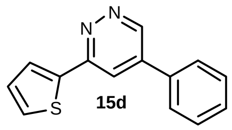

Yield of **15d** : 90% as a white soild. ^{1}H NMR (600 MHz, $CDCl_3$) δ (ppm) = 9.31 (s, 1H), 7.92 (s, 1H), 7.77 (d, *J* = 3.2 Hz, 1H), 7.72 (d, *J* = 7.1 Hz, 2H), 7.59 – 7.53 (m, 4H), 7.20 (t, *J* = 4.2 Hz, 1H). ^{13}C NMR (100 MHz, $CDCl_3$) δ (ppm) = 155.0, 148.3, 140.6, 139.0, 134.6, 130.2, 129.6, 129.4, 128.2, 127.1, 126.4, 119.2. M.P.: 135.7 – 136.3 ^{o}C. IR (in KBr): 1650, 1585, 1434, 1373, 1245, 1102 cm^{-1}. HRMS (EI): m/z $[M + H]^+$ calcd for $C_{14}H_{11}N_2S$: 239.0637; found: 239.0646.

Product 15e

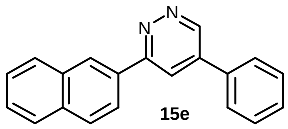

Yield of **15e** : 94% as a white solid. ^{1}H NMR (600 MHz, $CDCl_3$) δ (ppm) = 9.45 (d, *J* = 1.9 Hz, 1H), 8.64 (s, 1H), 8.32 – 8.31 (m, 1H), 8.16 (d, *J* = 2.0 Hz, 1H), 8.03 (d, *J* = 8.6 Hz, 1H), 8.01 – 7.99 (m, 1H), 7.93 – 7.92 (m, 1H), 7.78 (d, *J* = 7.1 Hz, 2H), 7.61 – 7.56 (m, 5H). ^{13}C NMR (100 MHz, $CDCl_3$) δ (ppm) = 159.2, 148.4, 139.1, 134.8, 134.0, 133.6, 133.3, 130.0, 129.5, 128.8, 128.8, 127.7, 127.1, 127.1, 127.0, 126.6, 124.2, 121.1. M.P.: 176.7 – 177.2 ^{o}C. IR (in KBr): 1639, 1381, 1103, 907, 826, 762 cm^{-1}. HRMS (EI): m/z $[M + H]^+$ calcd for $C_{20}H_{15}N_2$: 283.1230; found: 283.1236.

Product 15f

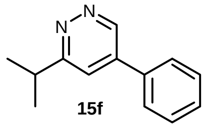

Yield of **15f** : 87% as a colorless oil. ^{1}H NMR (400 MHz, $CDCl_3$) δ (ppm) = 9.32 (d, *J* = 2.0 Hz, 1H), 7.68 – 7.66 (m, 2H), 7.57 – 7.50 (m, 4H), 3.43 – 3.36 (m, 1H), 1.45 (s, 3H), 1.43 (s, 3H). ^{13}C NMR (100 MHz, $CDCl_3$) δ (ppm) = 168.3, 148.1, 138.7, 135.0, 129.8, 129.4, 127.0, 121.2, 34.9, 22.4. IR (in KBr): 1591, 1497, 1380, 1331, 1067, 765 cm^{-1}. HRMS (EI): m/z $[M + H]^+$ calcd for $C_{13}H_{15}N_2$: 199.1230; found: 199.1227.

Product 15g

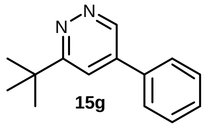

Yield of **15g** : 76% as a white solid. ^{1}H NMR (400 MHz, $CDCl_3$) δ (ppm) = 9.31 (d, *J* = 2.0 Hz, 1H), 7.68 (d, *J* = 1.5 Hz, 1H), 7.66 (d, *J* = 1.9 Hz, 2H), 7.57 – 7.51 (m, 3H), 1.52 (s, 9H). ^{13}C NMR (100 MHz, $CDCl_3$) δ (ppm) = 170.2, 147.7, 138.5, 135.4, 129.7, 129.4, 127.1, 120.1, 37.0, 30.0. M.P.: 87.1 – 87.9 ^{o}C. IR (in KBr): 1646, 1581, 1492, 1371, 1259, 1154 cm^{-1}. HRMS (EI): m/z $[M + H]^+$ calcd for $C_{14}H_{17}N_2$: 213.1386; found: 213.1387.

Product 15h

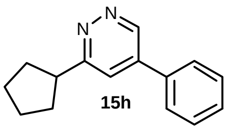

15h

Yield of **15h** : 85% as a white solid. ^{1}H NMR (600 MHz, $CDCl_3$) δ (ppm) = 9.30 (s, 1H), 7.66 (d, *J* = 7.2 Hz, 2H), 7.55 – 7.50 (m, 4H), 3.49 – 3.43 (m, 1H), 2.25 – 2.20 (m, 2H), 1.91 – 1.87 (m, 4H), 1.78 (s, 2H). ^{13}C NMR (100 MHz, $CDCl_3$) δ (ppm) = 167.0, 148.0, 138.5, 135.0, 129.7, 129.3, 127.0, 122.1, 46.1, 33.5, 25.8. M.P.: 83.6 – 84.8 °C. IR (in KBr): 1637, 1586, 1445, 1371, 1316, 1077 cm^{-1}. HRMS (EI): m/z [M + H]$^{+}$ calcd for $C_{15}H_{17}N_2$: 225.1386; found: 225.1386.

Product 15i

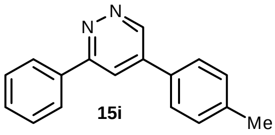

15i

Yield of **15i** : 81% yield as a white solid. ^{1}H NMR (600 MHz, $CDCl_3$) δ (ppm) = 9.41 (d, *J* = 2.0 Hz, 1H), 8.14 (d, *J* = 7.1 Hz, 2H), 7.99 (d, *J* = 2.0 Hz, 1H), 7.65 (d, *J* = 8.0 Hz, 2H), 7.57 – 7.51 (m, 3H), 7.38 (d, *J* = 7.9 Hz, 2H), 2.46 (s, 3H). ^{13}C NMR (150 MHz, $CDCl_3$) δ (ppm) = 159.3, 148.3, 140.4, 138.9, 136.5, 131.8, 130.2, 130.0, 129.0, 127.2, 126.9, 120.6, 21.3. M.P.: 143.2 – 143.5 °C. IR (in KBr): 1591, 1516, 1399, 1369, 1077, 816 cm^{-1}. HRMS (EI): m/z [M + H]$^{+}$ calcd for $C_{17}H_{15}N_2$: 247.1230; found: 247.1224.

Product 15j

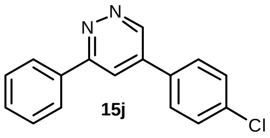

15j

Yield of **15j** : 89% as a white solid. ^{1}H NMR (600 MHz, $CDCl_3$) δ (ppm) = 9.39 (s, 1H), 8.14 (d, *J* = 7.7 Hz, 2H), 7.97 (s, 1H), 7.68 (d, *J* = 7.6 Hz, 2H), 7.58 – 7.52 (m, 5H). ^{13}C NMR (100 MHz, $CDCl_3$) δ (ppm) = 159.4, 148.0, 137.9, 136.4, 136.2, 133.2, 130.2, 129.8, 129.0, 128.4, 127.2, 120.7. M.P.: 196.1 – 196.7 °C. IR (in KBr): 1648, 1595, 1491, 1367, 1092, 833 cm^{-1}. HRMS (EI): m/z [M + H]$^{+}$ calcd for $C_{16}H_{12}ClN_2$: 267.0684; found: 267.0684.

Product 15k

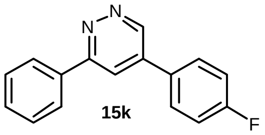

15k

Yield of **15k** : 80% as a white solid. ^{1}H NMR (600 MHz, $CDCl_3$) δ (ppm) = 9.39 (s, 1H), 8.14 (d, *J* = 7.8 Hz, 2H), 7.97 (s, 1H), 7.74 – 7.72 (m, 2H), 7.58 – 7.52 (m, 3H), 7.28 (d, *J* = 8.5 Hz, 2H). ^{13}C NMR (100 MHz, $CDCl_3$) δ (ppm) = 164.0 (d, *J* = 249.5 Hz), 159.4, 148.2, 138.0, 136.3, 131.0 (d, *J* = 3.4 Hz), 130.2, 129.1, 129.0, 127.2, 120.8, 116.7 (d, *J* = 21.8 Hz). ^{19}F NMR (376 MHz, $CDCl_3$) δ(ppm) = -111.7 (s, 1F). M.P.: 176.3 – 176.5 °C. IR (in KBr): 1653, 1604, 1513, 1390, 1233, 1104 cm^{-1}. HRMS (EI): m/z [M + H]$^{+}$ calcd for $C_{16}H_{12}FN_2$: 251.0979; found: 251.0979.

Product 15l

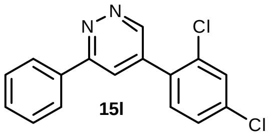

15l

Yield of **15l** : 87% as a white solid. ^{1}H NMR (600 MHz, $CDCl_3$) δ (ppm) = 9.24 (d, *J* = 2.0 Hz, 1H), 8.13 (d, *J* = 8.2 Hz, 2H), 7.93 (d, *J* = 2.0 Hz, 1H), 7.61 (d, *J* = 2.0 Hz, 1H), 7.57 – 7.53 (m, 3H), 7.45 – 7.43 (m, 1H), 7.37 (d, *J* = 8.2 Hz, 1H). ^{13}C NMR (100 MHz, $CDCl_3$) δ (ppm) = 159.0, 149.7, 137.0, 136.3, 136.0, 133.3, 132.9, 131.6, 130.4, 130.3, 129.1, 128.0, 127.2, 123.8. M.P.: 157.1 – 157.9 °C. IR (in KBr): 1650, 1592, 1384, 1359, 1106, 1055, 851 cm^{-1}. HRMS (EI): m/z [M + H]$^{+}$ calcd for $C_{16}H_{11}Cl_2N_2$: 301.0294; found: 301.0288.

Product 17a

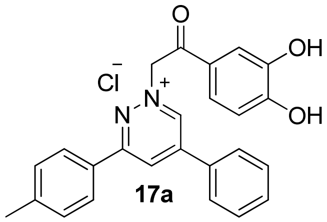
17a

Yield of **17a** : 85% as a gray solid. ^{1}H NMR (400 MHz, DMSO-d^6) δ (ppm) = 10.58 (s, 1H), 10.51 (s, 1H), 9.74 (s, 1H), 9.44 (s, 1H), 8.27 (d, *J* = 7.0 Hz, 4H), 7.73 (s, 3H), 7.58 (d, *J* = 8.3 Hz, 1H), 7.51 (s, 2H), 7.48 (s, 1H), 7.03 (d, *J* = 8.3 Hz, 1H), 6.72 (s, 2H), 2.44 (s, 3H). ^{13}C NMR (100 MHz, DMSO-d^6) δ (ppm) = 188.0, 160.8, 152.7, 147.7, 145.9, 143.0, 132.5, 130.7, 130.0, 129.7, 129.2, 128.7, 128.3, 128.1, 125.0, 122.1, 115.7, 115.2, 70.0, 21.1. M.P.: 252.8 – 253.3 °C. IR (in KBr): 3220, 1675, 1604, 1516, 1328, 1277 cm^{-1}. HRMS (EI): m/z $[M-Cl^-]^+$ calcd for $C_{25}H_{21}N_2O_3$: 397.1547; found: 397.1547.

Product 17b

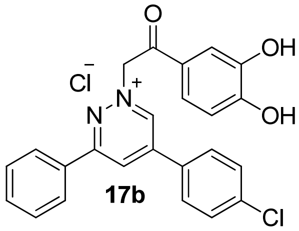
17b

Yield of **17b**: 76% as a gray solid. ^{1}H NMR (600 MHz, DMSO-d^6) δ (ppm) = 10.75 (s, 1H), 10.57 (s, 1H), 9.78 (s, 1H), 9.51 (s, 1H), 8.36 (t, *J* = 8.4 Hz, 4H), 7.82 (d, *J* = 8.4 Hz, 2H), 7.73 – 7.67 (m, 3H), 7.58 (d, *J* = 8.3 Hz, 1H), 7.53 (s, 1H), 7.06 (d, *J* = 8.3 Hz, 1H), 6.78 (s, 2H). ^{13}C NMR (100 MHz, DMSO-d^6) δ (ppm) = 187.7, 160.9, 152.6, 147.9, 145.7, 144.8, 137.5, 132.4, 131.9, 130.0, 129.6, 129.5, 129.3, 128.1, 125.0, 122.0, 115.6, 115.1, 69.8. M.P.: 253.9 – 254.3 °C. IR (in KBr): 3227, 1684, 1605, 1508, 1338, 1366 cm^{-1}. HRMS (EI): m/z $[M-Cl^-]^+$ calcd for $C_{24}H_{18}ClN_2O_3$: 417.1000; found: 417.1001.

Product 17c

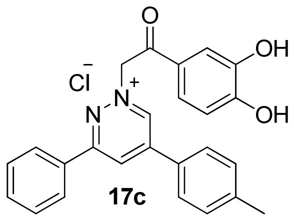
17c

Yield of **17c** : 82% as a gray solid. ^{1}H NMR (400 MHz, DMSO-d^6) δ (ppm) = 10.73 (s, 1H), 10.58 (s, 1H), 9.79 (s, 1H), 9.44 (d, *J* = 10.9 Hz, 1H), 8.35 (d, *J* = 7.7 Hz, 2H), 8.23 (d, *J* = 8.1 Hz, 2H), 7.69 (t, *J* = 8.0 Hz, 3H), 7.58 (d, *J* = 8.4 Hz, 1H), 7.52 (d, *J* = 8.3 Hz, 3H), 7.06 (d, *J* = 8.3 Hz, 1H), 6.78 (s, 2H), 2.44 (s, 3H). ^{13}C NMR (100 MHz, $CDCl_3$) δ (ppm) = 188.0, 160.8, 152.7, 147.8, 145.9, 145.8, 143.1, 132.5, 132.1, 130.6, 129.4, 128.4, 128.2, 128.1, 127.8, 125.0, 122.1, 115.6, 115.1, 70.0, 21.1. M.P.: 246.4 – 246.8 °C. IR (in KBr): 3193, 1687, 1600, 1515, 1392, 1337, 1258 cm^{-1}. HRMS (EI): m/z $[M-Cl^-]^+$ calcd for $C_{25}H_{21}N_2O_3$: 397.1547; found: 397.1546.

Product 17d

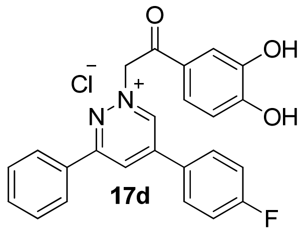
17d

Yield of **17d** : 63% as a gray solid. ^{1}H NMR (600 MHz, DMSO-d^6) δ (ppm) = 10.51 (s, 1H), 10.45 (s, 1H), 9.71 (s, 1H), 9.47 (s, 1H), 8.35 (d, *J* = 8.0 Hz, 4H), 7.75 – 7.67 (m, 3H), 7.62 (t, *J* = 8.7 Hz, 2H), 7.58 (d, *J* = 8.2 Hz, 1H), 7.48 (s, 1H), 7.00 (d, *J* = 8.3 Hz, 1H), 6.69 (s, 2H). ^{13}C NMR (100 MHz, DMSO-d^6) δ (ppm) = 187.7, 164.6 (d, *J* = 250.4 Hz), 160.8, 152.6, 147.9, 145.8, 144.9, 132.4, 132.0, 131.0 (d, *J* = 6.6 Hz), 129.3, 129.1, 128.1, 127.2, 125.0, 122.0, 116.8 (d, *J* = 22.0 Hz), 115.6, 115.1, 69.9. ^{19}F NMR (376 MHz, $CDCl_3$) δ(ppm) = -107.2 (s, 1F). M.P.: 252.1 – 252.8 °C. IR (in KBr): 3195, 1678, 1603, 1557, 1517, 1444, 1250 cm^{-1}. HRMS (EI): m/z $[M-Cl^-]^+$ calcd for $C_{24}H_{18}FN_2O_3$: 401.1296; found: 401.1291.

Product 17e

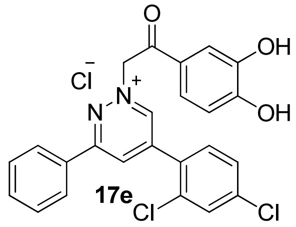

Yield of **17e** : 70% as a gray solid. ^{1}H NMR (600 MHz, DMSO-d^6) δ (ppm) = 10.43 (s, 1H), 10.22 (s, 1H), 9.69 (s, 1H), 9.44 (s, 1H), 8.29 (d, *J* = 7.4 Hz, 2H), 8.05 (s, 1H), 7.95 (d, *J* = 8.3 Hz, 1H), 7.84 (d, *J* = 8.5 Hz, 1H), 7.73 (t, *J* = 7.4 Hz, 1H), 7.68 (t, *J* = 7.5 Hz, 2H), 7.57 (d, *J* = 7.8 Hz, 1H), 7.47 (s, 1H), 6.98 (d, *J* = 8.2 Hz, 1H), 6.71 (s, 2H). ^{13}C NMR (100 MHz, DMSO-d^6) δ (ppm) = 187.7, 160.8, 152.6, 149.8, 145.8, 144.0, 137.0, 133.9, 133.6, 132.7, 132.6, 131.6, 129.9, 129.7, 129.5, 128.5, 128.1, 124.9, 122.1, 115.6, 115.2, 70.1. M.P.: 241.8 – 242.5 ^{o}C. IR (in KBr): 3245, 1683, 1605, 1511, 1463, 1270, 1104 cm^{-1}. HRMS (EI): m/z [M-Cl$^-$]$^+$ calcd for $C_{24}H_{17}Cl_2N_2O_3$: 451.0611; found: 451.0616.

Product 7

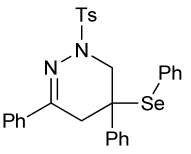

Yield of **7** : as a white solid. ^{1}H NMR (400 MHz, CDCl$_3$) δ (ppm) = 7.69 – 7.65 (m, 4H), 7.34 (s, 3H), 7.30 – 7.14 (m, 12H), 4.03 (d, *J* = 11.6 Hz, 1H), 3.91 (d, *J* = 11.5 Hz, 1H), 3.31 (d, *J* = 18.2 Hz, 1H), 3.07 (d, *J* = 18.1 Hz, 1H), 2.36 (s, 3H). ^{13}C NMR (100 MHz, CDCl$_3$) δ (ppm) = ^{13}C NMR (100 MHz, CDCl$_3$) δ = 146.6, 143.7, 140.8, 137.9, 136.0, 133.3, 129.4, 129.2, 128.6, 128.2, 128.2, 128.0, 127.4, 126.3, 125.9, 125.2, 51.0, 44.0, 35.0, 21.8. HRMS (EI): m/z [M + H]$^+$ calcd for $C_{29}H_{27}N_2O_2SSe$: 547.0953; found: 547.0959.

Supplementary References

1. Clinical and Laboratory Standards Institute/National Committee for Clinical Laboratory Standards: Reference method for broth dilution antifungal susceptibility testing of Yeast. Approved Standard, edn 3; Document M27-A3. Wayne, PA: Clinical and Laboratory Standards Institute; 2009.
2. Clinical and Laboratory Standards Institute/National Committee for Clinical Laboratory Standards: Reference method for broth dilution antifungal susceptibility testing of Yeast. Approved Standard, edn 3; Document M38-A2. Wayne, PA: Clinical and Laboratory Standards Institute; 2008.
3. Lee, C., Yang, W. & Parr, R. G. Development of the Colle-Salvetti correlation-energy formula into a functional of the electron density. *Physical Review B* **37**, 785-789 (1988).
4. Becke, A. D. Density-functional thermochemistry. III. The role of exact exchange. *J. Chem. Phys.* **98**, 5648-5652 (1993).
5. Peverati, R. & Truhlar, D. G. Exchange–correlation functional with good accuracy for both structural and energetic properties while depending only on the density and its gradient. *J. Chem. Theory Comput.* **8**, 2310-2319 (2012).
6. Harvey, J. N. & Aschi, M. Spin-forbidden dehydrogenation of methoxy cation: a statistical view. *Phys. Chem. Chem. Phys.* **1**, 5555-5563 (1999).
7. Poli, R. & Harvey, J. N. Spin forbidden chemical reactions of transition metal compounds. New ideas and new computational challenges. *Chem. Soc. Rev.* **32**, 1-8 (2003).
8.Peverati, R. & Truhlar, D. G. Exchange-Correlation Functional with Good Accuracy for Both Structural and Energetic Properties while Depending Only on the Density and Its Gradient. *J. Chem. Theory Comput.* **8**, 2310-2319 (2012).
9. Cossi, M., Barone, V., Cammi, R. & Tomasi, J. Ab initio study of solvated molecules: a new implementation of the polarizable continuum model. *Chem. Phys. Lett.* **255**, 327-335 (1996).

10. Cancès, E., Mennucci, B. & Tomasi, J. A new integral equation formalism for the polarizable continuum model: Theoretical background and applications to isotropic and anisotropic dielectrics. *J. Chem. Phys.* **107**, 3032 (1997).

11. Barone, V., Cossi, M. & Tomasi, J. Geometry optimization of molecular structures in solution by the polarizable continuum model. *J. Comput. Chem.* **19**, 404-417 (1998).

12. Chen, J.-R., *et al.* Enantioselective synthesis of dihydropyrazoles by formal [4 + 1] cycloaddition of in situ-derived Azoalkenes and sulfur ylides. *J. Am. Chem. Soc.* **134**, 6924-6927 (2012).

13. Hu, X.-Q., *et al.* [4+3] Cycloaddition of in-situ generated azoalkenes with C,N-cyclic azomethine imines: Efficient synthesis of tetrazepine derivatives. *Chem. Commun.* **49**, 7905-7907 (2013).

14. Hu, X.-Q., *et al.* Photocatalytic generation of N-centered hydrazonyl radicals: A strategy for hydroamination of β,γ-unsaturated hydrazones. *Angew. Chem. Int. Ed.* **53**, 12163-12167 (2014).
